# Host-specific assembly of sponge-associated prokaryotes at high taxonomic ranks

**DOI:** 10.1038/s41598-017-02656-6

**Published:** 2017-05-31

**Authors:** Georg Steinert, Sven Rohde, Dorte Janussen, Claudia Blaurock, Peter J. Schupp

**Affiliations:** 1 Institute for Chemistry and Biology of the Marine Environment, Carl-von-Ossietzky University Oldenburg, Wilhelmshaven, Germany; 2 Senckenberg Research Institute and Nature Museum, Frankfurt a.M., Germany; 3 Institute of Biology, Technical University Darmstadt, Darmstadt, Germany; 4 Helmholtz Institute for Functional Marine Biodiversity at the University of Oldenburg, Oldenburg, Germany

## Abstract

Sponges (Porifera) are abundant and diverse members of benthic filter feeding communities in most marine ecosystems, from the deep sea to tropical reefs. A characteristic feature is the associated dense and diverse prokaryotic community present within the sponge mesohyl. Previous molecular genetic studies revealed the importance of host identity for the community composition of the sponge-associated microbiota. However, little is known whether sponge host-specific prokaryotic community patterns observed at 97% 16S rRNA gene sequence similarity are consistent at high taxonomic ranks (from genus to phylum level). In the present study, we investigated the prokaryotic community structure and variation of 24 sponge specimens (seven taxa) and three seawater samples from Sweden. Results show that the resemblance of prokaryotic communities at different taxonomic ranks is consistent with patterns present at 97% operational taxonomic unit level.

## Introduction

Marine sponges (Porifera) are important benthic filter feeding organisms, which inhabit a large range of ecosystems, from the deep sea to continental shelves and shallow reefs, to tropical, temperate and polar regions^1^. Moreover, sponges are known to host diverse and complex prokaryotic communities^2^, and up to 38% of the sponge biomass can be made up of bacteria^3^. The relationships and interactions between sponge hosts and their microbial communities range from being simply a source of food for the sponge, over the synthesis of secondary metabolites for chemical defence, to mutual metabolic interactions^4, 5^. In addition, a growing amount of data, generated by different molecular genetic techniques, shows that sponge host-specific prokaryotic communities are generally stable across varying geographic and temporal gradients^2, 6–9^.

Molecular genetic community studies on the sponge-associated microbiota revealed apparent differential patterns of coexistence. Phylogenetic reconstruction based on 16S rRNA gene data showed sponge-specific clusters of prokaryotic and fungal clades that comprise only sponge-derived sequences^5, 10, 11^. Quantification of prokaryotic cell numbers in sponges provides a differentiation of high microbial abundance (HMA) and low microbial abundance (LMA) sponges^12^, while parallel sequencing technologies emphasized the apparent importance of host identity on prokaryotic composition and diversity^2, 8, 13–15^. However, regardless of the genetic methods used to characterize the sponge-associated microbiota, some bacterial phyla (i.e., Alpha-, Gamma-, Deltaproteobacteria, Chloroflexi, Actinobacteria, Acidobacteria, Nitrospirae and the candidate phylum Poribacteria) are usually found to be dominant within sponges with varying degrees of specificity^4^. In addition, recent quantitative approaches demonstrated divergent distributions of certain prokaryotic taxa that belong to sponge-associated phyla such as Chloroflexi, Actinobacteria, Cyanobacteria, and Poribacteria between HMA and LMA sponges^16–19^.

Nonetheless, while different sponge-species apparently possess these specific dominant prokaryotic phyla, little is known whether the observed beta-diversity patterns (i.e., community variation among individual sponge hosts, here defined as local habitats of prokaryotic alpha diversities) are consistently detectable across taxonomic ranks ranging from OTU to phylum level. It has been proposed that high prokaryotic taxa could constitute ecological meaningful units based on their phylogenetic delineation^20^. Thus an assessment at which taxonomic resolution sponge-host specific patterns appear is a promising approach for a better understanding of symbiotic long-term processes that shaped the sponge as a holobiont. For example, at which taxonomic depth can we detect host-specific patterns that are usually derived from 16S rRNA OTU amplicon libraries.

Therefore, in the present study we investigated whether prokaryotic 16S rRNA diversity patterns at high taxonomic ranks were specific and meaningful for the sponge-associated microbiota. For the analyses we use 24 temperate sponge specimens (seven sponge taxa) and three seawater samples, collected in Tjärnö (Sweden). This data subset was obtained from the first batch of the sponge-related Earth Microbiome Project (EMP - http://www.earthmicrobiome.org/). We hypothesize that even at this small scale (i.e. phylogenetically divergent sponge samples collected from the same locality), patterns of prokaryotic beta-diversity might reflect the putative functional traits and adaptation of sponge-specific deep branching prokaryotic lineages at high taxonomic ranks. For the analysis of beta-diversity among different sponge hosts the available community abundance data was collapsed at OTU level (97% sequence identity) and converted into high taxonomic ranks ranging from species to phylum level. In addition, we used indicator species analysis to assess the host-specificity of sponge-associated prokaryotic communities.

## Results and Discussion

### Prokaryotic community diversity

**Figure 1:**
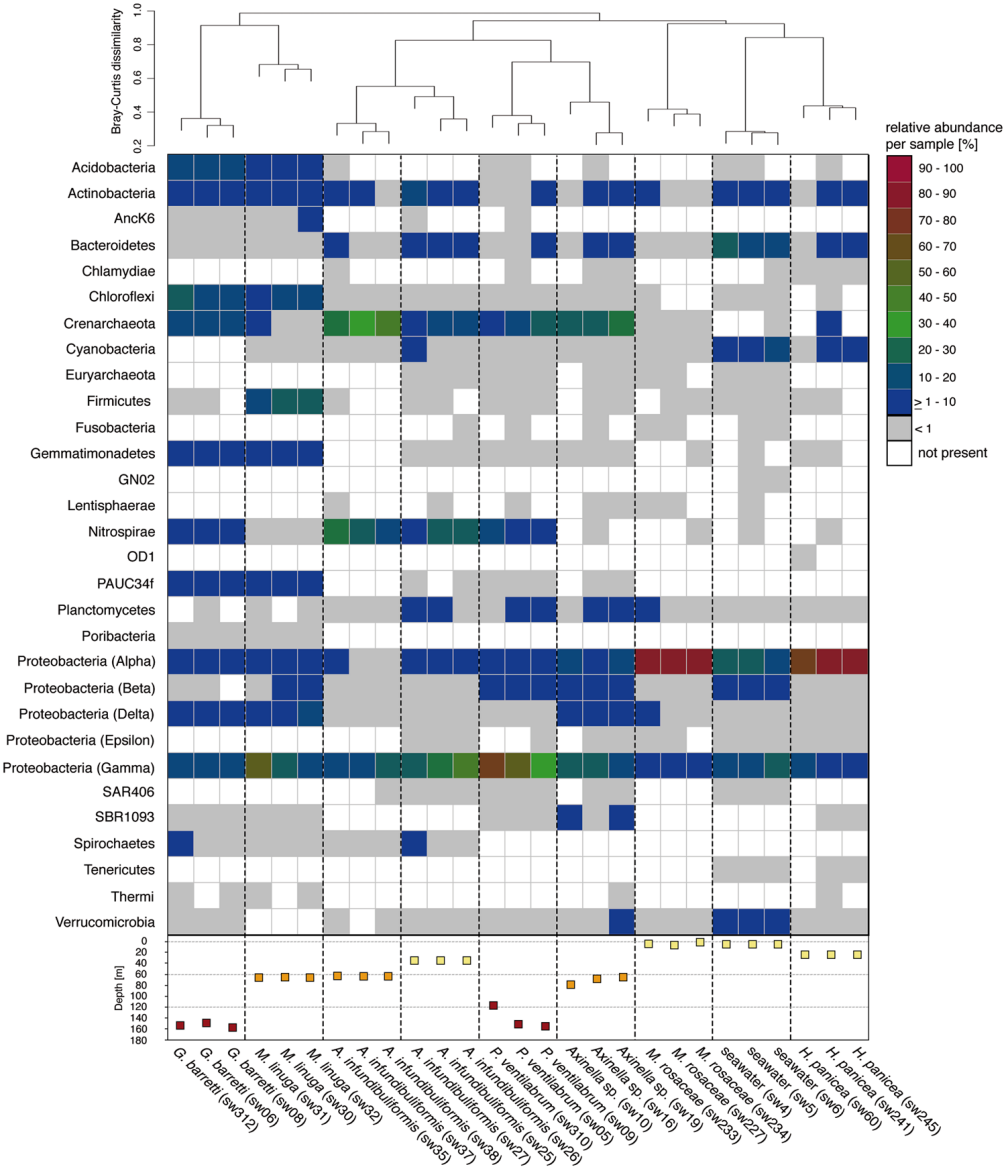
Taxonomic breakdown per sample at phylum level based on relative abundance of assigned 16S rRNA OTUs. Samples arranged by Bray-Curtis dissimilarity as shown by the dendrogram on top. Sampling depth of individual samples is shown below – color code for the hypothetical habitat groups: red for lower twilight, orange for upper twilight, yellow for shallow sites. Individual sample identifiers are given in brackets.

The analysis of 7197 OTUs at 97% 16S rRNA gene sequence identity level (referred as OTU from herein) revealed 24 bacterial and two archaeal phyla associated with 24 sponge and three seawater samples (Fig. 1). Subsurface seawater samples possessed the highest OTU richness and diversity followed by *Geodia barretti* specimens (~154 m sampling depth), while *Myxilla rosacea* (~25 m) showed the lowest OTU richness among all samples (see Table 1 & Supplementary Figure 1). Additionally, *G. barretti* individuals exhibited the highest OTU diversity among all sponge samples followed by the *Mycale lingua* samples, which is consistent with the observed high evenness (see Table 1 & Supplementary Figure 1). Thus, it appears that OTU richness and diversity are influenced by the different microhabitats (i.e. sponge or seawater sample replicates) and not the sampling depth. However, further comparison within individual microhabitats and between varying sampling depths was only possible for *Axinella infundibuliformis*. We observed significant differences among all three alpha diversity indices (Supplementary Table 1). The most abundant prokaryotic phyla among all samples belonged to Proteobacteria (Alpha- and Gamma-), Crenarchaeota, Bacteroidetes, Nitrospirae, Actinobacteria, Chloroflexi, Cyanobacteria and Acidobacteria (Fig. 1). The observed phyla are known to be generally sponge-specific and abundant, but they were differently distributed among the hosts^2, 4^.

**Table 1:** Sample data with sample identifiers (Core ID), the host/sample association and prokaryotic diversity estimates for sample on 16S rRNA OTUs level – showing: OTU richness (S), Shannon diversity index (H), OTU evenness (J), depth and depth zone.

Core ID	Host/Sample	S	H	J	depth	depth zone
SW.25	*Axinella infundibuliformis*	604	6.03	0.94	35	shallow
SW.26	*Axinella infundibuliformis*	617	6.10	0.95	35	shallow
SW.27	*Axinella infundibuliformis*	535	6.02	0.96	35	shallow
SW.35	*Axinella infundibuliformis*	355	5.41	0.92	59	upper twilight
SW.37	*Axinella infundibuliformis*	318	5.17	0.90	60	upper twilight
SW.38	*Axinella infundibuliformis*	352	5.30	0.90	62	upper twilight
SW.10	*Axinella* sp.	211	4.69	0.88	78	upper twilight
SW.16	*Axinella* sp.	627	5.92	0.92	68	upper twilight
SW.19	*Axinella* sp.	576	5.86	0.92	64	upper twilight
SW.06	*Geodia barretti*	1122	6.59	0.94	149	lower twilight
SW.08	*Geodia barretti*	967	6.48	0.94	158	lower twilight
SW.312	*Geodia barretti*	904	6.42	0.94	154	lower twilight
SW.241	*Halichondria panicea*	692	6.03	0.92	7	shallow
SW.245	*Halichondria panicea*	831	6.25	0.93	5	shallow
SW.60	*Halichondria panicea*	453	5.48	0.90	2	shallow
SW.30	*Mycale lingua*	798	6.41	0.96	66	upper twilight
SW.31	*Mycale lingua*	536	5.90	0.94	66	upper twilight
SW.32	*Mycale lingua*	667	6.14	0.94	66	upper twilight
SW.227	*Myxilla rosaceae*	160	4.15	0.82	25	shallow
SW.233	*Myxilla rosaceae*	220	4.69	0.87	25	shallow
SW.234	*Myxilla rosaceae*	138	4.14	0.84	25	shallow
SW.05	*Phakellia ventilabrum*	566	5.85	0.92	155	lower twilight
SW.09	*Phakellia ventilalbrum*	668	6.00	0.92	150	lower twilight
SW.310	*Phakellia ventilabrum*	449	5.40	0.89	117	lower twilight
SW.H2O.4	Seawater	1318	6.76	0.94	4	shallow
SW.H2O.5	Seawater	1445	6.85	0.94	4	shallow
SW.H2O.6	Seawater	1439	6.83	0.94	4	shallow

### Host-specificity of the sponge prokaryotic communities

**Figure 2:**
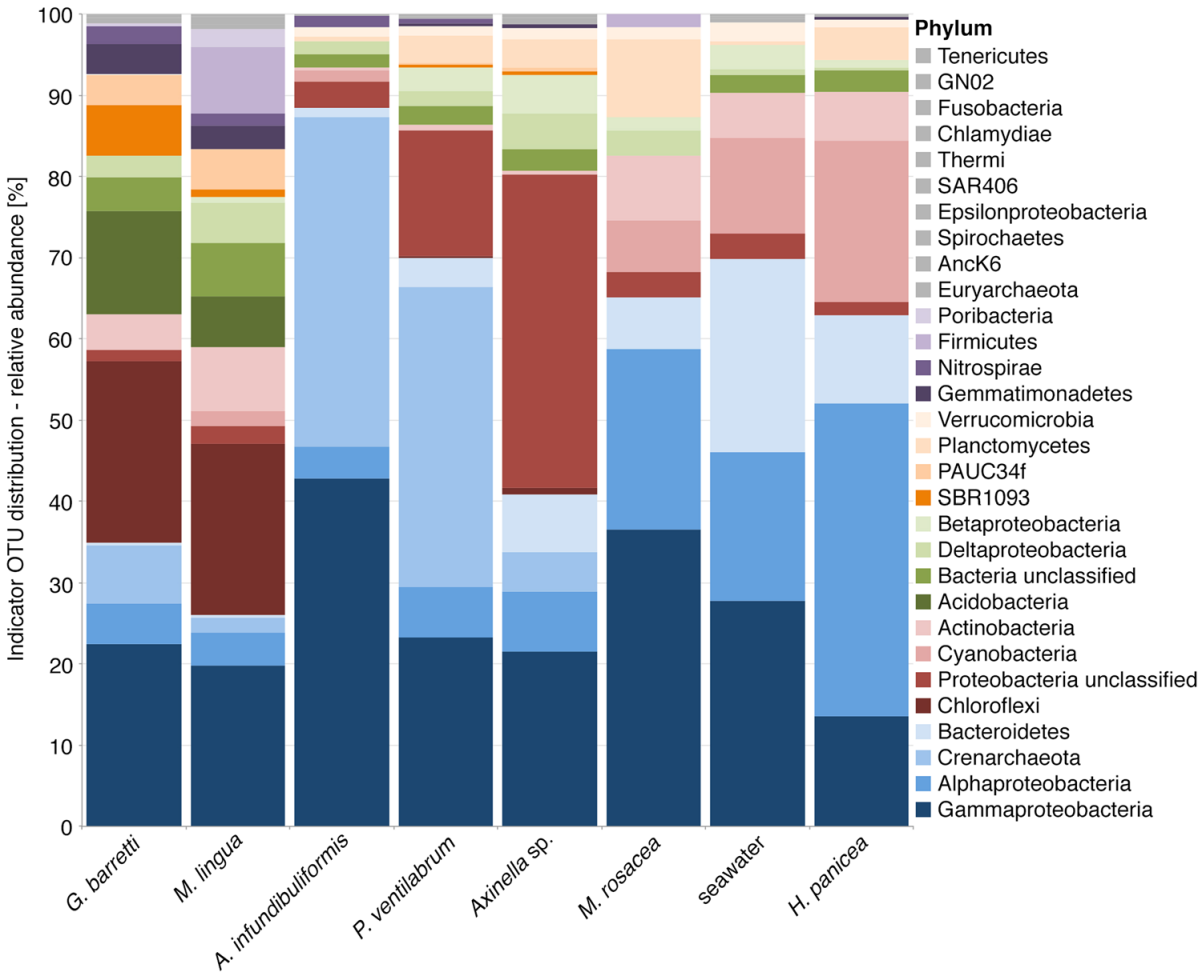
Relative abundance and diversity of significantly correlated indicator species collapsed to phylum level for each triplicate sample group (sponges and seawater). For better clarity only phyla with an abundance ≥0.25% are colorized – a more detailed table with all individual significant OTUs and taxonomic classification down to species level can be found in the Supplementary Table 2.

To investigate the specific associations of prokaryotic taxa with individual microhabitats, an indicator species analysis was performed to infer significant relationships of OTUs and sponge hosts. The resulting indicator OTUs were conflated at phylum level for each sample group (Fig. 2). The resulting compositions of indicator phylotypes are mostly consistent with the observed host-specific patterns observed in the OTU heatmap (Fig. 1, for a complete list of all indicator OTUs and related taxonomic ranks see Supplementary Table 2). Almost every phylum known to be associated with sponges, including Proteobacteria (Alpha- and Gamma-), Firmicutes, Chloroflexi, Actinobacteria, Bacteroidetes, Acidobacteria, Cyanobacteria, Planctomycetes, and Gemmatimonadetes were represented here by significant indicator OTUs (see Supplementary Table 2)^2^. Moreover, the relatively high abundance of Chloroflexi and Acidobacteria corroborates the classification of *G. barretti* as a HMA sponge, since the frequent presence of both of these bacterial phyla are presumably characteristic for HMA sponges^16, 21–23^. The individual comparison of indicator OTUs between shallow and upper twilight *A*. *infundibuliformis* replicates revealed an almost similar phyla composition of the most frequent OTUs (Supplementary Table 3 and Supplementary Figure 2). However, Cyanobacteria were only present in the shallow sponges, whereas Crenarchaeota were more dominant in the upper twilight replicates. This adds to the observations that conspecific sponges from habitats with varying environmental factors (e.g., temperature, light, nutrients) can exhibit diverging prokaryotic community compositions to a certain degree^14, 24–26^. Apparently, certain prokaryotic taxa known to be involved in the nitrogen cycle in sponges, such as *Nitrosopumilus*, *Nitrospira* ^27, 28^, or *Synechococcus* ^29^ are unequally distributed between the different depths in this intraspecific comparison. However, so far there are only scarce reports on the effect of depth^14, 24^ or other environmental parameters^25, 30, 31^ on prokaryotes, of which some potentially contribute to the nitrogen cycle in sponges.

### Consistent patterns of prokaryotic host-specificity

**Figure 3:**
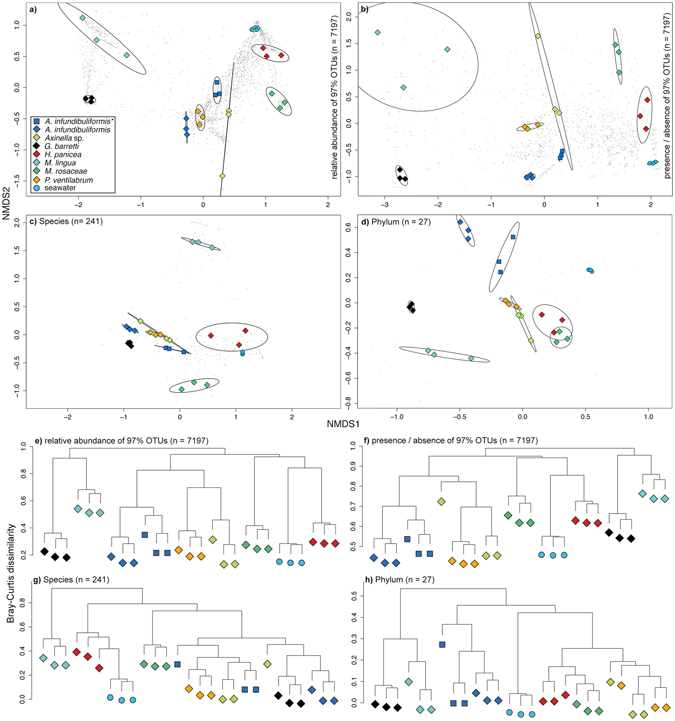
nMDS ordinations based on Bray-Curtis dissimilarities of all samples – (**a**) relative abundance OTUs, (**b**) presence/absence OTUs, (**c**) species, and (**d**) phylum. Available number of taxonomic variables for each dataset is given in brackets. Taxonomic variables are added as grey dots to the background of each ordination. Hypothetical sampling site groups are drawn as dispersion ellipses with a confidence interval of 95%. The cluster diagrams (**e**) to (**h**) show the results of the hierarchical cluster analysis for the same taxonomic ranks and variables as in (**a**) to (**d**). The same color code for each sponge and seawater sample triplicate has been used from (**a**) to (**h**). The asterisk marks the *A. infundibuliformis* replicates collected from the shallow site, while the second set *A. infundibuliformis* replicates were collected from the upper twilight site. Additional nMDS and cluster plots for genus, family, order and class levels can be found in Supplementary Figure 4.

Host-specificity of sponge-associated prokaryotic communities is an overall acknowledged phenomenon in the versatile and complex symbiotic sponge-microbe relationships^4, 5^. Sponge prokaryotic communities can be divided into three groups: a prokaryotic core community that is present in <70% of all sponges, a variable community with changing presence among different sponge species, and a species-specific community, which is specific to its host species^32^. Additionally, a recent large-scale 16S rRNA analysis among 81 sponge species showed that the sponge core prokaryotic community (OTUs present in at least 85% of the replicates for any host species) is comprised primarily by generalist symbionts (present in >62% of all host species), while generally the sponge symbiont communities are characterised by both generalists and specialists (found in only one or a few sponge species)^2^. Especially the latter group is presumably defined by a highly symbiotic relationship to its host and functionally involved in the sponge metabolism^33–35^. In addition to the indicator analysis, the present hierarchical clustering, multivariate analysis of community variation, and nMDS ordination based on OTU dissimilarity estimates (relative abundance & presence/absence) confirmed this apparent host-specificity (Fig. 3a,b,e,f, and Table 2). These host-specific diversity patterns observed at OTU level support the common understanding that host identity shapes sponge-associated prokaryotic communities^2, 8, 13, 14, 36^.

**Table 2:** Individual effects of sample identity (individual sponge and seawater in triplicates) on the prokaryotic community variance across all taxonomic ranks and OTUs (relative abundance & presence/absence). For each taxonomic rank the number of available variables and percentages of unclassified and therefore removed, reads are given next to each taxonomic rank, followed by nMDS stress values. Pairwise comparisons of group mean dispersions (permdisp: betadisper with permutest) are shown as *F* ratio (with degrees of freedom in brackets), correlation coefficient *R* ^*2*^ and *p* value. The same values are available for the permutational analysis of variance (permanova: adonis).

Datasets	Variables	Reads removed [%]	nMDS stress	permdisp			permanova		
				F(7,19)	*R* ^*2*^	*p* value	F(7/19)	*R* ^*2*^	*p* value
OTU relabund	7197	na	0.13	4.72	0.63	**0.01**	12.17	0.82	<**0.001**
OTU pa	7197	na	0.12	4.18	0.61	<**0.001**	4.18	0.61	<**0.001**
Species	241	81.44	0.11	3.66	0.57	**0.01**	9.91	0.79	<**0.001**
Genus	293	51.30	0.11	1.99	0.42	0.11	12.98	0.83	<**0.001**
Family	208	44.73	0.12	1.86	0.41	0.13	15.06	0.85	<**0.001**
Order	133	22.80	0.12	1.77	0.39	0.15	17.79	0.87	<**0.001**
Class	75	1.41	0.11	1.92	0.41	0.12	23.31	0.90	<**0.001**
Phylum	27	>0.01	0.12	2.47	0.48	0.06	22.61	0.89	<**0.001**

**Figure 4:**
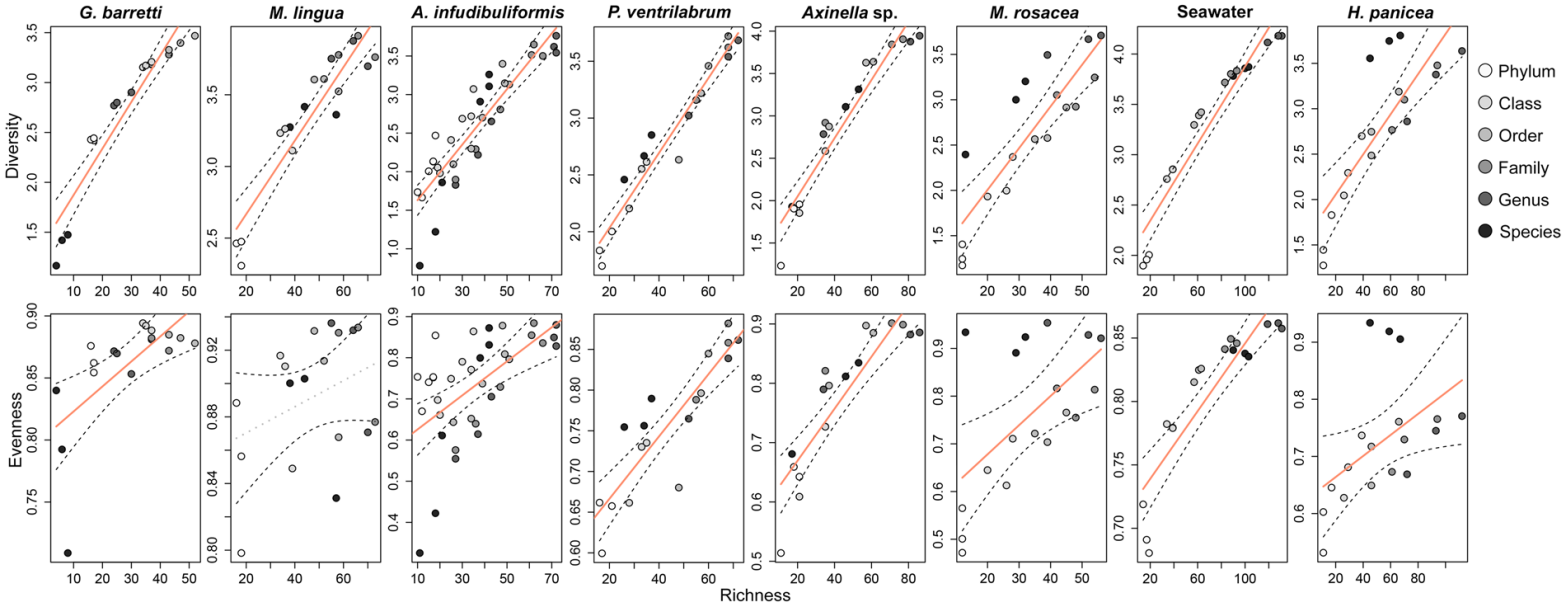
The relationships between richness & diversity and richness & evenness for sponge and seawater associated bacteria across high taxonomic ranks - from phylum to species level for each sponge taxon and the seawater samples. The trends along the taxonomic ranks were modelled with a linear model and plotted as a solid line. The one non-significant trend is shown with a dotted line. More details on the models are in Supplementary Table 6.

However, little is known about the consistency of sponge-microbe associations across different taxonomic ranks. Recent studies analysing marine environmental prokaryotic patterns, from sediment or seawater samples, found indications that prokaryotic community compositions can be meaningful and consistent across broad taxonomic ranks^37, 38^. Since sponge-associated prokaryotic communities exhibit such significant host-specificity, we hypothesize that this relatedness is also consistent at higher taxonomic ranks. Collapsing the present OTU table into individual tables revealed that with each ascending taxonomic rank, from species to phylum level, the number of available taxa decreased, while the number of classified OTUs available for each rank increased (Table 2). However, the species level was an exception, as there were less phylotypes available compared to genus level (n = 241 and n = 293, respectively). This was likely caused by the high percentage of unclassified sequence reads (>81%) at species level. The removal of the accumulating unclassified OTUs from phylum to species level had an apparent effect on alpha diversity measurements for each group (see Supplementary Figure 3). Especially on species level the information did not follow the pattern of increasing richness, diversity and evenness with lower taxonomic ranks. Still, the relationships between richness/diversity and richness/evenness showed overall consistent significant positive linear relationships for each sponge species and seawater (Fig. 4). The only exception was *M. lingua* where evenness was not significantly correlated with richness, and *G. barretti* exhibiting very low richness on species level compared to the other sponges. Despite the differences in richness and the increasing diversity with phylotype richness among high taxonomic ranks multivariate analyses and hierarchical clustering resulted in consistent and significant host-specific beta-diversity patterns from OTU up to phylum level (Fig. 3a–h, Table 2, and see Supplementary Figure 4a–h). The present results suggest that within sponge-associated prokaryotic communities coherent host-specific patterns at the OUT level are prominent enough to be visible at the lowest taxonomic resolution. Multivariate tests on group variation (*permanova*) showed consistent significant host relatedness across all taxonomic ranks (e.g., *R* ^*2*^ = 0.82, *p* < 0.001 on OTU level vs. *R* ^*2*^ = 0.89, *p* < 0.001 on phylum level). Significant group dispersion (*permdisp*) could be observed for the OTU and species datasets (Table 2 & see Supplementary Table 4 for pairwise comparisons of mean group dispersion). Interestingly, the consistent clear grouping of *A. infundibuliformis* replicates based on sampling depth at all taxonomic ranks (Fig. 3a–h and Supplementary Figure 4a–h) and the significant differences of alpha diversity indices within this subset (Supplementary Table 1) were not significant in the multivariate subset analysis at all taxonomic ranks (Supplementary Table 5).

The striking overall consistency of contrasting prokaryotic community patterns at high taxonomic ranks indicates divergent functional and ecological associations with the hosts among prokaryotic phylogenetic deep branches, such as the phylum level. For example, Rua *et al*.^39^ indicated different metabolic strategies between two very different sponge species (HMA vs. LMA specimen) along with host-specific community compositions and diversity. On the contrary, the most abundant prokaryotic taxa within each host-specific community also exhibited functional equivalence to some extent. This finding is in line with functional predictions of the prokaryotic nitrogen metabolism performed among two other tropical HMA/LMA sponges^40^. These observations corroborate current research on the functional roles of prokaryotic symbionts in sponges, which have collectively demonstrated evolutionary convergence and functional equivalence in these complex symbiotic prokaryotic communities^2, 33, 41^.

## Conclusion

The present study shows that prokaryotic sponge-associated communities exhibit consistent host-specific patterns and variation in alpha- and beta-diversity across high prokaryotic taxonomic ranks. In addition, the indicator OTU and the single *A. infundibuliformis* depth-related beta-diversity analyses further emphasize the general observation that host-identity significantly influences the composition of these communities. This suggests that sponge-associated prokaryotic communities exhibit ecological meaningful patterns on high taxonomic ranks. Future studies should focus on the intrinsic differences according to which sponges can be organized into ecological or physiological meaningful groups, such as HMA and LMA members, the abundance of ammonia-oxidizing-bacteria/archaea, environmental niches, or specific chemical compounds. For those analyses, large-scale datasets with great sequencing depth across large geographic and environmental gradients, and an extensive collection of metadata, will help to resolve the present observed consistent patterns in greater detail. Therefore, the sponge-specific EMP data presents an unique opportunity for future meta-analysis on the complex sponge-microbe relationships.

## Methods

We analysed a subset of the EMP sponge data released in 2013. This subset consisted of seven sponge taxa: *Axinella infundibuliformis* (35–62 m)*, Geodia barretti* (149–158 m), *Myxilla rosacea* (25 m), *Mycale lingua* (66 m), *Phakellia ventilabrum* (117–155 m), *Halichondria panicea* (2–7 m), one sponge taxon classified to genus level *Axinella* sp., (64–78 m) and one seawater sample (4 m). Hence, each sponge taxon and the seawater sample was taken in triplicates from individual depths, with the exception of the two *A. infundibuliformis* triplicates taken from two different depths: a) 35 m and b) 59–62 m (Table 1). In total 24 sponge specimens were collected, all belonging to the class Demospongiae. Slight deviations in sampling depth within each triplicate occurred due to varying technical constraints of the three sampling methods. Marine sponges were collected by snorkeling, SCUBA diving and by using a remotely automated vehicle (Sperre Sub-Fighter ROV) in a confined area of Kosterjforden bay (Tjärnö, Sweden, 58°52′53.1“N 11°07′17.5“E) in September 2012. Sponges were initially identified morphologically on location and dissected at the Tjärnö Marine Biology Laboratory field station. Obtained pieces of sponge body (encompassing internal and external parts) were immediately freeze-dried until further processing. In addition, smaller pieces of sponge body or larger parts of whole sponges were also freeze-dried and stored in ethanol 96% for additional morphological classification at the Senckenberg Institute (Frankfurt, Germany) to verify the initial sponge identities. To this end, skeletal preparation and histology were done according to the standard procedures^42, 43^. DNA extraction, Illumina MiSeq sequencing (V4 region of the 16S rRNA gene, using the 515 F/806 R archaeal/bacterial primer pair^44^) and raw sequence quality control of the samples analysed in the present study were carried out by the EMP collaborators^2^ (see Supplementary Material Section 2 for further details regarding the EMP dataset, sequence processing, and quality control).

The subset of the 27 samples described above obtained from the EMP sponge dataset (the full EMP dataset containing all processed sequences can be downloaded from the following portal: http://qiita.microbio.me - Study ID 10346) consisted of 332244 reads and 7201 OTUs in total. Four OTUs classified as *unknown* were removed prior to the analyses, resulting in a final number of 7197 OTUs. The EMP Greengenes classification (97% OTUs, 60% identity cut-off) was used as reference taxonomy. All data processing and subsequent analyses, e.g. alpha- & beta-diversity estimates and sequence statistics (i.e., OTU richness *S*, Shannon diversity index *H* and Pielou’ s evenness *J*) were executed in R v.3.0.3 with the vegan package unless stated otherwise^45, 46^. For beta-diversity analyses (i.e., betadisper/permanova & adonis) all samples were categorized by host-identity. Hypothetical habitat definitions based on the approximate prevailing daylight conditions of the sampling sites (shallow photic zone: 1–35 m; upper twilight zone: 35–120 m; lower twilight zone: 120–160 m) and sample type (i.e., host taxon or seawater) were used to overlay the samples in the generated ordination plots with ellipses. In addition to the full OTU table, individual amplicon abundance datasets were compiled in R by summing up the sequence reads for all available taxa and removing OTUs without approximate taxonomic classification from phylum to species level based on the available Greengenes taxonomy (see Supplementary Material Section 3 & Supplementary Table 7 for further details regarding OTU table processing including the applied R script and basic alpha- and beta-diversity analyses). Finally, at each taxonomic rank the absolute amplicon community data was standardized using *decostand* (method = ‘hellinger’).

The univariate relationships between richness/diversity and richness/evenness among all taxonomic ranks were explored for each sponge species and seawater with linear models in R using the base function *lm*. Multivariate analyses based on the host identity and non-metric multidimensional scaling (NMDS) on all datasets (OTUs, species, genus, family, order, class, phylum) were performed using the functions permutest.betadisper, permutational multivariate analysis of variance (*adonis*) and *metaMDS* (Bray-Curtis) functions of the vegan package. For presence/absence analysis the OTU abundance dataset was transformed via *decostand* (method = “pa”) and Jaccard distances were calculated using the *vegdist* function. Significance tests were based on 1000 permutations for all performed analyses.

The relative OTU abundance and distribution on phylum level was plotted as heatmap using JColorGrid^47^. Hierarchical clustering of OTUs (relative abundance & presence/absence) and rank-specific datasets were performed using *vegdist* (Bray-Curtis) and *hclust* (method = ‘average’). In addition to the individual cluster diagrams, the relative abundance OTU plot was added to the heatmap. For the OTU based indicator analysis, which utilized the *multipatt* (func = ‘IndVal.g’, duleg = TRUE, 1000 permutations) algorithm in the indicspecies package^48^, each sample triplicate (sponges and seawater) was pooled and then subsequently analysed.

In addition to the uni-, multivariate, and indicator analyses among all available microhabitats (i.e., sponge-species triplicates and seawater samples), hellinger-transformed subsets (from OTU to phylum level) of the conspecific *A. infundibuliformis* sponge replicates were created based on the two hypothetical sampling zones (i.e., shallow and upper twilight). These two groups were analysed and compared via permutest.betadisper, *adonis*, and *multipatt* as described above. Moreover, the R function *aov* has been used for a statistical comparison of the alpha diversity indices.

### Data Availability

The OTU (absolute) abundance table combined with the Greengenes classification for each individual OTU can be accessed via the Figshare online repository (https://dx.doi.org/10.6084/m9.figshare.3470696). The metadata connected to the individual samples can be accessed via the Figshare online repository (https://dx.doi.org/10.6084/m9.figshare.4299629.v1). T

## Electronic supplementary material

Supplementary material for: “Host-specific assembly of sponge-associated prokaryotes at high taxonomic ranks
