# Supplementary material for: Host-specific assembly of sponge-associated prokaryotes at high taxonomic ranks
Source: Sci Rep. 2017 May 31;7:2542. doi: 10.1038/s41598-017-02656-6 (PMC5451456; doi:10.1038/s41598-017-02656-6)
Supplement: Supplementary file 1 — Supplementary material for: “Host-specific assembly of sponge-associated prokaryotes at high taxonomic ranks [file 41598_2017_2656_MOESM1_ESM.pdf]

Supplementary material for: “Host-specific assembly of sponge-associated prokaryotes at high taxonomic ranks"

Georg Steinert[1], Sven Rohde[1], Dorte Janussen[2], Claudia Blaurock[3], Peter J. Schupp[1*]

[1]Institute for Chemistry and Biology of the Marine Environment, Carl-von-Ossietzky University Oldenburg, Wilhelmshaven, Germany (georg.steinert@iba-science.de, sven.rohde@uni-oldenburg.de, peter.schupp@uni-oldenburg.de)
[2]Senckenberg Research Institute and Nature Museum, Frankfurt a.M., Germany (dorte.janussen@senckenberg.de)
[3]Institute of Biology, Technical University Darmstadt (blaurock-claudia@t-online.de)

**Section 1: Supplementary Figures and Tables**

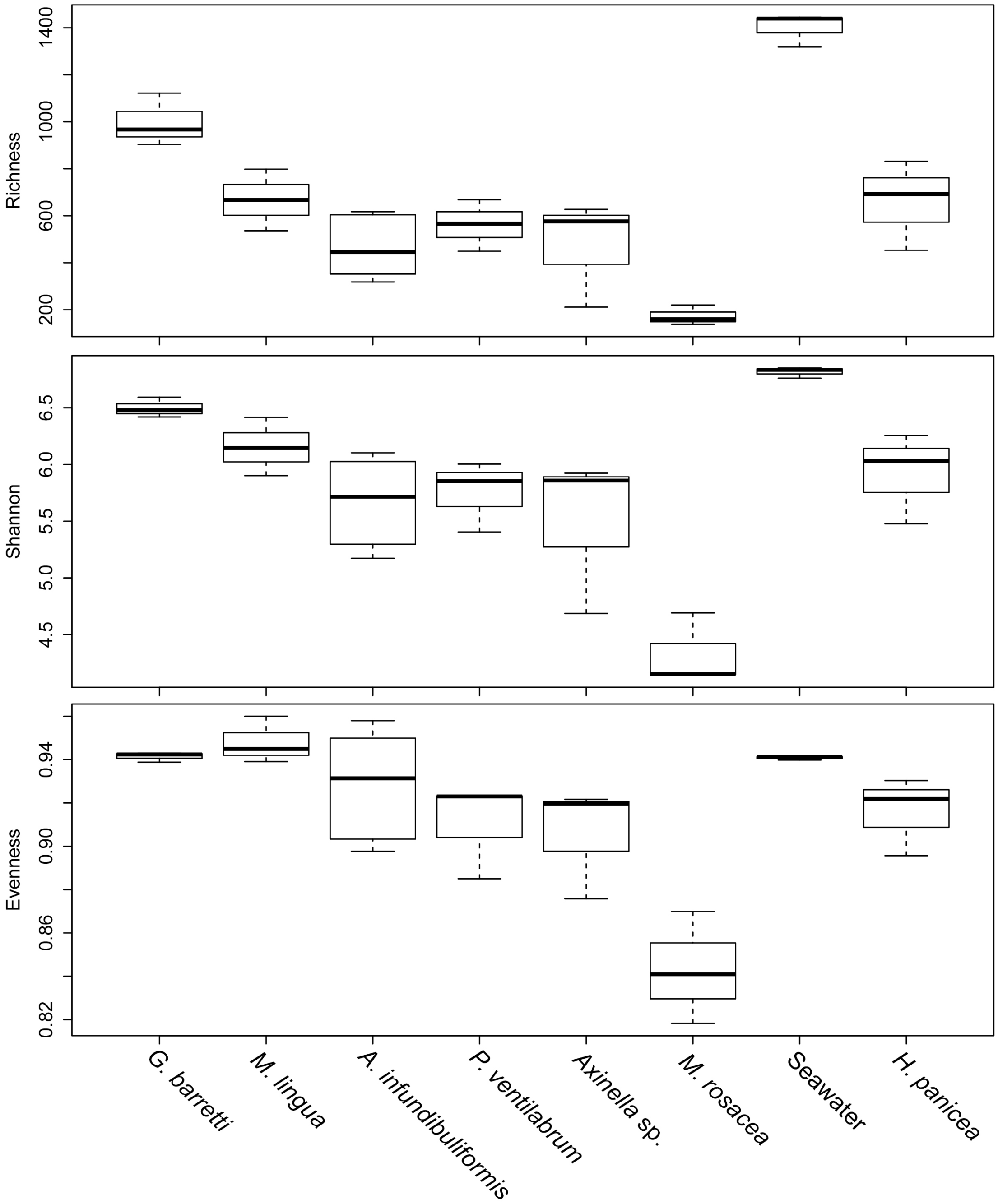


**Suppl. Figure 1** Number of average OTU richness and alpha diversity estimates for Shannon and Pielou's Evenness for all sponge and seawater samples. Top, middle, and bottom lines of the boxes represent the 25$^{th}$, 50$^{th}$ (median), and 75$^{th}$ percentiles, respectively. The end of the whiskers represent the 5$^{th}$ and 95$^{th}$ percentiles, respectively.

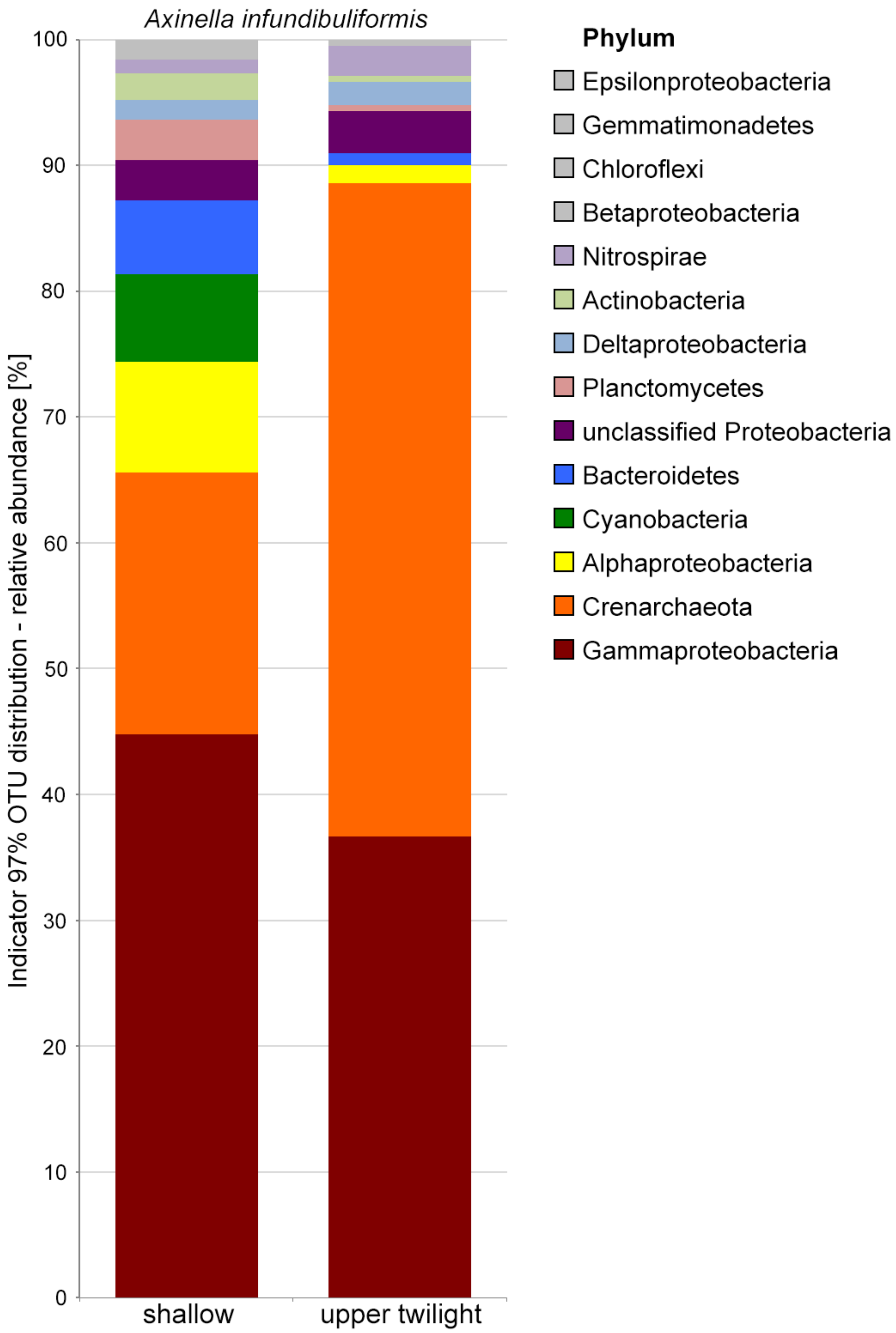


**Suppl. Figure 2** Relative abundance and diversity of significantly correlated indicator species collapsed to phylum level for the two existing *A. infundibuliformis* groups. For better clarity only phyla with an abundance $\geq$ 0.25% are colorized – a more detailed table with all individual significant OTUs and taxonomic classification down to species level can be found in the Supplementary Table 3.

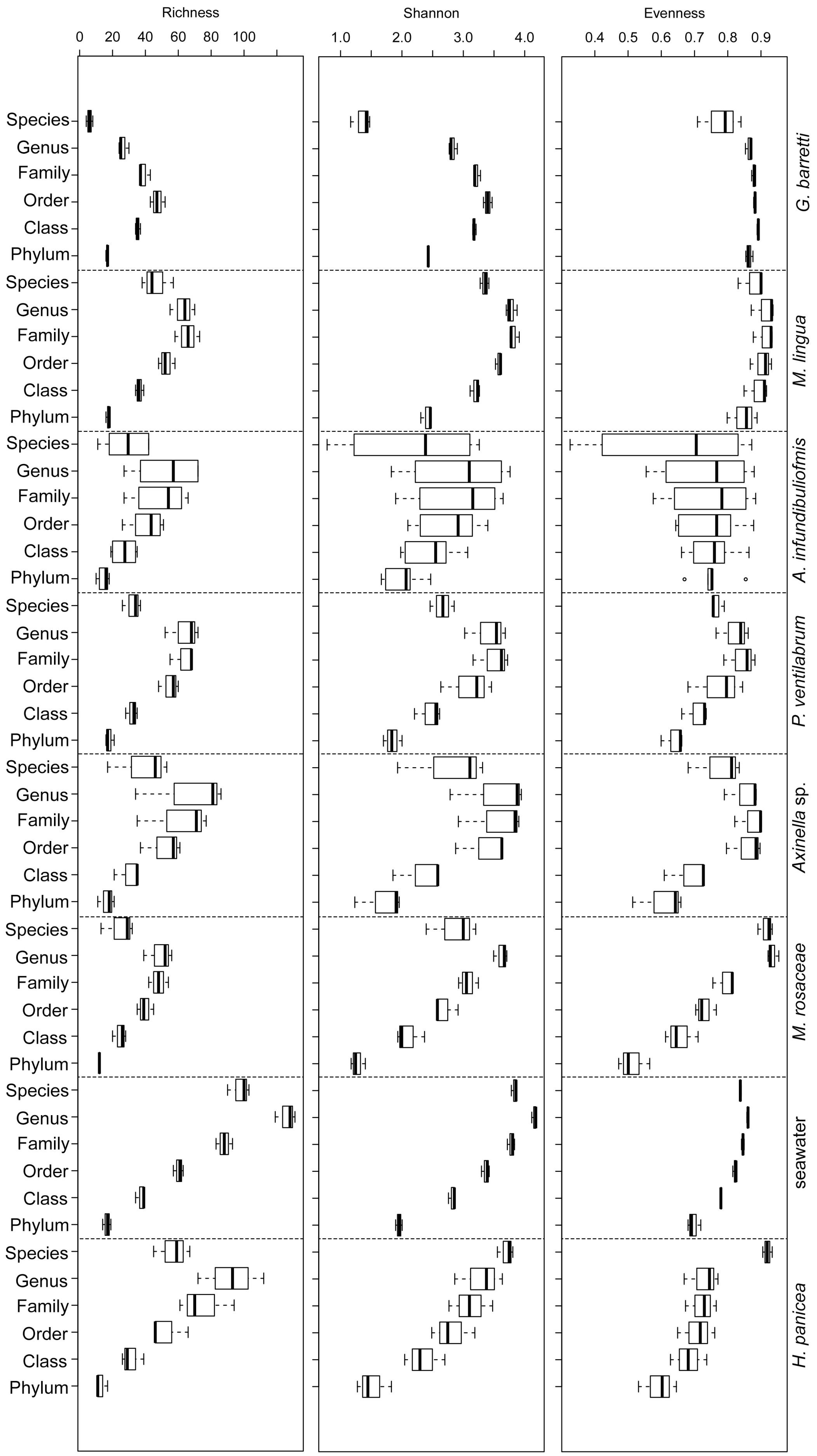


**Suppl. Figure 3**. Number of average richness and alpha diversity estimates for Shannon and Pielou's Evenness for all sponge and seawater samples across all high taxonomic ranks. Top, middle, and bottom lines of the boxes represent the 25th, 50th (median), and 75th percentiles, respectively. The end of the whiskers represent the 5th and 95th percentiles, respectively.

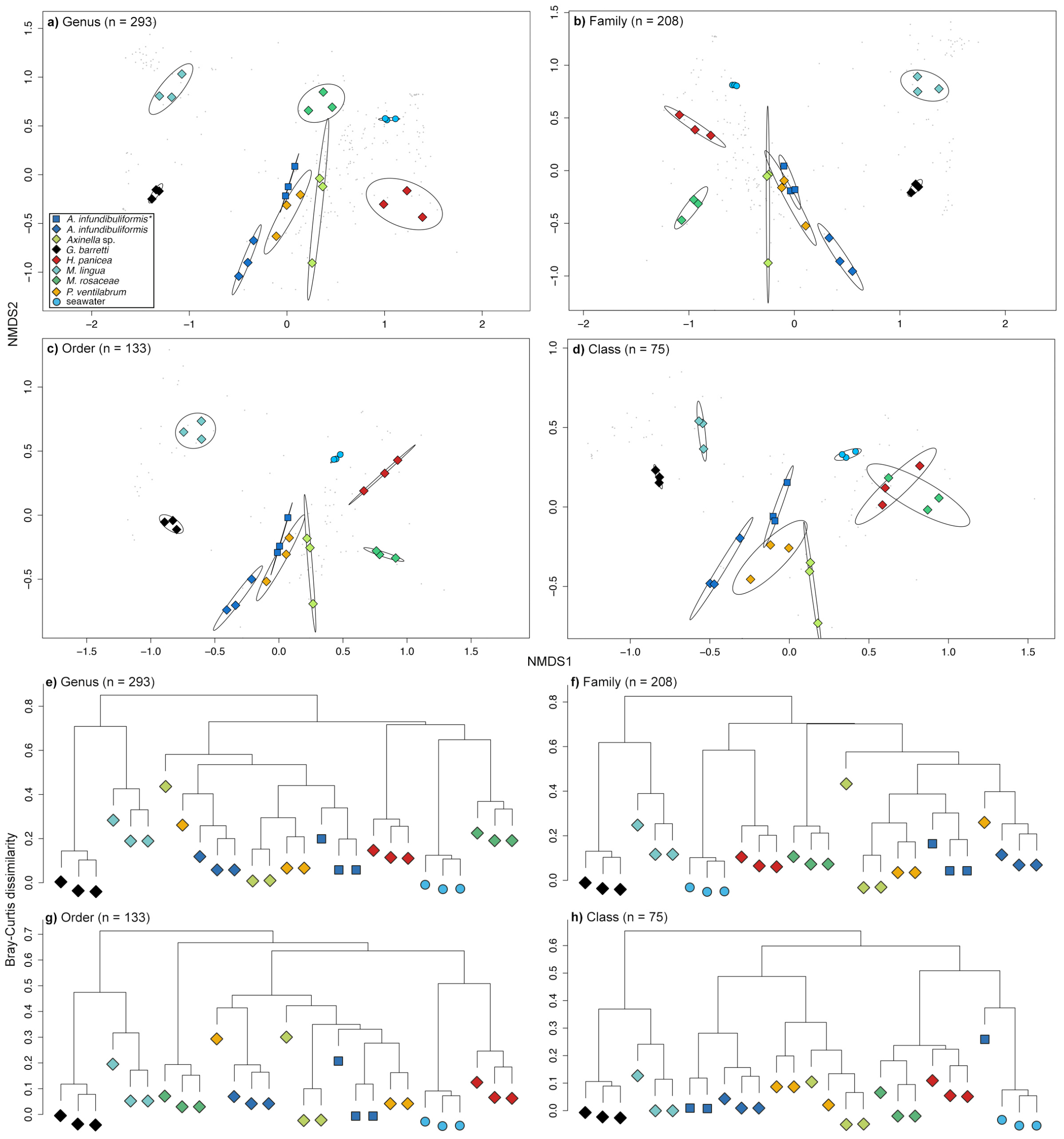


**Suppl. Figure 4** Additional nMDS and cluster plots for genus, family, order, and class levels. Details are the same as those provided for Figure 3.

**Suppl. Table 1** The OTU richness, Shannon diversity and Evenness relationships between *A. infundibuliformis* replicates from two different depths. *df* = degrees of freedom, *F values*, and *p* values.

| ***A. infundibuliformis* shallow vs. upper twilight** | ***Df*** | ***F* value** | ***p* value** |
|---|---|---|---|
| Richness (*S*) | 1, 4 | 75.33 | **0.00097** |
| Shannon (*H*) | 1, 4 | 105.2 | **0.00051** |
| Evenness (*J*) | 1, 4 | 24.14 | **0.00797** |

**Suppl. Table 2** Summary of the indicator species (i.e., OTUs) analysis performed by the function multipatt. Shown are 'Rest' and 'Stat' as values of the statistic for selecting the indicator OTUs. Only OTUs with significant $p$ values $\leq$ 0.05 are summarized. In addition for each OTU, Greengenes classification results are added from phylum to species level if available.

| Otus | *Phakellia ventilabrum* | Rest | stat | *p value* | Domain | Phylum | Class | Order | Family | Genus | Species |
|---|---|---|---|---|---|---|---|---|---|---|---|
| Otu000121 | 0.9979 | 1 | 0.999 | 0.001 | Bacteria | Proteobacteria | Gammaproteobacteria | unclassified | unclassified | unclassified | unclassified |
| Otu000129 | 0.6883 | 1 | 0.83 | 0.047 | Bacteria | Proteobacteria | unclassified | unclassified | unclassified | unclassified | unclassified |
| Otu000139 | 0.9433 | 1 | 0.971 | 0.001 | Bacteria | Proteobacteria | unclassified | unclassified | unclassified | unclassified | unclassified |
| Otu000342 | 0.9986 | 1 | 0.999 | 0.001 | Bacteria | Proteobacteria | Gammaproteobacteria | unclassified | unclassified | unclassified | unclassified |
| Otu000359 | 0.6661 | 1 | 0.816 | 0.01 | Bacteria | PAUC34f | unclassified | unclassified | unclassified | unclassified | unclassified |
| Otu000378 | 0.9939 | 1 | 0.997 | 0.001 | Bacteria | Proteobacteria | unclassified | unclassified | unclassified | unclassified | unclassified |
| Otu000729 | 0.7298 | 1 | 0.854 | 0.039 | Archaea | Crenarchaeota | Thaumarchaeota | Cenarchaeales | Cenarchaeaceae | Nitrosopumilus | unclassified |
| Otu001175 | 0.5978 | 1 | 0.773 | 0.028 | Bacteria | unclassified | unclassified | unclassified | unclassified | unclassified | unclassified |
| Otu001422 | 0.8082 | 1 | 0.899 | 0.015 | Archaea | Crenarchaeota | Thaumarchaeota | Cenarchaeales | Cenarchaeaceae | Nitrosopumilus | unclassified |
| Otu001859 | 1 | 1 | 1 | 0.001 | Bacteria | Proteobacteria | Gammaproteobacteria | unclassified | unclassified | unclassified | unclassified |
| Otu001934 | 1 | 1 | 1 | 0.001 | Bacteria | Proteobacteria | Alphaproteobacteria | unclassified | unclassified | unclassified | unclassified |
| Otu002228 | 1 | 1 | 1 | 0.001 | Bacteria | Proteobacteria | unclassified | unclassified | unclassified | unclassified | unclassified |
| Otu002275 | 0.923 | 1 | 0.961 | 0.002 | Bacteria | Proteobacteria | unclassified | unclassified | unclassified | unclassified | unclassified |
| Otu002284 | 0.8205 | 1 | 0.906 | 0.005 | Bacteria | Proteobacteria | Betaproteobacteria | Nitrosomonadales | Nitrosomonadaceae | unclassified | unclassified |
| Otu002516 | 0.9138 | 0.6667 | 0.781 | 0.026 | Bacteria | Proteobacteria | Gammaproteobacteria | Legionellales | Coxiellaceae | unclassified | unclassified |
| Otu002524 | 0.8325 | 1 | 0.912 | 0.015 | Archaea | Crenarchaeota | Thaumarchaeota | Cenarchaeales | Cenarchaeaceae | Nitrosopumilus | unclassified |
| Otu002903 | 1 | 1 | 1 | 0.001 | Bacteria | Proteobacteria | unclassified | unclassified | unclassified | unclassified | unclassified |
| Otu003144 | 0.9975 | 1 | 0.999 | 0.001 | Bacteria | Nitrospirae | Nitrospira | Nitrospirales | Nitrospiraceae | unclassified | unclassified |
| Otu003521 | 0.9444 | 0.6667 | 0.793 | 0.021 | Bacteria | Actinobacteria | Acidimicrobiia | Acidimicrobiales | ntu14 | unclassified | unclassified |
| Otu003537 | 1 | 1 | 1 | 0.001 | Bacteria | Proteobacteria | Gammaproteobacteria | unclassified | unclassified | unclassified | unclassified |
| Otu003584 | 0.7387 | 1 | 0.859 | 0.036 | Archaea | Crenarchaeota | Thaumarchaeota | Cenarchaeales | Cenarchaeaceae | Nitrosopumilus | unclassified |
| Otu004161 | 0.9808 | 1 | 0.99 | 0.001 | Bacteria | Proteobacteria | Gammaproteobacteria | unclassified | unclassified | unclassified | unclassified |
| Otu004353 | 0.9956 | 1 | 0.998 | 0.001 | Bacteria | Proteobacteria | unclassified | unclassified | unclassified | unclassified | unclassified |
| Otu004564 | 0.8302 | 1 | 0.911 | 0.012 | Bacteria | Proteobacteria | Deltaproteobacteria | Sva0853 | SAR324 | unclassified | unclassified |
| Otu004575 | 0.8601 | 1 | 0.927 | 0.002 | Bacteria | Proteobacteria | Gammaproteobacteria | Thiotrichales | Piscirickettsiaceae | unclassified | unclassified |
| Otu004594 | 1 | 1 | 1 | 0.001 | Bacteria | Proteobacteria | Gammaproteobacteria | unclassified | unclassified | unclassified | unclassified |
| Otu004636 | 0.8498 | 0.6667 | 0.753 | 0.02 | Bacteria | Proteobacteria | Gammaproteobacteria | unclassified | unclassified | unclassified | unclassified |
| Otu004850 | 0.8661 | 1 | 0.931 | 0.004 | Bacteria | Proteobacteria | Gammaproteobacteria | unclassified | unclassified | unclassified | unclassified |
| Otu004972 | 0.8581 | 1 | 0.926 | 0.005 | Archaea | Crenarchaeota | Thaumarchaeota | Cenarchaeales | Cenarchaeaceae | Nitrosopumilus | unclassified |
| Otu005164 | 0.8121 | 1 | 0.901 | 0.014 | Archaea | Crenarchaeota | Thaumarchaeota | Cenarchaeales | Cenarchaeaceae | Nitrosopumilus | unclassified |
| Otu005169 | 0.7779 | 1 | 0.882 | 0.017 | Archaea | Crenarchaeota | Thaumarchaeota | Cenarchaeales | Cenarchaeaceae | Nitrosopumilus | unclassified |
| Otu005262 | 1 | 0.6667 | 0.816 | 0.006 | Bacteria | unclassified | unclassified | unclassified | unclassified | unclassified | unclassified |
| Otu005425 | 0.7745 | 1 | 0.88 | 0.025 | Archaea | Crenarchaeota | Thaumarchaeota | Cenarchaeales | Cenarchaeaceae | Nitrosopumilus | unclassified |
| Otu005450 | 0.9284 | 0.6667 | 0.787 | 0.019 | Bacteria | Proteobacteria | Gammaproteobacteria | Oceanospirillales | Halomonadaceae | Candidatus_Portiera | unclassified |
| Otu005499 | 1 | 1 | 1 | 0.001 | Bacteria | Proteobacteria | Betaproteobacteria | unclassified | unclassified | unclassified | unclassified |
| Otu005626 | 0.8679 | 0.6667 | 0.761 | 0.031 | Bacteria | Proteobacteria | Gammaproteobacteria | unclassified | unclassified | unclassified | unclassified |
| Otu005649 | 1 | 1 | 1 | 0.001 | Bacteria | Proteobacteria | Gammaproteobacteria | unclassified | unclassified | unclassified | unclassified |
| Otu005877 | 0.738 | 1 | 0.859 | 0.034 | Bacteria | Proteobacteria | Gammaproteobacteria | unclassified | unclassified | unclassified | unclassified |
| Otu006002 | 0.7911 | 1 | 0.889 | 0.009 | Bacteria | Proteobacteria | Deltaproteobacteria | Desulfobacterales | Nitrospinaceae | Nitrospina | unclassified |
| Otu006017 | 0.8781 | 1 | 0.937 | 0.001 | Bacteria | Proteobacteria | Gammaproteobacteria | unclassified | unclassified | unclassified | unclassified |
| Otu006119 | 0.791 | 0.6667 | 0.726 | 0.036 | Bacteria | Proteobacteria | Gammaproteobacteria | unclassified | unclassified | unclassified | unclassified |
| Otu006137 | 0.9389 | 1 | 0.969 | 0.001 | Bacteria | Proteobacteria | unclassified | unclassified | unclassified | unclassified | unclassified |
| Otu006169 | 0.8581 | 0.6667 | 0.756 | 0.007 | Bacteria | Proteobacteria | Alphaproteobacteria | unclassified | unclassified | unclassified | unclassified |
| Otu006271 | 0.7491 | 1 | 0.865 | 0.034 | Bacteria | Proteobacteria | Betaproteobacteria | Methylophilales | Methylophilaceae | unclassified | unclassified |
| Otu006316 | 0.7374 | 1 | 0.859 | 0.038 | Archaea | Crenarchaeota | Thaumarchaeota | Cenarchaeales | Cenarchaeaceae | Nitrosopumilus | unclassified |
| Otu006886 | 0.7693 | 1 | 0.877 | 0.023 | Archaea | Crenarchaeota | Thaumarchaeota | Cenarchaeales | Cenarchaeaceae | Nitrosopumilus | unclassified |
| Otu006923 | 0.9376 | 1 | 0.968 | 0.001 | Archaea | Crenarchaeota | Thaumarchaeota | Cenarchaeales | Cenarchaeaceae | Nitrosopumilus | unclassified |
| Otu007001 | 0.7473 | 1 | 0.864 | 0.032 | Archaea | Crenarchaeota | Thaumarchaeota | Cenarchaeales | Cenarchaeaceae | Nitrosopumilus | unclassified |
| Otu007015 | 0.7351 | 1 | 0.857 | 0.006 | Bacteria | Proteobacteria | Betaproteobacteria | unclassified | unclassified | unclassified | unclassified |
| Otu007084 | 0.8537 | 1 | 0.924 | 0.007 | Bacteria | Proteobacteria | Betaproteobacteria | unclassified | unclassified | unclassified | unclassified |
| Otu007097 | 0.8169 | 1 | 0.904 | 0.009 | Archaea | Crenarchaeota | Thaumarchaeota | Cenarchaeales | Cenarchaeaceae | Nitrosopumilus | unclassified |
| Otu007299 | 0.8241 | 1 | 0.908 | 0.005 | Bacteria | Proteobacteria | unclassified | unclassified | unclassified | unclassified | unclassified |
| Otu007306 | 0.9152 | 1 | 0.957 | 0.002 | Bacteria | Proteobacteria | unclassified | unclassified | unclassified | unclassified | unclassified |
| Otu007812 | 0.8283 | 1 | 0.91 | 0.011 | Archaea | Crenarchaeota | Thaumarchaeota | Cenarchaeales | Cenarchaeaceae | Nitrosopumilus | unclassified |
| Otu007846 | 1 | 1 | 1 | 0.001 | Bacteria | unclassified | unclassified | unclassified | unclassified | unclassified | unclassified |
| Otu007911 | 0.7462 | 1 | 0.864 | 0.033 | Archaea | Crenarchaeota | Thaumarchaeota | Cenarchaeales | Cenarchaeaceae | Nitrosopumilus | unclassified |
| Otu008196 | 0.7909 | 1 | 0.889 | 0.017 | Archaea | Crenarchaeota | Thaumarchaeota | Cenarchaeales | Cenarchaeaceae | Nitrosopumilus | unclassified |
| Otu008208 | 0.7633 | 1 | 0.874 | 0.023 | Archaea | Crenarchaeota | Thaumarchaeota | Cenarchaeales | Cenarchaeaceae | Nitrosopumilus | unclassified |
| Otu008254 | 0.7539 | 1 | 0.868 | 0.02 | Bacteria | Proteobacteria | Alphaproteobacteria | unclassified | unclassified | unclassified | unclassified |
| Otu008651 | 1 | 1 | 1 | 0.001 | Bacteria | Proteobacteria | Gammaproteobacteria | unclassified | unclassified | unclassified | unclassified |
| Otu008760 | 0.9039 | 0.6667 | 0.776 | 0.008 | Bacteria | Planctomycetes | Planctomycetia | unclassified | unclassified | unclassified | unclassified |
| Otu008875 | 0.7196 | 1 | 0.848 | 0.02 | Bacteria | Chloroflexi | SAR202 | unclassified | unclassified | unclassified | unclassified |
| Otu008876 | 0.8109 | 1 | 0.9 | 0.019 | Archaea | Crenarchaeota | Thaumarchaeota | Cenarchaeales | Cenarchaeaceae | Nitrosopumilus | unclassified |
| Otu009043 | 0.734 | 1 | 0.857 | 0.035 | Archaea | Crenarchaeota | Thaumarchaeota | Cenarchaeales | Cenarchaeaceae | Nitrosopumilus | unclassified |
| Otu009079 | 0.8038 | 1 | 0.897 | 0.018 | Archaea | Crenarchaeota | Thaumarchaeota | Cenarchaeales | Cenarchaeaceae | Nitrosopumilus | unclassified |
| Otu009132 | 1 | 1 | 1 | 0.001 | Bacteria | Proteobacteria | Gammaproteobacteria | unclassified | unclassified | unclassified | unclassified |
| Otu009210 | 0.8329 | 1 | 0.913 | 0.012 | Archaea | Crenarchaeota | Thaumarchaeota | Cenarchaeales | Cenarchaeaceae | Nitrosopumilus | unclassified |
| Otu009308 | 0.7856 | 1 | 0.886 | 0.022 | Archaea | Crenarchaeota | Thaumarchaeota | Cenarchaeales | Cenarchaeaceae | Nitrosopumilus | unclassified |
| Otu009491 | 1 | 1 | 1 | 0.001 | Bacteria | Proteobacteria | Gammaproteobacteria | unclassified | unclassified | unclassified | unclassified |
| Otu009549 | 0.8887 | 1 | 0.943 | 0.001 | Bacteria | Planctomycetes | Phycisphaerae | Phycisphaerales | unclassified | unclassified | unclassified |
| Otu009610 | 0.9336 | 1 | 0.966 | 0.001 | Bacteria | Proteobacteria | unclassified | unclassified | unclassified | unclassified | unclassified |
| Otu009768 | 0.7826 | 1 | 0.885 | 0.023 | Archaea | Crenarchaeota | Thaumarchaeota | Cenarchaeales | Cenarchaeaceae | Nitrosopumilus | unclassified |
| Otu009847 | 0.8056 | 1 | 0.898 | 0.018 | Archaea | Crenarchaeota | Thaumarchaeota | Cenarchaeales | Cenarchaeaceae | Nitrosopumilus | unclassified |
| Otu009849 | 0.8139 | 1 | 0.902 | 0.011 | Archaea | Crenarchaeota | Thaumarchaeota | Cenarchaeales | Cenarchaeaceae | Nitrosopumilus | unclassified |
| Otu009854 | 0.7761 | 1 | 0.881 | 0.021 | Archaea | Crenarchaeota | Thaumarchaeota | Cenarchaeales | Cenarchaeaceae | Nitrosopumilus | unclassified |
| Otu010012 | 0.7647 | 1 | 0.874 | 0.016 | Bacteria | unclassified | unclassified | unclassified | unclassified | unclassified | unclassified |
| Otu010017 | 0.8883 | 1 | 0.943 | 0.006 | Bacteria | Proteobacteria | Alphaproteobacteria | Rhodobacterales | Rhodobacteraceae | Octadecabacter | unclassified |
| Otu010111 | 0.7235 | 1 | 0.851 | 0.024 | Archaea | Crenarchaeota | Thaumarchaeota | Cenarchaeales | Cenarchaeaceae | Nitrosopumilus | unclassified |
| Otu010153 | 0.8678 | 0.6667 | 0.761 | 0.006 | Bacteria | Bacteroidetes | unclassified | unclassified | unclassified | unclassified | unclassified |
| Otu010163 | 0.7254 | 1 | 0.852 | 0.043 | Bacteria | Proteobacteria | Gammaproteobacteria | Thiotrichales | Piscirickettsiaceae | unclassified | unclassified |
| Otu010171 | 0.9152 | 0.6667 | 0.781 | 0.031 | Bacteria | Bacteroidetes | Flavobacteriia | Flavobacteriales | Flavobacteriaceae | unclassified | unclassified |
| Otu010175 | 0.8147 | 1 | 0.903 | 0.004 | Bacteria | Bacteroidetes | Flavobacteriia | Flavobacteriales | Cryomorphaceae | Fluviicola | unclassified |
| Otu010210 | 0.9642 | 0.6667 | 0.802 | 0.006 | Archaea | Crenarchaeota | Thaumarchaeota | Cenarchaeales | Cenarchaeaceae | Nitrosopumilus | pIVWA5 |
| Otu010311 | 0.7191 | 1 | 0.848 | 0.042 | Archaea | Crenarchaeota | Thaumarchaeota | Cenarchaeales | Cenarchaeaceae | Nitrosopumilus | unclassified |
| Otu010379 | 0.7401 | 1 | 0.86 | 0.035 | Archaea | Crenarchaeota | Thaumarchaeota | Cenarchaeales | Cenarchaeaceae | Nitrosopumilus | unclassified |
| Otu010426 | 0.748 | 1 | 0.865 | 0.027 | Archaea | Crenarchaeota | Thaumarchaeota | Cenarchaeales | Cenarchaeaceae | Nitrosopumilus | unclassified |
| Otu010744 | 1 | 1 | 1 | 0.001 | Bacteria | Proteobacteria | unclassified | unclassified | unclassified | unclassified | unclassified |
| Otu010996 | 1 | 1 | 1 | 0.001 | Bacteria | Proteobacteria | unclassified | unclassified | unclassified | unclassified | unclassified |
| Otu011171 | 1 | 1 | 1 | 0.001 | Bacteria | Proteobacteria | Gammaproteobacteria | unclassified | unclassified | unclassified | unclassified |
| Otu011429 | 0.8046 | 1 | 0.897 | 0.016 | Archaea | Crenarchaeota | Thaumarchaeota | Cenarchaeales | Cenarchaeaceae | Nitrosopumilus | unclassified |
| Otu012309 | 0.7353 | 1 | 0.857 | 0.039 | Archaea | Crenarchaeota | Thaumarchaeota | Cenarchaeales | Cenarchaeaceae | Nitrosopumilus | unclassified |
| Otu012324 | 0.8147 | 1 | 0.903 | 0.013 | Archaea | Crenarchaeota | Thaumarchaeota | Cenarchaeales | Cenarchaeaceae | Nitrosopumilus | unclassified |
| Otu012362 | 0.7584 | 1 | 0.871 | 0.023 | Archaea | Crenarchaeota | Thaumarchaeota | Cenarchaeales | Cenarchaeaceae | Nitrosopumilus | unclassified |
| Otu012592 | 0.8003 | 1 | 0.895 | 0.017 | Bacteria | Gemmatimonadetes | Gemm-2 | unclassified | unclassified | unclassified | unclassified |
| Otu012626 | 0.9186 | 1 | 0.958 | 0.001 | Bacteria | Proteobacteria | unclassified | unclassified | unclassified | unclassified | unclassified |
| Otu012647 | 0.8294 | 1 | 0.911 | 0.005 | Bacteria | Planctomycetes | Planctomycetia | Planctomycetales | Planctomycetaceae | Planctomyces | unclassified |

| | | | | | | | | | | | |
|---|---|---|---|---|---|---|---|---|---|---|---|
| Otu012657 | 0.8935 | 1 | 0.945 | 0.002 | Bacteria | Proteobacteria | Deltaproteobacteria | Desulfobacterales | Nitrospinaceae | Nitrospina | unclassified |
| Otu012751 | 0.8347 | 1 | 0.914 | 0.011 | Archaea | Crenarchaeota | Thaumarchaeota | Cenarchaeales | Cenarchaeaceae | Nitrosopumilus | unclassified |
| Otu012962 | 0.8693 | 0.6667 | 0.761 | 0.023 | Bacteria | unclassified | unclassified | unclassified | unclassified | unclassified | unclassified |
| Otu013185 | 0.7949 | 1 | 0.892 | 0.01 | Bacteria | Planctomycetes | Phycisphaerae | Phycisphaerales | unclassified | unclassified | unclassified |
| Otu013336 | 0.8423 | 0.6667 | 0.749 | 0.028 | Bacteria | Planctomycetes | OM190 | CL500-15 | unclassified | unclassified | unclassified |
| Otu013345 | 1 | 1 | 1 | 0.001 | Bacteria | Proteobacteria | Gammaproteobacteria | unclassified | unclassified | unclassified | unclassified |
| Otu013594 | 1 | 1 | 1 | 0.001 | Bacteria | Proteobacteria | Gammaproteobacteria | unclassified | unclassified | unclassified | unclassified |
| Otu013658 | 0.7518 | 1 | 0.867 | 0.03 | Archaea | Crenarchaeota | Thaumarchaeota | Cenarchaeales | Cenarchaeaceae | Nitrosopumilus | unclassified |
| Otu013849 | 0.7185 | 1 | 0.848 | 0.036 | Archaea | Crenarchaeota | Thaumarchaeota | Cenarchaeales | Cenarchaeaceae | Nitrosopumilus | unclassified |
| Otu014109 | 0.8615 | 1 | 0.928 | 0.002 | Bacteria | Proteobacteria | unclassified | unclassified | unclassified | unclassified | unclassified |
| Otu014309 | 0.7712 | 1 | 0.878 | 0.022 | Bacteria | Proteobacteria | Gammaproteobacteria | Oceanospirillales | unclassified | unclassified | unclassified |
| Otu014416 | 1 | 1 | 1 | 0.001 | Bacteria | Proteobacteria | Gammaproteobacteria | unclassified | unclassified | unclassified | unclassified |
| Otu014467 | 1 | 1 | 1 | 0.001 | Bacteria | unclassified | unclassified | unclassified | unclassified | unclassified | unclassified |
| Otu014468 | 0.6503 | 1 | 0.806 | 0.036 | Bacteria | Proteobacteria | Gammaproteobacteria | unclassified | unclassified | unclassified | unclassified |
| Otu014589 | 0.7741 | 1 | 0.88 | 0.027 | Archaea | Crenarchaeota | Thaumarchaeota | Cenarchaeales | Cenarchaeaceae | Nitrosopumilus | unclassified |
| Otu014636 | 1 | 0.6667 | 0.816 | 0.007 | Archaea | Crenarchaeota | Thaumarchaeota | Cenarchaeales | Cenarchaeaceae | Nitrosopumilus | unclassified |
| Otu014638 | 0.6856 | 1 | 0.828 | 0.047 | Archaea | Crenarchaeota | Thaumarchaeota | Cenarchaeales | Cenarchaeaceae | Nitrosopumilus | unclassified |
| Otu014781 | 0.9362 | 1 | 0.968 | 0.001 | Bacteria | Bacteroidetes | Flavobacteriia | Flavobacteriales | Flavobacteriaceae | unclassified | unclassified |
| Otu014833 | 0.8011 | 1 | 0.895 | 0.01 | Archaea | Crenarchaeota | Thaumarchaeota | Cenarchaeales | Cenarchaeaceae | Nitrosopumilus | unclassified |
| Otu014951 | 0.7209 | 1 | 0.849 | 0.046 | Archaea | Crenarchaeota | Thaumarchaeota | Cenarchaeales | Cenarchaeaceae | Nitrosopumilus | unclassified |
| Otu014974 | 0.8517 | 1 | 0.923 | 0.01 | Archaea | Crenarchaeota | Thaumarchaeota | Cenarchaeales | Cenarchaeaceae | Nitrosopumilus | unclassified |
| Otu015022 | 0.7159 | 1 | 0.846 | 0.032 | Archaea | Crenarchaeota | Thaumarchaeota | Cenarchaeales | Cenarchaeaceae | Nitrosopumilus | unclassified |
| Otu015057 | 0.7864 | 1 | 0.887 | 0.025 | Archaea | Crenarchaeota | Thaumarchaeota | Cenarchaeales | Cenarchaeaceae | Nitrosopumilus | unclassified |
| Otu015125 | 0.9602 | 1 | 0.98 | 0.002 | Archaea | Crenarchaeota | Thaumarchaeota | Cenarchaeales | Cenarchaeaceae | Nitrosopumilus | unclassified |
| Otu015156 | 0.5876 | 1 | 0.767 | 0.033 | Archaea | Crenarchaeota | Thaumarchaeota | Cenarchaeales | Cenarchaeaceae | Nitrosopumilus | unclassified |
| Otu015221 | 0.8141 | 1 | 0.902 | 0.009 | Archaea | Crenarchaeota | Thaumarchaeota | Cenarchaeales | Cenarchaeaceae | Nitrosopumilus | unclassified |
| Otu015239 | 0.8213 | 1 | 0.906 | 0.008 | Archaea | Crenarchaeota | Thaumarchaeota | Cenarchaeales | Cenarchaeaceae | Nitrosopumilus | unclassified |
| Otu015370 | 0.7835 | 1 | 0.885 | 0.015 | Archaea | Crenarchaeota | Thaumarchaeota | Cenarchaeales | Cenarchaeaceae | Nitrosopumilus | unclassified |
| Otu015394 | 0.8792 | 0.6667 | 0.766 | 0.032 | Archaea | Crenarchaeota | Thaumarchaeota | Cenarchaeales | Cenarchaeaceae | Nitrosopumilus | unclassified |
| Otu015417 | 1 | 0.6667 | 0.816 | 0.006 | Bacteria | Proteobacteria | Gammaproteobacteria | Alteromonadales | HTCC2188 | HTCC | unclassified |
| Otu015430 | 1 | 1 | 1 | 0.001 | Bacteria | Proteobacteria | unclassified | unclassified | unclassified | unclassified | unclassified |
| Otu015492 | 0.7302 | 1 | 0.854 | 0.014 | Bacteria | Proteobacteria | unclassified | unclassified | unclassified | unclassified | unclassified |
| Otu015596 | 0.8528 | 1 | 0.923 | 0.008 | Bacteria | Proteobacteria | Deltaproteobacteria | NB1-j | JTB38 | unclassified | unclassified |
| Otu015681 | 0.7626 | 1 | 0.873 | 0.022 | Archaea | Crenarchaeota | Thaumarchaeota | Cenarchaeales | Cenarchaeaceae | Nitrosopumilus | unclassified |
| Otu015690 | 0.7935 | 1 | 0.891 | 0.019 | Archaea | Crenarchaeota | Thaumarchaeota | Cenarchaeales | Cenarchaeaceae | Nitrosopumilus | unclassified |
| Otu015890 | 0.813 | 1 | 0.902 | 0.007 | Archaea | Crenarchaeota | Thaumarchaeota | Cenarchaeales | Cenarchaeaceae | Nitrosopumilus | unclassified |
| Otu016022 | 0.8323 | 1 | 0.912 | 0.006 | Bacteria | Proteobacteria | unclassified | unclassified | unclassified | unclassified | unclassified |
| Otu016057 | 0.8547 | 0.6667 | 0.755 | 0.029 | Bacteria | Bacteroidetes | Cytophagia | Cytophagales | Flammeovirgaceae | unclassified | unclassified |
| Otu016238 | 0.7551 | 1 | 0.869 | 0.035 | Archaea | Crenarchaeota | Thaumarchaeota | Cenarchaeales | Cenarchaeaceae | Nitrosopumilus | unclassified |
| Otu016249 | 0.7294 | 1 | 0.854 | 0.035 | Archaea | Crenarchaeota | Thaumarchaeota | Cenarchaeales | Cenarchaeaceae | Nitrosopumilus | unclassified |
| Otu016318 | 0.9135 | 1 | 0.956 | 0.001 | Bacteria | Proteobacteria | Betaproteobacteria | Nitrosomonadales | Nitrosomonadaceae | unclassified | unclassified |
| Otu016459 | 0.9069 | 1 | 0.952 | 0.001 | Bacteria | Proteobacteria | Alphaproteobacteria | unclassified | unclassified | unclassified | unclassified |
| Otu016531 | 0.9557 | 0.6667 | 0.798 | 0.006 | Bacteria | Planctomycetes | Planctomycetia | Planctomycetales | Planctomycetaceae | Planctomyces | unclassified |
| Otu016634 | 0.7368 | 1 | 0.858 | 0.033 | Bacteria | Proteobacteria | Gammaproteobacteria | unclassified | unclassified | unclassified | unclassified |
| Otu017011 | 1 | 1 | 1 | 0.001 | Bacteria | Proteobacteria | Gammaproteobacteria | unclassified | unclassified | unclassified | unclassified |
| Otu017262 | 0.7207 | 1 | 0.849 | 0.034 | Archaea | Crenarchaeota | Thaumarchaeota | Cenarchaeales | Cenarchaeaceae | Nitrosopumilus | unclassified |
| Otu017721 | 0.8885 | 0.6667 | 0.77 | 0.016 | Archaea | Crenarchaeota | Thaumarchaeota | Cenarchaeales | Cenarchaeaceae | Nitrosopumilus | unclassified |
| Otu017843 | 1 | 0.6667 | 0.816 | 0.007 | Bacteria | Proteobacteria | Gammaproteobacteria | Alteromonadales | unclassified | unclassified | unclassified |
| Otu017894 | 1 | 1 | 1 | 0.001 | Bacteria | Proteobacteria | unclassified | unclassified | unclassified | unclassified | unclassified |
| Otu017938 | 0.9101 | 1 | 0.954 | 0.001 | Bacteria | Proteobacteria | Gammaproteobacteria | unclassified | unclassified | unclassified | unclassified |
| Otu018271 | 0.8421 | 1 | 0.918 | 0.004 | Bacteria | Proteobacteria | Gammaproteobacteria | unclassified | unclassified | unclassified | unclassified |
| Otu018285 | 0.8333 | 1 | 0.913 | 0.013 | Archaea | Crenarchaeota | Thaumarchaeota | Cenarchaeales | Cenarchaeaceae | Nitrosopumilus | unclassified |
| Otu018361 | 0.8154 | 1 | 0.903 | 0.007 | Archaea | Crenarchaeota | Thaumarchaeota | Cenarchaeales | Cenarchaeaceae | Nitrosopumilus | unclassified |
| Otu018469 | 0.8126 | 1 | 0.901 | 0.011 | Bacteria | unclassified | unclassified | unclassified | unclassified | unclassified | unclassified |
| Otu018601 | 0.839 | 0.6667 | 0.748 | 0.035 | Bacteria | Proteobacteria | Gammaproteobacteria | unclassified | unclassified | unclassified | unclassified |
| Otu018603 | 0.8477 | 1 | 0.921 | 0.006 | Bacteria | Proteobacteria | unclassified | unclassified | unclassified | unclassified | unclassified |
| Otu018738 | 0.8404 | 1 | 0.917 | 0.009 | Archaea | Crenarchaeota | Thaumarchaeota | Cenarchaeales | Cenarchaeaceae | Nitrosopumilus | unclassified |
| Otu018759 | 0.8929 | 1 | 0.945 | 0.003 | Bacteria | Proteobacteria | unclassified | unclassified | unclassified | unclassified | unclassified |
| Otu018909 | 0.7784 | 1 | 0.882 | 0.019 | Archaea | Crenarchaeota | Thaumarchaeota | Cenarchaeales | Cenarchaeaceae | Nitrosopumilus | unclassified |
| Otu018973 | 0.6752 | 1 | 0.822 | 0.032 | Archaea | Crenarchaeota | Thaumarchaeota | Cenarchaeales | Cenarchaeaceae | Nitrosopumilus | unclassified |
| Otu019001 | 0.7635 | 1 | 0.874 | 0.026 | Archaea | Crenarchaeota | Thaumarchaeota | Cenarchaeales | Cenarchaeaceae | Nitrosopumilus | unclassified |
| Otu019011 | 0.8172 | 1 | 0.904 | 0.018 | Archaea | Crenarchaeota | Thaumarchaeota | Cenarchaeales | Cenarchaeaceae | Nitrosopumilus | unclassified |
| Otu019019 | 0.7781 | 1 | 0.882 | 0.024 | Archaea | Crenarchaeota | Thaumarchaeota | Cenarchaeales | Cenarchaeaceae | Nitrosopumilus | unclassified |
| Otu019072 | 0.6834 | 1 | 0.827 | 0.04 | Archaea | Crenarchaeota | Thaumarchaeota | Cenarchaeales | Cenarchaeaceae | Nitrosopumilus | unclassified |
| Otu019079 | 0.8015 | 1 | 0.895 | 0.014 | Archaea | Crenarchaeota | Thaumarchaeota | Cenarchaeales | Cenarchaeaceae | Nitrosopumilus | unclassified |
| Otu019233 | 0.756 | 1 | 0.869 | 0.031 | Archaea | Crenarchaeota | Thaumarchaeota | Cenarchaeales | Cenarchaeaceae | Nitrosopumilus | unclassified |
| Otu019258 | 0.8038 | 1 | 0.897 | 0.012 | Archaea | Crenarchaeota | Thaumarchaeota | Cenarchaeales | Cenarchaeaceae | Nitrosopumilus | unclassified |
| Otu019361 | 0.8574 | 1 | 0.926 | 0.004 | Archaea | Crenarchaeota | Thaumarchaeota | Cenarchaeales | Cenarchaeaceae | Nitrosopumilus | unclassified |
| Otu019363 | 0.9327 | 1 | 0.966 | 0.001 | Bacteria | Planctomycetes | Pla3 | unclassified | unclassified | unclassified | unclassified |
| Otu019487 | 0.7406 | 1 | 0.861 | 0.034 | Archaea | Crenarchaeota | Thaumarchaeota | Cenarchaeales | Cenarchaeaceae | Nitrosopumilus | unclassified |
| Otu019508 | 0.9027 | 0.6667 | 0.776 | 0.016 | Archaea | Crenarchaeota | Thaumarchaeota | Cenarchaeales | Cenarchaeaceae | Nitrosopumilus | unclassified |
| Otu019835 | 0.9368 | 1 | 0.968 | 0.001 | Bacteria | Proteobacteria | unclassified | unclassified | unclassified | unclassified | unclassified |
| Otu020055 | 0.8091 | 0.6667 | 0.734 | 0.028 | Bacteria | Proteobacteria | Alphaproteobacteria | Rhodobacterales | Rhodobacteraceae | unclassified | unclassified |
| Otu020560 | 1 | 1 | 1 | 0.001 | Bacteria | Proteobacteria | unclassified | unclassified | unclassified | unclassified | unclassified |
| Otu020742 | 0.8284 | 1 | 0.91 | 0.006 | Bacteria | Proteobacteria | unclassified | unclassified | unclassified | unclassified | unclassified |
| Otu020753 | 0.6505 | 1 | 0.807 | 0.006 | Bacteria | Proteobacteria | unclassified | unclassified | unclassified | unclassified | unclassified |
| Otu020993 | 1 | 1 | 1 | 0.001 | Bacteria | Proteobacteria | unclassified | unclassified | unclassified | unclassified | unclassified |
| Otu021005 | 0.8995 | 1 | 0.948 | 0.003 | Bacteria | Proteobacteria | unclassified | unclassified | unclassified | unclassified | unclassified |
| Otu021237 | 0.7907 | 1 | 0.889 | 0.018 | Archaea | Crenarchaeota | Thaumarchaeota | Cenarchaeales | Cenarchaeaceae | Nitrosopumilus | unclassified |
| Otu021287 | 0.7286 | 1 | 0.854 | 0.032 | Archaea | Crenarchaeota | Thaumarchaeota | Cenarchaeales | Cenarchaeaceae | Nitrosopumilus | unclassified |
| Otu021359 | 0.8241 | 1 | 0.908 | 0.013 | Archaea | Crenarchaeota | Thaumarchaeota | Cenarchaeales | Cenarchaeaceae | Nitrosopumilus | unclassified |
| Otu021497 | 0.8432 | 0.6667 | 0.75 | 0.015 | Archaea | Crenarchaeota | Thaumarchaeota | Cenarchaeales | Cenarchaeaceae | Nitrosopumilus | unclassified |
| Otu021512 | 1 | 1 | 1 | 0.001 | Bacteria | Proteobacteria | unclassified | unclassified | unclassified | unclassified | unclassified |
| Otu021575 | 0.9458 | 0.6667 | 0.794 | 0.006 | Bacteria | Proteobacteria | Alphaproteobacteria | Rhodospirillales | Rhodospirillaceae | unclassified | unclassified |
| Otu021583 | 0.9513 | 0.6667 | 0.796 | 0.009 | Bacteria | Bacteroidetes | unclassified | unclassified | unclassified | unclassified | unclassified |
| Otu021604 | 0.8765 | 1 | 0.936 | 0.002 | Bacteria | SAR406 | AB16 | Arctic96B-7 | A714017 | SargSea-WGS | unclassified |
| Otu021772 | 0.7898 | 1 | 0.889 | 0.021 | Archaea | Crenarchaeota | Thaumarchaeota | Cenarchaeales | Cenarchaeaceae | Nitrosopumilus | unclassified |
| Otu021858 | 0.7482 | 1 | 0.865 | 0.041 | Archaea | Crenarchaeota | Thaumarchaeota | Cenarchaeales | Cenarchaeaceae | Nitrosopumilus | unclassified |
| Otu022351 | 0.8877 | 1 | 0.942 | 0.002 | Bacteria | Verrucomicrobia | Pedosphaerae | Arctic97B-4 | unclassified | unclassified | unclassified |
| Otu022881 | 0.8011 | 1 | 0.895 | 0.013 | Archaea | Crenarchaeota | Thaumarchaeota | Cenarchaeales | Cenarchaeaceae | Nitrosopumilus | unclassified |
| Otu022894 | 0.7486 | 1 | 0.865 | 0.037 | Archaea | Crenarchaeota | Thaumarchaeota | Cenarchaeales | Cenarchaeaceae | Nitrosopumilus | unclassified |
| Otu023541 | 0.7786 | 1 | 0.882 | 0.03 | Archaea | Crenarchaeota | Thaumarchaeota | Cenarchaeales | Cenarchaeaceae | Nitrosopumilus | unclassified |
| Otu023649 | 0.8209 | 1 | 0.906 | 0.008 | Archaea | Crenarchaeota | Thaumarchaeota | Cenarchaeales | Cenarchaeaceae | Nitrosopumilus | unclassified |
| Otu023917 | 1 | 1 | 1 | 0.001 | Bacteria | Proteobacteria | unclassified | unclassified | unclassified | unclassified | unclassified |
| Otu024304 | 0.8464 | 0.6667 | 0.751 | 0.043 | Bacteria | Proteobacteria | Alphaproteobacteria | unclassified | unclassified | unclassified | unclassified |
| Otu024592 | 0.7584 | 1 | 0.871 | 0.026 | Bacteria | Proteobacteria | Alphaproteobacteria | unclassified | unclassified | unclassified | unclassified |
| Otu024771 | 0.7473 | 1 | 0.864 | 0.038 | Archaea | Crenarchaeota | Thaumarchaeota | Cenarchaeales | Cenarchaeaceae | Nitrosopumilus | unclassified |
| Otu024937 | 0.7615 | 1 | 0.873 | 0.03 | Archaea | Crenarchaeota | Thaumarchaeota | Cenarchaeales | Cenarchaeaceae | Nitrosopumilus | unclassified |
| Otu024955 | 0.8232 | 1 | 0.907 | 0.009 | Bacteria | Proteobacteria | Betaproteobacteria | Nitrosomonadales | Nitrosomonadaceae | unclassified | unclassified |
| Otu025017 | 0.8392 | 1 | 0.916 | 0.003 | Bacteria | Proteobacteria | Betaproteobacteria | Methylophilales | Methylophilaceae | unclassified | unclassified |

| | | | | | | | | | | | |
|---|---|---|---|---|---|---|---|---|---|---|---|
| Otu025018 | 0.8919 | 0.6667 | 0.771 | 0.023 | Bacteria | Proteobacteria | Betaproteobacteria | Methylophilales | Methylophilaceae | unclassified | unclassified |
| Otu025033 | 0.8084 | 1 | 0.899 | 0.014 | Bacteria | Verrucomicrobia | Pedosphaerae | unclassified | unclassified | unclassified | unclassified |
| Otu025067 | 0.7379 | 1 | 0.859 | 0.037 | Archaea | Crenarchaeota | Thaumarchaeota | Cenarchaeales | Cenarchaeaceae | Nitrosopumilus | unclassified |
| Otu025148 | 0.8263 | 1 | 0.909 | 0.015 | Archaea | Crenarchaeota | Thaumarchaeota | Cenarchaeales | Cenarchaeaceae | Nitrosopumilus | unclassified |
| Otu025204 | 0.6676 | 1 | 0.817 | 0.02 | Archaea | Crenarchaeota | Thaumarchaeota | Cenarchaeales | Cenarchaeaceae | Nitrosopumilus | unclassified |
| Otu025402 | 0.7854 | 1 | 0.886 | 0.025 | Archaea | Crenarchaeota | Thaumarchaeota | Cenarchaeales | Cenarchaeaceae | Nitrosopumilus | unclassified |
| Otu025503 | 0.9694 | 0.6667 | 0.804 | 0.006 | Bacteria | Proteobacteria | Gammaproteobacteria | unclassified | unclassified | unclassified | unclassified |
| Otu025657 | 1 | 1 | 1 | 0.001 | Bacteria | Proteobacteria | unclassified | unclassified | unclassified | unclassified | unclassified |
| Otu025663 | 1 | 1 | 1 | 0.001 | Bacteria | Proteobacteria | unclassified | unclassified | unclassified | unclassified | unclassified |
| Otu025926 | 0.7704 | 1 | 0.878 | 0.028 | Archaea | Crenarchaeota | Thaumarchaeota | Cenarchaeales | Cenarchaeaceae | Nitrosopumilus | unclassified |
| Otu026415 | 0.6983 | 1 | 0.836 | 0.029 | Archaea | Crenarchaeota | Thaumarchaeota | Cenarchaeales | Cenarchaeaceae | Nitrosopumilus | unclassified |
| Otu026519 | 0.812 | 1 | 0.901 | 0.018 | Archaea | Crenarchaeota | Thaumarchaeota | Cenarchaeales | Cenarchaeaceae | Nitrosopumilus | unclassified |
| Otu026752 | 0.9567 | 1 | 0.978 | 0.001 | Archaea | Crenarchaeota | Thaumarchaeota | Cenarchaeales | Cenarchaeaceae | Nitrosopumilus | unclassified |
| Otu026873 | 1 | 1 | 1 | 0.001 | Bacteria | SBR1093 | EC214 | unclassified | unclassified | unclassified | unclassified |
| Otu026982 | 0.7886 | 1 | 0.888 | 0.006 | Bacteria | Proteobacteria | unclassified | unclassified | unclassified | unclassified | unclassified |
| Otu026986 | 0.764 | 1 | 0.874 | 0.006 | Bacteria | Proteobacteria | unclassified | unclassified | unclassified | unclassified | unclassified |
| Otu027228 | 0.7296 | 1 | 0.854 | 0.006 | Bacteria | Proteobacteria | unclassified | unclassified | unclassified | unclassified | unclassified |
| Otu027251 | 1 | 1 | 1 | 0.001 | Bacteria | Proteobacteria | Gammaproteobacteria | unclassified | unclassified | unclassified | unclassified |
| Otu027941 | 0.8368 | 1 | 0.915 | 0.016 | Archaea | Crenarchaeota | Thaumarchaeota | Cenarchaeales | Cenarchaeaceae | Nitrosopumilus | unclassified |
| Otu028358 | 0.7945 | 1 | 0.891 | 0.022 | Archaea | Crenarchaeota | Thaumarchaeota | Cenarchaeales | Cenarchaeaceae | Nitrosopumilus | unclassified |
| Otu028498 | 0.8532 | 1 | 0.924 | 0.002 | Archaea | Crenarchaeota | Thaumarchaeota | Cenarchaeales | Cenarchaeaceae | Nitrosopumilus | unclassified |
| Otu028848 | 0.7664 | 1 | 0.875 | 0.03 | Archaea | Crenarchaeota | Thaumarchaeota | Cenarchaeales | Cenarchaeaceae | Nitrosopumilus | unclassified |
| Otu029025 | 0.9334 | 1 | 0.966 | 0.001 | Bacteria | Proteobacteria | Gammaproteobacteria | unclassified | unclassified | unclassified | unclassified |
| Otu029327 | 0.8265 | 1 | 0.909 | 0.006 | Bacteria | Proteobacteria | Gammaproteobacteria | unclassified | unclassified | unclassified | unclassified |
| Otu029546 | 1 | 0.6667 | 0.816 | 0.007 | Bacteria | Proteobacteria | Gammaproteobacteria | unclassified | unclassified | unclassified | unclassified |
| Otu029599 | 0.8237 | 1 | 0.908 | 0.006 | Bacteria | Proteobacteria | Gammaproteobacteria | unclassified | unclassified | unclassified | unclassified |
| Otu029646 | 1 | 1 | 1 | 0.001 | Bacteria | Proteobacteria | Gammaproteobacteria | unclassified | unclassified | unclassified | unclassified |
| Otu030378 | 1 | 1 | 1 | 0.001 | Bacteria | Proteobacteria | Gammaproteobacteria | unclassified | unclassified | unclassified | unclassified |
| Otu030901 | 0.7696 | 1 | 0.877 | 0.024 | Archaea | Crenarchaeota | Thaumarchaeota | Cenarchaeales | Cenarchaeaceae | Nitrosopumilus | unclassified |
| Otu031597 | 0.7107 | 1 | 0.843 | 0.046 | Archaea | Crenarchaeota | Thaumarchaeota | Cenarchaeales | Cenarchaeaceae | Nitrosopumilus | unclassified |
| Otu031725 | 0.9545 | 1 | 0.977 | 0.001 | Bacteria | Proteobacteria | Gammaproteobacteria | unclassified | unclassified | unclassified | unclassified |
| Otu031773 | 1 | 1 | 1 | 0.001 | Bacteria | Proteobacteria | Gammaproteobacteria | unclassified | unclassified | unclassified | unclassified |
| Otu033291 | 0.7781 | 1 | 0.882 | 0.015 | Archaea | Crenarchaeota | Thaumarchaeota | Cenarchaeales | Cenarchaeaceae | Nitrosopumilus | unclassified |
| Otu033688 | 0.839 | 0.6667 | 0.748 | 0.029 | Bacteria | Proteobacteria | Alphaproteobacteria | Rhodospirillales | Rhodospirillaceae | unclassified | unclassified |
| Otu035248 | 0.82 | 1 | 0.906 | 0.014 | Archaea | Crenarchaeota | Thaumarchaeota | Cenarchaeales | Cenarchaeaceae | Nitrosopumilus | unclassified |
| Otu035838 | 0.8959 | 1 | 0.947 | 0.006 | Archaea | Crenarchaeota | Thaumarchaeota | Cenarchaeales | Cenarchaeaceae | Nitrosopumilus | unclassified |
| Otu036023 | 0.9914 | 1 | 0.996 | 0.001 | Bacteria | Proteobacteria | unclassified | unclassified | unclassified | unclassified | unclassified |
| Otu036104 | 1 | 1 | 1 | 0.001 | Bacteria | Proteobacteria | unclassified | unclassified | unclassified | unclassified | unclassified |
| Otu036260 | 0.7909 | 1 | 0.889 | 0.006 | Bacteria | Proteobacteria | unclassified | unclassified | unclassified | unclassified | unclassified |
| Otu036397 | 0.9569 | 1 | 0.978 | 0.001 | Bacteria | Proteobacteria | unclassified | unclassified | unclassified | unclassified | unclassified |
| Otu036661 | 0.9522 | 1 | 0.976 | 0.001 | Bacteria | Proteobacteria | unclassified | unclassified | unclassified | unclassified | unclassified |
| Otu036668 | 1 | 1 | 1 | 0.001 | Bacteria | Proteobacteria | unclassified | unclassified | unclassified | unclassified | unclassified |
| Otu036734 | 1 | 1 | 1 | 0.001 | Bacteria | Proteobacteria | unclassified | unclassified | unclassified | unclassified | unclassified |
| Otu036806 | 0.83 | 1 | 0.911 | 0.006 | Bacteria | Proteobacteria | unclassified | unclassified | unclassified | unclassified | unclassified |
| Otu037189 | 0.8241 | 1 | 0.908 | 0.006 | Archaea | Crenarchaeota | Thaumarchaeota | Cenarchaeales | Cenarchaeaceae | Nitrosopumilus | unclassified |
| Otu037287 | 0.7942 | 1 | 0.891 | 0.019 | Archaea | Crenarchaeota | Thaumarchaeota | Cenarchaeales | Cenarchaeaceae | Nitrosopumilus | unclassified |
| Otu038151 | 0.8636 | 1 | 0.929 | 0.005 | Archaea | Crenarchaeota | Thaumarchaeota | Cenarchaeales | Cenarchaeaceae | Nitrosopumilus | unclassified |
| Otu038409 | 0.9385 | 0.6667 | 0.791 | 0.016 | Bacteria | Proteobacteria | Gammaproteobacteria | unclassified | unclassified | unclassified | unclassified |
| Otu040608 | 0.7539 | 1 | 0.868 | 0.027 | Archaea | Crenarchaeota | Thaumarchaeota | Cenarchaeales | Cenarchaeaceae | Nitrosopumilus | unclassified |
| Otu043432 | 0.8129 | 1 | 0.902 | 0.017 | Archaea | Crenarchaeota | Thaumarchaeota | Cenarchaeales | Cenarchaeaceae | Nitrosopumilus | unclassified |
| Otu044090 | 1 | 1 | 1 | 0.001 | Bacteria | Proteobacteria | Gammaproteobacteria | unclassified | unclassified | unclassified | unclassified |
| Otu044442 | 0.8042 | 1 | 0.897 | 0.008 | Bacteria | Proteobacteria | unclassified | unclassified | unclassified | unclassified | unclassified |
| Otu044453 | 0.8635 | 1 | 0.929 | 0.006 | Bacteria | Proteobacteria | unclassified | unclassified | unclassified | unclassified | unclassified |
| Otu044732 | 0.827 | 1 | 0.909 | 0.006 | Bacteria | Proteobacteria | unclassified | unclassified | unclassified | unclassified | unclassified |
| Otu044910 | 1 | 1 | 1 | 0.001 | Bacteria | Proteobacteria | unclassified | unclassified | unclassified | unclassified | unclassified |
| Otu045070 | 0.8825 | 1 | 0.939 | 0.002 | Bacteria | Proteobacteria | Alphaproteobacteria | unclassified | unclassified | unclassified | unclassified |
| Otu046372 | 0.8662 | 1 | 0.931 | 0.008 | Archaea | Crenarchaeota | Thaumarchaeota | Cenarchaeales | Cenarchaeaceae | Nitrosopumilus | unclassified |
| Otu046395 | 0.769 | 1 | 0.877 | 0.019 | Archaea | Crenarchaeota | Thaumarchaeota | Cenarchaeales | Cenarchaeaceae | Nitrosopumilus | unclassified |
| Otu047308 | 0.8296 | 1 | 0.911 | 0.017 | Archaea | Crenarchaeota | Thaumarchaeota | Cenarchaeales | Cenarchaeaceae | Nitrosopumilus | unclassified |
| Otu047334 | 0.7879 | 1 | 0.888 | 0.019 | Archaea | Crenarchaeota | Thaumarchaeota | Cenarchaeales | Cenarchaeaceae | Nitrosopumilus | unclassified |
| Otu047355 | 0.7171 | 1 | 0.847 | 0.039 | Archaea | Crenarchaeota | Thaumarchaeota | Cenarchaeales | Cenarchaeaceae | Nitrosopumilus | unclassified |
| Otu048080 | 0.9376 | 1 | 0.968 | 0.002 | Bacteria | Proteobacteria | unclassified | unclassified | unclassified | unclassified | unclassified |
| Otu048942 | 0.7843 | 1 | 0.886 | 0.022 | Archaea | Crenarchaeota | Thaumarchaeota | Cenarchaeales | Cenarchaeaceae | Nitrosopumilus | unclassified |
| Otu049309 | 0.7725 | 1 | 0.879 | 0.026 | Archaea | Crenarchaeota | Thaumarchaeota | Cenarchaeales | Cenarchaeaceae | Nitrosopumilus | unclassified |
| Otu049800 | 1 | 0.6667 | 0.816 | 0.013 | Archaea | Crenarchaeota | Thaumarchaeota | Cenarchaeales | Cenarchaeaceae | Nitrosopumilus | unclassified |
| Otu050296 | 0.9267 | 0.6667 | 0.786 | 0.008 | Bacteria | Proteobacteria | Gammaproteobacteria | Alteromonadales | Psychromonadaceae | Psychromonas | unclassified |
| Otu050832 | 1 | 1 | 1 | 0.001 | Bacteria | Proteobacteria | unclassified | unclassified | unclassified | unclassified | unclassified |
| Otu052811 | 0.6912 | 1 | 0.831 | 0.048 | Bacteria | Proteobacteria | Gammaproteobacteria | unclassified | unclassified | unclassified | unclassified |
| Otu054005 | 1 | 0.6667 | 0.816 | 0.006 | Bacteria | Proteobacteria | Gammaproteobacteria | unclassified | unclassified | unclassified | unclassified |
| Otu055242 | 0.8594 | 0.6667 | 0.757 | 0.007 | Bacteria | Proteobacteria | Alphaproteobacteria | Rhodospirillales | Rhodospirillaceae | unclassified | unclassified |
| Otu057370 | 1 | 1 | 1 | 0.001 | Bacteria | Proteobacteria | Gammaproteobacteria | unclassified | unclassified | unclassified | unclassified |
| Otu057825 | 1 | 1 | 1 | 0.001 | Bacteria | Proteobacteria | Gammaproteobacteria | unclassified | unclassified | unclassified | unclassified |
| Otu057963 | 1 | 1 | 1 | 0.001 | Bacteria | Proteobacteria | Gammaproteobacteria | unclassified | unclassified | unclassified | unclassified |
| Otu058993 | 1 | 1 | 1 | 0.001 | Bacteria | Proteobacteria | Gammaproteobacteria | unclassified | unclassified | unclassified | unclassified |
| Otu059022 | 0.7996 | 1 | 0.894 | 0.007 | Bacteria | Proteobacteria | Alphaproteobacteria | Rhodospirillales | Rhodospirillaceae | Nisaea | unclassified |
| Otu061479 | 0.8871 | 1 | 0.942 | 0.004 | Archaea | Crenarchaeota | Thaumarchaeota | Cenarchaeales | Cenarchaeaceae | Nitrosopumilus | unclassified |
| Otu064692 | 1 | 1 | 1 | 0.001 | Bacteria | Proteobacteria | Gammaproteobacteria | unclassified | unclassified | unclassified | unclassified |
| Otu066913 | 0.8057 | 1 | 0.898 | 0.016 | Bacteria | Proteobacteria | Gammaproteobacteria | unclassified | unclassified | unclassified | unclassified |
| Otu067607 | 1 | 1 | 1 | 0.001 | Bacteria | Proteobacteria | Gammaproteobacteria | unclassified | unclassified | unclassified | unclassified |
| Otu068072 | 0.8161 | 1 | 0.903 | 0.002 | Archaea | Crenarchaeota | Thaumarchaeota | Cenarchaeales | Cenarchaeaceae | Nitrosopumilus | unclassified |
| Otu072728 | 0.8863 | 0.6667 | 0.769 | 0.038 | Bacteria | Verrucomicrobia | Verrucomicrobiae | Verrucomicrobiales | Verrucomicrobiaceae | Rubritalea | unclassified |
| Otu074070 | 0.8504 | 1 | 0.922 | 0.006 | Bacteria | Proteobacteria | Alphaproteobacteria | Rickettsiales | Pelagibacteraceae | unclassified | unclassified |
| Otu074145 | 0.8897 | 0.6667 | 0.77 | 0.025 | Bacteria | Proteobacteria | Alphaproteobacteria | Rickettsiales | Pelagibacteraceae | unclassified | unclassified |
| Otu075722 | 0.8619 | 0.6667 | 0.758 | 0.013 | Bacteria | Actinobacteria | Acidimicrobiia | Acidimicrobiales | wb1_P06 | unclassified | unclassified |
| Otu079526 | 0.9697 | 0.6667 | 0.804 | 0.006 | Bacteria | Proteobacteria | Deltaproteobacteria | unclassified | unclassified | unclassified | unclassified |
| Otu079604 | 0.9541 | 1 | 0.977 | 0.001 | Bacteria | Proteobacteria | Alphaproteobacteria | unclassified | unclassified | unclassified | unclassified |
| Otu081386 | 0.9548 | 0.6667 | 0.798 | 0.006 | Bacteria | Proteobacteria | Alphaproteobacteria | unclassified | unclassified | unclassified | unclassified |
| Otu082432 | 0.8415 | 1 | 0.917 | 0.009 | Bacteria | Proteobacteria | Betaproteobacteria | Methylophilales | Methylophilaceae | unclassified | unclassified |
| Otu083352 | 0.9111 | 0.6667 | 0.779 | 0.026 | Bacteria | Proteobacteria | Alphaproteobacteria | unclassified | unclassified | unclassified | unclassified |
| Otu090167 | 0.9914 | 1 | 0.996 | 0.001 | Bacteria | Nitrospirae | Nitrospira | Nitrospirales | Nitrospiraceae | unclassified | unclassified |
| Otu092218 | 0.8837 | 0.6667 | 0.768 | 0.009 | Archaea | Crenarchaeota | Thaumarchaeota | Cenarchaeales | Cenarchaeaceae | Nitrosopumilus | unclassified |
| Otu093768 | 0.8645 | 1 | 0.93 | 0.009 | Archaea | Crenarchaeota | Thaumarchaeota | Cenarchaeales | Cenarchaeaceae | Nitrosopumilus | unclassified |
| Otu094642 | 0.7822 | 1 | 0.884 | 0.02 | Archaea | Crenarchaeota | Thaumarchaeota | Cenarchaeales | Cenarchaeaceae | Nitrosopumilus | unclassified |
| Otu094669 | 0.8823 | 1 | 0.939 | 0.002 | Archaea | Crenarchaeota | Thaumarchaeota | Cenarchaeales | Cenarchaeaceae | Nitrosopumilus | unclassified |
| Otu094777 | 0.7802 | 1 | 0.883 | 0.023 | Archaea | Crenarchaeota | Thaumarchaeota | Cenarchaeales | Cenarchaeaceae | Nitrosopumilus | unclassified |
| Otu100239 | 0.8533 | 1 | 0.924 | 0.008 | Bacteria | unclassified | unclassified | unclassified | unclassified | unclassified | unclassified |
| Otu107337 | 1 | 1 | 1 | 0.001 | Bacteria | Proteobacteria | Gammaproteobacteria | unclassified | unclassified | unclassified | unclassified |
| Otu110882 | 1 | 1 | 1 | 0.001 | Bacteria | Proteobacteria | Gammaproteobacteria | unclassified | unclassified | unclassified | unclassified |
| Otu112347 | 1 | 0.6667 | 0.816 | 0.007 | Bacteria | Proteobacteria | unclassified | unclassified | unclassified | unclassified | unclassified |
| Otu115929 | 0.8772 | 0.6667 | 0.765 | 0.031 | Bacteria | Proteobacteria | Gammaproteobacteria | unclassified | unclassified | unclassified | unclassified |

| | | | | | | | | | | | |
|---|---|---|---|---|---|---|---|---|---|---|---|
| Otu116753 | 1 | 1 | 1 | 0.001 | Bacteria | Proteobacteria | Gammaproteobacteria | unclassified | unclassified | unclassified | unclassified |
| Otu118238 | 0.7608 | 1 | 0.872 | 0.033 | Bacteria | Bacteroidetes | Flavobacteriia | Flavobacteriales | Flavobacteriaceae | Polaribacter | unclassified |
| Otu119281 | 0.8665 | 1 | 0.931 | 0.003 | Bacteria | Bacteroidetes | Flavobacteriia | Flavobacteriales | unclassified | unclassified | unclassified |
| Otu120666 | 1 | 1 | 1 | 0.001 | Bacteria | Proteobacteria | Gammaproteobacteria | Thiotrichales | unclassified | unclassified | unclassified |
| Otu121987 | 1 | 1 | 1 | 0.001 | Bacteria | Proteobacteria | Gammaproteobacteria | unclassified | unclassified | unclassified | unclassified |
| Otu122119 | 1 | 1 | 1 | 0.001 | Bacteria | Proteobacteria | Gammaproteobacteria | unclassified | unclassified | unclassified | unclassified |
| Otu123464 | 0.8142 | 1 | 0.902 | 0.008 | Bacteria | Proteobacteria | Gammaproteobacteria | Thiohalorhabdales | Thiohalorhabdaceae | unclassified | unclassified |
| Otu124114 | 0.9528 | 1 | 0.976 | 0.001 | Bacteria | Proteobacteria | unclassified | unclassified | unclassified | unclassified | unclassified |
| Otu124607 | 1 | 0.6667 | 0.816 | 0.013 | Bacteria | Proteobacteria | Gammaproteobacteria | unclassified | unclassified | unclassified | unclassified |
| Otu125440 | 0.8949 | 1 | 0.946 | 0.002 | Bacteria | Proteobacteria | Deltaproteobacteria | Bdellovibrionales | Bacteriovoracaceae | Bacteriovorax | unclassified |
| Otu125598 | 0.8627 | 0.6667 | 0.758 | 0.019 | Bacteria | Proteobacteria | Gammaproteobacteria | Alteromonadales | unclassified | unclassified | unclassified |
| Otu125685 | 0.8935 | 1 | 0.945 | 0.002 | Bacteria | Planctomycetes | Planctomycetia | Planctomycetales | Planctomycetaceae | Planctomyces | unclassified |
| Otu125946 | 0.8897 | 0.6667 | 0.77 | 0.031 | Bacteria | Proteobacteria | Gammaproteobacteria | unclassified | unclassified | unclassified | unclassified |
| Otu126478 | 0.8173 | 1 | 0.904 | 0.002 | Bacteria | Proteobacteria | Gammaproteobacteria | Alteromonadales | unclassified | unclassified | unclassified |
| Otu130491 | 0.6248 | 1 | 0.79 | 0.048 | Bacteria | Proteobacteria | Gammaproteobacteria | HTCC2188 | HTCC2089 | unclassified | unclassified |
| Otu130640 | 0.8465 | 1 | 0.92 | 0.003 | Bacteria | Proteobacteria | Alphaproteobacteria | Ellin329 | unclassified | unclassified | unclassified |
| Otu133790 | 0.9066 | 1 | 0.952 | 0.002 | Bacteria | Verrucomicrobia | Pedosphaerae | Arctic97B-4 | unclassified | unclassified | unclassified |
| Otu134560 | 1 | 1 | 1 | 0.001 | Bacteria | Proteobacteria | Gammaproteobacteria | unclassified | unclassified | unclassified | unclassified |
| Otu137791 | 0.9578 | 1 | 0.979 | 0.001 | Bacteria | Proteobacteria | unclassified | unclassified | unclassified | unclassified | unclassified |
| Otu143398 | 0.921 | 0.6667 | 0.784 | 0.036 | Bacteria | Bacteroidetes | Flavobacteriia | Flavobacteriales | unclassified | unclassified | unclassified |
| Otu143535 | 0.8383 | 0.6667 | 0.748 | 0.045 | Bacteria | Bacteroidetes | Flavobacteriia | Flavobacteriales | Flavobacteriaceae | unclassified | unclassified |
| Otu156948 | 0.8637 | 1 | 0.929 | 0.004 | Bacteria | Planctomycetes | Planctomycetia | Planctomycetales | Planctomycetaceae | Planctomyces | unclassified |
| Otu175422 | 0.8524 | 1 | 0.923 | 0.008 | Bacteria | Proteobacteria | unclassified | unclassified | unclassified | unclassified | unclassified |
| Otu178604 | 0.887 | 1 | 0.942 | 0.002 | Bacteria | Proteobacteria | Gammaproteobacteria | unclassified | unclassified | unclassified | unclassified |
| Otu183235 | 0.9324 | 0.6667 | 0.788 | 0.025 | Bacteria | Proteobacteria | Gammaproteobacteria | unclassified | unclassified | unclassified | unclassified |
| Otu195480 | 0.9182 | 0.6667 | 0.782 | 0.024 | Bacteria | Bacteroidetes | Flavobacteriia | Flavobacteriales | Flavobacteriaceae | unclassified | unclassified |
| Otu199757 | 0.9375 | 0.6667 | 0.791 | 0.013 | Bacteria | Proteobacteria | Gammaproteobacteria | unclassified | unclassified | unclassified | unclassified |
| Otu205951 | 0.9455 | 0.6667 | 0.794 | 0.016 | Bacteria | Proteobacteria | Gammaproteobacteria | Oceanospirillales | Oceanospirillaceae | unclassified | unclassified |
| Otu208204 | 1 | 1 | 1 | 0.001 | Bacteria | Proteobacteria | Gammaproteobacteria | unclassified | unclassified | unclassified | unclassified |
| Otu212792 | 1 | 0.6667 | 0.816 | 0.006 | Bacteria | Bacteroidetes | Flavobacteriia | Flavobacteriales | unclassified | unclassified | unclassified |
| Otu215056 | 0.8604 | 0.6667 | 0.757 | 0.01 | Bacteria | Proteobacteria | Gammaproteobacteria | Oceanospirillales | Halomonadaceae | Candidatus_Portiera | unclassified |
| Otu217664 | 1 | 0.6667 | 0.816 | 0.007 | Bacteria | Proteobacteria | Gammaproteobacteria | unclassified | unclassified | unclassified | unclassified |
| Otu228123 | 0.9621 | 1 | 0.981 | 0.001 | Bacteria | Proteobacteria | Alphaproteobacteria | Rickettsiales | AEGEAN_112 | unclassified | unclassified |
| Otu228410 | 0.9403 | 1 | 0.97 | 0.001 | Bacteria | Proteobacteria | Alphaproteobacteria | unclassified | unclassified | unclassified | unclassified |
| Otu231175 | 0.9357 | 1 | 0.967 | 0.002 | Bacteria | Planctomycetes | Planctomycetia | Planctomycetales | Planctomycetaceae | Planctomyces | unclassified |
| Otu232670 | 0.9276 | 1 | 0.963 | 0.001 | Bacteria | Planctomycetes | Planctomycetia | Pirellulales | Pirellulaceae | unclassified | unclassified |
| Otu253916 | 1 | 1 | 1 | 0.001 | Bacteria | Proteobacteria | Gammaproteobacteria | unclassified | unclassified | unclassified | unclassified |
| Otu258316 | 1 | 0.6667 | 0.816 | 0.013 | Bacteria | Proteobacteria | Gammaproteobacteria | unclassified | unclassified | unclassified | unclassified |
| Otu275279 | 0.9274 | 0.6667 | 0.786 | 0.019 | Archaea | Crenarchaeota | Thaumarchaeota | Cenarchaeales | Cenarchaeaceae | Nitrosopumilus | pIVWA5 |
| Otu319725 | 0.8864 | 1 | 0.942 | 0.002 | Archaea | Euryarchaeota | Thermoplasmata | E2 | Marine_group_II | unclassified | unclassified |
| **0.03 Otus** | ***Myxilla rosacea*** | **Rest** | **stat** | ***p value*** | **Domain** | **Phylum** | **Class** | **Order** | **Family** | **Genus** | **Species** |
| Otu000022 | 0.9977 | 1 | 0.999 | 0.001 | Bacteria | Proteobacteria | Alphaproteobacteria | unclassified | unclassified | unclassified | unclassified |
| Otu000368 | 0.9976 | 1 | 0.999 | 0.001 | Bacteria | Proteobacteria | Alphaproteobacteria | Rhizobiales | unclassified | unclassified | unclassified |
| Otu000638 | 0.9437 | 1 | 0.971 | 0.003 | Bacteria | Proteobacteria | Gammaproteobacteria | Oceanospirillales | unclassified | unclassified | unclassified |
| Otu000806 | 1 | 1 | 1 | 0.001 | Bacteria | Proteobacteria | Gammaproteobacteria | unclassified | unclassified | unclassified | unclassified |
| Otu002056 | 1 | 1 | 1 | 0.001 | Bacteria | Proteobacteria | Gammaproteobacteria | Oceanospirillales | Endozoicimonaceae | unclassified | unclassified |
| Otu002523 | 0.7497 | 0.6667 | 0.707 | 0.042 | Bacteria | Verrucomicrobia | Verrucomicrobiae | Verrucomicrobiales | Verrucomicrobiaceae | unclassified | unclassified |
| Otu002801 | 1 | 0.6667 | 0.816 | 0.015 | Bacteria | Proteobacteria | Gammaproteobacteria | unclassified | unclassified | unclassified | unclassified |
| Otu002915 | 1 | 1 | 1 | 0.001 | Bacteria | Proteobacteria | Gammaproteobacteria | unclassified | unclassified | unclassified | unclassified |
| Otu002980 | 0.8902 | 0.6667 | 0.77 | 0.03 | Bacteria | Proteobacteria | Gammaproteobacteria | Oceanospirillales | Endozoicimonaceae | unclassified | unclassified |
| Otu003504 | 0.9985 | 1 | 0.999 | 0.001 | Bacteria | Proteobacteria | Alphaproteobacteria | unclassified | unclassified | unclassified | unclassified |
| Otu004203 | 0.7974 | 1 | 0.893 | 0.024 | Bacteria | Planctomycetes | Planctomycetia | Planctomycetales | Planctomycetaceae | Planctomyces | unclassified |
| Otu004570 | 1 | 0.6667 | 0.816 | 0.009 | Bacteria | Proteobacteria | Gammaproteobacteria | unclassified | unclassified | unclassified | unclassified |
| Otu005253 | 0.707 | 1 | 0.841 | 0.029 | Bacteria | Proteobacteria | Alphaproteobacteria | unclassified | unclassified | unclassified | unclassified |
| Otu005343 | 0.6856 | 1 | 0.828 | 0.032 | Bacteria | Proteobacteria | Gammaproteobacteria | Vibrionales | Vibrionaceae | Photobacterium | angustum |
| Otu006034 | 1 | 0.6667 | 0.816 | 0.015 | Bacteria | Proteobacteria | Betaproteobacteria | Burkholderiales | Comamonadaceae | unclassified | unclassified |
| Otu006475 | 1 | 0.6667 | 0.816 | 0.009 | Bacteria | Proteobacteria | Gammaproteobacteria | unclassified | unclassified | unclassified | unclassified |
| Otu006817 | 0.8017 | 1 | 0.895 | 0.014 | Bacteria | Planctomycetes | Phycisphaerae | Phycisphaerales | unclassified | unclassified | unclassified |
| Otu007313 | 1 | 0.6667 | 0.816 | 0.008 | Bacteria | Proteobacteria | Gammaproteobacteria | Oceanospirillales | Endozoicimonaceae | unclassified | unclassified |
| Otu007357 | 0.865 | 0.6667 | 0.759 | 0.049 | Bacteria | Proteobacteria | unclassified | unclassified | unclassified | unclassified | unclassified |
| Otu007496 | 0.7324 | 1 | 0.856 | 0.037 | Bacteria | Bacteroidetes | Flavobacteriia | Flavobacteriales | Flavobacteriaceae | unclassified | unclassified |
| Otu007928 | 0.7859 | 1 | 0.887 | 0.016 | Bacteria | Actinobacteria | unclassified | unclassified | unclassified | unclassified | unclassified |
| Otu008008 | 0.7442 | 1 | 0.863 | 0.019 | Bacteria | Actinobacteria | Actinobacteria | unclassified | unclassified | unclassified | unclassified |
| Otu008061 | 0.799 | 0.6667 | 0.73 | 0.029 | Bacteria | Cyanobacteria | Synechococcophycideae | Synechococcales | Synechococcaceae | unclassified | unclassified |
| Otu008310 | 0.6942 | 1 | 0.833 | 0.031 | Bacteria | Planctomycetes | Planctomycetia | Planctomycetales | Planctomycetaceae | Planctomyces | unclassified |
| Otu008632 | 1 | 0.6667 | 0.816 | 0.008 | Bacteria | Proteobacteria | Gammaproteobacteria | Oceanospirillales | Endozoicimonaceae | unclassified | unclassified |
| Otu008828 | 0.9495 | 0.6667 | 0.796 | 0.009 | Bacteria | Proteobacteria | Gammaproteobacteria | unclassified | unclassified | unclassified | unclassified |
| Otu008855 | 1 | 0.6667 | 0.816 | 0.009 | Bacteria | Proteobacteria | Gammaproteobacteria | Oceanospirillales | Endozoicimonaceae | unclassified | unclassified |
| Otu011866 | 1 | 0.6667 | 0.816 | 0.009 | Bacteria | Proteobacteria | Gammaproteobacteria | Oceanospirillales | Endozoicimonaceae | unclassified | unclassified |
| Otu012379 | 1 | 1 | 1 | 0.001 | Bacteria | Proteobacteria | Deltaproteobacteria | Bdellovibrionales | Bdellovibrionaceae | Bdellovibrio | unclassified |
| Otu012690 | 0.87 | 1 | 0.933 | 0.002 | Bacteria | Planctomycetes | Planctomycetia | Pirellulales | Pirellulaceae | unclassified | unclassified |
| Otu013094 | 1 | 0.6667 | 0.816 | 0.009 | Bacteria | Proteobacteria | Gammaproteobacteria | unclassified | unclassified | unclassified | unclassified |
| Otu013176 | 0.8433 | 1 | 0.918 | 0.007 | Bacteria | Proteobacteria | Gammaproteobacteria | Legionellales | Legionellaceae | unclassified | unclassified |
| Otu016360 | 1 | 1 | 1 | 0.001 | Bacteria | Proteobacteria | Alphaproteobacteria | Rhizobiales | unclassified | unclassified | unclassified |
| Otu016409 | 0.9679 | 0.6667 | 0.803 | 0.009 | Bacteria | Planctomycetes | Planctomycetia | Pirellulales | Pirellulaceae | unclassified | unclassified |
| Otu016422 | 1 | 1 | 1 | 0.001 | Bacteria | Proteobacteria | Alphaproteobacteria | Rhizobiales | unclassified | unclassified | unclassified |
| Otu016469 | 1 | 1 | 1 | 0.001 | Bacteria | Proteobacteria | Alphaproteobacteria | unclassified | unclassified | unclassified | unclassified |
| Otu016565 | 1 | 1 | 1 | 0.001 | Bacteria | Proteobacteria | Alphaproteobacteria | unclassified | unclassified | unclassified | unclassified |
| Otu016993 | 1 | 1 | 1 | 0.001 | Bacteria | Proteobacteria | Gammaproteobacteria | unclassified | unclassified | unclassified | unclassified |
| Otu017696 | 0.8335 | 1 | 0.913 | 0.006 | Bacteria | Proteobacteria | Gammaproteobacteria | Legionellales | Legionellaceae | unclassified | unclassified |
| Otu018142 | 1 | 0.6667 | 0.816 | 0.008 | Bacteria | Cyanobacteria | Synechococcophycideae | Synechococcales | Synechococcaceae | unclassified | unclassified |
| Otu018447 | 0.8354 | 0.6667 | 0.746 | 0.049 | Bacteria | Firmicutes | Clostridia | Clostridiales | Clostridiaceae | Clostridium | unclassified |
| Otu019043 | 0.772 | 1 | 0.879 | 0.006 | Bacteria | Proteobacteria | Gammaproteobacteria | unclassified | unclassified | unclassified | unclassified |
| Otu022040 | 0.8076 | 1 | 0.899 | 0.013 | Bacteria | Planctomycetes | Phycisphaerae | Phycisphaerales | unclassified | unclassified | unclassified |
| Otu022340 | 0.7946 | 1 | 0.891 | 0.013 | Bacteria | Actinobacteria | Actinobacteria | Actinomycetales | unclassified | unclassified | unclassified |
| Otu024307 | 1 | 1 | 1 | 0.001 | Bacteria | Proteobacteria | Alphaproteobacteria | unclassified | unclassified | unclassified | unclassified |
| Otu029487 | 0.9931 | 1 | 0.997 | 0.001 | Bacteria | Proteobacteria | Gammaproteobacteria | Oceanospirillales | Endozoicimonaceae | unclassified | unclassified |
| Otu030844 | 1 | 0.6667 | 0.816 | 0.008 | Bacteria | Bacteroidetes | Saprospirae | Saprospirales | Saprospiraceae | unclassified | unclassified |
| Otu043479 | 1 | 1 | 1 | 0.001 | Bacteria | Proteobacteria | Alphaproteobacteria | unclassified | unclassified | unclassified | unclassified |
| Otu050918 | 0.9146 | 1 | 0.956 | 0.001 | Bacteria | Actinobacteria | unclassified | unclassified | unclassified | unclassified | unclassified |
| Otu052820 | 0.9775 | 1 | 0.989 | 0.001 | Bacteria | Proteobacteria | Gammaproteobacteria | unclassified | unclassified | unclassified | unclassified |
| Otu053011 | 0.7362 | 1 | 0.858 | 0.013 | Bacteria | Bacteroidetes | Flavobacteriia | Flavobacteriales | unclassified | unclassified | unclassified |
| Otu055869 | 0.9929 | 1 | 0.996 | 0.001 | Bacteria | Proteobacteria | Alphaproteobacteria | unclassified | unclassified | unclassified | unclassified |
| Otu056201 | 1 | 1 | 1 | 0.001 | Bacteria | Proteobacteria | Alphaproteobacteria | unclassified | unclassified | unclassified | unclassified |
| Otu065269 | 0.8272 | 0.6667 | 0.743 | 0.034 | Bacteria | Cyanobacteria | Synechococcophycideae | Synechococcales | Synechococcaceae | unclassified | unclassified |
| Otu080389 | 1 | 1 | 1 | 0.001 | Bacteria | Proteobacteria | Alphaproteobacteria | unclassified | unclassified | unclassified | unclassified |
| Otu086960 | 1 | 1 | 1 | 0.001 | Bacteria | Proteobacteria | Alphaproteobacteria | unclassified | unclassified | unclassified | unclassified |
| Otu109135 | 1 | 1 | 1 | 0.001 | Bacteria | Proteobacteria | Gammaproteobacteria | Oceanospirillales | unclassified | unclassified | unclassified |
| Otu119155 | 0.969 | 1 | 0.984 | 0.001 | Bacteria | Cyanobacteria | Synechococcophycideae | Synechococcales | Synechococcaceae | Synechococcus | unclassified |
| Otu119408 | 0.7888 | 0.6667 | 0.725 | 0.043 | Bacteria | Bacteroidetes | Flavobacteriia | Flavobacteriales | Flavobacteriaceae | Olleya | unclassified |

| | | | | | | | | | | | |
|---|---|---|---|---|---|---|---|---|---|---|---|
| Otu137044 | 0.8976 | 1 | 0.947 | 0.004 | Bacteria | Proteobacteria | unclassified | unclassified | unclassified | unclassified | unclassified |
| Otu139538 | 0.8622 | 0.6667 | 0.758 | 0.047 | Bacteria | Proteobacteria | Deltaproteobacteria | Myxococcales | unclassified | unclassified | unclassified |
| Otu143840 | 0.9575 | 0.6667 | 0.799 | 0.015 | Bacteria | Actinobacteria | Actinobacteria | Actinomycetales | unclassified | unclassified | unclassified |
| Otu235837 | 1 | 0.6667 | 0.816 | 0.008 | Bacteria | Proteobacteria | Gammaproteobacteria | Oceanospirillales | Endozoicimonaceae | unclassified | unclassified |

| **0.03 Otus** | ***Mycale lingua*** | **Rest** | **stat** | ***p value*** | **Domain** | **Phylum** | **Class** | **Order** | **Family** | **Genus** | **Species** |
|---|---|---|---|---|---|---|---|---|---|---|---|
| Otu000019 | 1 | 1 | 1 | 0.001 | Bacteria | PAUC34f | unclassified | unclassified | unclassified | unclassified | unclassified |
| Otu000027 | 1 | 0.6667 | 0.816 | 0.011 | Bacteria | PAUC34f | unclassified | unclassified | unclassified | unclassified | unclassified |
| Otu000086 | 1 | 0.6667 | 0.816 | 0.011 | Bacteria | Proteobacteria | Gammaproteobacteria | unclassified | unclassified | unclassified | unclassified |
| Otu000087 | 0.866 | 1 | 0.931 | 0.006 | Bacteria | Proteobacteria | Gammaproteobacteria | unclassified | unclassified | unclassified | unclassified |
| Otu000094 | 1 | 0.6667 | 0.816 | 0.008 | Bacteria | Chloroflexi | SAR202 | unclassified | unclassified | unclassified | unclassified |
| Otu000102 | 1 | 0.6667 | 0.816 | 0.008 | Bacteria | Proteobacteria | Gammaproteobacteria | unclassified | unclassified | unclassified | unclassified |
| Otu000112 | 1 | 0.6667 | 0.816 | 0.008 | Bacteria | unclassified | unclassified | unclassified | unclassified | unclassified | unclassified |
| Otu000123 | 1 | 1 | 1 | 0.001 | Bacteria | Chloroflexi | Anaerolineae | Caldilineales | Caldilineaceae | unclassified | unclassified |
| Otu000125 | 0.9937 | 1 | 0.997 | 0.001 | Bacteria | Proteobacteria | Gammaproteobacteria | unclassified | unclassified | unclassified | unclassified |
| Otu000137 | 1 | 0.6667 | 0.816 | 0.011 | Bacteria | Chloroflexi | Ktedonobacteria | TK10 | unclassified | unclassified | unclassified |
| Otu000143 | 1 | 1 | 1 | 0.001 | Bacteria | Proteobacteria | Betaproteobacteria | unclassified | unclassified | unclassified | unclassified |
| Otu000159 | 0.9354 | 0.6667 | 0.79 | 0.014 | Bacteria | Actinobacteria | Acidimicrobiia | Acidimicrobiales | TK06 | unclassified | unclassified |
| Otu000162 | 0.9941 | 1 | 0.997 | 0.001 | Bacteria | Firmicutes | Bacilli | Bacillales | Alicyclobacillaceae | Alicyclobacillus | unclassified |
| Otu000182 | 1 | 0.6667 | 0.816 | 0.011 | Bacteria | Acidobacteria | Solibacteres | Solibacterales | PAUC26f | unclassified | unclassified |
| Otu000197 | 1 | 0.6667 | 0.816 | 0.008 | Bacteria | Actinobacteria | Acidimicrobiia | Acidimicrobiales | unclassified | unclassified | unclassified |
| Otu000203 | 0.9811 | 0.6667 | 0.809 | 0.008 | Bacteria | Firmicutes | Clostridia | Clostridiales | Clostridiaceae | SMB53 | unclassified |
| Otu000214 | 1 | 0.6667 | 0.816 | 0.011 | Bacteria | Acidobacteria | Acidobacteria-6 | iii1-15 | unclassified | unclassified | unclassified |
| Otu000218 | 0.7729 | 1 | 0.879 | 0.007 | Bacteria | Acidobacteria | PAUC37f | unclassified | unclassified | unclassified | unclassified |
| Otu000222 | 1 | 0.6667 | 0.816 | 0.008 | Bacteria | Cyanobacteria | Synechococcophycideae | Synechococcales | Synechococcaceae | Synechococcus | unclassified |
| Otu000230 | 0.9921 | 1 | 0.996 | 0.001 | Bacteria | Firmicutes | Bacilli | Bacillales | Staphylococcaceae | Staphylococcus | haemolyticus |
| Otu000238 | 0.9776 | 1 | 0.989 | 0.001 | Bacteria | Proteobacteria | Deltaproteobacteria | Entotheonellales | Entotheonellaceae | Candidatus_Entotheonell | unclassified |
| Otu000240 | 0.9928 | 1 | 0.996 | 0.001 | Bacteria | Proteobacteria | Gammaproteobacteria | Enterobacteriales | Enterobacteriaceae | Erwinia | unclassified |
| Otu000263 | 0.9674 | 1 | 0.984 | 0.001 | Bacteria | AncK6 | unclassified | unclassified | unclassified | unclassified | unclassified |
| Otu000268 | 1 | 0.6667 | 0.816 | 0.008 | Bacteria | Chloroflexi | SAR202 | unclassified | unclassified | unclassified | unclassified |
| Otu000277 | 1 | 1 | 1 | 0.001 | Bacteria | Proteobacteria | unclassified | unclassified | unclassified | unclassified | unclassified |
| Otu000279 | 1 | 0.6667 | 0.816 | 0.011 | Bacteria | Proteobacteria | Deltaproteobacteria | Syntrophobacterales | Syntrophobacteraceae | unclassified | unclassified |
| Otu000299 | 0.9043 | 0.6667 | 0.776 | 0.029 | Bacteria | Chloroflexi | SAR202 | unclassified | unclassified | unclassified | unclassified |
| Otu000317 | 1 | 1 | 1 | 0.001 | Bacteria | Chloroflexi | Anaerolineae | Caldilineales | Caldilineaceae | unclassified | unclassified |
| Otu000318 | 1 | 0.6667 | 0.816 | 0.008 | Bacteria | Gemmatimonadetes | Gemm-2 | unclassified | unclassified | unclassified | unclassified |
| Otu000319 | 1 | 0.6667 | 0.816 | 0.008 | Bacteria | Nitrospirae | Nitrospira | Nitrospirales | Nitrospiraceae | unclassified | unclassified |
| Otu000321 | 0.9387 | 1 | 0.969 | 0.001 | Bacteria | Proteobacteria | Gammaproteobacteria | Pseudomonadales | Moraxellaceae | Acinetobacter | unclassified |
| Otu000336 | 1 | 1 | 1 | 0.001 | Bacteria | Chloroflexi | SAR202 | unclassified | unclassified | unclassified | unclassified |
| Otu000353 | 1 | 0.6667 | 0.816 | 0.008 | Bacteria | Chloroflexi | SAR202 | unclassified | unclassified | unclassified | unclassified |
| Otu000359 | 0.8986 | 0.6667 | 0.774 | 0.018 | Bacteria | PAUC34f | unclassified | unclassified | unclassified | unclassified | unclassified |
| Otu000363 | 0.9772 | 0.6667 | 0.807 | 0.011 | Bacteria | Acidobacteria | PAUC37f | unclassified | unclassified | unclassified | unclassified |
| Otu000383 | 1 | 0.6667 | 0.816 | 0.011 | Bacteria | Chloroflexi | SAR202 | unclassified | unclassified | unclassified | unclassified |
| Otu000385 | 1 | 0.6667 | 0.816 | 0.008 | Bacteria | Proteobacteria | Deltaproteobacteria | Syntrophobacterales | Syntrophobacteraceae | unclassified | unclassified |
| Otu000388 | 1 | 1 | 1 | 0.001 | Bacteria | Spirochaetes | Spirochaetes | Spirochaetales | Spirochaetaceae | unclassified | unclassified |
| Otu000400 | 1 | 1 | 1 | 0.001 | Bacteria | Chloroflexi | Anaerolineae | Caldilineales | Caldilineaceae | unclassified | unclassified |
| Otu000415 | 0.9692 | 1 | 0.985 | 0.001 | Bacteria | Proteobacteria | Gammaproteobacteria | Enterobacteriales | Enterobacteriaceae | unclassified | unclassified |
| Otu000418 | 1 | 0.6667 | 0.816 | 0.011 | Bacteria | Chloroflexi | SAR202 | unclassified | unclassified | unclassified | unclassified |
| Otu000427 | 1 | 0.6667 | 0.816 | 0.011 | Bacteria | Chloroflexi | unclassified | unclassified | unclassified | unclassified | unclassified |
| Otu000440 | 1 | 0.6667 | 0.816 | 0.008 | Bacteria | Proteobacteria | Gammaproteobacteria | unclassified | unclassified | unclassified | unclassified |
| Otu000485 | 1 | 0.6667 | 0.816 | 0.008 | Bacteria | unclassified | unclassified | unclassified | unclassified | unclassified | unclassified |
| Otu000496 | 1 | 0.6667 | 0.816 | 0.011 | Bacteria | Proteobacteria | Alphaproteobacteria | unclassified | unclassified | unclassified | unclassified |
| Otu000497 | 1 | 1 | 1 | 0.001 | Bacteria | Proteobacteria | Alphaproteobacteria | unclassified | unclassified | unclassified | unclassified |
| Otu000509 | 0.9887 | 1 | 0.994 | 0.001 | Bacteria | Proteobacteria | Gammaproteobacteria | unclassified | unclassified | unclassified | unclassified |
| Otu000526 | 1 | 0.6667 | 0.816 | 0.011 | Bacteria | unclassified | unclassified | unclassified | unclassified | unclassified | unclassified |
| Otu000555 | 0.9881 | 1 | 0.994 | 0.001 | Bacteria | Chloroflexi | Anaerolineae | Caldilineales | Caldilineaceae | unclassified | unclassified |
| Otu000565 | 1 | 0.6667 | 0.816 | 0.008 | Bacteria | Proteobacteria | Deltaproteobacteria | Entotheonellales | Entotheonellaceae | unclassified | unclassified |
| Otu000588 | 1 | 0.6667 | 0.816 | 0.008 | Bacteria | Proteobacteria | Gammaproteobacteria | HTCC2188 | HTCC2089 | unclassified | unclassified |
| Otu000592 | 0.9913 | 1 | 0.996 | 0.001 | Bacteria | Chloroflexi | Anaerolineae | Caldilineales | Caldilineaceae | unclassified | unclassified |
| Otu000593 | 1 | 0.6667 | 0.816 | 0.008 | Bacteria | Proteobacteria | unclassified | unclassified | unclassified | unclassified | unclassified |
| Otu000602 | 0.9732 | 0.6667 | 0.805 | 0.008 | Bacteria | unclassified | unclassified | unclassified | unclassified | unclassified | unclassified |
| Otu000604 | 1 | 1 | 1 | 0.001 | Bacteria | Proteobacteria | unclassified | unclassified | unclassified | unclassified | unclassified |
| Otu000622 | 1 | 0.6667 | 0.816 | 0.011 | Bacteria | Chloroflexi | unclassified | unclassified | unclassified | unclassified | unclassified |
| Otu000640 | 1 | 0.6667 | 0.816 | 0.011 | Bacteria | Proteobacteria | unclassified | unclassified | unclassified | unclassified | unclassified |
| Otu000650 | 0.9901 | 0.6667 | 0.812 | 0.011 | Bacteria | Proteobacteria | Gammaproteobacteria | Oceanospirillales | Endozoicimonaceae | unclassified | unclassified |
| Otu000653 | 1 | 0.6667 | 0.816 | 0.011 | Bacteria | Chloroflexi | SAR202 | unclassified | unclassified | unclassified | unclassified |
| Otu000661 | 1 | 1 | 1 | 0.001 | Bacteria | Proteobacteria | Gammaproteobacteria | unclassified | unclassified | unclassified | unclassified |
| Otu000675 | 1 | 0.6667 | 0.816 | 0.011 | Bacteria | Proteobacteria | Gammaproteobacteria | HTCC2188 | HTCC2089 | unclassified | unclassified |
| Otu000679 | 1 | 0.6667 | 0.816 | 0.008 | Bacteria | Acidobacteria | Acidobacteria-6 | iii1-15 | unclassified | unclassified | unclassified |
| Otu000682 | 1 | 0.6667 | 0.816 | 0.011 | Bacteria | Proteobacteria | Deltaproteobacteria | Entotheonellales | Entotheonellaceae | unclassified | unclassified |
| Otu000689 | 0.9771 | 0.6667 | 0.807 | 0.008 | Bacteria | Proteobacteria | Gammaproteobacteria | unclassified | unclassified | unclassified | unclassified |
| Otu000708 | 1 | 0.6667 | 0.816 | 0.008 | Bacteria | Proteobacteria | Alphaproteobacteria | Rhodobacterales | Rhodobacteraceae | unclassified | unclassified |
| Otu000716 | 1 | 0.6667 | 0.816 | 0.008 | Bacteria | Proteobacteria | Alphaproteobacteria | unclassified | unclassified | unclassified | unclassified |
| Otu000718 | 1 | 0.6667 | 0.816 | 0.011 | Bacteria | PAUC34f | unclassified | unclassified | unclassified | unclassified | unclassified |
| Otu000730 | 0.87 | 1 | 0.933 | 0.004 | Bacteria | Acidobacteria | Solibacteres | Solibacterales | PAUC26f | unclassified | unclassified |
| Otu000732 | 1 | 0.6667 | 0.816 | 0.011 | Bacteria | Proteobacteria | Deltaproteobacteria | Syntrophobacterales | Syntrophobacteraceae | unclassified | unclassified |
| Otu000748 | 1 | 0.6667 | 0.816 | 0.011 | Bacteria | unclassified | unclassified | unclassified | unclassified | unclassified | unclassified |
| Otu000765 | 1 | 0.6667 | 0.816 | 0.008 | Bacteria | unclassified | unclassified | unclassified | unclassified | unclassified | unclassified |
| Otu000795 | 0.9958 | 1 | 0.998 | 0.001 | Bacteria | Acidobacteria | Acidobacteria-6 | BPC015 | unclassified | unclassified | unclassified |
| Otu000801 | 0.9599 | 0.6667 | 0.8 | 0.011 | Bacteria | Chloroflexi | SAR202 | unclassified | unclassified | unclassified | unclassified |
| Otu000858 | 0.9962 | 1 | 0.998 | 0.001 | Bacteria | Firmicutes | Bacilli | Bacillales | Bacillaceae | Bacillus | unclassified |
| Otu000883 | 0.9803 | 0.6667 | 0.808 | 0.011 | Bacteria | Chloroflexi | SAR202 | unclassified | unclassified | unclassified | unclassified |
| Otu000889 | 0.9665 | 0.6667 | 0.803 | 0.008 | Bacteria | Acidobacteria | Sva0725 | Sva0725 | unclassified | unclassified | unclassified |
| Otu000903 | 1 | 0.6667 | 0.816 | 0.011 | Bacteria | Chloroflexi | SAR202 | unclassified | unclassified | unclassified | unclassified |
| Otu000920 | 1 | 1 | 1 | 0.001 | Bacteria | Proteobacteria | Gammaproteobacteria | unclassified | unclassified | unclassified | unclassified |
| Otu000941 | 1 | 0.6667 | 0.816 | 0.011 | Bacteria | unclassified | unclassified | unclassified | unclassified | unclassified | unclassified |
| Otu000945 | 1 | 0.6667 | 0.816 | 0.011 | Bacteria | Gemmatimonadetes | Gemm-2 | unclassified | unclassified | unclassified | unclassified |
| Otu000947 | 0.9958 | 1 | 0.998 | 0.001 | Bacteria | Proteobacteria | Gammaproteobacteria | Chromatiales | Chromatiaceae | Nitrosococcus | unclassified |
| Otu000951 | 0.969 | 0.6667 | 0.804 | 0.008 | Bacteria | Proteobacteria | Gammaproteobacteria | HTCC2188 | HTCC2089 | unclassified | unclassified |
| Otu000971 | 1 | 0.6667 | 0.816 | 0.008 | Bacteria | Nitrospirae | Nitrospira | Nitrospirales | Nitrospiraceae | unclassified | unclassified |
| Otu000979 | 1 | 0.6667 | 0.816 | 0.008 | Archaea | Crenarchaeota | Thaumarchaeota | Cenarchaeales | Cenarchaeaceae | Nitrosopumilus | unclassified |
| Otu000984 | 1 | 1 | 1 | 0.001 | Bacteria | Chloroflexi | SAR202 | unclassified | unclassified | unclassified | unclassified |
| Otu001003 | 0.9189 | 1 | 0.959 | 0.001 | Bacteria | Chloroflexi | SAR202 | unclassified | unclassified | unclassified | unclassified |
| Otu001016 | 0.8633 | 0.6667 | 0.759 | 0.044 | Bacteria | Actinobacteria | unclassified | unclassified | unclassified | unclassified | unclassified |
| Otu001017 | 1 | 0.6667 | 0.816 | 0.011 | Bacteria | unclassified | unclassified | unclassified | unclassified | unclassified | unclassified |
| Otu001092 | 0.9861 | 0.6667 | 0.811 | 0.008 | Bacteria | Proteobacteria | Gammaproteobacteria | unclassified | unclassified | unclassified | unclassified |
| Otu001105 | 1 | 0.6667 | 0.816 | 0.008 | Bacteria | Acidobacteria | Sva0725 | Sva0725 | unclassified | unclassified | unclassified |
| Otu001161 | 0.97 | 1 | 0.985 | 0.001 | Bacteria | Proteobacteria | Deltaproteobacteria | Syntrophobacterales | Syntrophobacteraceae | unclassified | unclassified |
| Otu001180 | 0.8086 | 1 | 0.899 | 0.007 | Bacteria | Acidobacteria | Acidobacteria-6 | iii1-15 | unclassified | unclassified | unclassified |
| Otu001183 | 0.9938 | 1 | 0.997 | 0.001 | Bacteria | Chloroflexi | SAR202 | unclassified | unclassified | unclassified | unclassified |
| Otu001196 | 1 | 0.6667 | 0.816 | 0.008 | Bacteria | Gemmatimonadetes | Gemm-2 | unclassified | unclassified | unclassified | unclassified |
| Otu001218 | 0.8572 | 1 | 0.926 | 0.007 | Bacteria | PAUC34f | unclassified | unclassified | unclassified | unclassified | unclassified |

| | | | | | | | | | | | |
|---|---|---|---|---|---|---|---|---|---|---|---|
| Otu001227 | 0.9399 | 0.6667 | 0.792 | 0.02 | Bacteria | Proteobacteria | Gammaproteobacteria | Chromatiales | unclassified | unclassified | unclassified |
| Otu001242 | 1 | 0.6667 | 0.816 | 0.008 | Bacteria | Proteobacteria | Alphaproteobacteria | Rhizobiales | unclassified | unclassified | unclassified |
| Otu001280 | 0.9772 | 0.6667 | 0.807 | 0.011 | Bacteria | Chloroflexi | SAR202 | unclassified | unclassified | unclassified | unclassified |
| Otu001324 | 1 | 0.6667 | 0.816 | 0.011 | Archaea | Crenarchaeota | Thaumarchaeota | Cenarchaeales | Cenarchaeaceae | Nitrosopumilus | unclassified |
| Otu001325 | 1 | 1 | 1 | 0.001 | Bacteria | Chloroflexi | SAR202 | unclassified | unclassified | unclassified | unclassified |
| Otu001332 | 1 | 0.6667 | 0.816 | 0.011 | Bacteria | Chloroflexi | Anaerolineae | SBR1031 | A4b | unclassified | unclassified |
| Otu001338 | 1 | 0.6667 | 0.816 | 0.011 | Bacteria | Chloroflexi | SAR202 | unclassified | unclassified | unclassified | unclassified |
| Otu001342 | 0.9463 | 0.6667 | 0.794 | 0.015 | Bacteria | Proteobacteria | Gammaproteobacteria | unclassified | unclassified | unclassified | unclassified |
| Otu001360 | 1 | 0.6667 | 0.816 | 0.011 | Bacteria | Chloroflexi | SAR202 | unclassified | unclassified | unclassified | unclassified |
| Otu001404 | 1 | 0.6667 | 0.816 | 0.008 | Bacteria | Chloroflexi | SAR202 | unclassified | unclassified | unclassified | unclassified |
| Otu001417 | 1 | 0.6667 | 0.816 | 0.011 | Bacteria | Actinobacteria | unclassified | unclassified | unclassified | unclassified | unclassified |
| Otu001419 | 0.9852 | 0.6667 | 0.81 | 0.011 | Bacteria | Nitrospirae | Nitrospira | Nitrospirales | Nitrospiraceae | unclassified | unclassified |
| Otu001425 | 1 | 0.6667 | 0.816 | 0.011 | Bacteria | Actinobacteria | Actinobacteria | Actinomycetales | Micrococcaceae | Micrococcus | luteus |
| Otu001447 | 1 | 1 | 1 | 0.001 | Bacteria | Chloroflexi | SAR202 | unclassified | unclassified | unclassified | unclassified |
| Otu001510 | 1 | 0.6667 | 0.816 | 0.011 | Bacteria | unclassified | unclassified | unclassified | unclassified | unclassified | unclassified |
| Otu001537 | 0.5869 | 1 | 0.766 | 0.025 | Bacteria | Proteobacteria | Gammaproteobacteria | unclassified | unclassified | unclassified | unclassified |
| Otu001547 | 0.9942 | 1 | 0.997 | 0.001 | Bacteria | Chloroflexi | SAR202 | unclassified | unclassified | unclassified | unclassified |
| Otu001549 | 1 | 0.6667 | 0.816 | 0.011 | Bacteria | Gemmatimonadetes | Gemm-2 | unclassified | unclassified | unclassified | unclassified |
| Otu001564 | 1 | 0.6667 | 0.816 | 0.011 | Bacteria | Chloroflexi | TK17 | TK18 | unclassified | unclassified | unclassified |
| Otu001566 | 1 | 0.6667 | 0.816 | 0.008 | Bacteria | PAUC34f | unclassified | unclassified | unclassified | unclassified | unclassified |
| Otu001582 | 1 | 0.6667 | 0.816 | 0.011 | Bacteria | Proteobacteria | Gammaproteobacteria | unclassified | unclassified | unclassified | unclassified |
| Otu001586 | 0.853 | 0.6667 | 0.754 | 0.031 | Bacteria | Actinobacteria | Acidimicrobiia | Acidimicrobiales | wb1_P06 | unclassified | unclassified |
| Otu001589 | 0.9887 | 1 | 0.994 | 0.001 | Bacteria | Actinobacteria | Acidimicrobiia | Acidimicrobiales | wb1_P06 | unclassified | unclassified |
| Otu001599 | 1 | 0.6667 | 0.816 | 0.011 | Bacteria | Chloroflexi | unclassified | unclassified | unclassified | unclassified | unclassified |
| Otu001616 | 1 | 0.6667 | 0.816 | 0.008 | Bacteria | Proteobacteria | Gammaproteobacteria | HTCC2188 | HTCC2089 | unclassified | unclassified |
| Otu001699 | 0.9859 | 0.6667 | 0.811 | 0.011 | Bacteria | Thermi | Deinococci | Deinococcales | Trueperaceae | B-42 | unclassified |
| Otu001716 | 1 | 0.6667 | 0.816 | 0.011 | Bacteria | Gemmatimonadetes | Gemm-2 | unclassified | unclassified | unclassified | unclassified |
| Otu001721 | 0.9936 | 1 | 0.997 | 0.001 | Bacteria | Chloroflexi | SAR202 | unclassified | unclassified | unclassified | unclassified |
| Otu001734 | 1 | 0.6667 | 0.816 | 0.011 | Bacteria | PAUC34f | unclassified | unclassified | unclassified | unclassified | unclassified |
| Otu001737 | 0.9727 | 0.6667 | 0.805 | 0.008 | Bacteria | Proteobacteria | Deltaproteobacteria | Syntrophobacterales | Syntrophobacteraceae | unclassified | unclassified |
| Otu001803 | 1 | 0.6667 | 0.816 | 0.011 | Bacteria | unclassified | unclassified | unclassified | unclassified | unclassified | unclassified |
| Otu001836 | 1 | 1 | 1 | 0.001 | Bacteria | Poribacteria | unclassified | unclassified | unclassified | unclassified | unclassified |
| Otu001865 | 0.8989 | 1 | 0.948 | 0.004 | Bacteria | Chloroflexi | SAR202 | unclassified | unclassified | unclassified | unclassified |
| Otu001913 | 1 | 0.6667 | 0.816 | 0.008 | Bacteria | Proteobacteria | Gammaproteobacteria | unclassified | unclassified | unclassified | unclassified |
| Otu001928 | 1 | 1 | 1 | 0.001 | Bacteria | Actinobacteria | Acidimicrobiia | Acidimicrobiales | unclassified | unclassified | unclassified |
| Otu001954 | 0.9799 | 1 | 0.99 | 0.001 | Bacteria | Acidobacteria | Sva0725 | Sva0725 | unclassified | unclassified | unclassified |
| Otu001955 | 1 | 0.6667 | 0.816 | 0.008 | Bacteria | Poribacteria | unclassified | unclassified | unclassified | unclassified | unclassified |
| Otu001981 | 1 | 1 | 1 | 0.001 | Bacteria | Proteobacteria | Deltaproteobacteria | Syntrophobacterales | Syntrophobacteraceae | unclassified | unclassified |
| Otu001988 | 1 | 0.6667 | 0.816 | 0.008 | Bacteria | Gemmatimonadetes | Gemm-2 | unclassified | unclassified | unclassified | unclassified |
| Otu002071 | 1 | 0.6667 | 0.816 | 0.011 | Bacteria | Chloroflexi | SAR202 | unclassified | unclassified | unclassified | unclassified |
| Otu002124 | 1 | 0.6667 | 0.816 | 0.011 | Bacteria | Proteobacteria | Alphaproteobacteria | unclassified | unclassified | unclassified | unclassified |
| Otu002126 | 0.9598 | 0.6667 | 0.8 | 0.011 | Bacteria | unclassified | unclassified | unclassified | unclassified | unclassified | unclassified |
| Otu002176 | 1 | 0.6667 | 0.816 | 0.011 | Bacteria | Proteobacteria | Gammaproteobacteria | Pseudomonadales | Moraxellaceae | Enhydrobacter | unclassified |
| Otu002183 | 0.898 | 1 | 0.948 | 0.004 | Bacteria | Proteobacteria | Gammaproteobacteria | HTCC2188 | HTCC2089 | unclassified | unclassified |
| Otu002184 | 1 | 0.6667 | 0.816 | 0.011 | Bacteria | Proteobacteria | Alphaproteobacteria | Rhodobacterales | Rhodobacteraceae | unclassified | unclassified |
| Otu002215 | 0.9565 | 0.6667 | 0.799 | 0.011 | Bacteria | Proteobacteria | Gammaproteobacteria | Alteromonadales | unclassified | unclassified | unclassified |
| Otu002216 | 1 | 0.6667 | 0.816 | 0.008 | Bacteria | Proteobacteria | Gammaproteobacteria | unclassified | unclassified | unclassified | unclassified |
| Otu002246 | 1 | 0.6667 | 0.816 | 0.008 | Bacteria | Actinobacteria | unclassified | unclassified | unclassified | unclassified | unclassified |
| Otu002293 | 1 | 0.6667 | 0.816 | 0.008 | Bacteria | Acidobacteria | Acidobacteria-6 | iii1-15 | unclassified | unclassified | unclassified |
| Otu002308 | 1 | 1 | 1 | 0.001 | Bacteria | Actinobacteria | Acidimicrobiia | Acidimicrobiales | unclassified | unclassified | unclassified |
| Otu002420 | 0.9622 | 1 | 0.981 | 0.001 | Bacteria | Gemmatimonadetes | Gemm-2 | unclassified | unclassified | unclassified | unclassified |
| Otu002449 | 1 | 1 | 1 | 0.001 | Bacteria | Actinobacteria | Actinobacteria | Actinomycetales | Corynebacteriaceae | Corynebacterium | unclassified |
| Otu002531 | 0.8974 | 0.6667 | 0.773 | 0.017 | Bacteria | Acidobacteria | PAUC37f | unclassified | unclassified | unclassified | unclassified |
| Otu002540 | 1 | 0.6667 | 0.816 | 0.008 | Bacteria | Proteobacteria | Gammaproteobacteria | HTCC2188 | HTCC2089 | unclassified | unclassified |
| Otu002569 | 1 | 0.6667 | 0.816 | 0.008 | Bacteria | Proteobacteria | Gammaproteobacteria | unclassified | unclassified | unclassified | unclassified |
| Otu002623 | 1 | 1 | 1 | 0.001 | Bacteria | Chloroflexi | SAR202 | unclassified | unclassified | unclassified | unclassified |
| Otu002736 | 0.8066 | 1 | 0.898 | 0.007 | Bacteria | Actinobacteria | Acidimicrobiia | Acidimicrobiales | wb1_P06 | unclassified | unclassified |
| Otu002829 | 0.9311 | 1 | 0.965 | 0.003 | Bacteria | Chloroflexi | SAR202 | unclassified | unclassified | unclassified | unclassified |
| Otu002853 | 1 | 0.6667 | 0.816 | 0.011 | Bacteria | Poribacteria | unclassified | unclassified | unclassified | unclassified | unclassified |
| Otu002934 | 1 | 0.6667 | 0.816 | 0.008 | Bacteria | Chloroflexi | SAR202 | unclassified | unclassified | unclassified | unclassified |
| Otu003200 | 1 | 0.6667 | 0.816 | 0.008 | Bacteria | Proteobacteria | Gammaproteobacteria | unclassified | unclassified | unclassified | unclassified |
| Otu003264 | 1 | 0.6667 | 0.816 | 0.008 | Bacteria | SBR1093 | EC214 | unclassified | unclassified | unclassified | unclassified |
| Otu003276 | 1 | 0.6667 | 0.816 | 0.011 | Bacteria | Proteobacteria | Alphaproteobacteria | Rhodospirillales | Rhodospirillaceae | unclassified | unclassified |
| Otu003298 | 1 | 0.6667 | 0.816 | 0.011 | Bacteria | Proteobacteria | Gammaproteobacteria | HTCC2188 | HTCC2089 | unclassified | unclassified |
| Otu003311 | 1 | 1 | 1 | 0.001 | Bacteria | Acidobacteria | Acidobacteria-6 | iii1-15 | unclassified | unclassified | unclassified |
| Otu003348 | 1 | 0.6667 | 0.816 | 0.008 | Bacteria | Chloroflexi | Anaerolineae | Caldilineales | Caldilineaceae | unclassified | unclassified |
| Otu003357 | 1 | 0.6667 | 0.816 | 0.008 | Bacteria | Chloroflexi | SAR202 | unclassified | unclassified | unclassified | unclassified |
| Otu003460 | 1 | 0.6667 | 0.816 | 0.011 | Bacteria | Proteobacteria | Deltaproteobacteria | Entotheonellales | Entotheonellaceae | unclassified | unclassified |
| Otu003596 | 1 | 0.6667 | 0.816 | 0.011 | Bacteria | Chloroflexi | SAR202 | unclassified | unclassified | unclassified | unclassified |
| Otu003612 | 1 | 0.6667 | 0.816 | 0.011 | Bacteria | Firmicutes | Bacilli | Lactobacillales | Streptococcaceae | Streptococcus | unclassified |
| Otu003660 | 1 | 1 | 1 | 0.001 | Bacteria | Actinobacteria | Actinobacteria | Actinomycetales | Propionibacteriaceae | Propionibacterium | acnes |
| Otu003729 | 0.8853 | 0.6667 | 0.768 | 0.044 | Bacteria | Chloroflexi | SAR202 | unclassified | unclassified | unclassified | unclassified |
| Otu003834 | 0.9868 | 1 | 0.993 | 0.001 | Bacteria | Proteobacteria | Gammaproteobacteria | unclassified | unclassified | unclassified | unclassified |
| Otu003837 | 1 | 0.6667 | 0.816 | 0.008 | Bacteria | Actinobacteria | Acidimicrobiia | Acidimicrobiales | unclassified | unclassified | unclassified |
| Otu003887 | 1 | 0.6667 | 0.816 | 0.008 | Bacteria | Proteobacteria | Gammaproteobacteria | unclassified | unclassified | unclassified | unclassified |
| Otu003905 | 1 | 1 | 1 | 0.001 | Bacteria | Proteobacteria | unclassified | unclassified | unclassified | unclassified | unclassified |
| Otu003989 | 0.8884 | 0.6667 | 0.77 | 0.019 | Bacteria | Chloroflexi | SAR202 | unclassified | unclassified | unclassified | unclassified |
| Otu004221 | 0.8902 | 0.6667 | 0.77 | 0.023 | Bacteria | Chloroflexi | SAR202 | unclassified | unclassified | unclassified | unclassified |
| Otu004242 | 1 | 0.6667 | 0.816 | 0.011 | Bacteria | unclassified | unclassified | unclassified | unclassified | unclassified | unclassified |
| Otu004379 | 0.8394 | 1 | 0.916 | 0.007 | Bacteria | AncK6 | unclassified | unclassified | unclassified | unclassified | unclassified |
| Otu004529 | 1 | 1 | 1 | 0.001 | Bacteria | Firmicutes | Clostridia | Clostridiales | Clostridiaceae | unclassified | unclassified |
| Otu004657 | 1 | 1 | 1 | 0.001 | Bacteria | Firmicutes | unclassified | unclassified | unclassified | unclassified | unclassified |
| Otu004866 | 1 | 1 | 1 | 0.001 | Bacteria | Actinobacteria | Actinobacteria | Actinomycetales | Corynebacteriaceae | Corynebacterium | unclassified |
| Otu004905 | 1 | 0.6667 | 0.816 | 0.011 | Archaea | Crenarchaeota | Thaumarchaeota | Cenarchaeales | Cenarchaeaceae | Nitrosopumilus | pIVWA5 |
| Otu005011 | 1 | 0.6667 | 0.816 | 0.011 | Bacteria | Firmicutes | Clostridia | Clostridiales | Peptostreptococcaceae | unclassified | unclassified |
| Otu005130 | 0.996 | 1 | 0.998 | 0.001 | Bacteria | Firmicutes | Bacilli | Bacillales | Bacillaceae | unclassified | unclassified |
| Otu005167 | 1 | 0.6667 | 0.816 | 0.011 | Archaea | Crenarchaeota | Thaumarchaeota | Cenarchaeales | Cenarchaeaceae | Nitrosopumilus | pIVWA5 |
| Otu005245 | 1 | 1 | 1 | 0.001 | Bacteria | Chloroflexi | Anaerolineae | Caldilineales | Caldilineaceae | unclassified | unclassified |
| Otu005312 | 1 | 0.6667 | 0.816 | 0.011 | Bacteria | Poribacteria | unclassified | unclassified | unclassified | unclassified | unclassified |
| Otu005351 | 0.9946 | 1 | 0.997 | 0.001 | Bacteria | Firmicutes | Bacilli | Turicibacterales | Turicibacteraceae | Turicibacter | unclassified |
| Otu005353 | 0.89 | 0.6667 | 0.77 | 0.009 | Bacteria | Acidobacteria | Acidobacteria-6 | iii1-15 | unclassified | unclassified | unclassified |
| Otu005460 | 0.952 | 1 | 0.976 | 0.001 | Bacteria | Chloroflexi | SAR202 | unclassified | unclassified | unclassified | unclassified |
| Otu005506 | 1 | 0.6667 | 0.816 | 0.011 | Bacteria | Firmicutes | Bacilli | Bacillales | Bacillaceae | Bacillus | halodurans |
| Otu005669 | 1 | 1 | 1 | 0.001 | Bacteria | Firmicutes | Clostridia | Clostridiales | Peptococcaceae | Desulfosporosinus | meridiei |
| Otu005698 | 0.7191 | 1 | 0.848 | 0.007 | Bacteria | Proteobacteria | Deltaproteobacteria | unclassified | unclassified | unclassified | unclassified |
| Otu005714 | 1 | 0.6667 | 0.816 | 0.008 | Bacteria | Actinobacteria | Acidimicrobiia | Acidimicrobiales | TK06 | unclassified | unclassified |
| Otu005880 | 1 | 0.6667 | 0.816 | 0.008 | Bacteria | Proteobacteria | unclassified | unclassified | unclassified | unclassified | unclassified |
| Otu005930 | 0.9577 | 0.6667 | 0.799 | 0.011 | Bacteria | Actinobacteria | Acidimicrobiia | Acidimicrobiales | koll13 | unclassified | unclassified |
| Otu006023 | 1 | 1 | 1 | 0.001 | Bacteria | Actinobacteria | Actinobacteria | Actinomycetales | Micromonosporaceae | unclassified | unclassified |
| Otu006052 | 0.9917 | 1 | 0.996 | 0.001 | Bacteria | Chloroflexi | Anaerolineae | Caldilineales | Caldilineaceae | unclassified | unclassified |

| | | | | | | | | | | | |
|---|---|---|---|---|---|---|---|---|---|---|---|
| Otu006342 | 1 | 0.6667 | 0.816 | 0.011 | Bacteria | Proteobacteria | Gammaproteobacteria | unclassified | unclassified | unclassified | unclassified |
| Otu006397 | 0.894 | 1 | 0.946 | 0.003 | Bacteria | Proteobacteria | Gammaproteobacteria | HTCC2188 | HTCC2089 | unclassified | unclassified |
| Otu006628 | 0.8883 | 0.6667 | 0.77 | 0.025 | Bacteria | Chloroflexi | SAR202 | unclassified | unclassified | unclassified | unclassified |
| Otu006629 | 1 | 0.6667 | 0.816 | 0.008 | Bacteria | Poribacteria | unclassified | unclassified | unclassified | unclassified | unclassified |
| Otu006700 | 1 | 1 | 1 | 0.001 | Bacteria | PAUC34f | unclassified | unclassified | unclassified | unclassified | unclassified |
| Otu006764 | 1 | 0.6667 | 0.816 | 0.011 | Bacteria | Chloroflexi | TK17 | mle1-48 | unclassified | unclassified | unclassified |
| Otu006766 | 1 | 0.6667 | 0.816 | 0.008 | Bacteria | Chloroflexi | SAR202 | unclassified | unclassified | unclassified | unclassified |
| Otu006843 | 0.9913 | 1 | 0.996 | 0.001 | Bacteria | Proteobacteria | Alphaproteobacteria | unclassified | unclassified | unclassified | unclassified |
| Otu006877 | 1 | 0.6667 | 0.816 | 0.008 | Bacteria | Cyanobacteria | Synechococcophycideae | Synechococcales | Synechococcaceae | Synechococcus | unclassified |
| Otu007053 | 0.6789 | 1 | 0.824 | 0.012 | Archaea | Crenarchaeota | Thaumarchaeota | Cenarchaeales | Cenarchaeaceae | Nitrosopumilus | unclassified |
| Otu007119 | 1 | 0.6667 | 0.816 | 0.011 | Bacteria | Chloroflexi | SAR202 | unclassified | unclassified | unclassified | unclassified |
| Otu007127 | 1 | 0.6667 | 0.816 | 0.011 | Bacteria | PAUC34f | unclassified | unclassified | unclassified | unclassified | unclassified |
| Otu007172 | 0.9328 | 0.6667 | 0.789 | 0.027 | Bacteria | Chloroflexi | Ktedonobacteria | TK10 | unclassified | unclassified | unclassified |
| Otu007216 | 1 | 0.6667 | 0.816 | 0.008 | Bacteria | Chloroflexi | Anaerolineae | SBR1031 | A4b | unclassified | unclassified |
| Otu007346 | 1 | 0.6667 | 0.816 | 0.011 | Bacteria | PAUC34f | unclassified | unclassified | unclassified | unclassified | unclassified |
| Otu007442 | 1 | 0.6667 | 0.816 | 0.011 | Bacteria | Cyanobacteria | Synechococcophycideae | Synechococcales | Synechococcaceae | Synechococcus | unclassified |
| Otu007519 | 1 | 0.6667 | 0.816 | 0.011 | Bacteria | Firmicutes | Clostridia | Clostridiales | Peptococcaceae | Desulfosporosinus | meridiei |
| Otu007702 | 1 | 0.6667 | 0.816 | 0.011 | Bacteria | Proteobacteria | Gammaproteobacteria | unclassified | unclassified | unclassified | unclassified |
| Otu007704 | 1 | 0.6667 | 0.816 | 0.008 | Bacteria | Proteobacteria | Gammaproteobacteria | unclassified | unclassified | unclassified | unclassified |
| Otu007987 | 0.9896 | 1 | 0.995 | 0.001 | Bacteria | Firmicutes | Clostridia | Clostridiales | Clostridiaceae | Clostridium | butyricum |
| Otu008130 | 1 | 0.6667 | 0.816 | 0.011 | Bacteria | Cyanobacteria | Synechococcophycideae | Synechococcales | Synechococcaceae | unclassified | unclassified |
| Otu008215 | 1 | 0.6667 | 0.816 | 0.008 | Bacteria | Chloroflexi | SAR202 | unclassified | unclassified | unclassified | unclassified |
| Otu008383 | 1 | 0.6667 | 0.816 | 0.008 | Bacteria | Proteobacteria | Gammaproteobacteria | unclassified | unclassified | unclassified | unclassified |
| Otu008385 | 1 | 1 | 1 | 0.001 | Bacteria | Chloroflexi | SAR202 | unclassified | unclassified | unclassified | unclassified |
| Otu008823 | 1 | 0.6667 | 0.816 | 0.011 | Bacteria | Proteobacteria | Deltaproteobacteria | Syntrophobacterales | Syntrophobacteraceae | unclassified | unclassified |
| Otu008986 | 1 | 0.6667 | 0.816 | 0.008 | Bacteria | unclassified | unclassified | unclassified | unclassified | unclassified | unclassified |
| Otu009428 | 1 | 0.6667 | 0.816 | 0.011 | Bacteria | Chloroflexi | Anaerolineae | SBR1031 | A4b | unclassified | unclassified |
| Otu010196 | 0.9661 | 0.6667 | 0.803 | 0.008 | Bacteria | PAUC34f | unclassified | unclassified | unclassified | unclassified | unclassified |
| Otu010363 | 1 | 0.6667 | 0.816 | 0.011 | Bacteria | PAUC34f | unclassified | unclassified | unclassified | unclassified | unclassified |
| Otu010775 | 1 | 0.6667 | 0.816 | 0.008 | Bacteria | Proteobacteria | unclassified | unclassified | unclassified | unclassified | unclassified |
| Otu011501 | 1 | 0.6667 | 0.816 | 0.008 | Bacteria | Proteobacteria | Deltaproteobacteria | Entotheonellales | Entotheonellaceae | unclassified | unclassified |
| Otu011505 | 1 | 0.6667 | 0.816 | 0.011 | Bacteria | Chloroflexi | unclassified | unclassified | unclassified | unclassified | unclassified |
| Otu011581 | 1 | 0.6667 | 0.816 | 0.011 | Bacteria | Proteobacteria | Betaproteobacteria | Rhodocyclales | Rhodocyclaceae | unclassified | unclassified |
| Otu011615 | 1 | 0.6667 | 0.816 | 0.011 | Bacteria | Proteobacteria | Gammaproteobacteria | unclassified | unclassified | unclassified | unclassified |
| Otu012154 | 0.9589 | 1 | 0.979 | 0.001 | Bacteria | Proteobacteria | Gammaproteobacteria | unclassified | unclassified | unclassified | unclassified |
| Otu012197 | 1 | 0.6667 | 0.816 | 0.011 | Bacteria | Firmicutes | Clostridia | Clostridiales | Tissierellaceae | Anaerococcus | unclassified |
| Otu012203 | 1 | 1 | 1 | 0.001 | Bacteria | Firmicutes | Clostridia | Clostridiales | Peptococcaceae | Desulfosporosinus | meridiei |
| Otu012318 | 1 | 1 | 1 | 0.001 | Bacteria | Firmicutes | Bacilli | Bacillales | unclassified | unclassified | unclassified |
| Otu012912 | 1 | 0.6667 | 0.816 | 0.008 | Bacteria | Proteobacteria | Gammaproteobacteria | unclassified | unclassified | unclassified | unclassified |
| Otu013023 | 1 | 0.6667 | 0.816 | 0.011 | Bacteria | Nitrospirae | Nitrospira | Nitrospirales | Nitrospiraceae | unclassified | unclassified |
| Otu013171 | 1 | 0.6667 | 0.816 | 0.008 | Bacteria | PAUC34f | unclassified | unclassified | unclassified | unclassified | unclassified |
| Otu013468 | 1 | 1 | 1 | 0.001 | Bacteria | Firmicutes | Clostridia | Clostridiales | Peptococcaceae | Desulfosporosinus | meridiei |
| Otu013675 | 1 | 0.6667 | 0.816 | 0.011 | Bacteria | Actinobacteria | Actinobacteria | Actinomycetales | Micrococcaceae | Micrococcus | luteus |
| Otu014380 | 1 | 0.6667 | 0.816 | 0.011 | Bacteria | Proteobacteria | Gammaproteobacteria | Enterobacteriales | Enterobacteriaceae | unclassified | unclassified |
| Otu014773 | 1 | 0.6667 | 0.816 | 0.011 | Bacteria | Firmicutes | Bacilli | Bacillales | Bacillaceae | Bacillus | unclassified |
| Otu014909 | 1 | 0.6667 | 0.816 | 0.011 | Bacteria | Spirochaetes | Spirochaetes | Spirochaetales | Spirochaetaceae | unclassified | unclassified |
| Otu015008 | 1 | 0.6667 | 0.816 | 0.011 | Bacteria | Proteobacteria | Gammaproteobacteria | Pseudomonadales | Pseudomonadaceae | Pseudomonas | viridiflava |
| Otu015959 | 1 | 1 | 1 | 0.001 | Bacteria | Bacteroidetes | Rhodothermi | Rhodothermales | Rhodothermaceae | unclassified | unclassified |
| Otu016008 | 1 | 0.6667 | 0.816 | 0.008 | Bacteria | Actinobacteria | Acidimicrobiia | Acidimicrobiales | wb1_P06 | unclassified | unclassified |
| Otu016163 | 1 | 0.6667 | 0.816 | 0.011 | Bacteria | Chloroflexi | SAR202 | unclassified | unclassified | unclassified | unclassified |
| Otu016834 | 0.9203 | 0.6667 | 0.783 | 0.013 | Bacteria | Proteobacteria | Gammaproteobacteria | unclassified | unclassified | unclassified | unclassified |
| Otu017215 | 1 | 0.6667 | 0.816 | 0.011 | Bacteria | Proteobacteria | Deltaproteobacteria | Syntrophobacterales | Syntrophobacteraceae | unclassified | unclassified |
| Otu017344 | 1 | 0.6667 | 0.816 | 0.008 | Bacteria | Acidobacteria | Acidobacteria-6 | BPC015 | unclassified | unclassified | unclassified |
| Otu018060 | 1 | 0.6667 | 0.816 | 0.011 | Bacteria | Gemmatimonadetes | Gemm-4 | unclassified | unclassified | unclassified | unclassified |
| Otu018910 | 1 | 0.6667 | 0.816 | 0.011 | Bacteria | unclassified | unclassified | unclassified | unclassified | unclassified | unclassified |
| Otu019053 | 0.9733 | 1 | 0.987 | 0.001 | Bacteria | Proteobacteria | Alphaproteobacteria | Rickettsiales | mitochondria | unclassified | unclassified |
| Otu019058 | 1 | 0.6667 | 0.816 | 0.008 | Bacteria | Acidobacteria | unclassified | unclassified | unclassified | unclassified | unclassified |
| Otu019394 | 1 | 0.6667 | 0.816 | 0.011 | Bacteria | Proteobacteria | Gammaproteobacteria | Enterobacteriales | Enterobacteriaceae | Erwinia | unclassified |
| Otu019444 | 1 | 1 | 1 | 0.001 | Bacteria | Firmicutes | Clostridia | Clostridiales | Peptococcaceae | Desulfosporosinus | meridiei |
| Otu019447 | 1 | 0.6667 | 0.816 | 0.011 | Bacteria | Firmicutes | Clostridia | Clostridiales | Clostridiaceae | unclassified | unclassified |
| Otu019541 | 1 | 0.6667 | 0.816 | 0.008 | Bacteria | Cyanobacteria | Synechococcophycideae | Synechococcales | Synechococcaceae | Synechococcus | unclassified |
| Otu019660 | 0.9894 | 0.6667 | 0.812 | 0.011 | Bacteria | Proteobacteria | Gammaproteobacteria | HTCC2188 | HTCC2089 | unclassified | unclassified |
| Otu019929 | 1 | 0.6667 | 0.816 | 0.008 | Bacteria | Firmicutes | Bacilli | Bacillales | unclassified | unclassified | unclassified |
| Otu020563 | 0.9597 | 0.6667 | 0.8 | 0.008 | Bacteria | SBR1093 | EC214 | unclassified | unclassified | unclassified | unclassified |
| Otu021231 | 1 | 0.6667 | 0.816 | 0.011 | Bacteria | Spirochaetes | Spirochaetes | Spirochaetales | Spirochaetaceae | unclassified | unclassified |
| Otu021346 | 1 | 0.6667 | 0.816 | 0.008 | Bacteria | Acidobacteria | Sva0725 | Sva0725 | unclassified | unclassified | unclassified |
| Otu022185 | 0.8736 | 1 | 0.935 | 0.005 | Bacteria | PAUC34f | unclassified | unclassified | unclassified | unclassified | unclassified |
| Otu022339 | 0.9729 | 0.6667 | 0.805 | 0.011 | Bacteria | Actinobacteria | Actinobacteria | Actinomycetales | Mycobacteriaceae | Mycobacterium | gordonae |
| Otu022442 | 1 | 0.6667 | 0.816 | 0.011 | Bacteria | Firmicutes | Clostridia | Clostridiales | Clostridiaceae | SMB53 | unclassified |
| Otu023436 | 1 | 1 | 1 | 0.001 | Archaea | Crenarchaeota | Thaumarchaeota | Cenarchaeales | Cenarchaeaceae | Nitrosopumilus | unclassified |
| Otu025291 | 1 | 0.6667 | 0.816 | 0.008 | Bacteria | Chloroflexi | SAR202 | unclassified | unclassified | unclassified | unclassified |
| Otu025695 | 1 | 0.6667 | 0.816 | 0.011 | Bacteria | unclassified | unclassified | unclassified | unclassified | unclassified | unclassified |
| Otu026913 | 0.879 | 1 | 0.938 | 0.005 | Bacteria | Chloroflexi | SAR202 | unclassified | unclassified | unclassified | unclassified |
| Otu027239 | 0.9021 | 0.6667 | 0.776 | 0.028 | Bacteria | SBR1093 | EC214 | unclassified | unclassified | unclassified | unclassified |
| Otu027794 | 1 | 0.6667 | 0.816 | 0.008 | Bacteria | Chloroflexi | TK17 | TK18 | unclassified | unclassified | unclassified |
| Otu028315 | 1 | 0.6667 | 0.816 | 0.011 | Bacteria | unclassified | unclassified | unclassified | unclassified | unclassified | unclassified |
| Otu028411 | 1 | 0.6667 | 0.816 | 0.011 | Bacteria | Chloroflexi | Anaerolineae | Caldilineales | Caldilineaceae | unclassified | unclassified |
| Otu029648 | 1 | 1 | 1 | 0.001 | Bacteria | Proteobacteria | Gammaproteobacteria | unclassified | unclassified | unclassified | unclassified |
| Otu029969 | 0.9581 | 0.6667 | 0.799 | 0.011 | Bacteria | PAUC34f | unclassified | unclassified | unclassified | unclassified | unclassified |
| Otu030099 | 0.9906 | 0.6667 | 0.813 | 0.008 | Bacteria | Firmicutes | Bacilli | Bacillales | Bacillaceae | unclassified | unclassified |
| Otu030101 | 0.9919 | 0.6667 | 0.813 | 0.011 | Bacteria | Firmicutes | Bacilli | Bacillales | Thermoactinomycetaceae | Thermoactinomyces | unclassified |
| Otu030105 | 1 | 0.6667 | 0.816 | 0.011 | Bacteria | Firmicutes | Bacilli | Bacillales | Thermoactinomycetaceae | Thermoactinomyces | unclassified |
| Otu030704 | 1 | 0.6667 | 0.816 | 0.008 | Bacteria | Cyanobacteria | Synechococcophycideae | Synechococcales | Synechococcaceae | unclassified | unclassified |
| Otu030915 | 1 | 0.6667 | 0.816 | 0.011 | Bacteria | unclassified | unclassified | unclassified | unclassified | unclassified | unclassified |
| Otu032874 | 1 | 0.6667 | 0.816 | 0.011 | Bacteria | Proteobacteria | Deltaproteobacteria | Entotheonellales | Entotheonellaceae | Candidatus_Entotheonell | unclassified |
| Otu034187 | 1 | 0.6667 | 0.816 | 0.008 | Bacteria | Proteobacteria | Alphaproteobacteria | unclassified | unclassified | unclassified | unclassified |
| Otu034705 | 1 | 0.6667 | 0.816 | 0.011 | Bacteria | Actinobacteria | Acidimicrobiia | Acidimicrobiales | wb1_P06 | unclassified | unclassified |
| Otu035774 | 1 | 0.6667 | 0.816 | 0.011 | Bacteria | Proteobacteria | Gammaproteobacteria | Enterobacteriales | Enterobacteriaceae | Erwinia | soli |
| Otu035842 | 0.9917 | 1 | 0.996 | 0.001 | Bacteria | Proteobacteria | Gammaproteobacteria | Enterobacteriales | Enterobacteriaceae | Erwinia | unclassified |
| Otu035851 | 1 | 1 | 1 | 0.001 | Bacteria | Proteobacteria | Gammaproteobacteria | Enterobacteriales | Enterobacteriaceae | Erwinia | unclassified |
| Otu035854 | 1 | 0.6667 | 0.816 | 0.011 | Bacteria | Proteobacteria | Gammaproteobacteria | Enterobacteriales | Enterobacteriaceae | Erwinia | unclassified |
| Otu035857 | 1 | 0.6667 | 0.816 | 0.011 | Bacteria | Proteobacteria | Gammaproteobacteria | Enterobacteriales | Enterobacteriaceae | Erwinia | unclassified |
| Otu035937 | 0.9333 | 0.6667 | 0.789 | 0.033 | Bacteria | unclassified | unclassified | unclassified | unclassified | unclassified | unclassified |
| Otu036294 | 0.9913 | 1 | 0.996 | 0.001 | Bacteria | Proteobacteria | Alphaproteobacteria | Rhizobiales | Rhizobiaceae | Agrobacterium | vitis |
| Otu037095 | 1 | 0.6667 | 0.816 | 0.011 | Bacteria | Acidobacteria | Acidobacteria-6 | BPC015 | unclassified | unclassified | unclassified |
| Otu038063 | 1 | 0.6667 | 0.816 | 0.008 | Bacteria | Chloroflexi | SAR202 | unclassified | unclassified | unclassified | unclassified |
| Otu038216 | 1 | 0.6667 | 0.816 | 0.008 | Bacteria | Proteobacteria | Gammaproteobacteria | Oceanospirillales | Saccharospirillaceae | ML110J-20 | unclassified |
| Otu042667 | 1 | 1 | 1 | 0.001 | Bacteria | unclassified | unclassified | unclassified | unclassified | unclassified | unclassified |
| Otu047721 | 1 | 0.6667 | 0.816 | 0.011 | Bacteria | unclassified | unclassified | unclassified | unclassified | unclassified | unclassified |
| Otu049645 | 1 | 0.6667 | 0.816 | 0.011 | Bacteria | Poribacteria | unclassified | unclassified | unclassified | unclassified | unclassified |

| | | | | | | | | | | | |
|---|---|---|---|---|---|---|---|---|---|---|---|
| Otu050922 | 1 | 0.6667 | 0.816 | 0.011 | Bacteria | Actinobacteria | Actinobacteria | Actinomycetales | Corynebacteriaceae | Corynebacterium | unclassified |
| Otu051442 | 1 | 1 | 1 | 0.001 | Bacteria | Proteobacteria | Gammaproteobacteria | Enterobacteriales | Enterobacteriaceae | unclassified | unclassified |
| Otu058241 | 1 | 0.6667 | 0.816 | 0.008 | Bacteria | Chloroflexi | SAR202 | unclassified | unclassified | unclassified | unclassified |
| Otu059160 | 0.8237 | 1 | 0.908 | 0.015 | Bacteria | Chloroflexi | SAR202 | unclassified | unclassified | unclassified | unclassified |
| Otu059504 | 1 | 1 | 1 | 0.001 | Bacteria | Proteobacteria | Gammaproteobacteria | unclassified | unclassified | unclassified | unclassified |
| Otu061224 | 1 | 0.6667 | 0.816 | 0.011 | Bacteria | Gemmatimonadetes | Gemm-2 | unclassified | unclassified | unclassified | unclassified |
| Otu063036 | 1 | 0.6667 | 0.816 | 0.011 | Bacteria | Poribacteria | unclassified | unclassified | unclassified | unclassified | unclassified |
| Otu064746 | 1 | 0.6667 | 0.816 | 0.011 | Bacteria | Proteobacteria | Gammaproteobacteria | unclassified | unclassified | unclassified | unclassified |
| Otu065406 | 1 | 0.6667 | 0.816 | 0.011 | Bacteria | Proteobacteria | Gammaproteobacteria | Pseudomonadales | Moraxellaceae | Psychrobacter | unclassified |
| Otu066852 | 1 | 0.6667 | 0.816 | 0.011 | Bacteria | PAUC34f | unclassified | unclassified | unclassified | unclassified | unclassified |
| Otu074553 | 1 | 0.6667 | 0.816 | 0.011 | Bacteria | Actinobacteria | Actinobacteria | Actinomycetales | Dermabacteraceae | Brachybacterium | unclassified |
| Otu078401 | 0.9519 | 0.6667 | 0.797 | 0.008 | Bacteria | Proteobacteria | Deltaproteobacteria | unclassified | unclassified | unclassified | unclassified |
| Otu078602 | 1 | 0.6667 | 0.816 | 0.011 | Bacteria | unclassified | unclassified | unclassified | unclassified | unclassified | unclassified |
| Otu084335 | 0.9819 | 1 | 0.991 | 0.001 | Bacteria | Proteobacteria | Gammaproteobacteria | Enterobacteriales | Enterobacteriaceae | unclassified | unclassified |
| Otu086029 | 1 | 0.6667 | 0.816 | 0.008 | Bacteria | Actinobacteria | Acidimicrobiia | Acidimicrobiales | TK06 | unclassified | unclassified |
| Otu089571 | 1 | 0.6667 | 0.816 | 0.008 | Bacteria | Acidobacteria | Sva0725 | Sva0725 | unclassified | unclassified | unclassified |
| Otu095236 | 0.9666 | 0.6667 | 0.803 | 0.011 | Bacteria | Chloroflexi | SAR202 | unclassified | unclassified | unclassified | unclassified |
| Otu098275 | 1 | 0.6667 | 0.816 | 0.008 | Bacteria | Nitrospirae | Nitrospira | Nitrospirales | Nitrospiraceae | unclassified | unclassified |
| Otu124397 | 1 | 0.6667 | 0.816 | 0.011 | Bacteria | Proteobacteria | Gammaproteobacteria | Enterobacteriales | Enterobacteriaceae | unclassified | unclassified |
| Otu126637 | 1 | 0.6667 | 0.816 | 0.008 | Bacteria | Proteobacteria | Gammaproteobacteria | HTCC2188 | HTCC2089 | unclassified | unclassified |
| Otu127672 | 1 | 0.6667 | 0.816 | 0.008 | Bacteria | Proteobacteria | Gammaproteobacteria | unclassified | unclassified | unclassified | unclassified |
| Otu149996 | 1 | 0.6667 | 0.816 | 0.008 | Bacteria | Proteobacteria | Gammaproteobacteria | Pseudomonadales | Moraxellaceae | Acinetobacter | unclassified |
| Otu150471 | 1 | 0.6667 | 0.816 | 0.008 | Bacteria | Chloroflexi | SAR202 | unclassified | unclassified | unclassified | unclassified |
| Otu230145 | 1 | 0.6667 | 0.816 | 0.011 | Bacteria | Proteobacteria | Alphaproteobacteria | unclassified | unclassified | unclassified | unclassified |

| **0.03 Otus** | ***Halichondria panicea*** | **Rest** | **stat** | ***p value*** | **Domain** | **Phylum** | **Class** | **Order** | **Family** | **Genus** | **Species** |
|---|---|---|---|---|---|---|---|---|---|---|---|
| Otu000057 | 1 | 0.6667 | 0.816 | 0.005 | Bacteria | Proteobacteria | Gammaproteobacteria | unclassified | unclassified | unclassified | unclassified |
| Otu000089 | 0.9952 | 1 | 0.998 | 0.001 | Bacteria | Proteobacteria | Alphaproteobacteria | Rhodobacterales | Rhodobacteraceae | unclassified | unclassified |
| Otu000906 | 1 | 0.6667 | 0.816 | 0.011 | Bacteria | Proteobacteria | Alphaproteobacteria | Rhodobacterales | Rhodobacteraceae | unclassified | unclassified |
| Otu001049 | 0.9239 | 1 | 0.961 | 0.004 | Bacteria | Cyanobacteria | Synechococcophycideae | Synechococcales | Synechococcaceae | unclassified | unclassified |
| Otu001114 | 0.9641 | 1 | 0.982 | 0.001 | Bacteria | Proteobacteria | Alphaproteobacteria | Rhizobiales | unclassified | unclassified | unclassified |
| Otu001187 | 0.9456 | 0.6667 | 0.794 | 0.014 | Bacteria | Cyanobacteria | Synechococcophycideae | Synechococcales | Synechococcaceae | Prochlorococcus | unclassified |
| Otu001339 | 0.9024 | 1 | 0.95 | 0.002 | Bacteria | Cyanobacteria | Synechococcophycideae | Synechococcales | Synechococcaceae | Synechococcus | unclassified |
| Otu001624 | 0.8627 | 1 | 0.929 | 0.009 | Bacteria | Cyanobacteria | Synechococcophycideae | Synechococcales | Synechococcaceae | unclassified | unclassified |
| Otu001655 | 0.9899 | 1 | 0.995 | 0.001 | Bacteria | Proteobacteria | unclassified | unclassified | unclassified | unclassified | unclassified |
| Otu001724 | 0.7459 | 0.6667 | 0.705 | 0.05 | Bacteria | Cyanobacteria | Synechococcophycideae | Synechococcales | Synechococcaceae | unclassified | unclassified |
| Otu001971 | 0.9194 | 1 | 0.959 | 0.004 | Bacteria | Cyanobacteria | Synechococcophycideae | Synechococcales | Synechococcaceae | unclassified | unclassified |
| Otu002012 | 0.8447 | 1 | 0.919 | 0.007 | Bacteria | Cyanobacteria | unclassified | unclassified | unclassified | unclassified | unclassified |
| Otu002055 | 1 | 1 | 1 | 0.001 | Bacteria | Proteobacteria | Gammaproteobacteria | unclassified | unclassified | unclassified | unclassified |
| Otu002259 | 0.8257 | 0.6667 | 0.742 | 0.041 | Bacteria | Cyanobacteria | Synechococcophycideae | Synechococcales | Synechococcaceae | Synechococcus | unclassified |
| Otu002649 | 0.7115 | 1 | 0.844 | 0.026 | Bacteria | Actinobacteria | Actinobacteria | Actinomycetales | unclassified | unclassified | unclassified |
| Otu002696 | 0.8766 | 1 | 0.936 | 0.006 | Bacteria | Cyanobacteria | Synechococcophycideae | Synechococcales | Synechococcaceae | Synechococcus | unclassified |
| Otu002781 | 0.8512 | 0.6667 | 0.753 | 0.029 | Bacteria | Bacteroidetes | Flavobacteriia | Flavobacteriales | Flavobacteriaceae | unclassified | unclassified |
| Otu002906 | 0.8611 | 1 | 0.928 | 0.002 | Bacteria | Proteobacteria | Gammaproteobacteria | Legionellales | Coxiellaceae | Rickettsiella | unclassified |
| Otu002976 | 0.9422 | 1 | 0.971 | 0.003 | Bacteria | Proteobacteria | unclassified | unclassified | unclassified | unclassified | unclassified |
| Otu003331 | 0.813 | 1 | 0.902 | 0.017 | Bacteria | Actinobacteria | Actinobacteria | Actinomycetales | Microbacteriaceae | Candidatus_Aquiluna | rubra |
| Otu003366 | 1 | 0.6667 | 0.816 | 0.011 | Bacteria | Cyanobacteria | Synechococcophycideae | Synechococcales | Synechococcaceae | Synechococcus | unclassified |
| Otu003726 | 1 | 0.6667 | 0.816 | 0.014 | Bacteria | Cyanobacteria | Synechococcophycideae | Synechococcales | Synechococcaceae | unclassified | unclassified |
| Otu003761 | 1 | 1 | 1 | 0.001 | Bacteria | Proteobacteria | Alphaproteobacteria | Rhodobacterales | Rhodobacteraceae | Octadecabacter | unclassified |
| Otu003815 | 0.8966 | 0.6667 | 0.773 | 0.032 | Bacteria | Cyanobacteria | Synechococcophycideae | Synechococcales | Synechococcaceae | Synechococcus | unclassified |
| Otu003885 | 1 | 0.6667 | 0.816 | 0.005 | Bacteria | Proteobacteria | Gammaproteobacteria | unclassified | unclassified | unclassified | unclassified |
| Otu003899 | 0.9844 | 0.6667 | 0.81 | 0.014 | Bacteria | Cyanobacteria | Oscillatoriophycideae | Chroococcales | Cyanobacteriaceae | Crocosphaera | unclassified |
| Otu003911 | 1 | 1 | 1 | 0.001 | Bacteria | Proteobacteria | Gammaproteobacteria | unclassified | unclassified | unclassified | unclassified |
| Otu003980 | 0.8201 | 1 | 0.906 | 0.017 | Bacteria | Cyanobacteria | unclassified | unclassified | unclassified | unclassified | unclassified |
| Otu004008 | 1 | 1 | 1 | 0.001 | Bacteria | Bacteroidetes | Cytophagia | Cytophagales | Flammeovirgaceae | unclassified | unclassified |
| Otu004084 | 0.8301 | 1 | 0.911 | 0.01 | Bacteria | Proteobacteria | Gammaproteobacteria | unclassified | unclassified | unclassified | unclassified |
| Otu004873 | 0.7274 | 1 | 0.853 | 0.033 | Bacteria | Proteobacteria | Alphaproteobacteria | Rickettsiales | Pelagibacteraceae | unclassified | unclassified |
| Otu005054 | 0.8645 | 1 | 0.93 | 0.001 | Bacteria | Actinobacteria | Actinobacteria | Actinomycetales | unclassified | unclassified | unclassified |
| Otu005305 | 0.8461 | 1 | 0.92 | 0.002 | Bacteria | Cyanobacteria | unclassified | unclassified | unclassified | unclassified | unclassified |
| Otu005414 | 0.909 | 1 | 0.953 | 0.002 | Bacteria | unclassified | unclassified | unclassified | unclassified | unclassified | unclassified |
| Otu005461 | 1 | 1 | 1 | 0.001 | Bacteria | unclassified | unclassified | unclassified | unclassified | unclassified | unclassified |
| Otu006662 | 1 | 0.6667 | 0.816 | 0.011 | Bacteria | Proteobacteria | Alphaproteobacteria | Rhodobacterales | Rhodobacteraceae | unclassified | unclassified |
| Otu006795 | 0.643 | 1 | 0.802 | 0.049 | Bacteria | Bacteroidetes | unclassified | unclassified | unclassified | unclassified | unclassified |
| Otu006835 | 0.8453 | 0.6667 | 0.751 | 0.039 | Bacteria | Proteobacteria | Alphaproteobacteria | Rickettsiales | Pelagibacteraceae | unclassified | unclassified |
| Otu007357 | 0.909 | 0.6667 | 0.778 | 0.014 | Bacteria | Proteobacteria | unclassified | unclassified | unclassified | unclassified | unclassified |
| Otu007438 | 0.8893 | 0.6667 | 0.77 | 0.027 | Bacteria | Cyanobacteria | Synechococcophycideae | Synechococcales | Synechococcaceae | unclassified | unclassified |
| Otu007464 | 1 | 0.6667 | 0.816 | 0.014 | Bacteria | Proteobacteria | Gammaproteobacteria | unclassified | unclassified | unclassified | unclassified |
| Otu007465 | 0.8103 | 1 | 0.9 | 0.005 | Bacteria | Proteobacteria | Gammaproteobacteria | Legionellales | Francisellaceae | Francisella | unclassified |
| Otu007471 | 0.9545 | 0.6667 | 0.798 | 0.005 | Bacteria | Cyanobacteria | Synechococcophycideae | Synechococcales | Synechococcaceae | unclassified | unclassified |
| Otu007477 | 0.8889 | 1 | 0.943 | 0.005 | Bacteria | Bacteroidetes | Flavobacteriia | Flavobacteriales | Flavobacteriaceae | unclassified | unclassified |
| Otu007486 | 1 | 0.6667 | 0.816 | 0.011 | Bacteria | Bacteroidetes | Flavobacteriia | Flavobacteriales | Flavobacteriaceae | unclassified | unclassified |
| Otu007489 | 0.767 | 1 | 0.876 | 0.024 | Bacteria | Bacteroidetes | Flavobacteriia | Flavobacteriales | Flavobacteriaceae | unclassified | unclassified |
| Otu007581 | 1 | 1 | 1 | 0.001 | Bacteria | Proteobacteria | Alphaproteobacteria | Rhodobacterales | Rhodobacteraceae | unclassified | unclassified |
| Otu007594 | 0.7713 | 1 | 0.878 | 0.01 | Bacteria | Proteobacteria | Alphaproteobacteria | Rhodobacterales | Rhodobacteraceae | unclassified | unclassified |
| Otu007856 | 1 | 1 | 1 | 0.001 | Bacteria | Bacteroidetes | unclassified | unclassified | unclassified | unclassified | unclassified |
| Otu007928 | 0.7535 | 1 | 0.868 | 0.02 | Bacteria | Actinobacteria | unclassified | unclassified | unclassified | unclassified | unclassified |
| Otu008007 | 0.9026 | 1 | 0.95 | 0.001 | Bacteria | Actinobacteria | Actinobacteria | Actinomycetales | unclassified | unclassified | unclassified |
| Otu008008 | 0.7534 | 1 | 0.868 | 0.025 | Bacteria | Actinobacteria | Actinobacteria | unclassified | unclassified | unclassified | unclassified |
| Otu008009 | 1 | 0.6667 | 0.816 | 0.005 | Bacteria | Actinobacteria | Acidimicrobiia | Acidimicrobiales | JdFBGBact | unclassified | unclassified |
| Otu008010 | 0.7971 | 1 | 0.893 | 0.021 | Bacteria | Actinobacteria | Actinobacteria | Actinomycetales | unclassified | unclassified | unclassified |
| Otu008061 | 0.8146 | 1 | 0.903 | 0.02 | Bacteria | Cyanobacteria | Synechococcophycideae | Synechococcales | Synechococcaceae | unclassified | unclassified |
| Otu008312 | 0.9456 | 0.6667 | 0.794 | 0.014 | Bacteria | Planctomycetes | Planctomycetia | Pirellulales | Pirellulaceae | unclassified | unclassified |
| Otu008461 | 1 | 0.6667 | 0.816 | 0.014 | Bacteria | Proteobacteria | Gammaproteobacteria | unclassified | unclassified | unclassified | unclassified |
| Otu009015 | 0.6923 | 1 | 0.832 | 0.041 | Bacteria | Proteobacteria | Alphaproteobacteria | Rhodobacterales | Rhodobacteraceae | unclassified | unclassified |
| Otu009021 | 1 | 1 | 1 | 0.001 | Bacteria | Proteobacteria | Alphaproteobacteria | unclassified | unclassified | unclassified | unclassified |
| Otu009116 | 1 | 0.6667 | 0.816 | 0.014 | Bacteria | Proteobacteria | Gammaproteobacteria | unclassified | unclassified | unclassified | unclassified |
| Otu009321 | 0.8939 | 1 | 0.945 | 0.003 | Bacteria | Proteobacteria | Betaproteobacteria | MWH-UniP1 | unclassified | unclassified | unclassified |
| Otu009458 | 0.8908 | 0.6667 | 0.771 | 0.019 | Bacteria | Bacteroidetes | Flavobacteriia | Flavobacteriales | unclassified | unclassified | unclassified |
| Otu009730 | 0.8921 | 0.6667 | 0.771 | 0.025 | Bacteria | Cyanobacteria | Synechococcophycideae | Synechococcales | Synechococcaceae | Synechococcus | unclassified |
| Otu010020 | 0.9522 | 0.6667 | 0.797 | 0.005 | Bacteria | Proteobacteria | Alphaproteobacteria | Rhodobacterales | Rhodobacteraceae | unclassified | unclassified |
| Otu010024 | 1 | 1 | 1 | 0.001 | Bacteria | Proteobacteria | Alphaproteobacteria | Rhodobacterales | unclassified | unclassified | unclassified |
| Otu010028 | 0.9717 | 0.6667 | 0.805 | 0.011 | Bacteria | Proteobacteria | Alphaproteobacteria | Rhodobacterales | Rhodobacteraceae | unclassified | unclassified |
| Otu010098 | 0.9044 | 1 | 0.951 | 0.006 | Bacteria | Proteobacteria | Alphaproteobacteria | Rickettsiales | Pelagibacteraceae | unclassified | unclassified |
| Otu010132 | 0.8755 | 1 | 0.936 | 0.005 | Bacteria | Verrucomicrobia | Spartobacteria | Chthoniobacterales | Chthoniobacteraceae | Candidatus_Xiphinemato | unclassified |
| Otu010154 | 1 | 1 | 1 | 0.001 | Bacteria | Bacteroidetes | Flavobacteriia | Flavobacteriales | Flavobacteriaceae | unclassified | unclassified |
| Otu010182 | 0.8415 | 1 | 0.917 | 0.005 | Bacteria | Cyanobacteria | Synechococcophycideae | Synechococcales | Synechococcaceae | unclassified | unclassified |
| Otu010186 | 0.9589 | 0.6667 | 0.8 | 0.011 | Bacteria | Chlamydiae | Chlamydiia | Chlamydiales | unclassified | unclassified | unclassified |
| Otu010699 | 0.919 | 1 | 0.959 | 0.003 | Bacteria | Proteobacteria | Gammaproteobacteria | Oceanospirillales | Endozoicimonaceae | unclassified | unclassified |
| Otu010748 | 0.9018 | 1 | 0.95 | 0.004 | Bacteria | Proteobacteria | Alphaproteobacteria | Rhizobiales | unclassified | unclassified | unclassified |
| Otu010810 | 1 | 1 | 1 | 0.001 | Bacteria | Proteobacteria | Alphaproteobacteria | unclassified | unclassified | unclassified | unclassified |
| Otu011338 | 1 | 0.6667 | 0.816 | 0.005 | Bacteria | Proteobacteria | Alphaproteobacteria | Sphingomonadales | unclassified | unclassified | unclassified |

| | | | | | | | | | | | |
|---|---|---|---|---|---|---|---|---|---|---|---|
| Otu011340 | 0.9742 | 0.6667 | 0.806 | 0.005 | Bacteria | Proteobacteria | Alphaproteobacteria | Sphingomonadales | unclassified | unclassified | unclassified |
| Otu011395 | 0.8982 | 1 | 0.948 | 0.005 | Bacteria | Bacteroidetes | Flavobacteriia | Flavobacteriales | Flavobacteriaceae | Flavobacterium | gelidilacus |
| Otu011878 | 0.9916 | 1 | 0.996 | 0.001 | Bacteria | Proteobacteria | Gammaproteobacteria | unclassified | unclassified | unclassified | unclassified |
| Otu012473 | 1 | 1 | 1 | 0.001 | Bacteria | Proteobacteria | Alphaproteobacteria | Rhodobacterales | unclassified | unclassified | unclassified |
| Otu012504 | 1 | 1 | 1 | 0.001 | Bacteria | Proteobacteria | Alphaproteobacteria | Rhodobacterales | Rhodobacteraceae | unclassified | unclassified |
| Otu012506 | 0.9372 | 0.6667 | 0.79 | 0.026 | Bacteria | Proteobacteria | Alphaproteobacteria | Rhodobacterales | Rhodobacteraceae | Octadecabacter | unclassified |
| Otu012529 | 1 | 1 | 1 | 0.001 | Bacteria | Proteobacteria | Alphaproteobacteria | Rhodobacterales | Rhodobacteraceae | Octadecabacter | unclassified |
| Otu012538 | 1 | 1 | 1 | 0.001 | Bacteria | Proteobacteria | Alphaproteobacteria | Rhodobacterales | Rhodobacteraceae | unclassified | unclassified |
| Otu012544 | 1 | 1 | 1 | 0.001 | Bacteria | Proteobacteria | Alphaproteobacteria | Rhodobacterales | unclassified | unclassified | unclassified |
| Otu012545 | 1 | 1 | 1 | 0.001 | Bacteria | Proteobacteria | Alphaproteobacteria | Rhodobacterales | unclassified | unclassified | unclassified |
| Otu012546 | 1 | 1 | 1 | 0.001 | Bacteria | Proteobacteria | Alphaproteobacteria | Rhodobacterales | unclassified | unclassified | unclassified |
| Otu012690 | 0.7492 | 1 | 0.866 | 0.028 | Bacteria | Planctomycetes | Planctomycetia | Pirellulales | Pirellulaceae | unclassified | unclassified |
| Otu013076 | 0.7732 | 1 | 0.879 | 0.019 | Bacteria | Verrucomicrobia | Verrucomicrobiae | Verrucomicrobiales | Verrucomicrobiaceae | Verrucomicrobium | unclassified |
| Otu013178 | 1 | 0.6667 | 0.816 | 0.011 | Bacteria | Verrucomicrobia | Verrucomicrobiae | Verrucomicrobiales | Verrucomicrobiaceae | unclassified | unclassified |
| Otu013410 | 0.9457 | 0.6667 | 0.794 | 0.005 | Bacteria | Cyanobacteria | Synechococcophycideae | Synechococcales | Synechococcaceae | Synechococcus | unclassified |
| Otu013529 | 0.8056 | 0.6667 | 0.733 | 0.05 | Bacteria | Proteobacteria | Gammaproteobacteria | Vibrionales | Vibrionaceae | unclassified | unclassified |
| Otu013903 | 0.8536 | 1 | 0.924 | 0.007 | Bacteria | Planctomycetes | Planctomycetia | Pirellulales | Pirellulaceae | unclassified | unclassified |
| Otu014237 | 1 | 1 | 1 | 0.001 | Bacteria | Proteobacteria | Gammaproteobacteria | Oceanospirillales | HOC21 | unclassified | unclassified |
| Otu014870 | 0.8429 | 1 | 0.918 | 0.012 | Bacteria | Bacteroidetes | Flavobacteriia | Flavobacteriales | Flavobacteriaceae | unclassified | unclassified |
| Otu014931 | 0.9445 | 1 | 0.972 | 0.003 | Bacteria | Cyanobacteria | Synechococcophycideae | Synechococcales | Synechococcaceae | unclassified | unclassified |
| Otu015046 | 0.897 | 1 | 0.947 | 0.002 | Bacteria | Actinobacteria | Thermoleophilia | unclassified | unclassified | unclassified | unclassified |
| Otu015359 | 1 | 1 | 1 | 0.001 | Bacteria | Proteobacteria | Alphaproteobacteria | Rhodobacterales | unclassified | unclassified | unclassified |
| Otu015575 | 0.9884 | 1 | 0.994 | 0.001 | Bacteria | Proteobacteria | Alphaproteobacteria | Rhodobacterales | unclassified | unclassified | unclassified |
| Otu015666 | 0.8841 | 1 | 0.94 | 0.006 | Bacteria | unclassified | unclassified | unclassified | unclassified | unclassified | unclassified |
| Otu016397 | 0.7474 | 1 | 0.865 | 0.029 | Bacteria | Planctomycetes | Planctomycetia | Pirellulales | Pirellulaceae | unclassified | unclassified |
| Otu016473 | 1 | 1 | 1 | 0.001 | Bacteria | Proteobacteria | Alphaproteobacteria | Rhodobacterales | Rhodobacteraceae | unclassified | unclassified |
| Otu016720 | 1 | 0.6667 | 0.816 | 0.011 | Bacteria | Proteobacteria | Alphaproteobacteria | Rhodobacterales | unclassified | unclassified | unclassified |
| Otu017487 | 0.8742 | 0.6667 | 0.763 | 0.046 | Bacteria | Bacteroidetes | Flavobacteriia | Flavobacteriales | Flavobacteriaceae | Bizionia | unclassified |
| Otu017616 | 0.8556 | 0.6667 | 0.755 | 0.047 | Bacteria | Bacteroidetes | Flavobacteriia | Flavobacteriales | Flavobacteriaceae | unclassified | unclassified |
| Otu017997 | 0.8657 | 1 | 0.93 | 0.006 | Bacteria | Proteobacteria | Gammaproteobacteria | Thiotrichales | Thiotrichaceae | CF-26 | unclassified |
| Otu018022 | 0.9415 | 0.6667 | 0.792 | 0.014 | Bacteria | Cyanobacteria | Synechococcophycideae | Synechococcales | Synechococcaceae | Prochlorococcus | unclassified |
| Otu018556 | 0.8271 | 0.6667 | 0.743 | 0.037 | Bacteria | Proteobacteria | Alphaproteobacteria | Rhodobacterales | Rhodobacteraceae | unclassified | unclassified |
| Otu018635 | 1 | 1 | 1 | 0.001 | Bacteria | Proteobacteria | Alphaproteobacteria | Rhodobacterales | Rhodobacteraceae | unclassified | unclassified |
| Otu018637 | 0.9914 | 1 | 0.996 | 0.001 | Bacteria | Proteobacteria | Alphaproteobacteria | Rhodobacterales | Rhodobacteraceae | unclassified | unclassified |
| Otu018666 | 1 | 1 | 1 | 0.001 | Bacteria | Proteobacteria | Alphaproteobacteria | Rhodobacterales | unclassified | unclassified | unclassified |
| Otu018669 | 1 | 1 | 1 | 0.001 | Bacteria | Proteobacteria | Alphaproteobacteria | Rhodobacterales | Rhodobacteraceae | Octadecabacter | unclassified |
| Otu018701 | 0.9873 | 1 | 0.994 | 0.001 | Bacteria | Proteobacteria | Alphaproteobacteria | Rhodobacterales | unclassified | unclassified | unclassified |
| Otu019043 | 0.9414 | 1 | 0.97 | 0.004 | Bacteria | Proteobacteria | Gammaproteobacteria | unclassified | unclassified | unclassified | unclassified |
| Otu019406 | 0.9456 | 0.6667 | 0.794 | 0.014 | Bacteria | unclassified | unclassified | unclassified | unclassified | unclassified | unclassified |
| Otu019417 | 0.7482 | 1 | 0.865 | 0.026 | Bacteria | Proteobacteria | Gammaproteobacteria | Vibrionales | Vibrionaceae | Enterovibrio | unclassified |
| Otu019701 | 0.6717 | 1 | 0.82 | 0.012 | Bacteria | Proteobacteria | Gammaproteobacteria | Alteromonadales | HTCC2188 | HTCC | unclassified |
| Otu020054 | 1 | 1 | 1 | 0.001 | Bacteria | Proteobacteria | Alphaproteobacteria | Rhodobacterales | unclassified | unclassified | unclassified |
| Otu020062 | 1 | 1 | 1 | 0.001 | Bacteria | Proteobacteria | Alphaproteobacteria | unclassified | unclassified | unclassified | unclassified |
| Otu020069 | 1 | 1 | 1 | 0.001 | Bacteria | Proteobacteria | Alphaproteobacteria | Rhodobacterales | Rhodobacteraceae | unclassified | unclassified |
| Otu020222 | 1 | 1 | 1 | 0.001 | Bacteria | Proteobacteria | Alphaproteobacteria | Rhodobacterales | Rhodobacteraceae | unclassified | unclassified |
| Otu020294 | 1 | 1 | 1 | 0.001 | Bacteria | Proteobacteria | Alphaproteobacteria | Rhodobacterales | Rhodobacteraceae | unclassified | unclassified |
| Otu020299 | 1 | 1 | 1 | 0.001 | Bacteria | Proteobacteria | Alphaproteobacteria | Rhodobacterales | Rhodobacteraceae | unclassified | unclassified |
| Otu020340 | 0.7703 | 1 | 0.878 | 0.01 | Bacteria | Proteobacteria | Alphaproteobacteria | Rhodobacterales | Rhodobacteraceae | unclassified | unclassified |
| Otu020349 | 1 | 1 | 1 | 0.001 | Bacteria | Proteobacteria | Alphaproteobacteria | Rhodobacterales | unclassified | unclassified | unclassified |
| Otu020357 | 1 | 1 | 1 | 0.001 | Bacteria | Proteobacteria | Alphaproteobacteria | Rhodobacterales | Rhodobacteraceae | unclassified | unclassified |
| Otu020379 | 1 | 1 | 1 | 0.001 | Bacteria | Proteobacteria | Alphaproteobacteria | Rhodobacterales | unclassified | unclassified | unclassified |
| Otu020382 | 1 | 1 | 1 | 0.001 | Bacteria | Proteobacteria | Alphaproteobacteria | Rhodobacterales | Rhodobacteraceae | unclassified | unclassified |
| Otu021398 | 1 | 0.6667 | 0.816 | 0.011 | Bacteria | Proteobacteria | Gammaproteobacteria | Vibrionales | Vibrionaceae | Vibrio | unclassified |
| Otu021420 | 0.7971 | 1 | 0.893 | 0.012 | Bacteria | Actinobacteria | Acidimicrobiia | Acidimicrobiales | Iamiaceae | Iamia | unclassified |
| Otu022004 | 0.9483 | 1 | 0.974 | 0.002 | Bacteria | Cyanobacteria | Synechococcophycideae | Synechococcales | Synechococcaceae | Synechococcus | unclassified |
| Otu022092 | 1 | 0.6667 | 0.816 | 0.005 | Bacteria | Bacteroidetes | Flavobacteriia | Flavobacteriales | Flavobacteriaceae | Kordia | unclassified |
| Otu022114 | 0.8892 | 1 | 0.943 | 0.004 | Bacteria | Bacteroidetes | Flavobacteriia | Flavobacteriales | Flavobacteriaceae | Bizionia | unclassified |
| Otu022247 | 0.9447 | 0.6667 | 0.794 | 0.011 | Bacteria | Cyanobacteria | Synechococcophycideae | Synechococcales | Synechococcaceae | Synechococcus | unclassified |
| Otu022335 | 0.7431 | 1 | 0.862 | 0.033 | Bacteria | Actinobacteria | Actinobacteria | Actinomycetales | unclassified | unclassified | unclassified |
| Otu022340 | 0.7727 | 1 | 0.879 | 0.024 | Bacteria | Actinobacteria | Actinobacteria | Actinomycetales | unclassified | unclassified | unclassified |
| Otu022342 | 0.8029 | 1 | 0.896 | 0.017 | Bacteria | Actinobacteria | Actinobacteria | Actinomycetales | unclassified | unclassified | unclassified |
| Otu022349 | 0.7601 | 1 | 0.872 | 0.02 | Bacteria | unclassified | unclassified | unclassified | unclassified | unclassified | unclassified |
| Otu022548 | 0.8531 | 0.6667 | 0.754 | 0.048 | Bacteria | Cyanobacteria | Synechococcophycideae | Synechococcales | Synechococcaceae | Synechococcus | unclassified |
| Otu022578 | 0.9655 | 1 | 0.983 | 0.001 | Bacteria | Cyanobacteria | Synechococcophycideae | Synechococcales | Synechococcaceae | Synechococcus | unclassified |
| Otu022590 | 1 | 1 | 1 | 0.001 | Bacteria | Proteobacteria | Gammaproteobacteria | Legionellales | Francisellaceae | Francisella | unclassified |
| Otu022599 | 0.7144 | 1 | 0.845 | 0.047 | Bacteria | Cyanobacteria | Synechococcophycideae | Synechococcales | Synechococcaceae | Synechococcus | unclassified |
| Otu023097 | 0.9414 | 0.6667 | 0.792 | 0.022 | Bacteria | Proteobacteria | Gammaproteobacteria | unclassified | unclassified | unclassified | unclassified |
| Otu024568 | 1 | 0.6667 | 0.816 | 0.014 | Bacteria | Proteobacteria | Gammaproteobacteria | unclassified | unclassified | unclassified | unclassified |
| Otu028777 | 1 | 1 | 1 | 0.001 | Bacteria | Proteobacteria | Gammaproteobacteria | unclassified | unclassified | unclassified | unclassified |
| Otu029496 | 0.9731 | 0.6667 | 0.805 | 0.011 | Bacteria | Proteobacteria | Gammaproteobacteria | Thiotrichales | unclassified | unclassified | unclassified |
| Otu029742 | 1 | 0.6667 | 0.816 | 0.005 | Bacteria | Bacteroidetes | Flavobacteriia | Flavobacteriales | Flavobacteriaceae | unclassified | unclassified |
| Otu030199 | 1 | 0.6667 | 0.816 | 0.014 | Bacteria | Proteobacteria | Gammaproteobacteria | unclassified | unclassified | unclassified | unclassified |
| Otu031052 | 0.9139 | 0.6667 | 0.781 | 0.031 | Bacteria | Proteobacteria | Alphaproteobacteria | Rickettsiales | Pelagibacteraceae | unclassified | unclassified |
| Otu031090 | 0.7937 | 1 | 0.891 | 0.01 | Bacteria | Bacteroidetes | unclassified | unclassified | unclassified | unclassified | unclassified |
| Otu032333 | 0.8594 | 1 | 0.927 | 0.006 | Bacteria | Cyanobacteria | Synechococcophycideae | Synechococcales | Synechococcaceae | unclassified | unclassified |
| Otu032338 | 0.8157 | 1 | 0.903 | 0.015 | Bacteria | Cyanobacteria | Synechococcophycideae | Synechococcales | Synechococcaceae | Synechococcus | unclassified |
| Otu032492 | 0.7713 | 1 | 0.878 | 0.012 | Bacteria | Cyanobacteria | Synechococcophycideae | Synechococcales | Synechococcaceae | unclassified | unclassified |
| Otu032507 | 0.8297 | 1 | 0.911 | 0.007 | Bacteria | Cyanobacteria | Synechococcophycideae | Synechococcales | Synechococcaceae | Synechococcus | unclassified |
| Otu032518 | 0.8149 | 0.6667 | 0.737 | 0.041 | Bacteria | Cyanobacteria | Synechococcophycideae | Synechococcales | Synechococcaceae | Synechococcus | unclassified |
| Otu032779 | 1 | 0.6667 | 0.816 | 0.011 | Bacteria | Planctomycetes | Planctomycetia | Pirellulales | Pirellulaceae | unclassified | unclassified |
| Otu033793 | 1 | 1 | 1 | 0.001 | Bacteria | Proteobacteria | Alphaproteobacteria | Rhodobacterales | unclassified | unclassified | unclassified |
| Otu033805 | 1 | 1 | 1 | 0.001 | Bacteria | Proteobacteria | Alphaproteobacteria | Rhodobacterales | unclassified | unclassified | unclassified |
| Otu033806 | 1 | 1 | 1 | 0.001 | Bacteria | Proteobacteria | Alphaproteobacteria | Rhodobacterales | unclassified | unclassified | unclassified |
| Otu033818 | 1 | 1 | 1 | 0.001 | Bacteria | Proteobacteria | Alphaproteobacteria | Rhodobacterales | unclassified | unclassified | unclassified |
| Otu033907 | 1 | 1 | 1 | 0.001 | Bacteria | Proteobacteria | Alphaproteobacteria | Rhodobacterales | Rhodobacteraceae | unclassified | unclassified |
| Otu033941 | 1 | 1 | 1 | 0.001 | Bacteria | Proteobacteria | Alphaproteobacteria | Rhodobacterales | Rhodobacteraceae | unclassified | unclassified |
| Otu033957 | 1 | 1 | 1 | 0.001 | Bacteria | Proteobacteria | Alphaproteobacteria | Rhodobacterales | unclassified | unclassified | unclassified |
| Otu033963 | 1 | 1 | 1 | 0.001 | Bacteria | Proteobacteria | Alphaproteobacteria | Rhodobacterales | unclassified | unclassified | unclassified |
| Otu033972 | 1 | 1 | 1 | 0.001 | Bacteria | Proteobacteria | Alphaproteobacteria | Rhodobacterales | Rhodobacteraceae | unclassified | unclassified |
| Otu034001 | 1 | 1 | 1 | 0.001 | Bacteria | Proteobacteria | Alphaproteobacteria | Rhodobacterales | unclassified | unclassified | unclassified |
| Otu034005 | 1 | 1 | 1 | 0.001 | Bacteria | Proteobacteria | Alphaproteobacteria | Rhodobacterales | unclassified | unclassified | unclassified |
| Otu034117 | 0.8022 | 1 | 0.896 | 0.01 | Bacteria | unclassified | unclassified | unclassified | unclassified | unclassified | unclassified |
| Otu034811 | 1 | 1 | 1 | 0.001 | Bacteria | Proteobacteria | Alphaproteobacteria | Rhodobacterales | Rhodobacteraceae | unclassified | unclassified |
| Otu036876 | 0.9674 | 0.6667 | 0.803 | 0.005 | Bacteria | Proteobacteria | Gammaproteobacteria | unclassified | unclassified | unclassified | unclassified |
| Otu037505 | 1 | 0.6667 | 0.816 | 0.011 | Bacteria | Actinobacteria | Acidimicrobiia | Acidimicrobiales | wb1_P06 | unclassified | unclassified |
| Otu037801 | 0.9634 | 0.6667 | 0.801 | 0.011 | Bacteria | Actinobacteria | Acidimicrobiia | Acidimicrobiales | JdFBGBact | unclassified | unclassified |
| Otu038607 | 0.8477 | 1 | 0.921 | 0.01 | Bacteria | Bacteroidetes | Flavobacteriia | Flavobacteriales | Flavobacteriaceae | unclassified | unclassified |
| Otu038757 | 0.9724 | 0.6667 | 0.805 | 0.005 | Bacteria | Bacteroidetes | Flavobacteriia | Flavobacteriales | Flavobacteriaceae | unclassified | unclassified |
| Otu039785 | 0.9343 | 0.6667 | 0.789 | 0.012 | Bacteria | Bacteroidetes | Flavobacteriia | Flavobacteriales | Flavobacteriaceae | unclassified | unclassified |
| Otu040705 | 0.942 | 0.6667 | 0.792 | 0.011 | Bacteria | Planctomycetes | Planctomycetia | Pirellulales | Pirellulaceae | unclassified | unclassified |

| | | | | | | | | | | | |
|---|---|---|---|---|---|---|---|---|---|---|---|
| Otu040872 | 1 | 0.6667 | 0.816 | 0.014 | Bacteria | Planctomycetes | Planctomycetia | Pirellulales | Pirellulaceae | unclassified | unclassified |
| Otu040885 | 1 | 0.6667 | 0.816 | 0.011 | Bacteria | Bacteroidetes | Flavobacteriia | Flavobacteriales | unclassified | unclassified | unclassified |
| Otu041217 | 0.8091 | 0.6667 | 0.734 | 0.047 | Bacteria | Planctomycetes | Planctomycetia | Pirellulales | Pirellulaceae | unclassified | unclassified |
| Otu042402 | 0.8687 | 0.6667 | 0.761 | 0.035 | Bacteria | Bacteroidetes | Flavobacteriia | Flavobacteriales | Flavobacteriaceae | unclassified | unclassified |
| Otu046234 | 0.9832 | 1 | 0.992 | 0.001 | Bacteria | unclassified | unclassified | unclassified | unclassified | unclassified | unclassified |
| Otu046713 | 0.9101 | 0.6667 | 0.779 | 0.014 | Bacteria | Bacteroidetes | BME43 | unclassified | unclassified | unclassified | unclassified |
| Otu047692 | 0.861 | 1 | 0.928 | 0.005 | Bacteria | Proteobacteria | Betaproteobacteria | unclassified | unclassified | unclassified | unclassified |
| Otu048512 | 0.6676 | 1 | 0.817 | 0.039 | Bacteria | Proteobacteria | Betaproteobacteria | Methylophilales | Methylophilaceae | unclassified | unclassified |
| Otu050063 | 0.857 | 0.6667 | 0.756 | 0.035 | Bacteria | Proteobacteria | Gammaproteobacteria | Alteromonadales | unclassified | unclassified | unclassified |
| Otu050301 | 1 | 0.6667 | 0.816 | 0.014 | Bacteria | Proteobacteria | Gammaproteobacteria | unclassified | unclassified | unclassified | unclassified |
| Otu050879 | 0.9588 | 1 | 0.979 | 0.003 | Bacteria | Actinobacteria | Actinobacteria | Actinomycetales | unclassified | unclassified | unclassified |
| Otu050980 | 0.8697 | 0.6667 | 0.761 | 0.021 | Bacteria | Actinobacteria | Actinobacteria | Actinomycetales | Microbacteriaceae | Candidatus_Rhodoluna | unclassified |
| Otu054295 | 1 | 0.6667 | 0.816 | 0.011 | Bacteria | Proteobacteria | Alphaproteobacteria | Rickettsiales | Pelagibacteraceae | unclassified | unclassified |
| Otu057762 | 0.9421 | 1 | 0.971 | 0.003 | Bacteria | Proteobacteria | Gammaproteobacteria | unclassified | unclassified | unclassified | unclassified |
| Otu061741 | 1 | 1 | 1 | 0.001 | Bacteria | Proteobacteria | Alphaproteobacteria | Rhodobacterales | unclassified | unclassified | unclassified |
| Otu061743 | 1 | 1 | 1 | 0.001 | Bacteria | Proteobacteria | Alphaproteobacteria | Rhodobacterales | unclassified | unclassified | unclassified |
| Otu061755 | 1 | 1 | 1 | 0.001 | Bacteria | Proteobacteria | Alphaproteobacteria | unclassified | unclassified | unclassified | unclassified |
| Otu061781 | 1 | 1 | 1 | 0.001 | Bacteria | Proteobacteria | Alphaproteobacteria | Rhodobacterales | Rhodobacteraceae | unclassified | unclassified |
| Otu061785 | 0.9574 | 0.6667 | 0.799 | 0.005 | Bacteria | Proteobacteria | Alphaproteobacteria | Rhodobacterales | Rhodobacteraceae | unclassified | unclassified |
| Otu061807 | 1 | 1 | 1 | 0.001 | Bacteria | Proteobacteria | Alphaproteobacteria | Rhodobacterales | unclassified | unclassified | unclassified |
| Otu061808 | 1 | 1 | 1 | 0.001 | Bacteria | Proteobacteria | Alphaproteobacteria | Rhodobacterales | Rhodobacteraceae | unclassified | unclassified |
| Otu061819 | 1 | 1 | 1 | 0.001 | Bacteria | Proteobacteria | Alphaproteobacteria | Rhodobacterales | Rhodobacteraceae | unclassified | unclassified |
| Otu061822 | 1 | 1 | 1 | 0.001 | Bacteria | Proteobacteria | Alphaproteobacteria | Rhodobacterales | unclassified | unclassified | unclassified |
| Otu061970 | 1 | 1 | 1 | 0.001 | Bacteria | Proteobacteria | Alphaproteobacteria | Rhodobacterales | Rhodobacteraceae | unclassified | unclassified |
| Otu061980 | 1 | 1 | 1 | 0.001 | Bacteria | Proteobacteria | Alphaproteobacteria | Rhodobacterales | Rhodobacteraceae | unclassified | unclassified |
| Otu061985 | 1 | 1 | 1 | 0.001 | Bacteria | Proteobacteria | Alphaproteobacteria | Rhodobacterales | Rhodobacteraceae | unclassified | unclassified |
| Otu062090 | 1 | 1 | 1 | 0.001 | Bacteria | Proteobacteria | Alphaproteobacteria | unclassified | unclassified | unclassified | unclassified |
| Otu062112 | 1 | 1 | 1 | 0.001 | Bacteria | Proteobacteria | Alphaproteobacteria | Rhodobacterales | Rhodobacteraceae | unclassified | unclassified |
| Otu062122 | 1 | 1 | 1 | 0.001 | Bacteria | Proteobacteria | Alphaproteobacteria | Rhodobacterales | Rhodobacteraceae | Octadecabacter | unclassified |
| Otu062217 | 1 | 1 | 1 | 0.001 | Bacteria | Proteobacteria | Alphaproteobacteria | Rhodobacterales | unclassified | unclassified | unclassified |
| Otu062375 | 1 | 0.6667 | 0.816 | 0.005 | Bacteria | Proteobacteria | Deltaproteobacteria | Bdellovibrionales | Bacteriovoracaceae | Bacteriovorax | unclassified |
| Otu062675 | 1 | 1 | 1 | 0.001 | Bacteria | Proteobacteria | Alphaproteobacteria | Rhodobacterales | unclassified | unclassified | unclassified |
| Otu064934 | 1 | 0.6667 | 0.816 | 0.011 | Bacteria | Proteobacteria | Gammaproteobacteria | Legionellales | Coxiellaceae | Rickettsiella | unclassified |
| Otu065087 | 0.9447 | 0.6667 | 0.794 | 0.011 | Bacteria | Cyanobacteria | Synechococcophycideae | Synechococcales | Synechococcaceae | Synechococcus | unclassified |
| Otu065251 | 0.8729 | 0.6667 | 0.763 | 0.037 | Bacteria | Cyanobacteria | Synechococcophycideae | Synechococcales | Synechococcaceae | unclassified | unclassified |
| Otu065256 | 0.9035 | 1 | 0.951 | 0.006 | Bacteria | Cyanobacteria | Synechococcophycideae | Synechococcales | Synechococcaceae | Synechococcus | unclassified |
| Otu065269 | 0.8222 | 1 | 0.907 | 0.011 | Bacteria | Cyanobacteria | Synechococcophycideae | Synechococcales | Synechococcaceae | unclassified | unclassified |
| Otu065270 | 0.9116 | 0.6667 | 0.78 | 0.023 | Bacteria | Cyanobacteria | Synechococcophycideae | Synechococcales | Synechococcaceae | Synechococcus | unclassified |
| Otu065295 | 0.7655 | 1 | 0.875 | 0.02 | Bacteria | Cyanobacteria | Synechococcophycideae | Synechococcales | Synechococcaceae | unclassified | unclassified |
| Otu065324 | 0.8681 | 0.6667 | 0.761 | 0.023 | Bacteria | Cyanobacteria | Synechococcophycideae | Synechococcales | Synechococcaceae | unclassified | unclassified |
| Otu065463 | 0.8805 | 0.6667 | 0.766 | 0.026 | Bacteria | Cyanobacteria | Synechococcophycideae | Synechococcales | Synechococcaceae | Synechococcus | unclassified |
| Otu065466 | 0.7462 | 1 | 0.864 | 0.034 | Bacteria | Cyanobacteria | Synechococcophycideae | Synechococcales | Synechococcaceae | Synechococcus | unclassified |
| Otu065485 | 0.7833 | 1 | 0.885 | 0.016 | Bacteria | Cyanobacteria | Synechococcophycideae | Synechococcales | Synechococcaceae | unclassified | unclassified |
| Otu065491 | 0.7905 | 1 | 0.889 | 0.017 | Bacteria | Cyanobacteria | Synechococcophycideae | Synechococcales | Synechococcaceae | unclassified | unclassified |
| Otu065711 | 0.751 | 1 | 0.867 | 0.023 | Bacteria | Cyanobacteria | Synechococcophycideae | Synechococcales | Synechococcaceae | Synechococcus | unclassified |
| Otu065753 | 0.7848 | 0.6667 | 0.723 | 0.041 | Bacteria | Cyanobacteria | Synechococcophycideae | Synechococcales | Synechococcaceae | Synechococcus | unclassified |
| Otu066075 | 0.9025 | 0.6667 | 0.776 | 0.028 | Bacteria | Proteobacteria | Gammaproteobacteria | unclassified | unclassified | unclassified | unclassified |
| Otu074610 | 0.9367 | 1 | 0.968 | 0.004 | Bacteria | Actinobacteria | Actinobacteria | Actinomycetales | unclassified | unclassified | unclassified |
| Otu075893 | 1 | 1 | 1 | 0.001 | Bacteria | Proteobacteria | Alphaproteobacteria | Rhodobacterales | Rhodobacteraceae | unclassified | unclassified |
| Otu076847 | 1 | 1 | 1 | 0.001 | Bacteria | Proteobacteria | Alphaproteobacteria | Rhodobacterales | Rhodobacteraceae | unclassified | unclassified |
| Otu077031 | 1 | 1 | 1 | 0.001 | Bacteria | Proteobacteria | Alphaproteobacteria | Rhodobacterales | unclassified | unclassified | unclassified |
| Otu077085 | 1 | 1 | 1 | 0.001 | Bacteria | Proteobacteria | Alphaproteobacteria | Rhodobacterales | unclassified | unclassified | unclassified |
| Otu077191 | 1 | 1 | 1 | 0.001 | Bacteria | Proteobacteria | Alphaproteobacteria | Rhodobacterales | unclassified | unclassified | unclassified |
| Otu077193 | 1 | 1 | 1 | 0.001 | Bacteria | Proteobacteria | Alphaproteobacteria | Rhodobacterales | Rhodobacteraceae | unclassified | unclassified |
| Otu077213 | 1 | 1 | 1 | 0.001 | Bacteria | Proteobacteria | Alphaproteobacteria | Rhodobacterales | Rhodobacteraceae | unclassified | unclassified |
| Otu077287 | 1 | 1 | 1 | 0.001 | Bacteria | Proteobacteria | Alphaproteobacteria | Rhodobacterales | Rhodobacteraceae | unclassified | unclassified |
| Otu078921 | 1 | 1 | 1 | 0.001 | Bacteria | Proteobacteria | Alphaproteobacteria | Rhodobacterales | unclassified | unclassified | unclassified |
| Otu078929 | 0.8154 | 1 | 0.903 | 0.012 | Bacteria | Proteobacteria | Alphaproteobacteria | Rhodobacterales | Rhodobacteraceae | unclassified | unclassified |
| Otu080353 | 1 | 0.6667 | 0.816 | 0.011 | Bacteria | Proteobacteria | Alphaproteobacteria | Rhodospirillales | unclassified | unclassified | unclassified |
| Otu081116 | 0.8191 | 1 | 0.905 | 0.01 | Bacteria | Proteobacteria | unclassified | unclassified | unclassified | unclassified | unclassified |
| Otu083441 | 1 | 0.6667 | 0.816 | 0.005 | Bacteria | Proteobacteria | Alphaproteobacteria | Rhodospirillales | Rhodospirillaceae | unclassified | unclassified |
| Otu085558 | 0.9267 | 0.6667 | 0.786 | 0.013 | Bacteria | Proteobacteria | Gammaproteobacteria | Alteromonadales | Shewanellaceae | Shewanella | benthica |
| Otu090207 | 0.9574 | 0.6667 | 0.799 | 0.005 | Bacteria | Gemmatimonadetes | Gemm-2 | unclassified | unclassified | unclassified | unclassified |
| Otu116819 | 1 | 0.6667 | 0.816 | 0.011 | Bacteria | Bacteroidetes | Flavobacteriia | Flavobacteriales | Flavobacteriaceae | Aquimarina | unclassified |
| Otu118220 | 0.9538 | 0.6667 | 0.797 | 0.011 | Bacteria | Bacteroidetes | Flavobacteriia | Flavobacteriales | Flavobacteriaceae | unclassified | unclassified |
| Otu118546 | 0.8752 | 0.6667 | 0.764 | 0.045 | Bacteria | Bacteroidetes | Flavobacteriia | Flavobacteriales | Flavobacteriaceae | Lacinutrix | unclassified |
| Otu118578 | 1 | 0.6667 | 0.816 | 0.005 | Bacteria | Bacteroidetes | Flavobacteriia | Flavobacteriales | Flavobacteriaceae | unclassified | unclassified |
| Otu118697 | 0.9073 | 0.6667 | 0.778 | 0.014 | Bacteria | Cyanobacteria | Synechococcophycideae | Synechococcales | Synechococcaceae | Synechococcus | unclassified |
| Otu118721 | 0.717 | 1 | 0.847 | 0.023 | Bacteria | Cyanobacteria | Synechococcophycideae | Synechococcales | Synechococcaceae | unclassified | unclassified |
| Otu118799 | 0.9507 | 0.6667 | 0.796 | 0.014 | Bacteria | Cyanobacteria | Synechococcophycideae | Synechococcales | Synechococcaceae | unclassified | unclassified |
| Otu118860 | 0.7797 | 1 | 0.883 | 0.013 | Bacteria | Cyanobacteria | Synechococcophycideae | Synechococcales | Synechococcaceae | Synechococcus | unclassified |
| Otu118921 | 0.8021 | 1 | 0.896 | 0.018 | Bacteria | Cyanobacteria | Synechococcophycideae | Synechococcales | Synechococcaceae | unclassified | unclassified |
| Otu119125 | 0.8538 | 1 | 0.924 | 0.01 | Bacteria | Cyanobacteria | Synechococcophycideae | Synechococcales | Synechococcaceae | Synechococcus | unclassified |
| Otu119137 | 0.7555 | 1 | 0.869 | 0.027 | Bacteria | Cyanobacteria | Synechococcophycideae | Synechococcales | Synechococcaceae | Synechococcus | unclassified |
| Otu119267 | 0.9759 | 0.6667 | 0.807 | 0.005 | Bacteria | Bacteroidetes | Flavobacteriia | Flavobacteriales | Flavobacteriaceae | Lutimonas | unclassified |
| Otu119289 | 0.8021 | 0.6667 | 0.731 | 0.048 | Bacteria | Bacteroidetes | Flavobacteriia | Flavobacteriales | Flavobacteriaceae | unclassified | unclassified |
| Otu119600 | 0.8082 | 1 | 0.899 | 0.008 | Bacteria | Bacteroidetes | Flavobacteriia | Flavobacteriales | Cryomorphaceae | Fluviicola | unclassified |
| Otu124474 | 0.9371 | 0.6667 | 0.79 | 0.014 | Bacteria | Proteobacteria | Gammaproteobacteria | unclassified | unclassified | unclassified | unclassified |
| Otu125663 | 0.9428 | 0.6667 | 0.793 | 0.014 | Bacteria | Planctomycetes | Planctomycetia | Pirellulales | Pirellulaceae | unclassified | unclassified |
| Otu125781 | 0.8676 | 0.6667 | 0.761 | 0.023 | Bacteria | Planctomycetes | Planctomycetia | Pirellulales | Pirellulaceae | unclassified | unclassified |
| Otu127166 | 1 | 1 | 1 | 0.001 | Bacteria | Proteobacteria | Gammaproteobacteria | unclassified | unclassified | unclassified | unclassified |
| Otu129230 | 1 | 0.6667 | 0.816 | 0.014 | Bacteria | Proteobacteria | Gammaproteobacteria | unclassified | unclassified | unclassified | unclassified |
| Otu129888 | 0.8429 | 1 | 0.918 | 0.006 | Bacteria | Proteobacteria | Alphaproteobacteria | Rhizobiales | unclassified | unclassified | unclassified |
| Otu130882 | 1 | 1 | 1 | 0.001 | Bacteria | Proteobacteria | Alphaproteobacteria | unclassified | unclassified | unclassified | unclassified |
| Otu132281 | 0.9623 | 0.6667 | 0.801 | 0.011 | Bacteria | Proteobacteria | Alphaproteobacteria | Rhodobacterales | Rhodobacteraceae | unclassified | unclassified |
| Otu132385 | 1 | 1 | 1 | 0.001 | Bacteria | Proteobacteria | Alphaproteobacteria | unclassified | unclassified | unclassified | unclassified |
| Otu133109 | 0.9164 | 0.6667 | 0.782 | 0.026 | Bacteria | Proteobacteria | Gammaproteobacteria | unclassified | unclassified | unclassified | unclassified |
| Otu133300 | 1 | 1 | 1 | 0.001 | Bacteria | Proteobacteria | Alphaproteobacteria | Rhodobacterales | unclassified | unclassified | unclassified |
| Otu133390 | 1 | 1 | 1 | 0.001 | Bacteria | Proteobacteria | Alphaproteobacteria | Rhodobacterales | Rhodobacteraceae | unclassified | unclassified |
| Otu133739 | 1 | 1 | 1 | 0.001 | Bacteria | Proteobacteria | Alphaproteobacteria | Rhodobacterales | unclassified | unclassified | unclassified |
| Otu133956 | 0.9199 | 1 | 0.959 | 0.002 | Bacteria | Proteobacteria | Alphaproteobacteria | Rhodobacterales | Rhodobacteraceae | Octadecabacter | unclassified |
| Otu133965 | 1 | 1 | 1 | 0.001 | Bacteria | Proteobacteria | Alphaproteobacteria | Rhodobacterales | unclassified | unclassified | unclassified |
| Otu133995 | 1 | 1 | 1 | 0.001 | Bacteria | Proteobacteria | Alphaproteobacteria | Rhodobacterales | Rhodobacteraceae | unclassified | unclassified |
| Otu134010 | 1 | 1 | 1 | 0.001 | Bacteria | Proteobacteria | Alphaproteobacteria | Rhodobacterales | unclassified | unclassified | unclassified |
| Otu134025 | 1 | 1 | 1 | 0.001 | Bacteria | Proteobacteria | Alphaproteobacteria | unclassified | unclassified | unclassified | unclassified |
| Otu134126 | 1 | 1 | 1 | 0.001 | Bacteria | Proteobacteria | Alphaproteobacteria | Rhodobacterales | Rhodobacteraceae | unclassified | unclassified |
| Otu138929 | 0.9415 | 0.6667 | 0.792 | 0.014 | Bacteria | Proteobacteria | Alphaproteobacteria | Sphingomonadales | Erythrobacteraceae | Erythrobacter | longus |
| Otu148755 | 1 | 1 | 1 | 0.001 | Bacteria | Proteobacteria | Alphaproteobacteria | Rhodobacterales | Rhodobacteraceae | unclassified | unclassified |
| Otu148788 | 1 | 1 | 1 | 0.001 | Bacteria | Proteobacteria | Alphaproteobacteria | Rhodobacterales | unclassified | unclassified | unclassified |
| Otu148824 | 1 | 1 | 1 | 0.001 | Bacteria | Proteobacteria | Alphaproteobacteria | Rhodobacterales | unclassified | unclassified | unclassified |

| | | | | | | | | | | | |
|---|---|---|---|---|---|---|---|---|---|---|---|
| Otu148837 | 1 | 1 | 1 | 0.001 | Bacteria | Proteobacteria | Alphaproteobacteria | Rhodobacterales | unclassified | unclassified | unclassified |
| Otu150962 | 0.8251 | 0.6667 | 0.742 | 0.036 | Bacteria | Cyanobacteria | Synechococcophycideae | Synechococcales | Synechococcaceae | Synechococcus | unclassified |
| Otu159004 | 1 | 1 | 1 | 0.001 | Bacteria | Proteobacteria | Alphaproteobacteria | Rhodobacterales | Rhodobacteraceae | unclassified | unclassified |
| Otu159759 | 0.9538 | 0.6667 | 0.797 | 0.011 | Bacteria | Proteobacteria | Alphaproteobacteria | Rhodobacterales | Rhodobacteraceae | unclassified | unclassified |
| Otu164042 | 1 | 1 | 1 | 0.001 | Bacteria | Proteobacteria | Alphaproteobacteria | unclassified | unclassified | unclassified | unclassified |
| Otu175966 | 0.6845 | 1 | 0.827 | 0.03 | Bacteria | Cyanobacteria | Synechococcophycideae | Synechococcales | Synechococcaceae | Paulinella | unclassified |
| Otu176233 | 0.9059 | 0.6667 | 0.777 | 0.022 | Bacteria | Cyanobacteria | Synechococcophycideae | Synechococcales | Synechococcaceae | unclassified | unclassified |
| Otu177691 | 0.9749 | 1 | 0.987 | 0.001 | Bacteria | Proteobacteria | Alphaproteobacteria | Rhodobacterales | Rhodobacteraceae | Pseudoruegeria | unclassified |
| Otu179324 | 1 | 1 | 1 | 0.001 | Bacteria | Proteobacteria | Alphaproteobacteria | Rhodobacterales | unclassified | unclassified | unclassified |
| Otu179638 | 1 | 1 | 1 | 0.001 | Bacteria | Proteobacteria | Alphaproteobacteria | Rhodobacterales | Rhodobacteraceae | unclassified | unclassified |
| Otu179664 | 1 | 1 | 1 | 0.001 | Bacteria | Proteobacteria | Alphaproteobacteria | Rhodobacterales | Rhodobacteraceae | unclassified | unclassified |
| Otu183356 | 0.8621 | 0.6667 | 0.758 | 0.025 | Bacteria | Cyanobacteria | Synechococcophycideae | Synechococcales | Synechococcaceae | Synechococcus | unclassified |
| Otu183602 | 0.8247 | 0.6667 | 0.741 | 0.047 | Bacteria | Cyanobacteria | Synechococcophycideae | Synechococcales | Synechococcaceae | Synechococcus | unclassified |
| Otu183635 | 1 | 0.6667 | 0.816 | 0.011 | Bacteria | Cyanobacteria | Synechococcophycideae | Synechococcales | Synechococcaceae | Synechococcus | unclassified |
| Otu183683 | 0.8384 | 0.6667 | 0.748 | 0.046 | Bacteria | Cyanobacteria | Synechococcophycideae | Synechococcales | Synechococcaceae | unclassified | unclassified |
| Otu185412 | 1 | 0.6667 | 0.816 | 0.011 | Bacteria | Bacteroidetes | Flavobacteriia | Flavobacteriales | Flavobacteriaceae | unclassified | unclassified |
| Otu193423 | 1 | 0.6667 | 0.816 | 0.005 | Bacteria | Proteobacteria | Gammaproteobacteria | unclassified | unclassified | unclassified | unclassified |
| Otu195714 | 1 | 0.6667 | 0.816 | 0.011 | Bacteria | Bacteroidetes | Flavobacteriia | Flavobacteriales | Flavobacteriaceae | unclassified | unclassified |
| Otu195811 | 1 | 0.6667 | 0.816 | 0.014 | Bacteria | Bacteroidetes | Flavobacteriia | Flavobacteriales | Flavobacteriaceae | unclassified | unclassified |
| Otu222130 | 0.8894 | 0.6667 | 0.77 | 0.022 | Bacteria | Planctomycetes | Planctomycetia | Pirellulales | Pirellulaceae | unclassified | unclassified |
| Otu227006 | 0.846 | 1 | 0.92 | 0.01 | Bacteria | Proteobacteria | unclassified | unclassified | unclassified | unclassified | unclassified |
| Otu227591 | 0.7986 | 0.6667 | 0.73 | 0.049 | Bacteria | unclassified | unclassified | unclassified | unclassified | unclassified | unclassified |
| Otu232398 | 0.902 | 0.6667 | 0.775 | 0.025 | Bacteria | Planctomycetes | Planctomycetia | Pirellulales | Pirellulaceae | unclassified | unclassified |
| Otu234677 | 0.9401 | 0.6667 | 0.792 | 0.016 | Bacteria | Proteobacteria | Gammaproteobacteria | unclassified | unclassified | unclassified | unclassified |
| Otu243356 | 1 | 0.6667 | 0.816 | 0.014 | Bacteria | Proteobacteria | Gammaproteobacteria | unclassified | unclassified | unclassified | unclassified |
| Otu246002 | 0.9509 | 0.6667 | 0.796 | 0.005 | Bacteria | Proteobacteria | Gammaproteobacteria | unclassified | unclassified | unclassified | unclassified |
| Otu252609 | 1 | 0.6667 | 0.816 | 0.011 | Bacteria | Proteobacteria | Gammaproteobacteria | unclassified | unclassified | unclassified | unclassified |

| **0.03 Otus** | ***Geodia barretti*** | **Rest** | **stat** | ***p value*** | **Domain** | **Phylum** | **Class** | **Order** | **Family** | **Genus** | **Species** |
|---|---|---|---|---|---|---|---|---|---|---|---|
| Otu000087 | 0.9083 | 1 | 0.953 | 0.001 | Bacteria | Proteobacteria | Gammaproteobacteria | unclassified | unclassified | unclassified | unclassified |
| Otu000142 | 0.9082 | 1 | 0.953 | 0.001 | Bacteria | Proteobacteria | Gammaproteobacteria | Alteromonadales | HTCC2188 | HTCC | unclassified |
| Otu000159 | 0.7813 | 1 | 0.884 | 0.004 | Bacteria | Actinobacteria | Acidimicrobiia | Acidimicrobiales | TK06 | unclassified | unclassified |
| Otu000218 | 0.9495 | 1 | 0.974 | 0.001 | Bacteria | Acidobacteria | PAUC37f | unclassified | unclassified | unclassified | unclassified |
| Otu000292 | 0.9967 | 1 | 0.998 | 0.001 | Bacteria | Acidobacteria | Acidobacteria-6 | BPC015 | unclassified | unclassified | unclassified |
| Otu000299 | 0.8713 | 0.6667 | 0.762 | 0.033 | Bacteria | Chloroflexi | SAR202 | unclassified | unclassified | unclassified | unclassified |
| Otu000408 | 0.9424 | 0.6667 | 0.793 | 0.014 | Bacteria | Proteobacteria | unclassified | unclassified | unclassified | unclassified | unclassified |
| Otu000474 | 1 | 1 | 1 | 0.001 | Bacteria | Proteobacteria | Deltaproteobacteria | Syntrophobacterales | Syntrophobacteraceae | unclassified | unclassified |
| Otu000488 | 0.9953 | 1 | 0.998 | 0.001 | Bacteria | Chloroflexi | SAR202 | unclassified | unclassified | unclassified | unclassified |
| Otu000527 | 0.9817 | 1 | 0.991 | 0.001 | Bacteria | Proteobacteria | Alphaproteobacteria | Rhodospirillales | Rhodospirillaceae | unclassified | unclassified |
| Otu000531 | 0.9551 | 1 | 0.977 | 0.001 | Bacteria | Chloroflexi | SAR202 | unclassified | unclassified | unclassified | unclassified |
| Otu000540 | 0.9662 | 1 | 0.983 | 0.001 | Bacteria | Proteobacteria | Gammaproteobacteria | unclassified | unclassified | unclassified | unclassified |
| Otu000543 | 1 | 1 | 1 | 0.001 | Bacteria | Proteobacteria | Gammaproteobacteria | unclassified | unclassified | unclassified | unclassified |
| Otu000549 | 0.9724 | 1 | 0.986 | 0.001 | Bacteria | Chloroflexi | SAR202 | unclassified | unclassified | unclassified | unclassified |
| Otu000607 | 1 | 1 | 1 | 0.001 | Bacteria | PAUC34f | unclassified | unclassified | unclassified | unclassified | unclassified |
| Otu000629 | 0.9485 | 1 | 0.974 | 0.002 | Bacteria | Actinobacteria | Acidimicrobiia | Acidimicrobiales | wb1_P06 | unclassified | unclassified |
| Otu000698 | 0.9742 | 1 | 0.987 | 0.001 | Bacteria | Acidobacteria | Solibacteres | Solibacterales | PAUC26f | unclassified | unclassified |
| Otu000730 | 0.9054 | 1 | 0.952 | 0.002 | Bacteria | Acidobacteria | Solibacteres | Solibacterales | PAUC26f | unclassified | unclassified |
| Otu000762 | 0.993 | 1 | 0.996 | 0.001 | Archaea | Crenarchaeota | Thaumarchaeota | Cenarchaeales | Cenarchaeaceae | unclassified | unclassified |
| Otu000773 | 0.9978 | 1 | 0.999 | 0.001 | Bacteria | Proteobacteria | Gammaproteobacteria | unclassified | unclassified | unclassified | unclassified |
| Otu000799 | 0.9976 | 1 | 0.999 | 0.001 | Bacteria | Chloroflexi | Anaerolineae | Caldilineales | Caldilineaceae | unclassified | unclassified |
| Otu000800 | 0.9367 | 1 | 0.968 | 0.001 | Bacteria | Chloroflexi | SAR202 | unclassified | unclassified | unclassified | unclassified |
| Otu000886 | 0.9714 | 1 | 0.986 | 0.001 | Bacteria | Nitrospirae | Nitrospira | Nitrospirales | Nitrospiraceae | unclassified | unclassified |
| Otu000887 | 1 | 0.6667 | 0.816 | 0.01 | Bacteria | Proteobacteria | Deltaproteobacteria | Bdellovibrionales | Bdellovibrionaceae | Bdellovibrio | unclassified |
| Otu000898 | 0.968 | 1 | 0.984 | 0.001 | Bacteria | Chloroflexi | SAR202 | unclassified | unclassified | unclassified | unclassified |
| Otu000915 | 1 | 1 | 1 | 0.001 | Bacteria | Proteobacteria | Gammaproteobacteria | unclassified | unclassified | unclassified | unclassified |
| Otu000937 | 1 | 1 | 1 | 0.001 | Bacteria | Actinobacteria | Acidimicrobiia | Acidimicrobiales | wb1_P06 | unclassified | unclassified |
| Otu000969 | 0.9608 | 1 | 0.98 | 0.001 | Bacteria | Actinobacteria | Acidimicrobiia | Acidimicrobiales | wb1_P06 | unclassified | unclassified |
| Otu000990 | 0.9416 | 1 | 0.97 | 0.001 | Bacteria | SBR1093 | EC214 | unclassified | unclassified | unclassified | unclassified |
| Otu001002 | 1 | 1 | 1 | 0.001 | Bacteria | Chloroflexi | SAR202 | unclassified | unclassified | unclassified | unclassified |
| Otu001003 | 0.8496 | 1 | 0.922 | 0.008 | Bacteria | Chloroflexi | SAR202 | unclassified | unclassified | unclassified | unclassified |
| Otu001016 | 0.9102 | 1 | 0.954 | 0.002 | Bacteria | Actinobacteria | unclassified | unclassified | unclassified | unclassified | unclassified |
| Otu001041 | 1 | 1 | 1 | 0.001 | Bacteria | Chloroflexi | SAR202 | unclassified | unclassified | unclassified | unclassified |
| Otu001073 | 1 | 0.6667 | 0.816 | 0.008 | Bacteria | Proteobacteria | Gammaproteobacteria | unclassified | unclassified | unclassified | unclassified |
| Otu001108 | 1 | 1 | 1 | 0.001 | Bacteria | Chloroflexi | Anaerolineae | SBR1031 | A4b | unclassified | unclassified |
| Otu001135 | 0.9963 | 1 | 0.998 | 0.001 | Bacteria | Chloroflexi | SAR202 | unclassified | unclassified | unclassified | unclassified |
| Otu001180 | 0.9381 | 1 | 0.969 | 0.001 | Bacteria | Acidobacteria | Acidobacteria-6 | iii1-15 | unclassified | unclassified | unclassified |
| Otu001192 | 0.9714 | 1 | 0.986 | 0.001 | Bacteria | Chloroflexi | SAR202 | unclassified | unclassified | unclassified | unclassified |
| Otu001212 | 0.9967 | 1 | 0.998 | 0.001 | Bacteria | Proteobacteria | Deltaproteobacteria | Entotheonellales | Entotheonellaceae | unclassified | unclassified |
| Otu001218 | 0.9143 | 1 | 0.956 | 0.001 | Bacteria | PAUC34f | unclassified | unclassified | unclassified | unclassified | unclassified |
| Otu001237 | 1 | 1 | 1 | 0.001 | Bacteria | Acidobacteria | Solibacteres | Solibacterales | PAUC26f | unclassified | unclassified |
| Otu001257 | 1 | 0.6667 | 0.816 | 0.006 | Archaea | Crenarchaeota | Thaumarchaeota | Cenarchaeales | Cenarchaeaceae | Nitrosopumilus | unclassified |
| Otu001266 | 1 | 1 | 1 | 0.001 | Bacteria | Proteobacteria | Gammaproteobacteria | unclassified | unclassified | unclassified | unclassified |
| Otu001340 | 0.9938 | 1 | 0.997 | 0.001 | Bacteria | Proteobacteria | unclassified | unclassified | unclassified | unclassified | unclassified |
| Otu001342 | 0.7335 | 1 | 0.856 | 0.009 | Bacteria | Proteobacteria | Gammaproteobacteria | unclassified | unclassified | unclassified | unclassified |
| Otu001408 | 1 | 1 | 1 | 0.001 | Archaea | Crenarchaeota | Thaumarchaeota | Cenarchaeales | Cenarchaeaceae | unclassified | unclassified |
| Otu001434 | 1 | 1 | 1 | 0.001 | Bacteria | Proteobacteria | Gammaproteobacteria | unclassified | unclassified | unclassified | unclassified |
| Otu001449 | 1 | 0.6667 | 0.816 | 0.01 | Bacteria | Chloroflexi | SAR202 | unclassified | unclassified | unclassified | unclassified |
| Otu001450 | 0.9393 | 1 | 0.969 | 0.002 | Bacteria | Chloroflexi | SAR202 | unclassified | unclassified | unclassified | unclassified |
| Otu001478 | 1 | 0.6667 | 0.816 | 0.008 | Archaea | Crenarchaeota | Thaumarchaeota | Cenarchaeales | Cenarchaeaceae | Nitrosopumilus | unclassified |
| Otu001509 | 1 | 1 | 1 | 0.001 | Bacteria | Proteobacteria | Gammaproteobacteria | unclassified | unclassified | unclassified | unclassified |
| Otu001514 | 1 | 1 | 1 | 0.001 | Bacteria | Actinobacteria | Acidimicrobiia | Acidimicrobiales | wb1_P06 | unclassified | unclassified |
| Otu001523 | 1 | 1 | 1 | 0.001 | Bacteria | Actinobacteria | unclassified | unclassified | unclassified | unclassified | unclassified |
| Otu001537 | 0.9783 | 1 | 0.989 | 0.001 | Bacteria | Proteobacteria | Gammaproteobacteria | unclassified | unclassified | unclassified | unclassified |
| Otu001560 | 1 | 1 | 1 | 0.001 | Bacteria | Chloroflexi | Anaerolineae | Caldilineales | Caldilineaceae | unclassified | unclassified |
| Otu001586 | 0.9168 | 1 | 0.958 | 0.002 | Bacteria | Actinobacteria | Acidimicrobiia | Acidimicrobiales | wb1_P06 | unclassified | unclassified |
| Otu001666 | 1 | 1 | 1 | 0.001 | Bacteria | Proteobacteria | Gammaproteobacteria | unclassified | unclassified | unclassified | unclassified |
| Otu001737 | 0.6425 | 0.6667 | 0.654 | 0.044 | Bacteria | Proteobacteria | Deltaproteobacteria | Syntrophobacterales | Syntrophobacteraceae | unclassified | unclassified |
| Otu001752 | 1 | 0.6667 | 0.816 | 0.008 | Bacteria | Chloroflexi | SAR202 | unclassified | unclassified | unclassified | unclassified |
| Otu001757 | 1 | 1 | 1 | 0.001 | Archaea | Crenarchaeota | Thaumarchaeota | Cenarchaeales | Cenarchaeaceae | unclassified | unclassified |
| Otu001767 | 1 | 1 | 1 | 0.001 | Bacteria | Proteobacteria | Gammaproteobacteria | Oceanospirillales | Saccharospirillaceae | ML110J-20 | unclassified |
| Otu001826 | 0.9967 | 1 | 0.998 | 0.001 | Bacteria | Chloroflexi | SAR202 | unclassified | unclassified | unclassified | unclassified |
| Otu001849 | 0.9563 | 0.6667 | 0.798 | 0.006 | Bacteria | Acidobacteria | Acidobacteria-6 | iii1-15 | unclassified | unclassified | unclassified |
| Otu001865 | 0.878 | 1 | 0.937 | 0.006 | Bacteria | Chloroflexi | SAR202 | unclassified | unclassified | unclassified | unclassified |
| Otu001871 | 0.988 | 1 | 0.994 | 0.001 | Bacteria | Proteobacteria | Gammaproteobacteria | unclassified | unclassified | unclassified | unclassified |
| Otu001890 | 1 | 1 | 1 | 0.001 | Bacteria | Proteobacteria | Gammaproteobacteria | unclassified | unclassified | unclassified | unclassified |
| Otu001909 | 0.9598 | 0.6667 | 0.8 | 0.006 | Bacteria | Chloroflexi | SAR202 | unclassified | unclassified | unclassified | unclassified |
| Otu001950 | 1 | 0.6667 | 0.816 | 0.01 | Bacteria | Bacteroidetes | Rhodothermi | Rhodothermales | Rhodothermaceae | unclassified | unclassified |
| Otu002058 | 0.8686 | 0.6667 | 0.761 | 0.028 | Bacteria | Proteobacteria | Gammaproteobacteria | unclassified | unclassified | unclassified | unclassified |
| Otu002061 | 1 | 0.6667 | 0.816 | 0.006 | Bacteria | Proteobacteria | Gammaproteobacteria | unclassified | unclassified | unclassified | unclassified |
| Otu002088 | 0.9902 | 1 | 0.995 | 0.001 | Bacteria | Proteobacteria | Deltaproteobacteria | Syntrophobacterales | Syntrophobacteraceae | unclassified | unclassified |
| Otu002094 | 1 | 1 | 1 | 0.001 | Bacteria | Chloroflexi | SAR202 | unclassified | unclassified | unclassified | unclassified |

| | | | | | | | | | | | |
|---|---|---|---|---|---|---|---|---|---|---|---|
| Otu002115 | 1 | 0.6667 | 0.816 | 0.01 | Bacteria | Actinobacteria | Thermoleophilia | Gaiellales | Gaiellaceae | unclassified | unclassified |
| Otu002123 | 0.9879 | 1 | 0.994 | 0.001 | Bacteria | Chloroflexi | Anaerolineae | Caldilineales | Caldilineaceae | unclassified | unclassified |
| Otu002134 | 0.9793 | 1 | 0.99 | 0.001 | Archaea | Crenarchaeota | Thaumarchaeota | Cenarchaeales | Cenarchaeaceae | Nitrosopumilus | unclassified |
| Otu002146 | 1 | 1 | 1 | 0.001 | Bacteria | Proteobacteria | Gammaproteobacteria | unclassified | unclassified | unclassified | unclassified |
| Otu002151 | 1 | 1 | 1 | 0.001 | Bacteria | Proteobacteria | Gammaproteobacteria | HTCC2188 | HTCC2089 | unclassified | unclassified |
| Otu002183 | 0.8791 | 1 | 0.938 | 0.004 | Bacteria | Proteobacteria | Gammaproteobacteria | HTCC2188 | HTCC2089 | unclassified | unclassified |
| Otu002187 | 0.9718 | 1 | 0.986 | 0.001 | Bacteria | Proteobacteria | Gammaproteobacteria | Chromatiales | unclassified | unclassified | unclassified |
| Otu002188 | 1 | 0.6667 | 0.816 | 0.01 | Bacteria | Proteobacteria | Gammaproteobacteria | unclassified | unclassified | unclassified | unclassified |
| Otu002289 | 1 | 1 | 1 | 0.001 | Bacteria | Proteobacteria | Alphaproteobacteria | unclassified | unclassified | unclassified | unclassified |
| Otu002297 | 0.9838 | 1 | 0.992 | 0.001 | Archaea | Crenarchaeota | Thaumarchaeota | Cenarchaeales | Cenarchaeaceae | Nitrosopumilus | unclassified |
| Otu002298 | 1 | 0.6667 | 0.816 | 0.006 | Bacteria | SBR1093 | EC214 | unclassified | unclassified | unclassified | unclassified |
| Otu002366 | 0.9585 | 1 | 0.979 | 0.001 | Bacteria | Proteobacteria | Gammaproteobacteria | unclassified | unclassified | unclassified | unclassified |
| Otu002384 | 1 | 1 | 1 | 0.001 | Bacteria | Acidobacteria | BPC102 | B110 | unclassified | unclassified | unclassified |
| Otu002408 | 1 | 0.6667 | 0.816 | 0.008 | Bacteria | Chloroflexi | SAR202 | unclassified | unclassified | unclassified | unclassified |
| Otu002409 | 1 | 1 | 1 | 0.001 | Bacteria | Chloroflexi | unclassified | unclassified | unclassified | unclassified | unclassified |
| Otu002503 | 1 | 0.6667 | 0.816 | 0.006 | Bacteria | unclassified | unclassified | unclassified | unclassified | unclassified | unclassified |
| Otu002531 | 0.8798 | 0.6667 | 0.766 | 0.035 | Bacteria | Acidobacteria | PAUC37f | unclassified | unclassified | unclassified | unclassified |
| Otu002534 | 0.9878 | 1 | 0.994 | 0.001 | Bacteria | Chloroflexi | SAR202 | unclassified | unclassified | unclassified | unclassified |
| Otu002582 | 1 | 0.6667 | 0.816 | 0.008 | Bacteria | PAUC34f | unclassified | unclassified | unclassified | unclassified | unclassified |
| Otu002597 | 1 | 0.6667 | 0.816 | 0.01 | Bacteria | SBR1093 | EC214 | unclassified | unclassified | unclassified | unclassified |
| Otu002609 | 1 | 1 | 1 | 0.001 | Bacteria | unclassified | unclassified | unclassified | unclassified | unclassified | unclassified |
| Otu002642 | 1 | 1 | 1 | 0.001 | Bacteria | Gemmatimonadetes | Gemm-2 | unclassified | unclassified | unclassified | unclassified |
| Otu002663 | 1 | 1 | 1 | 0.001 | Bacteria | Proteobacteria | Gammaproteobacteria | Oceanospirillales | Saccharospirillaceae | ML110J-20 | unclassified |
| Otu002664 | 1 | 1 | 1 | 0.001 | Bacteria | Proteobacteria | Gammaproteobacteria | HTCC2188 | HTCC2089 | unclassified | unclassified |
| Otu002698 | 1 | 0.6667 | 0.816 | 0.008 | Bacteria | Chloroflexi | SAR202 | unclassified | unclassified | unclassified | unclassified |
| Otu002728 | 1 | 1 | 1 | 0.001 | Bacteria | Chloroflexi | SAR202 | unclassified | unclassified | unclassified | unclassified |
| Otu002732 | 0.9305 | 0.6667 | 0.788 | 0.021 | Bacteria | Actinobacteria | Acidimicrobiia | Acidimicrobiales | wb1_P06 | unclassified | unclassified |
| Otu002736 | 0.9388 | 1 | 0.969 | 0.001 | Bacteria | Actinobacteria | Acidimicrobiia | Acidimicrobiales | wb1_P06 | unclassified | unclassified |
| Otu002829 | 0.8257 | 1 | 0.909 | 0.008 | Bacteria | Chloroflexi | SAR202 | unclassified | unclassified | unclassified | unclassified |
| Otu002834 | 0.9033 | 0.6667 | 0.776 | 0.028 | Bacteria | Proteobacteria | Alphaproteobacteria | unclassified | unclassified | unclassified | unclassified |
| Otu002860 | 1 | 0.6667 | 0.816 | 0.01 | Bacteria | PAUC34f | unclassified | unclassified | unclassified | unclassified | unclassified |
| Otu002902 | 1 | 0.6667 | 0.816 | 0.008 | Bacteria | Proteobacteria | Gammaproteobacteria | unclassified | unclassified | unclassified | unclassified |
| Otu002984 | 1 | 1 | 1 | 0.001 | Bacteria | Chloroflexi | SAR202 | unclassified | unclassified | unclassified | unclassified |
| Otu003012 | 1 | 0.6667 | 0.816 | 0.008 | Bacteria | Chloroflexi | SAR202 | unclassified | unclassified | unclassified | unclassified |
| Otu003023 | 1 | 1 | 1 | 0.001 | Bacteria | Chloroflexi | unclassified | unclassified | unclassified | unclassified | unclassified |
| Otu003048 | 1 | 1 | 1 | 0.001 | Bacteria | Gemmatimonadetes | Gemm-2 | unclassified | unclassified | unclassified | unclassified |
| Otu003117 | 1 | 0.6667 | 0.816 | 0.008 | Bacteria | Chloroflexi | SAR202 | unclassified | unclassified | unclassified | unclassified |
| Otu003169 | 1 | 1 | 1 | 0.001 | Bacteria | Chloroflexi | SAR202 | unclassified | unclassified | unclassified | unclassified |
| Otu003240 | 1 | 1 | 1 | 0.001 | Bacteria | Chloroflexi | unclassified | unclassified | unclassified | unclassified | unclassified |
| Otu003291 | 1 | 1 | 1 | 0.001 | Bacteria | Proteobacteria | Gammaproteobacteria | unclassified | unclassified | unclassified | unclassified |
| Otu003307 | 1 | 0.6667 | 0.816 | 0.01 | Bacteria | Proteobacteria | Gammaproteobacteria | unclassified | unclassified | unclassified | unclassified |
| Otu003363 | 1 | 0.6667 | 0.816 | 0.01 | Bacteria | PAUC34f | unclassified | unclassified | unclassified | unclassified | unclassified |
| Otu003371 | 1 | 1 | 1 | 0.001 | Bacteria | Acidobacteria | Solibacteres | Solibacterales | PAUC26f | unclassified | unclassified |
| Otu003380 | 1 | 0.6667 | 0.816 | 0.008 | Archaea | Crenarchaeota | Thaumarchaeota | Cenarchaeales | Cenarchaeaceae | Nitrosopumilus | unclassified |
| Otu003414 | 1 | 1 | 1 | 0.001 | Bacteria | Proteobacteria | Gammaproteobacteria | unclassified | unclassified | unclassified | unclassified |
| Otu003449 | 1 | 0.6667 | 0.816 | 0.008 | Bacteria | Proteobacteria | Deltaproteobacteria | Bdellovibrionales | Bdellovibrionaceae | Bdellovibrio | unclassified |
| Otu003496 | 1 | 0.6667 | 0.816 | 0.006 | Bacteria | Chloroflexi | SAR202 | unclassified | unclassified | unclassified | unclassified |
| Otu003611 | 1 | 1 | 1 | 0.001 | Bacteria | Acidobacteria | Acidobacteria-6 | iii1-15 | unclassified | unclassified | unclassified |
| Otu003701 | 0.9785 | 1 | 0.989 | 0.001 | Bacteria | Proteobacteria | Gammaproteobacteria | unclassified | unclassified | unclassified | unclassified |
| Otu003729 | 0.8924 | 0.6667 | 0.771 | 0.017 | Bacteria | Chloroflexi | SAR202 | unclassified | unclassified | unclassified | unclassified |
| Otu003904 | 1 | 0.6667 | 0.816 | 0.01 | Bacteria | Acidobacteria | Acidobacteria-6 | iii1-15 | unclassified | unclassified | unclassified |
| Otu003923 | 1 | 0.6667 | 0.816 | 0.01 | Bacteria | Gemmatimonadetes | Gemm-2 | unclassified | unclassified | unclassified | unclassified |
| Otu003930 | 1 | 1 | 1 | 0.001 | Bacteria | Acidobacteria | Solibacteres | Solibacterales | PAUC26f | unclassified | unclassified |
| Otu003935 | 1 | 1 | 1 | 0.001 | Bacteria | unclassified | unclassified | unclassified | unclassified | unclassified | unclassified |
| Otu003989 | 0.8894 | 1 | 0.943 | 0.003 | Bacteria | Chloroflexi | SAR202 | unclassified | unclassified | unclassified | unclassified |
| Otu004219 | 1 | 1 | 1 | 0.001 | Bacteria | Chloroflexi | SAR202 | unclassified | unclassified | unclassified | unclassified |
| Otu004221 | 0.8876 | 0.6667 | 0.769 | 0.031 | Bacteria | Chloroflexi | SAR202 | unclassified | unclassified | unclassified | unclassified |
| Otu004232 | 1 | 0.6667 | 0.816 | 0.01 | Bacteria | Chloroflexi | SAR202 | unclassified | unclassified | unclassified | unclassified |
| Otu004240 | 1 | 1 | 1 | 0.001 | Bacteria | Acidobacteria | Acidobacteria-6 | iii1-15 | unclassified | unclassified | unclassified |
| Otu004250 | 1 | 1 | 1 | 0.001 | Bacteria | Acidobacteria | Solibacteres | Solibacterales | PAUC26f | unclassified | unclassified |
| Otu004304 | 0.9457 | 0.6667 | 0.794 | 0.021 | Archaea | Crenarchaeota | Thaumarchaeota | Cenarchaeales | Cenarchaeaceae | Nitrosopumilus | unclassified |
| Otu004308 | 1 | 0.6667 | 0.816 | 0.01 | Bacteria | Acidobacteria | PAUC37f | unclassified | unclassified | unclassified | unclassified |
| Otu004327 | 1 | 0.6667 | 0.816 | 0.01 | Bacteria | SBR1093 | EC214 | unclassified | unclassified | unclassified | unclassified |
| Otu004337 | 1 | 0.6667 | 0.816 | 0.006 | Archaea | Crenarchaeota | Thaumarchaeota | Cenarchaeales | Cenarchaeaceae | Nitrosopumilus | unclassified |
| Otu004340 | 0.9066 | 0.6667 | 0.777 | 0.028 | Bacteria | Proteobacteria | Deltaproteobacteria | Entotheonellales | Entotheonellaceae | unclassified | unclassified |
| Otu004341 | 1 | 0.6667 | 0.816 | 0.006 | Archaea | Crenarchaeota | Thaumarchaeota | Cenarchaeales | Cenarchaeaceae | Nitrosopumilus | unclassified |
| Otu004379 | 0.9245 | 1 | 0.962 | 0.001 | Bacteria | AncK6 | unclassified | unclassified | unclassified | unclassified | unclassified |
| Otu004394 | 0.9936 | 1 | 0.997 | 0.001 | Archaea | Crenarchaeota | Thaumarchaeota | Cenarchaeales | Cenarchaeaceae | unclassified | unclassified |
| Otu004403 | 1 | 0.6667 | 0.816 | 0.008 | Bacteria | Chloroflexi | SAR202 | unclassified | unclassified | unclassified | unclassified |
| Otu004406 | 1 | 0.6667 | 0.816 | 0.01 | Bacteria | Chloroflexi | SAR202 | unclassified | unclassified | unclassified | unclassified |
| Otu004762 | 1 | 1 | 1 | 0.001 | Bacteria | Proteobacteria | Gammaproteobacteria | unclassified | unclassified | unclassified | unclassified |
| Otu004789 | 1 | 1 | 1 | 0.001 | Bacteria | SBR1093 | EC214 | unclassified | unclassified | unclassified | unclassified |
| Otu004823 | 1 | 1 | 1 | 0.001 | Bacteria | Actinobacteria | Acidimicrobiia | Acidimicrobiales | wb1_P06 | unclassified | unclassified |
| Otu004828 | 0.972 | 1 | 0.986 | 0.001 | Bacteria | Acidobacteria | Acidobacteria-6 | iii1-15 | unclassified | unclassified | unclassified |
| Otu004964 | 1 | 1 | 1 | 0.001 | Bacteria | unclassified | unclassified | unclassified | unclassified | unclassified | unclassified |
| Otu004974 | 1 | 1 | 1 | 0.001 | Bacteria | Chloroflexi | SAR202 | unclassified | unclassified | unclassified | unclassified |
| Otu004976 | 1 | 1 | 1 | 0.001 | Bacteria | Chloroflexi | Anaerolineae | Caldilineales | Caldilineaceae | unclassified | unclassified |
| Otu005118 | 0.9233 | 0.6667 | 0.785 | 0.027 | Bacteria | Chloroflexi | SAR202 | unclassified | unclassified | unclassified | unclassified |
| Otu005120 | 1 | 0.6667 | 0.816 | 0.008 | Bacteria | Chloroflexi | SAR202 | unclassified | unclassified | unclassified | unclassified |
| Otu005122 | 1 | 0.6667 | 0.816 | 0.006 | Bacteria | Chloroflexi | SAR202 | unclassified | unclassified | unclassified | unclassified |
| Otu005126 | 1 | 1 | 1 | 0.001 | Bacteria | Chloroflexi | SAR202 | unclassified | unclassified | unclassified | unclassified |
| Otu005166 | 1 | 1 | 1 | 0.001 | Bacteria | Acidobacteria | Solibacteres | Solibacterales | PAUC26f | unclassified | unclassified |
| Otu005175 | 1 | 0.6667 | 0.816 | 0.01 | Archaea | Crenarchaeota | Thaumarchaeota | Cenarchaeales | Cenarchaeaceae | Nitrosopumilus | unclassified |
| Otu005221 | 1 | 1 | 1 | 0.001 | Bacteria | Actinobacteria | unclassified | unclassified | unclassified | unclassified | unclassified |
| Otu005229 | 1 | 1 | 1 | 0.001 | Bacteria | Chloroflexi | unclassified | unclassified | unclassified | unclassified | unclassified |
| Otu005230 | 1 | 1 | 1 | 0.001 | Bacteria | Acidobacteria | Acidobacteria-6 | iii1-15 | unclassified | unclassified | unclassified |
| Otu005241 | 1 | 1 | 1 | 0.001 | Bacteria | Chloroflexi | SAR202 | unclassified | unclassified | unclassified | unclassified |
| Otu005283 | 1 | 0.6667 | 0.816 | 0.01 | Bacteria | Proteobacteria | unclassified | unclassified | unclassified | unclassified | unclassified |
| Otu005296 | 0.985 | 1 | 0.992 | 0.001 | Bacteria | Proteobacteria | unclassified | unclassified | unclassified | unclassified | unclassified |
| Otu005345 | 1 | 0.6667 | 0.816 | 0.01 | Bacteria | Gemmatimonadetes | Gemm-4 | unclassified | unclassified | unclassified | unclassified |
| Otu005346 | 0.9978 | 1 | 0.999 | 0.001 | Bacteria | Proteobacteria | Alphaproteobacteria | unclassified | unclassified | unclassified | unclassified |
| Otu005353 | 0.8878 | 1 | 0.942 | 0.003 | Bacteria | Acidobacteria | Acidobacteria-6 | iii1-15 | unclassified | unclassified | unclassified |
| Otu005411 | 1 | 0.6667 | 0.816 | 0.01 | Bacteria | Chloroflexi | Ktedonobacteria | unclassified | unclassified | unclassified | unclassified |
| Otu005460 | 0.7634 | 1 | 0.874 | 0.008 | Bacteria | Chloroflexi | SAR202 | unclassified | unclassified | unclassified | unclassified |
| Otu005509 | 1 | 1 | 1 | 0.001 | Bacteria | Chloroflexi | SAR202 | unclassified | unclassified | unclassified | unclassified |
| Otu005511 | 1 | 0.6667 | 0.816 | 0.01 | Bacteria | Chloroflexi | SAR202 | unclassified | unclassified | unclassified | unclassified |
| Otu005534 | 1 | 0.6667 | 0.816 | 0.01 | Bacteria | Proteobacteria | Alphaproteobacteria | Rhodospirillales | unclassified | unclassified | unclassified |
| Otu005543 | 1 | 0.6667 | 0.816 | 0.01 | Bacteria | Proteobacteria | Alphaproteobacteria | unclassified | unclassified | unclassified | unclassified |
| Otu005596 | 0.9352 | 0.6667 | 0.79 | 0.018 | Bacteria | Proteobacteria | Gammaproteobacteria | HTCC2188 | HTCC2089 | unclassified | unclassified |
| Otu005624 | 0.9451 | 1 | 0.972 | 0.002 | Bacteria | Proteobacteria | Gammaproteobacteria | unclassified | unclassified | unclassified | unclassified |

| | | | | | | | | | | | |
|---|---|---|---|---|---|---|---|---|---|---|---|
| Otu005681 | 1 | 1 | 1 | 0.001 | Bacteria | Proteobacteria | Alphaproteobacteria | Ellin329 | unclassified | unclassified | unclassified |
| Otu005698 | 0.9615 | 1 | 0.981 | 0.001 | Bacteria | Proteobacteria | Deltaproteobacteria | unclassified | unclassified | unclassified | unclassified |
| Otu005811 | 0.9905 | 1 | 0.995 | 0.001 | Bacteria | Acidobacteria | Acidobacteria-6 | iii1-15 | unclassified | unclassified | unclassified |
| Otu005893 | 1 | 0.6667 | 0.816 | 0.008 | Bacteria | SBR1093 | EC214 | unclassified | unclassified | unclassified | unclassified |
| Otu005933 | 0.9433 | 1 | 0.971 | 0.001 | Bacteria | Actinobacteria | Acidimicrobiia | Acidimicrobiales | wb1_P06 | unclassified | unclassified |
| Otu006051 | 1 | 1 | 1 | 0.001 | Bacteria | Chloroflexi | Anaerolineae | SBR1031 | A4b | unclassified | unclassified |
| Otu006054 | 1 | 0.6667 | 0.816 | 0.01 | Bacteria | SBR1093 | EC214 | unclassified | unclassified | unclassified | unclassified |
| Otu006103 | 1 | 1 | 1 | 0.001 | Bacteria | Actinobacteria | Acidimicrobiia | Acidimicrobiales | wb1_P06 | unclassified | unclassified |
| Otu006115 | 0.9575 | 1 | 0.979 | 0.001 | Bacteria | Proteobacteria | Gammaproteobacteria | unclassified | unclassified | unclassified | unclassified |
| Otu006117 | 0.9807 | 1 | 0.99 | 0.001 | Bacteria | Proteobacteria | Gammaproteobacteria | unclassified | unclassified | unclassified | unclassified |
| Otu006259 | 1 | 1 | 1 | 0.001 | Bacteria | Chloroflexi | TK17 | TK18 | unclassified | unclassified | unclassified |
| Otu006335 | 0.9333 | 0.6667 | 0.789 | 0.01 | Archaea | Crenarchaeota | Thaumarchaeota | Cenarchaeales | Cenarchaeaceae | Nitrosopumilus | unclassified |
| Otu006357 | 1 | 0.6667 | 0.816 | 0.006 | Bacteria | Chloroflexi | TK17 | mle1-48 | unclassified | unclassified | unclassified |
| Otu006397 | 0.8835 | 1 | 0.94 | 0.004 | Bacteria | Proteobacteria | Gammaproteobacteria | HTCC2188 | HTCC2089 | unclassified | unclassified |
| Otu006474 | 1 | 0.6667 | 0.816 | 0.01 | Bacteria | Proteobacteria | Gammaproteobacteria | unclassified | unclassified | unclassified | unclassified |
| Otu006485 | 1 | 1 | 1 | 0.001 | Bacteria | PAUC34f | unclassified | unclassified | unclassified | unclassified | unclassified |
| Otu006503 | 1 | 0.6667 | 0.816 | 0.01 | Bacteria | Chloroflexi | Anaerolineae | SBR1031 | A4b | unclassified | unclassified |
| Otu006517 | 1 | 1 | 1 | 0.001 | Bacteria | Chloroflexi | Ktedonobacteria | TK10 | unclassified | unclassified | unclassified |
| Otu006526 | 1 | 0.6667 | 0.816 | 0.008 | Bacteria | Chloroflexi | SAR202 | unclassified | unclassified | unclassified | unclassified |
| Otu006596 | 1 | 0.6667 | 0.816 | 0.01 | Bacteria | Chloroflexi | SAR202 | unclassified | unclassified | unclassified | unclassified |
| Otu006628 | 0.8895 | 1 | 0.943 | 0.004 | Bacteria | Chloroflexi | SAR202 | unclassified | unclassified | unclassified | unclassified |
| Otu006746 | 1 | 1 | 1 | 0.001 | Bacteria | Spirochaetes | Spirochaetes | Spirochaetales | Spirochaetaceae | unclassified | unclassified |
| Otu006932 | 1 | 0.6667 | 0.816 | 0.006 | Bacteria | Acidobacteria | Acidobacteria-6 | iii1-15 | unclassified | unclassified | unclassified |
| Otu006947 | 1 | 1 | 1 | 0.001 | Bacteria | Acidobacteria | TM1 | unclassified | unclassified | unclassified | unclassified |
| Otu006982 | 0.9792 | 1 | 0.99 | 0.001 | Bacteria | Spirochaetes | Spirochaetes | Spirochaetales | Spirochaetaceae | unclassified | unclassified |
| Otu006993 | 0.9278 | 0.6667 | 0.786 | 0.032 | Bacteria | Acidobacteria | PAUC37f | unclassified | unclassified | unclassified | unclassified |
| Otu007049 | 1 | 1 | 1 | 0.001 | Archaea | Crenarchaeota | Thaumarchaeota | Cenarchaeales | Cenarchaeaceae | Nitrosopumilus | unclassified |
| Otu007050 | 1 | 0.6667 | 0.816 | 0.006 | Archaea | Crenarchaeota | Thaumarchaeota | Cenarchaeales | Cenarchaeaceae | Nitrosopumilus | unclassified |
| Otu007053 | 0.9648 | 1 | 0.982 | 0.001 | Archaea | Crenarchaeota | Thaumarchaeota | Cenarchaeales | Cenarchaeaceae | Nitrosopumilus | unclassified |
| Otu007080 | 0.9927 | 1 | 0.996 | 0.001 | Bacteria | Acidobacteria | Solibacteres | Solibacterales | PAUC26f | unclassified | unclassified |
| Otu007107 | 1 | 0.6667 | 0.816 | 0.008 | Archaea | Crenarchaeota | Thaumarchaeota | Cenarchaeales | Cenarchaeaceae | Nitrosopumilus | unclassified |
| Otu007155 | 1 | 0.6667 | 0.816 | 0.006 | Bacteria | Acidobacteria | BPC102 | B110 | unclassified | unclassified | unclassified |
| Otu007180 | 1 | 1 | 1 | 0.001 | Bacteria | Chloroflexi | unclassified | unclassified | unclassified | unclassified | unclassified |
| Otu007207 | 0.9925 | 1 | 0.996 | 0.001 | Bacteria | Chloroflexi | SAR202 | unclassified | unclassified | unclassified | unclassified |
| Otu007214 | 1 | 1 | 1 | 0.001 | Bacteria | Chloroflexi | Anaerolineae | SBR1031 | A4b | unclassified | unclassified |
| Otu007553 | 1 | 1 | 1 | 0.001 | Bacteria | Acidobacteria | Acidobacteria-6 | iii1-15 | unclassified | unclassified | unclassified |
| Otu007761 | 1 | 1 | 1 | 0.001 | Bacteria | Proteobacteria | Gammaproteobacteria | unclassified | unclassified | unclassified | unclassified |
| Otu007770 | 1 | 0.6667 | 0.816 | 0.008 | Bacteria | Proteobacteria | Gammaproteobacteria | unclassified | unclassified | unclassified | unclassified |
| Otu007792 | 0.9233 | 0.6667 | 0.785 | 0.027 | Bacteria | Proteobacteria | Gammaproteobacteria | unclassified | unclassified | unclassified | unclassified |
| Otu007813 | 1 | 0.6667 | 0.816 | 0.006 | Archaea | Crenarchaeota | Thaumarchaeota | Cenarchaeales | Cenarchaeaceae | Nitrosopumilus | unclassified |
| Otu007855 | 1 | 1 | 1 | 0.001 | Bacteria | Acidobacteria | Solibacteres | Solibacterales | PAUC26f | unclassified | unclassified |
| Otu007879 | 1 | 0.6667 | 0.816 | 0.01 | Archaea | Crenarchaeota | Thaumarchaeota | Cenarchaeales | Cenarchaeaceae | Nitrosopumilus | unclassified |
| Otu007892 | 1 | 1 | 1 | 0.001 | Archaea | Crenarchaeota | Thaumarchaeota | Cenarchaeales | Cenarchaeaceae | unclassified | unclassified |
| Otu007921 | 1 | 0.6667 | 0.816 | 0.008 | Bacteria | Chloroflexi | SAR202 | unclassified | unclassified | unclassified | unclassified |
| Otu007963 | 1 | 1 | 1 | 0.001 | Archaea | Crenarchaeota | Thaumarchaeota | Cenarchaeales | Cenarchaeaceae | unclassified | unclassified |
| Otu007996 | 1 | 1 | 1 | 0.001 | Bacteria | Gemmatimonadetes | Gemm-2 | unclassified | unclassified | unclassified | unclassified |
| Otu008193 | 1 | 1 | 1 | 0.001 | Bacteria | unclassified | unclassified | unclassified | unclassified | unclassified | unclassified |
| Otu008241 | 1 | 1 | 1 | 0.001 | Bacteria | Verrucomicrobia | Opitutae | unclassified | unclassified | unclassified | unclassified |
| Otu008254 | 0.89 | 1 | 0.943 | 0.001 | Bacteria | Proteobacteria | Alphaproteobacteria | unclassified | unclassified | unclassified | unclassified |
| Otu008336 | 1 | 0.6667 | 0.816 | 0.006 | Bacteria | Chloroflexi | SAR202 | unclassified | unclassified | unclassified | unclassified |
| Otu008384 | 0.9553 | 1 | 0.977 | 0.001 | Bacteria | Chloroflexi | SAR202 | unclassified | unclassified | unclassified | unclassified |
| Otu008388 | 1 | 1 | 1 | 0.001 | Bacteria | Chloroflexi | SAR202 | unclassified | unclassified | unclassified | unclassified |
| Otu008411 | 1 | 0.6667 | 0.816 | 0.006 | Bacteria | Proteobacteria | Gammaproteobacteria | unclassified | unclassified | unclassified | unclassified |
| Otu008662 | 1 | 0.6667 | 0.816 | 0.01 | Bacteria | Proteobacteria | Gammaproteobacteria | unclassified | unclassified | unclassified | unclassified |
| Otu008801 | 1 | 1 | 1 | 0.001 | Bacteria | Acidobacteria | Acidobacteria-6 | iii1-15 | unclassified | unclassified | unclassified |
| Otu008810 | 1 | 1 | 1 | 0.001 | Bacteria | Gemmatimonadetes | Gemm-2 | unclassified | unclassified | unclassified | unclassified |
| Otu009004 | 1 | 1 | 1 | 0.001 | Bacteria | unclassified | unclassified | unclassified | unclassified | unclassified | unclassified |
| Otu009048 | 1 | 0.6667 | 0.816 | 0.006 | Archaea | Crenarchaeota | Thaumarchaeota | Cenarchaeales | Cenarchaeaceae | Nitrosopumilus | unclassified |
| Otu009060 | 1 | 0.6667 | 0.816 | 0.01 | Bacteria | Gemmatimonadetes | Gemm-2 | unclassified | unclassified | unclassified | unclassified |
| Otu009072 | 1 | 0.6667 | 0.816 | 0.008 | Archaea | Crenarchaeota | Thaumarchaeota | Cenarchaeales | Cenarchaeaceae | Nitrosopumilus | unclassified |
| Otu009073 | 1 | 0.6667 | 0.816 | 0.01 | Bacteria | Chloroflexi | SAR202 | unclassified | unclassified | unclassified | unclassified |
| Otu009188 | 1 | 1 | 1 | 0.001 | Bacteria | Proteobacteria | Gammaproteobacteria | unclassified | unclassified | unclassified | unclassified |
| Otu009200 | 1 | 0.6667 | 0.816 | 0.008 | Bacteria | SBR1093 | EC214 | unclassified | unclassified | unclassified | unclassified |
| Otu009239 | 1 | 0.6667 | 0.816 | 0.01 | Bacteria | Proteobacteria | Alphaproteobacteria | unclassified | unclassified | unclassified | unclassified |
| Otu009295 | 1 | 1 | 1 | 0.001 | Bacteria | unclassified | unclassified | unclassified | unclassified | unclassified | unclassified |
| Otu009297 | 1 | 0.6667 | 0.816 | 0.01 | Bacteria | Proteobacteria | Deltaproteobacteria | Bdellovibrionales | Bdellovibrionaceae | Bdellovibrio | unclassified |
| Otu009318 | 1 | 1 | 1 | 0.001 | Bacteria | Proteobacteria | Gammaproteobacteria | unclassified | unclassified | unclassified | unclassified |
| Otu009325 | 1 | 0.6667 | 0.816 | 0.01 | Bacteria | Chloroflexi | SAR202 | unclassified | unclassified | unclassified | unclassified |
| Otu009415 | 1 | 0.6667 | 0.816 | 0.01 | Bacteria | unclassified | unclassified | unclassified | unclassified | unclassified | unclassified |
| Otu009482 | 1 | 1 | 1 | 0.001 | Bacteria | Proteobacteria | unclassified | unclassified | unclassified | unclassified | unclassified |
| Otu009505 | 1 | 0.6667 | 0.816 | 0.006 | Bacteria | SBR1093 | EC214 | unclassified | unclassified | unclassified | unclassified |
| Otu009786 | 1 | 1 | 1 | 0.001 | Bacteria | Chloroflexi | SAR202 | unclassified | unclassified | unclassified | unclassified |
| Otu009789 | 0.9924 | 1 | 0.996 | 0.001 | Archaea | Crenarchaeota | Thaumarchaeota | Cenarchaeales | Cenarchaeaceae | unclassified | unclassified |
| Otu009850 | 1 | 1 | 1 | 0.001 | Archaea | Crenarchaeota | Thaumarchaeota | Cenarchaeales | Cenarchaeaceae | unclassified | unclassified |
| Otu009997 | 1 | 0.6667 | 0.816 | 0.01 | Bacteria | Gemmatimonadetes | Gemm-2 | unclassified | unclassified | unclassified | unclassified |
| Otu010089 | 1 | 0.6667 | 0.816 | 0.006 | Bacteria | Chloroflexi | TK17 | TK18 | unclassified | unclassified | unclassified |
| Otu010091 | 1 | 1 | 1 | 0.001 | Bacteria | Acidobacteria | BPC102 | B110 | unclassified | unclassified | unclassified |
| Otu010093 | 1 | 1 | 1 | 0.001 | Bacteria | Acidobacteria | Acidobacteria-6 | iii1-15 | unclassified | unclassified | unclassified |
| Otu010105 | 1 | 1 | 1 | 0.001 | Archaea | Crenarchaeota | Thaumarchaeota | Cenarchaeales | Cenarchaeaceae | Nitrosopumilus | unclassified |
| Otu010194 | 1 | 0.6667 | 0.816 | 0.01 | Bacteria | PAUC34f | unclassified | unclassified | unclassified | unclassified | unclassified |
| Otu010318 | 1 | 1 | 1 | 0.001 | Bacteria | Gemmatimonadetes | Gemm-2 | unclassified | unclassified | unclassified | unclassified |
| Otu010347 | 1 | 0.6667 | 0.816 | 0.01 | Archaea | Crenarchaeota | Thaumarchaeota | Cenarchaeales | Cenarchaeaceae | Nitrosopumilus | unclassified |
| Otu010361 | 1 | 1 | 1 | 0.001 | Archaea | Crenarchaeota | Thaumarchaeota | Cenarchaeales | Cenarchaeaceae | Nitrosopumilus | unclassified |
| Otu010461 | 1 | 1 | 1 | 0.001 | Bacteria | Acidobacteria | Solibacteres | Solibacterales | PAUC26f | unclassified | unclassified |
| Otu010462 | 1 | 1 | 1 | 0.001 | Bacteria | Chloroflexi | SAR202 | unclassified | unclassified | unclassified | unclassified |
| Otu010465 | 1 | 0.6667 | 0.816 | 0.006 | Bacteria | Acidobacteria | Solibacteres | Solibacterales | PAUC26f | unclassified | unclassified |
| Otu010501 | 1 | 1 | 1 | 0.001 | Bacteria | Nitrospirae | Nitrospira | Nitrospirales | Nitrospiraceae | unclassified | unclassified |
| Otu010560 | 1 | 0.6667 | 0.816 | 0.01 | Bacteria | Proteobacteria | Gammaproteobacteria | unclassified | unclassified | unclassified | unclassified |
| Otu010725 | 1 | 1 | 1 | 0.001 | Bacteria | Proteobacteria | Gammaproteobacteria | unclassified | unclassified | unclassified | unclassified |
| Otu010778 | 1 | 1 | 1 | 0.001 | Bacteria | Acidobacteria | Acidobacteria-6 | iii1-15 | unclassified | unclassified | unclassified |
| Otu010871 | 1 | 0.6667 | 0.816 | 0.006 | Bacteria | Actinobacteria | Acidimicrobiia | Acidimicrobiales | koll13 | unclassified | unclassified |
| Otu010923 | 1 | 0.6667 | 0.816 | 0.008 | Bacteria | Actinobacteria | Acidimicrobiia | Acidimicrobiales | wb1_P06 | unclassified | unclassified |
| Otu010973 | 1 | 1 | 1 | 0.001 | Bacteria | Chloroflexi | SAR202 | unclassified | unclassified | unclassified | unclassified |
| Otu010980 | 1 | 1 | 1 | 0.001 | Bacteria | Chloroflexi | SAR202 | unclassified | unclassified | unclassified | unclassified |
| Otu011018 | 0.9807 | 1 | 0.99 | 0.001 | Bacteria | unclassified | unclassified | unclassified | unclassified | unclassified | unclassified |
| Otu011105 | 1 | 0.6667 | 0.816 | 0.008 | Bacteria | SBR1093 | EC214 | unclassified | unclassified | unclassified | unclassified |
| Otu011264 | 1 | 0.6667 | 0.816 | 0.01 | Bacteria | Chloroflexi | SAR202 | unclassified | unclassified | unclassified | unclassified |
| Otu011327 | 0.9337 | 0.6667 | 0.789 | 0.031 | Bacteria | Chloroflexi | SAR202 | unclassified | unclassified | unclassified | unclassified |
| Otu011364 | 1 | 1 | 1 | 0.001 | Bacteria | Chloroflexi | SAR202 | unclassified | unclassified | unclassified | unclassified |
| Otu011486 | 1 | 1 | 1 | 0.001 | Bacteria | Chloroflexi | SAR202 | unclassified | unclassified | unclassified | unclassified |

| | | | | | | | | | | | |
|---|---|---|---|---|---|---|---|---|---|---|---|
| Otu011513 | 1 | 1 | 1 | 0.001 | Bacteria | Chloroflexi | SAR202 | unclassified | unclassified | unclassified | unclassified |
| Otu011577 | 1 | 0.6667 | 0.816 | 0.006 | Bacteria | Acidobacteria | PAUC37f | unclassified | unclassified | unclassified | unclassified |
| Otu011662 | 1 | 1 | 1 | 0.001 | Bacteria | Proteobacteria | Gammaproteobacteria | unclassified | unclassified | unclassified | unclassified |
| Otu011688 | 0.9669 | 1 | 0.983 | 0.001 | Bacteria | Proteobacteria | Gammaproteobacteria | unclassified | unclassified | unclassified | unclassified |
| Otu011702 | 1 | 1 | 1 | 0.001 | Bacteria | Proteobacteria | Gammaproteobacteria | unclassified | unclassified | unclassified | unclassified |
| Otu011983 | 1 | 1 | 1 | 0.001 | Bacteria | Acidobacteria | Acidobacteria-5 | unclassified | unclassified | unclassified | unclassified |
| Otu012092 | 1 | 0.6667 | 0.816 | 0.008 | Bacteria | Proteobacteria | Alphaproteobacteria | unclassified | unclassified | unclassified | unclassified |
| Otu012154 | 0.7327 | 1 | 0.856 | 0.008 | Bacteria | Proteobacteria | Gammaproteobacteria | unclassified | unclassified | unclassified | unclassified |
| Otu012180 | 1 | 1 | 1 | 0.001 | Bacteria | Proteobacteria | Gammaproteobacteria | HTCC2188 | HTCC2089 | unclassified | unclassified |
| Otu012186 | 1 | 1 | 1 | 0.001 | Bacteria | Proteobacteria | Alphaproteobacteria | unclassified | unclassified | unclassified | unclassified |
| Otu012244 | 1 | 1 | 1 | 0.001 | Bacteria | Actinobacteria | Acidimicrobiia | Acidimicrobiales | unclassified | unclassified | unclassified |
| Otu012313 | 0.9379 | 0.6667 | 0.791 | 0.011 | Bacteria | SBR1093 | EC214 | unclassified | unclassified | unclassified | unclassified |
| Otu012314 | 1 | 1 | 1 | 0.001 | Bacteria | Acidobacteria | BPC102 | B110 | unclassified | unclassified | unclassified |
| Otu012394 | 1 | 0.6667 | 0.816 | 0.008 | Bacteria | Thermi | Deinococci | Deinococcales | unclassified | unclassified | unclassified |
| Otu012775 | 1 | 0.6667 | 0.816 | 0.01 | Bacteria | Actinobacteria | Acidimicrobiia | Acidimicrobiales | wb1_P06 | unclassified | unclassified |
| Otu012827 | 1 | 0.6667 | 0.816 | 0.01 | Bacteria | Acidobacteria | PAUC37f | unclassified | unclassified | unclassified | unclassified |
| Otu012836 | 1 | 0.6667 | 0.816 | 0.006 | Bacteria | SBR1093 | EC214 | unclassified | unclassified | unclassified | unclassified |
| Otu012858 | 1 | 0.6667 | 0.816 | 0.01 | Bacteria | Nitrospirae | Nitrospira | Nitrospirales | Nitrospiraceae | unclassified | unclassified |
| Otu012866 | 1 | 1 | 1 | 0.001 | Bacteria | Poribacteria | unclassified | unclassified | unclassified | unclassified | unclassified |
| Otu012996 | 1 | 1 | 1 | 0.001 | Bacteria | Acidobacteria | Acidobacteria-6 | BPC015 | unclassified | unclassified | unclassified |
| Otu013018 | 1 | 1 | 1 | 0.001 | Bacteria | Chloroflexi | SAR202 | unclassified | unclassified | unclassified | unclassified |
| Otu013067 | 1 | 0.6667 | 0.816 | 0.008 | Bacteria | SBR1093 | EC214 | unclassified | unclassified | unclassified | unclassified |
| Otu013106 | 0.9473 | 1 | 0.973 | 0.001 | Bacteria | SBR1093 | EC214 | unclassified | unclassified | unclassified | unclassified |
| Otu013115 | 1 | 0.6667 | 0.816 | 0.01 | Bacteria | SBR1093 | EC214 | unclassified | unclassified | unclassified | unclassified |
| Otu013143 | 1 | 0.6667 | 0.816 | 0.01 | Bacteria | PAUC34f | unclassified | unclassified | unclassified | unclassified | unclassified |
| Otu013174 | 1 | 0.6667 | 0.816 | 0.01 | Bacteria | PAUC34f | unclassified | unclassified | unclassified | unclassified | unclassified |
| Otu013255 | 0.9831 | 1 | 0.992 | 0.001 | Bacteria | Proteobacteria | Gammaproteobacteria | unclassified | unclassified | unclassified | unclassified |
| Otu013316 | 1 | 0.6667 | 0.816 | 0.006 | Bacteria | Proteobacteria | Gammaproteobacteria | Alteromonadales | HTCC2188 | HTCC | unclassified |
| Otu013343 | 1 | 0.6667 | 0.816 | 0.01 | Bacteria | Proteobacteria | Gammaproteobacteria | Alteromonadales | unclassified | unclassified | unclassified |
| Otu013695 | 1 | 1 | 1 | 0.001 | Archaea | Crenarchaeota | Thaumarchaeota | Cenarchaeales | Cenarchaeaceae | Nitrosopumilus | unclassified |
| Otu013873 | 1 | 1 | 1 | 0.001 | Bacteria | Proteobacteria | Alphaproteobacteria | unclassified | unclassified | unclassified | unclassified |
| Otu014388 | 1 | 1 | 1 | 0.001 | Bacteria | Proteobacteria | Alphaproteobacteria | Rhodospirillales | Rhodospirillaceae | unclassified | unclassified |
| Otu014582 | 1 | 0.6667 | 0.816 | 0.01 | Bacteria | Chloroflexi | unclassified | unclassified | unclassified | unclassified | unclassified |
| Otu014788 | 1 | 1 | 1 | 0.001 | Bacteria | SBR1093 | EC214 | unclassified | unclassified | unclassified | unclassified |
| Otu014948 | 0.9552 | 1 | 0.977 | 0.001 | Archaea | Crenarchaeota | Thaumarchaeota | Cenarchaeales | Cenarchaeaceae | Nitrosopumilus | unclassified |
| Otu014966 | 1 | 0.6667 | 0.816 | 0.008 | Bacteria | Chloroflexi | SAR202 | unclassified | unclassified | unclassified | unclassified |
| Otu015061 | 1 | 1 | 1 | 0.001 | Bacteria | unclassified | unclassified | unclassified | unclassified | unclassified | unclassified |
| Otu015087 | 1 | 0.6667 | 0.816 | 0.008 | Bacteria | Chloroflexi | SAR202 | unclassified | unclassified | unclassified | unclassified |
| Otu015093 | 1 | 1 | 1 | 0.001 | Bacteria | Chloroflexi | Anaerolineae | SBR1031 | A4b | unclassified | unclassified |
| Otu015107 | 1 | 1 | 1 | 0.001 | Bacteria | unclassified | unclassified | unclassified | unclassified | unclassified | unclassified |
| Otu015140 | 1 | 0.6667 | 0.816 | 0.01 | Bacteria | unclassified | unclassified | unclassified | unclassified | unclassified | unclassified |
| Otu015153 | 0.9451 | 0.6667 | 0.794 | 0.008 | Archaea | Crenarchaeota | Thaumarchaeota | Cenarchaeales | Cenarchaeaceae | Nitrosopumilus | unclassified |
| Otu015227 | 1 | 1 | 1 | 0.001 | Bacteria | Proteobacteria | Deltaproteobacteria | Bdellovibrionales | Bdellovibrionaceae | Bdellovibrio | unclassified |
| Otu015363 | 1 | 0.6667 | 0.816 | 0.008 | Bacteria | Chloroflexi | SAR202 | unclassified | unclassified | unclassified | unclassified |
| Otu015522 | 1 | 1 | 1 | 0.001 | Bacteria | SBR1093 | EC214 | unclassified | unclassified | unclassified | unclassified |
| Otu015542 | 1 | 0.6667 | 0.816 | 0.008 | Bacteria | Proteobacteria | Gammaproteobacteria | unclassified | unclassified | unclassified | unclassified |
| Otu015656 | 1 | 0.6667 | 0.816 | 0.008 | Bacteria | SBR1093 | EC214 | unclassified | unclassified | unclassified | unclassified |
| Otu015717 | 1 | 1 | 1 | 0.001 | Bacteria | Gemmatimonadetes | Gemm-2 | unclassified | unclassified | unclassified | unclassified |
| Otu015718 | 0.9902 | 1 | 0.995 | 0.001 | Bacteria | Gemmatimonadetes | Gemm-2 | unclassified | unclassified | unclassified | unclassified |
| Otu015726 | 1 | 0.6667 | 0.816 | 0.008 | Bacteria | Chloroflexi | SAR202 | unclassified | unclassified | unclassified | unclassified |
| Otu015776 | 1 | 0.6667 | 0.816 | 0.008 | Bacteria | Chloroflexi | SAR202 | unclassified | unclassified | unclassified | unclassified |
| Otu015854 | 1 | 1 | 1 | 0.001 | Bacteria | Proteobacteria | Alphaproteobacteria | Rhodospirillales | unclassified | unclassified | unclassified |
| Otu015970 | 1 | 1 | 1 | 0.001 | Bacteria | Gemmatimonadetes | Gemm-2 | unclassified | unclassified | unclassified | unclassified |
| Otu015988 | 0.9253 | 1 | 0.962 | 0.002 | Bacteria | Chloroflexi | TK17 | TK18 | unclassified | unclassified | unclassified |
| Otu016131 | 1 | 0.6667 | 0.816 | 0.006 | Bacteria | Chloroflexi | SAR202 | unclassified | unclassified | unclassified | unclassified |
| Otu016137 | 1 | 1 | 1 | 0.001 | Bacteria | Chloroflexi | SAR202 | unclassified | unclassified | unclassified | unclassified |
| Otu016254 | 1 | 1 | 1 | 0.001 | Bacteria | Gemmatimonadetes | Gemm-2 | unclassified | unclassified | unclassified | unclassified |
| Otu016346 | 1 | 0.6667 | 0.816 | 0.008 | Bacteria | Chloroflexi | SAR202 | unclassified | unclassified | unclassified | unclassified |
| Otu016363 | 1 | 1 | 1 | 0.001 | Bacteria | Gemmatimonadetes | Gemm-2 | unclassified | unclassified | unclassified | unclassified |
| Otu016418 | 1 | 0.6667 | 0.816 | 0.008 | Bacteria | Proteobacteria | Alphaproteobacteria | unclassified | unclassified | unclassified | unclassified |
| Otu016544 | 1 | 0.6667 | 0.816 | 0.01 | Bacteria | Proteobacteria | Alphaproteobacteria | unclassified | unclassified | unclassified | unclassified |
| Otu016621 | 1 | 0.6667 | 0.816 | 0.008 | Bacteria | Chloroflexi | SAR202 | unclassified | unclassified | unclassified | unclassified |
| Otu016725 | 1 | 1 | 1 | 0.001 | Bacteria | Proteobacteria | Alphaproteobacteria | unclassified | unclassified | unclassified | unclassified |
| Otu016735 | 1 | 0.6667 | 0.816 | 0.01 | Bacteria | Acidobacteria | Acidobacteria-6 | BPC015 | unclassified | unclassified | unclassified |
| Otu016736 | 1 | 1 | 1 | 0.001 | Bacteria | Acidobacteria | Solibacteres | Solibacterales | PAUC26f | unclassified | unclassified |
| Otu016834 | 0.7886 | 1 | 0.888 | 0.004 | Bacteria | Proteobacteria | Gammaproteobacteria | unclassified | unclassified | unclassified | unclassified |
| Otu016876 | 0.9523 | 0.6667 | 0.797 | 0.006 | Bacteria | Proteobacteria | Gammaproteobacteria | unclassified | unclassified | unclassified | unclassified |
| Otu016987 | 1 | 1 | 1 | 0.001 | Bacteria | Proteobacteria | Gammaproteobacteria | unclassified | unclassified | unclassified | unclassified |
| Otu017006 | 1 | 1 | 1 | 0.001 | Bacteria | Proteobacteria | Gammaproteobacteria | unclassified | unclassified | unclassified | unclassified |
| Otu017043 | 1 | 0.6667 | 0.816 | 0.01 | Bacteria | Proteobacteria | Gammaproteobacteria | Thiotrichales | Piscirickettsiaceae | unclassified | unclassified |
| Otu017063 | 1 | 0.6667 | 0.816 | 0.008 | Bacteria | Proteobacteria | Gammaproteobacteria | unclassified | unclassified | unclassified | unclassified |
| Otu017136 | 1 | 1 | 1 | 0.001 | Bacteria | Proteobacteria | Gammaproteobacteria | unclassified | unclassified | unclassified | unclassified |
| Otu017229 | 1 | 1 | 1 | 0.001 | Bacteria | Acidobacteria | Acidobacteria-6 | iii1-15 | unclassified | unclassified | unclassified |
| Otu017274 | 1 | 1 | 1 | 0.001 | Bacteria | Chloroflexi | Anaerolineae | Caldilineales | Caldilineaceae | unclassified | unclassified |
| Otu017359 | 1 | 1 | 1 | 0.001 | Bacteria | Proteobacteria | Deltaproteobacteria | Syntrophobacterales | Syntrophobacteraceae | unclassified | unclassified |
| Otu017415 | 1 | 1 | 1 | 0.001 | Bacteria | unclassified | unclassified | unclassified | unclassified | unclassified | unclassified |
| Otu017418 | 1 | 1 | 1 | 0.001 | Bacteria | Gemmatimonadetes | Gemm-2 | unclassified | unclassified | unclassified | unclassified |
| Otu017421 | 1 | 1 | 1 | 0.001 | Bacteria | Gemmatimonadetes | Gemm-2 | unclassified | unclassified | unclassified | unclassified |
| Otu017424 | 1 | 1 | 1 | 0.001 | Bacteria | Chloroflexi | SAR202 | unclassified | unclassified | unclassified | unclassified |
| Otu017430 | 1 | 1 | 1 | 0.001 | Bacteria | Acidobacteria | BPC102 | B110 | unclassified | unclassified | unclassified |
| Otu017564 | 1 | 1 | 1 | 0.001 | Bacteria | Proteobacteria | Gammaproteobacteria | unclassified | unclassified | unclassified | unclassified |
| Otu017900 | 1 | 1 | 1 | 0.001 | Bacteria | Proteobacteria | Gammaproteobacteria | unclassified | unclassified | unclassified | unclassified |
| Otu017903 | 1 | 1 | 1 | 0.001 | Bacteria | Proteobacteria | Gammaproteobacteria | unclassified | unclassified | unclassified | unclassified |
| Otu017912 | 1 | 1 | 1 | 0.001 | Bacteria | Proteobacteria | Gammaproteobacteria | unclassified | unclassified | unclassified | unclassified |
| Otu017937 | 1 | 1 | 1 | 0.001 | Bacteria | Proteobacteria | Gammaproteobacteria | unclassified | unclassified | unclassified | unclassified |
| Otu017956 | 1 | 1 | 1 | 0.001 | Bacteria | Proteobacteria | Gammaproteobacteria | unclassified | unclassified | unclassified | unclassified |
| Otu018057 | 1 | 1 | 1 | 0.001 | Bacteria | AncK6 | unclassified | unclassified | unclassified | unclassified | unclassified |
| Otu018238 | 1 | 0.6667 | 0.816 | 0.008 | Bacteria | Proteobacteria | Gammaproteobacteria | unclassified | unclassified | unclassified | unclassified |
| Otu018302 | 1 | 1 | 1 | 0.001 | Bacteria | Chloroflexi | SAR202 | unclassified | unclassified | unclassified | unclassified |
| Otu018501 | 1 | 0.6667 | 0.816 | 0.01 | Bacteria | Chloroflexi | SAR202 | unclassified | unclassified | unclassified | unclassified |
| Otu018526 | 1 | 0.6667 | 0.816 | 0.01 | Bacteria | Acidobacteria | PAUC37f | unclassified | unclassified | unclassified | unclassified |
| Otu018572 | 1 | 1 | 1 | 0.001 | Bacteria | SBR1093 | EC214 | unclassified | unclassified | unclassified | unclassified |
| Otu018619 | 0.934 | 0.6667 | 0.789 | 0.029 | Bacteria | SBR1093 | EC214 | unclassified | unclassified | unclassified | unclassified |
| Otu018749 | 1 | 0.6667 | 0.816 | 0.01 | Bacteria | Proteobacteria | unclassified | unclassified | unclassified | unclassified | unclassified |
| Otu018925 | 1 | 0.6667 | 0.816 | 0.006 | Archaea | Crenarchaeota | Thaumarchaeota | Cenarchaeales | Cenarchaeaceae | Nitrosopumilus | unclassified |
| Otu019007 | 1 | 1 | 1 | 0.001 | Bacteria | Proteobacteria | Deltaproteobacteria | Syntrophobacterales | Syntrophobacteraceae | unclassified | unclassified |
| Otu019184 | 1 | 1 | 1 | 0.001 | Bacteria | Chloroflexi | SAR202 | unclassified | unclassified | unclassified | unclassified |
| Otu019253 | 0.9318 | 1 | 0.965 | 0.001 | Bacteria | Chloroflexi | unclassified | unclassified | unclassified | unclassified | unclassified |
| Otu019270 | 1 | 0.6667 | 0.816 | 0.006 | Archaea | Crenarchaeota | Thaumarchaeota | Cenarchaeales | Cenarchaeaceae | Nitrosopumilus | unclassified |
| Otu019282 | 1 | 1 | 1 | 0.001 | Bacteria | Chloroflexi | unclassified | unclassified | unclassified | unclassified | unclassified |

| | | | | | | | | | | | | |
|---|---|---|---|---|---|---|---|---|---|---|---|---|
| Otu019326 | 1 | 0.6667 | 0.816 | 0.01 | Bacteria | Actinobacteria | Acidimicrobiia | Acidimicrobiales | koll13 | unclassified | unclassified |
| Otu019592 | 0.8604 | 1 | 0.928 | 0.003 | Archaea | Crenarchaeota | Thaumarchaeota | Cenarchaeales | Cenarchaeaceae | Nitrosopumilus | unclassified |
| Otu019620 | 1 | 0.6667 | 0.816 | 0.008 | Bacteria | Proteobacteria | Gammaproteobacteria | unclassified | unclassified | unclassified | unclassified |
| Otu019696 | 0.896 | 1 | 0.947 | 0.002 | Bacteria | Proteobacteria | Gammaproteobacteria | unclassified | unclassified | unclassified | unclassified |
| Otu019708 | 0.9838 | 1 | 0.992 | 0.001 | Bacteria | Proteobacteria | Gammaproteobacteria | HTCC2188 | HTCC2089 | unclassified | unclassified |
| Otu020239 | 0.9233 | 0.6667 | 0.785 | 0.027 | Bacteria | Actinobacteria | Acidimicrobiia | Acidimicrobiales | wb1_P06 | unclassified | unclassified |
| Otu020247 | 1 | 0.6667 | 0.816 | 0.006 | Bacteria | Actinobacteria | Acidimicrobiia | Acidimicrobiales | wb1_P06 | unclassified | unclassified |
| Otu020279 | 0.9255 | 1 | 0.962 | 0.002 | Bacteria | Actinobacteria | Acidimicrobiia | Acidimicrobiales | wb1_P06 | unclassified | unclassified |
| Otu020420 | 1 | 1 | 1 | 0.001 | Bacteria | Proteobacteria | Gammaproteobacteria | unclassified | unclassified | unclassified | unclassified |
| Otu020429 | 1 | 0.6667 | 0.816 | 0.008 | Bacteria | SBR1093 | EC214 | unclassified | unclassified | unclassified | unclassified |
| Otu020867 | 1 | 0.6667 | 0.816 | 0.008 | Bacteria | SBR1093 | EC214 | unclassified | unclassified | unclassified | unclassified |
| Otu020893 | 1 | 0.6667 | 0.816 | 0.01 | Bacteria | Proteobacteria | Deltaproteobacteria | Bdellovibrionales | Bdellovibrionaceae | Bdellovibrio | unclassified |
| Otu021184 | 1 | 1 | 1 | 0.001 | Bacteria | unclassified | unclassified | unclassified | unclassified | unclassified | unclassified |
| Otu021249 | 1 | 1 | 1 | 0.001 | Bacteria | Acidobacteria | BPC102 | B110 | unclassified | unclassified | unclassified |
| Otu021347 | 1 | 0.6667 | 0.816 | 0.01 | Bacteria | Gemmatimonadetes | Gemm-2 | unclassified | unclassified | unclassified | unclassified |
| Otu021446 | 1 | 1 | 1 | 0.001 | Bacteria | Actinobacteria | Acidimicrobiia | Acidimicrobiales | TK06 | unclassified | unclassified |
| Otu021474 | 1 | 1 | 1 | 0.001 | Bacteria | Chloroflexi | Anaerolineae | Caldilineales | Caldilineaceae | unclassified | unclassified |
| Otu021487 | 1 | 0.6667 | 0.816 | 0.01 | Bacteria | Chloroflexi | TK17 | TK18 | unclassified | unclassified | unclassified |
| Otu021542 | 1 | 1 | 1 | 0.001 | Bacteria | Acidobacteria | unclassified | unclassified | unclassified | unclassified | unclassified |
| Otu021732 | 1 | 0.6667 | 0.816 | 0.01 | Bacteria | Bacteroidetes | Rhodothermi | Rhodothermales | Rhodothermaceae | unclassified | unclassified |
| Otu021844 | 1 | 0.6667 | 0.816 | 0.006 | Bacteria | unclassified | unclassified | unclassified | unclassified | unclassified | unclassified |
| Otu022185 | 0.9026 | 1 | 0.95 | 0.002 | Bacteria | PAUC34f | unclassified | unclassified | unclassified | unclassified | unclassified |
| Otu022652 | 1 | 1 | 1 | 0.001 | Bacteria | Acidobacteria | Acidobacteria-6 | iii1-15 | unclassified | unclassified | unclassified |
| Otu022745 | 1 | 0.6667 | 0.816 | 0.008 | Bacteria | Proteobacteria | Gammaproteobacteria | unclassified | unclassified | unclassified | unclassified |
| Otu022951 | 0.9905 | 1 | 0.995 | 0.001 | Bacteria | unclassified | unclassified | unclassified | unclassified | unclassified | unclassified |
| Otu023058 | 1 | 1 | 1 | 0.001 | Bacteria | Proteobacteria | Gammaproteobacteria | HTCC2188 | HTCC2089 | unclassified | unclassified |
| Otu023235 | 1 | 1 | 1 | 0.001 | Bacteria | Acidobacteria | BPC102 | B110 | unclassified | unclassified | unclassified |
| Otu023462 | 1 | 1 | 1 | 0.001 | Bacteria | Gemmatimonadetes | Gemm-2 | unclassified | unclassified | unclassified | unclassified |
| Otu023547 | 1 | 1 | 1 | 0.001 | Bacteria | unclassified | unclassified | unclassified | unclassified | unclassified | unclassified |
| Otu023568 | 0.9278 | 0.6667 | 0.786 | 0.032 | Bacteria | Chloroflexi | SAR202 | unclassified | unclassified | unclassified | unclassified |
| Otu023573 | 1 | 0.6667 | 0.816 | 0.008 | Bacteria | Nitrospirae | Nitrospira | Nitrospirales | Nitrospiraceae | unclassified | unclassified |
| Otu023859 | 0.9722 | 1 | 0.986 | 0.001 | Bacteria | Proteobacteria | Alphaproteobacteria | unclassified | unclassified | unclassified | unclassified |
| Otu023860 | 0.9893 | 1 | 0.995 | 0.001 | Bacteria | Proteobacteria | Alphaproteobacteria | unclassified | unclassified | unclassified | unclassified |
| Otu024193 | 1 | 1 | 1 | 0.001 | Bacteria | Chloroflexi | SAR202 | unclassified | unclassified | unclassified | unclassified |
| Otu024256 | 1 | 1 | 1 | 0.001 | Bacteria | Chloroflexi | SAR202 | unclassified | unclassified | unclassified | unclassified |
| Otu024409 | 1 | 0.6667 | 0.816 | 0.01 | Bacteria | Proteobacteria | Alphaproteobacteria | unclassified | unclassified | unclassified | unclassified |
| Otu024436 | 1 | 1 | 1 | 0.001 | Bacteria | Proteobacteria | Alphaproteobacteria | unclassified | unclassified | unclassified | unclassified |
| Otu024538 | 1 | 0.6667 | 0.816 | 0.008 | Bacteria | Proteobacteria | Gammaproteobacteria | HTCC2188 | HTCC2089 | unclassified | unclassified |
| Otu024696 | 1 | 1 | 1 | 0.001 | Bacteria | Acidobacteria | BPC102 | B110 | unclassified | unclassified | unclassified |
| Otu024698 | 1 | 1 | 1 | 0.001 | Bacteria | Acidobacteria | Acidobacteria-6 | BPC015 | unclassified | unclassified | unclassified |
| Otu024743 | 1 | 0.6667 | 0.816 | 0.008 | Bacteria | Chloroflexi | SAR202 | unclassified | unclassified | unclassified | unclassified |
| Otu024999 | 1 | 1 | 1 | 0.001 | Bacteria | Chloroflexi | unclassified | unclassified | unclassified | unclassified | unclassified |
| Otu025002 | 1 | 1 | 1 | 0.001 | Bacteria | Nitrospirae | Nitrospira | Nitrospirales | Nitrospiraceae | unclassified | unclassified |
| Otu025434 | 1 | 0.6667 | 0.816 | 0.01 | Bacteria | Proteobacteria | Gammaproteobacteria | Thiotrichales | Piscirickettsiaceae | unclassified | unclassified |
| Otu025444 | 1 | 1 | 1 | 0.001 | Bacteria | Acidobacteria | Solibacteres | Solibacterales | PAUC26f | unclassified | unclassified |
| Otu025446 | 1 | 0.6667 | 0.816 | 0.01 | Bacteria | unclassified | unclassified | unclassified | unclassified | unclassified | unclassified |
| Otu025486 | 1 | 1 | 1 | 0.001 | Bacteria | Acidobacteria | Acidobacteria-5 | unclassified | unclassified | unclassified | unclassified |
| Otu025489 | 0.9899 | 1 | 0.995 | 0.001 | Bacteria | Acidobacteria | PAUC37f | unclassified | unclassified | unclassified | unclassified |
| Otu025515 | 1 | 0.6667 | 0.816 | 0.006 | Bacteria | SBR1093 | EC214 | unclassified | unclassified | unclassified | unclassified |
| Otu025567 | 1 | 0.6667 | 0.816 | 0.008 | Bacteria | SBR1093 | EC214 | unclassified | unclassified | unclassified | unclassified |
| Otu025591 | 1 | 0.6667 | 0.816 | 0.01 | Bacteria | Chloroflexi | SAR202 | unclassified | unclassified | unclassified | unclassified |
| Otu025595 | 1 | 1 | 1 | 0.001 | Bacteria | Chloroflexi | SAR202 | unclassified | unclassified | unclassified | unclassified |
| Otu025626 | 1 | 1 | 1 | 0.001 | Bacteria | Chloroflexi | SAR202 | unclassified | unclassified | unclassified | unclassified |
| Otu025646 | 1 | 0.6667 | 0.816 | 0.01 | Bacteria | Chloroflexi | SAR202 | unclassified | unclassified | unclassified | unclassified |
| Otu025678 | 0.9479 | 1 | 0.974 | 0.002 | Bacteria | unclassified | unclassified | unclassified | unclassified | unclassified | unclassified |
| Otu026006 | 1 | 1 | 1 | 0.001 | Bacteria | Proteobacteria | Deltaproteobacteria | Syntrophobacterales | Syntrophobacteraceae | unclassified | unclassified |
| Otu026396 | 0.9891 | 1 | 0.995 | 0.001 | Bacteria | Nitrospirae | Nitrospira | Nitrospirales | Nitrospiraceae | unclassified | unclassified |
| Otu026419 | 1 | 0.6667 | 0.816 | 0.01 | Bacteria | Proteobacteria | Deltaproteobacteria | Bdellovibrionales | Bdellovibrionaceae | Bdellovibrio | unclassified |
| Otu026421 | 1 | 1 | 1 | 0.001 | Bacteria | Acidobacteria | BPC102 | B110 | unclassified | unclassified | unclassified |
| Otu026600 | 1 | 1 | 1 | 0.001 | Bacteria | Chloroflexi | TK17 | mle1-48 | unclassified | unclassified | unclassified |
| Otu026731 | 1 | 1 | 1 | 0.001 | Archaea | Crenarchaeota | Thaumarchaeota | Cenarchaeales | Cenarchaeaceae | unclassified | unclassified |
| Otu026860 | 0.9314 | 0.6667 | 0.788 | 0.021 | Bacteria | SBR1093 | EC214 | unclassified | unclassified | unclassified | unclassified |
| Otu026912 | 1 | 1 | 1 | 0.001 | Archaea | Crenarchaeota | Thaumarchaeota | Cenarchaeales | Cenarchaeaceae | unclassified | unclassified |
| Otu026913 | 0.8912 | 1 | 0.944 | 0.003 | Bacteria | Chloroflexi | SAR202 | unclassified | unclassified | unclassified | unclassified |
| Otu027219 | 0.9549 | 1 | 0.977 | 0.001 | Bacteria | SBR1093 | EC214 | unclassified | unclassified | unclassified | unclassified |
| Otu027239 | 0.8741 | 0.6667 | 0.763 | 0.035 | Bacteria | SBR1093 | EC214 | unclassified | unclassified | unclassified | unclassified |
| Otu027374 | 1 | 0.6667 | 0.816 | 0.01 | Bacteria | Acidobacteria | BPC102 | B110 | unclassified | unclassified | unclassified |
| Otu027600 | 1 | 0.6667 | 0.816 | 0.006 | Bacteria | Proteobacteria | unclassified | unclassified | unclassified | unclassified | unclassified |
| Otu027719 | 1 | 1 | 1 | 0.001 | Bacteria | Gemmatimonadetes | Gemm-2 | unclassified | unclassified | unclassified | unclassified |
| Otu027856 | 1 | 1 | 1 | 0.001 | Bacteria | Acidobacteria | BPC102 | B110 | unclassified | unclassified | unclassified |
| Otu027869 | 1 | 1 | 1 | 0.001 | Bacteria | Chloroflexi | Ktedonobacteria | TK10 | unclassified | unclassified | unclassified |
| Otu028013 | 1 | 1 | 1 | 0.001 | Bacteria | Proteobacteria | Deltaproteobacteria | Bdellovibrionales | Bdellovibrionaceae | Bdellovibrio | unclassified |
| Otu028068 | 1 | 1 | 1 | 0.001 | Bacteria | Poribacteria | unclassified | unclassified | unclassified | unclassified | unclassified |
| Otu028164 | 1 | 0.6667 | 0.816 | 0.01 | Bacteria | Nitrospirae | Nitrospira | Nitrospirales | Nitrospiraceae | unclassified | unclassified |
| Otu028178 | 1 | 0.6667 | 0.816 | 0.01 | Bacteria | Chloroflexi | SAR202 | unclassified | unclassified | unclassified | unclassified |
| Otu028265 | 1 | 0.6667 | 0.816 | 0.01 | Bacteria | Nitrospirae | Nitrospira | Nitrospirales | Nitrospiraceae | unclassified | unclassified |
| Otu028376 | 1 | 1 | 1 | 0.001 | Bacteria | Acidobacteria | Solibacteres | Solibacterales | PAUC26f | unclassified | unclassified |
| Otu028396 | 1 | 0.6667 | 0.816 | 0.01 | Bacteria | Chloroflexi | SAR202 | unclassified | unclassified | unclassified | unclassified |
| Otu028475 | 1 | 1 | 1 | 0.001 | Bacteria | Chloroflexi | SAR202 | unclassified | unclassified | unclassified | unclassified |
| Otu028757 | 1 | 1 | 1 | 0.001 | Bacteria | Chloroflexi | Anaerolineae | unclassified | unclassified | unclassified | unclassified |
| Otu028769 | 1 | 0.6667 | 0.816 | 0.006 | Bacteria | Chloroflexi | unclassified | unclassified | unclassified | unclassified | unclassified |
| Otu028814 | 1 | 1 | 1 | 0.001 | Bacteria | Chloroflexi | Anaerolineae | SBR1031 | A4b | unclassified | unclassified |
| Otu028876 | 1 | 0.6667 | 0.816 | 0.006 | Bacteria | SBR1093 | EC214 | unclassified | unclassified | unclassified | unclassified |
| Otu028882 | 1 | 0.6667 | 0.816 | 0.006 | Bacteria | Actinobacteria | unclassified | unclassified | unclassified | unclassified | unclassified |
| Otu029034 | 1 | 0.6667 | 0.816 | 0.01 | Bacteria | Proteobacteria | Gammaproteobacteria | unclassified | unclassified | unclassified | unclassified |
| Otu029889 | 1 | 0.6667 | 0.816 | 0.01 | Bacteria | PAUC34f | unclassified | unclassified | unclassified | unclassified | unclassified |
| Otu029896 | 1 | 0.6667 | 0.816 | 0.01 | Bacteria | PAUC34f | unclassified | unclassified | unclassified | unclassified | unclassified |
| Otu029927 | 0.9451 | 1 | 0.972 | 0.002 | Bacteria | PAUC34f | unclassified | unclassified | unclassified | unclassified | unclassified |
| Otu029952 | 1 | 0.6667 | 0.816 | 0.008 | Bacteria | PAUC34f | unclassified | unclassified | unclassified | unclassified | unclassified |
| Otu030239 | 1 | 0.6667 | 0.816 | 0.01 | Bacteria | PAUC34f | unclassified | unclassified | unclassified | unclassified | unclassified |
| Otu030276 | 1 | 0.6667 | 0.816 | 0.01 | Bacteria | PAUC34f | unclassified | unclassified | unclassified | unclassified | unclassified |
| Otu030472 | 0.9337 | 0.6667 | 0.789 | 0.031 | Bacteria | Proteobacteria | Gammaproteobacteria | unclassified | unclassified | unclassified | unclassified |
| Otu030485 | 1 | 1 | 1 | 0.001 | Bacteria | Proteobacteria | Gammaproteobacteria | unclassified | unclassified | unclassified | unclassified |
| Otu030505 | 1 | 0.6667 | 0.816 | 0.01 | Bacteria | Proteobacteria | Gammaproteobacteria | unclassified | unclassified | unclassified | unclassified |
| Otu030765 | 1 | 1 | 1 | 0.001 | Bacteria | Proteobacteria | Gammaproteobacteria | unclassified | unclassified | unclassified | unclassified |
| Otu030780 | 1 | 0.6667 | 0.816 | 0.006 | Bacteria | Chloroflexi | SAR202 | unclassified | unclassified | unclassified | unclassified |
| Otu030895 | 1 | 1 | 1 | 0.001 | Bacteria | unclassified | unclassified | unclassified | unclassified | unclassified | unclassified |
| Otu031795 | 1 | 1 | 1 | 0.001 | Bacteria | Proteobacteria | Gammaproteobacteria | unclassified | unclassified | unclassified | unclassified |
| Otu032171 | 1 | 1 | 1 | 0.001 | Bacteria | Proteobacteria | Gammaproteobacteria | unclassified | unclassified | unclassified | unclassified |
| Otu032207 | 1 | 1 | 1 | 0.001 | Bacteria | Gemmatimonadetes | Gemm-2 | unclassified | unclassified | unclassified | unclassified |

| | | | | | | | | | | | |
|---|---|---|---|---|---|---|---|---|---|---|---|
| Otu032449 | 1 | 0.6667 | 0.816 | 0.01 | Bacteria | Gemmatimonadetes | Gemm-4 | unclassified | unclassified | unclassified | unclassified |
| Otu032524 | 1 | 1 | 1 | 0.001 | Bacteria | Chloroflexi | SAR202 | unclassified | unclassified | unclassified | unclassified |
| Otu032705 | 1 | 1 | 1 | 0.001 | Bacteria | Chloroflexi | SAR202 | unclassified | unclassified | unclassified | unclassified |
| Otu032733 | 0.9948 | 1 | 0.997 | 0.001 | Bacteria | Chloroflexi | SAR202 | unclassified | unclassified | unclassified | unclassified |
| Otu032792 | 1 | 0.6667 | 0.816 | 0.01 | Bacteria | Acidobacteria | Acidobacteria-6 | iii1-15 | unclassified | unclassified | unclassified |
| Otu032807 | 1 | 1 | 1 | 0.001 | Bacteria | Acidobacteria | Acidobacteria-6 | iii1-15 | unclassified | unclassified | unclassified |
| Otu032812 | 1 | 0.6667 | 0.816 | 0.006 | Bacteria | Acidobacteria | PAUC37f | unclassified | unclassified | unclassified | unclassified |
| Otu033030 | 1 | 1 | 1 | 0.001 | Bacteria | Chloroflexi | SAR202 | unclassified | unclassified | unclassified | unclassified |
| Otu033511 | 0.9877 | 1 | 0.994 | 0.001 | Bacteria | Proteobacteria | Alphaproteobacteria | Rhodospirillales | Rhodospirillaceae | unclassified | unclassified |
| Otu033899 | 1 | 1 | 1 | 0.001 | Bacteria | Proteobacteria | Alphaproteobacteria | unclassified | unclassified | unclassified | unclassified |
| Otu034261 | 1 | 1 | 1 | 0.001 | Bacteria | Proteobacteria | Alphaproteobacteria | unclassified | unclassified | unclassified | unclassified |
| Otu034313 | 1 | 1 | 1 | 0.001 | Bacteria | Proteobacteria | Alphaproteobacteria | Rhodospirillales | unclassified | unclassified | unclassified |
| Otu034343 | 1 | 0.6667 | 0.816 | 0.01 | Bacteria | Proteobacteria | Alphaproteobacteria | unclassified | unclassified | unclassified | unclassified |
| Otu034355 | 1 | 1 | 1 | 0.001 | Bacteria | Proteobacteria | Alphaproteobacteria | unclassified | unclassified | unclassified | unclassified |
| Otu034620 | 1 | 1 | 1 | 0.001 | Bacteria | Proteobacteria | Alphaproteobacteria | unclassified | unclassified | unclassified | unclassified |
| Otu034801 | 1 | 0.6667 | 0.816 | 0.01 | Bacteria | Actinobacteria | Acidimicrobiia | Acidimicrobiales | wb1_P06 | unclassified | unclassified |
| Otu034804 | 1 | 0.6667 | 0.816 | 0.008 | Bacteria | Actinobacteria | Acidimicrobiia | Acidimicrobiales | wb1_P06 | unclassified | unclassified |
| Otu034897 | 1 | 1 | 1 | 0.001 | Bacteria | Chloroflexi | SAR202 | unclassified | unclassified | unclassified | unclassified |
| Otu035239 | 1 | 0.6667 | 0.816 | 0.01 | Bacteria | Chloroflexi | SAR202 | unclassified | unclassified | unclassified | unclassified |
| Otu035809 | 1 | 0.6667 | 0.816 | 0.006 | Bacteria | Proteobacteria | Gammaproteobacteria | unclassified | unclassified | unclassified | unclassified |
| Otu035890 | 1 | 1 | 1 | 0.001 | Bacteria | Acidobacteria | unclassified | unclassified | unclassified | unclassified | unclassified |
| Otu036143 | 1 | 0.6667 | 0.816 | 0.006 | Bacteria | unclassified | unclassified | unclassified | unclassified | unclassified | unclassified |
| Otu036511 | 1 | 1 | 1 | 0.001 | Bacteria | SBR1093 | EC214 | unclassified | unclassified | unclassified | unclassified |
| Otu036849 | 1 | 0.6667 | 0.816 | 0.006 | Bacteria | Acidobacteria | Acidobacteria-6 | BPC015 | unclassified | unclassified | unclassified |
| Otu037081 | 1 | 0.6667 | 0.816 | 0.01 | Bacteria | Chloroflexi | SAR202 | unclassified | unclassified | unclassified | unclassified |
| Otu037141 | 1 | 1 | 1 | 0.001 | Archaea | Crenarchaeota | Thaumarchaeota | Cenarchaeales | Cenarchaeaceae | unclassified | unclassified |
| Otu037587 | 1 | 0.6667 | 0.816 | 0.006 | Bacteria | unclassified | unclassified | unclassified | unclassified | unclassified | unclassified |
| Otu037639 | 1 | 0.6667 | 0.816 | 0.006 | Bacteria | Actinobacteria | Acidimicrobiia | Acidimicrobiales | wb1_P06 | unclassified | unclassified |
| Otu038038 | 1 | 0.6667 | 0.816 | 0.01 | Bacteria | Actinobacteria | Acidimicrobiia | Acidimicrobiales | wb1_P06 | unclassified | unclassified |
| Otu038356 | 1 | 0.6667 | 0.816 | 0.01 | Bacteria | Actinobacteria | Acidimicrobiia | Acidimicrobiales | koll13 | unclassified | unclassified |
| Otu038538 | 1 | 0.6667 | 0.816 | 0.008 | Bacteria | Proteobacteria | Gammaproteobacteria | unclassified | unclassified | unclassified | unclassified |
| Otu039735 | 1 | 0.6667 | 0.816 | 0.006 | Bacteria | Nitrospirae | Nitrospira | Nitrospirales | Nitrospiraceae | unclassified | unclassified |
| Otu040190 | 1 | 1 | 1 | 0.001 | Archaea | Crenarchaeota | Thaumarchaeota | Cenarchaeales | Cenarchaeaceae | unclassified | unclassified |
| Otu041752 | 1 | 0.6667 | 0.816 | 0.006 | Bacteria | Proteobacteria | Gammaproteobacteria | unclassified | unclassified | unclassified | unclassified |
| Otu042715 | 1 | 1 | 1 | 0.001 | Archaea | Crenarchaeota | Thaumarchaeota | Cenarchaeales | Cenarchaeaceae | unclassified | unclassified |
| Otu043864 | 1 | 0.6667 | 0.816 | 0.01 | Bacteria | SBR1093 | EC214 | unclassified | unclassified | unclassified | unclassified |
| Otu044635 | 1 | 0.6667 | 0.816 | 0.01 | Bacteria | Proteobacteria | unclassified | unclassified | unclassified | unclassified | unclassified |
| Otu045735 | 1 | 0.6667 | 0.816 | 0.01 | Bacteria | Gemmatimonadetes | Gemm-2 | unclassified | unclassified | unclassified | unclassified |
| Otu045881 | 1 | 0.6667 | 0.816 | 0.006 | Bacteria | unclassified | unclassified | unclassified | unclassified | unclassified | unclassified |
| Otu046200 | 1 | 1 | 1 | 0.001 | Bacteria | Chloroflexi | SAR202 | unclassified | unclassified | unclassified | unclassified |
| Otu046379 | 1 | 1 | 1 | 0.001 | Bacteria | Chloroflexi | unclassified | unclassified | unclassified | unclassified | unclassified |
| Otu046468 | 0.9221 | 0.6667 | 0.784 | 0.029 | Bacteria | AncK6 | unclassified | unclassified | unclassified | unclassified | unclassified |
| Otu047109 | 1 | 1 | 1 | 0.001 | Archaea | Crenarchaeota | Thaumarchaeota | Cenarchaeales | Cenarchaeaceae | unclassified | unclassified |
| Otu047906 | 1 | 1 | 1 | 0.001 | Archaea | Crenarchaeota | Thaumarchaeota | Cenarchaeales | Cenarchaeaceae | unclassified | unclassified |
| Otu047947 | 1 | 0.6667 | 0.816 | 0.006 | Bacteria | Chloroflexi | unclassified | unclassified | unclassified | unclassified | unclassified |
| Otu047995 | 1 | 1 | 1 | 0.001 | Bacteria | Nitrospirae | Nitrospira | Nitrospirales | Nitrospiraceae | unclassified | unclassified |
| Otu048113 | 1 | 0.6667 | 0.816 | 0.008 | Bacteria | SBR1093 | EC214 | unclassified | unclassified | unclassified | unclassified |
| Otu048443 | 1 | 0.6667 | 0.816 | 0.006 | Bacteria | SBR1093 | EC214 | unclassified | unclassified | unclassified | unclassified |
| Otu049062 | 1 | 1 | 1 | 0.001 | Archaea | Crenarchaeota | Thaumarchaeota | Cenarchaeales | Cenarchaeaceae | unclassified | unclassified |
| Otu049315 | 1 | 1 | 1 | 0.001 | Bacteria | Chloroflexi | Anaerolineae | unclassified | unclassified | unclassified | unclassified |
| Otu050236 | 1 | 1 | 1 | 0.001 | Bacteria | Proteobacteria | Gammaproteobacteria | unclassified | unclassified | unclassified | unclassified |
| Otu050799 | 1 | 0.6667 | 0.816 | 0.01 | Bacteria | Proteobacteria | Gammaproteobacteria | Thiotrichales | Piscirickettsiaceae | unclassified | unclassified |
| Otu052518 | 1 | 1 | 1 | 0.001 | Bacteria | Gemmatimonadetes | Gemm-2 | unclassified | unclassified | unclassified | unclassified |
| Otu052543 | 1 | 0.6667 | 0.816 | 0.01 | Bacteria | unclassified | unclassified | unclassified | unclassified | unclassified | unclassified |
| Otu052785 | 1 | 0.6667 | 0.816 | 0.008 | Bacteria | Proteobacteria | Gammaproteobacteria | unclassified | unclassified | unclassified | unclassified |
| Otu054016 | 1 | 1 | 1 | 0.001 | Bacteria | Proteobacteria | Gammaproteobacteria | unclassified | unclassified | unclassified | unclassified |
| Otu054245 | 1 | 0.6667 | 0.816 | 0.006 | Bacteria | Proteobacteria | Gammaproteobacteria | unclassified | unclassified | unclassified | unclassified |
| Otu054429 | 1 | 1 | 1 | 0.001 | Bacteria | Acidobacteria | Acidobacteria-6 | iii1-15 | unclassified | unclassified | unclassified |
| Otu054439 | 1 | 1 | 1 | 0.001 | Bacteria | unclassified | unclassified | unclassified | unclassified | unclassified | unclassified |
| Otu054478 | 0.9685 | 1 | 0.984 | 0.002 | Bacteria | Acidobacteria | Acidobacteria-6 | iii1-15 | unclassified | unclassified | unclassified |
| Otu054556 | 1 | 1 | 1 | 0.001 | Bacteria | Chloroflexi | Anaerolineae | SBR1031 | A4b | unclassified | unclassified |
| Otu054641 | 0.9233 | 0.6667 | 0.785 | 0.027 | Bacteria | Chloroflexi | SAR202 | unclassified | unclassified | unclassified | unclassified |
| Otu054967 | 1 | 1 | 1 | 0.001 | Bacteria | Chloroflexi | SAR202 | unclassified | unclassified | unclassified | unclassified |
| Otu055025 | 0.9221 | 0.6667 | 0.784 | 0.029 | Bacteria | Chloroflexi | SAR202 | unclassified | unclassified | unclassified | unclassified |
| Otu055113 | 0.934 | 0.6667 | 0.789 | 0.029 | Bacteria | Chloroflexi | SAR202 | unclassified | unclassified | unclassified | unclassified |
| Otu055538 | 1 | 1 | 1 | 0.001 | Bacteria | Chloroflexi | SAR202 | unclassified | unclassified | unclassified | unclassified |
| Otu055916 | 1 | 1 | 1 | 0.001 | Bacteria | Proteobacteria | Alphaproteobacteria | unclassified | unclassified | unclassified | unclassified |
| Otu057124 | 1 | 0.6667 | 0.816 | 0.01 | Bacteria | Proteobacteria | Gammaproteobacteria | unclassified | unclassified | unclassified | unclassified |
| Otu057437 | 1 | 0.6667 | 0.816 | 0.01 | Bacteria | Proteobacteria | Gammaproteobacteria | unclassified | unclassified | unclassified | unclassified |
| Otu058168 | 1 | 1 | 1 | 0.001 | Bacteria | Acidobacteria | Acidobacteria-6 | iii1-15 | unclassified | unclassified | unclassified |
| Otu058175 | 1 | 0.6667 | 0.816 | 0.01 | Bacteria | Chloroflexi | SAR202 | unclassified | unclassified | unclassified | unclassified |
| Otu058178 | 1 | 1 | 1 | 0.001 | Bacteria | Acidobacteria | Acidobacteria-6 | iii1-15 | unclassified | unclassified | unclassified |
| Otu058364 | 1 | 1 | 1 | 0.001 | Bacteria | Acidobacteria | Acidobacteria-5 | unclassified | unclassified | unclassified | unclassified |
| Otu058369 | 1 | 0.6667 | 0.816 | 0.01 | Bacteria | Acidobacteria | PAUC37f | unclassified | unclassified | unclassified | unclassified |
| Otu058683 | 1 | 0.6667 | 0.816 | 0.01 | Bacteria | Chloroflexi | SAR202 | unclassified | unclassified | unclassified | unclassified |
| Otu058746 | 1 | 1 | 1 | 0.001 | Bacteria | Proteobacteria | Gammaproteobacteria | unclassified | unclassified | unclassified | unclassified |
| Otu059092 | 1 | 1 | 1 | 0.001 | Bacteria | Proteobacteria | Alphaproteobacteria | Rhodospirillales | Rhodospirillaceae | unclassified | unclassified |
| Otu059116 | 1 | 1 | 1 | 0.001 | Bacteria | Proteobacteria | Alphaproteobacteria | Rhodospirillales | Rhodospirillaceae | unclassified | unclassified |
| Otu059160 | 0.9214 | 1 | 0.96 | 0.001 | Bacteria | Chloroflexi | SAR202 | unclassified | unclassified | unclassified | unclassified |
| Otu059470 | 1 | 0.6667 | 0.816 | 0.006 | Bacteria | Proteobacteria | Gammaproteobacteria | unclassified | unclassified | unclassified | unclassified |
| Otu060515 | 1 | 1 | 1 | 0.001 | Bacteria | Chloroflexi | SAR202 | unclassified | unclassified | unclassified | unclassified |
| Otu061188 | 1 | 0.6667 | 0.816 | 0.006 | Bacteria | Nitrospirae | Nitrospira | Nitrospirales | Nitrospiraceae | unclassified | unclassified |
| Otu061561 | 1 | 0.6667 | 0.816 | 0.008 | Bacteria | Chloroflexi | SAR202 | unclassified | unclassified | unclassified | unclassified |
| Otu062589 | 1 | 0.6667 | 0.816 | 0.01 | Archaea | Crenarchaeota | Thaumarchaeota | Cenarchaeales | Cenarchaeaceae | unclassified | unclassified |
| Otu062877 | 1 | 0.6667 | 0.816 | 0.01 | Bacteria | Proteobacteria | Gammaproteobacteria | unclassified | unclassified | unclassified | unclassified |
| Otu063863 | 1 | 0.6667 | 0.816 | 0.006 | Bacteria | Acidobacteria | unclassified | unclassified | unclassified | unclassified | unclassified |
| Otu064067 | 1 | 0.6667 | 0.816 | 0.006 | Bacteria | AncK6 | unclassified | unclassified | unclassified | unclassified | unclassified |
| Otu064721 | 1 | 0.6667 | 0.816 | 0.01 | Bacteria | Proteobacteria | Gammaproteobacteria | unclassified | unclassified | unclassified | unclassified |
| Otu065628 | 1 | 1 | 1 | 0.001 | Bacteria | Proteobacteria | Gammaproteobacteria | Chromatiales | unclassified | unclassified | unclassified |
| Otu065915 | 1 | 0.6667 | 0.816 | 0.008 | Bacteria | Proteobacteria | Gammaproteobacteria | unclassified | unclassified | unclassified | unclassified |
| Otu066353 | 1 | 1 | 1 | 0.001 | Bacteria | Proteobacteria | Gammaproteobacteria | Chromatiales | unclassified | unclassified | unclassified |
| Otu066808 | 1 | 1 | 1 | 0.001 | Bacteria | PAUC34f | unclassified | unclassified | unclassified | unclassified | unclassified |
| Otu066834 | 1 | 0.6667 | 0.816 | 0.01 | Bacteria | PAUC34f | unclassified | unclassified | unclassified | unclassified | unclassified |
| Otu066915 | 0.9249 | 0.6667 | 0.785 | 0.022 | Bacteria | Proteobacteria | Gammaproteobacteria | unclassified | unclassified | unclassified | unclassified |
| Otu066955 | 1 | 1 | 1 | 0.001 | Bacteria | PAUC34f | unclassified | unclassified | unclassified | unclassified | unclassified |
| Otu067100 | 1 | 0.6667 | 0.816 | 0.01 | Bacteria | Proteobacteria | Deltaproteobacteria | NB1-j | MND4 | unclassified | unclassified |
| Otu067136 | 0.9195 | 0.6667 | 0.783 | 0.021 | Bacteria | Proteobacteria | Gammaproteobacteria | unclassified | unclassified | unclassified | unclassified |
| Otu067545 | 1 | 0.6667 | 0.816 | 0.006 | Bacteria | Proteobacteria | Gammaproteobacteria | unclassified | unclassified | unclassified | unclassified |
| Otu067842 | 1 | 1 | 1 | 0.001 | Bacteria | unclassified | unclassified | unclassified | unclassified | unclassified | unclassified |
| Otu068419 | 1 | 0.6667 | 0.816 | 0.006 | Bacteria | PAUC34f | unclassified | unclassified | unclassified | unclassified | unclassified |

| | | | | | | | | | | | |
|---|---|---|---|---|---|---|---|---|---|---|---|
| Otu068977 | 1 | 0.6667 | 0.816 | 0.01 | Bacteria | Chloroflexi | Anaerolineae | Caldilineales | Caldilineaceae | unclassified | unclassified |
| Otu069761 | 1 | 0.6667 | 0.816 | 0.008 | Bacteria | Nitrospirae | Nitrospira | Nitrospirales | Nitrospiraceae | unclassified | unclassified |
| Otu071686 | 1 | 1 | 1 | 0.001 | Bacteria | Chloroflexi | SAR202 | unclassified | unclassified | unclassified | unclassified |
| Otu071807 | 1 | 0.6667 | 0.816 | 0.006 | Bacteria | PAUC34f | unclassified | unclassified | unclassified | unclassified | unclassified |
| Otu072082 | 1 | 0.6667 | 0.816 | 0.01 | Bacteria | PAUC34f | unclassified | unclassified | unclassified | unclassified | unclassified |
| Otu072696 | 1 | 0.6667 | 0.816 | 0.01 | Bacteria | PAUC34f | unclassified | unclassified | unclassified | unclassified | unclassified |
| Otu074350 | 1 | 1 | 1 | 0.001 | Bacteria | Chloroflexi | SAR202 | unclassified | unclassified | unclassified | unclassified |
| Otu074491 | 1 | 1 | 1 | 0.001 | Bacteria | Gemmatimonadetes | Gemm-2 | unclassified | unclassified | unclassified | unclassified |
| Otu077926 | 1 | 0.6667 | 0.816 | 0.01 | Bacteria | unclassified | unclassified | unclassified | unclassified | unclassified | unclassified |
| Otu078415 | 1 | 1 | 1 | 0.001 | Bacteria | Proteobacteria | Deltaproteobacteria | Syntrophobacterales | Syntrophobacteraceae | unclassified | unclassified |
| Otu080882 | 1 | 0.6667 | 0.816 | 0.008 | Bacteria | Proteobacteria | Alphaproteobacteria | unclassified | unclassified | unclassified | unclassified |
| Otu080916 | 1 | 0.6667 | 0.816 | 0.01 | Bacteria | Proteobacteria | Alphaproteobacteria | Rhodospirillales | Rhodospirillaceae | unclassified | unclassified |
| Otu081851 | 1 | 1 | 1 | 0.001 | Bacteria | Acidobacteria | Acidobacteria-5 | unclassified | unclassified | unclassified | unclassified |
| Otu081868 | 1 | 0.6667 | 0.816 | 0.01 | Bacteria | Acidobacteria | PAUC37f | unclassified | unclassified | unclassified | unclassified |
| Otu081891 | 1 | 1 | 1 | 0.001 | Bacteria | Acidobacteria | Acidobacteria-6 | iii1-15 | unclassified | unclassified | unclassified |
| Otu081909 | 1 | 0.6667 | 0.816 | 0.006 | Bacteria | Acidobacteria | PAUC37f | unclassified | unclassified | unclassified | unclassified |
| Otu081916 | 1 | 0.6667 | 0.816 | 0.01 | Bacteria | Acidobacteria | Acidobacteria-6 | iii1-15 | unclassified | unclassified | unclassified |
| Otu081926 | 1 | 1 | 1 | 0.001 | Bacteria | Acidobacteria | Acidobacteria-6 | iii1-15 | unclassified | unclassified | unclassified |
| Otu081955 | 1 | 1 | 1 | 0.001 | Bacteria | Acidobacteria | Acidobacteria-5 | unclassified | unclassified | unclassified | unclassified |
| Otu081981 | 1 | 0.6667 | 0.816 | 0.01 | Bacteria | Acidobacteria | Acidobacteria-6 | unclassified | unclassified | unclassified | unclassified |
| Otu082306 | 1 | 1 | 1 | 0.001 | Bacteria | Acidobacteria | Acidobacteria-6 | iii1-15 | unclassified | unclassified | unclassified |
| Otu082308 | 1 | 1 | 1 | 0.001 | Bacteria | Acidobacteria | Acidobacteria-6 | iii1-15 | unclassified | unclassified | unclassified |
| Otu083047 | 1 | 1 | 1 | 0.001 | Bacteria | Proteobacteria | Alphaproteobacteria | unclassified | unclassified | unclassified | unclassified |
| Otu083244 | 1 | 1 | 1 | 0.001 | Bacteria | Chloroflexi | unclassified | unclassified | unclassified | unclassified | unclassified |
| Otu084490 | 1 | 0.6667 | 0.816 | 0.01 | Bacteria | Actinobacteria | Acidimicrobiia | Acidimicrobiales | TK06 | unclassified | unclassified |
| Otu086237 | 1 | 1 | 1 | 0.001 | Bacteria | Proteobacteria | Gammaproteobacteria | Alteromonadales | unclassified | unclassified | unclassified |
| Otu087663 | 1 | 0.6667 | 0.816 | 0.01 | Bacteria | Chloroflexi | SAR202 | unclassified | unclassified | unclassified | unclassified |
| Otu087743 | 1 | 1 | 1 | 0.001 | Bacteria | Chloroflexi | SAR202 | unclassified | unclassified | unclassified | unclassified |
| Otu088389 | 1 | 0.6667 | 0.816 | 0.008 | Bacteria | Chloroflexi | SAR202 | unclassified | unclassified | unclassified | unclassified |
| Otu089033 | 1 | 0.6667 | 0.816 | 0.008 | Bacteria | Chloroflexi | SAR202 | unclassified | unclassified | unclassified | unclassified |
| Otu090202 | 1 | 1 | 1 | 0.001 | Bacteria | Chloroflexi | TK17 | mle1-48 | unclassified | unclassified | unclassified |
| Otu090684 | 1 | 0.6667 | 0.816 | 0.006 | Bacteria | SBR1093 | EC214 | unclassified | unclassified | unclassified | unclassified |
| Otu091490 | 1 | 1 | 1 | 0.001 | Bacteria | Nitrospirae | Nitrospira | Nitrospirales | Nitrospiraceae | unclassified | unclassified |
| Otu091762 | 1 | 1 | 1 | 0.001 | Archaea | Crenarchaeota | Thaumarchaeota | Cenarchaeales | Cenarchaeaceae | unclassified | unclassified |
| Otu092093 | 0.9305 | 0.6667 | 0.788 | 0.021 | Bacteria | Chloroflexi | SAR202 | unclassified | unclassified | unclassified | unclassified |
| Otu092975 | 1 | 0.6667 | 0.816 | 0.006 | Bacteria | Chloroflexi | SAR202 | unclassified | unclassified | unclassified | unclassified |
| Otu093045 | 1 | 0.6667 | 0.816 | 0.008 | Bacteria | AncK6 | unclassified | unclassified | unclassified | unclassified | unclassified |
| Otu094674 | 1 | 1 | 1 | 0.001 | Bacteria | Chloroflexi | SAR202 | unclassified | unclassified | unclassified | unclassified |
| Otu095692 | 0.9912 | 1 | 0.996 | 0.001 | Bacteria | Chloroflexi | SAR202 | unclassified | unclassified | unclassified | unclassified |
| Otu095794 | 1 | 0.6667 | 0.816 | 0.01 | Bacteria | Gemmatimonadetes | Gemm-2 | unclassified | unclassified | unclassified | unclassified |
| Otu095941 | 1 | 1 | 1 | 0.001 | Bacteria | Acidobacteria | Sva0725 | Sva0725 | unclassified | unclassified | unclassified |
| Otu100591 | 1 | 0.6667 | 0.816 | 0.01 | Bacteria | Chloroflexi | SAR202 | unclassified | unclassified | unclassified | unclassified |
| Otu100690 | 1 | 0.6667 | 0.816 | 0.006 | Bacteria | Chloroflexi | SAR202 | unclassified | unclassified | unclassified | unclassified |
| Otu101503 | 1 | 0.6667 | 0.816 | 0.01 | Archaea | Crenarchaeota | Thaumarchaeota | Cenarchaeales | Cenarchaeaceae | unclassified | unclassified |
| Otu102198 | 1 | 0.6667 | 0.816 | 0.01 | Bacteria | Nitrospirae | Nitrospira | Nitrospirales | Nitrospiraceae | unclassified | unclassified |
| Otu103320 | 1 | 1 | 1 | 0.001 | Archaea | Crenarchaeota | Thaumarchaeota | Cenarchaeales | Cenarchaeaceae | unclassified | unclassified |
| Otu104303 | 1 | 0.6667 | 0.816 | 0.01 | Bacteria | unclassified | unclassified | unclassified | unclassified | unclassified | unclassified |
| Otu104786 | 1 | 0.6667 | 0.816 | 0.006 | Bacteria | Acidobacteria | PAUC37f | unclassified | unclassified | unclassified | unclassified |
| Otu105391 | 1 | 0.6667 | 0.816 | 0.008 | Bacteria | Chloroflexi | SAR202 | unclassified | unclassified | unclassified | unclassified |
| Otu105881 | 1 | 0.6667 | 0.816 | 0.01 | Bacteria | Nitrospirae | Nitrospira | Nitrospirales | Nitrospiraceae | unclassified | unclassified |
| Otu106823 | 1 | 0.6667 | 0.816 | 0.01 | Bacteria | Proteobacteria | Gammaproteobacteria | unclassified | unclassified | unclassified | unclassified |
| Otu108190 | 1 | 0.6667 | 0.816 | 0.006 | Bacteria | Proteobacteria | Gammaproteobacteria | unclassified | unclassified | unclassified | unclassified |
| Otu108513 | 1 | 1 | 1 | 0.001 | Bacteria | Proteobacteria | Gammaproteobacteria | unclassified | unclassified | unclassified | unclassified |
| Otu109147 | 1 | 0.6667 | 0.816 | 0.006 | Bacteria | Proteobacteria | Gammaproteobacteria | unclassified | unclassified | unclassified | unclassified |
| Otu109649 | 1 | 0.6667 | 0.816 | 0.008 | Bacteria | Proteobacteria | Gammaproteobacteria | unclassified | unclassified | unclassified | unclassified |
| Otu109759 | 1 | 0.6667 | 0.816 | 0.008 | Bacteria | Proteobacteria | Gammaproteobacteria | unclassified | unclassified | unclassified | unclassified |
| Otu110757 | 1 | 0.6667 | 0.816 | 0.01 | Bacteria | Proteobacteria | Gammaproteobacteria | unclassified | unclassified | unclassified | unclassified |
| Otu111040 | 1 | 1 | 1 | 0.001 | Bacteria | Proteobacteria | Gammaproteobacteria | unclassified | unclassified | unclassified | unclassified |
| Otu111332 | 0.9682 | 1 | 0.984 | 0.002 | Bacteria | SBR1093 | EC214 | unclassified | unclassified | unclassified | unclassified |
| Otu111424 | 1 | 0.6667 | 0.816 | 0.008 | Bacteria | Proteobacteria | Gammaproteobacteria | unclassified | unclassified | unclassified | unclassified |
| Otu112139 | 0.9166 | 0.6667 | 0.782 | 0.028 | Bacteria | SBR1093 | EC214 | unclassified | unclassified | unclassified | unclassified |
| Otu112548 | 1 | 0.6667 | 0.816 | 0.008 | Bacteria | SBR1093 | EC214 | unclassified | unclassified | unclassified | unclassified |
| Otu113370 | 1 | 1 | 1 | 0.001 | Bacteria | SBR1093 | EC214 | unclassified | unclassified | unclassified | unclassified |
| Otu114863 | 1 | 0.6667 | 0.816 | 0.008 | Bacteria | Proteobacteria | Deltaproteobacteria | unclassified | unclassified | unclassified | unclassified |
| Otu116006 | 1 | 0.6667 | 0.816 | 0.006 | Bacteria | Proteobacteria | Gammaproteobacteria | unclassified | unclassified | unclassified | unclassified |
| Otu116356 | 1 | 0.6667 | 0.816 | 0.01 | Bacteria | Proteobacteria | Gammaproteobacteria | unclassified | unclassified | unclassified | unclassified |
| Otu118502 | 1 | 0.6667 | 0.816 | 0.01 | Bacteria | Acidobacteria | Acidobacteria-6 | iii1-15 | unclassified | unclassified | unclassified |
| Otu120008 | 1 | 1 | 1 | 0.001 | Bacteria | Acidobacteria | Acidobacteria-6 | iii1-15 | unclassified | unclassified | unclassified |
| Otu121005 | 1 | 0.6667 | 0.816 | 0.008 | Bacteria | Proteobacteria | Gammaproteobacteria | unclassified | unclassified | unclassified | unclassified |
| Otu121830 | 1 | 1 | 1 | 0.001 | Bacteria | Proteobacteria | Gammaproteobacteria | unclassified | unclassified | unclassified | unclassified |
| Otu122093 | 1 | 0.6667 | 0.816 | 0.008 | Bacteria | Proteobacteria | Gammaproteobacteria | unclassified | unclassified | unclassified | unclassified |
| Otu123259 | 1 | 0.6667 | 0.816 | 0.008 | Bacteria | Proteobacteria | Gammaproteobacteria | unclassified | unclassified | unclassified | unclassified |
| Otu123340 | 1 | 1 | 1 | 0.001 | Bacteria | Proteobacteria | Gammaproteobacteria | unclassified | unclassified | unclassified | unclassified |
| Otu123529 | 1 | 1 | 1 | 0.001 | Bacteria | Proteobacteria | Gammaproteobacteria | unclassified | unclassified | unclassified | unclassified |
| Otu123890 | 1 | 0.6667 | 0.816 | 0.01 | Bacteria | Proteobacteria | Gammaproteobacteria | unclassified | unclassified | unclassified | unclassified |
| Otu124382 | 1 | 0.6667 | 0.816 | 0.01 | Bacteria | Proteobacteria | Gammaproteobacteria | unclassified | unclassified | unclassified | unclassified |
| Otu125066 | 1 | 0.6667 | 0.816 | 0.006 | Bacteria | Proteobacteria | Gammaproteobacteria | HTCC2188 | HTCC2089 | unclassified | unclassified |
| Otu125168 | 1 | 1 | 1 | 0.001 | Bacteria | Proteobacteria | Gammaproteobacteria | unclassified | unclassified | unclassified | unclassified |
| Otu125264 | 1 | 0.6667 | 0.816 | 0.008 | Bacteria | Proteobacteria | Gammaproteobacteria | unclassified | unclassified | unclassified | unclassified |
| Otu126446 | 1 | 1 | 1 | 0.001 | Bacteria | Proteobacteria | Gammaproteobacteria | unclassified | unclassified | unclassified | unclassified |
| Otu126721 | 1 | 0.6667 | 0.816 | 0.01 | Bacteria | Proteobacteria | Gammaproteobacteria | unclassified | unclassified | unclassified | unclassified |
| Otu127190 | 1 | 1 | 1 | 0.001 | Bacteria | Proteobacteria | Gammaproteobacteria | unclassified | unclassified | unclassified | unclassified |
| Otu128486 | 1 | 0.6667 | 0.816 | 0.006 | Bacteria | Proteobacteria | Gammaproteobacteria | unclassified | unclassified | unclassified | unclassified |
| Otu129588 | 1 | 0.6667 | 0.816 | 0.006 | Bacteria | Proteobacteria | Gammaproteobacteria | unclassified | unclassified | unclassified | unclassified |
| Otu130350 | 1 | 0.6667 | 0.816 | 0.008 | Bacteria | Proteobacteria | Gammaproteobacteria | unclassified | unclassified | unclassified | unclassified |
| Otu135077 | 0.9314 | 0.6667 | 0.788 | 0.021 | Bacteria | SBR1093 | EC214 | unclassified | unclassified | unclassified | unclassified |
| Otu135313 | 1 | 0.6667 | 0.816 | 0.008 | Bacteria | Proteobacteria | Gammaproteobacteria | unclassified | unclassified | unclassified | unclassified |
| Otu135352 | 1 | 0.6667 | 0.816 | 0.01 | Bacteria | SBR1093 | EC214 | unclassified | unclassified | unclassified | unclassified |
| Otu136565 | 1 | 0.6667 | 0.816 | 0.01 | Bacteria | SBR1093 | EC214 | unclassified | unclassified | unclassified | unclassified |
| Otu136790 | 1 | 0.6667 | 0.816 | 0.006 | Bacteria | Proteobacteria | Gammaproteobacteria | unclassified | unclassified | unclassified | unclassified |
| Otu139218 | 1 | 1 | 1 | 0.001 | Bacteria | SBR1093 | EC214 | unclassified | unclassified | unclassified | unclassified |
| Otu139835 | 1 | 0.6667 | 0.816 | 0.006 | Bacteria | Proteobacteria | unclassified | unclassified | unclassified | unclassified | unclassified |
| Otu140864 | 1 | 0.6667 | 0.816 | 0.006 | Bacteria | Proteobacteria | unclassified | unclassified | unclassified | unclassified | unclassified |
| Otu141923 | 1 | 0.6667 | 0.816 | 0.01 | Bacteria | Acidobacteria | Acidobacteria-6 | iii1-15 | unclassified | unclassified | unclassified |
| Otu145500 | 1 | 0.6667 | 0.816 | 0.01 | Bacteria | Proteobacteria | Alphaproteobacteria | Rhodobacterales | Rhodobacteraceae | unclassified | unclassified |
| Otu149687 | 1 | 0.6667 | 0.816 | 0.01 | Bacteria | Acidobacteria | Acidobacteria-6 | iii1-15 | unclassified | unclassified | unclassified |
| Otu161677 | 1 | 1 | 1 | 0.001 | Bacteria | unclassified | unclassified | unclassified | unclassified | unclassified | unclassified |
| Otu166126 | 1 | 0.6667 | 0.816 | 0.01 | Bacteria | Proteobacteria | Gammaproteobacteria | unclassified | unclassified | unclassified | unclassified |
| Otu171653 | 1 | 0.6667 | 0.816 | 0.01 | Bacteria | Actinobacteria | Acidimicrobiia | Acidimicrobiales | wb1_P06 | unclassified | unclassified |
| Otu174830 | 1 | 0.6667 | 0.816 | 0.006 | Bacteria | Proteobacteria | Gammaproteobacteria | unclassified | unclassified | unclassified | unclassified |

| | | | | | | | | | | | |
|---|---|---|---|---|---|---|---|---|---|---|---|
| Otu179948 | 1 | 0.6667 | 0.816 | 0.008 | Bacteria | SBR1093 | EC214 | unclassified | unclassified | unclassified | unclassified |
| Otu181526 | 1 | 1 | 1 | 0.001 | Bacteria | Proteobacteria | Alphaproteobacteria | unclassified | unclassified | unclassified | unclassified |
| Otu191352 | 1 | 0.6667 | 0.816 | 0.006 | Bacteria | PAUC34f | unclassified | unclassified | unclassified | unclassified | unclassified |
| Otu194626 | 1 | 0.6667 | 0.816 | 0.008 | Bacteria | PAUC34f | unclassified | unclassified | unclassified | unclassified | unclassified |
| Otu194675 | 1 | 0.6667 | 0.816 | 0.008 | Bacteria | PAUC34f | unclassified | unclassified | unclassified | unclassified | unclassified |
| Otu194734 | 1 | 0.6667 | 0.816 | 0.01 | Bacteria | PAUC34f | unclassified | unclassified | unclassified | unclassified | unclassified |
| Otu196702 | 1 | 1 | 1 | 0.001 | Bacteria | Proteobacteria | Gammaproteobacteria | unclassified | unclassified | unclassified | unclassified |
| Otu196942 | 1 | 1 | 1 | 0.001 | Bacteria | Poribacteria | unclassified | unclassified | unclassified | unclassified | unclassified |
| Otu199560 | 1 | 0.6667 | 0.816 | 0.008 | Bacteria | SBR1093 | EC214 | unclassified | unclassified | unclassified | unclassified |
| Otu201482 | 1 | 1 | 1 | 0.001 | Bacteria | Proteobacteria | Gammaproteobacteria | unclassified | unclassified | unclassified | unclassified |
| Otu204134 | 1 | 0.6667 | 0.816 | 0.01 | Bacteria | SBR1093 | EC214 | unclassified | unclassified | unclassified | unclassified |
| Otu204390 | 1 | 0.6667 | 0.816 | 0.006 | Bacteria | Proteobacteria | Gammaproteobacteria | unclassified | unclassified | unclassified | unclassified |
| Otu207449 | 1 | 0.6667 | 0.816 | 0.006 | Bacteria | Proteobacteria | Gammaproteobacteria | unclassified | unclassified | unclassified | unclassified |
| Otu207884 | 1 | 1 | 1 | 0.001 | Bacteria | Proteobacteria | Gammaproteobacteria | unclassified | unclassified | unclassified | unclassified |
| Otu209163 | 1 | 0.6667 | 0.816 | 0.008 | Bacteria | Proteobacteria | Gammaproteobacteria | unclassified | unclassified | unclassified | unclassified |
| Otu209321 | 1 | 0.6667 | 0.816 | 0.01 | Bacteria | Proteobacteria | Gammaproteobacteria | unclassified | unclassified | unclassified | unclassified |
| Otu213906 | 1 | 0.6667 | 0.816 | 0.01 | Bacteria | Proteobacteria | Gammaproteobacteria | unclassified | unclassified | unclassified | unclassified |
| Otu214615 | 1 | 0.6667 | 0.816 | 0.006 | Bacteria | Proteobacteria | Gammaproteobacteria | unclassified | unclassified | unclassified | unclassified |
| Otu216763 | 1 | 1 | 1 | 0.001 | Bacteria | Proteobacteria | Gammaproteobacteria | unclassified | unclassified | unclassified | unclassified |
| Otu218337 | 1 | 0.6667 | 0.816 | 0.006 | Bacteria | Proteobacteria | Gammaproteobacteria | unclassified | unclassified | unclassified | unclassified |
| Otu218453 | 1 | 1 | 1 | 0.001 | Bacteria | Proteobacteria | Gammaproteobacteria | unclassified | unclassified | unclassified | unclassified |
| Otu237587 | 1 | 0.6667 | 0.816 | 0.01 | Bacteria | Proteobacteria | Gammaproteobacteria | unclassified | unclassified | unclassified | unclassified |
| Otu241283 | 1 | 0.6667 | 0.816 | 0.008 | Bacteria | Proteobacteria | Gammaproteobacteria | unclassified | unclassified | unclassified | unclassified |
| Otu248385 | 1 | 0.6667 | 0.816 | 0.008 | Bacteria | Proteobacteria | Gammaproteobacteria | unclassified | unclassified | unclassified | unclassified |
| Otu248881 | 1 | 0.6667 | 0.816 | 0.006 | Bacteria | Proteobacteria | Gammaproteobacteria | Chromatiales | unclassified | unclassified | unclassified |
| Otu248960 | 1 | 1 | 1 | 0.001 | Bacteria | Proteobacteria | Gammaproteobacteria | unclassified | unclassified | unclassified | unclassified |
| Otu250950 | 1 | 0.6667 | 0.816 | 0.008 | Bacteria | Proteobacteria | Gammaproteobacteria | unclassified | unclassified | unclassified | unclassified |
| Otu252816 | 1 | 1 | 1 | 0.001 | Bacteria | Proteobacteria | Gammaproteobacteria | unclassified | unclassified | unclassified | unclassified |
| Otu253868 | 1 | 1 | 1 | 0.001 | Bacteria | Proteobacteria | Gammaproteobacteria | unclassified | unclassified | unclassified | unclassified |
| Otu254572 | 1 | 0.6667 | 0.816 | 0.006 | Bacteria | Proteobacteria | Gammaproteobacteria | unclassified | unclassified | unclassified | unclassified |
| Otu255850 | 1 | 0.6667 | 0.816 | 0.01 | Bacteria | Proteobacteria | Gammaproteobacteria | unclassified | unclassified | unclassified | unclassified |
| Otu257222 | 1 | 0.6667 | 0.816 | 0.01 | Bacteria | Proteobacteria | Gammaproteobacteria | unclassified | unclassified | unclassified | unclassified |
| Otu259932 | 1 | 0.6667 | 0.816 | 0.006 | Bacteria | Proteobacteria | Gammaproteobacteria | unclassified | unclassified | unclassified | unclassified |
| Otu265891 | 1 | 1 | 1 | 0.001 | Archaea | Crenarchaeota | Thaumarchaeota | Cenarchaeales | Cenarchaeaceae | unclassified | unclassified |
| Otu266239 | 1 | 1 | 1 | 0.001 | Archaea | Crenarchaeota | Thaumarchaeota | Cenarchaeales | Cenarchaeaceae | unclassified | unclassified |
| Otu274609 | 1 | 1 | 1 | 0.001 | Archaea | Crenarchaeota | Thaumarchaeota | Cenarchaeales | Cenarchaeaceae | unclassified | unclassified |
| Otu275296 | 1 | 1 | 1 | 0.001 | Bacteria | Gemmatimonadetes | Gemm-1 | unclassified | unclassified | unclassified | unclassified |
| Otu281405 | 0.9233 | 0.6667 | 0.785 | 0.027 | Bacteria | Acidobacteria | PAUC37f | unclassified | unclassified | unclassified | unclassified |
| Otu281446 | 1 | 1 | 1 | 0.001 | Bacteria | Acidobacteria | EC1113 | unclassified | unclassified | unclassified | unclassified |
| Otu281477 | 1 | 1 | 1 | 0.001 | Bacteria | unclassified | unclassified | unclassified | unclassified | unclassified | unclassified |
| Otu282073 | 1 | 1 | 1 | 0.001 | Bacteria | Acidobacteria | Acidobacteria-6 | iii1-15 | unclassified | unclassified | unclassified |
| Otu282080 | 1 | 0.6667 | 0.816 | 0.006 | Bacteria | Acidobacteria | PAUC37f | unclassified | unclassified | unclassified | unclassified |
| Otu296869 | 1 | 0.6667 | 0.816 | 0.01 | Bacteria | Acidobacteria | Solibacteres | Solibacterales | PAUC26f | unclassified | unclassified |
| Otu299511 | 1 | 1 | 1 | 0.001 | Archaea | Crenarchaeota | Thaumarchaeota | Cenarchaeales | Cenarchaeaceae | unclassified | unclassified |
| Otu304478 | 1 | 0.6667 | 0.816 | 0.01 | Bacteria | SBR1093 | EC214 | unclassified | unclassified | unclassified | unclassified |
| Otu306281 | 1 | 1 | 1 | 0.001 | Bacteria | Acidobacteria | Solibacteres | Solibacterales | PAUC26f | unclassified | unclassified |
| Otu311528 | 1 | 0.6667 | 0.816 | 0.01 | Archaea | Crenarchaeota | Thaumarchaeota | Cenarchaeales | Cenarchaeaceae | unclassified | unclassified |
| Otu313458 | 1 | 0.6667 | 0.816 | 0.008 | Bacteria | Nitrospirae | Nitrospira | Nitrospirales | Nitrospiraceae | unclassified | unclassified |
| Otu315572 | 1 | 1 | 1 | 0.001 | Bacteria | Acidobacteria | Acidobacteria-6 | iii1-15 | unclassified | unclassified | unclassified |
| Otu318732 | 1 | 0.6667 | 0.816 | 0.006 | Bacteria | Chloroflexi | SAR202 | unclassified | unclassified | unclassified | unclassified |

| **0.03 Otus** | ***Axinella* sp.** | **Rest** | **stat** | ***p value*** | **Domain** | **Phylum** | **Class** | **Order** | **Family** | **Genus** | **Species** |
|---|---|---|---|---|---|---|---|---|---|---|---|
| Otu000046 | 0.8858 | 0.6667 | 0.768 | 0.01 | Bacteria | Proteobacteria | Alphaproteobacteria | Rhodobacterales | Hyphomonadaceae | Maricaulis | unclassified |
| Otu000129 | 0.9546 | 1 | 0.977 | 0.001 | Bacteria | Proteobacteria | unclassified | unclassified | unclassified | unclassified | unclassified |
| Otu000139 | 0.7936 | 1 | 0.891 | 0.008 | Bacteria | Proteobacteria | unclassified | unclassified | unclassified | unclassified | unclassified |
| Otu001166 | 0.9959 | 1 | 0.998 | 0.001 | Bacteria | Proteobacteria | Gammaproteobacteria | unclassified | unclassified | unclassified | unclassified |
| Otu001175 | 0.9773 | 1 | 0.989 | 0.001 | Bacteria | unclassified | unclassified | unclassified | unclassified | unclassified | unclassified |
| Otu001317 | 0.971 | 1 | 0.985 | 0.001 | Archaea | Crenarchaeota | Thaumarchaeota | Cenarchaeales | Cenarchaeaceae | Nitrosopumilus | unclassified |
| Otu001502 | 0.9855 | 1 | 0.993 | 0.001 | Bacteria | Proteobacteria | Deltaproteobacteria | Desulfobacterales | Nitrospinaceae | Nitrospina | unclassified |
| Otu001727 | 0.8686 | 0.6667 | 0.761 | 0.025 | Bacteria | Proteobacteria | Deltaproteobacteria | Bdellovibrionales | Bacteriovoracaceae | unclassified | unclassified |
| Otu002275 | 0.8422 | 1 | 0.918 | 0.005 | Bacteria | Proteobacteria | unclassified | unclassified | unclassified | unclassified | unclassified |
| Otu002284 | 0.8492 | 0.6667 | 0.752 | 0.021 | Bacteria | Proteobacteria | Betaproteobacteria | Nitrosomonadales | Nitrosomonadaceae | unclassified | unclassified |
| Otu002651 | 0.8584 | 0.6667 | 0.757 | 0.027 | Bacteria | Proteobacteria | Deltaproteobacteria | NB1-j | JTB38 | unclassified | unclassified |
| Otu002672 | 0.978 | 1 | 0.989 | 0.001 | Bacteria | Proteobacteria | Gammaproteobacteria | unclassified | unclassified | unclassified | unclassified |
| Otu002797 | 1 | 0.6667 | 0.816 | 0.007 | Bacteria | Proteobacteria | unclassified | unclassified | unclassified | unclassified | unclassified |
| Otu002957 | 1 | 0.6667 | 0.816 | 0.007 | Bacteria | Bacteroidetes | unclassified | unclassified | unclassified | unclassified | unclassified |
| Otu003335 | 0.9313 | 0.6667 | 0.788 | 0.008 | Bacteria | Proteobacteria | Gammaproteobacteria | Legionellales | Endoecteinascidiaceae | unclassified | unclassified |
| Otu003466 | 0.9521 | 1 | 0.976 | 0.004 | Bacteria | Planctomycetes | Planctomycetia | Pirellulales | Pirellulaceae | unclassified | unclassified |
| Otu003540 | 0.9801 | 1 | 0.99 | 0.001 | Bacteria | Proteobacteria | Alphaproteobacteria | unclassified | unclassified | unclassified | unclassified |
| Otu003600 | 0.9553 | 0.6667 | 0.798 | 0.007 | Bacteria | Bacteroidetes | Saprospirae | Saprospirales | Saprospiraceae | unclassified | unclassified |
| Otu003657 | 0.7806 | 0.6667 | 0.721 | 0.032 | Bacteria | Proteobacteria | Gammaproteobacteria | Vibrionales | Pseudoalteromonadacea | Pseudoalteromonas | tunicata |
| Otu003721 | 1 | 1 | 1 | 0.001 | Bacteria | SBR1093 | EC214 | unclassified | unclassified | unclassified | unclassified |
| Otu004426 | 0.9883 | 1 | 0.994 | 0.001 | Bacteria | Proteobacteria | Alphaproteobacteria | unclassified | unclassified | unclassified | unclassified |
| Otu004564 | 0.7986 | 0.6667 | 0.73 | 0.03 | Bacteria | Proteobacteria | Deltaproteobacteria | Sva0853 | SAR324 | unclassified | unclassified |
| Otu004850 | 0.8856 | 1 | 0.941 | 0.003 | Bacteria | Proteobacteria | Gammaproteobacteria | unclassified | unclassified | unclassified | unclassified |
| Otu005024 | 0.9382 | 0.6667 | 0.791 | 0.015 | Bacteria | Proteobacteria | Alphaproteobacteria | Rickettsiales | Pelagibacteraceae | unclassified | unclassified |
| Otu005540 | 1 | 1 | 1 | 0.001 | Bacteria | Proteobacteria | unclassified | unclassified | unclassified | unclassified | unclassified |
| Otu005626 | 0.9069 | 0.6667 | 0.778 | 0.023 | Bacteria | Proteobacteria | Gammaproteobacteria | unclassified | unclassified | unclassified | unclassified |
| Otu005709 | 0.8574 | 0.6667 | 0.756 | 0.032 | Bacteria | Proteobacteria | Gammaproteobacteria | unclassified | unclassified | unclassified | unclassified |
| Otu005877 | 0.7421 | 1 | 0.861 | 0.024 | Bacteria | Proteobacteria | Gammaproteobacteria | unclassified | unclassified | unclassified | unclassified |
| Otu006002 | 0.8255 | 0.6667 | 0.742 | 0.02 | Bacteria | Proteobacteria | Deltaproteobacteria | Desulfobacterales | Nitrospinaceae | Nitrospina | unclassified |
| Otu006868 | 0.9503 | 1 | 0.975 | 0.001 | Bacteria | Proteobacteria | unclassified | unclassified | unclassified | unclassified | unclassified |
| Otu007015 | 0.9584 | 1 | 0.979 | 0.001 | Bacteria | Proteobacteria | Betaproteobacteria | unclassified | unclassified | unclassified | unclassified |
| Otu007084 | 0.8444 | 0.6667 | 0.75 | 0.042 | Bacteria | Proteobacteria | Betaproteobacteria | unclassified | unclassified | unclassified | unclassified |
| Otu007299 | 0.9318 | 1 | 0.965 | 0.003 | Bacteria | Proteobacteria | unclassified | unclassified | unclassified | unclassified | unclassified |
| Otu007306 | 0.8557 | 0.6667 | 0.755 | 0.034 | Bacteria | Proteobacteria | unclassified | unclassified | unclassified | unclassified | unclassified |
| Otu007564 | 0.9438 | 0.6667 | 0.793 | 0.007 | Bacteria | Proteobacteria | Alphaproteobacteria | Rickettsiales | Pelagibacteraceae | unclassified | unclassified |
| Otu008664 | 0.9592 | 1 | 0.979 | 0.001 | Bacteria | Proteobacteria | unclassified | unclassified | unclassified | unclassified | unclassified |
| Otu008760 | 0.8719 | 1 | 0.934 | 0.007 | Bacteria | Planctomycetes | Planctomycetia | unclassified | unclassified | unclassified | unclassified |
| Otu008875 | 0.7901 | 0.6667 | 0.726 | 0.032 | Bacteria | Chloroflexi | SAR202 | unclassified | unclassified | unclassified | unclassified |
| Otu009454 | 0.8969 | 0.6667 | 0.773 | 0.013 | Bacteria | Bacteroidetes | Saprospirae | Saprospirales | Saprospiraceae | unclassified | unclassified |
| Otu009610 | 0.82 | 1 | 0.906 | 0.008 | Bacteria | Proteobacteria | unclassified | unclassified | unclassified | unclassified | unclassified |
| Otu010012 | 0.8353 | 0.6667 | 0.746 | 0.023 | Bacteria | unclassified | unclassified | unclassified | unclassified | unclassified | unclassified |
| Otu010017 | 0.8458 | 0.6667 | 0.751 | 0.014 | Bacteria | Proteobacteria | Alphaproteobacteria | Rhodobacterales | Rhodobacteraceae | Octadecabacter | unclassified |
| Otu010175 | 0.8337 | 0.6667 | 0.746 | 0.037 | Bacteria | Bacteroidetes | Flavobacteriia | Flavobacteriales | Cryomorphaceae | Fluviicola | unclassified |
| Otu010443 | 0.8584 | 0.6667 | 0.756 | 0.029 | Bacteria | PAUC34f | unclassified | unclassified | unclassified | unclassified | unclassified |
| Otu010958 | 0.9388 | 0.6667 | 0.791 | 0.019 | Bacteria | Planctomycetes | Planctomycetia | Pirellulales | Pirellulaceae | unclassified | unclassified |
| Otu011957 | 1 | 1 | 1 | 0.001 | Bacteria | Proteobacteria | Alphaproteobacteria | unclassified | unclassified | unclassified | unclassified |
| Otu012558 | 0.9743 | 1 | 0.987 | 0.001 | Bacteria | Proteobacteria | unclassified | unclassified | unclassified | unclassified | unclassified |
| Otu012592 | 0.7972 | 0.6667 | 0.729 | 0.019 | Bacteria | Gemmatimonadetes | Gemm-2 | unclassified | unclassified | unclassified | unclassified |
| Otu012626 | 0.85 | 0.6667 | 0.753 | 0.037 | Bacteria | Proteobacteria | unclassified | unclassified | unclassified | unclassified | unclassified |

| | | | | | | | | | | | |
|---|---|---|---|---|---|---|---|---|---|---|---|
| Otu012647 | 0.8383 | 1 | 0.916 | 0.015 | Bacteria | Planctomycetes | Planctomycetia | Planctomycetales | Planctomycetaceae | Planctomyces | unclassified |
| Otu012855 | 0.987 | 1 | 0.994 | 0.001 | Archaea | Crenarchaeota | Thaumarchaeota | Cenarchaeales | Cenarchaeaceae | Nitrosopumilus | unclassified |
| Otu012955 | 0.9788 | 0.6667 | 0.808 | 0.007 | Archaea | Crenarchaeota | Thaumarchaeota | Cenarchaeales | Cenarchaeaceae | Nitrosopumilus | unclassified |
| Otu012962 | 0.9059 | 1 | 0.952 | 0.006 | Bacteria | unclassified | unclassified | unclassified | unclassified | unclassified | unclassified |
| Otu013287 | 1 | 0.6667 | 0.816 | 0.011 | Bacteria | Proteobacteria | Gammaproteobacteria | Legionellales | Endoecteinascidiaceae | unclassified | unclassified |
| Otu013336 | 0.8409 | 0.6667 | 0.749 | 0.041 | Bacteria | Planctomycetes | OM190 | CL500-15 | unclassified | unclassified | unclassified |
| Otu013687 | 0.9313 | 0.6667 | 0.788 | 0.008 | Bacteria | Proteobacteria | Gammaproteobacteria | unclassified | unclassified | unclassified | unclassified |
| Otu014109 | 0.9114 | 0.6667 | 0.779 | 0.019 | Bacteria | Proteobacteria | unclassified | unclassified | unclassified | unclassified | unclassified |
| Otu014161 | 1 | 1 | 1 | 0.001 | Bacteria | Proteobacteria | unclassified | unclassified | unclassified | unclassified | unclassified |
| Otu014464 | 0.9696 | 1 | 0.985 | 0.001 | Bacteria | Proteobacteria | Gammaproteobacteria | unclassified | unclassified | unclassified | unclassified |
| Otu014872 | 1 | 0.6667 | 0.816 | 0.007 | Bacteria | Bacteroidetes | Flavobacteriia | Flavobacteriales | Flavobacteriaceae | unclassified | unclassified |
| Otu015022 | 0.8299 | 1 | 0.911 | 0.003 | Archaea | Crenarchaeota | Thaumarchaeota | Cenarchaeales | Cenarchaeaceae | Nitrosopumilus | unclassified |
| Otu015498 | 0.9634 | 1 | 0.982 | 0.001 | Bacteria | Proteobacteria | unclassified | unclassified | unclassified | unclassified | unclassified |
| Otu015583 | 1 | 1 | 1 | 0.001 | Archaea | Crenarchaeota | Thaumarchaeota | Cenarchaeales | Cenarchaeaceae | Nitrosopumilus | unclassified |
| Otu015596 | 0.8866 | 1 | 0.942 | 0.004 | Bacteria | Proteobacteria | Deltaproteobacteria | NB1-j | JTB38 | unclassified | unclassified |
| Otu016018 | 0.9777 | 1 | 0.989 | 0.001 | Bacteria | Proteobacteria | unclassified | unclassified | unclassified | unclassified | unclassified |
| Otu016022 | 0.928 | 1 | 0.963 | 0.001 | Bacteria | Proteobacteria | unclassified | unclassified | unclassified | unclassified | unclassified |
| Otu016057 | 0.8397 | 0.6667 | 0.748 | 0.043 | Bacteria | Bacteroidetes | Cytophagia | Cytophagales | Flammeovirgaceae | unclassified | unclassified |
| Otu016318 | 0.8583 | 0.6667 | 0.756 | 0.038 | Bacteria | Proteobacteria | Betaproteobacteria | Nitrosomonadales | Nitrosomonadaceae | unclassified | unclassified |
| Otu016459 | 0.8678 | 0.6667 | 0.761 | 0.028 | Bacteria | Proteobacteria | Alphaproteobacteria | unclassified | unclassified | unclassified | unclassified |
| Otu016634 | 0.8065 | 0.6667 | 0.733 | 0.045 | Bacteria | Proteobacteria | Gammaproteobacteria | unclassified | unclassified | unclassified | unclassified |
| Otu016674 | 1 | 1 | 1 | 0.001 | Bacteria | Proteobacteria | Alphaproteobacteria | Rhizobiales | unclassified | unclassified | unclassified |
| Otu017696 | 0.7351 | 1 | 0.857 | 0.029 | Bacteria | Proteobacteria | Gammaproteobacteria | Legionellales | Legionellaceae | unclassified | unclassified |
| Otu017945 | 0.9556 | 1 | 0.978 | 0.002 | Bacteria | Proteobacteria | Gammaproteobacteria | unclassified | unclassified | unclassified | unclassified |
| Otu018113 | 1 | 1 | 1 | 0.001 | Bacteria | Proteobacteria | Gammaproteobacteria | unclassified | unclassified | unclassified | unclassified |
| Otu018175 | 0.9598 | 1 | 0.98 | 0.001 | Bacteria | Proteobacteria | unclassified | unclassified | unclassified | unclassified | unclassified |
| Otu018271 | 0.8682 | 0.6667 | 0.761 | 0.014 | Bacteria | Proteobacteria | Gammaproteobacteria | unclassified | unclassified | unclassified | unclassified |
| Otu018282 | 0.9509 | 1 | 0.975 | 0.001 | Bacteria | Proteobacteria | unclassified | unclassified | unclassified | unclassified | unclassified |
| Otu018549 | 0.9549 | 1 | 0.977 | 0.001 | Bacteria | Proteobacteria | unclassified | unclassified | unclassified | unclassified | unclassified |
| Otu018550 | 0.9778 | 1 | 0.989 | 0.001 | Bacteria | Proteobacteria | unclassified | unclassified | unclassified | unclassified | unclassified |
| Otu018601 | 0.9247 | 1 | 0.962 | 0.006 | Bacteria | Proteobacteria | Gammaproteobacteria | unclassified | unclassified | unclassified | unclassified |
| Otu018603 | 0.92 | 1 | 0.959 | 0.001 | Bacteria | Proteobacteria | unclassified | unclassified | unclassified | unclassified | unclassified |
| Otu018737 | 0.9753 | 1 | 0.988 | 0.001 | Archaea | Crenarchaeota | Thaumarchaeota | Cenarchaeales | Cenarchaeaceae | Nitrosopumilus | unclassified |
| Otu018759 | 0.8597 | 1 | 0.927 | 0.008 | Bacteria | Proteobacteria | unclassified | unclassified | unclassified | unclassified | unclassified |
| Otu018814 | 0.9654 | 1 | 0.983 | 0.001 | Bacteria | Proteobacteria | unclassified | unclassified | unclassified | unclassified | unclassified |
| Otu018836 | 0.9782 | 1 | 0.989 | 0.001 | Bacteria | Proteobacteria | unclassified | unclassified | unclassified | unclassified | unclassified |
| Otu019039 | 0.9532 | 1 | 0.976 | 0.005 | Bacteria | Proteobacteria | Gammaproteobacteria | Vibrionales | Pseudoalteromonadacea | Pseudoalteromonas | porphyrae |
| Otu019421 | 1 | 0.6667 | 0.816 | 0.007 | Bacteria | Proteobacteria | Gammaproteobacteria | Vibrionales | Vibrionaceae | Vibrio | unclassified |
| Otu019814 | 1 | 1 | 1 | 0.001 | Bacteria | Proteobacteria | Gammaproteobacteria | unclassified | unclassified | unclassified | unclassified |
| Otu019829 | 0.9632 | 1 | 0.981 | 0.001 | Bacteria | Proteobacteria | unclassified | unclassified | unclassified | unclassified | unclassified |
| Otu020469 | 0.981 | 1 | 0.99 | 0.001 | Bacteria | Proteobacteria | Betaproteobacteria | unclassified | unclassified | unclassified | unclassified |
| Otu020495 | 0.9682 | 1 | 0.984 | 0.002 | Bacteria | Proteobacteria | unclassified | unclassified | unclassified | unclassified | unclassified |
| Otu020689 | 0.9677 | 1 | 0.984 | 0.001 | Bacteria | Proteobacteria | unclassified | unclassified | unclassified | unclassified | unclassified |
| Otu020742 | 0.9299 | 1 | 0.964 | 0.001 | Bacteria | Proteobacteria | unclassified | unclassified | unclassified | unclassified | unclassified |
| Otu020753 | 0.9717 | 1 | 0.986 | 0.001 | Bacteria | Proteobacteria | unclassified | unclassified | unclassified | unclassified | unclassified |
| Otu021005 | 0.8773 | 1 | 0.937 | 0.004 | Bacteria | Proteobacteria | unclassified | unclassified | unclassified | unclassified | unclassified |
| Otu021575 | 0.7856 | 0.6667 | 0.724 | 0.046 | Bacteria | Proteobacteria | Alphaproteobacteria | Rhodospirillales | Rhodospirillaceae | unclassified | unclassified |
| Otu021604 | 0.8553 | 0.6667 | 0.755 | 0.01 | Bacteria | SAR406 | AB16 | Arctic96B-7 | A714017 | SargSea-WGS | unclassified |
| Otu022351 | 0.8901 | 0.6667 | 0.77 | 0.008 | Bacteria | Verrucomicrobia | Pedosphaerae | Arctic97B-4 | unclassified | unclassified | unclassified |
| Otu022517 | 0.8777 | 0.6667 | 0.765 | 0.039 | Bacteria | Proteobacteria | Gammaproteobacteria | Alteromonadales | Colwelliaceae | unclassified | unclassified |
| Otu024955 | 0.8511 | 0.6667 | 0.753 | 0.015 | Bacteria | Proteobacteria | Betaproteobacteria | Nitrosomonadales | Nitrosomonadaceae | unclassified | unclassified |
| Otu025018 | 0.8858 | 0.6667 | 0.768 | 0.027 | Bacteria | Proteobacteria | Betaproteobacteria | Methylophilales | Methylophilaceae | unclassified | unclassified |
| Otu025033 | 0.7998 | 0.6667 | 0.73 | 0.019 | Bacteria | Verrucomicrobia | Pedosphaerae | unclassified | unclassified | unclassified | unclassified |
| Otu025701 | 1 | 0.6667 | 0.816 | 0.007 | Archaea | Crenarchaeota | Thaumarchaeota | Cenarchaeales | Cenarchaeaceae | Nitrosopumilus | unclassified |
| Otu025832 | 0.7439 | 0.6667 | 0.704 | 0.043 | Bacteria | Bacteroidetes | Flavobacteriia | Flavobacteriales | Flavobacteriaceae | unclassified | unclassified |
| Otu026675 | 0.9704 | 1 | 0.985 | 0.001 | Bacteria | Proteobacteria | unclassified | unclassified | unclassified | unclassified | unclassified |
| Otu026982 | 0.9449 | 1 | 0.972 | 0.001 | Bacteria | Proteobacteria | unclassified | unclassified | unclassified | unclassified | unclassified |
| Otu026986 | 0.9518 | 1 | 0.976 | 0.001 | Bacteria | Proteobacteria | unclassified | unclassified | unclassified | unclassified | unclassified |
| Otu027109 | 0.7127 | 1 | 0.844 | 0.027 | Bacteria | Chloroflexi | SAR202 | unclassified | unclassified | unclassified | unclassified |
| Otu027170 | 0.9719 | 1 | 0.986 | 0.001 | Bacteria | Proteobacteria | unclassified | unclassified | unclassified | unclassified | unclassified |
| Otu027228 | 0.9595 | 1 | 0.98 | 0.001 | Bacteria | Proteobacteria | unclassified | unclassified | unclassified | unclassified | unclassified |
| Otu029327 | 0.9307 | 1 | 0.965 | 0.001 | Bacteria | Proteobacteria | Gammaproteobacteria | unclassified | unclassified | unclassified | unclassified |
| Otu029374 | 0.9635 | 1 | 0.982 | 0.001 | Bacteria | Proteobacteria | unclassified | unclassified | unclassified | unclassified | unclassified |
| Otu029599 | 0.785 | 0.6667 | 0.723 | 0.03 | Bacteria | Proteobacteria | Gammaproteobacteria | unclassified | unclassified | unclassified | unclassified |
| Otu029727 | 1 | 0.6667 | 0.816 | 0.01 | Bacteria | Proteobacteria | unclassified | unclassified | unclassified | unclassified | unclassified |
| Otu029948 | 0.9088 | 0.6667 | 0.778 | 0.033 | Bacteria | Proteobacteria | Gammaproteobacteria | Legionellales | Legionellaceae | unclassified | unclassified |
| Otu033129 | 0.826 | 0.6667 | 0.742 | 0.039 | Bacteria | Proteobacteria | Gammaproteobacteria | Oceanospirillales | Saccharospirillaceae | Reinekea | unclassified |
| Otu033291 | 0.7298 | 1 | 0.854 | 0.024 | Archaea | Crenarchaeota | Thaumarchaeota | Cenarchaeales | Cenarchaeaceae | Nitrosopumilus | unclassified |
| Otu033585 | 1 | 0.6667 | 0.816 | 0.011 | Bacteria | Planctomycetes | Planctomycetia | Pirellulales | Pirellulaceae | unclassified | unclassified |
| Otu035133 | 0.9652 | 1 | 0.982 | 0.001 | Bacteria | Proteobacteria | unclassified | unclassified | unclassified | unclassified | unclassified |
| Otu035352 | 1 | 0.6667 | 0.816 | 0.007 | Archaea | Euryarchaeota | Thermoplasmata | E2 | Marine_group_II | unclassified | unclassified |
| Otu035422 | 0.9878 | 1 | 0.994 | 0.001 | Archaea | Crenarchaeota | Thaumarchaeota | Cenarchaeales | Cenarchaeaceae | Nitrosopumilus | unclassified |
| Otu036260 | 0.9442 | 1 | 0.972 | 0.001 | Bacteria | Proteobacteria | unclassified | unclassified | unclassified | unclassified | unclassified |
| Otu036515 | 0.9553 | 1 | 0.977 | 0.001 | Bacteria | Proteobacteria | Betaproteobacteria | unclassified | unclassified | unclassified | unclassified |
| Otu036533 | 0.9818 | 1 | 0.991 | 0.001 | Bacteria | Proteobacteria | unclassified | unclassified | unclassified | unclassified | unclassified |
| Otu036806 | 0.9291 | 1 | 0.964 | 0.001 | Bacteria | Proteobacteria | unclassified | unclassified | unclassified | unclassified | unclassified |
| Otu036966 | 0.9742 | 1 | 0.987 | 0.001 | Bacteria | Proteobacteria | unclassified | unclassified | unclassified | unclassified | unclassified |
| Otu036999 | 0.9595 | 1 | 0.98 | 0.001 | Bacteria | Proteobacteria | unclassified | unclassified | unclassified | unclassified | unclassified |
| Otu037653 | 0.9041 | 0.6667 | 0.776 | 0.01 | Bacteria | Proteobacteria | Gammaproteobacteria | unclassified | unclassified | unclassified | unclassified |
| Otu039904 | 0.9744 | 1 | 0.987 | 0.001 | Bacteria | Proteobacteria | Betaproteobacteria | EC94 | unclassified | unclassified | unclassified |
| Otu041660 | 0.7803 | 1 | 0.883 | 0.028 | Bacteria | Bacteroidetes | Flavobacteriia | Flavobacteriales | Flavobacteriaceae | unclassified | unclassified |
| Otu042360 | 0.8799 | 1 | 0.938 | 0.003 | Archaea | Crenarchaeota | Thaumarchaeota | Cenarchaeales | Cenarchaeaceae | Nitrosopumilus | unclassified |
| Otu042850 | 1 | 0.6667 | 0.816 | 0.007 | Bacteria | Proteobacteria | Gammaproteobacteria | unclassified | unclassified | unclassified | unclassified |
| Otu043907 | 0.9385 | 1 | 0.969 | 0.001 | Bacteria | Proteobacteria | unclassified | unclassified | unclassified | unclassified | unclassified |
| Otu044038 | 0.9625 | 1 | 0.981 | 0.001 | Bacteria | Proteobacteria | unclassified | unclassified | unclassified | unclassified | unclassified |
| Otu044103 | 0.978 | 1 | 0.989 | 0.001 | Bacteria | Proteobacteria | unclassified | unclassified | unclassified | unclassified | unclassified |
| Otu044221 | 0.941 | 0.6667 | 0.792 | 0.007 | Bacteria | Proteobacteria | unclassified | unclassified | unclassified | unclassified | unclassified |
| Otu044442 | 0.9157 | 1 | 0.957 | 0.002 | Bacteria | Proteobacteria | unclassified | unclassified | unclassified | unclassified | unclassified |
| Otu044453 | 0.91 | 1 | 0.954 | 0.001 | Bacteria | Proteobacteria | unclassified | unclassified | unclassified | unclassified | unclassified |
| Otu044732 | 0.9305 | 1 | 0.965 | 0.001 | Bacteria | Proteobacteria | unclassified | unclassified | unclassified | unclassified | unclassified |
| Otu044935 | 0.9491 | 0.6667 | 0.795 | 0.007 | Bacteria | Proteobacteria | unclassified | unclassified | unclassified | unclassified | unclassified |
| Otu044974 | 0.9199 | 0.6667 | 0.783 | 0.007 | Bacteria | Proteobacteria | Deltaproteobacteria | unclassified | unclassified | unclassified | unclassified |
| Otu045070 | 0.895 | 0.6667 | 0.772 | 0.008 | Bacteria | Proteobacteria | Alphaproteobacteria | unclassified | unclassified | unclassified | unclassified |
| Otu045845 | 0.9698 | 1 | 0.985 | 0.001 | Bacteria | Proteobacteria | unclassified | unclassified | unclassified | unclassified | unclassified |
| Otu045997 | 0.9448 | 1 | 0.972 | 0.001 | Bacteria | Proteobacteria | unclassified | unclassified | unclassified | unclassified | unclassified |
| Otu046002 | 0.9654 | 1 | 0.983 | 0.001 | Bacteria | Proteobacteria | unclassified | unclassified | unclassified | unclassified | unclassified |
| Otu046049 | 0.9773 | 1 | 0.989 | 0.001 | Bacteria | Proteobacteria | unclassified | unclassified | unclassified | unclassified | unclassified |
| Otu047876 | 1 | 0.6667 | 0.816 | 0.007 | Archaea | Crenarchaeota | Thaumarchaeota | Cenarchaeales | Cenarchaeaceae | Nitrosopumilus | unclassified |
| Otu048389 | 0.9664 | 1 | 0.983 | 0.001 | Bacteria | Proteobacteria | unclassified | unclassified | unclassified | unclassified | unclassified |
| Otu048454 | 0.9781 | 1 | 0.989 | 0.001 | Bacteria | Proteobacteria | Gammaproteobacteria | unclassified | unclassified | unclassified | unclassified |

| | | | | | | | | | | | |
|---|---|---|---|---|---|---|---|---|---|---|---|
| Otu048732 | 0.9512 | 1 | 0.975 | 0.001 | Bacteria | Proteobacteria | unclassified | unclassified | unclassified | unclassified | unclassified |
| Otu049930 | 0.9683 | 0.6667 | 0.803 | 0.007 | Bacteria | Proteobacteria | Gammaproteobacteria | Alteromonadales | Alteromonadaceae | HTCC2207 | unclassified |
| Otu050296 | 0.8351 | 0.6667 | 0.746 | 0.041 | Bacteria | Proteobacteria | Gammaproteobacteria | Alteromonadales | Psychromonadaceae | Psychromonas | unclassified |
| Otu053527 | 1 | 0.6667 | 0.816 | 0.007 | Bacteria | Proteobacteria | Gammaproteobacteria | Oceanospirillales | Oleiphilaceae | unclassified | unclassified |
| Otu055242 | 0.8349 | 0.6667 | 0.746 | 0.048 | Bacteria | Proteobacteria | Alphaproteobacteria | Rhodospirillales | Rhodospirillaceae | unclassified | unclassified |
| Otu057716 | 0.8289 | 0.6667 | 0.743 | 0.043 | Bacteria | Proteobacteria | Gammaproteobacteria | Vibrionales | Vibrionaceae | Vibrio | aestuarianus |
| Otu058470 | 0.891 | 0.6667 | 0.771 | 0.019 | Bacteria | Bacteroidetes | Saprospirae | Saprospirales | Saprospiraceae | Saprospira | unclassified |
| Otu059022 | 0.7905 | 0.6667 | 0.726 | 0.049 | Bacteria | Proteobacteria | Alphaproteobacteria | Rhodospirillales | Rhodospirillaceae | Nisaea | unclassified |
| Otu060559 | 0.9148 | 0.6667 | 0.781 | 0.015 | Bacteria | Proteobacteria | Gammaproteobacteria | unclassified | unclassified | unclassified | unclassified |
| Otu064935 | 0.8475 | 0.6667 | 0.752 | 0.047 | Bacteria | Proteobacteria | Gammaproteobacteria | Legionellales | Coxiellaceae | Rickettsiella | unclassified |
| Otu065845 | 0.9773 | 1 | 0.989 | 0.001 | Bacteria | Proteobacteria | Gammaproteobacteria | unclassified | unclassified | unclassified | unclassified |
| Otu066913 | 0.7976 | 0.6667 | 0.729 | 0.04 | Bacteria | Proteobacteria | Gammaproteobacteria | unclassified | unclassified | unclassified | unclassified |
| Otu074145 | 0.8881 | 0.6667 | 0.769 | 0.024 | Bacteria | Proteobacteria | Alphaproteobacteria | Rickettsiales | Pelagibacteraceae | unclassified | unclassified |
| Otu075722 | 0.8085 | 0.6667 | 0.734 | 0.046 | Bacteria | Actinobacteria | Acidimicrobiia | Acidimicrobiales | wb1_P06 | unclassified | unclassified |
| Otu078176 | 0.9578 | 0.6667 | 0.799 | 0.012 | Bacteria | Proteobacteria | Deltaproteobacteria | Sva0853 | SAR324 | unclassified | unclassified |
| Otu080821 | 0.8122 | 0.6667 | 0.736 | 0.041 | Bacteria | Proteobacteria | Alphaproteobacteria | unclassified | unclassified | unclassified | unclassified |
| Otu082432 | 0.8379 | 0.6667 | 0.747 | 0.018 | Bacteria | Proteobacteria | Betaproteobacteria | Methylophilales | Methylophilaceae | unclassified | unclassified |
| Otu082435 | 1 | 0.6667 | 0.816 | 0.007 | Bacteria | Proteobacteria | unclassified | unclassified | unclassified | unclassified | unclassified |
| Otu082869 | 0.9583 | 0.6667 | 0.799 | 0.007 | Bacteria | Proteobacteria | Betaproteobacteria | unclassified | unclassified | unclassified | unclassified |
| Otu086208 | 1 | 0.6667 | 0.816 | 0.01 | Bacteria | Proteobacteria | Gammaproteobacteria | unclassified | unclassified | unclassified | unclassified |
| Otu103149 | 0.8837 | 1 | 0.94 | 0.005 | Bacteria | unclassified | unclassified | unclassified | unclassified | unclassified | unclassified |
| Otu108930 | 0.8562 | 0.6667 | 0.756 | 0.037 | Bacteria | Proteobacteria | Gammaproteobacteria | Alteromonadales | unclassified | unclassified | unclassified |
| Otu111290 | 0.9672 | 1 | 0.983 | 0.001 | Bacteria | Proteobacteria | unclassified | unclassified | unclassified | unclassified | unclassified |
| Otu112110 | 0.962 | 1 | 0.981 | 0.001 | Bacteria | Proteobacteria | unclassified | unclassified | unclassified | unclassified | unclassified |
| Otu112607 | 0.9565 | 1 | 0.978 | 0.001 | Bacteria | Proteobacteria | unclassified | unclassified | unclassified | unclassified | unclassified |
| Otu112615 | 0.9651 | 1 | 0.982 | 0.001 | Bacteria | Proteobacteria | unclassified | unclassified | unclassified | unclassified | unclassified |
| Otu113512 | 0.9682 | 1 | 0.984 | 0.001 | Bacteria | Proteobacteria | unclassified | unclassified | unclassified | unclassified | unclassified |
| Otu114932 | 0.9565 | 1 | 0.978 | 0.001 | Bacteria | Proteobacteria | unclassified | unclassified | unclassified | unclassified | unclassified |
| Otu115203 | 0.9799 | 1 | 0.99 | 0.001 | Bacteria | Proteobacteria | unclassified | unclassified | unclassified | unclassified | unclassified |
| Otu116590 | 0.8591 | 0.6667 | 0.757 | 0.018 | Bacteria | Bacteroidetes | Flavobacteriia | Flavobacteriales | unclassified | unclassified | unclassified |
| Otu116886 | 0.8056 | 0.6667 | 0.733 | 0.038 | Bacteria | Bacteroidetes | Flavobacteriia | Flavobacteriales | Flavobacteriaceae | unclassified | unclassified |
| Otu117280 | 0.9332 | 1 | 0.966 | 0.003 | Bacteria | Proteobacteria | Gammaproteobacteria | unclassified | unclassified | unclassified | unclassified |
| Otu119038 | 1 | 0.6667 | 0.816 | 0.007 | Bacteria | Bacteroidetes | unclassified | unclassified | unclassified | unclassified | unclassified |
| Otu119816 | 0.8572 | 0.6667 | 0.756 | 0.019 | Bacteria | Bacteroidetes | Flavobacteriia | Flavobacteriales | Cryomorphaceae | Fluviicola | unclassified |
| Otu123464 | 0.7698 | 0.6667 | 0.716 | 0.024 | Bacteria | Proteobacteria | Gammaproteobacteria | Thiohalorhabdales | Thiohalorhabdaceae | unclassified | unclassified |
| Otu124114 | 0.7601 | 1 | 0.872 | 0.008 | Bacteria | Proteobacteria | unclassified | unclassified | unclassified | unclassified | unclassified |
| Otu125440 | 0.8826 | 0.6667 | 0.767 | 0.019 | Bacteria | Proteobacteria | Deltaproteobacteria | Bdellovibrionales | Bacteriovoracaceae | Bacteriovorax | unclassified |
| Otu125598 | 0.8496 | 0.6667 | 0.753 | 0.035 | Bacteria | Proteobacteria | Gammaproteobacteria | Alteromonadales | unclassified | unclassified | unclassified |
| Otu125685 | 0.8373 | 0.6667 | 0.747 | 0.038 | Bacteria | Planctomycetes | Planctomycetia | Planctomycetales | Planctomycetaceae | Planctomyces | unclassified |
| Otu126478 | 0.9347 | 0.6667 | 0.789 | 0.008 | Bacteria | Proteobacteria | Gammaproteobacteria | Alteromonadales | unclassified | unclassified | unclassified |
| Otu130338 | 1 | 0.6667 | 0.816 | 0.007 | Bacteria | Proteobacteria | Gammaproteobacteria | unclassified | unclassified | unclassified | unclassified |
| Otu130491 | 0.8214 | 0.6667 | 0.74 | 0.04 | Bacteria | Proteobacteria | Gammaproteobacteria | HTCC2188 | HTCC2089 | unclassified | unclassified |
| Otu130640 | 0.8357 | 0.6667 | 0.746 | 0.03 | Bacteria | Proteobacteria | Alphaproteobacteria | Ellin329 | unclassified | unclassified | unclassified |
| Otu133790 | 0.8683 | 0.6667 | 0.761 | 0.026 | Bacteria | Verrucomicrobia | Pedosphaerae | Arctic97B-4 | unclassified | unclassified | unclassified |
| Otu134119 | 0.947 | 0.6667 | 0.795 | 0.01 | Bacteria | Proteobacteria | Alphaproteobacteria | Rhodobacterales | Rhodobacteraceae | Octadecabacter | unclassified |
| Otu134953 | 0.9704 | 1 | 0.985 | 0.001 | Bacteria | Proteobacteria | unclassified | unclassified | unclassified | unclassified | unclassified |
| Otu135934 | 0.9761 | 1 | 0.988 | 0.001 | Bacteria | Proteobacteria | unclassified | unclassified | unclassified | unclassified | unclassified |
| Otu136036 | 0.9888 | 1 | 0.994 | 0.001 | Bacteria | Proteobacteria | unclassified | unclassified | unclassified | unclassified | unclassified |
| Otu136885 | 0.9559 | 1 | 0.978 | 0.001 | Bacteria | Proteobacteria | unclassified | unclassified | unclassified | unclassified | unclassified |
| Otu137171 | 0.9706 | 1 | 0.985 | 0.001 | Bacteria | Proteobacteria | unclassified | unclassified | unclassified | unclassified | unclassified |
| Otu137245 | 0.9683 | 1 | 0.984 | 0.001 | Bacteria | Proteobacteria | unclassified | unclassified | unclassified | unclassified | unclassified |
| Otu137628 | 0.9677 | 1 | 0.984 | 0.001 | Bacteria | Proteobacteria | unclassified | unclassified | unclassified | unclassified | unclassified |
| Otu138268 | 0.965 | 1 | 0.982 | 0.001 | Bacteria | Proteobacteria | Gammaproteobacteria | unclassified | unclassified | unclassified | unclassified |
| Otu138514 | 0.9664 | 1 | 0.983 | 0.001 | Bacteria | Proteobacteria | unclassified | unclassified | unclassified | unclassified | unclassified |
| Otu139058 | 0.7885 | 0.6667 | 0.725 | 0.035 | Bacteria | Bacteroidetes | Cytophagia | Cytophagales | Flammeovirgaceae | JTB248 | unclassified |
| Otu140069 | 0.9749 | 1 | 0.987 | 0.001 | Bacteria | Proteobacteria | unclassified | unclassified | unclassified | unclassified | unclassified |
| Otu140449 | 0.8721 | 0.6667 | 0.762 | 0.012 | Bacteria | unclassified | unclassified | unclassified | unclassified | unclassified | unclassified |
| Otu141316 | 0.9527 | 1 | 0.976 | 0.001 | Bacteria | Proteobacteria | unclassified | unclassified | unclassified | unclassified | unclassified |
| Otu144040 | 0.9065 | 0.6667 | 0.777 | 0.019 | Bacteria | Bacteroidetes | Flavobacteriia | Flavobacteriales | unclassified | unclassified | unclassified |
| Otu148441 | 0.9715 | 1 | 0.986 | 0.001 | Bacteria | Proteobacteria | unclassified | unclassified | unclassified | unclassified | unclassified |
| Otu156948 | 0.8318 | 0.6667 | 0.745 | 0.02 | Bacteria | Planctomycetes | Planctomycetia | Planctomycetales | Planctomycetaceae | Planctomyces | unclassified |
| Otu167491 | 0.9797 | 1 | 0.99 | 0.001 | Bacteria | Proteobacteria | unclassified | unclassified | unclassified | unclassified | unclassified |
| Otu167633 | 0.9828 | 1 | 0.991 | 0.001 | Bacteria | Proteobacteria | unclassified | unclassified | unclassified | unclassified | unclassified |
| Otu175422 | 0.8929 | 0.6667 | 0.772 | 0.012 | Bacteria | Proteobacteria | unclassified | unclassified | unclassified | unclassified | unclassified |
| Otu178604 | 0.8908 | 0.6667 | 0.771 | 0.018 | Bacteria | Proteobacteria | Gammaproteobacteria | unclassified | unclassified | unclassified | unclassified |
| Otu179882 | 0.9781 | 1 | 0.989 | 0.001 | Bacteria | Proteobacteria | Gammaproteobacteria | unclassified | unclassified | unclassified | unclassified |
| Otu184713 | 0.9567 | 1 | 0.978 | 0.001 | Bacteria | Proteobacteria | unclassified | unclassified | unclassified | unclassified | unclassified |
| Otu189017 | 0.9649 | 1 | 0.982 | 0.001 | Bacteria | Proteobacteria | unclassified | unclassified | unclassified | unclassified | unclassified |
| Otu189060 | 0.9877 | 1 | 0.994 | 0.001 | Bacteria | Proteobacteria | unclassified | unclassified | unclassified | unclassified | unclassified |
| Otu189253 | 0.9472 | 1 | 0.973 | 0.001 | Bacteria | Proteobacteria | unclassified | unclassified | unclassified | unclassified | unclassified |
| Otu194846 | 0.9537 | 1 | 0.977 | 0.001 | Bacteria | unclassified | unclassified | unclassified | unclassified | unclassified | unclassified |
| Otu195617 | 1 | 0.6667 | 0.816 | 0.007 | Bacteria | Bacteroidetes | Flavobacteriia | Flavobacteriales | Cryomorphaceae | Crocinitomix | unclassified |
| Otu196299 | 0.9706 | 1 | 0.985 | 0.001 | Bacteria | Proteobacteria | unclassified | unclassified | unclassified | unclassified | unclassified |
| Otu200135 | 0.9798 | 1 | 0.99 | 0.001 | Bacteria | Proteobacteria | unclassified | unclassified | unclassified | unclassified | unclassified |
| Otu201196 | 0.9714 | 1 | 0.986 | 0.001 | Bacteria | Proteobacteria | unclassified | unclassified | unclassified | unclassified | unclassified |
| Otu203429 | 0.834 | 0.6667 | 0.746 | 0.037 | Bacteria | Proteobacteria | Deltaproteobacteria | Desulfobacterales | Nitrospinaceae | Nitrospina | unclassified |
| Otu215056 | 0.8359 | 0.6667 | 0.746 | 0.05 | Bacteria | Proteobacteria | Gammaproteobacteria | Oceanospirillales | Halomonadaceae | Candidatus_Portiera | unclassified |
| Otu251985 | 0.9382 | 0.6667 | 0.791 | 0.015 | Bacteria | Proteobacteria | Gammaproteobacteria | HTCC2188 | HTCC2089 | unclassified | unclassified |
| Otu319725 | 0.8913 | 0.6667 | 0.771 | 0.008 | Archaea | Euryarchaeota | Thermoplasmata | E2 | Marine_group_II | unclassified | unclassified |

| **0.03 Otus** | ***Axinella infundibulifor*** | **Rest** | **stat** | ***p value*** | **Domain** | **Phylum** | **Class** | **Order** | **Family** | **Genus** | **Species** |
|---|---|---|---|---|---|---|---|---|---|---|---|
| Otu000144 | 0.9793 | 1 | 0.99 | 0.001 | Bacteria | Nitrospirae | Nitrospira | Nitrospirales | Nitrospiraceae | unclassified | unclassified |
| Otu000165 | 0.9943 | 1 | 0.997 | 0.001 | Bacteria | Proteobacteria | Gammaproteobacteria | unclassified | unclassified | unclassified | unclassified |
| Otu000215 | 1 | 0.6667 | 0.816 | 0.001 | Bacteria | Nitrospirae | Nitrospira | Nitrospirales | Nitrospiraceae | unclassified | unclassified |
| Otu000723 | 1 | 0.5 | 0.707 | 0.01 | Bacteria | Proteobacteria | Gammaproteobacteria | unclassified | unclassified | unclassified | unclassified |
| Otu000729 | 0.8884 | 1 | 0.943 | 0.001 | Archaea | Crenarchaeota | Thaumarchaeota | Cenarchaeales | Cenarchaeaceae | Nitrosopumilus | unclassified |
| Otu000742 | 0.9135 | 1 | 0.956 | 0.001 | Archaea | Crenarchaeota | Thaumarchaeota | Cenarchaeales | Cenarchaeaceae | Nitrosopumilus | unclassified |
| Otu000838 | 0.7323 | 1 | 0.856 | 0.01 | Bacteria | Proteobacteria | Alphaproteobacteria | Rhodobacterales | Rhodobacteraceae | unclassified | unclassified |
| Otu001374 | 0.9947 | 1 | 0.997 | 0.001 | Bacteria | Actinobacteria | Acidimicrobiia | Acidimicrobiales | TK06 | unclassified | unclassified |
| Otu001422 | 0.8355 | 0.6667 | 0.746 | 0.01 | Archaea | Crenarchaeota | Thaumarchaeota | Cenarchaeales | Cenarchaeaceae | Nitrosopumilus | unclassified |
| Otu002028 | 0.7405 | 0.8333 | 0.786 | 0.042 | Bacteria | Proteobacteria | Gammaproteobacteria | Alteromonadales | HTCC2188 | HTCC | unclassified |
| Otu002418 | 0.9099 | 0.8333 | 0.871 | 0.002 | Archaea | Crenarchaeota | Thaumarchaeota | Cenarchaeales | Cenarchaeaceae | Nitrosopumilus | unclassified |
| Otu002431 | 0.9209 | 0.5 | 0.679 | 0.014 | Bacteria | Cyanobacteria | Synechococcophycideae | Synechococcales | Synechococcaceae | unclassified | unclassified |
| Otu002524 | 0.8493 | 0.6667 | 0.752 | 0.008 | Archaea | Crenarchaeota | Thaumarchaeota | Cenarchaeales | Cenarchaeaceae | Nitrosopumilus | unclassified |
| Otu002659 | 1 | 0.3333 | 0.577 | 0.043 | Bacteria | Verrucomicrobia | Verrucomicrobiae | Verrucomicrobiales | Verrucomicrobiaceae | Persicirhabdus | unclassified |
| Otu002707 | 1 | 0.8333 | 0.913 | 0.001 | Bacteria | Proteobacteria | Gammaproteobacteria | unclassified | unclassified | unclassified | unclassified |
| Otu003072 | 0.9164 | 1 | 0.957 | 0.001 | Archaea | Crenarchaeota | Thaumarchaeota | Cenarchaeales | Cenarchaeaceae | Nitrosopumilus | unclassified |
| Otu003184 | 0.9792 | 1 | 0.99 | 0.001 | Bacteria | Nitrospirae | Nitrospira | Nitrospirales | Nitrospiraceae | unclassified | unclassified |
| Otu003242 | 1 | 1 | 1 | 0.001 | Bacteria | unclassified | unclassified | unclassified | unclassified | unclassified | unclassified |
| Otu003584 | 0.8238 | 1 | 0.908 | 0.001 | Archaea | Crenarchaeota | Thaumarchaeota | Cenarchaeales | Cenarchaeaceae | Nitrosopumilus | unclassified |
| Otu003602 | 0.8526 | 1 | 0.923 | 0.001 | Archaea | Crenarchaeota | Thaumarchaeota | Cenarchaeales | Cenarchaeaceae | Nitrosopumilus | unclassified |

| | | | | | | | | | | | |
|---|---|---|---|---|---|---|---|---|---|---|---|
| Otu003632 | 0.9852 | 1 | 0.993 | 0.001 | Bacteria | Proteobacteria | Gammaproteobacteria | unclassified | unclassified | unclassified | unclassified |
| Otu003666 | 1 | 1 | 1 | 0.001 | Bacteria | Bacteroidetes | unclassified | unclassified | unclassified | unclassified | unclassified |
| Otu003921 | 0.8201 | 0.5 | 0.64 | 0.034 | Bacteria | Proteobacteria | Gammaproteobacteria | unclassified | unclassified | unclassified | unclassified |
| Otu004011 | 1 | 1 | 1 | 0.001 | Bacteria | Bacteroidetes | unclassified | unclassified | unclassified | unclassified | unclassified |
| Otu004330 | 1 | 0.3333 | 0.577 | 0.041 | Bacteria | Proteobacteria | unclassified | unclassified | unclassified | unclassified | unclassified |
| Otu004634 | 0.8765 | 0.8333 | 0.855 | 0.003 | Bacteria | Proteobacteria | Gammaproteobacteria | unclassified | unclassified | unclassified | unclassified |
| Otu004669 | 1 | 1 | 1 | 0.001 | Bacteria | Proteobacteria | Gammaproteobacteria | unclassified | unclassified | unclassified | unclassified |
| Otu004696 | 1 | 0.5 | 0.707 | 0.003 | Bacteria | Proteobacteria | unclassified | unclassified | unclassified | unclassified | unclassified |
| Otu004811 | 1 | 0.3333 | 0.577 | 0.041 | Bacteria | Proteobacteria | Gammaproteobacteria | unclassified | unclassified | unclassified | unclassified |
| Otu005015 | 1 | 0.5 | 0.707 | 0.009 | Bacteria | Verrucomicrobia | Pedosphaerae | Pedosphaerales | unclassified | unclassified | unclassified |
| Otu005116 | 1 | 0.6667 | 0.816 | 0.001 | Bacteria | Proteobacteria | Alphaproteobacteria | unclassified | unclassified | unclassified | unclassified |
| Otu005137 | 1 | 0.5 | 0.707 | 0.004 | Bacteria | Proteobacteria | Gammaproteobacteria | unclassified | unclassified | unclassified | unclassified |
| Otu005164 | 0.8295 | 0.6667 | 0.744 | 0.008 | Archaea | Crenarchaeota | Thaumarchaeota | Cenarchaeales | Cenarchaeaceae | Nitrosopumilus | unclassified |
| Otu005169 | 0.8317 | 1 | 0.912 | 0.001 | Archaea | Crenarchaeota | Thaumarchaeota | Cenarchaeales | Cenarchaeaceae | Nitrosopumilus | unclassified |
| Otu005177 | 1 | 1 | 1 | 0.001 | Bacteria | Spirochaetes | Spirochaetes | unclassified | unclassified | unclassified | unclassified |
| Otu005366 | 1 | 0.3333 | 0.577 | 0.049 | Bacteria | Proteobacteria | unclassified | unclassified | unclassified | unclassified | unclassified |
| Otu005402 | 1 | 0.3333 | 0.577 | 0.049 | Bacteria | Proteobacteria | Deltaproteobacteria | Spirobacillales | unclassified | unclassified | unclassified |
| Otu005425 | 0.8148 | 0.6667 | 0.737 | 0.008 | Archaea | Crenarchaeota | Thaumarchaeota | Cenarchaeales | Cenarchaeaceae | Nitrosopumilus | unclassified |
| Otu005609 | 0.9412 | 0.5 | 0.686 | 0.022 | Bacteria | Proteobacteria | Gammaproteobacteria | unclassified | unclassified | unclassified | unclassified |
| Otu006211 | 1 | 1 | 1 | 0.001 | Bacteria | Proteobacteria | Gammaproteobacteria | unclassified | unclassified | unclassified | unclassified |
| Otu006316 | 0.8791 | 1 | 0.938 | 0.001 | Archaea | Crenarchaeota | Thaumarchaeota | Cenarchaeales | Cenarchaeaceae | Nitrosopumilus | unclassified |
| Otu006387 | 0.7941 | 0.6667 | 0.728 | 0.022 | Archaea | Crenarchaeota | Thaumarchaeota | Cenarchaeales | Cenarchaeaceae | Nitrosopumilus | unclassified |
| Otu006521 | 1 | 0.3333 | 0.577 | 0.037 | Bacteria | Proteobacteria | Gammaproteobacteria | unclassified | unclassified | unclassified | unclassified |
| Otu006577 | 1 | 1 | 1 | 0.001 | Bacteria | Proteobacteria | Alphaproteobacteria | unclassified | unclassified | unclassified | unclassified |
| Otu006886 | 0.8521 | 1 | 0.923 | 0.001 | Archaea | Crenarchaeota | Thaumarchaeota | Cenarchaeales | Cenarchaeaceae | Nitrosopumilus | unclassified |
| Otu007001 | 0.819 | 1 | 0.905 | 0.003 | Archaea | Crenarchaeota | Thaumarchaeota | Cenarchaeales | Cenarchaeaceae | Nitrosopumilus | unclassified |
| Otu007093 | 0.9552 | 0.5 | 0.691 | 0.004 | Archaea | Crenarchaeota | Thaumarchaeota | Cenarchaeales | Cenarchaeaceae | Nitrosopumilus | unclassified |
| Otu007469 | 0.9269 | 0.5 | 0.681 | 0.009 | Bacteria | Cyanobacteria | Synechococcophycideae | Synechococcales | Synechococcaceae | unclassified | unclassified |
| Otu007812 | 0.8256 | 0.8333 | 0.829 | 0.003 | Archaea | Crenarchaeota | Thaumarchaeota | Cenarchaeales | Cenarchaeaceae | Nitrosopumilus | unclassified |
| Otu007904 | 0.914 | 0.6667 | 0.781 | 0.003 | Archaea | Crenarchaeota | Thaumarchaeota | Cenarchaeales | Cenarchaeaceae | Nitrosopumilus | unclassified |
| Otu007911 | 0.8479 | 1 | 0.921 | 0.001 | Archaea | Crenarchaeota | Thaumarchaeota | Cenarchaeales | Cenarchaeaceae | Nitrosopumilus | unclassified |
| Otu008011 | 1 | 0.3333 | 0.577 | 0.049 | Bacteria | Verrucomicrobia | Verrucomicrobiae | Verrucomicrobiales | Verrucomicrobiaceae | unclassified | unclassified |
| Otu008196 | 0.8495 | 1 | 0.922 | 0.001 | Archaea | Crenarchaeota | Thaumarchaeota | Cenarchaeales | Cenarchaeaceae | Nitrosopumilus | unclassified |
| Otu008208 | 0.8426 | 1 | 0.918 | 0.001 | Archaea | Crenarchaeota | Thaumarchaeota | Cenarchaeales | Cenarchaeaceae | Nitrosopumilus | unclassified |
| Otu008239 | 0.9338 | 0.3333 | 0.558 | 0.042 | Bacteria | Proteobacteria | Deltaproteobacteria | Desulfobacterales | Desulfobacteraceae | Desulfococcus | unclassified |
| Otu008256 | 0.8669 | 0.6667 | 0.76 | 0.017 | Bacteria | Proteobacteria | Alphaproteobacteria | Rhodospirillales | Rhodospirillaceae | unclassified | unclassified |
| Otu008876 | 0.829 | 0.8333 | 0.831 | 0.002 | Archaea | Crenarchaeota | Thaumarchaeota | Cenarchaeales | Cenarchaeaceae | Nitrosopumilus | unclassified |
| Otu008885 | 0.944 | 0.5 | 0.687 | 0.013 | Archaea | Crenarchaeota | Thaumarchaeota | Cenarchaeales | Cenarchaeaceae | Nitrosopumilus | unclassified |
| Otu008996 | 0.9201 | 0.8333 | 0.876 | 0.001 | Archaea | Crenarchaeota | Thaumarchaeota | Cenarchaeales | Cenarchaeaceae | Nitrosopumilus | unclassified |
| Otu009043 | 0.8403 | 0.8333 | 0.837 | 0.003 | Archaea | Crenarchaeota | Thaumarchaeota | Cenarchaeales | Cenarchaeaceae | Nitrosopumilus | unclassified |
| Otu009079 | 0.8723 | 1 | 0.934 | 0.001 | Archaea | Crenarchaeota | Thaumarchaeota | Cenarchaeales | Cenarchaeaceae | Nitrosopumilus | unclassified |
| Otu009086 | 0.8621 | 1 | 0.929 | 0.001 | Archaea | Crenarchaeota | Thaumarchaeota | Cenarchaeales | Cenarchaeaceae | Nitrosopumilus | unclassified |
| Otu009141 | 1 | 0.8333 | 0.913 | 0.001 | Bacteria | Proteobacteria | Gammaproteobacteria | unclassified | unclassified | unclassified | unclassified |
| Otu009308 | 0.8843 | 0.8333 | 0.858 | 0.001 | Archaea | Crenarchaeota | Thaumarchaeota | Cenarchaeales | Cenarchaeaceae | Nitrosopumilus | unclassified |
| Otu009768 | 0.8556 | 1 | 0.925 | 0.001 | Archaea | Crenarchaeota | Thaumarchaeota | Cenarchaeales | Cenarchaeaceae | Nitrosopumilus | unclassified |
| Otu009843 | 0.9172 | 0.8333 | 0.874 | 0.002 | Archaea | Crenarchaeota | Thaumarchaeota | Cenarchaeales | Cenarchaeaceae | Nitrosopumilus | unclassified |
| Otu009847 | 0.8463 | 0.6667 | 0.751 | 0.007 | Archaea | Crenarchaeota | Thaumarchaeota | Cenarchaeales | Cenarchaeaceae | Nitrosopumilus | unclassified |
| Otu009849 | 0.8477 | 1 | 0.921 | 0.002 | Archaea | Crenarchaeota | Thaumarchaeota | Cenarchaeales | Cenarchaeaceae | Nitrosopumilus | unclassified |
| Otu009854 | 0.864 | 1 | 0.929 | 0.001 | Archaea | Crenarchaeota | Thaumarchaeota | Cenarchaeales | Cenarchaeaceae | Nitrosopumilus | unclassified |
| Otu010111 | 0.8509 | 0.6667 | 0.753 | 0.01 | Archaea | Crenarchaeota | Thaumarchaeota | Cenarchaeales | Cenarchaeaceae | Nitrosopumilus | unclassified |
| Otu010163 | 0.6678 | 0.8333 | 0.746 | 0.046 | Bacteria | Proteobacteria | Gammaproteobacteria | Thiotrichales | Piscirickettsiaceae | unclassified | unclassified |
| Otu010311 | 0.79 | 1 | 0.889 | 0.003 | Archaea | Crenarchaeota | Thaumarchaeota | Cenarchaeales | Cenarchaeaceae | Nitrosopumilus | unclassified |
| Otu010354 | 1 | 0.8333 | 0.913 | 0.001 | Bacteria | Proteobacteria | Deltaproteobacteria | Bdellovibrionales | Bdellovibrionaceae | Bdellovibrio | unclassified |
| Otu010379 | 0.8786 | 0.8333 | 0.856 | 0.001 | Archaea | Crenarchaeota | Thaumarchaeota | Cenarchaeales | Cenarchaeaceae | Nitrosopumilus | unclassified |
| Otu010426 | 0.8787 | 0.8333 | 0.856 | 0.001 | Archaea | Crenarchaeota | Thaumarchaeota | Cenarchaeales | Cenarchaeaceae | Nitrosopumilus | unclassified |
| Otu010576 | 1 | 0.8333 | 0.913 | 0.001 | Bacteria | Proteobacteria | Gammaproteobacteria | unclassified | unclassified | unclassified | unclassified |
| Otu010678 | 1 | 0.8333 | 0.913 | 0.001 | Bacteria | Proteobacteria | Gammaproteobacteria | unclassified | unclassified | unclassified | unclassified |
| Otu010818 | 0.8311 | 0.8333 | 0.832 | 0.009 | Bacteria | Proteobacteria | Alphaproteobacteria | unclassified | unclassified | unclassified | unclassified |
| Otu011015 | 1 | 0.8333 | 0.913 | 0.001 | Bacteria | Proteobacteria | Gammaproteobacteria | unclassified | unclassified | unclassified | unclassified |
| Otu011060 | 0.9883 | 0.5 | 0.703 | 0.006 | Bacteria | Proteobacteria | unclassified | unclassified | unclassified | unclassified | unclassified |
| Otu011211 | 1 | 0.8333 | 0.913 | 0.001 | Bacteria | Proteobacteria | Gammaproteobacteria | unclassified | unclassified | unclassified | unclassified |
| Otu011429 | 0.8369 | 0.6667 | 0.747 | 0.01 | Archaea | Crenarchaeota | Thaumarchaeota | Cenarchaeales | Cenarchaeaceae | Nitrosopumilus | unclassified |
| Otu011440 | 0.8525 | 1 | 0.923 | 0.001 | Archaea | Crenarchaeota | Thaumarchaeota | Cenarchaeales | Cenarchaeaceae | Nitrosopumilus | unclassified |
| Otu011675 | 1 | 1 | 1 | 0.001 | Bacteria | Proteobacteria | Gammaproteobacteria | unclassified | unclassified | unclassified | unclassified |
| Otu011705 | 1 | 0.8333 | 0.913 | 0.001 | Bacteria | Proteobacteria | Gammaproteobacteria | unclassified | unclassified | unclassified | unclassified |
| Otu011795 | 1 | 1 | 1 | 0.001 | Bacteria | Proteobacteria | Gammaproteobacteria | unclassified | unclassified | unclassified | unclassified |
| Otu011820 | 1 | 0.5 | 0.707 | 0.013 | Bacteria | Proteobacteria | Gammaproteobacteria | unclassified | unclassified | unclassified | unclassified |
| Otu011841 | 1 | 1 | 1 | 0.001 | Bacteria | Proteobacteria | Gammaproteobacteria | unclassified | unclassified | unclassified | unclassified |
| Otu011857 | 1 | 0.8333 | 0.913 | 0.001 | Bacteria | Proteobacteria | Gammaproteobacteria | unclassified | unclassified | unclassified | unclassified |
| Otu011946 | 0.8374 | 0.5 | 0.647 | 0.026 | Bacteria | Proteobacteria | Alphaproteobacteria | unclassified | unclassified | unclassified | unclassified |
| Otu011993 | 0.7897 | 0.5 | 0.628 | 0.038 | Bacteria | Proteobacteria | Gammaproteobacteria | Alteromonadales | OM60 | unclassified | unclassified |
| Otu012171 | 1 | 1 | 1 | 0.001 | Bacteria | Proteobacteria | Gammaproteobacteria | unclassified | unclassified | unclassified | unclassified |
| Otu012295 | 0.9421 | 0.3333 | 0.56 | 0.043 | Archaea | Crenarchaeota | Thaumarchaeota | Cenarchaeales | Cenarchaeaceae | Nitrosopumilus | unclassified |
| Otu012309 | 0.8381 | 1 | 0.915 | 0.002 | Archaea | Crenarchaeota | Thaumarchaeota | Cenarchaeales | Cenarchaeaceae | Nitrosopumilus | unclassified |
| Otu012324 | 0.8643 | 0.8333 | 0.849 | 0.004 | Archaea | Crenarchaeota | Thaumarchaeota | Cenarchaeales | Cenarchaeaceae | Nitrosopumilus | unclassified |
| Otu012362 | 0.8195 | 1 | 0.905 | 0.001 | Archaea | Crenarchaeota | Thaumarchaeota | Cenarchaeales | Cenarchaeaceae | Nitrosopumilus | unclassified |
| Otu012514 | 1 | 0.3333 | 0.577 | 0.049 | Bacteria | Proteobacteria | unclassified | unclassified | unclassified | unclassified | unclassified |
| Otu012705 | 1 | 1 | 1 | 0.001 | Bacteria | unclassified | unclassified | unclassified | unclassified | unclassified | unclassified |
| Otu012751 | 0.8472 | 0.5 | 0.651 | 0.015 | Archaea | Crenarchaeota | Thaumarchaeota | Cenarchaeales | Cenarchaeaceae | Nitrosopumilus | unclassified |
| Otu012898 | 0.9516 | 0.6667 | 0.797 | 0.002 | Archaea | Crenarchaeota | Thaumarchaeota | Cenarchaeales | Cenarchaeaceae | Nitrosopumilus | unclassified |
| Otu013185 | 0.774 | 0.8333 | 0.803 | 0.016 | Bacteria | Planctomycetes | Phycisphaerae | Phycisphaerales | unclassified | unclassified | unclassified |
| Otu013224 | 1 | 0.8333 | 0.913 | 0.001 | Bacteria | Proteobacteria | Gammaproteobacteria | unclassified | unclassified | unclassified | unclassified |
| Otu013513 | 0.9514 | 0.5 | 0.69 | 0.009 | Bacteria | Proteobacteria | Gammaproteobacteria | Enterobacteriales | Enterobacteriaceae | unclassified | unclassified |
| Otu013540 | 0.8379 | 0.6667 | 0.747 | 0.01 | Archaea | Crenarchaeota | Thaumarchaeota | Cenarchaeales | Cenarchaeaceae | Nitrosopumilus | unclassified |
| Otu013576 | 1 | 1 | 1 | 0.001 | Bacteria | Proteobacteria | Gammaproteobacteria | unclassified | unclassified | unclassified | unclassified |
| Otu013578 | 0.9808 | 0.8333 | 0.904 | 0.001 | Bacteria | Proteobacteria | Gammaproteobacteria | unclassified | unclassified | unclassified | unclassified |
| Otu013658 | 0.8834 | 1 | 0.94 | 0.001 | Archaea | Crenarchaeota | Thaumarchaeota | Cenarchaeales | Cenarchaeaceae | Nitrosopumilus | unclassified |
| Otu013681 | 0.9868 | 1 | 0.993 | 0.001 | Bacteria | Proteobacteria | Gammaproteobacteria | unclassified | unclassified | unclassified | unclassified |
| Otu013840 | 1 | 1 | 1 | 0.001 | Bacteria | Proteobacteria | Gammaproteobacteria | unclassified | unclassified | unclassified | unclassified |
| Otu013849 | 0.8839 | 0.8333 | 0.858 | 0.002 | Archaea | Crenarchaeota | Thaumarchaeota | Cenarchaeales | Cenarchaeaceae | Nitrosopumilus | unclassified |
| Otu013979 | 0.9675 | 0.5 | 0.696 | 0.009 | Bacteria | Proteobacteria | Gammaproteobacteria | Vibrionales | Vibrionaceae | Photobacterium | angustum |
| Otu013980 | 0.9241 | 0.3333 | 0.555 | 0.049 | Bacteria | Proteobacteria | Gammaproteobacteria | Vibrionales | Vibrionaceae | Vibrio | unclassified |
| Otu014238 | 1 | 0.6667 | 0.816 | 0.001 | Bacteria | Proteobacteria | Gammaproteobacteria | unclassified | unclassified | unclassified | unclassified |
| Otu014309 | 0.7481 | 0.8333 | 0.79 | 0.016 | Bacteria | Proteobacteria | Gammaproteobacteria | Oceanospirillales | unclassified | unclassified | unclassified |
| Otu014430 | 1 | 0.3333 | 0.577 | 0.045 | Bacteria | Proteobacteria | Gammaproteobacteria | unclassified | unclassified | unclassified | unclassified |
| Otu014452 | 1 | 1 | 1 | 0.001 | Bacteria | Proteobacteria | Gammaproteobacteria | unclassified | unclassified | unclassified | unclassified |
| Otu014468 | 0.8685 | 0.6667 | 0.761 | 0.024 | Bacteria | Proteobacteria | Gammaproteobacteria | unclassified | unclassified | unclassified | unclassified |
| Otu014541 | 0.8279 | 0.6667 | 0.743 | 0.011 | Archaea | Crenarchaeota | Thaumarchaeota | Cenarchaeales | Cenarchaeaceae | Nitrosopumilus | unclassified |
| Otu014589 | 0.8714 | 1 | 0.933 | 0.001 | Archaea | Crenarchaeota | Thaumarchaeota | Cenarchaeales | Cenarchaeaceae | Nitrosopumilus | unclassified |
| Otu014643 | 0.9496 | 0.5 | 0.689 | 0.009 | Archaea | Crenarchaeota | Thaumarchaeota | Cenarchaeales | Cenarchaeaceae | Nitrosopumilus | unclassified |

| | | | | | | | | | | | |
|---|---|---|---|---|---|---|---|---|---|---|---|
| Otu014775 | 1 | 0.5 | 0.707 | 0.009 | Bacteria | Proteobacteria | Gammaproteobacteria | unclassified | unclassified | unclassified | unclassified |
| Otu014833 | 0.8035 | 0.8333 | 0.818 | 0.006 | Archaea | Crenarchaeota | Thaumarchaeota | Cenarchaeales | Cenarchaeaceae | Nitrosopumilus | unclassified |
| Otu014951 | 0.8358 | 1 | 0.914 | 0.001 | Archaea | Crenarchaeota | Thaumarchaeota | Cenarchaeales | Cenarchaeaceae | Nitrosopumilus | unclassified |
| Otu014974 | 0.7864 | 0.6667 | 0.724 | 0.016 | Archaea | Crenarchaeota | Thaumarchaeota | Cenarchaeales | Cenarchaeaceae | Nitrosopumilus | unclassified |
| Otu015014 | 0.9213 | 0.5 | 0.679 | 0.012 | unknown | unclassified | unclassified | unclassified | unclassified | unclassified | unclassified |
| Otu015104 | 0.8607 | 0.5 | 0.656 | 0.027 | Bacteria | Proteobacteria | Alphaproteobacteria | Rhodobacterales | Rhodobacteraceae | unclassified | unclassified |
| Otu015114 | 0.946 | 0.6667 | 0.794 | 0.001 | Archaea | Crenarchaeota | Thaumarchaeota | Cenarchaeales | Cenarchaeaceae | Nitrosopumilus | unclassified |
| Otu015156 | 0.9 | 0.8333 | 0.866 | 0.002 | Archaea | Crenarchaeota | Thaumarchaeota | Cenarchaeales | Cenarchaeaceae | Nitrosopumilus | unclassified |
| Otu015221 | 0.7596 | 0.6667 | 0.712 | 0.033 | Archaea | Crenarchaeota | Thaumarchaeota | Cenarchaeales | Cenarchaeaceae | Nitrosopumilus | unclassified |
| Otu015239 | 0.7934 | 0.8333 | 0.813 | 0.006 | Archaea | Crenarchaeota | Thaumarchaeota | Cenarchaeales | Cenarchaeaceae | Nitrosopumilus | unclassified |
| Otu015370 | 0.8195 | 1 | 0.905 | 0.001 | Archaea | Crenarchaeota | Thaumarchaeota | Cenarchaeales | Cenarchaeaceae | Nitrosopumilus | unclassified |
| Otu015681 | 0.855 | 1 | 0.925 | 0.001 | Archaea | Crenarchaeota | Thaumarchaeota | Cenarchaeales | Cenarchaeaceae | Nitrosopumilus | unclassified |
| Otu015690 | 0.8038 | 0.8333 | 0.818 | 0.005 | Archaea | Crenarchaeota | Thaumarchaeota | Cenarchaeales | Cenarchaeaceae | Nitrosopumilus | unclassified |
| Otu015890 | 0.8029 | 0.8333 | 0.818 | 0.006 | Archaea | Crenarchaeota | Thaumarchaeota | Cenarchaeales | Cenarchaeaceae | Nitrosopumilus | unclassified |
| Otu016238 | 0.8777 | 1 | 0.937 | 0.001 | Archaea | Crenarchaeota | Thaumarchaeota | Cenarchaeales | Cenarchaeaceae | Nitrosopumilus | unclassified |
| Otu016249 | 0.8925 | 1 | 0.945 | 0.001 | Archaea | Crenarchaeota | Thaumarchaeota | Cenarchaeales | Cenarchaeaceae | Nitrosopumilus | unclassified |
| Otu017014 | 1 | 1 | 1 | 0.001 | Bacteria | Proteobacteria | Gammaproteobacteria | unclassified | unclassified | unclassified | unclassified |
| Otu017078 | 1 | 1 | 1 | 0.001 | Bacteria | Proteobacteria | Gammaproteobacteria | unclassified | unclassified | unclassified | unclassified |
| Otu017137 | 1 | 1 | 1 | 0.001 | Bacteria | Proteobacteria | Gammaproteobacteria | unclassified | unclassified | unclassified | unclassified |
| Otu017262 | 0.8711 | 0.8333 | 0.852 | 0.003 | Archaea | Crenarchaeota | Thaumarchaeota | Cenarchaeales | Cenarchaeaceae | Nitrosopumilus | unclassified |
| Otu017459 | 1 | 0.5 | 0.707 | 0.009 | Bacteria | Bacteroidetes | Flavobacteriia | Flavobacteriales | Flavobacteriaceae | unclassified | unclassified |
| Otu017749 | 0.8328 | 0.5 | 0.645 | 0.014 | Archaea | Crenarchaeota | Thaumarchaeota | Cenarchaeales | Cenarchaeaceae | Nitrosopumilus | unclassified |
| Otu017786 | 0.8773 | 0.6667 | 0.765 | 0.009 | Archaea | Crenarchaeota | Thaumarchaeota | Cenarchaeales | Cenarchaeaceae | Nitrosopumilus | unclassified |
| Otu017835 | 1 | 0.3333 | 0.577 | 0.036 | Bacteria | Proteobacteria | Gammaproteobacteria | unclassified | unclassified | unclassified | unclassified |
| Otu018087 | 1 | 0.8333 | 0.913 | 0.001 | Bacteria | Proteobacteria | Gammaproteobacteria | unclassified | unclassified | unclassified | unclassified |
| Otu018090 | 0.9879 | 1 | 0.994 | 0.001 | Bacteria | Proteobacteria | Gammaproteobacteria | unclassified | unclassified | unclassified | unclassified |
| Otu018285 | 0.8486 | 0.6667 | 0.752 | 0.003 | Archaea | Crenarchaeota | Thaumarchaeota | Cenarchaeales | Cenarchaeaceae | Nitrosopumilus | unclassified |
| Otu018303 | 0.8406 | 0.3333 | 0.529 | 0.05 | Archaea | Crenarchaeota | Thaumarchaeota | Cenarchaeales | Cenarchaeaceae | Nitrosopumilus | unclassified |
| Otu018361 | 0.7275 | 0.6667 | 0.696 | 0.044 | Archaea | Crenarchaeota | Thaumarchaeota | Cenarchaeales | Cenarchaeaceae | Nitrosopumilus | unclassified |
| Otu018394 | 1 | 0.5 | 0.707 | 0.006 | Archaea | Crenarchaeota | Thaumarchaeota | Cenarchaeales | Cenarchaeaceae | Nitrosopumilus | unclassified |
| Otu018427 | 0.941 | 0.8333 | 0.886 | 0.001 | Archaea | Crenarchaeota | Thaumarchaeota | Cenarchaeales | Cenarchaeaceae | Nitrosopumilus | unclassified |
| Otu018469 | 0.7232 | 0.6667 | 0.694 | 0.044 | Bacteria | unclassified | unclassified | unclassified | unclassified | unclassified | unclassified |
| Otu018738 | 0.7861 | 0.5 | 0.627 | 0.045 | Archaea | Crenarchaeota | Thaumarchaeota | Cenarchaeales | Cenarchaeaceae | Nitrosopumilus | unclassified |
| Otu018973 | 0.9309 | 1 | 0.965 | 0.001 | Archaea | Crenarchaeota | Thaumarchaeota | Cenarchaeales | Cenarchaeaceae | Nitrosopumilus | unclassified |
| Otu019001 | 0.8587 | 1 | 0.927 | 0.001 | Archaea | Crenarchaeota | Thaumarchaeota | Cenarchaeales | Cenarchaeaceae | Nitrosopumilus | unclassified |
| Otu019011 | 0.8299 | 0.8333 | 0.832 | 0.005 | Archaea | Crenarchaeota | Thaumarchaeota | Cenarchaeales | Cenarchaeaceae | Nitrosopumilus | unclassified |
| Otu019019 | 0.8887 | 1 | 0.943 | 0.001 | Archaea | Crenarchaeota | Thaumarchaeota | Cenarchaeales | Cenarchaeaceae | Nitrosopumilus | unclassified |
| Otu019072 | 0.8684 | 0.8333 | 0.851 | 0.002 | Archaea | Crenarchaeota | Thaumarchaeota | Cenarchaeales | Cenarchaeaceae | Nitrosopumilus | unclassified |
| Otu019079 | 0.8241 | 0.8333 | 0.829 | 0.005 | Archaea | Crenarchaeota | Thaumarchaeota | Cenarchaeales | Cenarchaeaceae | Nitrosopumilus | unclassified |
| Otu019162 | 1 | 1 | 1 | 0.001 | Bacteria | Proteobacteria | Gammaproteobacteria | unclassified | unclassified | unclassified | unclassified |
| Otu019233 | 0.8678 | 0.8333 | 0.85 | 0.001 | Archaea | Crenarchaeota | Thaumarchaeota | Cenarchaeales | Cenarchaeaceae | Nitrosopumilus | unclassified |
| Otu019258 | 0.8723 | 1 | 0.934 | 0.001 | Archaea | Crenarchaeota | Thaumarchaeota | Cenarchaeales | Cenarchaeaceae | Nitrosopumilus | unclassified |
| Otu019361 | 0.7907 | 0.8333 | 0.812 | 0.004 | Archaea | Crenarchaeota | Thaumarchaeota | Cenarchaeales | Cenarchaeaceae | Nitrosopumilus | unclassified |
| Otu019405 | 0.8492 | 1 | 0.921 | 0.001 | Archaea | Crenarchaeota | Thaumarchaeota | Cenarchaeales | Cenarchaeaceae | Nitrosopumilus | unclassified |
| Otu019416 | 0.8046 | 0.5 | 0.634 | 0.026 | Bacteria | Proteobacteria | Gammaproteobacteria | Vibrionales | Vibrionaceae | Photobacterium | angustum |
| Otu019487 | 0.8356 | 1 | 0.914 | 0.001 | Archaea | Crenarchaeota | Thaumarchaeota | Cenarchaeales | Cenarchaeaceae | Nitrosopumilus | unclassified |
| Otu020585 | 1 | 0.6667 | 0.816 | 0.001 | Bacteria | Proteobacteria | unclassified | unclassified | unclassified | unclassified | unclassified |
| Otu020665 | 1 | 0.3333 | 0.577 | 0.042 | Bacteria | Proteobacteria | Deltaproteobacteria | Myxococcales | unclassified | unclassified | unclassified |
| Otu020934 | 1 | 1 | 1 | 0.001 | Bacteria | Proteobacteria | Gammaproteobacteria | unclassified | unclassified | unclassified | unclassified |
| Otu021176 | 0.9349 | 0.6667 | 0.789 | 0.003 | Archaea | Crenarchaeota | Thaumarchaeota | Cenarchaeales | Cenarchaeaceae | Nitrosopumilus | unclassified |
| Otu021237 | 0.8595 | 0.8333 | 0.846 | 0.004 | Archaea | Crenarchaeota | Thaumarchaeota | Cenarchaeales | Cenarchaeaceae | Nitrosopumilus | unclassified |
| Otu021287 | 0.8103 | 1 | 0.9 | 0.001 | Archaea | Crenarchaeota | Thaumarchaeota | Cenarchaeales | Cenarchaeaceae | Nitrosopumilus | unclassified |
| Otu021359 | 0.8567 | 0.8333 | 0.845 | 0.003 | Archaea | Crenarchaeota | Thaumarchaeota | Cenarchaeales | Cenarchaeaceae | Nitrosopumilus | unclassified |
| Otu021402 | 1 | 0.5 | 0.707 | 0.009 | Bacteria | Proteobacteria | Gammaproteobacteria | unclassified | unclassified | unclassified | unclassified |
| Otu021538 | 1 | 0.3333 | 0.577 | 0.043 | Archaea | Crenarchaeota | Thaumarchaeota | Cenarchaeales | Cenarchaeaceae | Nitrosopumilus | unclassified |
| Otu021560 | 0.7578 | 0.8333 | 0.795 | 0.027 | Bacteria | Proteobacteria | Alphaproteobacteria | Rhodospirillales | Rhodospirillaceae | unclassified | unclassified |
| Otu021656 | 0.9712 | 0.5 | 0.697 | 0.009 | Bacteria | Bacteroidetes | Saprospirae | Saprospirales | Saprospiraceae | Saprospira | unclassified |
| Otu021772 | 0.7834 | 0.6667 | 0.723 | 0.014 | Archaea | Crenarchaeota | Thaumarchaeota | Cenarchaeales | Cenarchaeaceae | Nitrosopumilus | unclassified |
| Otu021858 | 0.8412 | 0.8333 | 0.837 | 0.004 | Archaea | Crenarchaeota | Thaumarchaeota | Cenarchaeales | Cenarchaeaceae | Nitrosopumilus | unclassified |
| Otu021876 | 0.9327 | 0.8333 | 0.882 | 0.001 | Archaea | Crenarchaeota | Thaumarchaeota | Cenarchaeales | Cenarchaeaceae | Nitrosopumilus | unclassified |
| Otu022881 | 0.8494 | 1 | 0.922 | 0.001 | Archaea | Crenarchaeota | Thaumarchaeota | Cenarchaeales | Cenarchaeaceae | Nitrosopumilus | unclassified |
| Otu022894 | 0.8625 | 1 | 0.929 | 0.002 | Archaea | Crenarchaeota | Thaumarchaeota | Cenarchaeales | Cenarchaeaceae | Nitrosopumilus | unclassified |
| Otu022926 | 1 | 1 | 1 | 0.001 | Bacteria | Proteobacteria | Gammaproteobacteria | unclassified | unclassified | unclassified | unclassified |
| Otu023079 | 1 | 0.3333 | 0.577 | 0.049 | Bacteria | Proteobacteria | Gammaproteobacteria | unclassified | unclassified | unclassified | unclassified |
| Otu023113 | 1 | 1 | 1 | 0.001 | Bacteria | Proteobacteria | Gammaproteobacteria | unclassified | unclassified | unclassified | unclassified |
| Otu023541 | 0.8107 | 0.8333 | 0.822 | 0.003 | Archaea | Crenarchaeota | Thaumarchaeota | Cenarchaeales | Cenarchaeaceae | Nitrosopumilus | unclassified |
| Otu023649 | 0.8593 | 0.8333 | 0.846 | 0.004 | Archaea | Crenarchaeota | Thaumarchaeota | Cenarchaeales | Cenarchaeaceae | Nitrosopumilus | unclassified |
| Otu023864 | 1 | 1 | 1 | 0.001 | Bacteria | Proteobacteria | Gammaproteobacteria | unclassified | unclassified | unclassified | unclassified |
| Otu024313 | 1 | 0.8333 | 0.913 | 0.001 | Bacteria | Proteobacteria | Gammaproteobacteria | unclassified | unclassified | unclassified | unclassified |
| Otu024324 | 1 | 1 | 1 | 0.001 | Bacteria | Proteobacteria | Gammaproteobacteria | unclassified | unclassified | unclassified | unclassified |
| Otu024376 | 1 | 0.8333 | 0.913 | 0.001 | Bacteria | Proteobacteria | Gammaproteobacteria | unclassified | unclassified | unclassified | unclassified |
| Otu024580 | 0.9221 | 0.3333 | 0.554 | 0.049 | Bacteria | Proteobacteria | Gammaproteobacteria | unclassified | unclassified | unclassified | unclassified |
| Otu024771 | 0.8695 | 1 | 0.932 | 0.001 | Archaea | Crenarchaeota | Thaumarchaeota | Cenarchaeales | Cenarchaeaceae | Nitrosopumilus | unclassified |
| Otu024888 | 0.8756 | 0.6667 | 0.764 | 0.009 | Archaea | Crenarchaeota | Thaumarchaeota | Cenarchaeales | Cenarchaeaceae | Nitrosopumilus | unclassified |
| Otu024937 | 0.8594 | 1 | 0.927 | 0.001 | Archaea | Crenarchaeota | Thaumarchaeota | Cenarchaeales | Cenarchaeaceae | Nitrosopumilus | unclassified |
| Otu025046 | 0.9391 | 0.6667 | 0.791 | 0.001 | Archaea | Crenarchaeota | Thaumarchaeota | Cenarchaeales | Cenarchaeaceae | Nitrosopumilus | unclassified |
| Otu025067 | 0.8739 | 1 | 0.935 | 0.001 | Archaea | Crenarchaeota | Thaumarchaeota | Cenarchaeales | Cenarchaeaceae | Nitrosopumilus | unclassified |
| Otu025148 | 0.8547 | 0.6667 | 0.755 | 0.01 | Archaea | Crenarchaeota | Thaumarchaeota | Cenarchaeales | Cenarchaeaceae | Nitrosopumilus | unclassified |
| Otu025204 | 0.8748 | 0.6667 | 0.764 | 0.012 | Archaea | Crenarchaeota | Thaumarchaeota | Cenarchaeales | Cenarchaeaceae | Nitrosopumilus | unclassified |
| Otu025402 | 0.8452 | 0.8333 | 0.839 | 0.003 | Archaea | Crenarchaeota | Thaumarchaeota | Cenarchaeales | Cenarchaeaceae | Nitrosopumilus | unclassified |
| Otu025428 | 1 | 0.8333 | 0.913 | 0.001 | Bacteria | Proteobacteria | Gammaproteobacteria | unclassified | unclassified | unclassified | unclassified |
| Otu025480 | 1 | 0.6667 | 0.816 | 0.002 | Bacteria | Proteobacteria | Gammaproteobacteria | unclassified | unclassified | unclassified | unclassified |
| Otu025926 | 0.8586 | 0.8333 | 0.846 | 0.002 | Archaea | Crenarchaeota | Thaumarchaeota | Cenarchaeales | Cenarchaeaceae | Nitrosopumilus | unclassified |
| Otu025945 | 0.9011 | 0.6667 | 0.775 | 0.008 | Archaea | Crenarchaeota | Thaumarchaeota | Cenarchaeales | Cenarchaeaceae | Nitrosopumilus | unclassified |
| Otu026415 | 0.873 | 0.8333 | 0.853 | 0.001 | Archaea | Crenarchaeota | Thaumarchaeota | Cenarchaeales | Cenarchaeaceae | Nitrosopumilus | unclassified |
| Otu026519 | 0.8664 | 0.8333 | 0.85 | 0.002 | Archaea | Crenarchaeota | Thaumarchaeota | Cenarchaeales | Cenarchaeaceae | Nitrosopumilus | unclassified |
| Otu026571 | 1 | 0.5 | 0.707 | 0.009 | Archaea | Crenarchaeota | Thaumarchaeota | Cenarchaeales | Cenarchaeaceae | Nitrosopumilus | unclassified |
| Otu026968 | 0.9478 | 0.6667 | 0.795 | 0.004 | Bacteria | Proteobacteria | Deltaproteobacteria | Myxococcales | OM27 | unclassified | unclassified |
| Otu026987 | 1 | 1 | 1 | 0.001 | Bacteria | Proteobacteria | unclassified | unclassified | unclassified | unclassified | unclassified |
| Otu027696 | 1 | 1 | 1 | 0.001 | Bacteria | Proteobacteria | unclassified | unclassified | unclassified | unclassified | unclassified |
| Otu027885 | 0.9016 | 0.6667 | 0.775 | 0.004 | Archaea | Crenarchaeota | Thaumarchaeota | Cenarchaeales | Cenarchaeaceae | Nitrosopumilus | unclassified |
| Otu027941 | 0.8452 | 0.6667 | 0.751 | 0.006 | Archaea | Crenarchaeota | Thaumarchaeota | Cenarchaeales | Cenarchaeaceae | Nitrosopumilus | unclassified |
| Otu028358 | 0.8787 | 0.8333 | 0.856 | 0.002 | Archaea | Crenarchaeota | Thaumarchaeota | Cenarchaeales | Cenarchaeaceae | Nitrosopumilus | unclassified |
| Otu028476 | 0.9838 | 0.8333 | 0.905 | 0.001 | Bacteria | unclassified | unclassified | unclassified | unclassified | unclassified | unclassified |
| Otu028611 | 0.9129 | 0.5 | 0.676 | 0.014 | Bacteria | Proteobacteria | Gammaproteobacteria | Alteromonadales | Alteromonadaceae | Microbulbifer | unclassified |
| Otu028848 | 0.872 | 0.8333 | 0.852 | 0.001 | Archaea | Crenarchaeota | Thaumarchaeota | Cenarchaeales | Cenarchaeaceae | Nitrosopumilus | unclassified |
| Otu030283 | 1 | 0.3333 | 0.577 | 0.049 | Bacteria | Proteobacteria | Gammaproteobacteria | unclassified | unclassified | unclassified | unclassified |
| Otu030656 | 1 | 0.5 | 0.707 | 0.009 | Bacteria | Proteobacteria | Gammaproteobacteria | unclassified | unclassified | unclassified | unclassified |
| Otu030672 | 1 | 0.8333 | 0.913 | 0.001 | Bacteria | Proteobacteria | Gammaproteobacteria | unclassified | unclassified | unclassified | unclassified |
| Otu030803 | 1 | 0.3333 | 0.577 | 0.041 | Bacteria | Proteobacteria | Gammaproteobacteria | Alteromonadales | unclassified | unclassified | unclassified |

| | | | | | | | | | | | |
|---|---|---|---|---|---|---|---|---|---|---|---|
| Otu030879 | 1 | 0.8333 | 0.913 | 0.001 | Bacteria | Proteobacteria | Gammaproteobacteria | unclassified | unclassified | unclassified | unclassified |
| Otu030901 | 0.8127 | 0.8333 | 0.823 | 0.004 | Archaea | Crenarchaeota | Thaumarchaeota | Cenarchaeales | Cenarchaeaceae | Nitrosopumilus | unclassified |
| Otu031597 | 0.8741 | 1 | 0.935 | 0.001 | Archaea | Crenarchaeota | Thaumarchaeota | Cenarchaeales | Cenarchaeaceae | Nitrosopumilus | unclassified |
| Otu031704 | 1 | 0.8333 | 0.913 | 0.001 | Bacteria | Proteobacteria | Gammaproteobacteria | Enterobacteriales | Enterobacteriaceae | unclassified | unclassified |
| Otu031976 | 1 | 0.8333 | 0.913 | 0.001 | Bacteria | Proteobacteria | Gammaproteobacteria | unclassified | unclassified | unclassified | unclassified |
| Otu032365 | 0.8766 | 0.5 | 0.662 | 0.03 | Bacteria | Proteobacteria | Gammaproteobacteria | unclassified | unclassified | unclassified | unclassified |
| Otu032375 | 1 | 0.8333 | 0.913 | 0.001 | Bacteria | Proteobacteria | Gammaproteobacteria | unclassified | unclassified | unclassified | unclassified |
| Otu032393 | 1 | 0.5 | 0.707 | 0.004 | Bacteria | Proteobacteria | Gammaproteobacteria | unclassified | unclassified | unclassified | unclassified |
| Otu035127 | 0.8253 | 0.5 | 0.642 | 0.031 | Bacteria | Verrucomicrobia | Pedosphaerae | Pedosphaerales | unclassified | unclassified | unclassified |
| Otu035248 | 0.8601 | 0.6667 | 0.757 | 0.005 | Archaea | Crenarchaeota | Thaumarchaeota | Cenarchaeales | Cenarchaeaceae | Nitrosopumilus | unclassified |
| Otu035274 | 0.8806 | 0.8333 | 0.857 | 0.002 | Archaea | Crenarchaeota | Thaumarchaeota | Cenarchaeales | Cenarchaeaceae | Nitrosopumilus | unclassified |
| Otu035923 | 0.9046 | 0.5 | 0.673 | 0.011 | Bacteria | Proteobacteria | Deltaproteobacteria | Myxococcales | Cystobacterineae | unclassified | unclassified |
| Otu035981 | 0.902 | 0.6667 | 0.775 | 0.004 | Archaea | Crenarchaeota | Thaumarchaeota | Cenarchaeales | Cenarchaeaceae | Nitrosopumilus | unclassified |
| Otu036684 | 0.9466 | 0.6667 | 0.794 | 0.004 | Archaea | Crenarchaeota | Thaumarchaeota | Cenarchaeales | Cenarchaeaceae | Nitrosopumilus | unclassified |
| Otu037189 | 0.8566 | 0.6667 | 0.756 | 0.005 | Archaea | Crenarchaeota | Thaumarchaeota | Cenarchaeales | Cenarchaeaceae | Nitrosopumilus | unclassified |
| Otu037233 | 0.7826 | 0.5 | 0.626 | 0.031 | Archaea | Crenarchaeota | Thaumarchaeota | Cenarchaeales | Cenarchaeaceae | Nitrosopumilus | unclassified |
| Otu037263 | 0.908 | 0.8333 | 0.87 | 0.001 | Archaea | Crenarchaeota | Thaumarchaeota | Cenarchaeales | Cenarchaeaceae | Nitrosopumilus | unclassified |
| Otu037287 | 0.8437 | 0.8333 | 0.838 | 0.002 | Archaea | Crenarchaeota | Thaumarchaeota | Cenarchaeales | Cenarchaeaceae | Nitrosopumilus | unclassified |
| Otu038151 | 0.8155 | 0.6667 | 0.737 | 0.015 | Archaea | Crenarchaeota | Thaumarchaeota | Cenarchaeales | Cenarchaeaceae | Nitrosopumilus | unclassified |
| Otu039128 | 0.9596 | 0.8333 | 0.894 | 0.001 | Archaea | Crenarchaeota | Thaumarchaeota | Cenarchaeales | Cenarchaeaceae | Nitrosopumilus | unclassified |
| Otu039544 | 0.933 | 0.6667 | 0.789 | 0.001 | Archaea | Crenarchaeota | Thaumarchaeota | Cenarchaeales | Cenarchaeaceae | Nitrosopumilus | unclassified |
| Otu040165 | 0.9657 | 0.6667 | 0.802 | 0.001 | Archaea | Crenarchaeota | Thaumarchaeota | Cenarchaeales | Cenarchaeaceae | Nitrosopumilus | unclassified |
| Otu040608 | 0.8361 | 0.6667 | 0.747 | 0.015 | Archaea | Crenarchaeota | Thaumarchaeota | Cenarchaeales | Cenarchaeaceae | Nitrosopumilus | unclassified |
| Otu040719 | 0.9265 | 0.8333 | 0.879 | 0.002 | Archaea | Crenarchaeota | Thaumarchaeota | Cenarchaeales | Cenarchaeaceae | Nitrosopumilus | unclassified |
| Otu041365 | 1 | 0.3333 | 0.577 | 0.036 | Bacteria | Proteobacteria | Gammaproteobacteria | Alteromonadales | unclassified | unclassified | unclassified |
| Otu042095 | 1 | 0.5 | 0.707 | 0.009 | Bacteria | Proteobacteria | Gammaproteobacteria | unclassified | unclassified | unclassified | unclassified |
| Otu043335 | 0.9882 | 1 | 0.994 | 0.001 | Bacteria | Nitrospirae | Nitrospira | Nitrospirales | Nitrospiraceae | unclassified | unclassified |
| Otu043432 | 0.8657 | 0.8333 | 0.849 | 0.003 | Archaea | Crenarchaeota | Thaumarchaeota | Cenarchaeales | Cenarchaeaceae | Nitrosopumilus | unclassified |
| Otu043669 | 0.8613 | 0.5 | 0.656 | 0.017 | Archaea | Crenarchaeota | Thaumarchaeota | Cenarchaeales | Cenarchaeaceae | Nitrosopumilus | unclassified |
| Otu044173 | 0.9584 | 0.5 | 0.692 | 0.007 | Bacteria | Proteobacteria | Gammaproteobacteria | unclassified | unclassified | unclassified | unclassified |
| Otu046395 | 0.8938 | 0.8333 | 0.863 | 0.004 | Archaea | Crenarchaeota | Thaumarchaeota | Cenarchaeales | Cenarchaeaceae | Nitrosopumilus | unclassified |
| Otu047258 | 1 | 0.5 | 0.707 | 0.006 | Archaea | Crenarchaeota | Thaumarchaeota | Cenarchaeales | Cenarchaeaceae | Nitrosopumilus | unclassified |
| Otu047308 | 0.7829 | 0.5 | 0.626 | 0.038 | Archaea | Crenarchaeota | Thaumarchaeota | Cenarchaeales | Cenarchaeaceae | Nitrosopumilus | unclassified |
| Otu047334 | 0.8507 | 0.8333 | 0.842 | 0.004 | Archaea | Crenarchaeota | Thaumarchaeota | Cenarchaeales | Cenarchaeaceae | Nitrosopumilus | unclassified |
| Otu047355 | 0.8756 | 1 | 0.936 | 0.001 | Archaea | Crenarchaeota | Thaumarchaeota | Cenarchaeales | Cenarchaeaceae | Nitrosopumilus | unclassified |
| Otu047916 | 0.9849 | 0.6667 | 0.81 | 0.003 | Bacteria | Proteobacteria | Gammaproteobacteria | unclassified | unclassified | unclassified | unclassified |
| Otu048942 | 0.885 | 0.8333 | 0.859 | 0.002 | Archaea | Crenarchaeota | Thaumarchaeota | Cenarchaeales | Cenarchaeaceae | Nitrosopumilus | unclassified |
| Otu049309 | 0.8562 | 0.8333 | 0.845 | 0.002 | Archaea | Crenarchaeota | Thaumarchaeota | Cenarchaeales | Cenarchaeaceae | Nitrosopumilus | unclassified |
| Otu050227 | 1 | 0.5 | 0.707 | 0.009 | Bacteria | Proteobacteria | Gammaproteobacteria | unclassified | unclassified | unclassified | unclassified |
| Otu050514 | 1 | 0.5 | 0.707 | 0.012 | Bacteria | Proteobacteria | Gammaproteobacteria | unclassified | unclassified | unclassified | unclassified |
| Otu051199 | 1 | 0.5 | 0.707 | 0.008 | Bacteria | Proteobacteria | Gammaproteobacteria | unclassified | unclassified | unclassified | unclassified |
| Otu051521 | 1 | 0.3333 | 0.577 | 0.044 | Bacteria | Proteobacteria | Gammaproteobacteria | unclassified | unclassified | unclassified | unclassified |
| Otu051569 | 1 | 0.6667 | 0.816 | 0.002 | Bacteria | Proteobacteria | Gammaproteobacteria | unclassified | unclassified | unclassified | unclassified |
| Otu052808 | 1 | 0.8333 | 0.913 | 0.001 | Bacteria | Proteobacteria | Gammaproteobacteria | unclassified | unclassified | unclassified | unclassified |
| Otu053648 | 1 | 0.8333 | 0.913 | 0.001 | Bacteria | Proteobacteria | Gammaproteobacteria | unclassified | unclassified | unclassified | unclassified |
| Otu053691 | 1 | 0.6667 | 0.816 | 0.002 | Bacteria | Proteobacteria | Gammaproteobacteria | unclassified | unclassified | unclassified | unclassified |
| Otu054125 | 1 | 0.8333 | 0.913 | 0.001 | Bacteria | Proteobacteria | Gammaproteobacteria | unclassified | unclassified | unclassified | unclassified |
| Otu055256 | 0.8943 | 0.5 | 0.669 | 0.019 | Bacteria | Proteobacteria | unclassified | unclassified | unclassified | unclassified | unclassified |
| Otu056672 | 0.905 | 0.3333 | 0.549 | 0.043 | Bacteria | Proteobacteria | Alphaproteobacteria | unclassified | unclassified | unclassified | unclassified |
| Otu057077 | 1 | 1 | 1 | 0.001 | Bacteria | Proteobacteria | Gammaproteobacteria | unclassified | unclassified | unclassified | unclassified |
| Otu057172 | 1 | 0.8333 | 0.913 | 0.001 | Bacteria | Proteobacteria | Gammaproteobacteria | unclassified | unclassified | unclassified | unclassified |
| Otu057191 | 1 | 0.8333 | 0.913 | 0.001 | Bacteria | Proteobacteria | Gammaproteobacteria | unclassified | unclassified | unclassified | unclassified |
| Otu057419 | 1 | 0.6667 | 0.816 | 0.002 | Bacteria | Proteobacteria | Gammaproteobacteria | unclassified | unclassified | unclassified | unclassified |
| Otu057808 | 1 | 1 | 1 | 0.001 | Bacteria | Proteobacteria | Gammaproteobacteria | unclassified | unclassified | unclassified | unclassified |
| Otu058096 | 1 | 0.3333 | 0.577 | 0.045 | Bacteria | Proteobacteria | Gammaproteobacteria | Enterobacteriales | Enterobacteriaceae | Serratia | marcescens |
| Otu060131 | 0.9776 | 1 | 0.989 | 0.001 | Bacteria | Nitrospirae | Nitrospira | Nitrospirales | Nitrospiraceae | unclassified | unclassified |
| Otu060145 | 0.9252 | 0.6667 | 0.785 | 0.004 | Archaea | Crenarchaeota | Thaumarchaeota | Cenarchaeales | Cenarchaeaceae | Nitrosopumilus | unclassified |
| Otu060538 | 1 | 0.6667 | 0.816 | 0.001 | Bacteria | Proteobacteria | Gammaproteobacteria | unclassified | unclassified | unclassified | unclassified |
| Otu060722 | 1 | 0.8333 | 0.913 | 0.001 | Bacteria | Proteobacteria | Gammaproteobacteria | unclassified | unclassified | unclassified | unclassified |
| Otu061365 | 1 | 0.8333 | 0.913 | 0.001 | Bacteria | unclassified | unclassified | unclassified | unclassified | unclassified | unclassified |
| Otu061479 | 0.7809 | 0.6667 | 0.722 | 0.012 | Archaea | Crenarchaeota | Thaumarchaeota | Cenarchaeales | Cenarchaeaceae | Nitrosopumilus | unclassified |
| Otu061680 | 1 | 0.3333 | 0.577 | 0.049 | Bacteria | Proteobacteria | Alphaproteobacteria | Rhodobacterales | Rhodobacteraceae | Phaeobacter | unclassified |
| Otu062087 | 0.9244 | 0.6667 | 0.785 | 0.003 | Bacteria | Proteobacteria | Alphaproteobacteria | Rhodobacterales | Rhodobacteraceae | Loktanella | salsilacus |
| Otu062765 | 1 | 0.5 | 0.707 | 0.005 | Bacteria | Proteobacteria | Gammaproteobacteria | unclassified | unclassified | unclassified | unclassified |
| Otu063030 | 1 | 1 | 1 | 0.001 | Bacteria | Proteobacteria | Gammaproteobacteria | unclassified | unclassified | unclassified | unclassified |
| Otu063109 | 1 | 0.5 | 0.707 | 0.006 | Bacteria | Proteobacteria | unclassified | unclassified | unclassified | unclassified | unclassified |
| Otu063692 | 1 | 1 | 1 | 0.001 | Bacteria | Proteobacteria | Gammaproteobacteria | unclassified | unclassified | unclassified | unclassified |
| Otu063716 | 1 | 0.8333 | 0.913 | 0.001 | Bacteria | Proteobacteria | Gammaproteobacteria | unclassified | unclassified | unclassified | unclassified |
| Otu064765 | 1 | 0.6667 | 0.816 | 0.002 | Bacteria | Proteobacteria | Gammaproteobacteria | unclassified | unclassified | unclassified | unclassified |
| Otu065295 | 0.8227 | 0.6667 | 0.741 | 0.007 | Bacteria | Cyanobacteria | Synechococcophycideae | Synechococcales | Synechococcaceae | unclassified | unclassified |
| Otu065662 | 1 | 1 | 1 | 0.001 | Bacteria | Proteobacteria | Gammaproteobacteria | unclassified | unclassified | unclassified | unclassified |
| Otu066273 | 1 | 1 | 1 | 0.001 | Bacteria | Proteobacteria | Gammaproteobacteria | unclassified | unclassified | unclassified | unclassified |
| Otu066936 | 1 | 0.3333 | 0.577 | 0.044 | Bacteria | Proteobacteria | Gammaproteobacteria | unclassified | unclassified | unclassified | unclassified |
| Otu067769 | 0.9603 | 0.8333 | 0.895 | 0.001 | Archaea | Crenarchaeota | Thaumarchaeota | Cenarchaeales | Cenarchaeaceae | Nitrosopumilus | unclassified |
| Otu068072 | 0.782 | 1 | 0.884 | 0.002 | Archaea | Crenarchaeota | Thaumarchaeota | Cenarchaeales | Cenarchaeaceae | Nitrosopumilus | unclassified |
| Otu069568 | 1 | 0.3333 | 0.577 | 0.049 | Archaea | Crenarchaeota | Thaumarchaeota | Cenarchaeales | Cenarchaeaceae | Nitrosopumilus | unclassified |
| Otu077130 | 0.9186 | 0.5 | 0.678 | 0.01 | Bacteria | Proteobacteria | Alphaproteobacteria | Rhodobacterales | Rhodobacteraceae | unclassified | unclassified |
| Otu077267 | 0.7406 | 1 | 0.861 | 0.002 | Bacteria | Proteobacteria | Alphaproteobacteria | Rhodobacterales | Rhodobacteraceae | Octadecabacter | unclassified |
| Otu084293 | 1 | 0.3333 | 0.577 | 0.043 | Bacteria | Proteobacteria | Gammaproteobacteria | Vibrionales | Vibrionaceae | Vibrio | unclassified |
| Otu093768 | 0.8144 | 0.5 | 0.638 | 0.014 | Archaea | Crenarchaeota | Thaumarchaeota | Cenarchaeales | Cenarchaeaceae | Nitrosopumilus | unclassified |
| Otu094642 | 0.8538 | 0.6667 | 0.754 | 0.008 | Archaea | Crenarchaeota | Thaumarchaeota | Cenarchaeales | Cenarchaeaceae | Nitrosopumilus | unclassified |
| Otu094777 | 0.857 | 1 | 0.926 | 0.001 | Archaea | Crenarchaeota | Thaumarchaeota | Cenarchaeales | Cenarchaeaceae | Nitrosopumilus | unclassified |
| Otu097953 | 0.864 | 0.6667 | 0.759 | 0.007 | Archaea | Crenarchaeota | Thaumarchaeota | Cenarchaeales | Cenarchaeaceae | Nitrosopumilus | unclassified |
| Otu108091 | 1 | 0.6667 | 0.816 | 0.002 | Bacteria | Proteobacteria | Gammaproteobacteria | unclassified | unclassified | unclassified | unclassified |
| Otu109528 | 1 | 1 | 1 | 0.001 | Bacteria | Proteobacteria | Gammaproteobacteria | unclassified | unclassified | unclassified | unclassified |
| Otu109701 | 1 | 0.5 | 0.707 | 0.004 | Bacteria | Proteobacteria | Gammaproteobacteria | unclassified | unclassified | unclassified | unclassified |
| Otu109884 | 1 | 0.8333 | 0.913 | 0.001 | Bacteria | Proteobacteria | Gammaproteobacteria | unclassified | unclassified | unclassified | unclassified |
| Otu110247 | 1 | 0.3333 | 0.577 | 0.049 | Bacteria | Proteobacteria | Gammaproteobacteria | unclassified | unclassified | unclassified | unclassified |
| Otu110251 | 1 | 0.8333 | 0.913 | 0.001 | Bacteria | Proteobacteria | Gammaproteobacteria | unclassified | unclassified | unclassified | unclassified |
| Otu110262 | 1 | 0.3333 | 0.577 | 0.041 | Bacteria | Proteobacteria | Gammaproteobacteria | unclassified | unclassified | unclassified | unclassified |
| Otu110271 | 0.8981 | 0.6667 | 0.774 | 0.001 | Bacteria | Proteobacteria | Gammaproteobacteria | Alteromonadales | unclassified | unclassified | unclassified |
| Otu110337 | 1 | 0.6667 | 0.816 | 0.002 | Bacteria | Proteobacteria | Gammaproteobacteria | unclassified | unclassified | unclassified | unclassified |
| Otu110482 | 1 | 0.6667 | 0.816 | 0.001 | Bacteria | Proteobacteria | Gammaproteobacteria | unclassified | unclassified | unclassified | unclassified |
| Otu111028 | 1 | 0.8333 | 0.913 | 0.001 | Bacteria | Proteobacteria | Gammaproteobacteria | unclassified | unclassified | unclassified | unclassified |
| Otu113695 | 1 | 0.3333 | 0.577 | 0.043 | Bacteria | Proteobacteria | Gammaproteobacteria | unclassified | unclassified | unclassified | unclassified |
| Otu117884 | 1 | 0.8333 | 0.913 | 0.001 | Bacteria | Proteobacteria | Gammaproteobacteria | unclassified | unclassified | unclassified | unclassified |
| Otu117940 | 1 | 0.6667 | 0.816 | 0.002 | Bacteria | Proteobacteria | Gammaproteobacteria | unclassified | unclassified | unclassified | unclassified |
| Otu118879 | 0.8305 | 0.5 | 0.644 | 0.031 | Bacteria | Cyanobacteria | Synechococcophycideae | Synechococcales | Synechococcaceae | Synechococcus | unclassified |
| Otu118950 | 1 | 0.3333 | 0.577 | 0.045 | Bacteria | Cyanobacteria | Synechococcophycideae | Synechococcales | Synechococcaceae | unclassified | unclassified |
| Otu120711 | 1 | 0.6667 | 0.816 | 0.002 | Bacteria | Proteobacteria | Gammaproteobacteria | unclassified | unclassified | unclassified | unclassified |

| | | | | | | | | | | | |
|---|---|---|---|---|---|---|---|---|---|---|---|
| Otu120747 | 1 | 1 | 1 | 0.001 | Bacteria | Proteobacteria | Gammaproteobacteria | unclassified | unclassified | unclassified | unclassified |
| Otu121434 | 1 | 0.6667 | 0.816 | 0.002 | Bacteria | Proteobacteria | Gammaproteobacteria | unclassified | unclassified | unclassified | unclassified |
| Otu121450 | 0.9561 | 0.3333 | 0.565 | 0.049 | Bacteria | Proteobacteria | Gammaproteobacteria | Alteromonadales | unclassified | unclassified | unclassified |
| Otu121788 | 1 | 0.8333 | 0.913 | 0.001 | Bacteria | Proteobacteria | Gammaproteobacteria | unclassified | unclassified | unclassified | unclassified |
| Otu121795 | 1 | 0.3333 | 0.577 | 0.049 | Bacteria | Proteobacteria | Gammaproteobacteria | unclassified | unclassified | unclassified | unclassified |
| Otu121803 | 1 | 0.8333 | 0.913 | 0.001 | Bacteria | Proteobacteria | Gammaproteobacteria | unclassified | unclassified | unclassified | unclassified |
| Otu121900 | 1 | 1 | 1 | 0.001 | Bacteria | Proteobacteria | Gammaproteobacteria | unclassified | unclassified | unclassified | unclassified |
| Otu122390 | 1 | 0.3333 | 0.577 | 0.043 | Bacteria | Proteobacteria | Gammaproteobacteria | Enterobacteriales | Enterobacteriaceae | unclassified | unclassified |
| Otu122464 | 1 | 0.5 | 0.707 | 0.01 | Bacteria | Proteobacteria | Gammaproteobacteria | unclassified | unclassified | unclassified | unclassified |
| Otu122557 | 1 | 0.3333 | 0.577 | 0.041 | Bacteria | Proteobacteria | Gammaproteobacteria | unclassified | unclassified | unclassified | unclassified |
| Otu123201 | 1 | 1 | 1 | 0.001 | Bacteria | Proteobacteria | Gammaproteobacteria | unclassified | unclassified | unclassified | unclassified |
| Otu123893 | 1 | 0.6667 | 0.816 | 0.001 | Bacteria | Proteobacteria | Gammaproteobacteria | unclassified | unclassified | unclassified | unclassified |
| Otu124295 | 1 | 0.8333 | 0.913 | 0.001 | Bacteria | Proteobacteria | Gammaproteobacteria | unclassified | unclassified | unclassified | unclassified |
| Otu124518 | 1 | 0.8333 | 0.913 | 0.001 | Bacteria | Proteobacteria | Gammaproteobacteria | unclassified | unclassified | unclassified | unclassified |
| Otu124821 | 1 | 0.3333 | 0.577 | 0.049 | Bacteria | Proteobacteria | Gammaproteobacteria | unclassified | unclassified | unclassified | unclassified |
| Otu126595 | 1 | 1 | 1 | 0.001 | Bacteria | Proteobacteria | Gammaproteobacteria | unclassified | unclassified | unclassified | unclassified |
| Otu127072 | 1 | 1 | 1 | 0.001 | Bacteria | Proteobacteria | Gammaproteobacteria | unclassified | unclassified | unclassified | unclassified |
| Otu127473 | 1 | 0.3333 | 0.577 | 0.041 | Bacteria | Proteobacteria | Gammaproteobacteria | unclassified | unclassified | unclassified | unclassified |
| Otu127914 | 1 | 0.3333 | 0.577 | 0.044 | Bacteria | Proteobacteria | Gammaproteobacteria | unclassified | unclassified | unclassified | unclassified |
| Otu128129 | 1 | 1 | 1 | 0.001 | Bacteria | Proteobacteria | Gammaproteobacteria | unclassified | unclassified | unclassified | unclassified |
| Otu129207 | 1 | 0.6667 | 0.816 | 0.002 | Bacteria | Proteobacteria | Gammaproteobacteria | unclassified | unclassified | unclassified | unclassified |
| Otu136079 | 1 | 0.5 | 0.707 | 0.012 | Bacteria | Proteobacteria | unclassified | unclassified | unclassified | unclassified | unclassified |
| Otu137000 | 1 | 0.8333 | 0.913 | 0.001 | Bacteria | Proteobacteria | unclassified | unclassified | unclassified | unclassified | unclassified |
| Otu149554 | 0.9213 | 0.3333 | 0.554 | 0.049 | Bacteria | Proteobacteria | Alphaproteobacteria | Rhodobacterales | Rhodobacteraceae | Pseudoruegeria | unclassified |
| Otu152185 | 0.9173 | 0.5 | 0.677 | 0.012 | Bacteria | Proteobacteria | Gammaproteobacteria | Vibrionales | Vibrionaceae | Aliivibrio | fischeri |
| Otu158589 | 0.8668 | 0.6667 | 0.76 | 0.01 | Bacteria | Proteobacteria | Gammaproteobacteria | Alteromonadales | Alteromonadaceae | HTCC2207 | unclassified |
| Otu174598 | 0.9304 | 0.3333 | 0.557 | 0.049 | Bacteria | Proteobacteria | Gammaproteobacteria | unclassified | unclassified | unclassified | unclassified |
| Otu215775 | 1 | 0.8333 | 0.913 | 0.001 | Bacteria | Proteobacteria | Gammaproteobacteria | unclassified | unclassified | unclassified | unclassified |
| Otu217528 | 1 | 1 | 1 | 0.001 | Bacteria | Proteobacteria | Gammaproteobacteria | unclassified | unclassified | unclassified | unclassified |
| Otu217541 | 1 | 0.5 | 0.707 | 0.003 | Bacteria | Proteobacteria | Gammaproteobacteria | unclassified | unclassified | unclassified | unclassified |
| Otu220169 | 1 | 0.6667 | 0.816 | 0.001 | Bacteria | Proteobacteria | Gammaproteobacteria | unclassified | unclassified | unclassified | unclassified |
| Otu222567 | 0.9537 | 0.5 | 0.691 | 0.012 | Bacteria | Planctomycetes | Planctomycetia | Planctomycetales | Planctomycetaceae | Planctomyces | unclassified |
| Otu237000 | 1 | 0.5 | 0.707 | 0.013 | Bacteria | Proteobacteria | Gammaproteobacteria | unclassified | unclassified | unclassified | unclassified |
| Otu240018 | 1 | 0.6667 | 0.816 | 0.003 | Bacteria | Proteobacteria | Gammaproteobacteria | unclassified | unclassified | unclassified | unclassified |
| Otu240225 | 1 | 0.8333 | 0.913 | 0.001 | Bacteria | Proteobacteria | Gammaproteobacteria | unclassified | unclassified | unclassified | unclassified |
| Otu246892 | 1 | 0.5 | 0.707 | 0.009 | Bacteria | Proteobacteria | Gammaproteobacteria | unclassified | unclassified | unclassified | unclassified |
| Otu251196 | 1 | 0.5 | 0.707 | 0.009 | Bacteria | Proteobacteria | Gammaproteobacteria | unclassified | unclassified | unclassified | unclassified |
| Otu251484 | 1 | 0.8333 | 0.913 | 0.001 | Bacteria | Proteobacteria | Gammaproteobacteria | unclassified | unclassified | unclassified | unclassified |
| Otu252313 | 1 | 0.6667 | 0.816 | 0.002 | Bacteria | Proteobacteria | Gammaproteobacteria | unclassified | unclassified | unclassified | unclassified |
| Otu257013 | 1 | 0.8333 | 0.913 | 0.001 | Bacteria | Proteobacteria | Gammaproteobacteria | unclassified | unclassified | unclassified | unclassified |
| Otu259625 | 1 | 0.8333 | 0.913 | 0.001 | Bacteria | Proteobacteria | Gammaproteobacteria | unclassified | unclassified | unclassified | unclassified |
| Otu262863 | 1 | 0.3333 | 0.577 | 0.049 | Bacteria | Proteobacteria | Gammaproteobacteria | unclassified | unclassified | unclassified | unclassified |
| Otu272760 | 1 | 0.8333 | 0.913 | 0.001 | Bacteria | unclassified | unclassified | unclassified | unclassified | unclassified | unclassified |

| **0.03 Otus** | **marine metagenome** | **Rest** | **stat** | ***p value*** | **Domain** | **Phylum** | **Class** | **Order** | **Family** | **Genus** | **Species** |
|---|---|---|---|---|---|---|---|---|---|---|---|
| Otu000069 | 1 | 1 | 1 | 0.001 | Bacteria | Proteobacteria | Betaproteobacteria | unclassified | unclassified | unclassified | unclassified |
| Otu000075 | 1 | 1 | 1 | 0.001 | Bacteria | Bacteroidetes | Flavobacteriia | Flavobacteriales | Cryomorphaceae | unclassified | unclassified |
| Otu000085 | 1 | 1 | 1 | 0.001 | Bacteria | Actinobacteria | Acidimicrobiia | Acidimicrobiales | OCS155 | unclassified | unclassified |
| Otu000092 | 1 | 1 | 1 | 0.001 | Bacteria | Bacteroidetes | Flavobacteriia | Flavobacteriales | Flavobacteriaceae | unclassified | unclassified |
| Otu000095 | 1 | 1 | 1 | 0.001 | Bacteria | Proteobacteria | Gammaproteobacteria | Pseudomonadales | Moraxellaceae | Psychrobacter | pacificensis |
| Otu000106 | 1 | 1 | 1 | 0.001 | Bacteria | Proteobacteria | Gammaproteobacteria | Vibrionales | Vibrionaceae | Photobacterium | angustum |
| Otu000119 | 1 | 1 | 1 | 0.001 | Bacteria | Proteobacteria | Gammaproteobacteria | Oceanospirillales | Halomonadaceae | Candidatus_Portiera | unclassified |
| Otu000146 | 1 | 1 | 1 | 0.001 | Bacteria | Proteobacteria | Gammaproteobacteria | Alteromonadales | OM60 | unclassified | unclassified |
| Otu000148 | 1 | 1 | 1 | 0.001 | Bacteria | Proteobacteria | Gammaproteobacteria | Oceanospirillales | Halomonadaceae | Candidatus_Portiera | unclassified |
| Otu000154 | 1 | 0.6667 | 0.816 | 0.007 | Bacteria | Proteobacteria | Alphaproteobacteria | Rhodobacterales | Rhodobacteraceae | unclassified | unclassified |
| Otu000198 | 1 | 1 | 1 | 0.001 | Bacteria | Proteobacteria | Gammaproteobacteria | Oceanospirillales | Halomonadaceae | Candidatus_Portiera | unclassified |
| Otu000205 | 1 | 1 | 1 | 0.001 | Bacteria | Proteobacteria | Alphaproteobacteria | Rhodobacterales | Rhodobacteraceae | Octadecabacter | unclassified |
| Otu000217 | 1 | 1 | 1 | 0.001 | Bacteria | Bacteroidetes | Flavobacteriia | Flavobacteriales | Cryomorphaceae | unclassified | unclassified |
| Otu000258 | 1 | 1 | 1 | 0.001 | Bacteria | Bacteroidetes | Flavobacteriia | Flavobacteriales | unclassified | unclassified | unclassified |
| Otu000266 | 1 | 1 | 1 | 0.001 | Bacteria | Proteobacteria | Gammaproteobacteria | HTCC2188 | HTCC2089 | unclassified | unclassified |
| Otu000275 | 1 | 1 | 1 | 0.001 | Bacteria | Planctomycetes | Planctomycetia | Pirellulales | Pirellulaceae | unclassified | unclassified |
| Otu000288 | 1 | 0.6667 | 0.816 | 0.01 | Bacteria | Bacteroidetes | Flavobacteriia | Flavobacteriales | Flavobacteriaceae | unclassified | unclassified |
| Otu000295 | 1 | 1 | 1 | 0.001 | Bacteria | Bacteroidetes | Flavobacteriia | Flavobacteriales | Flavobacteriaceae | unclassified | unclassified |
| Otu000309 | 1 | 1 | 1 | 0.001 | Bacteria | Proteobacteria | Alphaproteobacteria | Rhodobacterales | Rhodobacteraceae | Octadecabacter | unclassified |
| Otu000321 | 0.7678 | 1 | 0.876 | 0.012 | Bacteria | Proteobacteria | Gammaproteobacteria | Pseudomonadales | Moraxellaceae | Acinetobacter | unclassified |
| Otu000323 | 1 | 1 | 1 | 0.001 | Bacteria | Proteobacteria | unclassified | unclassified | unclassified | unclassified | unclassified |
| Otu000325 | 1 | 1 | 1 | 0.001 | Bacteria | Proteobacteria | Betaproteobacteria | Methylophilales | Methylophilaceae | unclassified | unclassified |
| Otu000326 | 1 | 1 | 1 | 0.001 | Bacteria | Cyanobacteria | Synechococcophycideae | Synechococcales | Synechococcaceae | Synechococcus | unclassified |
| Otu000337 | 1 | 1 | 1 | 0.001 | Bacteria | Bacteroidetes | Flavobacteriia | Flavobacteriales | NS9 | unclassified | unclassified |
| Otu000358 | 1 | 1 | 1 | 0.001 | Archaea | Euryarchaeota | Thermoplasmata | E2 | Marine_group_II | unclassified | unclassified |
| Otu000374 | 1 | 1 | 1 | 0.001 | Bacteria | Proteobacteria | unclassified | unclassified | unclassified | unclassified | unclassified |
| Otu000380 | 1 | 1 | 1 | 0.001 | Bacteria | Proteobacteria | Alphaproteobacteria | Rickettsiales | Pelagibacteraceae | unclassified | unclassified |
| Otu000381 | 1 | 1 | 1 | 0.001 | Bacteria | Bacteroidetes | Flavobacteriia | Flavobacteriales | Flavobacteriaceae | unclassified | unclassified |
| Otu000389 | 1 | 1 | 1 | 0.001 | Bacteria | Proteobacteria | Gammaproteobacteria | Vibrionales | Vibrionaceae | Aliivibrio | fischeri |
| Otu000395 | 1 | 0.6667 | 0.816 | 0.007 | Bacteria | Proteobacteria | Gammaproteobacteria | Pseudomonadales | Moraxellaceae | Acinetobacter | guillouiae |
| Otu000424 | 1 | 1 | 1 | 0.001 | Bacteria | Proteobacteria | Gammaproteobacteria | unclassified | unclassified | unclassified | unclassified |
| Otu000425 | 1 | 1 | 1 | 0.001 | Bacteria | Bacteroidetes | Flavobacteriia | Flavobacteriales | Flavobacteriaceae | unclassified | unclassified |
| Otu000431 | 1 | 1 | 1 | 0.001 | Bacteria | Proteobacteria | Betaproteobacteria | Methylophilales | Methylophilaceae | Methylotenera | mobilis |
| Otu000473 | 1 | 1 | 1 | 0.001 | Bacteria | Bacteroidetes | Flavobacteriia | Flavobacteriales | unclassified | unclassified | unclassified |
| Otu000476 | 0.9683 | 1 | 0.984 | 0.001 | Bacteria | Proteobacteria | Alphaproteobacteria | Rhodobacterales | Rhodobacteraceae | unclassified | unclassified |
| Otu000479 | 1 | 1 | 1 | 0.001 | Bacteria | Proteobacteria | Gammaproteobacteria | Alteromonadales | Alteromonadaceae | HTCC2207 | unclassified |
| Otu000487 | 1 | 1 | 1 | 0.001 | Bacteria | Cyanobacteria | Synechococcophycideae | Synechococcales | Synechococcaceae | unclassified | unclassified |
| Otu000502 | 1 | 1 | 1 | 0.001 | Bacteria | Cyanobacteria | Synechococcophycideae | Synechococcales | Synechococcaceae | Synechococcus | unclassified |
| Otu000524 | 1 | 0.6667 | 0.816 | 0.007 | Bacteria | Cyanobacteria | Synechococcophycideae | Synechococcales | Synechococcaceae | Synechococcus | unclassified |
| Otu000596 | 1 | 1 | 1 | 0.001 | Bacteria | Verrucomicrobia | Opitutae | Puniceicoccales | Puniceicoccaceae | Coraliomargarita | unclassified |
| Otu000613 | 1 | 1 | 1 | 0.001 | Bacteria | Bacteroidetes | Flavobacteriia | Flavobacteriales | Flavobacteriaceae | unclassified | unclassified |
| Otu000615 | 1 | 1 | 1 | 0.001 | Bacteria | Bacteroidetes | Flavobacteriia | Flavobacteriales | Flavobacteriaceae | unclassified | unclassified |
| Otu000642 | 1 | 1 | 1 | 0.001 | Bacteria | Actinobacteria | Actinobacteria | Actinomycetales | unclassified | unclassified | unclassified |
| Otu000643 | 0.8994 | 1 | 0.948 | 0.003 | Bacteria | Proteobacteria | Gammaproteobacteria | Vibrionales | Pseudoalteromonadaceae | Pseudoalteromonas | unclassified |
| Otu000644 | 1 | 0.6667 | 0.816 | 0.009 | Bacteria | Bacteroidetes | unclassified | unclassified | unclassified | unclassified | unclassified |
| Otu000666 | 1 | 1 | 1 | 0.001 | Bacteria | unclassified | unclassified | unclassified | unclassified | unclassified | unclassified |
| Otu000674 | 1 | 1 | 1 | 0.001 | Bacteria | Bacteroidetes | unclassified | unclassified | unclassified | unclassified | unclassified |
| Otu000688 | 1 | 1 | 1 | 0.001 | Bacteria | Planctomycetes | Planctomycetia | Pirellulales | Pirellulaceae | unclassified | unclassified |
| Otu000713 | 1 | 1 | 1 | 0.001 | Bacteria | Cyanobacteria | Synechococcophycideae | Synechococcales | Synechococcaceae | unclassified | unclassified |
| Otu000715 | 1 | 1 | 1 | 0.001 | Bacteria | Proteobacteria | Gammaproteobacteria | Alteromonadales | HTCC2188 | HTCC | unclassified |
| Otu000719 | 1 | 0.6667 | 0.816 | 0.007 | Bacteria | Bacteroidetes | Flavobacteriia | Flavobacteriales | unclassified | unclassified | unclassified |
| Otu000727 | 1 | 1 | 1 | 0.001 | Bacteria | Bacteroidetes | Flavobacteriia | Flavobacteriales | unclassified | unclassified | unclassified |
| Otu000739 | 1 | 1 | 1 | 0.001 | Bacteria | Bacteroidetes | Flavobacteriia | Flavobacteriales | Flavobacteriaceae | Tenacibaculum | unclassified |
| Otu000755 | 1 | 0.6667 | 0.816 | 0.009 | Bacteria | Proteobacteria | Betaproteobacteria | Burkholderiales | Comamonadaceae | unclassified | unclassified |
| Otu000756 | 1 | 1 | 1 | 0.001 | Bacteria | Proteobacteria | unclassified | unclassified | unclassified | unclassified | unclassified |
| Otu000771 | 1 | 0.6667 | 0.816 | 0.01 | Bacteria | Cyanobacteria | Synechococcophycideae | Synechococcales | Synechococcaceae | unclassified | unclassified |

| | | | | | | | | | | | |
|---|---|---|---|---|---|---|---|---|---|---|---|
| Otu000775 | 1 | 1 | 1 | 0.001 | Bacteria | Proteobacteria | Alphaproteobacteria | Rickettsiales | Pelagibacteraceae | unclassified | unclassified |
| Otu000778 | 1 | 1 | 1 | 0.001 | Bacteria | Cyanobacteria | Synechococcophycideae | Synechococcales | Synechococcaceae | Synechococcus | unclassified |
| Otu000780 | 1 | 1 | 1 | 0.001 | Bacteria | Cyanobacteria | Synechococcophycideae | Synechococcales | Synechococcaceae | Synechococcus | unclassified |
| Otu000790 | 1 | 0.6667 | 0.816 | 0.01 | Bacteria | Bacteroidetes | Flavobacteriia | Flavobacteriales | Cryomorphaceae | unclassified | unclassified |
| Otu000805 | 1 | 1 | 1 | 0.001 | Bacteria | Proteobacteria | Gammaproteobacteria | unclassified | unclassified | unclassified | unclassified |
| Otu000810 | 1 | 1 | 1 | 0.001 | Bacteria | Proteobacteria | Alphaproteobacteria | unclassified | unclassified | unclassified | unclassified |
| Otu000839 | 1 | 1 | 1 | 0.001 | Bacteria | Cyanobacteria | Synechococcophycideae | Synechococcales | Synechococcaceae | Prochlorococcus | unclassified |
| Otu000841 | 1 | 1 | 1 | 0.001 | Bacteria | Bacteroidetes | Flavobacteriia | Flavobacteriales | Flavobacteriaceae | unclassified | unclassified |
| Otu000871 | 1 | 1 | 1 | 0.001 | Bacteria | Bacteroidetes | Flavobacteriia | Flavobacteriales | Flavobacteriaceae | Bizionia | unclassified |
| Otu000872 | 0.9373 | 0.6667 | 0.79 | 0.025 | Bacteria | Bacteroidetes | Flavobacteriia | Flavobacteriales | unclassified | unclassified | unclassified |
| Otu000873 | 1 | 1 | 1 | 0.001 | Bacteria | Bacteroidetes | Flavobacteriia | Flavobacteriales | Flavobacteriaceae | Formosa | unclassified |
| Otu000927 | 1 | 1 | 1 | 0.001 | Bacteria | Proteobacteria | Gammaproteobacteria | unclassified | unclassified | unclassified | unclassified |
| Otu000933 | 1 | 1 | 1 | 0.001 | Bacteria | Bacteroidetes | Flavobacteriia | Flavobacteriales | unclassified | unclassified | unclassified |
| Otu000934 | 1 | 0.6667 | 0.816 | 0.01 | Bacteria | Bacteroidetes | Saprospirae | Saprospirales | Saprospiraceae | unclassified | unclassified |
| Otu000965 | 0.9041 | 1 | 0.951 | 0.001 | Bacteria | Proteobacteria | unclassified | unclassified | unclassified | unclassified | unclassified |
| Otu000966 | 1 | 1 | 1 | 0.001 | Bacteria | Bacteroidetes | Flavobacteriia | Flavobacteriales | NS9 | unclassified | unclassified |
| Otu000998 | 1 | 0.6667 | 0.816 | 0.01 | Bacteria | Cyanobacteria | Synechococcophycideae | Synechococcales | Synechococcaceae | unclassified | unclassified |
| Otu001013 | 1 | 1 | 1 | 0.001 | Bacteria | Bacteroidetes | Flavobacteriia | Flavobacteriales | Flavobacteriaceae | Olleya | unclassified |
| Otu001021 | 0.7806 | 0.6667 | 0.721 | 0.027 | Bacteria | unclassified | unclassified | unclassified | unclassified | unclassified | unclassified |
| Otu001040 | 1 | 1 | 1 | 0.001 | Bacteria | Bacteroidetes | unclassified | unclassified | unclassified | unclassified | unclassified |
| Otu001050 | 1 | 1 | 1 | 0.001 | Bacteria | Cyanobacteria | Synechococcophycideae | Synechococcales | Synechococcaceae | Prochlorococcus | unclassified |
| Otu001055 | 1 | 1 | 1 | 0.001 | Bacteria | Actinobacteria | Actinobacteria | Actinomycetales | Microbacteriaceae | Candidatus_Aquiluna | rubra |
| Otu001066 | 1 | 1 | 1 | 0.001 | Bacteria | Bacteroidetes | Flavobacteriia | Flavobacteriales | Flavobacteriaceae | unclassified | unclassified |
| Otu001070 | 0.7532 | 1 | 0.868 | 0.017 | Bacteria | Proteobacteria | Gammaproteobacteria | Vibrionales | Vibrionaceae | Aliivibrio | fischeri |
| Otu001076 | 1 | 1 | 1 | 0.001 | Bacteria | Proteobacteria | Alphaproteobacteria | Rhodobacterales | Rhodobacteraceae | Pseudoruegeria | unclassified |
| Otu001080 | 0.9563 | 0.6667 | 0.798 | 0.009 | Bacteria | Proteobacteria | Gammaproteobacteria | unclassified | unclassified | unclassified | unclassified |
| Otu001128 | 1 | 0.6667 | 0.816 | 0.007 | Bacteria | Bacteroidetes | unclassified | unclassified | unclassified | unclassified | unclassified |
| Otu001136 | 1 | 1 | 1 | 0.001 | Bacteria | Proteobacteria | Gammaproteobacteria | unclassified | unclassified | unclassified | unclassified |
| Otu001147 | 1 | 1 | 1 | 0.001 | Bacteria | Cyanobacteria | Synechococcophycideae | Synechococcales | Synechococcaceae | unclassified | unclassified |
| Otu001150 | 1 | 1 | 1 | 0.001 | Bacteria | Proteobacteria | Gammaproteobacteria | Vibrionales | Vibrionaceae | Vibrio | unclassified |
| Otu001176 | 1 | 1 | 1 | 0.001 | Bacteria | Proteobacteria | Gammaproteobacteria | unclassified | unclassified | unclassified | unclassified |
| Otu001181 | 1 | 1 | 1 | 0.001 | Bacteria | Proteobacteria | Alphaproteobacteria | Rickettsiales | Pelagibacteraceae | unclassified | unclassified |
| Otu001190 | 1 | 0.6667 | 0.816 | 0.01 | Bacteria | Cyanobacteria | Synechococcophycideae | Synechococcales | Synechococcaceae | unclassified | unclassified |
| Otu001202 | 1 | 1 | 1 | 0.001 | Bacteria | Proteobacteria | Betaproteobacteria | Burkholderiales | unclassified | unclassified | unclassified |
| Otu001210 | 1 | 1 | 1 | 0.001 | Bacteria | Proteobacteria | Gammaproteobacteria | Alteromonadales | unclassified | unclassified | unclassified |
| Otu001240 | 1 | 1 | 1 | 0.001 | Bacteria | Bacteroidetes | unclassified | unclassified | unclassified | unclassified | unclassified |
| Otu001251 | 1 | 0.6667 | 0.816 | 0.007 | Bacteria | Cyanobacteria | Synechococcophycideae | Synechococcales | Synechococcaceae | Prochlorococcus | unclassified |
| Otu001252 | 1 | 1 | 1 | 0.001 | Bacteria | Cyanobacteria | Synechococcophycideae | Synechococcales | Synechococcaceae | unclassified | unclassified |
| Otu001292 | 1 | 1 | 1 | 0.001 | Bacteria | Cyanobacteria | Synechococcophycideae | Synechococcales | Synechococcaceae | unclassified | unclassified |
| Otu001293 | 1 | 1 | 1 | 0.001 | Bacteria | Cyanobacteria | Synechococcophycideae | Synechococcales | Synechococcaceae | unclassified | unclassified |
| Otu001313 | 1 | 1 | 1 | 0.001 | Bacteria | Proteobacteria | Alphaproteobacteria | Rickettsiales | Pelagibacteraceae | unclassified | unclassified |
| Otu001337 | 1 | 0.6667 | 0.816 | 0.007 | Bacteria | Proteobacteria | Gammaproteobacteria | Oceanospirillales | Halomonadaceae | Halomonas | unclassified |
| Otu001339 | 0.8303 | 1 | 0.911 | 0.009 | Bacteria | Cyanobacteria | Synechococcophycideae | Synechococcales | Synechococcaceae | Synechococcus | unclassified |
| Otu001365 | 1 | 1 | 1 | 0.001 | Bacteria | Bacteroidetes | Flavobacteriia | Flavobacteriales | Flavobacteriaceae | unclassified | unclassified |
| Otu001371 | 1 | 1 | 1 | 0.001 | Bacteria | Proteobacteria | Alphaproteobacteria | Rickettsiales | Pelagibacteraceae | unclassified | unclassified |
| Otu001383 | 1 | 1 | 1 | 0.001 | Bacteria | Cyanobacteria | Synechococcophycideae | Synechococcales | Synechococcaceae | unclassified | unclassified |
| Otu001390 | 1 | 1 | 1 | 0.001 | Bacteria | Proteobacteria | Alphaproteobacteria | Rhodobacterales | Rhodobacteraceae | Rhodobacter | unclassified |
| Otu001421 | 1 | 1 | 1 | 0.001 | Bacteria | Proteobacteria | unclassified | unclassified | unclassified | unclassified | unclassified |
| Otu001428 | 1 | 1 | 1 | 0.001 | Bacteria | Proteobacteria | Alphaproteobacteria | Rickettsiales | Pelagibacteraceae | unclassified | unclassified |
| Otu001438 | 1 | 1 | 1 | 0.001 | Bacteria | Proteobacteria | Gammaproteobacteria | Vibrionales | Vibrionaceae | Vibrio | shilonii |
| Otu001440 | 1 | 1 | 1 | 0.001 | Bacteria | Bacteroidetes | Flavobacteriia | Flavobacteriales | unclassified | unclassified | unclassified |
| Otu001453 | 1 | 1 | 1 | 0.001 | Bacteria | Bacteroidetes | Saprospirae | Saprospirales | Saprospiraceae | unclassified | unclassified |
| Otu001475 | 0.944 | 1 | 0.972 | 0.001 | Bacteria | Proteobacteria | Alphaproteobacteria | Rickettsiales | Rickettsiaceae | unclassified | unclassified |
| Otu001483 | 1 | 0.6667 | 0.816 | 0.007 | Bacteria | Cyanobacteria | Synechococcophycideae | Synechococcales | Synechococcaceae | unclassified | unclassified |
| Otu001501 | 1 | 1 | 1 | 0.001 | Bacteria | Bacteroidetes | Flavobacteriia | Flavobacteriales | Flavobacteriaceae | unclassified | unclassified |
| Otu001556 | 1 | 1 | 1 | 0.001 | Bacteria | Proteobacteria | Alphaproteobacteria | Rhodobacterales | Rhodobacteraceae | unclassified | unclassified |
| Otu001637 | 1 | 1 | 1 | 0.001 | Bacteria | Bacteroidetes | Flavobacteriia | Flavobacteriales | Flavobacteriaceae | unclassified | unclassified |
| Otu001641 | 1 | 0.6667 | 0.816 | 0.01 | Bacteria | Planctomycetes | Planctomycetia | Pirellulales | Pirellulaceae | unclassified | unclassified |
| Otu001706 | 1 | 1 | 1 | 0.001 | Bacteria | Proteobacteria | Gammaproteobacteria | HTCC2188 | HTCC2089 | unclassified | unclassified |
| Otu001710 | 1 | 1 | 1 | 0.001 | Bacteria | Proteobacteria | unclassified | unclassified | unclassified | unclassified | unclassified |
| Otu001726 | 1 | 1 | 1 | 0.001 | Bacteria | Cyanobacteria | Synechococcophycideae | Synechococcales | Synechococcaceae | unclassified | unclassified |
| Otu001740 | 1 | 1 | 1 | 0.001 | Bacteria | Proteobacteria | unclassified | unclassified | unclassified | unclassified | unclassified |
| Otu001741 | 1 | 0.6667 | 0.816 | 0.009 | Bacteria | Proteobacteria | Gammaproteobacteria | unclassified | unclassified | unclassified | unclassified |
| Otu001763 | 1 | 1 | 1 | 0.001 | Bacteria | Bacteroidetes | Flavobacteriia | Flavobacteriales | Flavobacteriaceae | unclassified | unclassified |
| Otu001781 | 1 | 0.6667 | 0.816 | 0.007 | Bacteria | Proteobacteria | Gammaproteobacteria | Alteromonadales | Alteromonadaceae | nsmpVI18 | unclassified |
| Otu001783 | 1 | 0.6667 | 0.816 | 0.007 | Bacteria | Proteobacteria | Gammaproteobacteria | unclassified | unclassified | unclassified | unclassified |
| Otu001785 | 1 | 0.6667 | 0.816 | 0.009 | Bacteria | Proteobacteria | Gammaproteobacteria | Alteromonadales | unclassified | unclassified | unclassified |
| Otu001829 | 1 | 1 | 1 | 0.001 | Bacteria | Proteobacteria | Alphaproteobacteria | unclassified | unclassified | unclassified | unclassified |
| Otu001854 | 1 | 0.6667 | 0.816 | 0.007 | Bacteria | Cyanobacteria | Synechococcophycideae | Synechococcales | Synechococcaceae | Synechococcus | unclassified |
| Otu001920 | 1 | 1 | 1 | 0.001 | Bacteria | Proteobacteria | Alphaproteobacteria | unclassified | unclassified | unclassified | unclassified |
| Otu001925 | 1 | 1 | 1 | 0.001 | Bacteria | Proteobacteria | Gammaproteobacteria | Pseudomonadales | Moraxellaceae | Acinetobacter | johnsonii |
| Otu001931 | 1 | 0.6667 | 0.816 | 0.01 | Bacteria | Bacteroidetes | Flavobacteriia | Flavobacteriales | unclassified | unclassified | unclassified |
| Otu001971 | 0.8488 | 0.6667 | 0.752 | 0.04 | Bacteria | Cyanobacteria | Synechococcophycideae | Synechococcales | Synechococcaceae | unclassified | unclassified |
| Otu001972 | 1 | 1 | 1 | 0.001 | Bacteria | Cyanobacteria | Synechococcophycideae | Synechococcales | Synechococcaceae | Synechococcus | unclassified |
| Otu001973 | 0.8842 | 1 | 0.94 | 0.004 | Bacteria | Cyanobacteria | Synechococcophycideae | Synechococcales | Synechococcaceae | unclassified | unclassified |
| Otu001974 | 1 | 1 | 1 | 0.001 | Bacteria | Cyanobacteria | Synechococcophycideae | Synechococcales | Synechococcaceae | unclassified | unclassified |
| Otu002008 | 1 | 1 | 1 | 0.001 | Bacteria | Proteobacteria | Gammaproteobacteria | Alteromonadales | Shewanellaceae | Shewanella | benthica |
| Otu002025 | 1 | 1 | 1 | 0.001 | Bacteria | Cyanobacteria | Synechococcophycideae | Synechococcales | Synechococcaceae | Synechococcus | unclassified |
| Otu002085 | 0.9527 | 1 | 0.976 | 0.001 | Bacteria | Bacteroidetes | Flavobacteriia | Flavobacteriales | Cryomorphaceae | Fluviicola | unclassified |
| Otu002107 | 1 | 0.6667 | 0.816 | 0.007 | Bacteria | Proteobacteria | Gammaproteobacteria | unclassified | unclassified | unclassified | unclassified |
| Otu002112 | 1 | 0.6667 | 0.816 | 0.01 | Bacteria | Proteobacteria | Gammaproteobacteria | Alteromonadales | OM60 | Congregibacter | unclassified |
| Otu002174 | 1 | 1 | 1 | 0.001 | Bacteria | Proteobacteria | Gammaproteobacteria | Oceanospirillales | Oceanospirillaceae | Marinomonas | unclassified |
| Otu002180 | 1 | 1 | 1 | 0.001 | Bacteria | Proteobacteria | Alphaproteobacteria | Rickettsiales | Pelagibacteraceae | unclassified | unclassified |
| Otu002182 | 1 | 0.6667 | 0.816 | 0.007 | Bacteria | Cyanobacteria | Synechococcophycideae | Synechococcales | Synechococcaceae | unclassified | unclassified |
| Otu002194 | 1 | 1 | 1 | 0.001 | Bacteria | Proteobacteria | Gammaproteobacteria | Alteromonadales | unclassified | unclassified | unclassified |
| Otu002205 | 1 | 0.6667 | 0.816 | 0.009 | Bacteria | Proteobacteria | Gammaproteobacteria | unclassified | unclassified | unclassified | unclassified |
| Otu002251 | 1 | 0.6667 | 0.816 | 0.009 | Bacteria | Proteobacteria | Alphaproteobacteria | Rickettsiales | Pelagibacteraceae | unclassified | unclassified |
| Otu002252 | 1 | 0.6667 | 0.816 | 0.007 | Bacteria | Proteobacteria | Alphaproteobacteria | Rhodobacterales | Rhodobacteraceae | unclassified | unclassified |
| Otu002258 | 1 | 0.6667 | 0.816 | 0.01 | Bacteria | Cyanobacteria | Synechococcophycideae | Synechococcales | Synechococcaceae | unclassified | unclassified |
| Otu002267 | 1 | 1 | 1 | 0.001 | Bacteria | Verrucomicrobia | Verrucomicrobiae | Verrucomicrobiales | Verrucomicrobiaceae | unclassified | unclassified |
| Otu002306 | 1 | 1 | 1 | 0.001 | Bacteria | Actinobacteria | Actinobacteria | Actinomycetales | Microbacteriaceae | Candidatus_Rhodoluna | unclassified |
| Otu002330 | 1 | 0.6667 | 0.816 | 0.009 | Bacteria | Proteobacteria | Gammaproteobacteria | Alteromonadales | HTCC2188 | HTCC | unclassified |
| Otu002379 | 1 | 1 | 1 | 0.001 | Bacteria | Cyanobacteria | Synechococcophycideae | Synechococcales | Synechococcaceae | unclassified | unclassified |
| Otu002381 | 1 | 1 | 1 | 0.001 | Bacteria | Proteobacteria | Alphaproteobacteria | Rhodobacterales | Rhodobacteraceae | unclassified | unclassified |
| Otu002404 | 1 | 1 | 1 | 0.001 | Bacteria | Bacteroidetes | unclassified | unclassified | unclassified | unclassified | unclassified |
| Otu002424 | 1 | 1 | 1 | 0.001 | Bacteria | Cyanobacteria | Synechococcophycideae | Synechococcales | Synechococcaceae | Synechococcus | unclassified |
| Otu002444 | 0.9797 | 1 | 0.99 | 0.001 | Bacteria | Proteobacteria | Betaproteobacteria | Burkholderiales | Comamonadaceae | unclassified | unclassified |
| Otu002452 | 1 | 1 | 1 | 0.001 | Bacteria | Proteobacteria | Betaproteobacteria | Burkholderiales | Comamonadaceae | unclassified | unclassified |
| Otu002468 | 1 | 1 | 1 | 0.001 | Bacteria | Proteobacteria | Alphaproteobacteria | Rickettsiales | Pelagibacteraceae | unclassified | unclassified |
| Otu002477 | 0.8479 | 1 | 0.921 | 0.002 | Bacteria | Verrucomicrobia | Verrucomicrobiae | Verrucomicrobiales | Verrucomicrobiaceae | unclassified | unclassified |

| | | | | | | | | | | | |
|---|---|---|---|---|---|---|---|---|---|---|---|
| Otu002523 | 0.7642 | 1 | 0.874 | 0.03 | Bacteria | Verrucomicrobia | Verrucomicrobiae | Verrucomicrobiales | Verrucomicrobiaceae | unclassified | unclassified |
| Otu002529 | 1 | 1 | 1 | 0.001 | Bacteria | Bacteroidetes | Flavobacteriia | Flavobacteriales | Cryomorphaceae | Fluviicola | unclassified |
| Otu002536 | 1 | 1 | 1 | 0.001 | Bacteria | Verrucomicrobia | Verrucomicrobiae | Verrucomicrobiales | Verrucomicrobiaceae | unclassified | unclassified |
| Otu002550 | 0.9315 | 1 | 0.965 | 0.001 | Bacteria | Cyanobacteria | Synechococcophycideae | Synechococcales | Synechococcaceae | Synechococcus | unclassified |
| Otu002555 | 1 | 1 | 1 | 0.001 | Bacteria | Verrucomicrobia | Verrucomicrobiae | Verrucomicrobiales | Verrucomicrobiaceae | unclassified | unclassified |
| Otu002606 | 1 | 1 | 1 | 0.001 | Bacteria | Proteobacteria | Betaproteobacteria | unclassified | unclassified | unclassified | unclassified |
| Otu002666 | 1 | 1 | 1 | 0.001 | Bacteria | Proteobacteria | Gammaproteobacteria | Alteromonadales | OM60 | Congregibacter | unclassified |
| Otu002675 | 1 | 0.6667 | 0.816 | 0.007 | Bacteria | Cyanobacteria | Synechococcophycideae | Synechococcales | Synechococcaceae | Synechococcus | unclassified |
| Otu002696 | 0.9001 | 0.6667 | 0.775 | 0.011 | Bacteria | Cyanobacteria | Synechococcophycideae | Synechococcales | Synechococcaceae | Synechococcus | unclassified |
| Otu002701 | 0.9477 | 1 | 0.973 | 0.001 | Bacteria | Tenericutes | Mollicutes | Acholeplasmatales | Acholeplasmataceae | Acholeplasma | unclassified |
| Otu002708 | 1 | 1 | 1 | 0.001 | Bacteria | Bacteroidetes | Rhodothermi | Rhodothermales | Balneolaceae | Balneola | unclassified |
| Otu002719 | 1 | 1 | 1 | 0.001 | Bacteria | Verrucomicrobia | Opitutae | Puniceicoccales | Puniceicoccaceae | Coraliomargarita | unclassified |
| Otu002744 | 1 | 0.6667 | 0.816 | 0.009 | Bacteria | Proteobacteria | Gammaproteobacteria | Marinicellales | Marinicellaceae | Marinicella | unclassified |
| Otu002760 | 1 | 0.6667 | 0.816 | 0.01 | Bacteria | Cyanobacteria | Synechococcophycideae | Synechococcales | Synechococcaceae | Synechococcus | unclassified |
| Otu002761 | 0.9125 | 1 | 0.955 | 0.006 | Bacteria | Cyanobacteria | Synechococcophycideae | Synechococcales | Synechococcaceae | unclassified | unclassified |
| Otu002781 | 0.9179 | 1 | 0.958 | 0.001 | Bacteria | Bacteroidetes | Flavobacteriia | Flavobacteriales | Flavobacteriaceae | unclassified | unclassified |
| Otu002798 | 0.9298 | 1 | 0.964 | 0.003 | Bacteria | Proteobacteria | Deltaproteobacteria | unclassified | unclassified | unclassified | unclassified |
| Otu002805 | 1 | 1 | 1 | 0.001 | Bacteria | Proteobacteria | Gammaproteobacteria | unclassified | unclassified | unclassified | unclassified |
| Otu002832 | 1 | 1 | 1 | 0.001 | Bacteria | Proteobacteria | Alphaproteobacteria | Rickettsiales | Pelagibacteraceae | unclassified | unclassified |
| Otu002833 | 1 | 0.6667 | 0.816 | 0.007 | Bacteria | Proteobacteria | Gammaproteobacteria | unclassified | unclassified | unclassified | unclassified |
| Otu002843 | 1 | 1 | 1 | 0.001 | Bacteria | Proteobacteria | Gammaproteobacteria | unclassified | unclassified | unclassified | unclassified |
| Otu002883 | 1 | 1 | 1 | 0.001 | Bacteria | Cyanobacteria | Synechococcophycideae | Synechococcales | Synechococcaceae | Synechococcus | unclassified |
| Otu002964 | 1 | 1 | 1 | 0.001 | Bacteria | Bacteroidetes | Flavobacteriia | Flavobacteriales | Flavobacteriaceae | unclassified | unclassified |
| Otu002966 | 1 | 1 | 1 | 0.001 | Bacteria | Bacteroidetes | Flavobacteriia | Flavobacteriales | NS9 | unclassified | unclassified |
| Otu002991 | 0.9691 | 1 | 0.984 | 0.001 | Bacteria | Bacteroidetes | Flavobacteriia | Flavobacteriales | Flavobacteriaceae | Formosa | unclassified |
| Otu002999 | 1 | 1 | 1 | 0.001 | Bacteria | Bacteroidetes | Flavobacteriia | Flavobacteriales | Cryomorphaceae | unclassified | unclassified |
| Otu003089 | 0.8495 | 0.6667 | 0.753 | 0.043 | Bacteria | Cyanobacteria | Synechococcophycideae | Synechococcales | Synechococcaceae | Synechococcus | unclassified |
| Otu003091 | 1 | 1 | 1 | 0.001 | Bacteria | Cyanobacteria | Synechococcophycideae | Synechococcales | Synechococcaceae | unclassified | unclassified |
| Otu003132 | 0.9918 | 1 | 0.996 | 0.001 | Bacteria | Bacteroidetes | Saprospirae | Saprospirales | Saprospiraceae | unclassified | unclassified |
| Otu003160 | 1 | 0.6667 | 0.816 | 0.009 | Bacteria | Proteobacteria | Alphaproteobacteria | Sphingomonadales | Erythrobacteraceae | unclassified | unclassified |
| Otu003217 | 1 | 0.6667 | 0.816 | 0.009 | Bacteria | Proteobacteria | Gammaproteobacteria | Oceanospirillales | unclassified | unclassified | unclassified |
| Otu003266 | 1 | 0.6667 | 0.816 | 0.009 | Bacteria | Bacteroidetes | Flavobacteriia | Flavobacteriales | Flavobacteriaceae | unclassified | unclassified |
| Otu003267 | 1 | 0.6667 | 0.816 | 0.009 | Bacteria | Bacteroidetes | Flavobacteriia | Flavobacteriales | Flavobacteriaceae | Lutimonas | unclassified |
| Otu003322 | 1 | 1 | 1 | 0.001 | Bacteria | Proteobacteria | Gammaproteobacteria | unclassified | unclassified | unclassified | unclassified |
| Otu003331 | 0.9202 | 1 | 0.959 | 0.001 | Bacteria | Actinobacteria | Actinobacteria | Actinomycetales | Microbacteriaceae | Candidatus_Aquiluna | rubra |
| Otu003362 | 0.9488 | 0.6667 | 0.795 | 0.013 | Bacteria | Proteobacteria | Alphaproteobacteria | Rhodobacterales | unclassified | unclassified | unclassified |
| Otu003364 | 1 | 1 | 1 | 0.001 | Bacteria | Cyanobacteria | Synechococcophycideae | Synechococcales | Synechococcaceae | unclassified | unclassified |
| Otu003367 | 1 | 0.6667 | 0.816 | 0.007 | Bacteria | Cyanobacteria | Synechococcophycideae | Synechococcales | Synechococcaceae | unclassified | unclassified |
| Otu003394 | 1 | 1 | 1 | 0.001 | Bacteria | Cyanobacteria | Synechococcophycideae | Synechococcales | Synechococcaceae | unclassified | unclassified |
| Otu003509 | 1 | 1 | 1 | 0.001 | Bacteria | Proteobacteria | Alphaproteobacteria | Rhodobacterales | Rhodobacteraceae | unclassified | unclassified |
| Otu003606 | 0.9751 | 1 | 0.987 | 0.001 | Bacteria | Cyanobacteria | unclassified | unclassified | unclassified | unclassified | unclassified |
| Otu003653 | 1 | 0.6667 | 0.816 | 0.007 | Bacteria | Proteobacteria | Gammaproteobacteria | Alteromonadales | unclassified | unclassified | unclassified |
| Otu003657 | 0.7859 | 1 | 0.887 | 0.012 | Bacteria | Proteobacteria | Gammaproteobacteria | Vibrionales | Pseudoalteromonadaceae | Pseudoalteromonas | tunicata |
| Otu003678 | 0.9243 | 0.6667 | 0.785 | 0.023 | Bacteria | Proteobacteria | Gammaproteobacteria | unclassified | unclassified | unclassified | unclassified |
| Otu003690 | 1 | 1 | 1 | 0.001 | Bacteria | Cyanobacteria | Synechococcophycideae | Synechococcales | Synechococcaceae | Synechococcus | unclassified |
| Otu003756 | 1 | 1 | 1 | 0.001 | Bacteria | Bacteroidetes | Flavobacteriia | Flavobacteriales | Flavobacteriaceae | unclassified | unclassified |
| Otu003775 | 1 | 0.6667 | 0.816 | 0.007 | Bacteria | Proteobacteria | Gammaproteobacteria | Vibrionales | Pseudoalteromonadaceae | Pseudoalteromonas | piscicida |
| Otu003794 | 1 | 1 | 1 | 0.001 | Bacteria | Bacteroidetes | Flavobacteriia | Flavobacteriales | Cryomorphaceae | unclassified | unclassified |
| Otu003797 | 1 | 1 | 1 | 0.001 | Bacteria | Bacteroidetes | Flavobacteriia | Flavobacteriales | Flavobacteriaceae | unclassified | unclassified |
| Otu003798 | 0.9396 | 0.6667 | 0.791 | 0.016 | Bacteria | unclassified | unclassified | unclassified | unclassified | unclassified | unclassified |
| Otu003813 | 0.7795 | 0.6667 | 0.721 | 0.036 | Bacteria | Cyanobacteria | Synechococcophycideae | Synechococcales | Synechococcaceae | unclassified | unclassified |
| Otu003814 | 1 | 1 | 1 | 0.001 | Bacteria | Cyanobacteria | Synechococcophycideae | Synechococcales | Synechococcaceae | Synechococcus | unclassified |
| Otu003827 | 1 | 0.6667 | 0.816 | 0.01 | Bacteria | Proteobacteria | Alphaproteobacteria | Rhodospirillales | Rhodospirillaceae | unclassified | unclassified |
| Otu003831 | 1 | 1 | 1 | 0.001 | Bacteria | Bacteroidetes | Flavobacteriia | Flavobacteriales | Flavobacteriaceae | unclassified | unclassified |
| Otu003833 | 1 | 0.6667 | 0.816 | 0.007 | Bacteria | Bacteroidetes | Flavobacteriia | Flavobacteriales | unclassified | unclassified | unclassified |
| Otu003842 | 1 | 1 | 1 | 0.001 | Bacteria | Proteobacteria | Gammaproteobacteria | unclassified | unclassified | unclassified | unclassified |
| Otu003860 | 1 | 1 | 1 | 0.001 | Bacteria | Bacteroidetes | unclassified | unclassified | unclassified | unclassified | unclassified |
| Otu003933 | 1 | 1 | 1 | 0.001 | Archaea | Euryarchaeota | Thermoplasmata | E2 | Marine_group_II | unclassified | unclassified |
| Otu004084 | 0.9291 | 1 | 0.964 | 0.001 | Bacteria | Proteobacteria | Gammaproteobacteria | unclassified | unclassified | unclassified | unclassified |
| Otu004097 | 1 | 1 | 1 | 0.001 | Bacteria | Proteobacteria | Gammaproteobacteria | Alteromonadales | unclassified | unclassified | unclassified |
| Otu004099 | 1 | 1 | 1 | 0.001 | Bacteria | Proteobacteria | Alphaproteobacteria | Rhodobacterales | Rhodobacteraceae | unclassified | unclassified |
| Otu004101 | 0.9107 | 1 | 0.954 | 0.001 | Bacteria | Proteobacteria | Alphaproteobacteria | Rhodobacterales | unclassified | unclassified | unclassified |
| Otu004171 | 1 | 0.6667 | 0.816 | 0.007 | Bacteria | Cyanobacteria | Synechococcophycideae | Synechococcales | Synechococcaceae | unclassified | unclassified |
| Otu004180 | 1 | 1 | 1 | 0.001 | Bacteria | Bacteroidetes | Flavobacteriia | Flavobacteriales | Flavobacteriaceae | unclassified | unclassified |
| Otu004191 | 1 | 1 | 1 | 0.001 | Bacteria | Proteobacteria | Alphaproteobacteria | Rhodospirillales | Rhodospirillaceae | unclassified | unclassified |
| Otu004226 | 1 | 1 | 1 | 0.001 | Bacteria | Bacteroidetes | Cytophagia | Cytophagales | Flammeovirgaceae | unclassified | unclassified |
| Otu004322 | 0.7944 | 1 | 0.891 | 0.009 | Bacteria | Proteobacteria | Alphaproteobacteria | unclassified | unclassified | unclassified | unclassified |
| Otu004326 | 1 | 1 | 1 | 0.001 | Bacteria | Proteobacteria | unclassified | unclassified | unclassified | unclassified | unclassified |
| Otu004398 | 1 | 0.6667 | 0.816 | 0.007 | Bacteria | Proteobacteria | Gammaproteobacteria | Oceanospirillales | Halomonadaceae | Candidatus_Portiera | unclassified |
| Otu004402 | 1 | 1 | 1 | 0.001 | Bacteria | Proteobacteria | Gammaproteobacteria | unclassified | unclassified | unclassified | unclassified |
| Otu004498 | 1 | 1 | 1 | 0.001 | Bacteria | Proteobacteria | Gammaproteobacteria | HTCC2188 | HTCC2089 | unclassified | unclassified |
| Otu004508 | 1 | 0.6667 | 0.816 | 0.009 | Bacteria | Proteobacteria | Gammaproteobacteria | Vibrionales | Vibrionaceae | Vibrio | shilonii |
| Otu004525 | 1 | 1 | 1 | 0.001 | Bacteria | Actinobacteria | Acidimicrobiia | Acidimicrobiales | OCS155 | unclassified | unclassified |
| Otu004548 | 1 | 1 | 1 | 0.001 | Bacteria | Cyanobacteria | Synechococcophycideae | Synechococcales | Synechococcaceae | Synechococcus | unclassified |
| Otu004549 | 0.9079 | 0.6667 | 0.778 | 0.024 | Bacteria | Cyanobacteria | Synechococcophycideae | Synechococcales | Synechococcaceae | unclassified | unclassified |
| Otu004551 | 1 | 0.6667 | 0.816 | 0.009 | Bacteria | Cyanobacteria | Synechococcophycideae | Synechococcales | Synechococcaceae | Synechococcus | unclassified |
| Otu004593 | 1 | 1 | 1 | 0.001 | Bacteria | Proteobacteria | Gammaproteobacteria | unclassified | unclassified | unclassified | unclassified |
| Otu004640 | 0.9554 | 1 | 0.977 | 0.001 | Bacteria | Proteobacteria | Gammaproteobacteria | unclassified | unclassified | unclassified | unclassified |
| Otu004654 | 0.9492 | 0.6667 | 0.795 | 0.014 | Bacteria | Cyanobacteria | Synechococcophycideae | Synechococcales | Synechococcaceae | unclassified | unclassified |
| Otu004686 | 1 | 1 | 1 | 0.001 | Bacteria | Proteobacteria | Betaproteobacteria | Methylophilales | Methylophilaceae | unclassified | unclassified |
| Otu004707 | 1 | 1 | 1 | 0.001 | Bacteria | Bacteroidetes | Flavobacteriia | Flavobacteriales | Flavobacteriaceae | Bizionia | unclassified |
| Otu004729 | 1 | 1 | 1 | 0.001 | Bacteria | Proteobacteria | Deltaproteobacteria | Sva0853 | S25_1238 | unclassified | unclassified |
| Otu004808 | 1 | 0.6667 | 0.816 | 0.009 | Bacteria | Proteobacteria | Gammaproteobacteria | Oceanospirillales | Endozoicimonaceae | unclassified | unclassified |
| Otu004873 | 0.8533 | 1 | 0.924 | 0.001 | Bacteria | Proteobacteria | Alphaproteobacteria | Rickettsiales | Pelagibacteraceae | unclassified | unclassified |
| Otu004919 | 1 | 0.6667 | 0.816 | 0.009 | Bacteria | Proteobacteria | Alphaproteobacteria | unclassified | unclassified | unclassified | unclassified |
| Otu004928 | 1 | 1 | 1 | 0.001 | Bacteria | Proteobacteria | Gammaproteobacteria | Oceanospirillales | Halomonadaceae | Candidatus_Portiera | unclassified |
| Otu004929 | 0.9349 | 1 | 0.967 | 0.001 | Bacteria | Proteobacteria | Gammaproteobacteria | Oceanospirillales | unclassified | unclassified | unclassified |
| Otu004930 | 1 | 1 | 1 | 0.001 | Bacteria | Proteobacteria | Gammaproteobacteria | Oceanospirillales | unclassified | unclassified | unclassified |
| Otu004996 | 0.9388 | 0.6667 | 0.791 | 0.015 | Bacteria | Cyanobacteria | Synechococcophycideae | Synechococcales | Synechococcaceae | unclassified | unclassified |
| Otu005046 | 1 | 1 | 1 | 0.001 | Bacteria | Bacteroidetes | Flavobacteriia | Flavobacteriales | unclassified | unclassified | unclassified |
| Otu005055 | 0.9562 | 1 | 0.978 | 0.001 | Bacteria | Proteobacteria | Gammaproteobacteria | unclassified | unclassified | unclassified | unclassified |
| Otu005305 | 0.7742 | 1 | 0.88 | 0.013 | Bacteria | Cyanobacteria | unclassified | unclassified | unclassified | unclassified | unclassified |
| Otu005347 | 1 | 1 | 1 | 0.001 | Bacteria | Proteobacteria | Deltaproteobacteria | PB19 | unclassified | unclassified | unclassified |
| Otu005371 | 1 | 1 | 1 | 0.001 | Bacteria | Bacteroidetes | unclassified | unclassified | unclassified | unclassified | unclassified |
| Otu005405 | 1 | 0.6667 | 0.816 | 0.01 | Bacteria | Proteobacteria | unclassified | unclassified | unclassified | unclassified | unclassified |
| Otu005496 | 1 | 1 | 1 | 0.001 | Bacteria | Bacteroidetes | Flavobacteriia | Flavobacteriales | unclassified | unclassified | unclassified |
| Otu005544 | 0.9318 | 0.6667 | 0.788 | 0.022 | Bacteria | Cyanobacteria | Synechococcophycideae | Synechococcales | Synechococcaceae | unclassified | unclassified |
| Otu005548 | 1 | 0.6667 | 0.816 | 0.009 | Bacteria | Proteobacteria | unclassified | unclassified | unclassified | unclassified | unclassified |
| Otu005692 | 1 | 1 | 1 | 0.001 | Bacteria | Proteobacteria | Gammaproteobacteria | Thiotrichales | Piscirickettsiaceae | unclassified | unclassified |
| Otu005701 | 0.9641 | 1 | 0.982 | 0.001 | Bacteria | Proteobacteria | Gammaproteobacteria | Alteromonadales | Alteromonadaceae | HTCC2207 | unclassified |

| | | | | | | | | | | | |
|---|---|---|---|---|---|---|---|---|---|---|---|
| Otu005838 | 1 | 1 | 1 | 0.001 | Bacteria | Proteobacteria | Gammaproteobacteria | unclassified | unclassified | unclassified | unclassified |
| Otu005924 | 0.9584 | 1 | 0.979 | 0.001 | Bacteria | Proteobacteria | Gammaproteobacteria | Oceanospirillales | unclassified | unclassified | unclassified |
| Otu005947 | 1 | 0.6667 | 0.816 | 0.009 | Bacteria | unclassified | unclassified | unclassified | unclassified | unclassified | unclassified |
| Otu005974 | 1 | 1 | 1 | 0.001 | Bacteria | Cyanobacteria | Synechococcophycideae | Synechococcales | Synechococcaceae | Prochlorococcus | unclassified |
| Otu005987 | 1 | 1 | 1 | 0.001 | Bacteria | Proteobacteria | Alphaproteobacteria | Rhodobacterales | Rhodobacteraceae | Phaeobacter | unclassified |
| Otu006005 | 1 | 0.6667 | 0.816 | 0.009 | Bacteria | Proteobacteria | Gammaproteobacteria | Alteromonadales | Colwelliaceae | unclassified | unclassified |
| Otu006013 | 1 | 1 | 1 | 0.001 | Bacteria | Proteobacteria | Gammaproteobacteria | Alteromonadales | unclassified | unclassified | unclassified |
| Otu006030 | 1 | 1 | 1 | 0.001 | Bacteria | Verrucomicrobia | Pedosphaerae | Pedosphaerales | unclassified | unclassified | unclassified |
| Otu006057 | 1 | 1 | 1 | 0.001 | Bacteria | Proteobacteria | Gammaproteobacteria | Alteromonadales | Alteromonadaceae | ZD0117 | unclassified |
| Otu006073 | 1 | 1 | 1 | 0.001 | Bacteria | Proteobacteria | Gammaproteobacteria | Alteromonadales | unclassified | unclassified | unclassified |
| Otu006100 | 1 | 0.6667 | 0.816 | 0.009 | Bacteria | Proteobacteria | Alphaproteobacteria | Rickettsiales | Pelagibacteraceae | unclassified | unclassified |
| Otu006161 | 0.9767 | 1 | 0.988 | 0.001 | Bacteria | Proteobacteria | Gammaproteobacteria | unclassified | unclassified | unclassified | unclassified |
| Otu006243 | 0.9863 | 1 | 0.993 | 0.001 | Bacteria | Bacteroidetes | Flavobacteriia | Flavobacteriales | Flavobacteriaceae | unclassified | unclassified |
| Otu006244 | 0.9516 | 1 | 0.975 | 0.001 | Bacteria | Bacteroidetes | Flavobacteriia | Flavobacteriales | Cryomorphaceae | unclassified | unclassified |
| Otu006306 | 1 | 1 | 1 | 0.001 | Bacteria | Proteobacteria | unclassified | unclassified | unclassified | unclassified | unclassified |
| Otu006390 | 0.8539 | 0.6667 | 0.754 | 0.041 | Bacteria | Bacteroidetes | Flavobacteriia | Flavobacteriales | NS9 | unclassified | unclassified |
| Otu006520 | 0.7518 | 1 | 0.867 | 0.001 | Bacteria | Proteobacteria | Gammaproteobacteria | unclassified | unclassified | unclassified | unclassified |
| Otu006562 | 1 | 0.6667 | 0.816 | 0.009 | Bacteria | Actinobacteria | Acidimicrobiia | Acidimicrobiales | JdFBGBact | unclassified | unclassified |
| Otu006582 | 1 | 1 | 1 | 0.001 | Bacteria | Proteobacteria | Alphaproteobacteria | Rhodospirillales | Rhodospirillaceae | unclassified | unclassified |
| Otu006617 | 0.9166 | 0.6667 | 0.782 | 0.02 | Bacteria | Proteobacteria | Gammaproteobacteria | unclassified | unclassified | unclassified | unclassified |
| Otu006634 | 0.923 | 1 | 0.961 | 0.003 | Bacteria | Proteobacteria | Gammaproteobacteria | Alteromonadales | unclassified | unclassified | unclassified |
| Otu006646 | 0.9609 | 0.6667 | 0.8 | 0.01 | Bacteria | Proteobacteria | Gammaproteobacteria | unclassified | unclassified | unclassified | unclassified |
| Otu006694 | 0.9761 | 1 | 0.988 | 0.001 | Bacteria | Bacteroidetes | unclassified | unclassified | unclassified | unclassified | unclassified |
| Otu006696 | 0.986 | 1 | 0.993 | 0.001 | Bacteria | Bacteroidetes | Flavobacteriia | Flavobacteriales | Cryomorphaceae | unclassified | unclassified |
| Otu006698 | 1 | 1 | 1 | 0.001 | Bacteria | Proteobacteria | Gammaproteobacteria | Pseudomonadales | Moraxellaceae | Acinetobacter | venetianus |
| Otu006712 | 1 | 1 | 1 | 0.001 | Bacteria | Proteobacteria | Alphaproteobacteria | Kiloniellales | unclassified | unclassified | unclassified |
| Otu006719 | 1 | 1 | 1 | 0.001 | Bacteria | Bacteroidetes | Flavobacteriia | Flavobacteriales | Flavobacteriaceae | unclassified | unclassified |
| Otu006728 | 0.9672 | 1 | 0.983 | 0.001 | Bacteria | Bacteroidetes | Flavobacteriia | Flavobacteriales | Cryomorphaceae | Fluviicola | unclassified |
| Otu006773 | 1 | 1 | 1 | 0.001 | Bacteria | Actinobacteria | Acidimicrobiia | Acidimicrobiales | OCS155 | unclassified | unclassified |
| Otu006780 | 1 | 1 | 1 | 0.001 | Bacteria | Bacteroidetes | Sphingobacteriia | Sphingobacteriales | NS11-12 | unclassified | unclassified |
| Otu006786 | 0.9715 | 1 | 0.986 | 0.001 | Bacteria | Bacteroidetes | Flavobacteriia | Flavobacteriales | Cryomorphaceae | Crocinitomix | unclassified |
| Otu006789 | 0.9413 | 0.6667 | 0.792 | 0.015 | Bacteria | Bacteroidetes | Flavobacteriia | Flavobacteriales | Flavobacteriaceae | Tenacibaculum | unclassified |
| Otu006793 | 1 | 0.6667 | 0.816 | 0.009 | Bacteria | Bacteroidetes | unclassified | unclassified | unclassified | unclassified | unclassified |
| Otu006795 | 0.873 | 1 | 0.934 | 0.001 | Bacteria | Bacteroidetes | unclassified | unclassified | unclassified | unclassified | unclassified |
| Otu006836 | 1 | 0.6667 | 0.816 | 0.009 | Bacteria | Proteobacteria | Alphaproteobacteria | Rickettsiales | Pelagibacteraceae | unclassified | unclassified |
| Otu006846 | 1 | 0.6667 | 0.816 | 0.009 | Bacteria | Proteobacteria | Gammaproteobacteria | Alteromonadales | Alteromonadaceae | Alteromonas | unclassified |
| Otu006966 | 0.7209 | 1 | 0.849 | 0.03 | Bacteria | Proteobacteria | unclassified | unclassified | unclassified | unclassified | unclassified |
| Otu007025 | 1 | 1 | 1 | 0.001 | Bacteria | Cyanobacteria | Synechococcophycideae | Synechococcales | Synechococcaceae | Synechococcus | unclassified |
| Otu007048 | 1 | 0.6667 | 0.816 | 0.007 | Bacteria | unclassified | unclassified | unclassified | unclassified | unclassified | unclassified |
| Otu007225 | 0.9113 | 0.6667 | 0.779 | 0.026 | Archaea | Euryarchaeota | Thermoplasmata | E2 | Marine_group_II | unclassified | unclassified |
| Otu007369 | 0.6904 | 1 | 0.831 | 0.037 | Bacteria | Proteobacteria | Gammaproteobacteria | Legionellales | Endoecteinascidiaceae | unclassified | unclassified |
| Otu007445 | 0.9629 | 1 | 0.981 | 0.001 | Bacteria | Cyanobacteria | Synechococcophycideae | Synechococcales | Synechococcaceae | unclassified | unclassified |
| Otu007477 | 0.8544 | 1 | 0.924 | 0.005 | Bacteria | Bacteroidetes | Flavobacteriia | Flavobacteriales | Flavobacteriaceae | unclassified | unclassified |
| Otu007489 | 0.9121 | 1 | 0.955 | 0.001 | Bacteria | Bacteroidetes | Flavobacteriia | Flavobacteriales | Flavobacteriaceae | unclassified | unclassified |
| Otu007565 | 1 | 1 | 1 | 0.001 | Bacteria | Proteobacteria | Alphaproteobacteria | Rickettsiales | Pelagibacteraceae | unclassified | unclassified |
| Otu007594 | 0.95 | 1 | 0.975 | 0.001 | Bacteria | Proteobacteria | Alphaproteobacteria | Rhodobacterales | Rhodobacteraceae | unclassified | unclassified |
| Otu007717 | 0.8479 | 1 | 0.921 | 0.01 | Bacteria | Proteobacteria | Gammaproteobacteria | unclassified | unclassified | unclassified | unclassified |
| Otu007781 | 1 | 0.6667 | 0.816 | 0.007 | Bacteria | Proteobacteria | Gammaproteobacteria | unclassified | unclassified | unclassified | unclassified |
| Otu007801 | 0.9227 | 0.6667 | 0.784 | 0.021 | Bacteria | Proteobacteria | Gammaproteobacteria | Oceanospirillales | Halomonadaceae | Candidatus_Portiera | unclassified |
| Otu007826 | 0.9011 | 1 | 0.949 | 0.003 | Bacteria | unclassified | unclassified | unclassified | unclassified | unclassified | unclassified |
| Otu007907 | 1 | 1 | 1 | 0.001 | Bacteria | unclassified | unclassified | unclassified | unclassified | unclassified | unclassified |
| Otu008000 | 0.9388 | 0.6667 | 0.791 | 0.015 | Bacteria | Proteobacteria | Alphaproteobacteria | Rickettsiales | Pelagibacteraceae | unclassified | unclassified |
| Otu008010 | 0.8912 | 1 | 0.944 | 0.001 | Bacteria | Actinobacteria | Actinobacteria | Actinomycetales | unclassified | unclassified | unclassified |
| Otu008058 | 1 | 0.6667 | 0.816 | 0.007 | Bacteria | Cyanobacteria | Synechococcophycideae | Synechococcales | Synechococcaceae | unclassified | unclassified |
| Otu008063 | 1 | 0.6667 | 0.816 | 0.007 | Bacteria | Cyanobacteria | Synechococcophycideae | Synechococcales | Synechococcaceae | Synechococcus | unclassified |
| Otu008064 | 1 | 0.6667 | 0.816 | 0.007 | Bacteria | Cyanobacteria | Synechococcophycideae | Synechococcales | Synechococcaceae | unclassified | unclassified |
| Otu008074 | 1 | 0.6667 | 0.816 | 0.007 | Bacteria | Bacteroidetes | Flavobacteriia | Flavobacteriales | unclassified | unclassified | unclassified |
| Otu008078 | 0.9923 | 1 | 0.996 | 0.001 | Bacteria | Bacteroidetes | Flavobacteriia | Flavobacteriales | unclassified | unclassified | unclassified |
| Otu008079 | 0.9708 | 1 | 0.985 | 0.001 | Bacteria | Bacteroidetes | Flavobacteriia | Flavobacteriales | Cryomorphaceae | unclassified | unclassified |
| Otu008093 | 0.9779 | 1 | 0.989 | 0.001 | Bacteria | Bacteroidetes | Flavobacteriia | Flavobacteriales | Cryomorphaceae | unclassified | unclassified |
| Otu008120 | 1 | 1 | 1 | 0.001 | Bacteria | Bacteroidetes | Flavobacteriia | Flavobacteriales | unclassified | unclassified | unclassified |
| Otu008256 | 0.5817 | 1 | 0.763 | 0.039 | Bacteria | Proteobacteria | Alphaproteobacteria | Rhodospirillales | Rhodospirillaceae | unclassified | unclassified |
| Otu008277 | 0.7446 | 1 | 0.863 | 0.016 | Bacteria | Proteobacteria | Gammaproteobacteria | Alteromonadales | HTCC2188 | HTCC | unclassified |
| Otu008280 | 1 | 0.6667 | 0.816 | 0.01 | Bacteria | Proteobacteria | Gammaproteobacteria | Oceanospirillales | unclassified | unclassified | unclassified |
| Otu008281 | 0.9768 | 1 | 0.988 | 0.001 | Bacteria | Proteobacteria | Gammaproteobacteria | unclassified | unclassified | unclassified | unclassified |
| Otu008315 | 0.9226 | 1 | 0.961 | 0.002 | Bacteria | Bacteroidetes | unclassified | unclassified | unclassified | unclassified | unclassified |
| Otu008403 | 1 | 0.6667 | 0.816 | 0.007 | Bacteria | Proteobacteria | Alphaproteobacteria | unclassified | unclassified | unclassified | unclassified |
| Otu008436 | 0.95 | 0.6667 | 0.796 | 0.016 | Bacteria | unclassified | unclassified | unclassified | unclassified | unclassified | unclassified |
| Otu008459 | 1 | 0.6667 | 0.816 | 0.009 | Bacteria | Proteobacteria | Gammaproteobacteria | unclassified | unclassified | unclassified | unclassified |
| Otu008562 | 0.9651 | 1 | 0.982 | 0.001 | Bacteria | Proteobacteria | Alphaproteobacteria | Sphingomonadales | Erythrobacteraceae | Erythrobacter | unclassified |
| Otu008599 | 1 | 1 | 1 | 0.001 | Bacteria | Proteobacteria | Gammaproteobacteria | unclassified | unclassified | unclassified | unclassified |
| Otu008776 | 1 | 1 | 1 | 0.001 | Bacteria | Proteobacteria | Gammaproteobacteria | unclassified | unclassified | unclassified | unclassified |
| Otu008791 | 1 | 1 | 1 | 0.001 | Bacteria | Proteobacteria | Gammaproteobacteria | Alteromonadales | Alteromonadaceae | HTCC2207 | unclassified |
| Otu008794 | 1 | 1 | 1 | 0.001 | Bacteria | Proteobacteria | Gammaproteobacteria | Alteromonadales | Alteromonadaceae | HTCC2207 | unclassified |
| Otu008795 | 0.9429 | 0.6667 | 0.793 | 0.017 | Bacteria | Proteobacteria | Gammaproteobacteria | Alteromonadales | Alteromonadaceae | HTCC2207 | unclassified |
| Otu008836 | 0.9399 | 0.6667 | 0.792 | 0.016 | Bacteria | Proteobacteria | Gammaproteobacteria | unclassified | unclassified | unclassified | unclassified |
| Otu008854 | 1 | 0.6667 | 0.816 | 0.007 | Bacteria | Proteobacteria | Gammaproteobacteria | HTCC2188 | HTCC2089 | unclassified | unclassified |
| Otu008867 | 0.9162 | 0.6667 | 0.782 | 0.024 | Bacteria | Proteobacteria | Gammaproteobacteria | HTCC2188 | HTCC2089 | unclassified | unclassified |
| Otu009009 | 1 | 1 | 1 | 0.001 | Bacteria | Proteobacteria | Alphaproteobacteria | Rhodobacterales | Rhodobacteraceae | unclassified | unclassified |
| Otu009015 | 0.8887 | 1 | 0.943 | 0.001 | Bacteria | Proteobacteria | Alphaproteobacteria | Rhodobacterales | Rhodobacteraceae | unclassified | unclassified |
| Otu009022 | 1 | 0.6667 | 0.816 | 0.01 | Bacteria | Proteobacteria | Alphaproteobacteria | Rhodobacterales | Rhodobacteraceae | unclassified | unclassified |
| Otu009215 | 0.954 | 1 | 0.977 | 0.001 | Bacteria | Proteobacteria | Deltaproteobacteria | Myxococcales | OM27 | unclassified | unclassified |
| Otu009268 | 0.9675 | 1 | 0.984 | 0.001 | Bacteria | Proteobacteria | Gammaproteobacteria | Alteromonadales | unclassified | unclassified | unclassified |
| Otu009313 | 0.9396 | 0.6667 | 0.791 | 0.016 | Bacteria | Bacteroidetes | Flavobacteriia | Flavobacteriales | Flavobacteriaceae | unclassified | unclassified |
| Otu009316 | 0.9286 | 1 | 0.964 | 0.002 | Bacteria | Bacteroidetes | Flavobacteriia | Flavobacteriales | Flavobacteriaceae | unclassified | unclassified |
| Otu009319 | 0.9358 | 1 | 0.967 | 0.001 | Bacteria | Proteobacteria | Betaproteobacteria | unclassified | unclassified | unclassified | unclassified |
| Otu009321 | 0.8837 | 0.6667 | 0.768 | 0.013 | Bacteria | Proteobacteria | Betaproteobacteria | MWH-UniP1 | unclassified | unclassified | unclassified |
| Otu009441 | 0.9763 | 1 | 0.988 | 0.001 | Bacteria | Bacteroidetes | Saprospirae | Saprospirales | Saprospiraceae | unclassified | unclassified |
| Otu009463 | 1 | 1 | 1 | 0.001 | Bacteria | Bacteroidetes | Flavobacteriia | Flavobacteriales | Cryomorphaceae | unclassified | unclassified |
| Otu009470 | 0.961 | 1 | 0.98 | 0.001 | Bacteria | Bacteroidetes | Flavobacteriia | Flavobacteriales | Cryomorphaceae | Fluviicola | unclassified |
| Otu009473 | 1 | 1 | 1 | 0.001 | Bacteria | Bacteroidetes | Flavobacteriia | Flavobacteriales | unclassified | unclassified | unclassified |
| Otu009480 | 0.9016 | 0.6667 | 0.775 | 0.025 | Bacteria | Proteobacteria | Alphaproteobacteria | unclassified | unclassified | unclassified | unclassified |
| Otu009602 | 1 | 1 | 1 | 0.001 | Bacteria | Proteobacteria | Gammaproteobacteria | Thiotrichales | Piscirickettsiaceae | unclassified | unclassified |
| Otu009694 | 0.8969 | 0.6667 | 0.773 | 0.025 | Bacteria | Proteobacteria | Gammaproteobacteria | unclassified | unclassified | unclassified | unclassified |
| Otu009730 | 0.8856 | 0.6667 | 0.768 | 0.027 | Bacteria | Cyanobacteria | Synechococcophycideae | Synechococcales | Synechococcaceae | Synechococcus | unclassified |
| Otu009732 | 0.9658 | 1 | 0.983 | 0.001 | Bacteria | Cyanobacteria | Synechococcophycideae | Synechococcales | Synechococcaceae | unclassified | unclassified |
| Otu009733 | 0.9209 | 0.6667 | 0.784 | 0.025 | Bacteria | Cyanobacteria | Synechococcophycideae | Synechococcales | Synechococcaceae | unclassified | unclassified |
| Otu009737 | 1 | 1 | 1 | 0.001 | Bacteria | Proteobacteria | Gammaproteobacteria | Oceanospirillales | Halomonadaceae | Candidatus_Portiera | unclassified |
| Otu009754 | 1 | 1 | 1 | 0.001 | Bacteria | Proteobacteria | unclassified | unclassified | unclassified | unclassified | unclassified |
| Otu010098 | 0.8712 | 1 | 0.933 | 0.003 | Bacteria | Proteobacteria | Alphaproteobacteria | Rickettsiales | Pelagibacteraceae | unclassified | unclassified |

| | | | | | | | | | | | |
|---|---|---|---|---|---|---|---|---|---|---|---|
| Otu010132 | 0.901 | 1 | 0.949 | 0.001 | Bacteria | Verrucomicrobia | Spartobacteria | Chthoniobacterales | Chthoniobacteraceae | Candidatus_Xiphinemato | unclassified |
| Otu010139 | 0.9492 | 1 | 0.974 | 0.001 | Bacteria | Proteobacteria | Gammaproteobacteria | Alteromonadales | OM60 | unclassified | unclassified |
| Otu010158 | 1 | 0.6667 | 0.816 | 0.01 | Bacteria | Bacteroidetes | Flavobacteriia | Flavobacteriales | unclassified | unclassified | unclassified |
| Otu010181 | 1 | 1 | 1 | 0.001 | Bacteria | Cyanobacteria | Synechococcophycideae | Synechococcales | Synechococcaceae | unclassified | unclassified |
| Otu010205 | 1 | 0.6667 | 0.816 | 0.009 | Bacteria | Bacteroidetes | Flavobacteriia | Flavobacteriales | NS9 | unclassified | unclassified |
| Otu010207 | 1 | 1 | 1 | 0.001 | Bacteria | Bacteroidetes | Flavobacteriia | Flavobacteriales | Cryomorphaceae | Crocinitomix | unclassified |
| Otu010492 | 0.9654 | 1 | 0.983 | 0.002 | Bacteria | Proteobacteria | Gammaproteobacteria | Vibrionales | Vibrionaceae | Aliivibrio | fischeri |
| Otu010748 | 0.8745 | 0.6667 | 0.764 | 0.024 | Bacteria | Proteobacteria | Alphaproteobacteria | Rhizobiales | unclassified | unclassified | unclassified |
| Otu010756 | 0.6964 | 1 | 0.835 | 0.032 | Bacteria | Proteobacteria | Alphaproteobacteria | Kiloniellales | Kiloniellaceae | unclassified | unclassified |
| Otu010969 | 0.8553 | 1 | 0.925 | 0.003 | Bacteria | Proteobacteria | Betaproteobacteria | unclassified | unclassified | unclassified | unclassified |
| Otu011148 | 1 | 1 | 1 | 0.001 | Bacteria | Proteobacteria | Gammaproteobacteria | Alteromonadales | OM60 | Congregibacter | unclassified |
| Otu011156 | 1 | 1 | 1 | 0.001 | Bacteria | Proteobacteria | Gammaproteobacteria | unclassified | unclassified | unclassified | unclassified |
| Otu011168 | 1 | 0.6667 | 0.816 | 0.01 | Bacteria | Proteobacteria | Gammaproteobacteria | Alteromonadales | unclassified | unclassified | unclassified |
| Otu011353 | 0.888 | 0.6667 | 0.769 | 0.023 | Bacteria | Cyanobacteria | Synechococcophycideae | Synechococcales | Synechococcaceae | unclassified | unclassified |
| Otu011386 | 0.9451 | 0.6667 | 0.794 | 0.007 | Bacteria | Cyanobacteria | Synechococcophycideae | Synechococcales | Synechococcaceae | unclassified | unclassified |
| Otu011389 | 0.8191 | 0.6667 | 0.739 | 0.037 | Bacteria | Cyanobacteria | Synechococcophycideae | Synechococcales | Synechococcaceae | unclassified | unclassified |
| Otu011392 | 0.916 | 0.6667 | 0.781 | 0.026 | Bacteria | Bacteroidetes | Flavobacteriia | Flavobacteriales | Flavobacteriaceae | unclassified | unclassified |
| Otu011395 | 0.8788 | 1 | 0.937 | 0.002 | Bacteria | Bacteroidetes | Flavobacteriia | Flavobacteriales | Flavobacteriaceae | Flavobacterium | gelidilacus |
| Otu011405 | 0.9173 | 0.6667 | 0.782 | 0.019 | Bacteria | Bacteroidetes | Flavobacteriia | Flavobacteriales | unclassified | unclassified | unclassified |
| Otu011418 | 0.8568 | 0.6667 | 0.756 | 0.023 | Bacteria | Bacteroidetes | Flavobacteriia | Flavobacteriales | Flavobacteriaceae | unclassified | unclassified |
| Otu011457 | 0.9327 | 1 | 0.966 | 0.002 | Bacteria | Bacteroidetes | Flavobacteriia | Flavobacteriales | Cryomorphaceae | unclassified | unclassified |
| Otu011464 | 0.8485 | 1 | 0.921 | 0.002 | Bacteria | Bacteroidetes | Flavobacteriia | Flavobacteriales | unclassified | unclassified | unclassified |
| Otu011539 | 1 | 0.6667 | 0.816 | 0.007 | Bacteria | Cyanobacteria | Synechococcophycideae | Synechococcales | Synechococcaceae | unclassified | unclassified |
| Otu011568 | 0.9578 | 0.6667 | 0.799 | 0.009 | Bacteria | Bacteroidetes | Flavobacteriia | Flavobacteriales | Flavobacteriaceae | unclassified | unclassified |
| Otu011569 | 1 | 0.6667 | 0.816 | 0.007 | Bacteria | Bacteroidetes | Flavobacteriia | Flavobacteriales | Cryomorphaceae | unclassified | unclassified |
| Otu011642 | 1 | 0.6667 | 0.816 | 0.007 | Bacteria | Proteobacteria | Gammaproteobacteria | unclassified | unclassified | unclassified | unclassified |
| Otu011666 | 0.9491 | 0.6667 | 0.795 | 0.01 | Bacteria | Proteobacteria | Gammaproteobacteria | unclassified | unclassified | unclassified | unclassified |
| Otu011993 | 0.839 | 1 | 0.916 | 0.007 | Bacteria | Proteobacteria | Gammaproteobacteria | Alteromonadales | OM60 | unclassified | unclassified |
| Otu012122 | 0.8572 | 0.6667 | 0.756 | 0.036 | Bacteria | Proteobacteria | Gammaproteobacteria | Vibrionales | Vibrionaceae | Enterovibrio | unclassified |
| Otu012358 | 0.8216 | 1 | 0.906 | 0.006 | Bacteria | unclassified | unclassified | unclassified | unclassified | unclassified | unclassified |
| Otu012359 | 1 | 1 | 1 | 0.001 | Bacteria | unclassified | unclassified | unclassified | unclassified | unclassified | unclassified |
| Otu012532 | 0.9135 | 1 | 0.956 | 0.003 | Bacteria | Proteobacteria | Alphaproteobacteria | Rhodobacterales | Rhodobacteraceae | Pseudoruegeria | unclassified |
| Otu012535 | 0.9917 | 1 | 0.996 | 0.001 | Bacteria | Proteobacteria | Alphaproteobacteria | Rhodobacterales | Rhodobacteraceae | unclassified | unclassified |
| Otu012624 | 1 | 1 | 1 | 0.001 | Bacteria | Proteobacteria | Alphaproteobacteria | Kiloniellales | unclassified | unclassified | unclassified |
| Otu012919 | 0.7959 | 1 | 0.892 | 0.001 | Bacteria | Proteobacteria | Gammaproteobacteria | Oceanospirillales | Halomonadaceae | Candidatus_Portiera | unclassified |
| Otu013076 | 0.9226 | 1 | 0.96 | 0.001 | Bacteria | Verrucomicrobia | Verrucomicrobiae | Verrucomicrobiales | Verrucomicrobiaceae | Verrucomicrobium | unclassified |
| Otu013369 | 1 | 0.6667 | 0.816 | 0.01 | Bacteria | SAR406 | AB16 | Arctic96B-7 | A714017 | ZA3312c | unclassified |
| Otu013534 | 1 | 0.6667 | 0.816 | 0.007 | Bacteria | Proteobacteria | Gammaproteobacteria | unclassified | unclassified | unclassified | unclassified |
| Otu013564 | 0.9258 | 0.6667 | 0.786 | 0.021 | Bacteria | Proteobacteria | Gammaproteobacteria | unclassified | unclassified | unclassified | unclassified |
| Otu013687 | 0.8252 | 0.6667 | 0.742 | 0.049 | Bacteria | Proteobacteria | Gammaproteobacteria | unclassified | unclassified | unclassified | unclassified |
| Otu013721 | 1 | 1 | 1 | 0.001 | Bacteria | Proteobacteria | Gammaproteobacteria | Alteromonadales | Alteromonadaceae | ZD0117 | unclassified |
| Otu013723 | 1 | 1 | 1 | 0.001 | Bacteria | Proteobacteria | Gammaproteobacteria | Alteromonadales | unclassified | unclassified | unclassified |
| Otu013725 | 1 | 1 | 1 | 0.001 | Bacteria | Proteobacteria | Gammaproteobacteria | Alteromonadales | Alteromonadaceae | Glaciecola | unclassified |
| Otu014356 | 0.7778 | 1 | 0.882 | 0.025 | Bacteria | Proteobacteria | Gammaproteobacteria | unclassified | unclassified | unclassified | unclassified |
| Otu014747 | 0.9734 | 1 | 0.987 | 0.001 | Bacteria | Bacteroidetes | Sphingobacteriia | Sphingobacteriales | NS11-12 | unclassified | unclassified |
| Otu014763 | 0.9567 | 0.6667 | 0.799 | 0.01 | Bacteria | Proteobacteria | unclassified | unclassified | unclassified | unclassified | unclassified |
| Otu014808 | 1 | 1 | 1 | 0.001 | Bacteria | Bacteroidetes | Flavobacteriia | Flavobacteriales | unclassified | unclassified | unclassified |
| Otu014815 | 1 | 0.6667 | 0.816 | 0.01 | Bacteria | Bacteroidetes | Flavobacteriia | Flavobacteriales | unclassified | unclassified | unclassified |
| Otu014837 | 0.9457 | 1 | 0.972 | 0.001 | Bacteria | Bacteroidetes | Flavobacteriia | Flavobacteriales | unclassified | unclassified | unclassified |
| Otu014838 | 1 | 0.6667 | 0.816 | 0.007 | Bacteria | Bacteroidetes | Flavobacteriia | Flavobacteriales | NS9 | unclassified | unclassified |
| Otu014846 | 1 | 1 | 1 | 0.001 | Bacteria | Bacteroidetes | Flavobacteriia | Flavobacteriales | Cryomorphaceae | unclassified | unclassified |
| Otu014848 | 0.9321 | 1 | 0.965 | 0.001 | Bacteria | Bacteroidetes | Flavobacteriia | Flavobacteriales | Flavobacteriaceae | unclassified | unclassified |
| Otu014866 | 1 | 0.6667 | 0.816 | 0.007 | Bacteria | Bacteroidetes | Flavobacteriia | Flavobacteriales | unclassified | unclassified | unclassified |
| Otu014867 | 0.5856 | 1 | 0.765 | 0.027 | Bacteria | Bacteroidetes | Flavobacteriia | Flavobacteriales | unclassified | unclassified | unclassified |
| Otu014870 | 0.9055 | 1 | 0.952 | 0.001 | Bacteria | Bacteroidetes | Flavobacteriia | Flavobacteriales | Flavobacteriaceae | unclassified | unclassified |
| Otu014874 | 0.9869 | 1 | 0.993 | 0.001 | Bacteria | Bacteroidetes | Flavobacteriia | Flavobacteriales | unclassified | unclassified | unclassified |
| Otu014878 | 0.9675 | 1 | 0.984 | 0.001 | Bacteria | Bacteroidetes | Flavobacteriia | Flavobacteriales | NS9 | unclassified | unclassified |
| Otu014879 | 0.9461 | 0.6667 | 0.794 | 0.014 | Bacteria | Bacteroidetes | Flavobacteriia | Flavobacteriales | Flavobacteriaceae | unclassified | unclassified |
| Otu014932 | 1 | 1 | 1 | 0.001 | Bacteria | Cyanobacteria | Synechococcophycideae | Synechococcales | Synechococcaceae | unclassified | unclassified |
| Otu014935 | 1 | 1 | 1 | 0.001 | Bacteria | Cyanobacteria | Synechococcophycideae | Synechococcales | Synechococcaceae | unclassified | unclassified |
| Otu015046 | 0.6267 | 1 | 0.792 | 0.045 | Bacteria | Actinobacteria | Thermoleophilia | unclassified | unclassified | unclassified | unclassified |
| Otu015089 | 0.7992 | 1 | 0.894 | 0.007 | Bacteria | Bacteroidetes | Cytophagia | Cytophagales | Flammeovirgaceae | JTB248 | unclassified |
| Otu015164 | 1 | 1 | 1 | 0.001 | Bacteria | Proteobacteria | Betaproteobacteria | Burkholderiales | Comamonadaceae | Azohydromonas | unclassified |
| Otu015245 | 1 | 0.6667 | 0.816 | 0.01 | Bacteria | Proteobacteria | Alphaproteobacteria | Rhodobacterales | Rhodobacteraceae | unclassified | unclassified |
| Otu015258 | 0.8528 | 1 | 0.923 | 0.005 | Bacteria | Proteobacteria | Alphaproteobacteria | Rickettsiales | Pelagibacteraceae | unclassified | unclassified |
| Otu015302 | 0.9373 | 0.6667 | 0.79 | 0.025 | Bacteria | Proteobacteria | Alphaproteobacteria | Rickettsiales | Pelagibacteraceae | unclassified | unclassified |
| Otu015323 | 0.9348 | 1 | 0.967 | 0.001 | Bacteria | Proteobacteria | Alphaproteobacteria | Rhodobacterales | Rhodobacteraceae | Loktanella | vestfoldensis |
| Otu015324 | 1 | 1 | 1 | 0.001 | Bacteria | Proteobacteria | Alphaproteobacteria | Rhodobacterales | Rhodobacteraceae | Octadecabacter | unclassified |
| Otu015382 | 0.8612 | 1 | 0.928 | 0.003 | Bacteria | Verrucomicrobia | Opitutae | Opitutales | Opitutaceae | Opitutus | unclassified |
| Otu015444 | 0.9206 | 0.6667 | 0.783 | 0.025 | Bacteria | Proteobacteria | Gammaproteobacteria | Oceanospirillales | Halomonadaceae | Candidatus_Portiera | unclassified |
| Otu015447 | 1 | 0.6667 | 0.816 | 0.009 | Bacteria | Proteobacteria | Gammaproteobacteria | unclassified | unclassified | unclassified | unclassified |
| Otu015464 | 0.9766 | 1 | 0.988 | 0.001 | Bacteria | Proteobacteria | Gammaproteobacteria | Oceanospirillales | Halomonadaceae | Candidatus_Portiera | unclassified |
| Otu015494 | 1 | 1 | 1 | 0.001 | Bacteria | Proteobacteria | unclassified | unclassified | unclassified | unclassified | unclassified |
| Otu015496 | 1 | 0.6667 | 0.816 | 0.007 | Bacteria | Proteobacteria | Deltaproteobacteria | PB19 | unclassified | unclassified | unclassified |
| Otu015630 | 0.9637 | 1 | 0.982 | 0.001 | Bacteria | Proteobacteria | Alphaproteobacteria | Rhodobacterales | Rhodobacteraceae | unclassified | unclassified |
| Otu015669 | 1 | 1 | 1 | 0.001 | Bacteria | Proteobacteria | unclassified | unclassified | unclassified | unclassified | unclassified |
| Otu015673 | 1 | 1 | 1 | 0.001 | Bacteria | unclassified | unclassified | unclassified | unclassified | unclassified | unclassified |
| Otu016066 | 0.9633 | 1 | 0.982 | 0.001 | Bacteria | Proteobacteria | unclassified | unclassified | unclassified | unclassified | unclassified |
| Otu016071 | 0.9636 | 1 | 0.982 | 0.001 | Bacteria | Verrucomicrobia | Opitutae | Puniceicoccales | Puniceicoccaceae | Coraliomargarita | unclassified |
| Otu016208 | 1 | 1 | 1 | 0.001 | Bacteria | Proteobacteria | unclassified | unclassified | unclassified | unclassified | unclassified |
| Otu016308 | 0.9677 | 1 | 0.984 | 0.001 | Bacteria | Proteobacteria | Betaproteobacteria | Burkholderiales | Comamonadaceae | unclassified | unclassified |
| Otu016323 | 1 | 1 | 1 | 0.001 | Bacteria | Proteobacteria | Betaproteobacteria | unclassified | unclassified | unclassified | unclassified |
| Otu016397 | 0.7036 | 1 | 0.839 | 0.034 | Bacteria | Planctomycetes | Planctomycetia | Pirellulales | Pirellulaceae | unclassified | unclassified |
| Otu016576 | 0.9082 | 1 | 0.953 | 0.001 | Bacteria | Proteobacteria | Gammaproteobacteria | unclassified | unclassified | unclassified | unclassified |
| Otu016592 | 0.9622 | 1 | 0.981 | 0.001 | Bacteria | Proteobacteria | Gammaproteobacteria | Oceanospirillales | unclassified | unclassified | unclassified |
| Otu016632 | 0.9402 | 0.6667 | 0.792 | 0.02 | Bacteria | Proteobacteria | Gammaproteobacteria | Oceanospirillales | Halomonadaceae | Candidatus_Portiera | unclassified |
| Otu016636 | 0.9639 | 1 | 0.982 | 0.001 | Bacteria | Proteobacteria | Gammaproteobacteria | Oceanospirillales | Oceanospirillaceae | Marinomonas | unclassified |
| Otu016641 | 0.7356 | 1 | 0.858 | 0.019 | Bacteria | Proteobacteria | Gammaproteobacteria | Alteromonadales | HTCC2188 | HTCC | unclassified |
| Otu016644 | 1 | 0.6667 | 0.816 | 0.007 | Bacteria | Proteobacteria | Gammaproteobacteria | Oceanospirillales | Halomonadaceae | Candidatus_Portiera | unclassified |
| Otu016995 | 0.8371 | 0.6667 | 0.747 | 0.048 | Bacteria | Proteobacteria | Gammaproteobacteria | unclassified | unclassified | unclassified | unclassified |
| Otu017044 | 1 | 1 | 1 | 0.001 | Bacteria | Proteobacteria | Gammaproteobacteria | unclassified | unclassified | unclassified | unclassified |
| Otu017139 | 0.9662 | 0.6667 | 0.803 | 0.007 | Bacteria | Proteobacteria | Gammaproteobacteria | unclassified | unclassified | unclassified | unclassified |
| Otu017239 | 1 | 0.6667 | 0.816 | 0.01 | Bacteria | Proteobacteria | Alphaproteobacteria | Rickettsiales | Pelagibacteraceae | unclassified | unclassified |
| Otu017341 | 0.9575 | 0.6667 | 0.799 | 0.009 | Bacteria | Proteobacteria | Gammaproteobacteria | unclassified | unclassified | unclassified | unclassified |
| Otu017465 | 1 | 1 | 1 | 0.001 | Bacteria | Bacteroidetes | Flavobacteriia | Flavobacteriales | Flavobacteriaceae | unclassified | unclassified |
| Otu017466 | 1 | 0.6667 | 0.816 | 0.01 | Bacteria | Bacteroidetes | Flavobacteriia | Flavobacteriales | unclassified | unclassified | unclassified |
| Otu017494 | 0.9499 | 1 | 0.975 | 0.001 | Bacteria | Bacteroidetes | Flavobacteriia | Flavobacteriales | Cryomorphaceae | Fluviicola | unclassified |
| Otu017495 | 1 | 1 | 1 | 0.001 | Bacteria | Bacteroidetes | Flavobacteriia | Flavobacteriales | Flavobacteriaceae | unclassified | unclassified |
| Otu017567 | 1 | 1 | 1 | 0.001 | Bacteria | Bacteroidetes | Flavobacteriia | Flavobacteriales | Flavobacteriaceae | Formosa | unclassified |
| Otu017596 | 0.8729 | 0.6667 | 0.763 | 0.012 | Bacteria | Bacteroidetes | Flavobacteriia | Flavobacteriales | NS9 | unclassified | unclassified |

| | | | | | | | | | | | |
|---|---|---|---|---|---|---|---|---|---|---|---|
| Otu017597 | 0.9672 | 1 | 0.983 | 0.001 | Bacteria | Bacteroidetes | Flavobacteriia | Flavobacteriales | Cryomorphaceae | unclassified | unclassified |
| Otu017607 | 0.9637 | 1 | 0.982 | 0.001 | Bacteria | Bacteroidetes | Flavobacteriia | Flavobacteriales | Cryomorphaceae | unclassified | unclassified |
| Otu017610 | 0.8593 | 0.6667 | 0.757 | 0.016 | Bacteria | Bacteroidetes | Flavobacteriia | Flavobacteriales | unclassified | unclassified | unclassified |
| Otu017623 | 0.9733 | 1 | 0.987 | 0.001 | Bacteria | Bacteroidetes | Flavobacteriia | Flavobacteriales | Flavobacteriaceae | unclassified | unclassified |
| Otu017625 | 0.9571 | 1 | 0.978 | 0.001 | Bacteria | Bacteroidetes | Flavobacteriia | Flavobacteriales | Flavobacteriaceae | unclassified | unclassified |
| Otu017629 | 1 | 0.6667 | 0.816 | 0.009 | Bacteria | Bacteroidetes | Flavobacteriia | Flavobacteriales | unclassified | unclassified | unclassified |
| Otu017920 | 0.8495 | 0.6667 | 0.753 | 0.033 | Bacteria | Proteobacteria | Gammaproteobacteria | Alteromonadales | unclassified | unclassified | unclassified |
| Otu017931 | 0.9366 | 1 | 0.968 | 0.001 | Bacteria | Proteobacteria | Gammaproteobacteria | unclassified | unclassified | unclassified | unclassified |
| Otu017997 | 0.9085 | 1 | 0.953 | 0.001 | Bacteria | Proteobacteria | Gammaproteobacteria | Thiotrichales | Thiotrichaceae | CF-26 | unclassified |
| Otu018017 | 0.907 | 1 | 0.952 | 0.002 | Bacteria | Cyanobacteria | Synechococcophycideae | Synechococcales | Synechococcaceae | unclassified | unclassified |
| Otu018019 | 1 | 0.6667 | 0.816 | 0.009 | Bacteria | Cyanobacteria | Synechococcophycideae | Synechococcales | Synechococcaceae | Synechococcus | unclassified |
| Otu018026 | 1 | 1 | 1 | 0.001 | Bacteria | Cyanobacteria | Synechococcophycideae | Synechococcales | Synechococcaceae | unclassified | unclassified |
| Otu018033 | 1 | 0.6667 | 0.816 | 0.009 | Bacteria | Cyanobacteria | Synechococcophycideae | Synechococcales | Synechococcaceae | unclassified | unclassified |
| Otu018048 | 1 | 0.6667 | 0.816 | 0.007 | Bacteria | Proteobacteria | Gammaproteobacteria | Pseudomonadales | Pseudomonadaceae | Pseudomonas | stutzeri |
| Otu018107 | 1 | 1 | 1 | 0.001 | Bacteria | Proteobacteria | Gammaproteobacteria | unclassified | unclassified | unclassified | unclassified |
| Otu018108 | 0.97 | 1 | 0.985 | 0.001 | Bacteria | Proteobacteria | unclassified | unclassified | unclassified | unclassified | unclassified |
| Otu018141 | 1 | 0.6667 | 0.816 | 0.007 | Bacteria | Cyanobacteria | Synechococcophycideae | Synechococcales | Synechococcaceae | Synechococcus | unclassified |
| Otu018146 | 1 | 0.6667 | 0.816 | 0.007 | Bacteria | Cyanobacteria | Synechococcophycideae | Synechococcales | Synechococcaceae | unclassified | unclassified |
| Otu018230 | 0.7842 | 1 | 0.886 | 0.012 | Bacteria | Proteobacteria | Alphaproteobacteria | Rhodospirillales | Rhodospirillaceae | unclassified | unclassified |
| Otu018234 | 0.9375 | 0.6667 | 0.791 | 0.026 | Bacteria | Proteobacteria | unclassified | unclassified | unclassified | unclassified | unclassified |
| Otu018626 | 1 | 1 | 1 | 0.001 | Bacteria | Proteobacteria | Alphaproteobacteria | Rhodobacterales | Rhodobacteraceae | Anaerospora | hongkongensis |
| Otu018640 | 0.8942 | 1 | 0.946 | 0.001 | Bacteria | Proteobacteria | Alphaproteobacteria | Rhodobacterales | Rhodobacteraceae | unclassified | unclassified |
| Otu018675 | 1 | 0.6667 | 0.816 | 0.009 | Bacteria | Proteobacteria | Alphaproteobacteria | Rhodobacterales | Rhodobacteraceae | Phaeobacter | unclassified |
| Otu018677 | 1 | 0.6667 | 0.816 | 0.009 | Bacteria | Proteobacteria | Alphaproteobacteria | Rhodobacterales | Rhodobacteraceae | unclassified | unclassified |
| Otu018699 | 1 | 1 | 1 | 0.001 | Bacteria | Proteobacteria | Alphaproteobacteria | Rhodobacterales | Rhodobacteraceae | unclassified | unclassified |
| Otu018704 | 0.9654 | 1 | 0.983 | 0.001 | Bacteria | Proteobacteria | Alphaproteobacteria | Rhodobacterales | Rhodobacteraceae | unclassified | unclassified |
| Otu018711 | 1 | 0.6667 | 0.816 | 0.009 | Bacteria | Proteobacteria | Alphaproteobacteria | Rhodobacterales | Rhodobacteraceae | unclassified | unclassified |
| Otu018774 | 0.9025 | 0.6667 | 0.776 | 0.028 | Bacteria | Proteobacteria | Deltaproteobacteria | Desulfobacterales | Desulfobulbaceae | unclassified | unclassified |
| Otu018938 | 0.9442 | 1 | 0.972 | 0.001 | Bacteria | Actinobacteria | Actinobacteria | Actinomycetales | Microbacteriaceae | Candidatus_Aquiluna | rubra |
| Otu019038 | 1 | 0.6667 | 0.816 | 0.007 | Bacteria | Proteobacteria | Gammaproteobacteria | Vibrionales | Pseudoalteromonadaceae | Pseudoalteromonas | unclassified |
| Otu019061 | 1 | 0.6667 | 0.816 | 0.01 | Bacteria | Proteobacteria | Gammaproteobacteria | Vibrionales | Pseudoalteromonadaceae | Pseudoalteromonas | porphyrae |
| Otu019067 | 1 | 0.6667 | 0.816 | 0.01 | Bacteria | Proteobacteria | Gammaproteobacteria | Alteromonadales | Moritellaceae | Moritella | unclassified |
| Otu019269 | 0.9667 | 1 | 0.983 | 0.001 | Bacteria | unclassified | unclassified | unclassified | unclassified | unclassified | unclassified |
| Otu019416 | 0.8093 | 1 | 0.9 | 0.009 | Bacteria | Proteobacteria | Gammaproteobacteria | Vibrionales | Vibrionaceae | Photobacterium | angustum |
| Otu019417 | 0.8504 | 1 | 0.922 | 0.005 | Bacteria | Proteobacteria | Gammaproteobacteria | Vibrionales | Vibrionaceae | Enterovibrio | unclassified |
| Otu019617 | 0.9611 | 1 | 0.98 | 0.001 | Bacteria | Proteobacteria | Gammaproteobacteria | Alteromonadales | HTCC2188 | HTCC | unclassified |
| Otu019624 | 0.9726 | 1 | 0.986 | 0.001 | Bacteria | Proteobacteria | Gammaproteobacteria | Oceanospirillales | Halomonadaceae | Candidatus_Portiera | unclassified |
| Otu019656 | 1 | 1 | 1 | 0.001 | Bacteria | Proteobacteria | Gammaproteobacteria | unclassified | unclassified | unclassified | unclassified |
| Otu019668 | 0.9003 | 1 | 0.949 | 0.001 | Bacteria | Proteobacteria | Gammaproteobacteria | Oceanospirillales | unclassified | unclassified | unclassified |
| Otu019671 | 0.9272 | 0.6667 | 0.786 | 0.021 | Bacteria | Proteobacteria | Gammaproteobacteria | Oceanospirillales | Halomonadaceae | Candidatus_Portiera | unclassified |
| Otu019689 | 1 | 0.6667 | 0.816 | 0.007 | Bacteria | Proteobacteria | Gammaproteobacteria | Oceanospirillales | unclassified | unclassified | unclassified |
| Otu019693 | 1 | 0.6667 | 0.816 | 0.007 | Bacteria | Proteobacteria | Gammaproteobacteria | Oceanospirillales | unclassified | unclassified | unclassified |
| Otu019701 | 0.9493 | 1 | 0.974 | 0.001 | Bacteria | Proteobacteria | Gammaproteobacteria | Alteromonadales | HTCC2188 | HTCC | unclassified |
| Otu019911 | 0.9764 | 1 | 0.988 | 0.001 | Bacteria | Verrucomicrobia | Opitutae | Puniceicoccales | Puniceicoccaceae | Coraliomargarita | unclassified |
| Otu019918 | 1 | 1 | 1 | 0.001 | Bacteria | Verrucomicrobia | Opitutae | Puniceicoccales | Puniceicoccaceae | MB11C04 | unclassified |
| Otu019935 | 0.9391 | 0.6667 | 0.791 | 0.022 | Bacteria | Proteobacteria | Gammaproteobacteria | Oceanospirillales | unclassified | unclassified | unclassified |
| Otu019939 | 1 | 1 | 1 | 0.001 | Bacteria | Proteobacteria | Gammaproteobacteria | Oceanospirillales | Halomonadaceae | Candidatus_Portiera | unclassified |
| Otu020052 | 1 | 0.6667 | 0.816 | 0.009 | Bacteria | Proteobacteria | Alphaproteobacteria | Rhodobacterales | Rhodobacteraceae | unclassified | unclassified |
| Otu020055 | 0.8458 | 1 | 0.92 | 0.009 | Bacteria | Proteobacteria | Alphaproteobacteria | Rhodobacterales | Rhodobacteraceae | unclassified | unclassified |
| Otu020057 | 0.9157 | 1 | 0.957 | 0.001 | Bacteria | Proteobacteria | Alphaproteobacteria | Rhodobacterales | Rhodobacteraceae | unclassified | unclassified |
| Otu020096 | 1 | 1 | 1 | 0.001 | Bacteria | Proteobacteria | Alphaproteobacteria | Rhodobacterales | Rhodobacteraceae | unclassified | unclassified |
| Otu020182 | 0.9396 | 0.6667 | 0.791 | 0.016 | Bacteria | Cyanobacteria | unclassified | unclassified | unclassified | unclassified | unclassified |
| Otu020296 | 0.9603 | 1 | 0.98 | 0.001 | Bacteria | Proteobacteria | Alphaproteobacteria | Rhodobacterales | Rhodobacteraceae | Octadecabacter | unclassified |
| Otu020297 | 0.9617 | 1 | 0.981 | 0.001 | Bacteria | Proteobacteria | Alphaproteobacteria | Rhodobacterales | Rhodobacteraceae | unclassified | unclassified |
| Otu020300 | 0.9719 | 1 | 0.986 | 0.001 | Bacteria | Proteobacteria | Alphaproteobacteria | Rhodobacterales | Rhodobacteraceae | unclassified | unclassified |
| Otu020311 | 1 | 0.6667 | 0.816 | 0.01 | Bacteria | Proteobacteria | Alphaproteobacteria | Rhodobacterales | Rhodobacteraceae | unclassified | unclassified |
| Otu020340 | 0.9502 | 1 | 0.975 | 0.001 | Bacteria | Proteobacteria | Alphaproteobacteria | Rhodobacterales | Rhodobacteraceae | unclassified | unclassified |
| Otu021371 | 0.8174 | 0.6667 | 0.738 | 0.044 | Bacteria | Proteobacteria | Gammaproteobacteria | unclassified | unclassified | unclassified | unclassified |
| Otu021373 | 0.9783 | 1 | 0.989 | 0.001 | Bacteria | Proteobacteria | Gammaproteobacteria | Vibrionales | Vibrionaceae | unclassified | unclassified |
| Otu021380 | 1 | 1 | 1 | 0.001 | Bacteria | Proteobacteria | Alphaproteobacteria | unclassified | unclassified | unclassified | unclassified |
| Otu021381 | 0.8463 | 1 | 0.92 | 0.007 | Bacteria | Proteobacteria | Alphaproteobacteria | unclassified | unclassified | unclassified | unclassified |
| Otu021396 | 0.7997 | 0.6667 | 0.73 | 0.043 | Bacteria | Proteobacteria | Gammaproteobacteria | Vibrionales | Vibrionaceae | unclassified | unclassified |
| Otu021420 | 0.7599 | 1 | 0.872 | 0.012 | Bacteria | Actinobacteria | Acidimicrobiia | Acidimicrobiales | Iamiaceae | Iamia | unclassified |
| Otu021560 | 0.6877 | 1 | 0.829 | 0.044 | Bacteria | Proteobacteria | Alphaproteobacteria | Rhodospirillales | Rhodospirillaceae | unclassified | unclassified |
| Otu021953 | 0.9832 | 1 | 0.992 | 0.001 | Bacteria | Bacteroidetes | Flavobacteriia | Flavobacteriales | Flavobacteriaceae | unclassified | unclassified |
| Otu021967 | 1 | 0.6667 | 0.816 | 0.007 | Bacteria | Bacteroidetes | Flavobacteriia | Flavobacteriales | Cryomorphaceae | unclassified | unclassified |
| Otu021978 | 1 | 1 | 1 | 0.001 | Bacteria | Bacteroidetes | Flavobacteriia | Flavobacteriales | unclassified | unclassified | unclassified |
| Otu022018 | 0.9563 | 1 | 0.978 | 0.001 | Bacteria | Bacteroidetes | Flavobacteriia | Flavobacteriales | unclassified | unclassified | unclassified |
| Otu022060 | 0.7296 | 1 | 0.854 | 0.022 | Bacteria | Bacteroidetes | Flavobacteriia | Flavobacteriales | Flavobacteriaceae | unclassified | unclassified |
| Otu022065 | 0.9399 | 0.6667 | 0.792 | 0.016 | Bacteria | Bacteroidetes | Flavobacteriia | Flavobacteriales | unclassified | unclassified | unclassified |
| Otu022084 | 0.9791 | 1 | 0.989 | 0.001 | Bacteria | Bacteroidetes | Flavobacteriia | Flavobacteriales | Cryomorphaceae | unclassified | unclassified |
| Otu022087 | 0.9505 | 1 | 0.975 | 0.001 | Bacteria | Bacteroidetes | Flavobacteriia | Flavobacteriales | Flavobacteriaceae | unclassified | unclassified |
| Otu022093 | 0.9582 | 1 | 0.979 | 0.001 | Bacteria | Bacteroidetes | Flavobacteriia | Flavobacteriales | Cryomorphaceae | Fluviicola | unclassified |
| Otu022109 | 1 | 1 | 1 | 0.001 | Bacteria | Bacteroidetes | Flavobacteriia | Flavobacteriales | Cryomorphaceae | Fluviicola | unclassified |
| Otu022114 | 0.8223 | 0.6667 | 0.74 | 0.033 | Bacteria | Bacteroidetes | Flavobacteriia | Flavobacteriales | Flavobacteriaceae | Bizionia | unclassified |
| Otu022127 | 0.9704 | 1 | 0.985 | 0.001 | Bacteria | Bacteroidetes | Flavobacteriia | Flavobacteriales | Flavobacteriaceae | unclassified | unclassified |
| Otu022319 | 1 | 0.6667 | 0.816 | 0.01 | Bacteria | Verrucomicrobia | Verrucomicrobiae | Verrucomicrobiales | Verrucomicrobiaceae | Verrucomicrobium | unclassified |
| Otu022338 | 0.9401 | 1 | 0.97 | 0.001 | Bacteria | Actinobacteria | Actinobacteria | Actinomycetales | Microbacteriaceae | Candidatus_Aquiluna | rubra |
| Otu022342 | 0.7498 | 1 | 0.866 | 0.02 | Bacteria | Actinobacteria | Actinobacteria | Actinomycetales | unclassified | unclassified | unclassified |
| Otu022348 | 0.9595 | 1 | 0.98 | 0.001 | Bacteria | Verrucomicrobia | unclassified | unclassified | unclassified | unclassified | unclassified |
| Otu022349 | 0.8866 | 1 | 0.942 | 0.001 | Bacteria | unclassified | unclassified | unclassified | unclassified | unclassified | unclassified |
| Otu022480 | 0.9043 | 0.6667 | 0.776 | 0.026 | Bacteria | Proteobacteria | Gammaproteobacteria | Vibrionales | Pseudoalteromonadaceae | Pseudoalteromonas | porphyrae |
| Otu022489 | 0.916 | 0.6667 | 0.781 | 0.022 | Bacteria | Proteobacteria | Gammaproteobacteria | Alteromonadales | Alteromonadaceae | Umboniibacter | unclassified |
| Otu022494 | 1 | 0.6667 | 0.816 | 0.007 | Bacteria | Proteobacteria | Gammaproteobacteria | Vibrionales | Pseudoalteromonadaceae | Pseudoalteromonas | unclassified |
| Otu022517 | 0.8991 | 0.6667 | 0.774 | 0.029 | Bacteria | Proteobacteria | Gammaproteobacteria | Alteromonadales | Colwelliaceae | unclassified | unclassified |
| Otu022526 | 1 | 1 | 1 | 0.001 | Bacteria | Proteobacteria | Gammaproteobacteria | unclassified | unclassified | unclassified | unclassified |
| Otu022585 | 1 | 0.6667 | 0.816 | 0.007 | Bacteria | Cyanobacteria | Synechococcophycideae | Synechococcales | Synechococcaceae | unclassified | unclassified |
| Otu022595 | 1 | 1 | 1 | 0.001 | Bacteria | Proteobacteria | Gammaproteobacteria | Pseudomonadales | Moraxellaceae | Psychrobacter | pacificensis |
| Otu022599 | 0.5964 | 1 | 0.772 | 0.049 | Bacteria | Cyanobacteria | Synechococcophycideae | Synechococcales | Synechococcaceae | Synechococcus | unclassified |
| Otu022605 | 1 | 0.6667 | 0.816 | 0.009 | Bacteria | Cyanobacteria | Synechococcophycideae | Synechococcales | Synechococcaceae | Synechococcus | unclassified |
| Otu022613 | 1 | 0.6667 | 0.816 | 0.01 | Bacteria | Cyanobacteria | Synechococcophycideae | Synechococcales | Synechococcaceae | unclassified | unclassified |
| Otu022619 | 1 | 1 | 1 | 0.001 | Bacteria | Cyanobacteria | Synechococcophycideae | Synechococcales | Synechococcaceae | unclassified | unclassified |
| Otu022647 | 0.9388 | 0.6667 | 0.791 | 0.015 | Bacteria | Proteobacteria | Alphaproteobacteria | Rickettsiales | Pelagibacteraceae | unclassified | unclassified |
| Otu022750 | 0.9254 | 1 | 0.962 | 0.002 | Bacteria | Proteobacteria | Gammaproteobacteria | Alteromonadales | unclassified | unclassified | unclassified |
| Otu023321 | 0.8969 | 0.6667 | 0.773 | 0.025 | Bacteria | Verrucomicrobia | unclassified | unclassified | unclassified | unclassified | unclassified |
| Otu023545 | 0.9042 | 1 | 0.951 | 0.003 | Bacteria | unclassified | unclassified | unclassified | unclassified | unclassified | unclassified |
| Otu023816 | 0.8092 | 1 | 0.9 | 0.008 | Bacteria | Proteobacteria | Alphaproteobacteria | Rhodospirillales | Rhodospirillaceae | unclassified | unclassified |
| Otu023947 | 0.8653 | 0.6667 | 0.759 | 0.034 | Bacteria | Proteobacteria | Gammaproteobacteria | unclassified | unclassified | unclassified | unclassified |
| Otu024397 | 0.9717 | 1 | 0.986 | 0.001 | Bacteria | Proteobacteria | Gammaproteobacteria | Alteromonadales | unclassified | unclassified | unclassified |
| Otu024414 | 0.7852 | 1 | 0.886 | 0.011 | Bacteria | Proteobacteria | Alphaproteobacteria | unclassified | unclassified | unclassified | unclassified |

| | | | | | | | | | | | |
|---|---|---|---|---|---|---|---|---|---|---|---|
| Otu024619 | 1 | 0.6667 | 0.816 | 0.009 | Bacteria | Proteobacteria | Gammaproteobacteria | Alteromonadales | unclassified | unclassified | unclassified |
| Otu024770 | 1 | 0.6667 | 0.816 | 0.009 | Bacteria | unclassified | unclassified | unclassified | unclassified | unclassified | unclassified |
| Otu025016 | 0.8281 | 1 | 0.91 | 0.004 | Bacteria | Proteobacteria | Betaproteobacteria | Methylophilales | Methylophilaceae | unclassified | unclassified |
| Otu025359 | 0.9715 | 1 | 0.986 | 0.001 | Bacteria | Proteobacteria | Gammaproteobacteria | unclassified | unclassified | unclassified | unclassified |
| Otu025373 | 1 | 1 | 1 | 0.001 | Bacteria | Proteobacteria | Gammaproteobacteria | unclassified | unclassified | unclassified | unclassified |
| Otu025672 | 0.9752 | 1 | 0.988 | 0.001 | Bacteria | Proteobacteria | Betaproteobacteria | Burkholderiales | unclassified | unclassified | unclassified |
| Otu025697 | 0.9668 | 0.6667 | 0.803 | 0.01 | Bacteria | Proteobacteria | Betaproteobacteria | Burkholderiales | unclassified | unclassified | unclassified |
| Otu025726 | 1 | 0.6667 | 0.816 | 0.009 | Bacteria | Bacteroidetes | Flavobacteriia | Flavobacteriales | unclassified | unclassified | unclassified |
| Otu025740 | 1 | 0.6667 | 0.816 | 0.01 | Bacteria | Bacteroidetes | Flavobacteriia | Flavobacteriales | unclassified | unclassified | unclassified |
| Otu025746 | 1 | 0.6667 | 0.816 | 0.01 | Bacteria | Bacteroidetes | Flavobacteriia | Flavobacteriales | Flavobacteriaceae | unclassified | unclassified |
| Otu025817 | 1 | 0.6667 | 0.816 | 0.007 | Bacteria | Bacteroidetes | Flavobacteriia | Flavobacteriales | Flavobacteriaceae | unclassified | unclassified |
| Otu025839 | 0.9638 | 1 | 0.982 | 0.001 | Bacteria | Bacteroidetes | Flavobacteriia | Flavobacteriales | unclassified | unclassified | unclassified |
| Otu025855 | 1 | 1 | 1 | 0.001 | Bacteria | Bacteroidetes | Flavobacteriia | Flavobacteriales | unclassified | unclassified | unclassified |
| Otu025860 | 0.9492 | 0.6667 | 0.795 | 0.014 | Bacteria | Bacteroidetes | Flavobacteriia | Flavobacteriales | unclassified | unclassified | unclassified |
| Otu025873 | 1 | 0.6667 | 0.816 | 0.007 | Bacteria | Bacteroidetes | Flavobacteriia | Flavobacteriales | Cryomorphaceae | unclassified | unclassified |
| Otu026199 | 1 | 0.6667 | 0.816 | 0.007 | Bacteria | Actinobacteria | Actinobacteria | Actinomycetales | Microbacteriaceae | unclassified | unclassified |
| Otu026456 | 1 | 0.6667 | 0.816 | 0.009 | Bacteria | Bacteroidetes | Flavobacteriia | Flavobacteriales | Flavobacteriaceae | unclassified | unclassified |
| Otu026457 | 0.9561 | 0.6667 | 0.798 | 0.007 | Bacteria | Bacteroidetes | Flavobacteriia | Flavobacteriales | Flavobacteriaceae | unclassified | unclassified |
| Otu026477 | 1 | 0.6667 | 0.816 | 0.009 | Bacteria | Bacteroidetes | Flavobacteriia | Flavobacteriales | Flavobacteriaceae | unclassified | unclassified |
| Otu026478 | 0.9318 | 0.6667 | 0.788 | 0.025 | Bacteria | Bacteroidetes | Flavobacteriia | Flavobacteriales | Flavobacteriaceae | unclassified | unclassified |
| Otu026498 | 1 | 1 | 1 | 0.001 | Bacteria | Bacteroidetes | Flavobacteriia | Flavobacteriales | unclassified | unclassified | unclassified |
| Otu026500 | 1 | 1 | 1 | 0.001 | Bacteria | Bacteroidetes | Flavobacteriia | Flavobacteriales | Flavobacteriaceae | Formosa | unclassified |
| Otu026503 | 0.9827 | 1 | 0.991 | 0.001 | Bacteria | Bacteroidetes | unclassified | unclassified | unclassified | unclassified | unclassified |
| Otu026518 | 0.8553 | 1 | 0.925 | 0.007 | Bacteria | Verrucomicrobia | Verrucomicrobiae | Verrucomicrobiales | Verrucomicrobiaceae | unclassified | unclassified |
| Otu026958 | 1 | 0.6667 | 0.816 | 0.007 | Bacteria | Proteobacteria | Gammaproteobacteria | unclassified | unclassified | unclassified | unclassified |
| Otu027005 | 0.8437 | 1 | 0.919 | 0.007 | Bacteria | Proteobacteria | Gammaproteobacteria | Alteromonadales | unclassified | unclassified | unclassified |
| Otu027036 | 1 | 1 | 1 | 0.001 | Bacteria | Proteobacteria | Gammaproteobacteria | Vibrionales | Pseudoalteromonadaceae | Pseudoalteromonas | unclassified |
| Otu027314 | 0.8969 | 0.6667 | 0.773 | 0.025 | Bacteria | Fusobacteria | Fusobacteriia | Fusobacteriales | Fusobacteriaceae | Propionigenium | unclassified |
| Otu028461 | 1 | 0.6667 | 0.816 | 0.007 | Bacteria | Proteobacteria | Gammaproteobacteria | Oceanospirillales | unclassified | unclassified | unclassified |
| Otu028548 | 0.9534 | 1 | 0.976 | 0.001 | Bacteria | Proteobacteria | Betaproteobacteria | Burkholderiales | Comamonadaceae | unclassified | unclassified |
| Otu028595 | 1 | 0.6667 | 0.816 | 0.01 | Bacteria | Proteobacteria | Gammaproteobacteria | Alteromonadales | OM60 | unclassified | unclassified |
| Otu028600 | 0.9413 | 0.6667 | 0.792 | 0.015 | Bacteria | Proteobacteria | Gammaproteobacteria | Alteromonadales | OM60 | unclassified | unclassified |
| Otu028614 | 1 | 1 | 1 | 0.001 | Bacteria | Proteobacteria | Gammaproteobacteria | Alteromonadales | Alteromonadaceae | unclassified | unclassified |
| Otu028618 | 1 | 0.6667 | 0.816 | 0.01 | Bacteria | Proteobacteria | Gammaproteobacteria | unclassified | unclassified | unclassified | unclassified |
| Otu028642 | 0.938 | 0.6667 | 0.791 | 0.019 | Bacteria | Proteobacteria | Gammaproteobacteria | unclassified | unclassified | unclassified | unclassified |
| Otu028869 | 1 | 0.6667 | 0.816 | 0.007 | Bacteria | Cyanobacteria | unclassified | unclassified | unclassified | unclassified | unclassified |
| Otu029180 | 0.881 | 1 | 0.939 | 0.001 | Bacteria | Verrucomicrobia | Opitutae | Puniceicoccales | Puniceicoccaceae | MB11C04 | unclassified |
| Otu029443 | 0.9682 | 0.6667 | 0.803 | 0.007 | Bacteria | Verrucomicrobia | Opitutae | Puniceicoccales | Puniceicoccaceae | Coraliomargarita | unclassified |
| Otu029663 | 0.801 | 1 | 0.895 | 0.006 | Bacteria | Bacteroidetes | Flavobacteriia | Flavobacteriales | Flavobacteriaceae | unclassified | unclassified |
| Otu029671 | 1 | 1 | 1 | 0.001 | Bacteria | Bacteroidetes | Flavobacteriia | Flavobacteriales | Flavobacteriaceae | unclassified | unclassified |
| Otu029707 | 1 | 1 | 1 | 0.001 | Bacteria | Bacteroidetes | Flavobacteriia | Flavobacteriales | Cryomorphaceae | unclassified | unclassified |
| Otu029712 | 1 | 0.6667 | 0.816 | 0.01 | Bacteria | Bacteroidetes | Flavobacteriia | Flavobacteriales | Flavobacteriaceae | Formosa | unclassified |
| Otu029720 | 0.9235 | 1 | 0.961 | 0.002 | Bacteria | Bacteroidetes | Flavobacteriia | Flavobacteriales | Flavobacteriaceae | unclassified | unclassified |
| Otu029722 | 0.8672 | 1 | 0.931 | 0.001 | Bacteria | Bacteroidetes | Flavobacteriia | Flavobacteriales | unclassified | unclassified | unclassified |
| Otu029746 | 1 | 0.6667 | 0.816 | 0.01 | Bacteria | Bacteroidetes | Flavobacteriia | Flavobacteriales | Flavobacteriaceae | unclassified | unclassified |
| Otu030608 | 0.9774 | 1 | 0.989 | 0.001 | Bacteria | Proteobacteria | Gammaproteobacteria | unclassified | unclassified | unclassified | unclassified |
| Otu031090 | 0.9433 | 1 | 0.971 | 0.001 | Bacteria | Bacteroidetes | unclassified | unclassified | unclassified | unclassified | unclassified |
| Otu031667 | 1 | 1 | 1 | 0.001 | Bacteria | Proteobacteria | Gammaproteobacteria | unclassified | unclassified | unclassified | unclassified |
| Otu032118 | 1 | 0.6667 | 0.816 | 0.01 | Bacteria | Proteobacteria | Gammaproteobacteria | Alteromonadales | unclassified | unclassified | unclassified |
| Otu032293 | 0.9575 | 0.6667 | 0.799 | 0.009 | Bacteria | Proteobacteria | Gammaproteobacteria | Alteromonadales | unclassified | unclassified | unclassified |
| Otu032304 | 0.9291 | 0.6667 | 0.787 | 0.026 | Bacteria | Cyanobacteria | Synechococcophycideae | Synechococcales | Synechococcaceae | unclassified | unclassified |
| Otu032307 | 0.9184 | 0.6667 | 0.782 | 0.023 | Bacteria | Cyanobacteria | Synechococcophycideae | Synechococcales | Synechococcaceae | unclassified | unclassified |
| Otu032327 | 1 | 0.6667 | 0.816 | 0.01 | Bacteria | Cyanobacteria | Synechococcophycideae | Synechococcales | Synechococcaceae | unclassified | unclassified |
| Otu032345 | 0.8676 | 0.6667 | 0.761 | 0.023 | Bacteria | Cyanobacteria | Synechococcophycideae | Synechococcales | Synechococcaceae | Prochlorococcus | unclassified |
| Otu032423 | 0.9005 | 0.6667 | 0.775 | 0.03 | Bacteria | Proteobacteria | unclassified | unclassified | unclassified | unclassified | unclassified |
| Otu032474 | 0.9392 | 0.6667 | 0.791 | 0.023 | Bacteria | Cyanobacteria | Synechococcophycideae | Synechococcales | Synechococcaceae | unclassified | unclassified |
| Otu032488 | 1 | 1 | 1 | 0.001 | Bacteria | Cyanobacteria | Synechococcophycideae | Synechococcales | Synechococcaceae | Synechococcus | unclassified |
| Otu032492 | 0.7064 | 1 | 0.84 | 0.017 | Bacteria | Cyanobacteria | Synechococcophycideae | Synechococcales | Synechococcaceae | unclassified | unclassified |
| Otu032498 | 1 | 1 | 1 | 0.001 | Bacteria | Cyanobacteria | Synechococcophycideae | Synechococcales | Synechococcaceae | unclassified | unclassified |
| Otu032509 | 1 | 0.6667 | 0.816 | 0.009 | Bacteria | Cyanobacteria | Synechococcophycideae | Synechococcales | Synechococcaceae | unclassified | unclassified |
| Otu033181 | 0.9536 | 1 | 0.977 | 0.001 | Bacteria | Proteobacteria | Betaproteobacteria | Methylophilales | Methylophilaceae | Methylotenera | mobilis |
| Otu033351 | 1 | 0.6667 | 0.816 | 0.009 | Bacteria | Proteobacteria | Gammaproteobacteria | unclassified | unclassified | unclassified | unclassified |
| Otu033352 | 0.9059 | 0.6667 | 0.777 | 0.025 | Bacteria | Proteobacteria | Gammaproteobacteria | Oceanospirillales | Halomonadaceae | Candidatus_Portiera | unclassified |
| Otu033384 | 1 | 1 | 1 | 0.001 | Bacteria | Proteobacteria | Betaproteobacteria | Rhodocyclales | Rhodocyclaceae | Azovibrio | unclassified |
| Otu033495 | 0.9527 | 1 | 0.976 | 0.001 | Bacteria | Proteobacteria | Alphaproteobacteria | Rhodospirillales | Rhodospirillaceae | unclassified | unclassified |
| Otu033794 | 1 | 1 | 1 | 0.001 | Bacteria | Proteobacteria | Alphaproteobacteria | Rhodobacterales | Rhodobacteraceae | unclassified | unclassified |
| Otu033819 | 0.9661 | 1 | 0.983 | 0.001 | Bacteria | Proteobacteria | Alphaproteobacteria | Rhodobacterales | Rhodobacteraceae | unclassified | unclassified |
| Otu033821 | 0.8649 | 0.6667 | 0.759 | 0.03 | Bacteria | Proteobacteria | Alphaproteobacteria | Rhodobacterales | Rhodobacteraceae | unclassified | unclassified |
| Otu033859 | 0.9448 | 0.6667 | 0.794 | 0.014 | Bacteria | Proteobacteria | Alphaproteobacteria | unclassified | unclassified | unclassified | unclassified |
| Otu033873 | 0.7853 | 1 | 0.886 | 0.003 | Bacteria | Proteobacteria | Alphaproteobacteria | unclassified | unclassified | unclassified | unclassified |
| Otu033886 | 0.9715 | 1 | 0.986 | 0.001 | Bacteria | Proteobacteria | Alphaproteobacteria | Rhodospirillales | Rhodospirillaceae | unclassified | unclassified |
| Otu034000 | 1 | 1 | 1 | 0.001 | Bacteria | Proteobacteria | Alphaproteobacteria | Rhodobacterales | Rhodobacteraceae | Octadecabacter | unclassified |
| Otu034008 | 0.9468 | 1 | 0.973 | 0.001 | Bacteria | Proteobacteria | Alphaproteobacteria | Rhodobacterales | Rhodobacteraceae | unclassified | unclassified |
| Otu034020 | 0.9797 | 1 | 0.99 | 0.001 | Bacteria | Proteobacteria | Alphaproteobacteria | Rhodobacterales | Rhodobacteraceae | unclassified | unclassified |
| Otu034095 | 0.9269 | 1 | 0.963 | 0.001 | Bacteria | Proteobacteria | Alphaproteobacteria | unclassified | unclassified | unclassified | unclassified |
| Otu034117 | 0.9404 | 1 | 0.97 | 0.001 | Bacteria | unclassified | unclassified | unclassified | unclassified | unclassified | unclassified |
| Otu034156 | 1 | 0.6667 | 0.816 | 0.009 | Bacteria | Proteobacteria | Alphaproteobacteria | Sphingomonadales | Erythrobacteraceae | Erythrobacter | unclassified |
| Otu034246 | 1 | 0.6667 | 0.816 | 0.007 | Bacteria | Proteobacteria | unclassified | unclassified | unclassified | unclassified | unclassified |
| Otu034272 | 0.661 | 1 | 0.813 | 0.037 | Bacteria | Proteobacteria | Alphaproteobacteria | unclassified | unclassified | unclassified | unclassified |
| Otu034749 | 0.9636 | 1 | 0.982 | 0.001 | Bacteria | Proteobacteria | Alphaproteobacteria | Rhodobacterales | Rhodobacteraceae | Rhodobacter | unclassified |
| Otu034764 | 0.9437 | 1 | 0.971 | 0.001 | Bacteria | Proteobacteria | Alphaproteobacteria | Rhodobacterales | Rhodobacteraceae | unclassified | unclassified |
| Otu034806 | 1 | 1 | 1 | 0.001 | Bacteria | Proteobacteria | Alphaproteobacteria | Rhodobacterales | Rhodobacteraceae | unclassified | unclassified |
| Otu034824 | 1 | 0.6667 | 0.816 | 0.009 | Bacteria | Proteobacteria | Alphaproteobacteria | Rhodobacterales | Rhodobacteraceae | unclassified | unclassified |
| Otu035657 | 0.9696 | 1 | 0.985 | 0.001 | Bacteria | Proteobacteria | Gammaproteobacteria | Oceanospirillales | Oceanospirillaceae | Oleispira | unclassified |
| Otu035967 | 0.9508 | 0.6667 | 0.796 | 0.009 | Bacteria | unclassified | unclassified | unclassified | unclassified | unclassified | unclassified |
| Otu036279 | 0.9606 | 0.6667 | 0.8 | 0.009 | Bacteria | Proteobacteria | unclassified | unclassified | unclassified | unclassified | unclassified |
| Otu036596 | 1 | 1 | 1 | 0.001 | Bacteria | Actinobacteria | Acidimicrobiia | Acidimicrobiales | OCS155 | unclassified | unclassified |
| Otu036907 | 1 | 0.6667 | 0.816 | 0.01 | Bacteria | Actinobacteria | Acidimicrobiia | Acidimicrobiales | OCS155 | unclassified | unclassified |
| Otu036939 | 0.9495 | 1 | 0.974 | 0.003 | Bacteria | Actinobacteria | Acidimicrobiia | Acidimicrobiales | OCS155 | unclassified | unclassified |
| Otu036952 | 1 | 1 | 1 | 0.001 | Bacteria | Actinobacteria | Acidimicrobiia | Acidimicrobiales | OCS155 | unclassified | unclassified |
| Otu037244 | 0.9437 | 1 | 0.971 | 0.002 | Bacteria | Proteobacteria | Gammaproteobacteria | Alteromonadales | HTCC2188 | HTCC | unclassified |
| Otu037246 | 0.9666 | 1 | 0.983 | 0.002 | Bacteria | Proteobacteria | Gammaproteobacteria | unclassified | unclassified | unclassified | unclassified |
| Otu037955 | 0.9032 | 1 | 0.95 | 0.001 | Bacteria | Bacteroidetes | Flavobacteriia | Flavobacteriales | Flavobacteriaceae | Bizionia | unclassified |
| Otu037963 | 1 | 1 | 1 | 0.001 | Bacteria | Bacteroidetes | Flavobacteriia | Flavobacteriales | Flavobacteriaceae | unclassified | unclassified |
| Otu037971 | 1 | 0.6667 | 0.816 | 0.007 | Bacteria | Bacteroidetes | Flavobacteriia | Flavobacteriales | Cryomorphaceae | unclassified | unclassified |
| Otu037983 | 0.9679 | 1 | 0.984 | 0.001 | Bacteria | Bacteroidetes | Flavobacteriia | Flavobacteriales | Flavobacteriaceae | unclassified | unclassified |
| Otu038107 | 1 | 0.6667 | 0.816 | 0.01 | Bacteria | Proteobacteria | Gammaproteobacteria | Oceanospirillales | unclassified | unclassified | unclassified |
| Otu038126 | 0.9287 | 1 | 0.964 | 0.002 | Bacteria | Proteobacteria | Gammaproteobacteria | Alteromonadales | unclassified | unclassified | unclassified |
| Otu038179 | 1 | 0.6667 | 0.816 | 0.01 | Bacteria | Proteobacteria | Gammaproteobacteria | Oceanospirillales | Halomonadaceae | Candidatus_Portiera | unclassified |
| Otu038189 | 1 | 0.6667 | 0.816 | 0.007 | Bacteria | Proteobacteria | Gammaproteobacteria | Oceanospirillales | Halomonadaceae | Candidatus_Portiera | unclassified |

| | | | | | | | | | | | |
|---|---|---|---|---|---|---|---|---|---|---|---|
| Otu038242 | 1 | 0.6667 | 0.816 | 0.007 | Bacteria | Proteobacteria | Gammaproteobacteria | Oceanospirillales | unclassified | unclassified | unclassified |
| Otu038291 | 0.93 | 1 | 0.964 | 0.002 | Bacteria | Proteobacteria | Gammaproteobacteria | Oceanospirillales | Halomonadaceae | Candidatus_Portiera | unclassified |
| Otu038322 | 0.9707 | 1 | 0.985 | 0.001 | Bacteria | Proteobacteria | Gammaproteobacteria | unclassified | unclassified | unclassified | unclassified |
| Otu038335 | 0.9684 | 0.6667 | 0.804 | 0.007 | Bacteria | Proteobacteria | Gammaproteobacteria | Alteromonadales | unclassified | unclassified | unclassified |
| Otu038371 | 0.9427 | 1 | 0.971 | 0.001 | Bacteria | Proteobacteria | Gammaproteobacteria | Alteromonadales | HTCC2188 | HTCC | unclassified |
| Otu038483 | 0.9672 | 1 | 0.983 | 0.001 | Bacteria | Proteobacteria | Gammaproteobacteria | Alteromonadales | HTCC2188 | HTCC | unclassified |
| Otu038524 | 1 | 0.6667 | 0.816 | 0.009 | Bacteria | Proteobacteria | Gammaproteobacteria | unclassified | unclassified | unclassified | unclassified |
| Otu038527 | 0.9873 | 1 | 0.994 | 0.001 | Bacteria | Proteobacteria | Gammaproteobacteria | Alteromonadales | Alteromonadaceae | unclassified | unclassified |
| Otu038546 | 0.8124 | 0.6667 | 0.736 | 0.042 | Bacteria | Bacteroidetes | Flavobacteriia | Flavobacteriales | Flavobacteriaceae | unclassified | unclassified |
| Otu038567 | 0.943 | 1 | 0.971 | 0.001 | Bacteria | Bacteroidetes | Flavobacteriia | Flavobacteriales | Flavobacteriaceae | Formosa | unclassified |
| Otu038579 | 1 | 0.6667 | 0.816 | 0.009 | Bacteria | Bacteroidetes | Flavobacteriia | Flavobacteriales | Cryomorphaceae | Fluviicola | unclassified |
| Otu038607 | 0.8475 | 1 | 0.921 | 0.006 | Bacteria | Bacteroidetes | Flavobacteriia | Flavobacteriales | Flavobacteriaceae | unclassified | unclassified |
| Otu038729 | 0.9432 | 1 | 0.971 | 0.001 | Bacteria | Bacteroidetes | Flavobacteriia | Flavobacteriales | Flavobacteriaceae | unclassified | unclassified |
| Otu038764 | 0.9166 | 1 | 0.957 | 0.002 | Bacteria | Bacteroidetes | Flavobacteriia | Flavobacteriales | Flavobacteriaceae | unclassified | unclassified |
| Otu038796 | 1 | 0.6667 | 0.816 | 0.007 | Bacteria | Bacteroidetes | Flavobacteriia | Flavobacteriales | unclassified | unclassified | unclassified |
| Otu038815 | 1 | 1 | 1 | 0.001 | Bacteria | Bacteroidetes | Flavobacteriia | Flavobacteriales | unclassified | unclassified | unclassified |
| Otu038823 | 1 | 0.6667 | 0.816 | 0.01 | Bacteria | Bacteroidetes | Flavobacteriia | Flavobacteriales | Flavobacteriaceae | unclassified | unclassified |
| Otu038842 | 1 | 1 | 1 | 0.001 | Bacteria | Bacteroidetes | Flavobacteriia | Flavobacteriales | Flavobacteriaceae | unclassified | unclassified |
| Otu039360 | 0.9636 | 1 | 0.982 | 0.002 | Bacteria | Bacteroidetes | Flavobacteriia | Flavobacteriales | unclassified | unclassified | unclassified |
| Otu039684 | 1 | 1 | 1 | 0.001 | Bacteria | Bacteroidetes | Flavobacteriia | Flavobacteriales | Flavobacteriaceae | unclassified | unclassified |
| Otu039785 | 0.8182 | 0.6667 | 0.739 | 0.045 | Bacteria | Bacteroidetes | Flavobacteriia | Flavobacteriales | Flavobacteriaceae | unclassified | unclassified |
| Otu039811 | 1 | 0.6667 | 0.816 | 0.01 | Bacteria | Bacteroidetes | Flavobacteriia | Flavobacteriales | NS9 | unclassified | unclassified |
| Otu039954 | 1 | 1 | 1 | 0.001 | Bacteria | Proteobacteria | Gammaproteobacteria | unclassified | unclassified | unclassified | unclassified |
| Otu040277 | 1 | 0.6667 | 0.816 | 0.01 | Bacteria | Proteobacteria | Gammaproteobacteria | Oceanospirillales | Halomonadaceae | Candidatus_Portiera | unclassified |
| Otu040309 | 1 | 0.6667 | 0.816 | 0.009 | Bacteria | Verrucomicrobia | Verrucomicrobiae | Verrucomicrobiales | Verrucomicrobiaceae | unclassified | unclassified |
| Otu040345 | 1 | 0.6667 | 0.816 | 0.01 | Bacteria | Proteobacteria | Gammaproteobacteria | unclassified | unclassified | unclassified | unclassified |
| Otu040427 | 0.9563 | 1 | 0.978 | 0.002 | Bacteria | Proteobacteria | Gammaproteobacteria | unclassified | unclassified | unclassified | unclassified |
| Otu040512 | 1 | 1 | 1 | 0.001 | Bacteria | Proteobacteria | Gammaproteobacteria | Alteromonadales | 211ds20 | unclassified | unclassified |
| Otu040535 | 1 | 1 | 1 | 0.001 | Bacteria | Proteobacteria | Gammaproteobacteria | Oceanospirillales | Halomonadaceae | Candidatus_Portiera | unclassified |
| Otu040544 | 1 | 0.6667 | 0.816 | 0.01 | Bacteria | Proteobacteria | Gammaproteobacteria | unclassified | unclassified | unclassified | unclassified |
| Otu040550 | 1 | 0.6667 | 0.816 | 0.007 | Bacteria | Proteobacteria | Gammaproteobacteria | Oceanospirillales | Halomonadaceae | Candidatus_Portiera | unclassified |
| Otu041027 | 1 | 0.6667 | 0.816 | 0.01 | Bacteria | Cyanobacteria | Synechococcophycideae | Pseudanabaenales | Pseudanabaenaceae | Halomicronema | unclassified |
| Otu041271 | 1 | 0.6667 | 0.816 | 0.007 | Bacteria | Bacteroidetes | Flavobacteriia | Flavobacteriales | Flavobacteriaceae | unclassified | unclassified |
| Otu041599 | 1 | 0.6667 | 0.816 | 0.009 | Bacteria | Bacteroidetes | Flavobacteriia | Flavobacteriales | Flavobacteriaceae | unclassified | unclassified |
| Otu041856 | 1 | 0.6667 | 0.816 | 0.01 | Bacteria | Bacteroidetes | Flavobacteriia | Flavobacteriales | Flavobacteriaceae | unclassified | unclassified |
| Otu041859 | 1 | 1 | 1 | 0.001 | Bacteria | Bacteroidetes | Flavobacteriia | Flavobacteriales | Flavobacteriaceae | Polaribacter | unclassified |
| Otu042265 | 0.919 | 0.6667 | 0.783 | 0.022 | Bacteria | Bacteroidetes | Flavobacteriia | Flavobacteriales | Flavobacteriaceae | unclassified | unclassified |
| Otu042274 | 0.9786 | 1 | 0.989 | 0.001 | Bacteria | Bacteroidetes | Flavobacteriia | Flavobacteriales | unclassified | unclassified | unclassified |
| Otu042320 | 1 | 0.6667 | 0.816 | 0.007 | Bacteria | Bacteroidetes | Flavobacteriia | Flavobacteriales | Flavobacteriaceae | unclassified | unclassified |
| Otu042379 | 0.8716 | 1 | 0.934 | 0.002 | Bacteria | Bacteroidetes | Flavobacteriia | Flavobacteriales | Flavobacteriaceae | Formosa | unclassified |
| Otu042402 | 0.9063 | 0.6667 | 0.777 | 0.021 | Bacteria | Bacteroidetes | Flavobacteriia | Flavobacteriales | Flavobacteriaceae | unclassified | unclassified |
| Otu045988 | 0.806 | 1 | 0.898 | 0.003 | Bacteria | Proteobacteria | Gammaproteobacteria | Thiotrichales | Piscirickettsiaceae | unclassified | unclassified |
| Otu046257 | 1 | 1 | 1 | 0.001 | Bacteria | unclassified | unclassified | unclassified | unclassified | unclassified | unclassified |
| Otu046503 | 0.9559 | 1 | 0.978 | 0.001 | Bacteria | Proteobacteria | Epsilonproteobacteria | Campylobacterales | Campylobacteraceae | Arcobacter | unclassified |
| Otu046525 | 1 | 0.6667 | 0.816 | 0.01 | Bacteria | Proteobacteria | Epsilonproteobacteria | Campylobacterales | Campylobacteraceae | Arcobacter | unclassified |
| Otu046536 | 1 | 0.6667 | 0.816 | 0.007 | Bacteria | Proteobacteria | unclassified | unclassified | unclassified | unclassified | unclassified |
| Otu046539 | 1 | 1 | 1 | 0.001 | Bacteria | Proteobacteria | unclassified | unclassified | unclassified | unclassified | unclassified |
| Otu046547 | 0.9399 | 0.6667 | 0.792 | 0.016 | Bacteria | Proteobacteria | unclassified | unclassified | unclassified | unclassified | unclassified |
| Otu046580 | 0.7851 | 0.6667 | 0.723 | 0.047 | Bacteria | unclassified | unclassified | unclassified | unclassified | unclassified | unclassified |
| Otu046754 | 0.9492 | 0.6667 | 0.795 | 0.014 | Bacteria | Bacteroidetes | Flavobacteriia | Flavobacteriales | unclassified | unclassified | unclassified |
| Otu047014 | 0.951 | 0.6667 | 0.796 | 0.015 | Bacteria | Bacteroidetes | Flavobacteriia | Flavobacteriales | Cryomorphaceae | Fluviicola | unclassified |
| Otu047024 | 0.9761 | 1 | 0.988 | 0.001 | Bacteria | Bacteroidetes | Flavobacteriia | Flavobacteriales | unclassified | unclassified | unclassified |
| Otu047692 | 0.8975 | 1 | 0.947 | 0.001 | Bacteria | Proteobacteria | Betaproteobacteria | unclassified | unclassified | unclassified | unclassified |
| Otu047744 | 1 | 0.6667 | 0.816 | 0.007 | unknown | unclassified | unclassified | unclassified | unclassified | unclassified | unclassified |
| Otu048318 | 0.8913 | 0.6667 | 0.771 | 0.024 | Bacteria | Proteobacteria | Gammaproteobacteria | Vibrionales | Vibrionaceae | unclassified | unclassified |
| Otu048512 | 0.7467 | 1 | 0.864 | 0.014 | Bacteria | Proteobacteria | Betaproteobacteria | Methylophilales | Methylophilaceae | unclassified | unclassified |
| Otu048562 | 0.7616 | 1 | 0.873 | 0.016 | Bacteria | Proteobacteria | Betaproteobacteria | Methylophilales | Methylophilaceae | unclassified | unclassified |
| Otu049550 | 0.9618 | 1 | 0.981 | 0.001 | Bacteria | Proteobacteria | Betaproteobacteria | Burkholderiales | Comamonadaceae | unclassified | unclassified |
| Otu049901 | 1 | 1 | 1 | 0.001 | Bacteria | Proteobacteria | Gammaproteobacteria | Vibrionales | Pseudoalteromonadacea | Pseudoalteromonas | unclassified |
| Otu049903 | 0.9593 | 1 | 0.979 | 0.001 | Bacteria | Proteobacteria | Gammaproteobacteria | unclassified | unclassified | unclassified | unclassified |
| Otu050050 | 1 | 1 | 1 | 0.001 | Bacteria | Proteobacteria | Gammaproteobacteria | Alteromonadales | unclassified | unclassified | unclassified |
| Otu050063 | 0.9144 | 0.6667 | 0.781 | 0.014 | Bacteria | Proteobacteria | Gammaproteobacteria | Alteromonadales | unclassified | unclassified | unclassified |
| Otu050206 | 0.9399 | 0.6667 | 0.792 | 0.016 | Bacteria | Proteobacteria | Gammaproteobacteria | Thiotrichales | Thiotrichaceae | Cocleimonas | unclassified |
| Otu050256 | 1 | 0.6667 | 0.816 | 0.007 | Bacteria | Proteobacteria | Gammaproteobacteria | unclassified | unclassified | unclassified | unclassified |
| Otu050281 | 0.8643 | 0.6667 | 0.759 | 0.03 | Bacteria | Proteobacteria | Gammaproteobacteria | Alteromonadales | unclassified | unclassified | unclassified |
| Otu050289 | 1 | 0.6667 | 0.816 | 0.01 | Bacteria | Proteobacteria | Gammaproteobacteria | unclassified | unclassified | unclassified | unclassified |
| Otu050980 | 0.9056 | 1 | 0.952 | 0.001 | Bacteria | Actinobacteria | Actinobacteria | Actinomycetales | Microbacteriaceae | Candidatus_Rhodoluna | unclassified |
| Otu051027 | 0.9186 | 1 | 0.958 | 0.003 | Bacteria | Actinobacteria | Acidimicrobiia | Acidimicrobiales | OCS155 | unclassified | unclassified |
| Otu051059 | 0.9778 | 1 | 0.989 | 0.001 | Bacteria | Actinobacteria | Acidimicrobiia | Acidimicrobiales | OCS155 | unclassified | unclassified |
| Otu051143 | 1 | 0.6667 | 0.816 | 0.007 | Bacteria | Proteobacteria | Gammaproteobacteria | unclassified | unclassified | unclassified | unclassified |
| Otu051218 | 1 | 1 | 1 | 0.001 | Bacteria | Actinobacteria | Acidimicrobiia | Acidimicrobiales | OCS155 | unclassified | unclassified |
| Otu051219 | 1 | 1 | 1 | 0.001 | Bacteria | Actinobacteria | Acidimicrobiia | Acidimicrobiales | OCS155 | unclassified | unclassified |
| Otu051220 | 0.9185 | 0.6667 | 0.783 | 0.019 | Bacteria | Actinobacteria | Acidimicrobiia | Acidimicrobiales | ZA3409c | unclassified | unclassified |
| Otu051225 | 0.9006 | 0.6667 | 0.775 | 0.022 | Bacteria | Actinobacteria | Acidimicrobiia | Acidimicrobiales | OCS155 | unclassified | unclassified |
| Otu051252 | 1 | 1 | 1 | 0.001 | Bacteria | Actinobacteria | Acidimicrobiia | Acidimicrobiales | OCS155 | unclassified | unclassified |
| Otu051269 | 1 | 1 | 1 | 0.001 | Bacteria | Actinobacteria | Acidimicrobiia | Acidimicrobiales | OCS155 | unclassified | unclassified |
| Otu051716 | 0.9505 | 0.6667 | 0.796 | 0.009 | Bacteria | Proteobacteria | Alphaproteobacteria | Rhizobiales | unclassified | unclassified | unclassified |
| Otu051884 | 0.8998 | 0.6667 | 0.775 | 0.033 | Bacteria | Proteobacteria | Gammaproteobacteria | unclassified | unclassified | unclassified | unclassified |
| Otu051945 | 1 | 0.6667 | 0.816 | 0.009 | Bacteria | Proteobacteria | Gammaproteobacteria | HTCC2188 | HTCC2089 | unclassified | unclassified |
| Otu052124 | 1 | 0.6667 | 0.816 | 0.009 | Bacteria | Proteobacteria | Gammaproteobacteria | HTCC2188 | HTCC2089 | unclassified | unclassified |
| Otu052811 | 0.604 | 1 | 0.777 | 0.047 | Bacteria | Proteobacteria | Gammaproteobacteria | unclassified | unclassified | unclassified | unclassified |
| Otu052967 | 1 | 1 | 1 | 0.001 | Bacteria | Bacteroidetes | Flavobacteriia | Flavobacteriales | Flavobacteriaceae | unclassified | unclassified |
| Otu052993 | 0.9632 | 1 | 0.981 | 0.001 | Bacteria | Bacteroidetes | Flavobacteriia | Flavobacteriales | Cryomorphaceae | unclassified | unclassified |
| Otu052994 | 1 | 0.6667 | 0.816 | 0.007 | Bacteria | Bacteroidetes | Flavobacteriia | Flavobacteriales | unclassified | unclassified | unclassified |
| Otu053011 | 0.9066 | 1 | 0.952 | 0.001 | Bacteria | Bacteroidetes | Flavobacteriia | Flavobacteriales | unclassified | unclassified | unclassified |
| Otu053046 | 1 | 0.6667 | 0.816 | 0.009 | Bacteria | Bacteroidetes | Flavobacteriia | Flavobacteriales | unclassified | unclassified | unclassified |
| Otu053560 | 0.8621 | 0.6667 | 0.758 | 0.046 | Bacteria | Proteobacteria | Gammaproteobacteria | Marinicellales | Marinicellaceae | unclassified | unclassified |
| Otu053653 | 1 | 1 | 1 | 0.001 | Bacteria | Proteobacteria | Gammaproteobacteria | unclassified | unclassified | unclassified | unclassified |
| Otu054286 | 1 | 0.6667 | 0.816 | 0.007 | Bacteria | Proteobacteria | Alphaproteobacteria | Rickettsiales | Pelagibacteraceae | unclassified | unclassified |
| Otu055244 | 0.9441 | 1 | 0.972 | 0.002 | Bacteria | Proteobacteria | Alphaproteobacteria | unclassified | unclassified | unclassified | unclassified |
| Otu055981 | 0.8138 | 1 | 0.902 | 0.008 | Bacteria | Proteobacteria | Alphaproteobacteria | unclassified | unclassified | unclassified | unclassified |
| Otu055992 | 1 | 1 | 1 | 0.001 | Bacteria | Proteobacteria | Alphaproteobacteria | unclassified | unclassified | unclassified | unclassified |
| Otu056517 | 1 | 1 | 1 | 0.001 | Bacteria | Proteobacteria | unclassified | unclassified | unclassified | unclassified | unclassified |
| Otu057696 | 1 | 0.6667 | 0.816 | 0.01 | Bacteria | Proteobacteria | Gammaproteobacteria | Vibrionales | Vibrionaceae | unclassified | unclassified |
| Otu057718 | 1 | 0.6667 | 0.816 | 0.01 | Bacteria | Proteobacteria | Gammaproteobacteria | Vibrionales | Vibrionaceae | unclassified | unclassified |
| Otu058499 | 0.9561 | 0.6667 | 0.798 | 0.007 | Bacteria | Bacteroidetes | Cytophagia | Cytophagales | Flammeovirgaceae | JTB248 | unclassified |
| Otu061848 | 0.9406 | 1 | 0.97 | 0.002 | Bacteria | Proteobacteria | Alphaproteobacteria | Rhodobacterales | Rhodobacteraceae | unclassified | unclassified |
| Otu061901 | 0.9386 | 0.6667 | 0.791 | 0.023 | Bacteria | Proteobacteria | Alphaproteobacteria | Rhodobacterales | Rhodobacteraceae | Octadecabacter | unclassified |
| Otu061976 | 1 | 1 | 1 | 0.001 | Bacteria | Proteobacteria | Alphaproteobacteria | Rhodobacterales | Rhodobacteraceae | unclassified | unclassified |
| Otu062070 | 0.9781 | 1 | 0.989 | 0.001 | Bacteria | Proteobacteria | Alphaproteobacteria | Rhodobacterales | Rhodobacteraceae | unclassified | unclassified |

| | | | | | | | | | | | |
|---|---|---|---|---|---|---|---|---|---|---|---|
| Otu062079 | 0.9676 | 0.6667 | 0.803 | 0.009 | Bacteria | Proteobacteria | Alphaproteobacteria | Rhodobacterales | Rhodobacteraceae | Octadecabacter | unclassified |
| Otu062096 | 1 | 0.6667 | 0.816 | 0.01 | Bacteria | Proteobacteria | Alphaproteobacteria | Rhodobacterales | Rhodobacteraceae | Phaeobacter | unclassified |
| Otu062153 | 0.9399 | 1 | 0.97 | 0.001 | Bacteria | Proteobacteria | Alphaproteobacteria | Rhodobacterales | Rhodobacteraceae | Octadecabacter | unclassified |
| Otu062175 | 0.9613 | 1 | 0.98 | 0.001 | Bacteria | Proteobacteria | Alphaproteobacteria | Rhodobacterales | Rhodobacteraceae | unclassified | unclassified |
| Otu062232 | 0.984 | 1 | 0.992 | 0.001 | Bacteria | Proteobacteria | Alphaproteobacteria | Rhodobacterales | unclassified | unclassified | unclassified |
| Otu062251 | 1 | 0.6667 | 0.816 | 0.01 | Bacteria | Proteobacteria | Alphaproteobacteria | Rhodobacterales | Hyphomonadaceae | Hirschia | baltica |
| Otu063607 | 0.9586 | 1 | 0.979 | 0.001 | Bacteria | Proteobacteria | Alphaproteobacteria | Rhodobacterales | Rhodobacteraceae | Octadecabacter | unclassified |
| Otu063639 | 1 | 1 | 1 | 0.001 | Bacteria | Proteobacteria | Alphaproteobacteria | Rhodobacterales | Rhodobacteraceae | unclassified | unclassified |
| Otu063681 | 1 | 1 | 1 | 0.001 | Bacteria | Proteobacteria | Gammaproteobacteria | unclassified | unclassified | unclassified | unclassified |
| Otu065251 | 0.9031 | 0.6667 | 0.776 | 0.018 | Bacteria | Cyanobacteria | Synechococcophycideae | Synechococcales | Synechococcaceae | unclassified | unclassified |
| Otu065320 | 1 | 1 | 1 | 0.001 | Bacteria | Cyanobacteria | Synechococcophycideae | Synechococcales | Synechococcaceae | unclassified | unclassified |
| Otu065324 | 0.9067 | 1 | 0.952 | 0.001 | Bacteria | Cyanobacteria | Synechococcophycideae | Synechococcales | Synechococcaceae | unclassified | unclassified |
| Otu065333 | 1 | 0.6667 | 0.816 | 0.007 | Bacteria | Cyanobacteria | Synechococcophycideae | Synechococcales | Synechococcaceae | unclassified | unclassified |
| Otu065365 | 0.9737 | 1 | 0.987 | 0.001 | Bacteria | Cyanobacteria | Synechococcophycideae | Synechococcales | Synechococcaceae | Synechococcus | unclassified |
| Otu065370 | 0.9428 | 0.6667 | 0.793 | 0.014 | Bacteria | Cyanobacteria | Synechococcophycideae | Synechococcales | Synechococcaceae | Synechococcus | unclassified |
| Otu065448 | 0.9198 | 0.6667 | 0.783 | 0.011 | Bacteria | Proteobacteria | Gammaproteobacteria | Pseudomonadales | Moraxellaceae | Psychrobacter | pacificensis |
| Otu065466 | 0.7391 | 1 | 0.86 | 0.02 | Bacteria | Cyanobacteria | Synechococcophycideae | Synechococcales | Synechococcaceae | Synechococcus | unclassified |
| Otu065485 | 0.8156 | 1 | 0.903 | 0.008 | Bacteria | Cyanobacteria | Synechococcophycideae | Synechococcales | Synechococcaceae | unclassified | unclassified |
| Otu065493 | 0.8375 | 0.6667 | 0.747 | 0.034 | Bacteria | Cyanobacteria | Synechococcophycideae | Synechococcales | Synechococcaceae | unclassified | unclassified |
| Otu065690 | 0.9494 | 0.6667 | 0.796 | 0.01 | Bacteria | Cyanobacteria | Synechococcophycideae | Synechococcales | Synechococcaceae | unclassified | unclassified |
| Otu065711 | 0.7424 | 1 | 0.862 | 0.012 | Bacteria | Cyanobacteria | Synechococcophycideae | Synechococcales | Synechococcaceae | Synechococcus | unclassified |
| Otu065730 | 0.8287 | 1 | 0.91 | 0.002 | Bacteria | Cyanobacteria | Synechococcophycideae | Synechococcales | Synechococcaceae | unclassified | unclassified |
| Otu065749 | 1 | 0.6667 | 0.816 | 0.007 | Bacteria | Cyanobacteria | Synechococcophycideae | Synechococcales | Synechococcaceae | unclassified | unclassified |
| Otu065754 | 1 | 0.6667 | 0.816 | 0.01 | Bacteria | Cyanobacteria | Synechococcophycideae | Synechococcales | Synechococcaceae | Synechococcus | unclassified |
| Otu068218 | 0.9055 | 0.6667 | 0.777 | 0.011 | Bacteria | Verrucomicrobia | Verrucomicrobiae | Verrucomicrobiales | Verrucomicrobiaceae | unclassified | unclassified |
| Otu072414 | 1 | 0.6667 | 0.816 | 0.01 | Bacteria | Verrucomicrobia | Verrucomicrobiae | Verrucomicrobiales | Verrucomicrobiaceae | Rubritalea | unclassified |
| Otu072462 | 0.8369 | 1 | 0.915 | 0.006 | Bacteria | Cyanobacteria | Synechococcophycideae | Synechococcales | Synechococcaceae | unclassified | unclassified |
| Otu073593 | 0.8769 | 0.6667 | 0.765 | 0.034 | Bacteria | Proteobacteria | Gammaproteobacteria | Alteromonadales | Shewanellaceae | Shewanella | benthica |
| Otu074105 | 0.8987 | 1 | 0.948 | 0.002 | Bacteria | Proteobacteria | Alphaproteobacteria | Rickettsiales | Pelagibacteraceae | unclassified | unclassified |
| Otu074123 | 1 | 0.6667 | 0.816 | 0.01 | Bacteria | Proteobacteria | Alphaproteobacteria | Rickettsiales | Pelagibacteraceae | unclassified | unclassified |
| Otu074254 | 1 | 0.6667 | 0.816 | 0.009 | Bacteria | Proteobacteria | Alphaproteobacteria | Rickettsiales | Pelagibacteraceae | unclassified | unclassified |
| Otu074785 | 0.9398 | 1 | 0.969 | 0.001 | Bacteria | Actinobacteria | Actinobacteria | Actinomycetales | unclassified | unclassified | unclassified |
| Otu075854 | 0.9853 | 1 | 0.993 | 0.001 | Bacteria | Proteobacteria | Alphaproteobacteria | Rhodobacterales | Rhodobacteraceae | unclassified | unclassified |
| Otu075858 | 0.8817 | 1 | 0.939 | 0.001 | Bacteria | Proteobacteria | Alphaproteobacteria | Rhodobacterales | Rhodobacteraceae | Octadecabacter | unclassified |
| Otu075919 | 1 | 0.6667 | 0.816 | 0.009 | Bacteria | Proteobacteria | Alphaproteobacteria | Rhodobacterales | Rhodobacteraceae | Octadecabacter | unclassified |
| Otu076842 | 1 | 1 | 1 | 0.001 | Bacteria | Proteobacteria | Alphaproteobacteria | Rhodobacterales | Rhodobacteraceae | Octadecabacter | unclassified |
| Otu076875 | 0.9526 | 0.6667 | 0.797 | 0.01 | Bacteria | Proteobacteria | Alphaproteobacteria | Rhodobacterales | Rhodobacteraceae | unclassified | unclassified |
| Otu076936 | 0.9759 | 1 | 0.988 | 0.001 | Bacteria | Proteobacteria | Alphaproteobacteria | Rhodobacterales | Rhodobacteraceae | Pseudoruegeria | unclassified |
| Otu077012 | 0.9772 | 1 | 0.989 | 0.001 | Bacteria | Proteobacteria | Alphaproteobacteria | Rhodobacterales | Rhodobacteraceae | Octadecabacter | unclassified |
| Otu077119 | 0.9548 | 1 | 0.977 | 0.001 | Bacteria | Proteobacteria | Alphaproteobacteria | Rhodobacterales | Rhodobacteraceae | Octadecabacter | unclassified |
| Otu077126 | 0.9573 | 1 | 0.978 | 0.001 | Bacteria | Proteobacteria | Alphaproteobacteria | Rhodobacterales | Rhodobacteraceae | Octadecabacter | unclassified |
| Otu077237 | 0.981 | 1 | 0.99 | 0.001 | Bacteria | Proteobacteria | Alphaproteobacteria | Rhodobacterales | Rhodobacteraceae | unclassified | unclassified |
| Otu077267 | 0.7346 | 1 | 0.857 | 0.011 | Bacteria | Proteobacteria | Alphaproteobacteria | Rhodobacterales | Rhodobacteraceae | Octadecabacter | unclassified |
| Otu077275 | 1 | 1 | 1 | 0.001 | Bacteria | Proteobacteria | Alphaproteobacteria | Rhodobacterales | Rhodobacteraceae | Marivita | unclassified |
| Otu077308 | 0.9483 | 1 | 0.974 | 0.002 | Bacteria | Proteobacteria | Alphaproteobacteria | Rhodobacterales | Rhodobacteraceae | Phaeobacter | unclassified |
| Otu077340 | 1 | 1 | 1 | 0.001 | Bacteria | Proteobacteria | Alphaproteobacteria | Rhodobacterales | Rhodobacteraceae | Octadecabacter | unclassified |
| Otu077345 | 1 | 0.6667 | 0.816 | 0.01 | Bacteria | Proteobacteria | Alphaproteobacteria | Rhodobacterales | Rhodobacteraceae | unclassified | unclassified |
| Otu077350 | 0.9534 | 1 | 0.976 | 0.001 | Bacteria | Proteobacteria | Alphaproteobacteria | Rhodobacterales | Rhodobacteraceae | unclassified | unclassified |
| Otu077801 | 1 | 1 | 1 | 0.001 | Bacteria | Cyanobacteria | Synechococcophycideae | Synechococcales | Synechococcaceae | unclassified | unclassified |
| Otu078780 | 0.9533 | 1 | 0.976 | 0.001 | Bacteria | Proteobacteria | Alphaproteobacteria | Rhodobacterales | Rhodobacteraceae | unclassified | unclassified |
| Otu078854 | 0.9082 | 1 | 0.953 | 0.002 | Bacteria | Proteobacteria | Alphaproteobacteria | Rhodobacterales | Rhodobacteraceae | unclassified | unclassified |
| Otu078876 | 0.9186 | 0.6667 | 0.783 | 0.024 | Bacteria | Proteobacteria | Alphaproteobacteria | unclassified | unclassified | unclassified | unclassified |
| Otu078883 | 0.9825 | 1 | 0.991 | 0.001 | Bacteria | Proteobacteria | Alphaproteobacteria | Rhodobacterales | Rhodobacteraceae | Octadecabacter | unclassified |
| Otu078929 | 0.8614 | 1 | 0.928 | 0.007 | Bacteria | Proteobacteria | Alphaproteobacteria | Rhodobacterales | Rhodobacteraceae | unclassified | unclassified |
| Otu079006 | 0.8482 | 1 | 0.921 | 0.003 | Bacteria | Proteobacteria | Alphaproteobacteria | Rhodobacterales | Rhodobacteraceae | unclassified | unclassified |
| Otu079018 | 1 | 1 | 1 | 0.001 | Bacteria | Proteobacteria | Alphaproteobacteria | Rhodobacterales | Rhodobacteraceae | Octadecabacter | unclassified |
| Otu079028 | 0.9798 | 0.6667 | 0.808 | 0.009 | Bacteria | Proteobacteria | Alphaproteobacteria | Rhodobacterales | unclassified | unclassified | unclassified |
| Otu079037 | 0.9373 | 0.6667 | 0.79 | 0.022 | Bacteria | Proteobacteria | Alphaproteobacteria | Rhodobacterales | Rhodobacteraceae | unclassified | unclassified |
| Otu079778 | 1 | 1 | 1 | 0.001 | Bacteria | Proteobacteria | Alphaproteobacteria | Rhodobacterales | Rhodobacteraceae | unclassified | unclassified |
| Otu080370 | 0.8206 | 1 | 0.906 | 0.004 | Bacteria | Proteobacteria | Alphaproteobacteria | Rhodospirillales | Rhodospirillaceae | unclassified | unclassified |
| Otu080511 | 0.8198 | 0.6667 | 0.739 | 0.04 | Bacteria | Proteobacteria | unclassified | unclassified | unclassified | unclassified | unclassified |
| Otu081116 | 0.9339 | 1 | 0.966 | 0.001 | Bacteria | Proteobacteria | unclassified | unclassified | unclassified | unclassified | unclassified |
| Otu081117 | 1 | 0.6667 | 0.816 | 0.009 | Bacteria | Proteobacteria | Alphaproteobacteria | Rhodospirillales | Rhodospirillaceae | unclassified | unclassified |
| Otu082421 | 0.9133 | 0.6667 | 0.78 | 0.01 | Bacteria | Proteobacteria | Betaproteobacteria | unclassified | unclassified | unclassified | unclassified |
| Otu082427 | 1 | 0.6667 | 0.816 | 0.01 | Bacteria | Proteobacteria | unclassified | unclassified | unclassified | unclassified | unclassified |
| Otu084181 | 0.9497 | 1 | 0.975 | 0.002 | Bacteria | Actinobacteria | Acidimicrobiia | Acidimicrobiales | OCS155 | unclassified | unclassified |
| Otu084381 | 0.9735 | 1 | 0.987 | 0.001 | Bacteria | Actinobacteria | Acidimicrobiia | Acidimicrobiales | OCS155 | unclassified | unclassified |
| Otu084396 | 1 | 1 | 1 | 0.001 | Bacteria | Actinobacteria | Acidimicrobiia | Acidimicrobiales | OCS155 | unclassified | unclassified |
| Otu084404 | 1 | 1 | 1 | 0.001 | Bacteria | Actinobacteria | Acidimicrobiia | Acidimicrobiales | OCS155 | unclassified | unclassified |
| Otu084440 | 0.9861 | 1 | 0.993 | 0.001 | Bacteria | Actinobacteria | Acidimicrobiia | Acidimicrobiales | OCS155 | unclassified | unclassified |
| Otu084482 | 1 | 1 | 1 | 0.001 | Bacteria | Actinobacteria | Acidimicrobiia | Acidimicrobiales | wb1_P06 | unclassified | unclassified |
| Otu084526 | 1 | 1 | 1 | 0.001 | Bacteria | Actinobacteria | Acidimicrobiia | Acidimicrobiales | OCS155 | unclassified | unclassified |
| Otu084543 | 1 | 1 | 1 | 0.001 | Bacteria | Actinobacteria | Acidimicrobiia | Acidimicrobiales | OCS155 | unclassified | unclassified |
| Otu084571 | 1 | 1 | 1 | 0.001 | Bacteria | Actinobacteria | Acidimicrobiia | Acidimicrobiales | OCS155 | unclassified | unclassified |
| Otu084579 | 1 | 0.6667 | 0.816 | 0.01 | Bacteria | Actinobacteria | Acidimicrobiia | Acidimicrobiales | OCS155 | unclassified | unclassified |
| Otu084583 | 1 | 0.6667 | 0.816 | 0.009 | Bacteria | Actinobacteria | Acidimicrobiia | Acidimicrobiales | OCS155 | unclassified | unclassified |
| Otu084594 | 1 | 1 | 1 | 0.001 | Bacteria | Actinobacteria | Acidimicrobiia | Acidimicrobiales | OCS155 | unclassified | unclassified |
| Otu084652 | 1 | 1 | 1 | 0.001 | Bacteria | Actinobacteria | Acidimicrobiia | Acidimicrobiales | OCS155 | unclassified | unclassified |
| Otu085025 | 1 | 0.6667 | 0.816 | 0.007 | Bacteria | Proteobacteria | Gammaproteobacteria | Vibrionales | Vibrionaceae | unclassified | unclassified |
| Otu085503 | 1 | 0.6667 | 0.816 | 0.01 | Bacteria | Proteobacteria | Gammaproteobacteria | Alteromonadales | Colwelliaceae | unclassified | unclassified |
| Otu085525 | 1 | 0.6667 | 0.816 | 0.007 | Bacteria | Proteobacteria | Gammaproteobacteria | Vibrionales | Pseudoalteromonadaceae | Pseudoalteromonas | unclassified |
| Otu085547 | 0.8436 | 1 | 0.918 | 0.001 | Bacteria | Proteobacteria | Gammaproteobacteria | Alteromonadales | unclassified | unclassified | unclassified |
| Otu085571 | 1 | 0.6667 | 0.816 | 0.009 | Bacteria | Proteobacteria | Gammaproteobacteria | Vibrionales | Pseudoalteromonadaceae | Pseudoalteromonas | unclassified |
| Otu085928 | 1 | 1 | 1 | 0.001 | Bacteria | Actinobacteria | Acidimicrobiia | Acidimicrobiales | OCS155 | unclassified | unclassified |
| Otu085934 | 0.9338 | 0.6667 | 0.789 | 0.023 | Bacteria | Actinobacteria | Acidimicrobiia | Acidimicrobiales | OCS155 | unclassified | unclassified |
| Otu087366 | 0.9212 | 0.6667 | 0.784 | 0.026 | Bacteria | Bacteroidetes | Saprospirae | Saprospirales | Saprospiraceae | unclassified | unclassified |
| Otu091152 | 0.8799 | 0.6667 | 0.766 | 0.026 | Bacteria | Verrucomicrobia | Verrucomicrobiae | Verrucomicrobiales | Verrucomicrobiaceae | unclassified | unclassified |
| Otu101873 | 0.9028 | 1 | 0.95 | 0.002 | Archaea | Euryarchaeota | Thermoplasmata | E2 | Marine_group_II | unclassified | unclassified |
| Otu107332 | 0.89 | 0.6667 | 0.77 | 0.028 | Bacteria | Proteobacteria | Gammaproteobacteria | unclassified | unclassified | unclassified | unclassified |
| Otu108562 | 0.9488 | 0.6667 | 0.795 | 0.019 | Bacteria | Proteobacteria | Gammaproteobacteria | Alteromonadales | unclassified | unclassified | unclassified |
| Otu109454 | 0.9488 | 0.6667 | 0.795 | 0.019 | Bacteria | Proteobacteria | Gammaproteobacteria | Alteromonadales | unclassified | unclassified | unclassified |
| Otu110106 | 1 | 1 | 1 | 0.001 | Bacteria | Proteobacteria | Gammaproteobacteria | Oceanospirillales | Halomonadaceae | Candidatus_Portiera | unclassified |
| Otu110313 | 1 | 0.6667 | 0.816 | 0.009 | Bacteria | Proteobacteria | Gammaproteobacteria | unclassified | unclassified | unclassified | unclassified |
| Otu110784 | 0.9413 | 0.6667 | 0.792 | 0.015 | Bacteria | Proteobacteria | Gammaproteobacteria | unclassified | unclassified | unclassified | unclassified |
| Otu111378 | 0.8654 | 0.6667 | 0.76 | 0.038 | Bacteria | Proteobacteria | Gammaproteobacteria | unclassified | unclassified | unclassified | unclassified |
| Otu113951 | 0.8691 | 1 | 0.932 | 0.006 | Bacteria | Proteobacteria | Gammaproteobacteria | unclassified | unclassified | unclassified | unclassified |
| Otu116238 | 1 | 1 | 1 | 0.001 | Bacteria | Proteobacteria | Gammaproteobacteria | Alteromonadales | unclassified | unclassified | unclassified |
| Otu116422 | 1 | 1 | 1 | 0.001 | Bacteria | Bacteroidetes | Flavobacteriia | Flavobacteriales | unclassified | unclassified | unclassified |
| Otu116453 | 1 | 0.6667 | 0.816 | 0.01 | Bacteria | Bacteroidetes | Flavobacteriia | Flavobacteriales | unclassified | unclassified | unclassified |
| Otu116570 | 0.9677 | 1 | 0.984 | 0.001 | Bacteria | Bacteroidetes | Flavobacteriia | Flavobacteriales | Flavobacteriaceae | Formosa | unclassified |

| | | | | | | | | | | | |
|---|---|---|---|---|---|---|---|---|---|---|---|
| Otu116596 | 1 | 0.6667 | 0.816 | 0.009 | Bacteria | Bacteroidetes | Flavobacteriia | Flavobacteriales | Flavobacteriaceae | unclassified | unclassified |
| Otu116597 | 0.9824 | 1 | 0.991 | 0.001 | Bacteria | Bacteroidetes | Flavobacteriia | Flavobacteriales | unclassified | unclassified | unclassified |
| Otu116601 | 0.8261 | 0.6667 | 0.742 | 0.049 | Bacteria | Bacteroidetes | Flavobacteriia | Flavobacteriales | Flavobacteriaceae | unclassified | unclassified |
| Otu116684 | 0.9392 | 0.6667 | 0.791 | 0.023 | Bacteria | Bacteroidetes | Flavobacteriia | Flavobacteriales | Flavobacteriaceae | unclassified | unclassified |
| Otu116707 | 1 | 0.6667 | 0.816 | 0.007 | Bacteria | Bacteroidetes | Flavobacteriia | Flavobacteriales | Flavobacteriaceae | unclassified | unclassified |
| Otu116714 | 0.9399 | 0.6667 | 0.792 | 0.016 | Bacteria | Bacteroidetes | Flavobacteriia | Flavobacteriales | Cryomorphaceae | unclassified | unclassified |
| Otu116741 | 1 | 1 | 1 | 0.001 | Bacteria | Bacteroidetes | Flavobacteriia | Flavobacteriales | unclassified | unclassified | unclassified |
| Otu116744 | 0.9502 | 1 | 0.975 | 0.001 | Bacteria | Bacteroidetes | Flavobacteriia | Flavobacteriales | Flavobacteriaceae | unclassified | unclassified |
| Otu116780 | 0.9517 | 1 | 0.976 | 0.001 | Bacteria | Bacteroidetes | Flavobacteriia | Flavobacteriales | Flavobacteriaceae | unclassified | unclassified |
| Otu116785 | 0.9364 | 1 | 0.968 | 0.001 | Bacteria | Bacteroidetes | Flavobacteriia | Flavobacteriales | Flavobacteriaceae | Sediminicola | unclassified |
| Otu116840 | 0.9504 | 1 | 0.975 | 0.001 | Bacteria | Bacteroidetes | Flavobacteriia | Flavobacteriales | Flavobacteriaceae | unclassified | unclassified |
| Otu116843 | 0.9566 | 1 | 0.978 | 0.001 | Bacteria | Bacteroidetes | Flavobacteriia | Flavobacteriales | unclassified | unclassified | unclassified |
| Otu116854 | 0.973 | 1 | 0.986 | 0.001 | Bacteria | Bacteroidetes | unclassified | unclassified | unclassified | unclassified | unclassified |
| Otu116914 | 0.9369 | 0.6667 | 0.79 | 0.026 | Bacteria | Bacteroidetes | Flavobacteriia | Flavobacteriales | unclassified | unclassified | unclassified |
| Otu117272 | 0.8371 | 1 | 0.915 | 0.003 | Bacteria | Proteobacteria | Gammaproteobacteria | HTCC2188 | HTCC2089 | unclassified | unclassified |
| Otu117442 | 0.9501 | 1 | 0.975 | 0.001 | Bacteria | Proteobacteria | Gammaproteobacteria | Alteromonadales | unclassified | unclassified | unclassified |
| Otu118104 | 0.8605 | 0.6667 | 0.757 | 0.037 | Bacteria | Proteobacteria | Gammaproteobacteria | Alteromonadales | unclassified | unclassified | unclassified |
| Otu118154 | 1 | 1 | 1 | 0.001 | Bacteria | Bacteroidetes | Flavobacteriia | Flavobacteriales | Cryomorphaceae | Fluviicola | unclassified |
| Otu118175 | 0.9711 | 0.6667 | 0.805 | 0.01 | Bacteria | Bacteroidetes | Flavobacteriia | Flavobacteriales | Flavobacteriaceae | unclassified | unclassified |
| Otu118238 | 0.6385 | 1 | 0.799 | 0.037 | Bacteria | Bacteroidetes | Flavobacteriia | Flavobacteriales | Flavobacteriaceae | Polaribacter | unclassified |
| Otu118262 | 1 | 0.6667 | 0.816 | 0.007 | Bacteria | Bacteroidetes | Flavobacteriia | Flavobacteriales | NS9 | unclassified | unclassified |
| Otu118371 | 0.9904 | 1 | 0.995 | 0.001 | Bacteria | Bacteroidetes | Flavobacteriia | Flavobacteriales | unclassified | unclassified | unclassified |
| Otu118443 | 0.9456 | 0.6667 | 0.794 | 0.021 | Bacteria | Bacteroidetes | Flavobacteriia | Flavobacteriales | Flavobacteriaceae | unclassified | unclassified |
| Otu118546 | 0.9012 | 0.6667 | 0.775 | 0.016 | Bacteria | Bacteroidetes | Flavobacteriia | Flavobacteriales | Flavobacteriaceae | Lacinutrix | unclassified |
| Otu118550 | 0.9596 | 1 | 0.98 | 0.001 | Bacteria | Bacteroidetes | Flavobacteriia | Flavobacteriales | Cryomorphaceae | Fluviicola | unclassified |
| Otu118584 | 1 | 1 | 1 | 0.001 | Bacteria | Bacteroidetes | Flavobacteriia | Flavobacteriales | Flavobacteriaceae | unclassified | unclassified |
| Otu118591 | 1 | 0.6667 | 0.816 | 0.007 | Bacteria | Bacteroidetes | Flavobacteriia | Flavobacteriales | unclassified | unclassified | unclassified |
| Otu118655 | 1 | 0.6667 | 0.816 | 0.01 | Bacteria | Bacteroidetes | Flavobacteriia | Flavobacteriales | unclassified | unclassified | unclassified |
| Otu118686 | 0.8317 | 1 | 0.912 | 0.006 | Bacteria | Cyanobacteria | Synechococcophycideae | Synechococcales | Synechococcaceae | Synechococcus | unclassified |
| Otu118697 | 0.8674 | 0.6667 | 0.76 | 0.044 | Bacteria | Cyanobacteria | Synechococcophycideae | Synechococcales | Synechococcaceae | Synechococcus | unclassified |
| Otu118701 | 0.8833 | 1 | 0.94 | 0.003 | Bacteria | Cyanobacteria | Synechococcophycideae | Synechococcales | Synechococcaceae | Synechococcus | unclassified |
| Otu118921 | 0.7405 | 1 | 0.861 | 0.012 | Bacteria | Cyanobacteria | Synechococcophycideae | Synechococcales | Synechococcaceae | unclassified | unclassified |
| Otu118960 | 1 | 1 | 1 | 0.001 | Bacteria | Cyanobacteria | Synechococcophycideae | Synechococcales | Synechococcaceae | unclassified | unclassified |
| Otu119034 | 1 | 0.6667 | 0.816 | 0.009 | Bacteria | Bacteroidetes | Flavobacteriia | Flavobacteriales | Cryomorphaceae | Fluviicola | unclassified |
| Otu119119 | 0.9413 | 0.6667 | 0.792 | 0.015 | Bacteria | Cyanobacteria | Synechococcophycideae | Synechococcales | Synechococcaceae | Synechococcus | unclassified |
| Otu119181 | 0.8653 | 1 | 0.93 | 0.005 | Bacteria | Cyanobacteria | Synechococcophycideae | Synechococcales | Synechococcaceae | Synechococcus | unclassified |
| Otu119182 | 0.8221 | 1 | 0.907 | 0.014 | Bacteria | Cyanobacteria | Synechococcophycideae | Synechococcales | Synechococcaceae | Synechococcus | unclassified |
| Otu119208 | 0.9812 | 1 | 0.991 | 0.001 | Bacteria | Bacteroidetes | Flavobacteriia | Flavobacteriales | unclassified | unclassified | unclassified |
| Otu119214 | 1 | 0.6667 | 0.816 | 0.01 | Bacteria | Bacteroidetes | Flavobacteriia | Flavobacteriales | Flavobacteriaceae | Olleya | unclassified |
| Otu119232 | 0.8291 | 1 | 0.911 | 0.011 | Bacteria | Cyanobacteria | Synechococcophycideae | Synechococcales | Synechococcaceae | Synechococcus | unclassified |
| Otu119347 | 1 | 0.6667 | 0.816 | 0.01 | Bacteria | Bacteroidetes | Flavobacteriia | Flavobacteriales | Flavobacteriaceae | unclassified | unclassified |
| Otu119421 | 0.9615 | 1 | 0.981 | 0.001 | Bacteria | Bacteroidetes | Flavobacteriia | Flavobacteriales | Cryomorphaceae | Fluviicola | unclassified |
| Otu119518 | 1 | 1 | 1 | 0.001 | Bacteria | Bacteroidetes | Flavobacteriia | Flavobacteriales | Flavobacteriaceae | Formosa | unclassified |
| Otu119531 | 0.959 | 1 | 0.979 | 0.001 | Bacteria | Bacteroidetes | Flavobacteriia | Flavobacteriales | Flavobacteriaceae | unclassified | unclassified |
| Otu119593 | 0.9708 | 1 | 0.985 | 0.001 | Bacteria | Bacteroidetes | Flavobacteriia | Flavobacteriales | unclassified | unclassified | unclassified |
| Otu119600 | 0.9382 | 1 | 0.969 | 0.001 | Bacteria | Bacteroidetes | Flavobacteriia | Flavobacteriales | Cryomorphaceae | Fluviicola | unclassified |
| Otu119655 | 0.916 | 0.6667 | 0.781 | 0.026 | Bacteria | Bacteroidetes | Flavobacteriia | Flavobacteriales | Cryomorphaceae | unclassified | unclassified |
| Otu119806 | 1 | 1 | 1 | 0.001 | Bacteria | Bacteroidetes | Flavobacteriia | Flavobacteriales | Cryomorphaceae | unclassified | unclassified |
| Otu119816 | 0.9142 | 1 | 0.956 | 0.003 | Bacteria | Bacteroidetes | Flavobacteriia | Flavobacteriales | Cryomorphaceae | Fluviicola | unclassified |
| Otu119887 | 0.8942 | 1 | 0.946 | 0.004 | Bacteria | Bacteroidetes | unclassified | unclassified | unclassified | unclassified | unclassified |
| Otu119971 | 1 | 0.6667 | 0.816 | 0.007 | Bacteria | Bacteroidetes | Flavobacteriia | Flavobacteriales | unclassified | unclassified | unclassified |
| Otu120086 | 0.9565 | 0.6667 | 0.799 | 0.015 | Bacteria | Bacteroidetes | Flavobacteriia | Flavobacteriales | Flavobacteriaceae | Formosa | unclassified |
| Otu120187 | 0.9287 | 0.6667 | 0.787 | 0.015 | Bacteria | Bacteroidetes | Flavobacteriia | Flavobacteriales | Flavobacteriaceae | unclassified | unclassified |
| Otu120189 | 1 | 1 | 1 | 0.001 | Bacteria | Bacteroidetes | Flavobacteriia | Flavobacteriales | Cryomorphaceae | Fluviicola | unclassified |
| Otu120880 | 1 | 1 | 1 | 0.001 | Bacteria | Proteobacteria | Gammaproteobacteria | Alteromonadales | Alteromonadaceae | unclassified | unclassified |
| Otu125013 | 0.8646 | 0.6667 | 0.759 | 0.025 | Bacteria | Proteobacteria | Gammaproteobacteria | unclassified | unclassified | unclassified | unclassified |
| Otu125073 | 1 | 1 | 1 | 0.001 | Bacteria | Proteobacteria | Gammaproteobacteria | Oceanospirillales | Halomonadaceae | Candidatus_Portiera | unclassified |
| Otu125330 | 1 | 1 | 1 | 0.001 | Bacteria | Proteobacteria | Gammaproteobacteria | Oceanospirillales | Halomonadaceae | Candidatus_Portiera | unclassified |
| Otu125502 | 1 | 1 | 1 | 0.001 | Bacteria | Proteobacteria | Gammaproteobacteria | unclassified | unclassified | unclassified | unclassified |
| Otu125518 | 1 | 0.6667 | 0.816 | 0.01 | Bacteria | Proteobacteria | Gammaproteobacteria | Oceanospirillales | Halomonadaceae | Candidatus_Portiera | unclassified |
| Otu125579 | 0.7752 | 1 | 0.88 | 0.018 | Bacteria | Proteobacteria | Gammaproteobacteria | unclassified | unclassified | unclassified | unclassified |
| Otu125647 | 1 | 0.6667 | 0.816 | 0.009 | Bacteria | Planctomycetes | Planctomycetia | Pirellulales | Pirellulaceae | unclassified | unclassified |
| Otu125714 | 0.9548 | 0.6667 | 0.798 | 0.009 | Bacteria | Proteobacteria | Gammaproteobacteria | Alteromonadales | HTCC2188 | HTCC | unclassified |
| Otu125925 | 1 | 0.6667 | 0.816 | 0.01 | Bacteria | Proteobacteria | Gammaproteobacteria | Oceanospirillales | Halomonadaceae | Candidatus_Portiera | unclassified |
| Otu126010 | 0.9635 | 1 | 0.982 | 0.001 | Bacteria | Proteobacteria | Gammaproteobacteria | Oceanospirillales | Halomonadaceae | Candidatus_Portiera | unclassified |
| Otu126046 | 1 | 1 | 1 | 0.001 | Bacteria | Proteobacteria | Gammaproteobacteria | unclassified | unclassified | unclassified | unclassified |
| Otu126113 | 0.9383 | 0.6667 | 0.791 | 0.026 | Bacteria | Proteobacteria | Gammaproteobacteria | Alteromonadales | unclassified | unclassified | unclassified |
| Otu126115 | 1 | 0.6667 | 0.816 | 0.01 | Bacteria | Proteobacteria | Gammaproteobacteria | unclassified | unclassified | unclassified | unclassified |
| Otu126135 | 0.9663 | 0.6667 | 0.803 | 0.007 | Bacteria | Proteobacteria | Gammaproteobacteria | Oceanospirillales | Halomonadaceae | Candidatus_Portiera | unclassified |
| Otu126758 | 1 | 0.6667 | 0.816 | 0.009 | Bacteria | Proteobacteria | Gammaproteobacteria | unclassified | unclassified | unclassified | unclassified |
| Otu127192 | 0.941 | 0.6667 | 0.792 | 0.015 | Bacteria | Proteobacteria | Gammaproteobacteria | Oceanospirillales | unclassified | unclassified | unclassified |
| Otu127218 | 1 | 1 | 1 | 0.001 | Bacteria | Proteobacteria | Gammaproteobacteria | unclassified | unclassified | unclassified | unclassified |
| Otu127331 | 1 | 1 | 1 | 0.001 | Bacteria | Proteobacteria | Gammaproteobacteria | Oceanospirillales | Halomonadaceae | Candidatus_Portiera | unclassified |
| Otu127416 | 1 | 0.6667 | 0.816 | 0.007 | Bacteria | Proteobacteria | Gammaproteobacteria | unclassified | unclassified | unclassified | unclassified |
| Otu127872 | 1 | 0.6667 | 0.816 | 0.007 | Bacteria | Proteobacteria | Gammaproteobacteria | HTCC2188 | HTCC2089 | unclassified | unclassified |
| Otu128443 | 1 | 1 | 1 | 0.001 | Bacteria | Proteobacteria | Gammaproteobacteria | unclassified | unclassified | unclassified | unclassified |
| Otu129153 | 1 | 0.6667 | 0.816 | 0.007 | Bacteria | Verrucomicrobia | Opitutae | Puniceicoccales | Puniceicoccaceae | Coraliomargarita | unclassified |
| Otu129311 | 1 | 1 | 1 | 0.001 | Bacteria | Proteobacteria | Gammaproteobacteria | Oceanospirillales | Halomonadaceae | Candidatus_Portiera | unclassified |
| Otu129406 | 1 | 0.6667 | 0.816 | 0.009 | Bacteria | Proteobacteria | Gammaproteobacteria | Pseudomonadales | Pseudomonadaceae | unclassified | unclassified |
| Otu129888 | 0.9226 | 1 | 0.961 | 0.001 | Bacteria | Proteobacteria | Alphaproteobacteria | Rhizobiales | unclassified | unclassified | unclassified |
| Otu129906 | 1 | 1 | 1 | 0.001 | Bacteria | Proteobacteria | Alphaproteobacteria | unclassified | unclassified | unclassified | unclassified |
| Otu129994 | 1 | 1 | 1 | 0.001 | Bacteria | Proteobacteria | Betaproteobacteria | Rhodocyclales | Rhodocyclaceae | unclassified | unclassified |
| Otu129998 | 0.9123 | 0.6667 | 0.78 | 0.015 | Bacteria | Proteobacteria | Betaproteobacteria | Burkholderiales | Oxalobacteraceae | Polynucleobacter | unclassified |
| Otu130115 | 0.9388 | 0.6667 | 0.791 | 0.015 | Bacteria | Proteobacteria | Betaproteobacteria | Rhodocyclales | Rhodocyclaceae | unclassified | unclassified |
| Otu130854 | 0.8094 | 0.6667 | 0.735 | 0.05 | Bacteria | Proteobacteria | Alphaproteobacteria | unclassified | unclassified | unclassified | unclassified |
| Otu131583 | 0.9824 | 1 | 0.991 | 0.001 | Bacteria | Proteobacteria | Betaproteobacteria | Burkholderiales | unclassified | unclassified | unclassified |
| Otu131693 | 1 | 1 | 1 | 0.001 | Bacteria | Proteobacteria | Alphaproteobacteria | Rhodobacterales | unclassified | unclassified | unclassified |
| Otu131809 | 1 | 1 | 1 | 0.001 | Bacteria | Proteobacteria | Alphaproteobacteria | unclassified | unclassified | unclassified | unclassified |
| Otu132172 | 0.9703 | 1 | 0.985 | 0.001 | Bacteria | Proteobacteria | Gammaproteobacteria | unclassified | unclassified | unclassified | unclassified |
| Otu132354 | 0.9754 | 1 | 0.988 | 0.001 | Bacteria | Proteobacteria | Alphaproteobacteria | Rhodobacterales | Rhodobacteraceae | unclassified | unclassified |
| Otu132365 | 1 | 1 | 1 | 0.001 | Bacteria | Proteobacteria | Alphaproteobacteria | Rhodobacterales | unclassified | unclassified | unclassified |
| Otu132400 | 0.9738 | 1 | 0.987 | 0.001 | Bacteria | Proteobacteria | Alphaproteobacteria | Rhodobacterales | unclassified | unclassified | unclassified |
| Otu132412 | 1 | 1 | 1 | 0.001 | Bacteria | Proteobacteria | Alphaproteobacteria | Rhodobacterales | Rhodobacteraceae | unclassified | unclassified |
| Otu132927 | 1 | 0.6667 | 0.816 | 0.007 | Bacteria | Proteobacteria | Gammaproteobacteria | unclassified | unclassified | unclassified | unclassified |
| Otu133165 | 0.9872 | 1 | 0.994 | 0.001 | Bacteria | Proteobacteria | Betaproteobacteria | Methylophilales | Methylophilaceae | Methylotenera | mobilis |
| Otu133167 | 0.9338 | 0.6667 | 0.789 | 0.023 | Bacteria | Proteobacteria | Betaproteobacteria | Methylophilales | Methylophilaceae | unclassified | unclassified |
| Otu133291 | 1 | 1 | 1 | 0.001 | Bacteria | Proteobacteria | Alphaproteobacteria | Rhodobacterales | unclassified | unclassified | unclassified |
| Otu133296 | 1 | 0.6667 | 0.816 | 0.009 | Bacteria | Proteobacteria | Alphaproteobacteria | Rhodobacterales | Rhodobacteraceae | Loktanella | vestfoldensis |
| Otu133313 | 0.9742 | 1 | 0.987 | 0.001 | Bacteria | Proteobacteria | Alphaproteobacteria | Rhodobacterales | Rhodobacteraceae | Octadecabacter | unclassified |
| Otu133318 | 1 | 0.6667 | 0.816 | 0.009 | Bacteria | Proteobacteria | Alphaproteobacteria | Rhodobacterales | Rhodobacteraceae | Octadecabacter | unclassified |

| | | | | | | | | | | | |
|---|---|---|---|---|---|---|---|---|---|---|---|
| Otu133403 | 1 | 1 | 1 | 0.001 | Bacteria | Proteobacteria | Alphaproteobacteria | Rhodobacterales | Rhodobacteraceae | unclassified | unclassified |
| Otu133751 | 1 | 1 | 1 | 0.001 | Bacteria | Proteobacteria | Alphaproteobacteria | Rhodobacterales | Rhodobacteraceae | unclassified | unclassified |
| Otu133769 | 0.9723 | 1 | 0.986 | 0.001 | Bacteria | Proteobacteria | Alphaproteobacteria | Rhodobacterales | Rhodobacteraceae | unclassified | unclassified |
| Otu133871 | 0.942 | 0.6667 | 0.792 | 0.022 | Bacteria | Proteobacteria | Alphaproteobacteria | Rhodobacterales | Rhodobacteraceae | Phaeobacter | unclassified |
| Otu133915 | 1 | 1 | 1 | 0.001 | Bacteria | Proteobacteria | Alphaproteobacteria | Rhodobacterales | Rhodobacteraceae | unclassified | unclassified |
| Otu133929 | 1 | 1 | 1 | 0.001 | Bacteria | Proteobacteria | Alphaproteobacteria | Rhodobacterales | unclassified | unclassified | unclassified |
| Otu133956 | 0.8032 | 1 | 0.896 | 0.006 | Bacteria | Proteobacteria | Alphaproteobacteria | Rhodobacterales | Rhodobacteraceae | Octadecabacter | unclassified |
| Otu133959 | 0.82 | 0.6667 | 0.739 | 0.041 | Bacteria | Proteobacteria | Alphaproteobacteria | Rhodobacterales | Rhodobacteraceae | unclassified | unclassified |
| Otu133993 | 1 | 1 | 1 | 0.001 | Bacteria | Proteobacteria | Alphaproteobacteria | Rhodobacterales | Rhodobacteraceae | Rhodobacter | unclassified |
| Otu134019 | 1 | 0.6667 | 0.816 | 0.009 | Bacteria | Proteobacteria | Alphaproteobacteria | Rhodobacterales | Rhodobacteraceae | Octadecabacter | unclassified |
| Otu134021 | 1 | 0.6667 | 0.816 | 0.009 | Bacteria | Proteobacteria | Alphaproteobacteria | Rhodobacterales | Rhodobacteraceae | unclassified | unclassified |
| Otu134029 | 1 | 0.6667 | 0.816 | 0.009 | Bacteria | Proteobacteria | Alphaproteobacteria | Rhodobacterales | Rhodobacteraceae | unclassified | unclassified |
| Otu134115 | 1 | 1 | 1 | 0.001 | Bacteria | Proteobacteria | Alphaproteobacteria | Rhodobacterales | Rhodobacteraceae | Octadecabacter | unclassified |
| Otu134128 | 0.929 | 1 | 0.964 | 0.001 | Bacteria | Proteobacteria | Alphaproteobacteria | Rhodobacterales | Rhodobacteraceae | Octadecabacter | unclassified |
| Otu134594 | 1 | 0.6667 | 0.816 | 0.007 | Bacteria | Proteobacteria | Gammaproteobacteria | unclassified | unclassified | unclassified | unclassified |
| Otu134642 | 0.9676 | 0.6667 | 0.803 | 0.009 | Bacteria | Proteobacteria | Gammaproteobacteria | Thiotrichales | Piscirickettsiaceae | unclassified | unclassified |
| Otu137032 | 1 | 1 | 1 | 0.001 | Bacteria | Proteobacteria | Gammaproteobacteria | unclassified | unclassified | unclassified | unclassified |
| Otu139245 | 1 | 1 | 1 | 0.001 | Bacteria | Bacteroidetes | Sphingobacteriia | Sphingobacteriales | NS11-12 | unclassified | unclassified |
| Otu140409 | 0.972 | 1 | 0.986 | 0.001 | Bacteria | unclassified | unclassified | unclassified | unclassified | unclassified | unclassified |
| Otu140449 | 0.9037 | 1 | 0.951 | 0.003 | Bacteria | unclassified | unclassified | unclassified | unclassified | unclassified | unclassified |
| Otu140450 | 1 | 0.6667 | 0.816 | 0.009 | Bacteria | unclassified | unclassified | unclassified | unclassified | unclassified | unclassified |
| Otu140532 | 1 | 1 | 1 | 0.001 | Bacteria | Proteobacteria | unclassified | unclassified | unclassified | unclassified | unclassified |
| Otu140736 | 1 | 1 | 1 | 0.001 | Bacteria | Bacteroidetes | Sphingobacteriia | Sphingobacteriales | NS11-12 | unclassified | unclassified |
| Otu140996 | 1 | 0.6667 | 0.816 | 0.009 | Bacteria | Proteobacteria | Alphaproteobacteria | Rhodospirillales | Rhodospirillaceae | unclassified | unclassified |
| Otu141001 | 1 | 0.6667 | 0.816 | 0.007 | Bacteria | Proteobacteria | unclassified | unclassified | unclassified | unclassified | unclassified |
| Otu141060 | 0.9005 | 0.6667 | 0.775 | 0.03 | Bacteria | unclassified | unclassified | unclassified | unclassified | unclassified | unclassified |
| Otu141193 | 0.863 | 0.6667 | 0.758 | 0.02 | Bacteria | Proteobacteria | Epsilonproteobacteria | Campylobacterales | Campylobacteraceae | Arcobacter | unclassified |
| Otu142574 | 1 | 1 | 1 | 0.001 | Bacteria | Proteobacteria | Alphaproteobacteria | Rhodobacterales | Hyphomonadaceae | Hirschia | baltica |
| Otu143388 | 0.9011 | 1 | 0.949 | 0.003 | Bacteria | Bacteroidetes | Flavobacteriia | Flavobacteriales | NS9 | unclassified | unclassified |
| Otu143624 | 1 | 1 | 1 | 0.001 | Bacteria | Bacteroidetes | Flavobacteriia | Flavobacteriales | Cryomorphaceae | unclassified | unclassified |
| Otu144040 | 0.8684 | 0.6667 | 0.761 | 0.034 | Bacteria | Bacteroidetes | Flavobacteriia | Flavobacteriales | unclassified | unclassified | unclassified |
| Otu144907 | 1 | 0.6667 | 0.816 | 0.01 | Bacteria | Bacteroidetes | Flavobacteriia | Flavobacteriales | unclassified | unclassified | unclassified |
| Otu144999 | 1 | 0.6667 | 0.816 | 0.01 | Bacteria | Bacteroidetes | Flavobacteriia | Flavobacteriales | unclassified | unclassified | unclassified |
| Otu145243 | 1 | 0.6667 | 0.816 | 0.007 | Bacteria | Proteobacteria | Alphaproteobacteria | Rickettsiales | Pelagibacteraceae | unclassified | unclassified |
| Otu145501 | 1 | 0.6667 | 0.816 | 0.007 | Bacteria | Proteobacteria | Alphaproteobacteria | Rhodobacterales | Rhodobacteraceae | unclassified | unclassified |
| Otu148604 | 0.9747 | 1 | 0.987 | 0.001 | Bacteria | Proteobacteria | Deltaproteobacteria | PB19 | unclassified | unclassified | unclassified |
| Otu148655 | 1 | 1 | 1 | 0.001 | Bacteria | Proteobacteria | Alphaproteobacteria | Rhodobacterales | Rhodobacteraceae | Octadecabacter | unclassified |
| Otu148664 | 1 | 1 | 1 | 0.001 | Bacteria | Proteobacteria | Alphaproteobacteria | Rhodobacterales | Rhodobacteraceae | unclassified | unclassified |
| Otu148690 | 1 | 1 | 1 | 0.001 | Bacteria | Proteobacteria | Alphaproteobacteria | Rhodobacterales | Rhodobacteraceae | Octadecabacter | unclassified |
| Otu149202 | 1 | 0.6667 | 0.816 | 0.01 | Bacteria | Cyanobacteria | Synechococcophycideae | Synechococcales | Synechococcaceae | Synechococcus | unclassified |
| Otu149206 | 0.7509 | 1 | 0.867 | 0.006 | Bacteria | Cyanobacteria | Synechococcophycideae | Synechococcales | Synechococcaceae | unclassified | unclassified |
| Otu149393 | 0.951 | 0.6667 | 0.796 | 0.015 | Bacteria | Proteobacteria | Gammaproteobacteria | Thiotrichales | Thiotrichaceae | Leucothrix | unclassified |
| Otu149902 | 1 | 0.6667 | 0.816 | 0.01 | Bacteria | Proteobacteria | Gammaproteobacteria | Pseudomonadales | Moraxellaceae | Psychrobacter | pacificensis |
| Otu150168 | 1 | 0.6667 | 0.816 | 0.007 | Bacteria | Proteobacteria | Gammaproteobacteria | Pseudomonadales | Moraxellaceae | Psychrobacter | pacificensis |
| Otu150206 | 1 | 0.6667 | 0.816 | 0.009 | Bacteria | Proteobacteria | Gammaproteobacteria | Pseudomonadales | Moraxellaceae | Psychrobacter | marincola |
| Otu152236 | 1 | 0.6667 | 0.816 | 0.007 | Bacteria | Proteobacteria | Gammaproteobacteria | Vibrionales | Vibrionaceae | Aliivibrio | fischeri |
| Otu152290 | 1 | 0.6667 | 0.816 | 0.01 | Bacteria | Proteobacteria | Gammaproteobacteria | Vibrionales | Vibrionaceae | unclassified | unclassified |
| Otu154346 | 0.8488 | 0.6667 | 0.752 | 0.037 | Bacteria | Proteobacteria | Gammaproteobacteria | Vibrionales | Vibrionaceae | Aliivibrio | fischeri |
| Otu155856 | 0.7141 | 1 | 0.845 | 0.036 | Bacteria | Proteobacteria | Alphaproteobacteria | Rhodospirillales | Rhodospirillaceae | unclassified | unclassified |
| Otu158011 | 0.9142 | 0.6667 | 0.781 | 0.033 | Bacteria | Proteobacteria | Gammaproteobacteria | Alteromonadales | unclassified | unclassified | unclassified |
| Otu158043 | 1 | 0.6667 | 0.816 | 0.01 | Bacteria | Proteobacteria | Gammaproteobacteria | Alteromonadales | Alteromonadaceae | Glaciecola | unclassified |
| Otu158056 | 0.9039 | 1 | 0.951 | 0.002 | Bacteria | Proteobacteria | Gammaproteobacteria | Alteromonadales | unclassified | unclassified | unclassified |
| Otu158592 | 0.9587 | 1 | 0.979 | 0.001 | Bacteria | Proteobacteria | Gammaproteobacteria | unclassified | unclassified | unclassified | unclassified |
| Otu159196 | 0.947 | 0.6667 | 0.795 | 0.018 | Bacteria | Proteobacteria | Gammaproteobacteria | Vibrionales | Pseudoalteromonadaceae | Pseudoalteromonas | unclassified |
| Otu159233 | 1 | 1 | 1 | 0.001 | Bacteria | Proteobacteria | Gammaproteobacteria | unclassified | unclassified | unclassified | unclassified |
| Otu159252 | 0.912 | 0.6667 | 0.78 | 0.025 | Bacteria | Proteobacteria | Gammaproteobacteria | Alteromonadales | Shewanellaceae | Shewanella | pacifica |
| Otu159392 | 1 | 0.6667 | 0.816 | 0.01 | Bacteria | Proteobacteria | Gammaproteobacteria | unclassified | unclassified | unclassified | unclassified |
| Otu159526 | 1 | 1 | 1 | 0.001 | Bacteria | Proteobacteria | Alphaproteobacteria | Rhodobacterales | unclassified | unclassified | unclassified |
| Otu159722 | 1 | 1 | 1 | 0.001 | Bacteria | Proteobacteria | Alphaproteobacteria | Rhodobacterales | Rhodobacteraceae | Octadecabacter | unclassified |
| Otu159888 | 0.9494 | 0.6667 | 0.796 | 0.01 | Bacteria | Bacteroidetes | Flavobacteriia | Flavobacteriales | unclassified | unclassified | unclassified |
| Otu159900 | 1 | 0.6667 | 0.816 | 0.009 | Bacteria | Bacteroidetes | Flavobacteriia | Flavobacteriales | unclassified | unclassified | unclassified |
| Otu159929 | 0.951 | 1 | 0.975 | 0.002 | Bacteria | Proteobacteria | Alphaproteobacteria | Rhodobacterales | Rhodobacteraceae | unclassified | unclassified |
| Otu160160 | 0.9624 | 1 | 0.981 | 0.001 | Bacteria | Proteobacteria | Alphaproteobacteria | Rhodobacterales | Rhodobacteraceae | unclassified | unclassified |
| Otu160308 | 0.964 | 1 | 0.982 | 0.001 | Bacteria | Proteobacteria | Gammaproteobacteria | Alteromonadales | Alteromonadaceae | unclassified | unclassified |
| Otu160429 | 0.9769 | 1 | 0.988 | 0.001 | Bacteria | Proteobacteria | Alphaproteobacteria | Rhodobacterales | Rhodobacteraceae | unclassified | unclassified |
| Otu160644 | 1 | 0.6667 | 0.816 | 0.01 | Bacteria | Proteobacteria | Alphaproteobacteria | Rhodobacterales | Rhodobacteraceae | Octadecabacter | unclassified |
| Otu160840 | 1 | 1 | 1 | 0.001 | Bacteria | Proteobacteria | Alphaproteobacteria | Rhodobacterales | Rhodobacteraceae | unclassified | unclassified |
| Otu160925 | 1 | 1 | 1 | 0.001 | Bacteria | Proteobacteria | Alphaproteobacteria | Rhodobacterales | Hyphomonadaceae | Hirschia | baltica |
| Otu160932 | 0.9632 | 1 | 0.981 | 0.001 | Bacteria | Proteobacteria | Alphaproteobacteria | Rhodobacterales | Rhodobacteraceae | unclassified | unclassified |
| Otu160972 | 0.9545 | 1 | 0.977 | 0.001 | Bacteria | Proteobacteria | Alphaproteobacteria | Rhodobacterales | unclassified | unclassified | unclassified |
| Otu161010 | 0.9719 | 1 | 0.986 | 0.001 | Bacteria | Proteobacteria | Alphaproteobacteria | Rhodobacterales | Rhodobacteraceae | unclassified | unclassified |
| Otu161062 | 1 | 0.6667 | 0.816 | 0.01 | Bacteria | Actinobacteria | Acidimicrobiia | Acidimicrobiales | OCS155 | unclassified | unclassified |
| Otu161076 | 1 | 1 | 1 | 0.001 | Bacteria | Actinobacteria | Acidimicrobiia | Acidimicrobiales | OCS155 | unclassified | unclassified |
| Otu161202 | 0.9005 | 0.6667 | 0.775 | 0.03 | Bacteria | Actinobacteria | Acidimicrobiia | Acidimicrobiales | OCS155 | unclassified | unclassified |
| Otu161257 | 0.9092 | 1 | 0.953 | 0.002 | Bacteria | Actinobacteria | Acidimicrobiia | Acidimicrobiales | OCS155 | unclassified | unclassified |
| Otu161309 | 1 | 1 | 1 | 0.001 | Bacteria | Actinobacteria | Acidimicrobiia | Acidimicrobiales | OCS155 | unclassified | unclassified |
| Otu161314 | 1 | 1 | 1 | 0.001 | Bacteria | Actinobacteria | Acidimicrobiia | Acidimicrobiales | OCS155 | unclassified | unclassified |
| Otu161315 | 0.9428 | 1 | 0.971 | 0.002 | Bacteria | Actinobacteria | Acidimicrobiia | Acidimicrobiales | OCS155 | unclassified | unclassified |
| Otu161669 | 0.9471 | 0.6667 | 0.795 | 0.021 | Bacteria | Actinobacteria | Acidimicrobiia | Acidimicrobiales | OCS155 | unclassified | unclassified |
| Otu161747 | 0.9603 | 0.6667 | 0.8 | 0.015 | Bacteria | Actinobacteria | Acidimicrobiia | Acidimicrobiales | OCS155 | unclassified | unclassified |
| Otu161905 | 1 | 0.6667 | 0.816 | 0.01 | Bacteria | Actinobacteria | Acidimicrobiia | Acidimicrobiales | OCS155 | unclassified | unclassified |
| Otu161914 | 1 | 1 | 1 | 0.001 | Bacteria | Actinobacteria | Acidimicrobiia | Acidimicrobiales | OCS155 | unclassified | unclassified |
| Otu161946 | 1 | 1 | 1 | 0.001 | Bacteria | Actinobacteria | Acidimicrobiia | Acidimicrobiales | OCS155 | unclassified | unclassified |
| Otu162329 | 1 | 0.6667 | 0.816 | 0.007 | Bacteria | Actinobacteria | Acidimicrobiia | Acidimicrobiales | OCS155 | unclassified | unclassified |
| Otu162343 | 1 | 1 | 1 | 0.001 | Bacteria | Actinobacteria | Acidimicrobiia | Acidimicrobiales | OCS155 | unclassified | unclassified |
| Otu162490 | 1 | 0.6667 | 0.816 | 0.007 | Bacteria | Actinobacteria | Acidimicrobiia | Acidimicrobiales | OCS155 | unclassified | unclassified |
| Otu162517 | 1 | 1 | 1 | 0.001 | Bacteria | Actinobacteria | Acidimicrobiia | Acidimicrobiales | OCS155 | unclassified | unclassified |
| Otu163322 | 0.8845 | 0.6667 | 0.768 | 0.025 | Bacteria | Proteobacteria | Gammaproteobacteria | HTCC2188 | HTCC2089 | unclassified | unclassified |
| Otu172128 | 1 | 0.6667 | 0.816 | 0.009 | Bacteria | Proteobacteria | unclassified | unclassified | unclassified | unclassified | unclassified |
| Otu174651 | 1 | 0.6667 | 0.816 | 0.01 | Bacteria | Proteobacteria | Gammaproteobacteria | unclassified | unclassified | unclassified | unclassified |
| Otu174994 | 1 | 0.6667 | 0.816 | 0.01 | Bacteria | Proteobacteria | Gammaproteobacteria | Alteromonadales | unclassified | unclassified | unclassified |
| Otu175064 | 1 | 1 | 1 | 0.001 | Bacteria | Proteobacteria | Gammaproteobacteria | unclassified | unclassified | unclassified | unclassified |
| Otu175133 | 1 | 0.6667 | 0.816 | 0.007 | Bacteria | Proteobacteria | Gammaproteobacteria | Oceanospirillales | Halomonadaceae | Candidatus_Portiera | unclassified |
| Otu175253 | 0.9262 | 0.6667 | 0.786 | 0.023 | Bacteria | Proteobacteria | Gammaproteobacteria | Oceanospirillales | Halomonadaceae | Candidatus_Portiera | unclassified |
| Otu175484 | 0.919 | 0.6667 | 0.783 | 0.022 | Bacteria | Proteobacteria | Gammaproteobacteria | unclassified | unclassified | unclassified | unclassified |
| Otu175737 | 0.9005 | 0.6667 | 0.775 | 0.03 | Bacteria | Proteobacteria | Gammaproteobacteria | Oceanospirillales | unclassified | unclassified | unclassified |
| Otu175936 | 1 | 1 | 1 | 0.001 | Bacteria | Cyanobacteria | Synechococcophycideae | Synechococcales | Synechococcaceae | unclassified | unclassified |
| Otu175966 | 0.7091 | 1 | 0.842 | 0.028 | Bacteria | Cyanobacteria | Synechococcophycideae | Synechococcales | Synechococcaceae | Paulinella | unclassified |
| Otu176232 | 1 | 0.6667 | 0.816 | 0.007 | Bacteria | Cyanobacteria | Synechococcophycideae | Synechococcales | Synechococcaceae | unclassified | unclassified |
| Otu176240 | 0.8412 | 1 | 0.917 | 0.007 | Bacteria | Cyanobacteria | Synechococcophycideae | Synechococcales | Synechococcaceae | Synechococcus | unclassified |

| | | | | | | | | | | | |
|---|---|---|---|---|---|---|---|---|---|---|---|
| Otu176277 | 0.7396 | 1 | 0.86 | 0.022 | Bacteria | Cyanobacteria | Synechococcophycideae | Synechococcales | Synechococcaceae | Synechococcus | unclassified |
| Otu176476 | 0.9276 | 1 | 0.963 | 0.001 | Bacteria | Cyanobacteria | Synechococcophycideae | Synechococcales | Synechococcaceae | Synechococcus | unclassified |
| Otu176686 | 0.9073 | 0.6667 | 0.778 | 0.016 | Bacteria | Proteobacteria | Gammaproteobacteria | Oceanospirillales | Oceanospirillaceae | Marinomonas | primoryensis |
| Otu176754 | 1 | 0.6667 | 0.816 | 0.007 | Bacteria | Proteobacteria | Gammaproteobacteria | Alteromonadales | HTCC2188 | HTCC | unclassified |
| Otu177271 | 0.9398 | 0.6667 | 0.792 | 0.028 | Bacteria | Proteobacteria | Alphaproteobacteria | Rhodobacterales | unclassified | unclassified | unclassified |
| Otu178224 | 1 | 1 | 1 | 0.001 | Bacteria | Proteobacteria | Alphaproteobacteria | Rhodobacterales | Rhodobacteraceae | Octadecabacter | unclassified |
| Otu178252 | 1 | 1 | 1 | 0.001 | Bacteria | Proteobacteria | Alphaproteobacteria | Rhodobacterales | Rhodobacteraceae | Octadecabacter | unclassified |
| Otu178524 | 1 | 0.6667 | 0.816 | 0.01 | Bacteria | Proteobacteria | Gammaproteobacteria | unclassified | unclassified | unclassified | unclassified |
| Otu178557 | 0.9582 | 0.6667 | 0.799 | 0.012 | Bacteria | Proteobacteria | Gammaproteobacteria | Oceanospirillales | Halomonadaceae | Candidatus_Portiera | unclassified |
| Otu178739 | 1 | 1 | 1 | 0.001 | Bacteria | Proteobacteria | Gammaproteobacteria | unclassified | unclassified | unclassified | unclassified |
| Otu178954 | 0.8815 | 1 | 0.939 | 0.003 | Bacteria | Proteobacteria | Gammaproteobacteria | Oceanospirillales | Halomonadaceae | Cobetia | unclassified |
| Otu178987 | 1 | 0.6667 | 0.816 | 0.01 | Bacteria | Proteobacteria | Gammaproteobacteria | Oceanospirillales | Halomonadaceae | Candidatus_Portiera | unclassified |
| Otu179340 | 1 | 0.6667 | 0.816 | 0.007 | Bacteria | Proteobacteria | Alphaproteobacteria | Rhodobacterales | unclassified | unclassified | unclassified |
| Otu182832 | 1 | 0.6667 | 0.816 | 0.01 | Bacteria | Proteobacteria | Gammaproteobacteria | unclassified | unclassified | unclassified | unclassified |
| Otu183266 | 0.8877 | 1 | 0.942 | 0.003 | Bacteria | Cyanobacteria | Synechococcophycideae | Synechococcales | Synechococcaceae | Synechococcus | unclassified |
| Otu183356 | 0.911 | 1 | 0.954 | 0.001 | Bacteria | Cyanobacteria | Synechococcophycideae | Synechococcales | Synechococcaceae | Synechococcus | unclassified |
| Otu183527 | 0.9029 | 1 | 0.95 | 0.003 | Bacteria | Cyanobacteria | Synechococcophycideae | Synechococcales | Synechococcaceae | unclassified | unclassified |
| Otu183572 | 0.8191 | 0.6667 | 0.739 | 0.037 | Bacteria | Cyanobacteria | Synechococcophycideae | Synechococcales | Synechococcaceae | Synechococcus | unclassified |
| Otu183596 | 0.7927 | 1 | 0.89 | 0.007 | Bacteria | Cyanobacteria | Synechococcophycideae | Synechococcales | Synechococcaceae | Synechococcus | unclassified |
| Otu185557 | 0.8807 | 1 | 0.938 | 0.002 | Bacteria | Bacteroidetes | Flavobacteriia | Flavobacteriales | Flavobacteriaceae | unclassified | unclassified |
| Otu185641 | 1 | 0.6667 | 0.816 | 0.007 | Bacteria | Bacteroidetes | Flavobacteriia | Flavobacteriales | Cryomorphaceae | unclassified | unclassified |
| Otu185919 | 1 | 0.6667 | 0.816 | 0.009 | Bacteria | Bacteroidetes | unclassified | unclassified | unclassified | unclassified | unclassified |
| Otu186294 | 1 | 0.6667 | 0.816 | 0.007 | Bacteria | GN02 | BD1-5 | unclassified | unclassified | unclassified | unclassified |
| Otu187123 | 0.9171 | 0.6667 | 0.782 | 0.028 | Bacteria | Bacteroidetes | Flavobacteriia | Flavobacteriales | Flavobacteriaceae | unclassified | unclassified |
| Otu187136 | 1 | 0.6667 | 0.816 | 0.01 | Bacteria | Bacteroidetes | Flavobacteriia | Flavobacteriales | Flavobacteriaceae | unclassified | unclassified |
| Otu195571 | 1 | 0.6667 | 0.816 | 0.01 | Bacteria | Bacteroidetes | Flavobacteriia | Flavobacteriales | Flavobacteriaceae | unclassified | unclassified |
| Otu195697 | 0.9066 | 0.6667 | 0.777 | 0.025 | Bacteria | Bacteroidetes | Flavobacteriia | Flavobacteriales | Cryomorphaceae | Fluviicola | unclassified |
| Otu200944 | 1 | 1 | 1 | 0.001 | Bacteria | Proteobacteria | Gammaproteobacteria | unclassified | unclassified | unclassified | unclassified |
| Otu206949 | 0.8613 | 1 | 0.928 | 0.007 | Bacteria | Bacteroidetes | Flavobacteriia | Flavobacteriales | Flavobacteriaceae | unclassified | unclassified |
| Otu207167 | 0.9679 | 1 | 0.984 | 0.001 | Bacteria | Bacteroidetes | Flavobacteriia | Flavobacteriales | Cryomorphaceae | Fluviicola | unclassified |
| Otu211339 | 0.9391 | 0.6667 | 0.791 | 0.026 | Bacteria | Bacteroidetes | Flavobacteriia | Flavobacteriales | unclassified | unclassified | unclassified |
| Otu211355 | 0.827 | 1 | 0.909 | 0.003 | Bacteria | Bacteroidetes | Flavobacteriia | Flavobacteriales | Flavobacteriaceae | unclassified | unclassified |
| Otu211757 | 0.8633 | 1 | 0.929 | 0.005 | Bacteria | Bacteroidetes | Flavobacteriia | Flavobacteriales | Flavobacteriaceae | unclassified | unclassified |
| Otu212317 | 0.9291 | 0.6667 | 0.787 | 0.026 | Bacteria | Proteobacteria | Alphaproteobacteria | Rhodobacterales | Hyphomonadaceae | Hirschia | baltica |
| Otu212714 | 0.978 | 1 | 0.989 | 0.001 | Bacteria | Bacteroidetes | Flavobacteriia | Flavobacteriales | Flavobacteriaceae | unclassified | unclassified |
| Otu213331 | 0.9488 | 0.6667 | 0.795 | 0.019 | Bacteria | Proteobacteria | Gammaproteobacteria | unclassified | unclassified | unclassified | unclassified |
| Otu215545 | 0.9501 | 1 | 0.975 | 0.001 | Bacteria | Proteobacteria | Gammaproteobacteria | unclassified | unclassified | unclassified | unclassified |
| Otu215692 | 1 | 0.6667 | 0.816 | 0.007 | Bacteria | Proteobacteria | Gammaproteobacteria | unclassified | unclassified | unclassified | unclassified |
| Otu217137 | 0.934 | 1 | 0.966 | 0.003 | Bacteria | Proteobacteria | Gammaproteobacteria | Alteromonadales | unclassified | unclassified | unclassified |
| Otu226675 | 1 | 1 | 1 | 0.001 | Bacteria | Proteobacteria | Alphaproteobacteria | Rhodobacterales | unclassified | unclassified | unclassified |
| Otu227006 | 0.9209 | 1 | 0.96 | 0.001 | Bacteria | Proteobacteria | unclassified | unclassified | unclassified | unclassified | unclassified |
| Otu227573 | 1 | 0.6667 | 0.816 | 0.01 | Bacteria | Proteobacteria | unclassified | unclassified | unclassified | unclassified | unclassified |
| Otu227591 | 0.9417 | 0.6667 | 0.792 | 0.007 | Bacteria | unclassified | unclassified | unclassified | unclassified | unclassified | unclassified |
| Otu227982 | 1 | 1 | 1 | 0.001 | Bacteria | unclassified | unclassified | unclassified | unclassified | unclassified | unclassified |
| Otu228243 | 0.8576 | 0.6667 | 0.756 | 0.023 | Bacteria | Proteobacteria | Alphaproteobacteria | Rickettsiales | Rickettsiaceae | unclassified | unclassified |
| Otu232100 | 0.902 | 1 | 0.95 | 0.002 | Bacteria | Planctomycetes | Planctomycetia | Planctomycetales | Planctomycetaceae | Planctomyces | unclassified |
| Otu232871 | 0.9413 | 0.6667 | 0.792 | 0.015 | Bacteria | Proteobacteria | Alphaproteobacteria | Rhodobacterales | unclassified | unclassified | unclassified |
| Otu240643 | 1 | 1 | 1 | 0.001 | Bacteria | Proteobacteria | Gammaproteobacteria | unclassified | unclassified | unclassified | unclassified |
| Otu241506 | 1 | 1 | 1 | 0.001 | Bacteria | Proteobacteria | Gammaproteobacteria | unclassified | unclassified | unclassified | unclassified |
| Otu243787 | 0.9255 | 0.6667 | 0.785 | 0.03 | Bacteria | Proteobacteria | Gammaproteobacteria | unclassified | unclassified | unclassified | unclassified |
| Otu244233 | 0.941 | 0.6667 | 0.792 | 0.015 | Bacteria | Proteobacteria | Betaproteobacteria | unclassified | unclassified | unclassified | unclassified |
| Otu251151 | 0.8725 | 0.6667 | 0.763 | 0.041 | Bacteria | Proteobacteria | Gammaproteobacteria | unclassified | unclassified | unclassified | unclassified |
| Otu278236 | 0.9059 | 0.6667 | 0.777 | 0.025 | Bacteria | Bacteroidetes | Saprospirae | Saprospirales | Saprospiraceae | unclassified | unclassified |
| Otu289795 | 1 | 0.6667 | 0.816 | 0.007 | Archaea | Euryarchaeota | Thermoplasmata | E2 | Marine_group_II | unclassified | unclassified |
| Otu306924 | 1 | 0.6667 | 0.816 | 0.007 | Bacteria | Actinobacteria | Acidimicrobiia | Acidimicrobiales | OCS155 | unclassified | unclassified |
| Otu349126 | 0.9396 | 1 | 0.969 | 0.002 | Bacteria | Bacteroidetes | unclassified | unclassified | unclassified | unclassified | unclassified |

**Suppl. Table 3** Summary of the indicator species (i.e., OTUs) analysis performed by the function multipatt only for the two *A. infundibuliformis* groups. Shown are 'Rest' and 'Stat' as values of the statistic for selecting the indicator OTUs. Only OTUs with significant *p* values $\leq$ 0.05 are summarized. In addition for each OTU, Greengenes classification results are added from phylum to species level if available.

| Otus | *Axinella infundibuliformis* shallow | Rest | stat | p value | Domain | Phylum | Class | Order | Family | Genus | Species |
|---|---|---|---|---|---|---|---|---|---|---|---|
| Otu094777 | 0.8108 | 1 | 0.9 | 0.004 | Archaea | Crenarchaeota | Thaumarchaeota | Cenarchaeales | Cenarchaeaceae | Nitrosopumilus | Nitrosopumilus unclassified |
| Otu094642 | 0.8062 | 0.6667 | 0.733 | 0.031 | Archaea | Crenarchaeota | Thaumarchaeota | Cenarchaeales | Cenarchaeaceae | Nitrosopumilus | Nitrosopumilus unclassified |
| Otu093768 | 0.9093 | 1 | 0.954 | 0.001 | Archaea | Crenarchaeota | Thaumarchaeota | Cenarchaeales | Cenarchaeaceae | Nitrosopumilus | Nitrosopumilus unclassified |
| Otu068072 | 0.7835 | 1 | 0.885 | 0.01 | Archaea | Crenarchaeota | Thaumarchaeota | Cenarchaeales | Cenarchaeaceae | Nitrosopumilus | Nitrosopumilus unclassified |
| Otu062572 | 0.95 | 0.6667 | 0.796 | 0.012 | Archaea | Crenarchaeota | Thaumarchaeota | Cenarchaeales | Cenarchaeaceae | Nitrosopumilus | Nitrosopumilus unclassified |
| Otu048942 | 0.8702 | 1 | 0.933 | 0.002 | Archaea | Crenarchaeota | Thaumarchaeota | Cenarchaeales | Cenarchaeaceae | Nitrosopumilus | Nitrosopumilus unclassified |
| Otu047355 | 0.7763 | 1 | 0.881 | 0.012 | Archaea | Crenarchaeota | Thaumarchaeota | Cenarchaeales | Cenarchaeaceae | Nitrosopumilus | Nitrosopumilus unclassified |
| Otu047308 | 0.827 | 0.6667 | 0.743 | 0.023 | Archaea | Crenarchaeota | Thaumarchaeota | Cenarchaeales | Cenarchaeaceae | Nitrosopumilus | Nitrosopumilus unclassified |
| Otu046395 | 0.7963 | 0.6667 | 0.729 | 0.037 | Archaea | Crenarchaeota | Thaumarchaeota | Cenarchaeales | Cenarchaeaceae | Nitrosopumilus | Nitrosopumilus unclassified |
| Otu046372 | 0.8134 | 0.6667 | 0.736 | 0.049 | Archaea | Crenarchaeota | Thaumarchaeota | Cenarchaeales | Cenarchaeaceae | Nitrosopumilus | Nitrosopumilus unclassified |
| Otu043432 | 0.856 | 1 | 0.925 | 0.003 | Archaea | Crenarchaeota | Thaumarchaeota | Cenarchaeales | Cenarchaeaceae | Nitrosopumilus | Nitrosopumilus unclassified |
| Otu040608 | 0.8487 | 1 | 0.921 | 0.003 | Archaea | Crenarchaeota | Thaumarchaeota | Cenarchaeales | Cenarchaeaceae | Nitrosopumilus | Nitrosopumilus unclassified |
| Otu035981 | 0.8268 | 0.6667 | 0.742 | 0.039 | Archaea | Crenarchaeota | Thaumarchaeota | Cenarchaeales | Cenarchaeaceae | Nitrosopumilus | Nitrosopumilus unclassified |
| Otu035838 | 0.8248 | 0.6667 | 0.742 | 0.033 | Archaea | Crenarchaeota | Thaumarchaeota | Cenarchaeales | Cenarchaeaceae | Nitrosopumilus | Nitrosopumilus unclassified |
| Otu031597 | 0.7991 | 1 | 0.894 | 0.007 | Archaea | Crenarchaeota | Thaumarchaeota | Cenarchaeales | Cenarchaeaceae | Nitrosopumilus | Nitrosopumilus unclassified |
| Otu030901 | 0.7231 | 0.6667 | 0.694 | 0.046 | Archaea | Crenarchaeota | Thaumarchaeota | Cenarchaeales | Cenarchaeaceae | Nitrosopumilus | Nitrosopumilus unclassified |
| Otu028848 | 0.8691 | 1 | 0.932 | 0.001 | Archaea | Crenarchaeota | Thaumarchaeota | Cenarchaeales | Cenarchaeaceae | Nitrosopumilus | Nitrosopumilus unclassified |
| Otu028358 | 0.8909 | 1 | 0.944 | 0.001 | Archaea | Crenarchaeota | Thaumarchaeota | Cenarchaeales | Cenarchaeaceae | Nitrosopumilus | Nitrosopumilus unclassified |
| Otu027941 | 0.8059 | 0.6667 | 0.733 | 0.015 | Archaea | Crenarchaeota | Thaumarchaeota | Cenarchaeales | Cenarchaeaceae | Nitrosopumilus | Nitrosopumilus unclassified |
| Otu027885 | 0.8218 | 0.6667 | 0.74 | 0.03 | Archaea | Crenarchaeota | Thaumarchaeota | Cenarchaeales | Cenarchaeaceae | Nitrosopumilus | Nitrosopumilus unclassified |
| Otu025148 | 0.8945 | 1 | 0.946 | 0.003 | Archaea | Crenarchaeota | Thaumarchaeota | Cenarchaeales | Cenarchaeaceae | Nitrosopumilus | Nitrosopumilus unclassified |
| Otu025067 | 0.7965 | 1 | 0.892 | 0.007 | Archaea | Crenarchaeota | Thaumarchaeota | Cenarchaeales | Cenarchaeaceae | Nitrosopumilus | Nitrosopumilus unclassified |
| Otu024937 | 0.8177 | 1 | 0.904 | 0.003 | Archaea | Crenarchaeota | Thaumarchaeota | Cenarchaeales | Cenarchaeaceae | Nitrosopumilus | Nitrosopumilus unclassified |
| Otu024771 | 0.817 | 1 | 0.904 | 0.004 | Archaea | Crenarchaeota | Thaumarchaeota | Cenarchaeales | Cenarchaeaceae | Nitrosopumilus | Nitrosopumilus unclassified |
| Otu022894 | 0.7939 | 1 | 0.891 | 0.012 | Archaea | Crenarchaeota | Thaumarchaeota | Cenarchaeales | Cenarchaeaceae | Nitrosopumilus | Nitrosopumilus unclassified |
| Otu022881 | 0.7771 | 1 | 0.882 | 0.014 | Archaea | Crenarchaeota | Thaumarchaeota | Cenarchaeales | Cenarchaeaceae | Nitrosopumilus | Nitrosopumilus unclassified |
| Otu021858 | 0.8241 | 1 | 0.908 | 0.005 | Archaea | Crenarchaeota | Thaumarchaeota | Cenarchaeales | Cenarchaeaceae | Nitrosopumilus | Nitrosopumilus unclassified |
| Otu021287 | 0.7886 | 1 | 0.888 | 0.004 | Archaea | Crenarchaeota | Thaumarchaeota | Cenarchaeales | Cenarchaeaceae | Nitrosopumilus | Nitrosopumilus unclassified |
| Otu019487 | 0.7675 | 1 | 0.876 | 0.01 | Archaea | Crenarchaeota | Thaumarchaeota | Cenarchaeales | Cenarchaeaceae | Nitrosopumilus | Nitrosopumilus unclassified |
| Otu019405 | 0.8313 | 1 | 0.912 | 0.001 | Archaea | Crenarchaeota | Thaumarchaeota | Cenarchaeales | Cenarchaeaceae | Nitrosopumilus | Nitrosopumilus unclassified |
| Otu019258 | 0.8191 | 1 | 0.905 | 0.007 | Archaea | Crenarchaeota | Thaumarchaeota | Cenarchaeales | Cenarchaeaceae | Nitrosopumilus | Nitrosopumilus unclassified |
| Otu019233 | 0.8573 | 1 | 0.926 | 0.001 | Archaea | Crenarchaeota | Thaumarchaeota | Cenarchaeales | Cenarchaeaceae | Nitrosopumilus | Nitrosopumilus unclassified |
| Otu019072 | 0.8679 | 1 | 0.932 | 0.001 | Archaea | Crenarchaeota | Thaumarchaeota | Cenarchaeales | Cenarchaeaceae | Nitrosopumilus | Nitrosopumilus unclassified |
| Otu019019 | 0.8519 | 1 | 0.923 | 0.002 | Archaea | Crenarchaeota | Thaumarchaeota | Cenarchaeales | Cenarchaeaceae | Nitrosopumilus | Nitrosopumilus unclassified |
| Otu019011 | 0.816 | 1 | 0.903 | 0.007 | Archaea | Crenarchaeota | Thaumarchaeota | Cenarchaeales | Cenarchaeaceae | Nitrosopumilus | Nitrosopumilus unclassified |
| Otu019001 | 0.8118 | 1 | 0.901 | 0.004 | Archaea | Crenarchaeota | Thaumarchaeota | Cenarchaeales | Cenarchaeaceae | Nitrosopumilus | Nitrosopumilus unclassified |
| Otu018973 | 0.8646 | 1 | 0.93 | 0.002 | Archaea | Crenarchaeota | Thaumarchaeota | Cenarchaeales | Cenarchaeaceae | Nitrosopumilus | Nitrosopumilus unclassified |
| Otu017749 | 0.8475 | 0.6667 | 0.752 | 0.01 | Archaea | Crenarchaeota | Thaumarchaeota | Cenarchaeales | Cenarchaeaceae | Nitrosopumilus | Nitrosopumilus unclassified |
| Otu016249 | 0.8256 | 1 | 0.909 | 0.004 | Archaea | Crenarchaeota | Thaumarchaeota | Cenarchaeales | Cenarchaeaceae | Nitrosopumilus | Nitrosopumilus unclassified |
| Otu016238 | 0.8225 | 1 | 0.907 | 0.004 | Archaea | Crenarchaeota | Thaumarchaeota | Cenarchaeales | Cenarchaeaceae | Nitrosopumilus | Nitrosopumilus unclassified |
| Otu015681 | 0.7765 | 1 | 0.881 | 0.01 | Archaea | Crenarchaeota | Thaumarchaeota | Cenarchaeales | Cenarchaeaceae | Nitrosopumilus | Nitrosopumilus unclassified |
| Otu015370 | 0.8063 | 1 | 0.898 | 0.004 | Archaea | Crenarchaeota | Thaumarchaeota | Cenarchaeales | Cenarchaeaceae | Nitrosopumilus | Nitrosopumilus unclassified |
| Otu015239 | 0.7829 | 1 | 0.885 | 0.012 | Archaea | Crenarchaeota | Thaumarchaeota | Cenarchaeales | Cenarchaeaceae | Nitrosopumilus | Nitrosopumilus unclassified |
| Otu015221 | 0.7929 | 1 | 0.89 | 0.014 | Archaea | Crenarchaeota | Thaumarchaeota | Cenarchaeales | Cenarchaeaceae | Nitrosopumilus | Nitrosopumilus unclassified |
| Otu015057 | 0.8189 | 0.6667 | 0.739 | 0.043 | Archaea | Crenarchaeota | Thaumarchaeota | Cenarchaeales | Cenarchaeaceae | Nitrosopumilus | Nitrosopumilus unclassified |
| Otu014951 | 0.7576 | 1 | 0.87 | 0.018 | Archaea | Crenarchaeota | Thaumarchaeota | Cenarchaeales | Cenarchaeaceae | Nitrosopumilus | Nitrosopumilus unclassified |
| Otu014833 | 0.8074 | 1 | 0.899 | 0.008 | Archaea | Crenarchaeota | Thaumarchaeota | Cenarchaeales | Cenarchaeaceae | Nitrosopumilus | Nitrosopumilus unclassified |
| Otu014643 | 0.9035 | 0.6667 | 0.776 | 0.022 | Archaea | Crenarchaeota | Thaumarchaeota | Cenarchaeales | Cenarchaeaceae | Nitrosopumilus | Nitrosopumilus unclassified |
| Otu014589 | 0.8376 | 1 | 0.915 | 0.002 | Archaea | Crenarchaeota | Thaumarchaeota | Cenarchaeales | Cenarchaeaceae | Nitrosopumilus | Nitrosopumilus unclassified |
| Otu013849 | 0.8461 | 1 | 0.92 | 0.005 | Archaea | Crenarchaeota | Thaumarchaeota | Cenarchaeales | Cenarchaeaceae | Nitrosopumilus | Nitrosopumilus unclassified |
| Otu013658 | 0.8417 | 1 | 0.917 | 0.004 | Archaea | Crenarchaeota | Thaumarchaeota | Cenarchaeales | Cenarchaeaceae | Nitrosopumilus | Nitrosopumilus unclassified |
| Otu012751 | 0.8513 | 0.6667 | 0.753 | 0.018 | Archaea | Crenarchaeota | Thaumarchaeota | Cenarchaeales | Cenarchaeaceae | Nitrosopumilus | Nitrosopumilus unclassified |
| Otu012362 | 0.797 | 1 | 0.893 | 0.002 | Archaea | Crenarchaeota | Thaumarchaeota | Cenarchaeales | Cenarchaeaceae | Nitrosopumilus | Nitrosopumilus unclassified |
| Otu012309 | 0.8138 | 1 | 0.902 | 0.006 | Archaea | Crenarchaeota | Thaumarchaeota | Cenarchaeales | Cenarchaeaceae | Nitrosopumilus | Nitrosopumilus unclassified |
| Otu011440 | 0.7896 | 1 | 0.889 | 0.002 | Archaea | Crenarchaeota | Thaumarchaeota | Cenarchaeales | Cenarchaeaceae | Nitrosopumilus | Nitrosopumilus unclassified |
| Otu010379 | 0.8534 | 1 | 0.924 | 0.002 | Archaea | Crenarchaeota | Thaumarchaeota | Cenarchaeales | Cenarchaeaceae | Nitrosopumilus | Nitrosopumilus unclassified |
| Otu010311 | 0.7288 | 1 | 0.854 | 0.026 | Archaea | Crenarchaeota | Thaumarchaeota | Cenarchaeales | Cenarchaeaceae | Nitrosopumilus | Nitrosopumilus unclassified |
| Otu009854 | 0.8219 | 1 | 0.907 | 0.003 | Archaea | Crenarchaeota | Thaumarchaeota | Cenarchaeales | Cenarchaeaceae | Nitrosopumilus | Nitrosopumilus unclassified |
| Otu009849 | 0.8045 | 1 | 0.897 | 0.007 | Archaea | Crenarchaeota | Thaumarchaeota | Cenarchaeales | Cenarchaeaceae | Nitrosopumilus | Nitrosopumilus unclassified |
| Otu009847 | 0.7861 | 0.6667 | 0.724 | 0.04 | Archaea | Crenarchaeota | Thaumarchaeota | Cenarchaeales | Cenarchaeaceae | Nitrosopumilus | Nitrosopumilus unclassified |
| Otu009768 | 0.8174 | 1 | 0.904 | 0.004 | Archaea | Crenarchaeota | Thaumarchaeota | Cenarchaeales | Cenarchaeaceae | Nitrosopumilus | Nitrosopumilus unclassified |
| Otu009086 | 0.8024 | 1 | 0.896 | 0.002 | Archaea | Crenarchaeota | Thaumarchaeota | Cenarchaeales | Cenarchaeaceae | Nitrosopumilus | Nitrosopumilus unclassified |
| Otu009079 | 0.7979 | 1 | 0.893 | 0.012 | Archaea | Crenarchaeota | Thaumarchaeota | Cenarchaeales | Cenarchaeaceae | Nitrosopumilus | Nitrosopumilus unclassified |
| Otu008885 | 0.9334 | 0.6667 | 0.789 | 0.01 | Archaea | Crenarchaeota | Thaumarchaeota | Cenarchaeales | Cenarchaeaceae | Nitrosopumilus | Nitrosopumilus unclassified |
| Otu008208 | 0.7997 | 1 | 0.894 | 0.004 | Archaea | Crenarchaeota | Thaumarchaeota | Cenarchaeales | Cenarchaeaceae | Nitrosopumilus | Nitrosopumilus unclassified |
| Otu008196 | 0.7949 | 1 | 0.892 | 0.008 | Archaea | Crenarchaeota | Thaumarchaeota | Cenarchaeales | Cenarchaeaceae | Nitrosopumilus | Nitrosopumilus unclassified |
| Otu007911 | 0.8225 | 1 | 0.907 | 0.002 | Archaea | Crenarchaeota | Thaumarchaeota | Cenarchaeales | Cenarchaeaceae | Nitrosopumilus | Nitrosopumilus unclassified |
| Otu007904 | 0.882 | 0.6667 | 0.767 | 0.009 | Archaea | Crenarchaeota | Thaumarchaeota | Cenarchaeales | Cenarchaeaceae | Nitrosopumilus | Nitrosopumilus unclassified |
| Otu007001 | 0.772 | 1 | 0.879 | 0.014 | Archaea | Crenarchaeota | Thaumarchaeota | Cenarchaeales | Cenarchaeaceae | Nitrosopumilus | Nitrosopumilus unclassified |
| Otu006886 | 0.8072 | 1 | 0.898 | 0.003 | Archaea | Crenarchaeota | Thaumarchaeota | Cenarchaeales | Cenarchaeaceae | Nitrosopumilus | Nitrosopumilus unclassified |
| Otu006316 | 0.8352 | 1 | 0.914 | 0.001 | Archaea | Crenarchaeota | Thaumarchaeota | Cenarchaeales | Cenarchaeaceae | Nitrosopumilus | Nitrosopumilus unclassified |
| Otu005169 | 0.794 | 1 | 0.891 | 0.006 | Archaea | Crenarchaeota | Thaumarchaeota | Cenarchaeales | Cenarchaeaceae | Nitrosopumilus | Nitrosopumilus unclassified |
| Otu005164 | 0.787 | 0.6667 | 0.724 | 0.016 | Archaea | Crenarchaeota | Thaumarchaeota | Cenarchaeales | Cenarchaeaceae | Nitrosopumilus | Nitrosopumilus unclassified |
| Otu003602 | 0.8006 | 1 | 0.895 | 0.005 | Archaea | Crenarchaeota | Thaumarchaeota | Cenarchaeales | Cenarchaeaceae | Nitrosopumilus | Nitrosopumilus unclassified |
| Otu003584 | 0.7886 | 1 | 0.888 | 0.004 | Archaea | Crenarchaeota | Thaumarchaeota | Cenarchaeales | Cenarchaeaceae | Nitrosopumilus | Nitrosopumilus unclassified |
| Otu003072 | 0.6247 | 1 | 0.79 | 0.037 | Archaea | Crenarchaeota | Thaumarchaeota | Cenarchaeales | Cenarchaeaceae | Nitrosopumilus | Nitrosopumilus unclassified |
| Otu001422 | 0.8898 | 1 | 0.943 | 0.003 | Archaea | Crenarchaeota | Thaumarchaeota | Cenarchaeales | Cenarchaeaceae | Nitrosopumilus | Nitrosopumilus unclassified |
| Otu000729 | 0.8068 | 1 | 0.898 | 0.006 | Archaea | Crenarchaeota | Thaumarchaeota | Cenarchaeales | Cenarchaeaceae | Nitrosopumilus | Nitrosopumilus unclassified |
| Otu019322 | 0.877 | 1 | 0.936 | 0.003 | Bacteria | Actinobacteria | Acidimicrobiia | Acidimicrobiales | C111 | C111 unclassified | unclassified |
| Otu008050 | 0.8701 | 1 | 0.933 | 0.001 | Bacteria | Actinobacteria | Acidimicrobiia | Acidimicrobiales | C111 | C111 unclassified | unclassified |
| Otu161257 | 0.8051 | 0.6667 | 0.733 | 0.05 | Bacteria | Actinobacteria | Acidimicrobiia | Acidimicrobiales | OCS155 | OCS155 unclassified | unclassified |
| Otu084559 | 0.8699 | 1 | 0.933 | 0.001 | Bacteria | Actinobacteria | Acidimicrobiia | Acidimicrobiales | SC3-41 | SC3-41 unclassified | unclassified |
| Otu037843 | 0.8949 | 1 | 0.946 | 0.001 | Bacteria | Actinobacteria | Acidimicrobiia | Acidimicrobiales | SC3-41 | SC3-41 unclassified | unclassified |

| | | | | | | | | | | | |
|---|---|---|---|---|---|---|---|---|---|---|---|
| Otu001374 | 0.9229 | 1 | 0.961 | 0.002 | Bacteria | Actinobacteria | Acidimicrobiia | Acidimicrobiales | TK06 | TK06 unclassified | unclassified |
| Otu008008 | 0.7211 | 1 | 0.849 | 0.033 | Bacteria | Actinobacteria | Actinobacteria | Actinobacteria unclassified | unclassified | unclassified | unclassified |
| Otu002305 | 0.7899 | 1 | 0.889 | 0.027 | Bacteria | Actinobacteria | Actinobacteria | Actinomycetales | Actinomycetales unclassified | unclassified | unclassified |
| Otu227930 | 0.9311 | 0.6667 | 0.788 | 0.006 | Bacteria | Bacteria unclassified | unclassified | unclassified | unclassified | unclassified | unclassified |
| Otu103149 | 0.8546 | 0.6667 | 0.755 | 0.022 | Bacteria | Bacteria unclassified | unclassified | unclassified | unclassified | unclassified | unclassified |
| Otu100239 | 0.8745 | 0.6667 | 0.764 | 0.012 | Bacteria | Bacteria unclassified | unclassified | unclassified | unclassified | unclassified | unclassified |
| Otu046580 | 0.946 | 0.6667 | 0.794 | 0.011 | Bacteria | Bacteria unclassified | unclassified | unclassified | unclassified | unclassified | unclassified |
| Otu046302 | 0.9089 | 0.6667 | 0.778 | 0.012 | Bacteria | Bacteria unclassified | unclassified | unclassified | unclassified | unclassified | unclassified |
| Otu018469 | 0.8127 | 1 | 0.901 | 0.011 | Bacteria | Bacteria unclassified | unclassified | unclassified | unclassified | unclassified | unclassified |
| Otu012705 | 0.8571 | 1 | 0.926 | 0.005 | Bacteria | Bacteria unclassified | unclassified | unclassified | unclassified | unclassified | unclassified |
| Otu012358 | 0.893 | 0.6667 | 0.772 | 0.011 | Bacteria | Bacteria unclassified | unclassified | unclassified | unclassified | unclassified | unclassified |
| Otu012356 | 0.8713 | 0.6667 | 0.762 | 0.011 | Bacteria | Bacteria unclassified | unclassified | unclassified | unclassified | unclassified | unclassified |
| Otu003242 | 0.9237 | 1 | 0.961 | 0.003 | Bacteria | Bacteria unclassified | unclassified | unclassified | unclassified | unclassified | unclassified |
| Otu119887 | 0.8579 | 1 | 0.926 | 0.005 | Bacteria | Bacteroidetes | Bacteroidetes unclassified | unclassified | unclassified | unclassified | unclassified |
| Otu047076 | 0.832 | 0.6667 | 0.745 | 0.037 | Bacteria | Bacteroidetes | Bacteroidetes unclassified | unclassified | unclassified | unclassified | unclassified |
| Otu006795 | 0.775 | 1 | 0.88 | 0.018 | Bacteria | Bacteroidetes | Bacteroidetes unclassified | unclassified | unclassified | unclassified | unclassified |
| Otu004011 | 0.9222 | 1 | 0.96 | 0.002 | Bacteria | Bacteroidetes | Bacteroidetes unclassified | unclassified | unclassified | unclassified | unclassified |
| Otu003666 | 0.9274 | 1 | 0.963 | 0.002 | Bacteria | Bacteroidetes | Bacteroidetes unclassified | unclassified | unclassified | unclassified | unclassified |
| Otu015089 | 0.8761 | 0.6667 | 0.764 | 0.021 | Bacteria | Bacteroidetes | Cytophagia | Cytophagales | Flammeovirgaceae | JTB248 | JTB248 unclassified |
| Otu006693 | 0.9151 | 1 | 0.957 | 0.001 | Bacteria | Bacteroidetes | Flavobacteriia | Flavobacteriales | Cryomorphaceae | Fluviicola | Fluviicola unclassified |
| Otu211355 | 0.8048 | 1 | 0.897 | 0.011 | Bacteria | Bacteroidetes | Flavobacteriia | Flavobacteriales | Flavobacteriaceae | Flavobacteriaceae unclassified | unclassified |
| Otu116861 | 0.8987 | 0.6667 | 0.774 | 0.011 | Bacteria | Bacteroidetes | Flavobacteriia | Flavobacteriales | Flavobacteriaceae | Flavobacteriaceae unclassified | unclassified |
| Otu041660 | 0.7884 | 0.6667 | 0.725 | 0.048 | Bacteria | Bacteroidetes | Flavobacteriia | Flavobacteriales | Flavobacteriaceae | Flavobacteriaceae unclassified | unclassified |
| Otu022060 | 0.7845 | 0.6667 | 0.723 | 0.035 | Bacteria | Bacteroidetes | Flavobacteriia | Flavobacteriales | Flavobacteriaceae | Flavobacteriaceae unclassified | unclassified |
| Otu017459 | 1 | 1 | 1 | 0.001 | Bacteria | Bacteroidetes | Flavobacteriia | Flavobacteriales | Flavobacteriaceae | Flavobacteriaceae unclassified | unclassified |
| Otu007496 | 0.8531 | 1 | 0.924 | 0.001 | Bacteria | Bacteroidetes | Flavobacteriia | Flavobacteriales | Flavobacteriaceae | Flavobacteriaceae unclassified | unclassified |
| Otu003757 | 0.8094 | 1 | 0.9 | 0.001 | Bacteria | Bacteroidetes | Flavobacteriia | Flavobacteriales | Flavobacteriaceae | Flavobacteriaceae unclassified | unclassified |
| Otu321727 | 0.8965 | 0.6667 | 0.773 | 0.019 | Bacteria | Bacteroidetes | Flavobacteriia | Flavobacteriales | Flavobacteriaceae | Polaribacter | Polaribacter unclassified |
| Otu211877 | 0.8878 | 0.6667 | 0.769 | 0.01 | Bacteria | Bacteroidetes | Flavobacteriia | Flavobacteriales | Flavobacteriales unclassified | unclassified | unclassified |
| Otu118209 | 0.8603 | 1 | 0.927 | 0.003 | Bacteria | Bacteroidetes | Flavobacteriia | Flavobacteriales | Flavobacteriales unclassified | unclassified | unclassified |
| Otu014867 | 0.9302 | 1 | 0.964 | 0.001 | Bacteria | Bacteroidetes | Flavobacteriia | Flavobacteriales | Flavobacteriales unclassified | unclassified | unclassified |
| Otu021656 | 0.9872 | 1 | 0.994 | 0.001 | Bacteria | Bacteroidetes | Saprospirae | Saprospirales | Saprospiraceae | Saprospira | Saprospira unclassified |
| Otu016096 | 0.882 | 0.6667 | 0.767 | 0.019 | Bacteria | Bacteroidetes | Saprospirae | Saprospirales | Saprospiraceae | Saprospira | Saprospira unclassified |
| Otu139019 | 0.8942 | 0.6667 | 0.772 | 0.019 | Bacteria | Bacteroidetes | Saprospirae | Saprospirales | Saprospiraceae | Saprospiraceae unclassified | unclassified |
| Otu008163 | 0.9248 | 0.6667 | 0.785 | 0.01 | Bacteria | Bacteroidetes | Sphingobacteriia | Sphingobacteriales | NS11-12 | NS11-12 unclassified | unclassified |
| Otu027109 | 0.9219 | 1 | 0.96 | 0.001 | Bacteria | Chloroflexi | SAR202 | SAR202 unclassified | unclassified | unclassified | unclassified |
| Otu008875 | 0.8614 | 1 | 0.928 | 0.002 | Bacteria | Chloroflexi | SAR202 | SAR202 unclassified | unclassified | unclassified | unclassified |
| Otu175966 | 0.8535 | 0.6667 | 0.754 | 0.039 | Bacteria | Cyanobacteria | Synechococcophycideae | Synechococcales | Synechococcaceae | Paulinella | Paulinella unclassified |
| Otu149206 | 0.866 | 0.6667 | 0.76 | 0.015 | Bacteria | Cyanobacteria | Synechococcophycideae | Synechococcales | Synechococcaceae | Synechococcaceae unclassified | unclassified |
| Otu118921 | 0.8425 | 0.6667 | 0.749 | 0.018 | Bacteria | Cyanobacteria | Synechococcophycideae | Synechococcales | Synechococcaceae | Synechococcaceae unclassified | unclassified |
| Otu118721 | 0.8707 | 0.6667 | 0.762 | 0.016 | Bacteria | Cyanobacteria | Synechococcophycideae | Synechococcales | Synechococcaceae | Synechococcaceae unclassified | unclassified |
| Otu065493 | 0.9255 | 0.6667 | 0.785 | 0.009 | Bacteria | Cyanobacteria | Synechococcophycideae | Synechococcales | Synechococcaceae | Synechococcaceae unclassified | unclassified |
| Otu065491 | 0.8595 | 0.6667 | 0.757 | 0.01 | Bacteria | Cyanobacteria | Synechococcophycideae | Synechococcales | Synechococcaceae | Synechococcaceae unclassified | unclassified |
| Otu065295 | 0.7859 | 0.6667 | 0.724 | 0.033 | Bacteria | Cyanobacteria | Synechococcophycideae | Synechococcales | Synechococcaceae | Synechococcaceae unclassified | unclassified |
| Otu032492 | 0.8774 | 0.6667 | 0.765 | 0.024 | Bacteria | Cyanobacteria | Synechococcophycideae | Synechococcales | Synechococcaceae | Synechococcaceae unclassified | unclassified |
| Otu022612 | 0.7926 | 0.6667 | 0.727 | 0.044 | Bacteria | Cyanobacteria | Synechococcophycideae | Synechococcales | Synechococcaceae | Synechococcaceae unclassified | unclassified |
| Otu010182 | 0.8836 | 0.6667 | 0.768 | 0.012 | Bacteria | Cyanobacteria | Synechococcophycideae | Synechococcales | Synechococcaceae | Synechococcaceae unclassified | unclassified |
| Otu008066 | 0.8734 | 0.6667 | 0.763 | 0.011 | Bacteria | Cyanobacteria | Synechococcophycideae | Synechococcales | Synechococcaceae | Synechococcaceae unclassified | unclassified |
| Otu007473 | 0.8708 | 0.6667 | 0.762 | 0.014 | Bacteria | Cyanobacteria | Synechococcophycideae | Synechococcales | Synechococcaceae | Synechococcaceae unclassified | unclassified |
| Otu007469 | 0.9666 | 1 | 0.983 | 0.001 | Bacteria | Cyanobacteria | Synechococcophycideae | Synechococcales | Synechococcaceae | Synechococcaceae unclassified | unclassified |
| Otu004653 | 0.973 | 0.6667 | 0.805 | 0.01 | Bacteria | Cyanobacteria | Synechococcophycideae | Synechococcales | Synechococcaceae | Synechococcaceae unclassified | unclassified |
| Otu004547 | 0.9435 | 0.6667 | 0.793 | 0.011 | Bacteria | Cyanobacteria | Synechococcophycideae | Synechococcales | Synechococcaceae | Synechococcaceae unclassified | unclassified |
| Otu002431 | 0.9638 | 1 | 0.982 | 0.001 | Bacteria | Cyanobacteria | Synechococcophycideae | Synechococcales | Synechococcaceae | Synechococcaceae unclassified | unclassified |
| Otu001723 | 0.8424 | 0.6667 | 0.749 | 0.013 | Bacteria | Cyanobacteria | Synechococcophycideae | Synechococcales | Synechococcaceae | Synechococcaceae unclassified | unclassified |
| Otu000717 | 0.8951 | 0.6667 | 0.772 | 0.006 | Bacteria | Cyanobacteria | Synechococcophycideae | Synechococcales | Synechococcaceae | Synechococcaceae unclassified | unclassified |
| Otu183596 | 0.8684 | 0.6667 | 0.761 | 0.012 | Bacteria | Cyanobacteria | Synechococcophycideae | Synechococcales | Synechococcaceae | Synechococcus | Synechococcus unclassified |
| Otu176250 | 0.8455 | 0.6667 | 0.751 | 0.018 | Bacteria | Cyanobacteria | Synechococcophycideae | Synechococcales | Synechococcaceae | Synechococcus | Synechococcus unclassified |
| Otu118879 | 0.8545 | 0.6667 | 0.755 | 0.024 | Bacteria | Cyanobacteria | Synechococcophycideae | Synechococcales | Synechococcaceae | Synechococcus | Synechococcus unclassified |
| Otu118686 | 0.8205 | 0.6667 | 0.74 | 0.023 | Bacteria | Cyanobacteria | Synechococcophycideae | Synechococcales | Synechococcaceae | Synechococcus | Synechococcus unclassified |
| Otu065711 | 0.8727 | 0.6667 | 0.763 | 0.018 | Bacteria | Cyanobacteria | Synechococcophycideae | Synechococcales | Synechococcaceae | Synechococcus | Synechococcus unclassified |
| Otu065337 | 0.9457 | 0.6667 | 0.794 | 0.01 | Bacteria | Cyanobacteria | Synechococcophycideae | Synechococcales | Synechococcaceae | Synechococcus | Synechococcus unclassified |
| Otu022599 | 0.8458 | 1 | 0.92 | 0.002 | Bacteria | Cyanobacteria | Synechococcophycideae | Synechococcales | Synechococcaceae | Synechococcus | Synechococcus unclassified |
| Otu011357 | 0.9268 | 0.6667 | 0.786 | 0.006 | Bacteria | Cyanobacteria | Synechococcophycideae | Synechococcales | Synechococcaceae | Synechococcus | Synechococcus unclassified |
| Otu003589 | 0.7942 | 0.6667 | 0.728 | 0.049 | Bacteria | Gemmatimonadetes | Gemm-2 | Gemm-2 unclassified | unclassified | unclassified | unclassified |
| Otu060131 | 0.8744 | 1 | 0.935 | 0.003 | Bacteria | Nitrospirae | Nitrospira | Nitrospirales | Nitrospiraceae | Nitrospiraceae unclassified | unclassified |
| Otu043335 | 0.8411 | 1 | 0.917 | 0.006 | Bacteria | Nitrospirae | Nitrospira | Nitrospirales | Nitrospiraceae | Nitrospiraceae unclassified | unclassified |
| Otu003184 | 0.8487 | 1 | 0.921 | 0.005 | Bacteria | Nitrospirae | Nitrospira | Nitrospirales | Nitrospiraceae | Nitrospiraceae unclassified | unclassified |
| Otu000144 | 0.8476 | 1 | 0.921 | 0.005 | Bacteria | Nitrospirae | Nitrospira | Nitrospirales | Nitrospiraceae | Nitrospiraceae unclassified | unclassified |
| Otu225454 | 0.9147 | 0.6667 | 0.781 | 0.012 | Bacteria | Planctomycetes | OM190 | CL500-15 | CL500-15 unclassified | unclassified | unclassified |
| Otu019483 | 0.8714 | 1 | 0.933 | 0.001 | Bacteria | Planctomycetes | OM190 | CL500-15 | CL500-15 unclassified | unclassified | unclassified |
| Otu022040 | 0.8731 | 1 | 0.934 | 0.001 | Bacteria | Planctomycetes | Phycisphaerae | Phycisphaerales | Phycisphaerales unclassified | unclassified | unclassified |
| Otu006817 | 0.8503 | 1 | 0.922 | 0.001 | Bacteria | Planctomycetes | Phycisphaerae | Phycisphaerales | Phycisphaerales unclassified | unclassified | unclassified |
| Otu002742 | 0.8622 | 1 | 0.929 | 0.001 | Bacteria | Planctomycetes | Phycisphaerae | Phycisphaerales | Phycisphaerales unclassified | unclassified | unclassified |
| Otu222567 | 0.9792 | 1 | 0.99 | 0.001 | Bacteria | Planctomycetes | Planctomycetia | Planctomycetales | Planctomycetaceae | Planctomyces | Planctomyces unclassified |
| Otu041134 | 0.8771 | 1 | 0.937 | 0.002 | Bacteria | Planctomycetes | Planctomycetia | Planctomycetales | Planctomycetaceae | Planctomyces | Planctomyces unclassified |
| Otu032760 | 0.9336 | 1 | 0.966 | 0.001 | Bacteria | Planctomycetes | Planctomycetia | Planctomycetales | Planctomycetaceae | Planctomyces | Planctomyces unclassified |
| Otu019765 | 0.8667 | 0.6667 | 0.76 | 0.02 | Bacteria | Planctomycetes | Planctomycetia | Planctomycetales | Planctomycetaceae | Planctomyces | Planctomyces unclassified |
| Otu008310 | 0.9423 | 1 | 0.971 | 0.001 | Bacteria | Planctomycetes | Planctomycetia | Planctomycetales | Planctomycetaceae | Planctomyces | Planctomyces unclassified |
| Otu005939 | 0.8396 | 1 | 0.916 | 0.002 | Bacteria | Planctomycetes | Planctomycetia | Planctomycetales | Planctomycetaceae | Planctomyces | Planctomyces unclassified |
| Otu004203 | 0.8658 | 1 | 0.931 | 0.003 | Bacteria | Planctomycetes | Planctomycetia | Planctomycetales | Planctomycetaceae | Planctomyces | Planctomyces unclassified |
| Otu080821 | 0.8674 | 1 | 0.931 | 0.004 | Bacteria | Proteobacteria | Alphaproteobacteria | Alphaproteobacteria unclassified | unclassified | unclassified | unclassified |
| Otu079862 | 0.8222 | 0.6667 | 0.74 | 0.025 | Bacteria | Proteobacteria | Alphaproteobacteria | Alphaproteobacteria unclassified | unclassified | unclassified | unclassified |
| Otu056797 | 0.8859 | 1 | 0.941 | 0.001 | Bacteria | Proteobacteria | Alphaproteobacteria | Alphaproteobacteria unclassified | unclassified | unclassified | unclassified |
| Otu034272 | 0.8834 | 1 | 0.94 | 0.001 | Bacteria | Proteobacteria | Alphaproteobacteria | Alphaproteobacteria unclassified | unclassified | unclassified | unclassified |
| Otu033873 | 0.7277 | 1 | 0.853 | 0.022 | Bacteria | Proteobacteria | Alphaproteobacteria | Alphaproteobacteria unclassified | unclassified | unclassified | unclassified |
| Otu024592 | 0.8039 | 1 | 0.897 | 0.005 | Bacteria | Proteobacteria | Alphaproteobacteria | Alphaproteobacteria unclassified | unclassified | unclassified | unclassified |
| Otu024414 | 0.8082 | 0.6667 | 0.734 | 0.03 | Bacteria | Proteobacteria | Alphaproteobacteria | Alphaproteobacteria unclassified | unclassified | unclassified | unclassified |
| Otu011946 | 0.9217 | 1 | 0.96 | 0.001 | Bacteria | Proteobacteria | Alphaproteobacteria | Alphaproteobacteria unclassified | unclassified | unclassified | unclassified |

| | | | | | | | | | | | |
|---|---|---|---|---|---|---|---|---|---|---|---|
| Otu010818 | 0.8896 | 1 | 0.943 | 0.001 | Bacteria | Proteobacteria | Alphaproteobacteria | Alphaproteobacteria unclassified | unclassified | unclassified | unclassified |
| Otu006577 | 0.8705 | 1 | 0.933 | 0.006 | Bacteria | Proteobacteria | Alphaproteobacteria | Alphaproteobacteria unclassified | unclassified | unclassified | unclassified |
| Otu005253 | 0.9367 | 1 | 0.968 | 0.001 | Bacteria | Proteobacteria | Alphaproteobacteria | Alphaproteobacteria unclassified | unclassified | unclassified | unclassified |
| Otu005116 | 0.9857 | 1 | 0.993 | 0.001 | Bacteria | Proteobacteria | Alphaproteobacteria | Alphaproteobacteria unclassified | unclassified | unclassified | unclassified |
| Otu002941 | 0.8486 | 0.6667 | 0.752 | 0.028 | Bacteria | Proteobacteria | Alphaproteobacteria | Alphaproteobacteria unclassified | unclassified | unclassified | unclassified |
| Otu062087 | 0.9343 | 1 | 0.967 | 0.001 | Bacteria | Proteobacteria | Alphaproteobacteria | Rhodobacterales | Rhodobacteraceae | Loktanella | Loktanella salsilacus |
| Otu142563 | 0.9372 | 0.6667 | 0.79 | 0.013 | Bacteria | Proteobacteria | Alphaproteobacteria | Rhodobacterales | Rhodobacteraceae | Octadecabacter | Octadecabacter unclassified |
| Otu077267 | 0.7649 | 1 | 0.875 | 0.006 | Bacteria | Proteobacteria | Alphaproteobacteria | Rhodobacterales | Rhodobacteraceae | Octadecabacter | Octadecabacter unclassified |
| Otu061680 | 1 | 0.6667 | 0.816 | 0.006 | Bacteria | Proteobacteria | Alphaproteobacteria | Rhodobacterales | Rhodobacteraceae | Phaeobacter | Phaeobacter unclassified |
| Otu149554 | 0.964 | 0.6667 | 0.802 | 0.006 | Bacteria | Proteobacteria | Alphaproteobacteria | Rhodobacterales | Rhodobacteraceae | Pseudoruegeria | Pseudoruegeria unclassified |
| Otu133959 | 0.9335 | 0.6667 | 0.789 | 0.005 | Bacteria | Proteobacteria | Alphaproteobacteria | Rhodobacterales | Rhodobacteraceae | Rhodobacteraceae unclassified | unclassified |
| Otu079006 | 0.7892 | 0.6667 | 0.725 | 0.038 | Bacteria | Proteobacteria | Alphaproteobacteria | Rhodobacterales | Rhodobacteraceae | Rhodobacteraceae unclassified | unclassified |
| Otu078923 | 1 | 0.6667 | 0.816 | 0.01 | Bacteria | Proteobacteria | Alphaproteobacteria | Rhodobacterales | Rhodobacteraceae | Rhodobacteraceae unclassified | unclassified |
| Otu077130 | 0.9627 | 1 | 0.981 | 0.001 | Bacteria | Proteobacteria | Alphaproteobacteria | Rhodobacterales | Rhodobacteraceae | Rhodobacteraceae unclassified | unclassified |
| Otu018640 | 0.76 | 1 | 0.872 | 0.025 | Bacteria | Proteobacteria | Alphaproteobacteria | Rhodobacterales | Rhodobacteraceae | Rhodobacteraceae unclassified | unclassified |
| Otu015104 | 0.9339 | 1 | 0.966 | 0.001 | Bacteria | Proteobacteria | Alphaproteobacteria | Rhodobacterales | Rhodobacteraceae | Rhodobacteraceae unclassified | unclassified |
| Otu000838 | 0.7695 | 1 | 0.877 | 0.02 | Bacteria | Proteobacteria | Alphaproteobacteria | Rhodobacterales | Rhodobacteraceae | Rhodobacteraceae unclassified | unclassified |
| Otu004101 | 0.8272 | 1 | 0.909 | 0.008 | Bacteria | Proteobacteria | Alphaproteobacteria | Rhodobacterales | Rhodobacterales unclassified | unclassified | unclassified |
| Otu080370 | 0.8461 | 1 | 0.92 | 0.004 | Bacteria | Proteobacteria | Alphaproteobacteria | Rhodospirillales | Rhodospirillaceae | Rhodospirillaceae unclassified | unclassified |
| Otu059154 | 0.7985 | 0.6667 | 0.73 | 0.026 | Bacteria | Proteobacteria | Alphaproteobacteria | Rhodospirillales | Rhodospirillaceae | Rhodospirillaceae unclassified | unclassified |
| Otu055727 | 0.8593 | 1 | 0.927 | 0.002 | Bacteria | Proteobacteria | Alphaproteobacteria | Rhodospirillales | Rhodospirillaceae | Rhodospirillaceae unclassified | unclassified |
| Otu023816 | 0.8739 | 0.6667 | 0.763 | 0.021 | Bacteria | Proteobacteria | Alphaproteobacteria | Rhodospirillales | Rhodospirillaceae | Rhodospirillaceae unclassified | unclassified |
| Otu021560 | 0.8271 | 1 | 0.909 | 0.001 | Bacteria | Proteobacteria | Alphaproteobacteria | Rhodospirillales | Rhodospirillaceae | Rhodospirillaceae unclassified | unclassified |
| Otu018230 | 0.8593 | 1 | 0.927 | 0.006 | Bacteria | Proteobacteria | Alphaproteobacteria | Rhodospirillales | Rhodospirillaceae | Rhodospirillaceae unclassified | unclassified |
| Otu008256 | 0.9207 | 1 | 0.96 | 0.001 | Bacteria | Proteobacteria | Alphaproteobacteria | Rhodospirillales | Rhodospirillaceae | Rhodospirillaceae unclassified | unclassified |
| Otu048559 | 0.866 | 1 | 0.931 | 0.003 | Bacteria | Proteobacteria | Betaproteobacteria | Betaproteobacteria unclassified | unclassified | unclassified | unclassified |
| Otu010968 | 0.7599 | 1 | 0.872 | 0.002 | Bacteria | Proteobacteria | Betaproteobacteria | Betaproteobacteria unclassified | unclassified | unclassified | unclassified |
| Otu010354 | 0.852 | 1 | 0.923 | 0.004 | Bacteria | Proteobacteria | Deltaproteobacteria | Bdellovibrionales | Bdellovibrionaceae | Bdellovibrio | Bdellovibrio unclassified |
| Otu035923 | 0.9559 | 1 | 0.978 | 0.001 | Bacteria | Proteobacteria | Deltaproteobacteria | Myxococcales | Cystobacterineae | Cystobacterineae unclassified | unclassified |
| Otu139538 | 0.9109 | 0.6667 | 0.779 | 0.006 | Bacteria | Proteobacteria | Deltaproteobacteria | Myxococcales | Myxococcales unclassified | unclassified | unclassified |
| Otu043576 | 0.9084 | 0.6667 | 0.778 | 0.006 | Bacteria | Proteobacteria | Deltaproteobacteria | Myxococcales | Myxococcales unclassified | unclassified | unclassified |
| Otu026968 | 0.9439 | 1 | 0.972 | 0.001 | Bacteria | Proteobacteria | Deltaproteobacteria | Myxococcales | OM27 | OM27 unclassified | unclassified |
| Otu005402 | 1 | 0.6667 | 0.816 | 0.006 | Bacteria | Proteobacteria | Deltaproteobacteria | Spirobacillales | Spirobacillales unclassified | unclassified | unclassified |
| Otu020000 | 0.8831 | 0.6667 | 0.767 | 0.008 | Bacteria | Proteobacteria | Epsilonproteobacteria | Campylobacterales | Helicobacteraceae | Helicobacteraceae unclassified | unclassified |
| Otu158589 | 0.9014 | 1 | 0.949 | 0.001 | Bacteria | Proteobacteria | Gammaproteobacteria | Alteromonadales | Alteromonadaceae | HTCC2207 | HTCC2207 unclassified |
| Otu028611 | 0.9019 | 0.6667 | 0.775 | 0.014 | Bacteria | Proteobacteria | Gammaproteobacteria | Alteromonadales | Alteromonadaceae | Microbulbifer | Microbulbifer unclassified |
| Otu123690 | 0.8522 | 0.6667 | 0.754 | 0.015 | Bacteria | Proteobacteria | Gammaproteobacteria | Alteromonadales | Alteromonadales unclassified | unclassified | unclassified |
| Otu121450 | 0.9803 | 0.6667 | 0.808 | 0.006 | Bacteria | Proteobacteria | Gammaproteobacteria | Alteromonadales | Alteromonadales unclassified | unclassified | unclassified |
| Otu110271 | 0.8476 | 0.6667 | 0.752 | 0.02 | Bacteria | Proteobacteria | Gammaproteobacteria | Alteromonadales | Alteromonadales unclassified | unclassified | unclassified |
| Otu085547 | 0.7561 | 1 | 0.87 | 0.024 | Bacteria | Proteobacteria | Gammaproteobacteria | Alteromonadales | Alteromonadales unclassified | unclassified | unclassified |
| Otu006014 | 0.8509 | 1 | 0.922 | 0.001 | Bacteria | Proteobacteria | Gammaproteobacteria | Alteromonadales | Alteromonadales unclassified | unclassified | unclassified |
| Otu016641 | 0.8035 | 1 | 0.896 | 0.001 | Bacteria | Proteobacteria | Gammaproteobacteria | Alteromonadales | HTCC2188 | HTCC | HTCC unclassified |
| Otu008277 | 0.81 | 1 | 0.9 | 0.001 | Bacteria | Proteobacteria | Gammaproteobacteria | Alteromonadales | HTCC2188 | HTCC | HTCC unclassified |
| Otu002028 | 0.8382 | 1 | 0.916 | 0.001 | Bacteria | Proteobacteria | Gammaproteobacteria | Alteromonadales | HTCC2188 | HTCC | HTCC unclassified |
| Otu011993 | 0.8957 | 1 | 0.946 | 0.003 | Bacteria | Proteobacteria | Gammaproteobacteria | Alteromonadales | OM60 | OM60 unclassified | unclassified |
| Otu122390 | 1 | 0.6667 | 0.816 | 0.005 | Bacteria | Proteobacteria | Gammaproteobacteria | Enterobacteriales | Enterobacteriaceae | Enterobacteriaceae unclassified | unclassified |
| Otu031704 | 0.8919 | 0.6667 | 0.771 | 0.016 | Bacteria | Proteobacteria | Gammaproteobacteria | Enterobacteriales | Enterobacteriaceae | Enterobacteriaceae unclassified | unclassified |
| Otu013513 | 0.9782 | 1 | 0.989 | 0.001 | Bacteria | Proteobacteria | Gammaproteobacteria | Enterobacteriales | Enterobacteriaceae | Enterobacteriaceae unclassified | unclassified |
| Otu262863 | 1 | 0.6667 | 0.816 | 0.006 | Bacteria | Proteobacteria | Gammaproteobacteria | Gammaproteobacteria unclassified | unclassified | unclassified | unclassified |
| Otu259625 | 0.8593 | 0.6667 | 0.757 | 0.024 | Bacteria | Proteobacteria | Gammaproteobacteria | Gammaproteobacteria unclassified | unclassified | unclassified | unclassified |
| Otu257013 | 0.9356 | 1 | 0.967 | 0.002 | Bacteria | Proteobacteria | Gammaproteobacteria | Gammaproteobacteria unclassified | unclassified | unclassified | unclassified |
| Otu252313 | 0.9595 | 1 | 0.98 | 0.002 | Bacteria | Proteobacteria | Gammaproteobacteria | Gammaproteobacteria unclassified | unclassified | unclassified | unclassified |
| Otu251699 | 1 | 0.6667 | 0.816 | 0.01 | Bacteria | Proteobacteria | Gammaproteobacteria | Gammaproteobacteria unclassified | unclassified | unclassified | unclassified |
| Otu251484 | 0.9533 | 1 | 0.976 | 0.001 | Bacteria | Proteobacteria | Gammaproteobacteria | Gammaproteobacteria unclassified | unclassified | unclassified | unclassified |
| Otu246892 | 1 | 1 | 1 | 0.001 | Bacteria | Proteobacteria | Gammaproteobacteria | Gammaproteobacteria unclassified | unclassified | unclassified | unclassified |
| Otu240225 | 0.9428 | 1 | 0.971 | 0.001 | Bacteria | Proteobacteria | Gammaproteobacteria | Gammaproteobacteria unclassified | unclassified | unclassified | unclassified |
| Otu240018 | 0.9547 | 1 | 0.977 | 0.001 | Bacteria | Proteobacteria | Gammaproteobacteria | Gammaproteobacteria unclassified | unclassified | unclassified | unclassified |
| Otu237000 | 0.9389 | 0.6667 | 0.791 | 0.021 | Bacteria | Proteobacteria | Gammaproteobacteria | Gammaproteobacteria unclassified | unclassified | unclassified | unclassified |
| Otu220169 | 0.9713 | 1 | 0.986 | 0.001 | Bacteria | Proteobacteria | Gammaproteobacteria | Gammaproteobacteria unclassified | unclassified | unclassified | unclassified |
| Otu218880 | 1 | 0.6667 | 0.816 | 0.01 | Bacteria | Proteobacteria | Gammaproteobacteria | Gammaproteobacteria unclassified | unclassified | unclassified | unclassified |
| Otu217528 | 0.91 | 1 | 0.954 | 0.002 | Bacteria | Proteobacteria | Gammaproteobacteria | Gammaproteobacteria unclassified | unclassified | unclassified | unclassified |
| Otu215775 | 0.9574 | 1 | 0.978 | 0.001 | Bacteria | Proteobacteria | Gammaproteobacteria | Gammaproteobacteria unclassified | unclassified | unclassified | unclassified |
| Otu206069 | 1 | 0.6667 | 0.816 | 0.01 | Bacteria | Proteobacteria | Gammaproteobacteria | Gammaproteobacteria unclassified | unclassified | unclassified | unclassified |
| Otu174598 | 0.9683 | 0.6667 | 0.803 | 0.006 | Bacteria | Proteobacteria | Gammaproteobacteria | Gammaproteobacteria unclassified | unclassified | unclassified | unclassified |
| Otu129207 | 0.9761 | 1 | 0.988 | 0.001 | Bacteria | Proteobacteria | Gammaproteobacteria | Gammaproteobacteria unclassified | unclassified | unclassified | unclassified |
| Otu128129 | 0.924 | 1 | 0.961 | 0.001 | Bacteria | Proteobacteria | Gammaproteobacteria | Gammaproteobacteria unclassified | unclassified | unclassified | unclassified |
| Otu127072 | 0.9377 | 1 | 0.968 | 0.001 | Bacteria | Proteobacteria | Gammaproteobacteria | Gammaproteobacteria unclassified | unclassified | unclassified | unclassified |
| Otu126871 | 0.879 | 0.6667 | 0.766 | 0.012 | Bacteria | Proteobacteria | Gammaproteobacteria | Gammaproteobacteria unclassified | unclassified | unclassified | unclassified |
| Otu126595 | 0.9248 | 1 | 0.962 | 0.001 | Bacteria | Proteobacteria | Gammaproteobacteria | Gammaproteobacteria unclassified | unclassified | unclassified | unclassified |
| Otu125579 | 0.8897 | 1 | 0.943 | 0.001 | Bacteria | Proteobacteria | Gammaproteobacteria | Gammaproteobacteria unclassified | unclassified | unclassified | unclassified |
| Otu124821 | 1 | 0.6667 | 0.816 | 0.006 | Bacteria | Proteobacteria | Gammaproteobacteria | Gammaproteobacteria unclassified | unclassified | unclassified | unclassified |
| Otu124518 | 0.9463 | 1 | 0.973 | 0.001 | Bacteria | Proteobacteria | Gammaproteobacteria | Gammaproteobacteria unclassified | unclassified | unclassified | unclassified |
| Otu124374 | 1 | 0.6667 | 0.816 | 0.01 | Bacteria | Proteobacteria | Gammaproteobacteria | Gammaproteobacteria unclassified | unclassified | unclassified | unclassified |
| Otu124295 | 0.9202 | 0.6667 | 0.783 | 0.012 | Bacteria | Proteobacteria | Gammaproteobacteria | Gammaproteobacteria unclassified | unclassified | unclassified | unclassified |
| Otu123893 | 0.9761 | 1 | 0.988 | 0.001 | Bacteria | Proteobacteria | Gammaproteobacteria | Gammaproteobacteria unclassified | unclassified | unclassified | unclassified |
| Otu123201 | 0.9349 | 1 | 0.967 | 0.001 | Bacteria | Proteobacteria | Gammaproteobacteria | Gammaproteobacteria unclassified | unclassified | unclassified | unclassified |
| Otu122464 | 0.9487 | 0.6667 | 0.795 | 0.006 | Bacteria | Proteobacteria | Gammaproteobacteria | Gammaproteobacteria unclassified | unclassified | unclassified | unclassified |
| Otu121900 | 0.9151 | 1 | 0.957 | 0.001 | Bacteria | Proteobacteria | Gammaproteobacteria | Gammaproteobacteria unclassified | unclassified | unclassified | unclassified |
| Otu121803 | 0.8852 | 0.6667 | 0.768 | 0.016 | Bacteria | Proteobacteria | Gammaproteobacteria | Gammaproteobacteria unclassified | unclassified | unclassified | unclassified |
| Otu121795 | 1 | 0.6667 | 0.816 | 0.006 | Bacteria | Proteobacteria | Gammaproteobacteria | Gammaproteobacteria unclassified | unclassified | unclassified | unclassified |
| Otu121788 | 0.9395 | 1 | 0.969 | 0.001 | Bacteria | Proteobacteria | Gammaproteobacteria | Gammaproteobacteria unclassified | unclassified | unclassified | unclassified |
| Otu121434 | 0.9467 | 0.6667 | 0.794 | 0.01 | Bacteria | Proteobacteria | Gammaproteobacteria | Gammaproteobacteria unclassified | unclassified | unclassified | unclassified |
| Otu120747 | 0.9339 | 1 | 0.966 | 0.001 | Bacteria | Proteobacteria | Gammaproteobacteria | Gammaproteobacteria unclassified | unclassified | unclassified | unclassified |
| Otu120711 | 0.9631 | 1 | 0.981 | 0.002 | Bacteria | Proteobacteria | Gammaproteobacteria | Gammaproteobacteria unclassified | unclassified | unclassified | unclassified |
| Otu117940 | 0.9832 | 1 | 0.992 | 0.001 | Bacteria | Proteobacteria | Gammaproteobacteria | Gammaproteobacteria unclassified | unclassified | unclassified | unclassified |
| Otu117884 | 0.9541 | 1 | 0.977 | 0.001 | Bacteria | Proteobacteria | Gammaproteobacteria | Gammaproteobacteria unclassified | unclassified | unclassified | unclassified |
| Otu111028 | 0.8904 | 0.6667 | 0.77 | 0.016 | Bacteria | Proteobacteria | Gammaproteobacteria | Gammaproteobacteria unclassified | unclassified | unclassified | unclassified |
| Otu110337 | 0.8639 | 0.6667 | 0.759 | 0.046 | Bacteria | Proteobacteria | Gammaproteobacteria | Gammaproteobacteria unclassified | unclassified | unclassified | unclassified |

| | | | | | | | | | | | |
|---|---|---|---|---|---|---|---|---|---|---|---|
| Otu110251 | 0.9027 | 1 | 0.95 | 0.004 | Bacteria | Proteobacteria | Gammaproteobacteria | Gammaproteobacteria unclassified | unclassified | unclassified | unclassified |
| Otu110247 | 1 | 0.6667 | 0.816 | 0.006 | Bacteria | Proteobacteria | Gammaproteobacteria | Gammaproteobacteria unclassified | unclassified | unclassified | unclassified |
| Otu109884 | 0.9469 | 1 | 0.973 | 0.001 | Bacteria | Proteobacteria | Gammaproteobacteria | Gammaproteobacteria unclassified | unclassified | unclassified | unclassified |
| Otu109528 | 0.9137 | 1 | 0.956 | 0.004 | Bacteria | Proteobacteria | Gammaproteobacteria | Gammaproteobacteria unclassified | unclassified | unclassified | unclassified |
| Otu108091 | 0.9291 | 0.6667 | 0.787 | 0.011 | Bacteria | Proteobacteria | Gammaproteobacteria | Gammaproteobacteria unclassified | unclassified | unclassified | unclassified |
| Otu066273 | 0.9096 | 1 | 0.954 | 0.004 | Bacteria | Proteobacteria | Gammaproteobacteria | Gammaproteobacteria unclassified | unclassified | unclassified | unclassified |
| Otu065662 | 0.9341 | 1 | 0.967 | 0.001 | Bacteria | Proteobacteria | Gammaproteobacteria | Gammaproteobacteria unclassified | unclassified | unclassified | unclassified |
| Otu064765 | 0.9618 | 1 | 0.981 | 0.002 | Bacteria | Proteobacteria | Gammaproteobacteria | Gammaproteobacteria unclassified | unclassified | unclassified | unclassified |
| Otu063716 | 0.8727 | 0.6667 | 0.763 | 0.016 | Bacteria | Proteobacteria | Gammaproteobacteria | Gammaproteobacteria unclassified | unclassified | unclassified | unclassified |
| Otu063692 | 0.9375 | 1 | 0.968 | 0.002 | Bacteria | Proteobacteria | Gammaproteobacteria | Gammaproteobacteria unclassified | unclassified | unclassified | unclassified |
| Otu063030 | 0.9346 | 1 | 0.967 | 0.001 | Bacteria | Proteobacteria | Gammaproteobacteria | Gammaproteobacteria unclassified | unclassified | unclassified | unclassified |
| Otu060722 | 0.9765 | 1 | 0.988 | 0.001 | Bacteria | Proteobacteria | Gammaproteobacteria | Gammaproteobacteria unclassified | unclassified | unclassified | unclassified |
| Otu060538 | 0.9752 | 1 | 0.988 | 0.001 | Bacteria | Proteobacteria | Gammaproteobacteria | Gammaproteobacteria unclassified | unclassified | unclassified | unclassified |
| Otu057808 | 0.9476 | 1 | 0.973 | 0.001 | Bacteria | Proteobacteria | Gammaproteobacteria | Gammaproteobacteria unclassified | unclassified | unclassified | unclassified |
| Otu057419 | 0.965 | 1 | 0.982 | 0.002 | Bacteria | Proteobacteria | Gammaproteobacteria | Gammaproteobacteria unclassified | unclassified | unclassified | unclassified |
| Otu057191 | 0.9538 | 1 | 0.977 | 0.001 | Bacteria | Proteobacteria | Gammaproteobacteria | Gammaproteobacteria unclassified | unclassified | unclassified | unclassified |
| Otu057172 | 0.9465 | 1 | 0.973 | 0.001 | Bacteria | Proteobacteria | Gammaproteobacteria | Gammaproteobacteria unclassified | unclassified | unclassified | unclassified |
| Otu057083 | 1 | 0.6667 | 0.816 | 0.01 | Bacteria | Proteobacteria | Gammaproteobacteria | Gammaproteobacteria unclassified | unclassified | unclassified | unclassified |
| Otu057077 | 0.949 | 1 | 0.974 | 0.001 | Bacteria | Proteobacteria | Gammaproteobacteria | Gammaproteobacteria unclassified | unclassified | unclassified | unclassified |
| Otu054125 | 0.9285 | 1 | 0.964 | 0.001 | Bacteria | Proteobacteria | Gammaproteobacteria | Gammaproteobacteria unclassified | unclassified | unclassified | unclassified |
| Otu053691 | 0.9398 | 0.6667 | 0.792 | 0.011 | Bacteria | Proteobacteria | Gammaproteobacteria | Gammaproteobacteria unclassified | unclassified | unclassified | unclassified |
| Otu053648 | 0.9491 | 1 | 0.974 | 0.002 | Bacteria | Proteobacteria | Gammaproteobacteria | Gammaproteobacteria unclassified | unclassified | unclassified | unclassified |
| Otu052811 | 0.8544 | 1 | 0.924 | 0.001 | Bacteria | Proteobacteria | Gammaproteobacteria | Gammaproteobacteria unclassified | unclassified | unclassified | unclassified |
| Otu052808 | 0.9043 | 0.6667 | 0.776 | 0.012 | Bacteria | Proteobacteria | Gammaproteobacteria | Gammaproteobacteria unclassified | unclassified | unclassified | unclassified |
| Otu052275 | 0.9307 | 0.6667 | 0.788 | 0.007 | Bacteria | Proteobacteria | Gammaproteobacteria | Gammaproteobacteria unclassified | unclassified | unclassified | unclassified |
| Otu051884 | 0.877 | 0.6667 | 0.765 | 0.032 | Bacteria | Proteobacteria | Gammaproteobacteria | Gammaproteobacteria unclassified | unclassified | unclassified | unclassified |
| Otu051569 | 0.9822 | 1 | 0.991 | 0.001 | Bacteria | Proteobacteria | Gammaproteobacteria | Gammaproteobacteria unclassified | unclassified | unclassified | unclassified |
| Otu051199 | 0.9531 | 0.6667 | 0.797 | 0.01 | Bacteria | Proteobacteria | Gammaproteobacteria | Gammaproteobacteria unclassified | unclassified | unclassified | unclassified |
| Otu050514 | 0.9632 | 0.6667 | 0.801 | 0.005 | Bacteria | Proteobacteria | Gammaproteobacteria | Gammaproteobacteria unclassified | unclassified | unclassified | unclassified |
| Otu050227 | 1 | 1 | 1 | 0.001 | Bacteria | Proteobacteria | Gammaproteobacteria | Gammaproteobacteria unclassified | unclassified | unclassified | unclassified |
| Otu050200 | 0.7972 | 0.6667 | 0.729 | 0.048 | Bacteria | Proteobacteria | Gammaproteobacteria | Gammaproteobacteria unclassified | unclassified | unclassified | unclassified |
| Otu047916 | 0.9786 | 1 | 0.989 | 0.001 | Bacteria | Proteobacteria | Gammaproteobacteria | Gammaproteobacteria unclassified | unclassified | unclassified | unclassified |
| Otu042128 | 0.8943 | 0.6667 | 0.772 | 0.017 | Bacteria | Proteobacteria | Gammaproteobacteria | Gammaproteobacteria unclassified | unclassified | unclassified | unclassified |
| Otu038184 | 0.8814 | 0.6667 | 0.767 | 0.012 | Bacteria | Proteobacteria | Gammaproteobacteria | Gammaproteobacteria unclassified | unclassified | unclassified | unclassified |
| Otu032375 | 0.8973 | 0.6667 | 0.773 | 0.008 | Bacteria | Proteobacteria | Gammaproteobacteria | Gammaproteobacteria unclassified | unclassified | unclassified | unclassified |
| Otu032365 | 0.942 | 1 | 0.971 | 0.001 | Bacteria | Proteobacteria | Gammaproteobacteria | Gammaproteobacteria unclassified | unclassified | unclassified | unclassified |
| Otu031976 | 0.9536 | 1 | 0.977 | 0.001 | Bacteria | Proteobacteria | Gammaproteobacteria | Gammaproteobacteria unclassified | unclassified | unclassified | unclassified |
| Otu030879 | 0.8686 | 0.6667 | 0.761 | 0.024 | Bacteria | Proteobacteria | Gammaproteobacteria | Gammaproteobacteria unclassified | unclassified | unclassified | unclassified |
| Otu030672 | 0.8709 | 0.6667 | 0.762 | 0.012 | Bacteria | Proteobacteria | Gammaproteobacteria | Gammaproteobacteria unclassified | unclassified | unclassified | unclassified |
| Otu030656 | 0.968 | 0.6667 | 0.803 | 0.006 | Bacteria | Proteobacteria | Gammaproteobacteria | Gammaproteobacteria unclassified | unclassified | unclassified | unclassified |
| Otu025480 | 0.9667 | 1 | 0.983 | 0.002 | Bacteria | Proteobacteria | Gammaproteobacteria | Gammaproteobacteria unclassified | unclassified | unclassified | unclassified |
| Otu025428 | 0.9454 | 1 | 0.972 | 0.001 | Bacteria | Proteobacteria | Gammaproteobacteria | Gammaproteobacteria unclassified | unclassified | unclassified | unclassified |
| Otu024580 | 0.9644 | 0.6667 | 0.802 | 0.006 | Bacteria | Proteobacteria | Gammaproteobacteria | Gammaproteobacteria unclassified | unclassified | unclassified | unclassified |
| Otu024376 | 0.939 | 1 | 0.969 | 0.001 | Bacteria | Proteobacteria | Gammaproteobacteria | Gammaproteobacteria unclassified | unclassified | unclassified | unclassified |
| Otu024324 | 0.9181 | 1 | 0.958 | 0.003 | Bacteria | Proteobacteria | Gammaproteobacteria | Gammaproteobacteria unclassified | unclassified | unclassified | unclassified |
| Otu024313 | 0.9591 | 1 | 0.979 | 0.002 | Bacteria | Proteobacteria | Gammaproteobacteria | Gammaproteobacteria unclassified | unclassified | unclassified | unclassified |
| Otu023864 | 0.9007 | 1 | 0.949 | 0.003 | Bacteria | Proteobacteria | Gammaproteobacteria | Gammaproteobacteria unclassified | unclassified | unclassified | unclassified |
| Otu023113 | 0.9457 | 1 | 0.972 | 0.001 | Bacteria | Proteobacteria | Gammaproteobacteria | Gammaproteobacteria unclassified | unclassified | unclassified | unclassified |
| Otu023079 | 1 | 0.6667 | 0.816 | 0.006 | Bacteria | Proteobacteria | Gammaproteobacteria | Gammaproteobacteria unclassified | unclassified | unclassified | unclassified |
| Otu022926 | 0.8933 | 1 | 0.945 | 0.003 | Bacteria | Proteobacteria | Gammaproteobacteria | Gammaproteobacteria unclassified | unclassified | unclassified | unclassified |
| Otu021402 | 1 | 1 | 1 | 0.001 | Bacteria | Proteobacteria | Gammaproteobacteria | Gammaproteobacteria unclassified | unclassified | unclassified | unclassified |
| Otu021371 | 0.8955 | 0.6667 | 0.773 | 0.018 | Bacteria | Proteobacteria | Gammaproteobacteria | Gammaproteobacteria unclassified | unclassified | unclassified | unclassified |
| Otu020934 | 0.872 | 1 | 0.934 | 0.005 | Bacteria | Proteobacteria | Gammaproteobacteria | Gammaproteobacteria unclassified | unclassified | unclassified | unclassified |
| Otu019193 | 0.9123 | 0.6667 | 0.78 | 0.009 | Bacteria | Proteobacteria | Gammaproteobacteria | Gammaproteobacteria unclassified | unclassified | unclassified | unclassified |
| Otu019162 | 0.892 | 1 | 0.944 | 0.004 | Bacteria | Proteobacteria | Gammaproteobacteria | Gammaproteobacteria unclassified | unclassified | unclassified | unclassified |
| Otu018090 | 0.9015 | 1 | 0.949 | 0.003 | Bacteria | Proteobacteria | Gammaproteobacteria | Gammaproteobacteria unclassified | unclassified | unclassified | unclassified |
| Otu018087 | 0.9565 | 1 | 0.978 | 0.001 | Bacteria | Proteobacteria | Gammaproteobacteria | Gammaproteobacteria unclassified | unclassified | unclassified | unclassified |
| Otu017137 | 0.9185 | 1 | 0.958 | 0.002 | Bacteria | Proteobacteria | Gammaproteobacteria | Gammaproteobacteria unclassified | unclassified | unclassified | unclassified |
| Otu017078 | 0.9148 | 1 | 0.956 | 0.002 | Bacteria | Proteobacteria | Gammaproteobacteria | Gammaproteobacteria unclassified | unclassified | unclassified | unclassified |
| Otu017014 | 0.9532 | 1 | 0.976 | 0.001 | Bacteria | Proteobacteria | Gammaproteobacteria | Gammaproteobacteria unclassified | unclassified | unclassified | unclassified |
| Otu016634 | 0.7614 | 1 | 0.873 | 0.018 | Bacteria | Proteobacteria | Gammaproteobacteria | Gammaproteobacteria unclassified | unclassified | unclassified | unclassified |
| Otu014775 | 0.9569 | 0.6667 | 0.799 | 0.006 | Bacteria | Proteobacteria | Gammaproteobacteria | Gammaproteobacteria unclassified | unclassified | unclassified | unclassified |
| Otu014468 | 0.9197 | 1 | 0.959 | 0.001 | Bacteria | Proteobacteria | Gammaproteobacteria | Gammaproteobacteria unclassified | unclassified | unclassified | unclassified |
| Otu014452 | 0.928 | 1 | 0.963 | 0.003 | Bacteria | Proteobacteria | Gammaproteobacteria | Gammaproteobacteria unclassified | unclassified | unclassified | unclassified |
| Otu014238 | 0.9752 | 1 | 0.988 | 0.001 | Bacteria | Proteobacteria | Gammaproteobacteria | Gammaproteobacteria unclassified | unclassified | unclassified | unclassified |
| Otu013840 | 0.9257 | 1 | 0.962 | 0.002 | Bacteria | Proteobacteria | Gammaproteobacteria | Gammaproteobacteria unclassified | unclassified | unclassified | unclassified |
| Otu013681 | 0.9391 | 1 | 0.969 | 0.001 | Bacteria | Proteobacteria | Gammaproteobacteria | Gammaproteobacteria unclassified | unclassified | unclassified | unclassified |
| Otu013578 | 0.9056 | 0.6667 | 0.777 | 0.023 | Bacteria | Proteobacteria | Gammaproteobacteria | Gammaproteobacteria unclassified | unclassified | unclassified | unclassified |
| Otu013576 | 0.9286 | 1 | 0.964 | 0.001 | Bacteria | Proteobacteria | Gammaproteobacteria | Gammaproteobacteria unclassified | unclassified | unclassified | unclassified |
| Otu013224 | 0.944 | 1 | 0.972 | 0.002 | Bacteria | Proteobacteria | Gammaproteobacteria | Gammaproteobacteria unclassified | unclassified | unclassified | unclassified |
| Otu012171 | 0.9297 | 1 | 0.964 | 0.002 | Bacteria | Proteobacteria | Gammaproteobacteria | Gammaproteobacteria unclassified | unclassified | unclassified | unclassified |
| Otu011857 | 0.9102 | 0.6667 | 0.779 | 0.027 | Bacteria | Proteobacteria | Gammaproteobacteria | Gammaproteobacteria unclassified | unclassified | unclassified | unclassified |
| Otu011841 | 0.9382 | 1 | 0.969 | 0.002 | Bacteria | Proteobacteria | Gammaproteobacteria | Gammaproteobacteria unclassified | unclassified | unclassified | unclassified |
| Otu011820 | 0.9699 | 0.6667 | 0.804 | 0.01 | Bacteria | Proteobacteria | Gammaproteobacteria | Gammaproteobacteria unclassified | unclassified | unclassified | unclassified |
| Otu011795 | 0.9299 | 1 | 0.964 | 0.001 | Bacteria | Proteobacteria | Gammaproteobacteria | Gammaproteobacteria unclassified | unclassified | unclassified | unclassified |
| Otu011705 | 0.8727 | 0.6667 | 0.763 | 0.016 | Bacteria | Proteobacteria | Gammaproteobacteria | Gammaproteobacteria unclassified | unclassified | unclassified | unclassified |
| Otu011675 | 0.9348 | 1 | 0.967 | 0.001 | Bacteria | Proteobacteria | Gammaproteobacteria | Gammaproteobacteria unclassified | unclassified | unclassified | unclassified |
| Otu011015 | 0.9009 | 0.6667 | 0.775 | 0.008 | Bacteria | Proteobacteria | Gammaproteobacteria | Gammaproteobacteria unclassified | unclassified | unclassified | unclassified |
| Otu010678 | 0.9371 | 1 | 0.968 | 0.003 | Bacteria | Proteobacteria | Gammaproteobacteria | Gammaproteobacteria unclassified | unclassified | unclassified | unclassified |
| Otu010576 | 0.9406 | 1 | 0.97 | 0.001 | Bacteria | Proteobacteria | Gammaproteobacteria | Gammaproteobacteria unclassified | unclassified | unclassified | unclassified |
| Otu009141 | 0.9472 | 1 | 0.973 | 0.001 | Bacteria | Proteobacteria | Gammaproteobacteria | Gammaproteobacteria unclassified | unclassified | unclassified | unclassified |
| Otu007611 | 0.8673 | 1 | 0.931 | 0.001 | Bacteria | Proteobacteria | Gammaproteobacteria | Gammaproteobacteria unclassified | unclassified | unclassified | unclassified |
| Otu006713 | 0.8862 | 1 | 0.941 | 0.001 | Bacteria | Proteobacteria | Gammaproteobacteria | Gammaproteobacteria unclassified | unclassified | unclassified | unclassified |
| Otu006520 | 0.692 | 1 | 0.832 | 0.03 | Bacteria | Proteobacteria | Gammaproteobacteria | Gammaproteobacteria unclassified | unclassified | unclassified | unclassified |
| Otu006211 | 0.9292 | 1 | 0.964 | 0.002 | Bacteria | Proteobacteria | Gammaproteobacteria | Gammaproteobacteria unclassified | unclassified | unclassified | unclassified |
| Otu005609 | 0.9387 | 0.6667 | 0.791 | 0.01 | Bacteria | Proteobacteria | Gammaproteobacteria | Gammaproteobacteria unclassified | unclassified | unclassified | unclassified |
| Otu005137 | 0.9568 | 0.6667 | 0.799 | 0.005 | Bacteria | Proteobacteria | Gammaproteobacteria | Gammaproteobacteria unclassified | unclassified | unclassified | unclassified |
| Otu004669 | 0.9258 | 1 | 0.962 | 0.001 | Bacteria | Proteobacteria | Gammaproteobacteria | Gammaproteobacteria unclassified | unclassified | unclassified | unclassified |

| | | | | | | | | | | | |
|---|---|---|---|---|---|---|---|---|---|---|---|
| Otu004634 | 0.8761 | 1 | 0.936 | 0.003 | Bacteria | Proteobacteria | Gammaproteobacteria | Gammaproteobacteria unclassified | unclassified | unclassified | unclassified |
| Otu003921 | 0.8679 | 0.6667 | 0.761 | 0.006 | Bacteria | Proteobacteria | Gammaproteobacteria | Gammaproteobacteria unclassified | unclassified | unclassified | unclassified |
| Otu003632 | 0.9242 | 1 | 0.961 | 0.002 | Bacteria | Proteobacteria | Gammaproteobacteria | Gammaproteobacteria unclassified | unclassified | unclassified | unclassified |
| Otu002707 | 0.8926 | 0.6667 | 0.771 | 0.015 | Bacteria | Proteobacteria | Gammaproteobacteria | Gammaproteobacteria unclassified | unclassified | unclassified | unclassified |
| Otu000723 | 0.9656 | 0.6667 | 0.802 | 0.006 | Bacteria | Proteobacteria | Gammaproteobacteria | Gammaproteobacteria unclassified | unclassified | unclassified | unclassified |
| Otu000165 | 0.9002 | 1 | 0.949 | 0.004 | Bacteria | Proteobacteria | Gammaproteobacteria | Gammaproteobacteria unclassified | unclassified | unclassified | unclassified |
| Otu117272 | 0.8835 | 0.6667 | 0.767 | 0.01 | Bacteria | Proteobacteria | Gammaproteobacteria | HTCC2188 | HTCC2089 | HTCC2089 unclassified | unclassified |
| Otu019709 | 0.8495 | 1 | 0.922 | 0.003 | Bacteria | Proteobacteria | Gammaproteobacteria | HTCC2188 | HTCC2089 | HTCC2089 unclassified | unclassified |
| Otu010239 | 0.8575 | 1 | 0.926 | 0.001 | Bacteria | Proteobacteria | Gammaproteobacteria | HTCC2188 | HTCC2089 | HTCC2089 unclassified | unclassified |
| Otu006193 | 0.9311 | 1 | 0.965 | 0.001 | Bacteria | Proteobacteria | Gammaproteobacteria | HTCC2188 | HTCC2089 | HTCC2089 unclassified | unclassified |
| Otu007369 | 0.8624 | 1 | 0.929 | 0.004 | Bacteria | Proteobacteria | Gammaproteobacteria | Legionellales | Endoecteinascidiaceae | Endoecteinascidiaceae unclassified | unclassified |
| Otu007465 | 0.8818 | 0.6667 | 0.767 | 0.018 | Bacteria | Proteobacteria | Gammaproteobacteria | Legionellales | Francisellaceae | Francisella | Francisella unclassified |
| Otu013176 | 0.8532 | 1 | 0.924 | 0.005 | Bacteria | Proteobacteria | Gammaproteobacteria | Legionellales | Legionellaceae | Legionellaceae unclassified | unclassified |
| Otu014309 | 0.7421 | 1 | 0.861 | 0.029 | Bacteria | Proteobacteria | Gammaproteobacteria | Oceanospirillales | Oceanospirillales unclassified | unclassified | unclassified |
| Otu033129 | 0.8298 | 0.6667 | 0.744 | 0.033 | Bacteria | Proteobacteria | Gammaproteobacteria | Oceanospirillales | Saccharospirillaceae | Reinekea | Reinekea unclassified |
| Otu045988 | 0.77 | 1 | 0.878 | 0.014 | Bacteria | Proteobacteria | Gammaproteobacteria | Thiotrichales | Piscirickettsiaceae | Piscirickettsiaceae unclassified | unclassified |
| Otu010163 | 0.7596 | 1 | 0.872 | 0.013 | Bacteria | Proteobacteria | Gammaproteobacteria | Thiotrichales | Piscirickettsiaceae | Piscirickettsiaceae unclassified | unclassified |
| Otu152185 | 0.9171 | 0.6667 | 0.782 | 0.009 | Bacteria | Proteobacteria | Gammaproteobacteria | Vibrionales | Vibrionaceae | Aliivibrio | Aliivibrio fischeri |
| Otu001070 | 0.8443 | 1 | 0.919 | 0.001 | Bacteria | Proteobacteria | Gammaproteobacteria | Vibrionales | Vibrionaceae | Aliivibrio | Aliivibrio fischeri |
| Otu019416 | 0.904 | 1 | 0.951 | 0.001 | Bacteria | Proteobacteria | Gammaproteobacteria | Vibrionales | Vibrionaceae | Photobacterium | Photobacterium angustum |
| Otu013979 | 0.9585 | 0.6667 | 0.799 | 0.008 | Bacteria | Proteobacteria | Gammaproteobacteria | Vibrionales | Vibrionaceae | Photobacterium | Photobacterium angustum |
| Otu005343 | 0.9196 | 1 | 0.959 | 0.001 | Bacteria | Proteobacteria | Gammaproteobacteria | Vibrionales | Vibrionaceae | Photobacterium | Photobacterium angustum |
| Otu057716 | 0.8404 | 0.6667 | 0.749 | 0.019 | Bacteria | Proteobacteria | Gammaproteobacteria | Vibrionales | Vibrionaceae | Vibrio | Vibrio aestuarianus |
| Otu084293 | 1 | 0.6667 | 0.816 | 0.005 | Bacteria | Proteobacteria | Gammaproteobacteria | Vibrionales | Vibrionaceae | Vibrio | Vibrio unclassified |
| Otu013980 | 0.9653 | 0.6667 | 0.802 | 0.006 | Bacteria | Proteobacteria | Gammaproteobacteria | Vibrionales | Vibrionaceae | Vibrio | Vibrio unclassified |
| Otu006737 | 0.8165 | 0.6667 | 0.738 | 0.023 | Bacteria | Proteobacteria | Gammaproteobacteria | Vibrionales | Vibrionaceae | Vibrio | Vibrio unclassified |
| Otu137044 | 0.8323 | 0.6667 | 0.745 | 0.028 | Bacteria | Proteobacteria | Proteobacteria unclassified | unclassified | unclassified | unclassified | unclassified |
| Otu137000 | 0.8624 | 0.6667 | 0.758 | 0.015 | Bacteria | Proteobacteria | Proteobacteria unclassified | unclassified | unclassified | unclassified | unclassified |
| Otu136079 | 0.9444 | 0.6667 | 0.793 | 0.015 | Bacteria | Proteobacteria | Proteobacteria unclassified | unclassified | unclassified | unclassified | unclassified |
| Otu080511 | 0.9336 | 0.6667 | 0.789 | 0.006 | Bacteria | Proteobacteria | Proteobacteria unclassified | unclassified | unclassified | unclassified | unclassified |
| Otu055256 | 0.9508 | 1 | 0.975 | 0.001 | Bacteria | Proteobacteria | Proteobacteria unclassified | unclassified | unclassified | unclassified | unclassified |
| Otu027696 | 0.9011 | 1 | 0.949 | 0.002 | Bacteria | Proteobacteria | Proteobacteria unclassified | unclassified | unclassified | unclassified | unclassified |
| Otu026987 | 0.8704 | 1 | 0.933 | 0.006 | Bacteria | Proteobacteria | Proteobacteria unclassified | unclassified | unclassified | unclassified | unclassified |
| Otu012514 | 1 | 0.6667 | 0.816 | 0.006 | Bacteria | Proteobacteria | Proteobacteria unclassified | unclassified | unclassified | unclassified | unclassified |
| Otu009612 | 0.8463 | 0.6667 | 0.751 | 0.032 | Bacteria | Proteobacteria | Proteobacteria unclassified | unclassified | unclassified | unclassified | unclassified |
| Otu006966 | 0.732 | 1 | 0.856 | 0.039 | Bacteria | Proteobacteria | Proteobacteria unclassified | unclassified | unclassified | unclassified | unclassified |
| Otu005366 | 1 | 0.6667 | 0.816 | 0.006 | Bacteria | Proteobacteria | Proteobacteria unclassified | unclassified | unclassified | unclassified | unclassified |
| Otu000965 | 0.7654 | 1 | 0.875 | 0.02 | Bacteria | Proteobacteria | Proteobacteria unclassified | unclassified | unclassified | unclassified | unclassified |
| Otu088133 | 0.9089 | 1 | 0.953 | 0.001 | Bacteria | SAR406 | AB16 | Arctic96B-7 | A714017 | SGSH944 | SGSH944 unclassified |
| Otu005177 | 0.9396 | 1 | 0.969 | 0.002 | Bacteria | Spirochaetes | Spirochaetes | Spirochaetes unclassified | unclassified | unclassified | unclassified |
| Otu015014 | 0.964 | 1 | 0.982 | 0.001 | Bacteria | unknown unclassified | unclassified | unclassified | unclassified | unclassified | unclassified |
| Otu015382 | 0.8421 | 1 | 0.918 | 0.005 | Bacteria | Verrucomicrobia | Opitutae | Opitutales | Opitutaceae | Opitutus | Opitutus unclassified |
| Otu035127 | 0.8452 | 0.6667 | 0.751 | 0.025 | Bacteria | Verrucomicrobia | Pedosphaerae | Pedosphaerales | Pedosphaerales unclassified | unclassified | unclassified |
| Otu005015 | 1 | 1 | 1 | 0.001 | Bacteria | Verrucomicrobia | Pedosphaerae | Pedosphaerales | Pedosphaerales unclassified | unclassified | unclassified |
| Otu009007 | 0.8454 | 1 | 0.919 | 0.001 | Bacteria | Verrucomicrobia | Verrucomicrobiae | Verrucomicrobiales | Verrucomicrobiaceae | Persicirhabdus | Persicirhabdus unclassified |
| Otu002659 | 1 | 0.6667 | 0.816 | 0.005 | Bacteria | Verrucomicrobia | Verrucomicrobiae | Verrucomicrobiales | Verrucomicrobiaceae | Persicirhabdus | Persicirhabdus unclassified |
| Otu072748 | 0.84 | 1 | 0.917 | 0.004 | Bacteria | Verrucomicrobia | Verrucomicrobiae | Verrucomicrobiales | Verrucomicrobiaceae | Rubritalea | Rubritalea unclassified |
| Otu026518 | 0.8133 | 0.6667 | 0.736 | 0.044 | Bacteria | Verrucomicrobia | Verrucomicrobiae | Verrucomicrobiales | Verrucomicrobiaceae | Verrucomicrobiaceae unclassified | unclassified |
| Otu008011 | 1 | 0.6667 | 0.816 | 0.006 | Bacteria | Verrucomicrobia | Verrucomicrobiae | Verrucomicrobiales | Verrucomicrobiaceae | Verrucomicrobiaceae unclassified | unclassified |
| Otu002477 | 0.811 | 1 | 0.901 | 0.017 | Bacteria | Verrucomicrobia | Verrucomicrobiae | Verrucomicrobiales | Verrucomicrobiaceae | Verrucomicrobiaceae unclassified | unclassified |

| **Otus** | ***Axinella infundibuliformis* upper twilight** | **Rest** | **stat** | **p value** | **Domain** | **Phylum** | **Class** | **Order** | **Family** | **Genus** | **Species** |
|---|---|---|---|---|---|---|---|---|---|---|---|
| Otu000729 | 0.8127 | 1 | 0.902 | 0.004 | Archaea | Crenarchaeota | Thaumarchaeota | Cenarchaeales | Cenarchaeaceae | Nitrosopumilus | Nitrosopumilus unclassified |
| Otu000742 | 0.9149 | 1 | 0.957 | 0.001 | Archaea | Crenarchaeota | Thaumarchaeota | Cenarchaeales | Cenarchaeaceae | Nitrosopumilus | Nitrosopumilus unclassified |
| Otu002418 | 0.9221 | 1 | 0.96 | 0.001 | Archaea | Crenarchaeota | Thaumarchaeota | Cenarchaeales | Cenarchaeaceae | Nitrosopumilus | Nitrosopumilus unclassified |
| Otu003072 | 0.9188 | 1 | 0.959 | 0.001 | Archaea | Crenarchaeota | Thaumarchaeota | Cenarchaeales | Cenarchaeaceae | Nitrosopumilus | Nitrosopumilus unclassified |
| Otu003584 | 0.7313 | 1 | 0.855 | 0.025 | Archaea | Crenarchaeota | Thaumarchaeota | Cenarchaeales | Cenarchaeaceae | Nitrosopumilus | Nitrosopumilus unclassified |
| Otu003602 | 0.7647 | 1 | 0.874 | 0.014 | Archaea | Crenarchaeota | Thaumarchaeota | Cenarchaeales | Cenarchaeaceae | Nitrosopumilus | Nitrosopumilus unclassified |
| Otu004972 | 0.8436 | 0.6667 | 0.75 | 0.017 | Archaea | Crenarchaeota | Thaumarchaeota | Cenarchaeales | Cenarchaeaceae | Nitrosopumilus | Nitrosopumilus unclassified |
| Otu005169 | 0.7379 | 1 | 0.859 | 0.027 | Archaea | Crenarchaeota | Thaumarchaeota | Cenarchaeales | Cenarchaeaceae | Nitrosopumilus | Nitrosopumilus unclassified |
| Otu006316 | 0.7632 | 1 | 0.874 | 0.018 | Archaea | Crenarchaeota | Thaumarchaeota | Cenarchaeales | Cenarchaeaceae | Nitrosopumilus | Nitrosopumilus unclassified |
| Otu006387 | 0.8056 | 1 | 0.898 | 0.007 | Archaea | Crenarchaeota | Thaumarchaeota | Cenarchaeales | Cenarchaeaceae | Nitrosopumilus | Nitrosopumilus unclassified |
| Otu006886 | 0.7554 | 1 | 0.869 | 0.024 | Archaea | Crenarchaeota | Thaumarchaeota | Cenarchaeales | Cenarchaeaceae | Nitrosopumilus | Nitrosopumilus unclassified |
| Otu007001 | 0.7439 | 1 | 0.862 | 0.029 | Archaea | Crenarchaeota | Thaumarchaeota | Cenarchaeales | Cenarchaeaceae | Nitrosopumilus | Nitrosopumilus unclassified |
| Otu007093 | 0.9048 | 0.6667 | 0.777 | 0.021 | Archaea | Crenarchaeota | Thaumarchaeota | Cenarchaeales | Cenarchaeaceae | Nitrosopumilus | Nitrosopumilus unclassified |
| Otu007812 | 0.7887 | 1 | 0.888 | 0.018 | Archaea | Crenarchaeota | Thaumarchaeota | Cenarchaeales | Cenarchaeaceae | Nitrosopumilus | Nitrosopumilus unclassified |
| Otu007911 | 0.7246 | 1 | 0.851 | 0.031 | Archaea | Crenarchaeota | Thaumarchaeota | Cenarchaeales | Cenarchaeaceae | Nitrosopumilus | Nitrosopumilus unclassified |
| Otu008196 | 0.7666 | 1 | 0.876 | 0.023 | Archaea | Crenarchaeota | Thaumarchaeota | Cenarchaeales | Cenarchaeaceae | Nitrosopumilus | Nitrosopumilus unclassified |
| Otu008208 | 0.7491 | 1 | 0.865 | 0.024 | Archaea | Crenarchaeota | Thaumarchaeota | Cenarchaeales | Cenarchaeaceae | Nitrosopumilus | Nitrosopumilus unclassified |
| Otu008876 | 0.8161 | 1 | 0.903 | 0.013 | Archaea | Crenarchaeota | Thaumarchaeota | Cenarchaeales | Cenarchaeaceae | Nitrosopumilus | Nitrosopumilus unclassified |
| Otu008996 | 0.9343 | 1 | 0.967 | 0.001 | Archaea | Crenarchaeota | Thaumarchaeota | Cenarchaeales | Cenarchaeaceae | Nitrosopumilus | Nitrosopumilus unclassified |
| Otu009043 | 0.8444 | 1 | 0.919 | 0.001 | Archaea | Crenarchaeota | Thaumarchaeota | Cenarchaeales | Cenarchaeaceae | Nitrosopumilus | Nitrosopumilus unclassified |
| Otu009079 | 0.7982 | 1 | 0.893 | 0.016 | Archaea | Crenarchaeota | Thaumarchaeota | Cenarchaeales | Cenarchaeaceae | Nitrosopumilus | Nitrosopumilus unclassified |
| Otu009086 | 0.7779 | 1 | 0.882 | 0.005 | Archaea | Crenarchaeota | Thaumarchaeota | Cenarchaeales | Cenarchaeaceae | Nitrosopumilus | Nitrosopumilus unclassified |
| Otu009308 | 0.8693 | 1 | 0.932 | 0.003 | Archaea | Crenarchaeota | Thaumarchaeota | Cenarchaeales | Cenarchaeaceae | Nitrosopumilus | Nitrosopumilus unclassified |
| Otu009768 | 0.7473 | 1 | 0.864 | 0.027 | Archaea | Crenarchaeota | Thaumarchaeota | Cenarchaeales | Cenarchaeaceae | Nitrosopumilus | Nitrosopumilus unclassified |
| Otu009843 | 0.9087 | 1 | 0.953 | 0.001 | Archaea | Crenarchaeota | Thaumarchaeota | Cenarchaeales | Cenarchaeaceae | Nitrosopumilus | Nitrosopumilus unclassified |
| Otu009849 | 0.7516 | 1 | 0.867 | 0.024 | Archaea | Crenarchaeota | Thaumarchaeota | Cenarchaeales | Cenarchaeaceae | Nitrosopumilus | Nitrosopumilus unclassified |
| Otu009854 | 0.7558 | 1 | 0.869 | 0.019 | Archaea | Crenarchaeota | Thaumarchaeota | Cenarchaeales | Cenarchaeaceae | Nitrosopumilus | Nitrosopumilus unclassified |
| Otu010111 | 0.8626 | 1 | 0.929 | 0.002 | Archaea | Crenarchaeota | Thaumarchaeota | Cenarchaeales | Cenarchaeaceae | Nitrosopumilus | Nitrosopumilus unclassified |
| Otu010311 | 0.7441 | 1 | 0.863 | 0.017 | Archaea | Crenarchaeota | Thaumarchaeota | Cenarchaeales | Cenarchaeaceae | Nitrosopumilus | Nitrosopumilus unclassified |
| Otu010426 | 0.8778 | 1 | 0.937 | 0.001 | Archaea | Crenarchaeota | Thaumarchaeota | Cenarchaeales | Cenarchaeaceae | Nitrosopumilus | Nitrosopumilus unclassified |
| Otu011440 | 0.7771 | 1 | 0.882 | 0.006 | Archaea | Crenarchaeota | Thaumarchaeota | Cenarchaeales | Cenarchaeaceae | Nitrosopumilus | Nitrosopumilus unclassified |
| Otu012295 | 0.9738 | 0.6667 | 0.806 | 0.005 | Archaea | Crenarchaeota | Thaumarchaeota | Cenarchaeales | Cenarchaeaceae | Nitrosopumilus | Nitrosopumilus unclassified |
| Otu012309 | 0.7201 | 1 | 0.849 | 0.037 | Archaea | Crenarchaeota | Thaumarchaeota | Cenarchaeales | Cenarchaeaceae | Nitrosopumilus | Nitrosopumilus unclassified |
| Otu012324 | 0.8256 | 1 | 0.909 | 0.004 | Archaea | Crenarchaeota | Thaumarchaeota | Cenarchaeales | Cenarchaeaceae | Nitrosopumilus | Nitrosopumilus unclassified |
| Otu012362 | 0.7114 | 1 | 0.843 | 0.035 | Archaea | Crenarchaeota | Thaumarchaeota | Cenarchaeales | Cenarchaeaceae | Nitrosopumilus | Nitrosopumilus unclassified |
| Otu012898 | 0.9528 | 1 | 0.976 | 0.001 | Archaea | Crenarchaeota | Thaumarchaeota | Cenarchaeales | Cenarchaeaceae | Nitrosopumilus | Nitrosopumilus unclassified |

| | | | | | | | | | | | |
|---|---|---|---|---|---|---|---|---|---|---|---|
| Otu013658 | 0.761 | 1 | 0.872 | 0.019 | Archaea | Crenarchaeota | Thaumarchaeota | Cenarchaeales | Cenarchaeaceae | Nitrosopumilus | Nitrosopumilus unclassified |
| Otu014541 | 0.8794 | 1 | 0.938 | 0.001 | Archaea | Crenarchaeota | Thaumarchaeota | Cenarchaeales | Cenarchaeaceae | Nitrosopumilus | Nitrosopumilus unclassified |
| Otu014589 | 0.7449 | 1 | 0.863 | 0.03 | Archaea | Crenarchaeota | Thaumarchaeota | Cenarchaeales | Cenarchaeaceae | Nitrosopumilus | Nitrosopumilus unclassified |
| Otu014638 | 0.8246 | 1 | 0.908 | 0.001 | Archaea | Crenarchaeota | Thaumarchaeota | Cenarchaeales | Cenarchaeaceae | Nitrosopumilus | Nitrosopumilus unclassified |
| Otu014951 | 0.7837 | 1 | 0.885 | 0.004 | Archaea | Crenarchaeota | Thaumarchaeota | Cenarchaeales | Cenarchaeaceae | Nitrosopumilus | Nitrosopumilus unclassified |
| Otu015114 | 0.9348 | 1 | 0.967 | 0.001 | Archaea | Crenarchaeota | Thaumarchaeota | Cenarchaeales | Cenarchaeaceae | Nitrosopumilus | Nitrosopumilus unclassified |
| Otu015156 | 0.9003 | 1 | 0.949 | 0.001 | Archaea | Crenarchaeota | Thaumarchaeota | Cenarchaeales | Cenarchaeaceae | Nitrosopumilus | Nitrosopumilus unclassified |
| Otu015370 | 0.6959 | 1 | 0.834 | 0.045 | Archaea | Crenarchaeota | Thaumarchaeota | Cenarchaeales | Cenarchaeaceae | Nitrosopumilus | Nitrosopumilus unclassified |
| Otu015681 | 0.7937 | 1 | 0.891 | 0.01 | Archaea | Crenarchaeota | Thaumarchaeota | Cenarchaeales | Cenarchaeaceae | Nitrosopumilus | Nitrosopumilus unclassified |
| Otu015690 | 0.794 | 1 | 0.891 | 0.015 | Archaea | Crenarchaeota | Thaumarchaeota | Cenarchaeales | Cenarchaeaceae | Nitrosopumilus | Nitrosopumilus unclassified |
| Otu015890 | 0.7891 | 1 | 0.888 | 0.01 | Archaea | Crenarchaeota | Thaumarchaeota | Cenarchaeales | Cenarchaeaceae | Nitrosopumilus | Nitrosopumilus unclassified |
| Otu016238 | 0.7786 | 1 | 0.882 | 0.02 | Archaea | Crenarchaeota | Thaumarchaeota | Cenarchaeales | Cenarchaeaceae | Nitrosopumilus | Nitrosopumilus unclassified |
| Otu016249 | 0.7989 | 1 | 0.894 | 0.01 | Archaea | Crenarchaeota | Thaumarchaeota | Cenarchaeales | Cenarchaeaceae | Nitrosopumilus | Nitrosopumilus unclassified |
| Otu017262 | 0.855 | 1 | 0.925 | 0.001 | Archaea | Crenarchaeota | Thaumarchaeota | Cenarchaeales | Cenarchaeaceae | Nitrosopumilus | Nitrosopumilus unclassified |
| Otu017786 | 0.893 | 1 | 0.945 | 0.001 | Archaea | Crenarchaeota | Thaumarchaeota | Cenarchaeales | Cenarchaeaceae | Nitrosopumilus | Nitrosopumilus unclassified |
| Otu018285 | 0.8306 | 1 | 0.911 | 0.011 | Archaea | Crenarchaeota | Thaumarchaeota | Cenarchaeales | Cenarchaeaceae | Nitrosopumilus | Nitrosopumilus unclassified |
| Otu018303 | 0.9234 | 0.6667 | 0.785 | 0.008 | Archaea | Crenarchaeota | Thaumarchaeota | Cenarchaeales | Cenarchaeaceae | Nitrosopumilus | Nitrosopumilus unclassified |
| Otu018394 | 1 | 1 | 1 | 0.001 | Archaea | Crenarchaeota | Thaumarchaeota | Cenarchaeales | Cenarchaeaceae | Nitrosopumilus | Nitrosopumilus unclassified |
| Otu018427 | 0.9218 | 1 | 0.96 | 0.001 | Archaea | Crenarchaeota | Thaumarchaeota | Cenarchaeales | Cenarchaeaceae | Nitrosopumilus | Nitrosopumilus unclassified |
| Otu018909 | 0.8227 | 0.6667 | 0.741 | 0.032 | Archaea | Crenarchaeota | Thaumarchaeota | Cenarchaeales | Cenarchaeaceae | Nitrosopumilus | Nitrosopumilus unclassified |
| Otu018973 | 0.8115 | 1 | 0.901 | 0.011 | Archaea | Crenarchaeota | Thaumarchaeota | Cenarchaeales | Cenarchaeaceae | Nitrosopumilus | Nitrosopumilus unclassified |
| Otu019001 | 0.7606 | 1 | 0.872 | 0.023 | Archaea | Crenarchaeota | Thaumarchaeota | Cenarchaeales | Cenarchaeaceae | Nitrosopumilus | Nitrosopumilus unclassified |
| Otu019019 | 0.7539 | 1 | 0.868 | 0.019 | Archaea | Crenarchaeota | Thaumarchaeota | Cenarchaeales | Cenarchaeaceae | Nitrosopumilus | Nitrosopumilus unclassified |
| Otu019079 | 0.7835 | 1 | 0.885 | 0.017 | Archaea | Crenarchaeota | Thaumarchaeota | Cenarchaeales | Cenarchaeaceae | Nitrosopumilus | Nitrosopumilus unclassified |
| Otu019258 | 0.7741 | 1 | 0.88 | 0.022 | Archaea | Crenarchaeota | Thaumarchaeota | Cenarchaeales | Cenarchaeaceae | Nitrosopumilus | Nitrosopumilus unclassified |
| Otu019361 | 0.7564 | 1 | 0.87 | 0.024 | Archaea | Crenarchaeota | Thaumarchaeota | Cenarchaeales | Cenarchaeaceae | Nitrosopumilus | Nitrosopumilus unclassified |
| Otu019405 | 0.7112 | 1 | 0.843 | 0.043 | Archaea | Crenarchaeota | Thaumarchaeota | Cenarchaeales | Cenarchaeaceae | Nitrosopumilus | Nitrosopumilus unclassified |
| Otu019487 | 0.7742 | 1 | 0.88 | 0.013 | Archaea | Crenarchaeota | Thaumarchaeota | Cenarchaeales | Cenarchaeaceae | Nitrosopumilus | Nitrosopumilus unclassified |
| Otu021176 | 0.934 | 1 | 0.966 | 0.001 | Archaea | Crenarchaeota | Thaumarchaeota | Cenarchaeales | Cenarchaeaceae | Nitrosopumilus | Nitrosopumilus unclassified |
| Otu021237 | 0.8632 | 1 | 0.929 | 0.001 | Archaea | Crenarchaeota | Thaumarchaeota | Cenarchaeales | Cenarchaeaceae | Nitrosopumilus | Nitrosopumilus unclassified |
| Otu021287 | 0.7069 | 1 | 0.841 | 0.039 | Archaea | Crenarchaeota | Thaumarchaeota | Cenarchaeales | Cenarchaeaceae | Nitrosopumilus | Nitrosopumilus unclassified |
| Otu021359 | 0.8042 | 1 | 0.897 | 0.019 | Archaea | Crenarchaeota | Thaumarchaeota | Cenarchaeales | Cenarchaeaceae | Nitrosopumilus | Nitrosopumilus unclassified |
| Otu021772 | 0.8118 | 1 | 0.901 | 0.013 | Archaea | Crenarchaeota | Thaumarchaeota | Cenarchaeales | Cenarchaeaceae | Nitrosopumilus | Nitrosopumilus unclassified |
| Otu021876 | 0.9236 | 1 | 0.961 | 0.001 | Archaea | Crenarchaeota | Thaumarchaeota | Cenarchaeales | Cenarchaeaceae | Nitrosopumilus | Nitrosopumilus unclassified |
| Otu022881 | 0.7851 | 1 | 0.886 | 0.017 | Archaea | Crenarchaeota | Thaumarchaeota | Cenarchaeales | Cenarchaeaceae | Nitrosopumilus | Nitrosopumilus unclassified |
| Otu022894 | 0.7878 | 1 | 0.888 | 0.008 | Archaea | Crenarchaeota | Thaumarchaeota | Cenarchaeales | Cenarchaeaceae | Nitrosopumilus | Nitrosopumilus unclassified |
| Otu023541 | 0.793 | 1 | 0.89 | 0.012 | Archaea | Crenarchaeota | Thaumarchaeota | Cenarchaeales | Cenarchaeaceae | Nitrosopumilus | Nitrosopumilus unclassified |
| Otu023649 | 0.8745 | 1 | 0.935 | 0.003 | Archaea | Crenarchaeota | Thaumarchaeota | Cenarchaeales | Cenarchaeaceae | Nitrosopumilus | Nitrosopumilus unclassified |
| Otu024771 | 0.7721 | 1 | 0.879 | 0.02 | Archaea | Crenarchaeota | Thaumarchaeota | Cenarchaeales | Cenarchaeaceae | Nitrosopumilus | Nitrosopumilus unclassified |
| Otu024888 | 0.9006 | 1 | 0.949 | 0.001 | Archaea | Crenarchaeota | Thaumarchaeota | Cenarchaeales | Cenarchaeaceae | Nitrosopumilus | Nitrosopumilus unclassified |
| Otu024937 | 0.7537 | 1 | 0.868 | 0.023 | Archaea | Crenarchaeota | Thaumarchaeota | Cenarchaeales | Cenarchaeaceae | Nitrosopumilus | Nitrosopumilus unclassified |
| Otu025046 | 0.9334 | 1 | 0.966 | 0.001 | Archaea | Crenarchaeota | Thaumarchaeota | Cenarchaeales | Cenarchaeaceae | Nitrosopumilus | Nitrosopumilus unclassified |
| Otu025067 | 0.8018 | 1 | 0.895 | 0.005 | Archaea | Crenarchaeota | Thaumarchaeota | Cenarchaeales | Cenarchaeaceae | Nitrosopumilus | Nitrosopumilus unclassified |
| Otu025204 | 0.9024 | 1 | 0.95 | 0.001 | Archaea | Crenarchaeota | Thaumarchaeota | Cenarchaeales | Cenarchaeaceae | Nitrosopumilus | Nitrosopumilus unclassified |
| Otu025402 | 0.8265 | 1 | 0.909 | 0.006 | Archaea | Crenarchaeota | Thaumarchaeota | Cenarchaeales | Cenarchaeaceae | Nitrosopumilus | Nitrosopumilus unclassified |
| Otu025926 | 0.8259 | 1 | 0.909 | 0.001 | Archaea | Crenarchaeota | Thaumarchaeota | Cenarchaeales | Cenarchaeaceae | Nitrosopumilus | Nitrosopumilus unclassified |
| Otu025945 | 0.9206 | 1 | 0.959 | 0.001 | Archaea | Crenarchaeota | Thaumarchaeota | Cenarchaeales | Cenarchaeaceae | Nitrosopumilus | Nitrosopumilus unclassified |
| Otu026415 | 0.8485 | 1 | 0.921 | 0.007 | Archaea | Crenarchaeota | Thaumarchaeota | Cenarchaeales | Cenarchaeaceae | Nitrosopumilus | Nitrosopumilus unclassified |
| Otu026519 | 0.8455 | 1 | 0.919 | 0.008 | Archaea | Crenarchaeota | Thaumarchaeota | Cenarchaeales | Cenarchaeaceae | Nitrosopumilus | Nitrosopumilus unclassified |
| Otu026571 | 0.9307 | 0.6667 | 0.788 | 0.019 | Archaea | Crenarchaeota | Thaumarchaeota | Cenarchaeales | Cenarchaeaceae | Nitrosopumilus | Nitrosopumilus unclassified |
| Otu027885 | 0.8168 | 0.6667 | 0.738 | 0.043 | Archaea | Crenarchaeota | Thaumarchaeota | Cenarchaeales | Cenarchaeaceae | Nitrosopumilus | Nitrosopumilus unclassified |
| Otu030901 | 0.7802 | 1 | 0.883 | 0.02 | Archaea | Crenarchaeota | Thaumarchaeota | Cenarchaeales | Cenarchaeaceae | Nitrosopumilus | Nitrosopumilus unclassified |
| Otu031597 | 0.7997 | 1 | 0.894 | 0.005 | Archaea | Crenarchaeota | Thaumarchaeota | Cenarchaeales | Cenarchaeaceae | Nitrosopumilus | Nitrosopumilus unclassified |
| Otu035248 | 0.85 | 1 | 0.922 | 0.009 | Archaea | Crenarchaeota | Thaumarchaeota | Cenarchaeales | Cenarchaeaceae | Nitrosopumilus | Nitrosopumilus unclassified |
| Otu035274 | 0.8344 | 1 | 0.913 | 0.003 | Archaea | Crenarchaeota | Thaumarchaeota | Cenarchaeales | Cenarchaeaceae | Nitrosopumilus | Nitrosopumilus unclassified |
| Otu036684 | 0.949 | 1 | 0.974 | 0.001 | Archaea | Crenarchaeota | Thaumarchaeota | Cenarchaeales | Cenarchaeaceae | Nitrosopumilus | Nitrosopumilus unclassified |
| Otu037189 | 0.8805 | 1 | 0.938 | 0.003 | Archaea | Crenarchaeota | Thaumarchaeota | Cenarchaeales | Cenarchaeaceae | Nitrosopumilus | Nitrosopumilus unclassified |
| Otu037263 | 0.8719 | 1 | 0.934 | 0.003 | Archaea | Crenarchaeota | Thaumarchaeota | Cenarchaeales | Cenarchaeaceae | Nitrosopumilus | Nitrosopumilus unclassified |
| Otu037287 | 0.796 | 1 | 0.892 | 0.013 | Archaea | Crenarchaeota | Thaumarchaeota | Cenarchaeales | Cenarchaeaceae | Nitrosopumilus | Nitrosopumilus unclassified |
| Otu039128 | 0.9294 | 1 | 0.964 | 0.001 | Archaea | Crenarchaeota | Thaumarchaeota | Cenarchaeales | Cenarchaeaceae | Nitrosopumilus | Nitrosopumilus unclassified |
| Otu039544 | 0.9294 | 1 | 0.964 | 0.001 | Archaea | Crenarchaeota | Thaumarchaeota | Cenarchaeales | Cenarchaeaceae | Nitrosopumilus | Nitrosopumilus unclassified |
| Otu040165 | 0.9471 | 1 | 0.973 | 0.001 | Archaea | Crenarchaeota | Thaumarchaeota | Cenarchaeales | Cenarchaeaceae | Nitrosopumilus | Nitrosopumilus unclassified |
| Otu040719 | 0.9178 | 1 | 0.958 | 0.001 | Archaea | Crenarchaeota | Thaumarchaeota | Cenarchaeales | Cenarchaeaceae | Nitrosopumilus | Nitrosopumilus unclassified |
| Otu043669 | 0.858 | 0.6667 | 0.756 | 0.026 | Archaea | Crenarchaeota | Thaumarchaeota | Cenarchaeales | Cenarchaeaceae | Nitrosopumilus | Nitrosopumilus unclassified |
| Otu046395 | 0.8294 | 1 | 0.911 | 0.003 | Archaea | Crenarchaeota | Thaumarchaeota | Cenarchaeales | Cenarchaeaceae | Nitrosopumilus | Nitrosopumilus unclassified |
| Otu047258 | 1 | 1 | 1 | 0.001 | Archaea | Crenarchaeota | Thaumarchaeota | Cenarchaeales | Cenarchaeaceae | Nitrosopumilus | Nitrosopumilus unclassified |
| Otu047334 | 0.8329 | 1 | 0.913 | 0.007 | Archaea | Crenarchaeota | Thaumarchaeota | Cenarchaeales | Cenarchaeaceae | Nitrosopumilus | Nitrosopumilus unclassified |
| Otu047355 | 0.8217 | 1 | 0.906 | 0.002 | Archaea | Crenarchaeota | Thaumarchaeota | Cenarchaeales | Cenarchaeaceae | Nitrosopumilus | Nitrosopumilus unclassified |
| Otu049309 | 0.8355 | 1 | 0.914 | 0.003 | Archaea | Crenarchaeota | Thaumarchaeota | Cenarchaeales | Cenarchaeaceae | Nitrosopumilus | Nitrosopumilus unclassified |
| Otu060145 | 0.9351 | 1 | 0.967 | 0.001 | Archaea | Crenarchaeota | Thaumarchaeota | Cenarchaeales | Cenarchaeaceae | Nitrosopumilus | Nitrosopumilus unclassified |
| Otu067769 | 0.9413 | 1 | 0.97 | 0.001 | Archaea | Crenarchaeota | Thaumarchaeota | Cenarchaeales | Cenarchaeaceae | Nitrosopumilus | Nitrosopumilus unclassified |
| Otu068072 | 0.6591 | 1 | 0.812 | 0.044 | Archaea | Crenarchaeota | Thaumarchaeota | Cenarchaeales | Cenarchaeaceae | Nitrosopumilus | Nitrosopumilus unclassified |
| Otu094777 | 0.759 | 1 | 0.871 | 0.023 | Archaea | Crenarchaeota | Thaumarchaeota | Cenarchaeales | Cenarchaeaceae | Nitrosopumilus | Nitrosopumilus unclassified |
| Otu001374 | 0.832 | 1 | 0.912 | 0.007 | Bacteria | Actinobacteria | Acidimicrobiia | Acidimicrobiales | TK06 | TK06 unclassified | unclassified |
| Otu003242 | 0.8409 | 1 | 0.917 | 0.005 | Bacteria | Bacteria unclassified | unclassified | unclassified | unclassified | unclassified | unclassified |
| Otu012705 | 0.9143 | 1 | 0.956 | 0.002 | Bacteria | Bacteria unclassified | unclassified | unclassified | unclassified | unclassified | unclassified |
| Otu028476 | 0.9265 | 1 | 0.963 | 0.001 | Bacteria | Bacteria unclassified | unclassified | unclassified | unclassified | unclassified | unclassified |
| Otu061365 | 0.9413 | 1 | 0.97 | 0.001 | Bacteria | Bacteria unclassified | unclassified | unclassified | unclassified | unclassified | unclassified |
| Otu272760 | 0.9473 | 1 | 0.973 | 0.002 | Bacteria | Bacteria unclassified | unclassified | unclassified | unclassified | unclassified | unclassified |
| Otu003666 | 0.8335 | 1 | 0.913 | 0.005 | Bacteria | Bacteroidetes | Bacteroidetes unclassified | unclassified | unclassified | unclassified | unclassified |
| Otu004011 | 0.8437 | 1 | 0.919 | 0.004 | Bacteria | Bacteroidetes | Bacteroidetes unclassified | unclassified | unclassified | unclassified | unclassified |
| Otu000144 | 0.897 | 1 | 0.947 | 0.001 | Bacteria | Nitrospirae | Nitrospira | Nitrospirales | Nitrospiraceae | Nitrospiraceae unclassified | unclassified |
| Otu000215 | 0.956 | 1 | 0.978 | 0.001 | Bacteria | Nitrospirae | Nitrospira | Nitrospirales | Nitrospiraceae | Nitrospiraceae unclassified | unclassified |
| Otu003184 | 0.8961 | 1 | 0.947 | 0.001 | Bacteria | Nitrospirae | Nitrospira | Nitrospirales | Nitrospiraceae | Nitrospiraceae unclassified | unclassified |
| Otu043335 | 0.9112 | 1 | 0.955 | 0.001 | Bacteria | Nitrospirae | Nitrospira | Nitrospirales | Nitrospiraceae | Nitrospiraceae unclassified | unclassified |
| Otu060131 | 0.8722 | 1 | 0.934 | 0.004 | Bacteria | Nitrospirae | Nitrospira | Nitrospirales | Nitrospiraceae | Nitrospiraceae unclassified | unclassified |
| Otu013185 | 0.8148 | 1 | 0.903 | 0.01 | Bacteria | Planctomycetes | Phycisphaerae | Phycisphaerales | Phycisphaerales unclassified | unclassified | unclassified |
| Otu006577 | 0.905 | 1 | 0.951 | 0.001 | Bacteria | Proteobacteria | Alphaproteobacteria | Alphaproteobacteria unclassified | unclassified | unclassified | unclassified |

| | | | | | | | | | | | |
|---|---|---|---|---|---|---|---|---|---|---|---|
| Otu056672 | 0.9561 | 0.6667 | 0.798 | 0.005 | Bacteria | Proteobacteria | Alphaproteobacteria | Alphaproteobacteria unclassified | unclassified | unclassified | unclassified |
| Otu160075 | 0.9104 | 0.6667 | 0.779 | 0.01 | Bacteria | Proteobacteria | Alphaproteobacteria | Rhodobacterales | Rhodobacteraceae | Rhodobacteraceae unclassified | unclassified |
| Otu014768 | 0.8489 | 0.6667 | 0.752 | 0.039 | Bacteria | Proteobacteria | Betaproteobacteria | Rhodocyclales | Rhodocyclaceae | Rhodocyclaceae unclassified | unclassified |
| Otu010354 | 0.9175 | 0.6667 | 0.782 | 0.008 | Bacteria | Proteobacteria | Deltaproteobacteria | Bdellovibrionales | Bdellovibrionaceae | Bdellovibrio | Bdellovibrio unclassified |
| Otu008239 | 0.9699 | 0.6667 | 0.804 | 0.012 | Bacteria | Proteobacteria | Deltaproteobacteria | Desulfobacterales | Desulfobacteraceae | Desulfococcus | Desulfococcus unclassified |
| Otu020665 | 1 | 0.6667 | 0.816 | 0.012 | Bacteria | Proteobacteria | Deltaproteobacteria | Myxococcales | Myxococcales unclassified | unclassified | unclassified |
| Otu004564 | 0.6341 | 1 | 0.796 | 0.034 | Bacteria | Proteobacteria | Deltaproteobacteria | Sva0853 | SAR324 | SAR324 unclassified | unclassified |
| Otu041365 | 1 | 0.6667 | 0.816 | 0.007 | Bacteria | Proteobacteria | Gammaproteobacteria | Alteromonadales | Alteromonadales unclassified | unclassified | unclassified |
| Otu031704 | 0.8858 | 1 | 0.941 | 0.002 | Bacteria | Proteobacteria | Gammaproteobacteria | Enterobacteriales | Enterobacteriaceae | Enterobacteriaceae unclassified | unclassified |
| Otu000165 | 0.8677 | 1 | 0.932 | 0.003 | Bacteria | Proteobacteria | Gammaproteobacteria | Gammaproteobacteria unclassified | unclassified | unclassified | unclassified |
| Otu002707 | 0.8851 | 1 | 0.941 | 0.002 | Bacteria | Proteobacteria | Gammaproteobacteria | Gammaproteobacteria unclassified | unclassified | unclassified | unclassified |
| Otu003632 | 0.8091 | 1 | 0.9 | 0.008 | Bacteria | Proteobacteria | Gammaproteobacteria | Gammaproteobacteria unclassified | unclassified | unclassified | unclassified |
| Otu004669 | 0.8368 | 1 | 0.915 | 0.005 | Bacteria | Proteobacteria | Gammaproteobacteria | Gammaproteobacteria unclassified | unclassified | unclassified | unclassified |
| Otu006211 | 0.8298 | 1 | 0.911 | 0.004 | Bacteria | Proteobacteria | Gammaproteobacteria | Gammaproteobacteria unclassified | unclassified | unclassified | unclassified |
| Otu007601 | 0.8616 | 0.6667 | 0.758 | 0.032 | Bacteria | Proteobacteria | Gammaproteobacteria | Gammaproteobacteria unclassified | unclassified | unclassified | unclassified |
| Otu007717 | 0.8753 | 0.6667 | 0.764 | 0.017 | Bacteria | Proteobacteria | Gammaproteobacteria | Gammaproteobacteria unclassified | unclassified | unclassified | unclassified |
| Otu010678 | 0.8112 | 0.6667 | 0.735 | 0.039 | Bacteria | Proteobacteria | Gammaproteobacteria | Gammaproteobacteria unclassified | unclassified | unclassified | unclassified |
| Otu011015 | 0.8756 | 1 | 0.936 | 0.002 | Bacteria | Proteobacteria | Gammaproteobacteria | Gammaproteobacteria unclassified | unclassified | unclassified | unclassified |
| Otu011211 | 0.9669 | 1 | 0.983 | 0.001 | Bacteria | Proteobacteria | Gammaproteobacteria | Gammaproteobacteria unclassified | unclassified | unclassified | unclassified |
| Otu011675 | 0.817 | 1 | 0.904 | 0.005 | Bacteria | Proteobacteria | Gammaproteobacteria | Gammaproteobacteria unclassified | unclassified | unclassified | unclassified |
| Otu011705 | 0.9033 | 1 | 0.95 | 0.002 | Bacteria | Proteobacteria | Gammaproteobacteria | Gammaproteobacteria unclassified | unclassified | unclassified | unclassified |
| Otu011735 | 0.8771 | 0.6667 | 0.765 | 0.023 | Bacteria | Proteobacteria | Gammaproteobacteria | Gammaproteobacteria unclassified | unclassified | unclassified | unclassified |
| Otu011795 | 0.8283 | 1 | 0.91 | 0.004 | Bacteria | Proteobacteria | Gammaproteobacteria | Gammaproteobacteria unclassified | unclassified | unclassified | unclassified |
| Otu011841 | 0.8084 | 1 | 0.899 | 0.005 | Bacteria | Proteobacteria | Gammaproteobacteria | Gammaproteobacteria unclassified | unclassified | unclassified | unclassified |
| Otu011857 | 0.8633 | 1 | 0.929 | 0.002 | Bacteria | Proteobacteria | Gammaproteobacteria | Gammaproteobacteria unclassified | unclassified | unclassified | unclassified |
| Otu012171 | 0.8287 | 1 | 0.91 | 0.004 | Bacteria | Proteobacteria | Gammaproteobacteria | Gammaproteobacteria unclassified | unclassified | unclassified | unclassified |
| Otu013224 | 0.7915 | 0.6667 | 0.726 | 0.05 | Bacteria | Proteobacteria | Gammaproteobacteria | Gammaproteobacteria unclassified | unclassified | unclassified | unclassified |
| Otu013576 | 0.8311 | 1 | 0.912 | 0.005 | Bacteria | Proteobacteria | Gammaproteobacteria | Gammaproteobacteria unclassified | unclassified | unclassified | unclassified |
| Otu013578 | 0.8368 | 1 | 0.915 | 0.004 | Bacteria | Proteobacteria | Gammaproteobacteria | Gammaproteobacteria unclassified | unclassified | unclassified | unclassified |
| Otu013681 | 0.7714 | 1 | 0.878 | 0.01 | Bacteria | Proteobacteria | Gammaproteobacteria | Gammaproteobacteria unclassified | unclassified | unclassified | unclassified |
| Otu013840 | 0.837 | 1 | 0.915 | 0.004 | Bacteria | Proteobacteria | Gammaproteobacteria | Gammaproteobacteria unclassified | unclassified | unclassified | unclassified |
| Otu014452 | 0.8324 | 1 | 0.912 | 0.004 | Bacteria | Proteobacteria | Gammaproteobacteria | Gammaproteobacteria unclassified | unclassified | unclassified | unclassified |
| Otu017014 | 0.7585 | 1 | 0.871 | 0.005 | Bacteria | Proteobacteria | Gammaproteobacteria | Gammaproteobacteria unclassified | unclassified | unclassified | unclassified |
| Otu017078 | 0.8563 | 1 | 0.925 | 0.005 | Bacteria | Proteobacteria | Gammaproteobacteria | Gammaproteobacteria unclassified | unclassified | unclassified | unclassified |
| Otu017137 | 0.8502 | 1 | 0.922 | 0.004 | Bacteria | Proteobacteria | Gammaproteobacteria | Gammaproteobacteria unclassified | unclassified | unclassified | unclassified |
| Otu017835 | 1 | 0.6667 | 0.816 | 0.007 | Bacteria | Proteobacteria | Gammaproteobacteria | Gammaproteobacteria unclassified | unclassified | unclassified | unclassified |
| Otu018090 | 0.8555 | 1 | 0.925 | 0.004 | Bacteria | Proteobacteria | Gammaproteobacteria | Gammaproteobacteria unclassified | unclassified | unclassified | unclassified |
| Otu019162 | 0.8857 | 1 | 0.941 | 0.005 | Bacteria | Proteobacteria | Gammaproteobacteria | Gammaproteobacteria unclassified | unclassified | unclassified | unclassified |
| Otu020934 | 0.9038 | 1 | 0.951 | 0.003 | Bacteria | Proteobacteria | Gammaproteobacteria | Gammaproteobacteria unclassified | unclassified | unclassified | unclassified |
| Otu022926 | 0.8843 | 1 | 0.94 | 0.002 | Bacteria | Proteobacteria | Gammaproteobacteria | Gammaproteobacteria unclassified | unclassified | unclassified | unclassified |
| Otu023113 | 0.786 | 1 | 0.887 | 0.005 | Bacteria | Proteobacteria | Gammaproteobacteria | Gammaproteobacteria unclassified | unclassified | unclassified | unclassified |
| Otu023864 | 0.8759 | 1 | 0.936 | 0.004 | Bacteria | Proteobacteria | Gammaproteobacteria | Gammaproteobacteria unclassified | unclassified | unclassified | unclassified |
| Otu024324 | 0.851 | 1 | 0.923 | 0.004 | Bacteria | Proteobacteria | Gammaproteobacteria | Gammaproteobacteria unclassified | unclassified | unclassified | unclassified |
| Otu029599 | 0.7135 | 1 | 0.845 | 0.027 | Bacteria | Proteobacteria | Gammaproteobacteria | Gammaproteobacteria unclassified | unclassified | unclassified | unclassified |
| Otu030672 | 0.9046 | 1 | 0.951 | 0.004 | Bacteria | Proteobacteria | Gammaproteobacteria | Gammaproteobacteria unclassified | unclassified | unclassified | unclassified |
| Otu030879 | 0.9064 | 1 | 0.952 | 0.004 | Bacteria | Proteobacteria | Gammaproteobacteria | Gammaproteobacteria unclassified | unclassified | unclassified | unclassified |
| Otu032375 | 0.8798 | 1 | 0.938 | 0.002 | Bacteria | Proteobacteria | Gammaproteobacteria | Gammaproteobacteria unclassified | unclassified | unclassified | unclassified |
| Otu032393 | 0.9402 | 0.6667 | 0.792 | 0.01 | Bacteria | Proteobacteria | Gammaproteobacteria | Gammaproteobacteria unclassified | unclassified | unclassified | unclassified |
| Otu042095 | 0.9588 | 0.6667 | 0.799 | 0.005 | Bacteria | Proteobacteria | Gammaproteobacteria | Gammaproteobacteria unclassified | unclassified | unclassified | unclassified |
| Otu044173 | 0.8588 | 0.6667 | 0.757 | 0.025 | Bacteria | Proteobacteria | Gammaproteobacteria | Gammaproteobacteria unclassified | unclassified | unclassified | unclassified |
| Otu052808 | 0.8713 | 1 | 0.933 | 0.004 | Bacteria | Proteobacteria | Gammaproteobacteria | Gammaproteobacteria unclassified | unclassified | unclassified | unclassified |
| Otu053648 | 0.7744 | 0.6667 | 0.719 | 0.05 | Bacteria | Proteobacteria | Gammaproteobacteria | Gammaproteobacteria unclassified | unclassified | unclassified | unclassified |
| Otu053691 | 0.8039 | 0.6667 | 0.732 | 0.04 | Bacteria | Proteobacteria | Gammaproteobacteria | Gammaproteobacteria unclassified | unclassified | unclassified | unclassified |
| Otu054125 | 0.8314 | 0.6667 | 0.744 | 0.039 | Bacteria | Proteobacteria | Gammaproteobacteria | Gammaproteobacteria unclassified | unclassified | unclassified | unclassified |
| Otu057077 | 0.7748 | 1 | 0.88 | 0.005 | Bacteria | Proteobacteria | Gammaproteobacteria | Gammaproteobacteria unclassified | unclassified | unclassified | unclassified |
| Otu057808 | 0.7797 | 1 | 0.883 | 0.005 | Bacteria | Proteobacteria | Gammaproteobacteria | Gammaproteobacteria unclassified | unclassified | unclassified | unclassified |
| Otu062765 | 0.9345 | 0.6667 | 0.789 | 0.02 | Bacteria | Proteobacteria | Gammaproteobacteria | Gammaproteobacteria unclassified | unclassified | unclassified | unclassified |
| Otu063030 | 0.8176 | 1 | 0.904 | 0.005 | Bacteria | Proteobacteria | Gammaproteobacteria | Gammaproteobacteria unclassified | unclassified | unclassified | unclassified |
| Otu063692 | 0.8101 | 1 | 0.9 | 0.005 | Bacteria | Proteobacteria | Gammaproteobacteria | Gammaproteobacteria unclassified | unclassified | unclassified | unclassified |
| Otu063716 | 0.9033 | 1 | 0.95 | 0.002 | Bacteria | Proteobacteria | Gammaproteobacteria | Gammaproteobacteria unclassified | unclassified | unclassified | unclassified |
| Otu065662 | 0.8186 | 1 | 0.905 | 0.005 | Bacteria | Proteobacteria | Gammaproteobacteria | Gammaproteobacteria unclassified | unclassified | unclassified | unclassified |
| Otu066273 | 0.8641 | 1 | 0.93 | 0.004 | Bacteria | Proteobacteria | Gammaproteobacteria | Gammaproteobacteria unclassified | unclassified | unclassified | unclassified |
| Otu108091 | 0.83 | 0.6667 | 0.744 | 0.04 | Bacteria | Proteobacteria | Gammaproteobacteria | Gammaproteobacteria unclassified | unclassified | unclassified | unclassified |
| Otu109528 | 0.858 | 1 | 0.926 | 0.004 | Bacteria | Proteobacteria | Gammaproteobacteria | Gammaproteobacteria unclassified | unclassified | unclassified | unclassified |
| Otu109701 | 0.9214 | 0.6667 | 0.784 | 0.021 | Bacteria | Proteobacteria | Gammaproteobacteria | Gammaproteobacteria unclassified | unclassified | unclassified | unclassified |
| Otu110251 | 0.8734 | 0.6667 | 0.763 | 0.012 | Bacteria | Proteobacteria | Gammaproteobacteria | Gammaproteobacteria unclassified | unclassified | unclassified | unclassified |
| Otu110337 | 0.9098 | 0.6667 | 0.779 | 0.007 | Bacteria | Proteobacteria | Gammaproteobacteria | Gammaproteobacteria unclassified | unclassified | unclassified | unclassified |
| Otu110482 | 0.9464 | 1 | 0.973 | 0.002 | Bacteria | Proteobacteria | Gammaproteobacteria | Gammaproteobacteria unclassified | unclassified | unclassified | unclassified |
| Otu111028 | 0.8874 | 1 | 0.942 | 0.002 | Bacteria | Proteobacteria | Gammaproteobacteria | Gammaproteobacteria unclassified | unclassified | unclassified | unclassified |
| Otu120747 | 0.8193 | 1 | 0.905 | 0.005 | Bacteria | Proteobacteria | Gammaproteobacteria | Gammaproteobacteria unclassified | unclassified | unclassified | unclassified |
| Otu121434 | 0.7829 | 0.6667 | 0.722 | 0.038 | Bacteria | Proteobacteria | Gammaproteobacteria | Gammaproteobacteria unclassified | unclassified | unclassified | unclassified |
| Otu121803 | 0.8925 | 1 | 0.945 | 0.002 | Bacteria | Proteobacteria | Gammaproteobacteria | Gammaproteobacteria unclassified | unclassified | unclassified | unclassified |
| Otu121900 | 0.8559 | 1 | 0.925 | 0.005 | Bacteria | Proteobacteria | Gammaproteobacteria | Gammaproteobacteria unclassified | unclassified | unclassified | unclassified |
| Otu123201 | 0.8167 | 1 | 0.904 | 0.005 | Bacteria | Proteobacteria | Gammaproteobacteria | Gammaproteobacteria unclassified | unclassified | unclassified | unclassified |
| Otu124295 | 0.8473 | 1 | 0.92 | 0.004 | Bacteria | Proteobacteria | Gammaproteobacteria | Gammaproteobacteria unclassified | unclassified | unclassified | unclassified |
| Otu126595 | 0.8387 | 1 | 0.916 | 0.005 | Bacteria | Proteobacteria | Gammaproteobacteria | Gammaproteobacteria unclassified | unclassified | unclassified | unclassified |
| Otu127072 | 0.8097 | 1 | 0.9 | 0.005 | Bacteria | Proteobacteria | Gammaproteobacteria | Gammaproteobacteria unclassified | unclassified | unclassified | unclassified |
| Otu128129 | 0.8403 | 1 | 0.917 | 0.005 | Bacteria | Proteobacteria | Gammaproteobacteria | Gammaproteobacteria unclassified | unclassified | unclassified | unclassified |
| Otu217528 | 0.8635 | 1 | 0.929 | 0.004 | Bacteria | Proteobacteria | Gammaproteobacteria | Gammaproteobacteria unclassified | unclassified | unclassified | unclassified |
| Otu217541 | 0.9393 | 0.6667 | 0.791 | 0.013 | Bacteria | Proteobacteria | Gammaproteobacteria | Gammaproteobacteria unclassified | unclassified | unclassified | unclassified |
| Otu251196 | 0.9419 | 0.6667 | 0.792 | 0.012 | Bacteria | Proteobacteria | Gammaproteobacteria | Gammaproteobacteria unclassified | unclassified | unclassified | unclassified |
| Otu259625 | 0.9129 | 1 | 0.955 | 0.004 | Bacteria | Proteobacteria | Gammaproteobacteria | Gammaproteobacteria unclassified | unclassified | unclassified | unclassified |
| Otu123464 | 0.8125 | 1 | 0.901 | 0.015 | Bacteria | Proteobacteria | Gammaproteobacteria | Thiohalorhabdales | Thiohalorhabdaceae | Thiohalorhabdaceae unclassified | unclassified |
| Otu013073 | 0.9016 | 0.6667 | 0.775 | 0.023 | Bacteria | Proteobacteria | Gammaproteobacteria | Thiotrichales | Piscirickettsiaceae | Piscirickettsiaceae unclassified | unclassified |
| Otu004696 | 0.9563 | 0.6667 | 0.798 | 0.007 | Bacteria | Proteobacteria | Proteobacteria unclassified | unclassified | unclassified | unclassified | unclassified |
| Otu011060 | 0.9372 | 0.6667 | 0.79 | 0.018 | Bacteria | Proteobacteria | Proteobacteria unclassified | unclassified | unclassified | unclassified | unclassified |
| Otu020585 | 0.9771 | 1 | 0.988 | 0.001 | Bacteria | Proteobacteria | Proteobacteria unclassified | unclassified | unclassified | unclassified | unclassified |
| Otu026987 | 0.9051 | 1 | 0.951 | 0.001 | Bacteria | Proteobacteria | Proteobacteria unclassified | unclassified | unclassified | unclassified | unclassified |

| | | | | | | | | | | | | |
|---|---|---|---|---|---|---|---|---|---|---|---|---|
| Otu027696 | 0.8754 | 1 | 0.936 | 0.005 | Bacteria | Proteobacteria | Proteobacteria unclassified | unclassified | unclassified | unclassified | unclassified |
| Otu063109 | 1 | 1 | 1 | 0.001 | Bacteria | Proteobacteria | Proteobacteria unclassified | unclassified | unclassified | unclassified | unclassified |
| Otu137000 | 0.9108 | 1 | 0.954 | 0.001 | Bacteria | Proteobacteria | Proteobacteria unclassified | unclassified | unclassified | unclassified | unclassified |
| Otu005177 | 0.8045 | 1 | 0.897 | 0.004 | Bacteria | Spirochaetes | Spirochaetes | Spirochaetes unclassified | unclassified | unclassified | unclassified |

**Suppl. Table 4** Pairwise comparisons of mean group dispersions of the permutation test for homogeneity of multivariate dispersions (permanova). For each taxonomic rank & relative abundance and presence / absence OTUs pairwise comparisons with observed *p*-value below diagonal, permuted *p*-value above diagonal are given for each sample triplicate.

| Tax Rank | Sponge hosts | *A. infundibuliformis* | *Axinella* sp. | *G. barretti* | *H. panicea* | seawater | *M. lingua* | *M. rosacea* | *P. ventilabrum* |
|---|---|---|---|---|---|---|---|---|---|
| Otu relabund | *A. infundibuliformis* | - | 0.1988 | **0.0480** | 0.2088 | **0.0170** | 0.1518 | 0.1449 | 0.0799 |
| | *Axinella* sp. | 0.2011 | - | 0.7393 | 0.7512 | 0.3986 | 0.0769 | 0.8921 | 0.8212 |
| | *G. barretti* | **0.0471** | 0.7314 | - | **0.0330** | 0.0879 | **0.0030** | 0.2887 | 0.8422 |
| | *H. panicea* | 0.2028 | 0.7337 | **0.0441** | - | **0.0020** | **0.0020** | 0.5255 | 0.2617 |
| | seawater | **0.0137** | 0.3974 | 0.0943 | **0.0003** | - | **0.0010** | **0.0210** | 0.2148 |
| | *M. lingua* | 0.1737 | 0.0777 | **0.0018** | **0.0023** | **0.0004** | - | **0.0080** | **0.0100** |
| | *M. rosacea* | 0.1391 | 0.9018 | 0.2558 | 0.5020 | **0.0258** | **0.0052** | - | 0.5135 |
| | *P. ventilabrum* | 0.0736 | 0.8256 | 0.8352 | 0.2621 | 0.2261 | **0.0082** | 0.5186 | - |
| Otu pa | *A. infundibuliformis* | - | 0.7662 | 0.1309 | 0.8542 | **0.0030** | **0.0280** | 0.7832 | **0.0010** |
| | *Axinella* sp. | 0.7742 | - | 0.7702 | 0.8791 | 0.3437 | 0.3537 | 0.7782 | 0.2887 |
| | *G. barretti* | 0.1207 | 0.7833 | - | **0.0490** | **0.0140** | **0.0120** | 0.0719 | **0.0080** |
| | *H. panicea* | 0.8715 | 0.8768 | 0.0574 | - | **0.0020** | **0.0120** | 0.5335 | **0.0020** |
| | seawater | **0.0025** | 0.3679 | **0.0124** | **0.0000** | - | **0.0010** | **0.0020** | 0.1389 |
| | *M. lingua* | **0.0212** | 0.3556 | **0.0039** | **0.0059** | **0.0002** | - | **0.0220** | **0.0030** |
| | *M. rosacea* | 0.7929 | 0.7893 | 0.0679 | 0.5278 | **0.0016** | **0.0292** | - | **0.0030** |
| | *P. ventilabrum* | **0.0013** | 0.2905 | **0.0100** | **0.0004** | 0.1435 | **0.0003** | **0.0018** | - |
| Species | *A. infundibuliformis* | - | 0.2108 | **0.0180** | 0.5924 | **0.0030** | 0.7752 | 0.4875 | **0.0190** |
| | *Axinella* sp. | 0.2251 | - | 0.4795 | 0.4725 | 0.3826 | 0.4416 | 0.5155 | 0.5784 |
| | *G. barretti* | **0.0096** | 0.5191 | - | **0.0400** | 0.4356 | **0.0360** | **0.0080** | 0.7383 |
| | *H. panicea* | 0.6152 | 0.4977 | **0.0296** | - | **0.0130** | 0.8472 | 0.8611 | 0.0559 |
| | seawater | **0.0042** | 0.3971 | 0.4679 | **0.0110** | - | **0.0210** | **0.0010** | 0.3237 |
| | *M. lingua* | 0.7638 | 0.4489 | **0.0294** | 0.8524 | **0.0123** | - | 0.6494 | 0.0569 |
| | *M. rosacea* | 0.4704 | 0.5168 | **0.0060** | 0.8372 | **0.0001** | 0.6673 | - | **0.0280** |
| | *P. ventilabrum* | **0.0150** | 0.5986 | 0.7626 | **0.0470** | 0.3381 | **0.0448** | **0.0150** | - |
| Genus | *A. infundibuliformis* | - | 0.6114 | **0.0020** | 0.0519 | **0.0050** | 0.4336 | 0.2008 | 0.1768 |
| | *Axinella* sp. | 0.5883 | - | 0.3726 | 0.7013 | 0.3407 | 0.9590 | 0.9321 | 0.8122 |
| | *G. barretti* | **0.0045** | 0.3444 | - | **0.0430** | 0.7582 | **0.0460** | 0.0050 | 0.2358 |
| | *H. panicea* | 0.0529 | 0.6795 | **0.0420** | - | **0.0300** | 0.2797 | 0.1748 | 0.8232 |
| | seawater | **0.0036** | 0.3231 | 0.7268 | **0.0256** | - | **0.0360** | **0.0020** | 0.2058 |
| | *M. lingua* | 0.4080 | 0.9650 | **0.0374** | 0.2714 | **0.0302** | - | 0.7263 | 0.6184 |
| | *M. rosacea* | 0.1988 | 0.9314 | **0.0028** | 0.1535 | **0.0011** | 0.7167 | - | 0.7403 |
| | *P. ventilabrum* | 0.1875 | 0.7993 | 0.2324 | 0.8119 | 0.2038 | 0.6059 | 0.7117 | - |
| Family | *A. infundibuliformis* | - | 0.5844 | **0.0100** | **0.0280** | **0.0030** | 0.2767 | **0.0230** | 0.1878 |
| | *Axinella* sp. | 0.5506 | - | 0.3846 | 0.6573 | 0.3377 | 0.9720 | 0.6733 | 0.8192 |
| | *G. barretti* | **0.0044** | 0.3901 | - | 0.0749 | 0.3257 | 0.0649 | 0.0569 | 0.2657 |
| | *H. panicea* | **0.0287** | 0.6344 | 0.0666 | - | **0.0330** | 0.3147 | 0.9431 | 0.7033 |
| | seawater | **0.0030** | 0.3452 | 0.3427 | **0.0352** | - | 0.0579 | **0.0270** | 0.2018 |
| | *M. lingua* | 0.2760 | 0.9702 | 0.0821 | 0.3240 | 0.0613 | - | 0.3307 | 0.7562 |
| | *M. rosacea* | **0.0315** | 0.6484 | 0.0611 | 0.9298 | **0.0329** | 0.3451 | - | 0.7532 |
| | *P. ventilabrum* | 0.1753 | 0.8178 | 0.2776 | 0.6919 | 0.2202 | 0.7410 | 0.7184 | - |
| Order | *A. infundibuliformis* | - | 0.4286 | **0.0110** | 0.0769 | **0.0100** | 0.2637 | **0.0400** | 0.4236 |
| | *Axinella* sp. | 0.4217 | - | 0.3866 | 0.7662 | 0.3267 | 0.9940 | 0.6583 | 0.9500 |
| | *G. barretti* | **0.0094** | 0.3911 | - | 0.2108 | 0.5554 | 0.1808 | 0.1149 | 0.1838 |
| | *H. panicea* | 0.0996 | 0.7810 | 0.2141 | - | 0.1339 | 0.6933 | 0.6993 | 0.6224 |
| | seawater | **0.0061** | 0.3360 | 0.5406 | 0.1406 | - | 0.1249 | **0.0500** | 0.1459 |
| | *M. lingua* | 0.2741 | 0.9913 | 0.1755 | 0.6808 | 0.1299 | - | 0.4505 | 0.9141 |
| | *M. rosacea* | **0.0381** | 0.6399 | 0.1201 | 0.6904 | **0.0413** | 0.4428 | - | 0.4595 |
| | *P. ventilabrum* | 0.3812 | 0.9414 | 0.1917 | 0.6220 | 0.1491 | 0.9077 | 0.4246 | - |
| Class | *A. infundibuliformis* | - | 0.1079 | **0.0490** | 0.2178 | **0.0180** | 0.2478 | 0.0929 | 0.4016 |
| | *Axinella* sp. | 0.1249 | - | 0.6733 | 0.7153 | 0.4216 | 0.7173 | 0.9800 | 0.5095 |
| | *G. barretti* | **0.0372** | 0.6570 | - | 0.3177 | 0.2108 | 0.3237 | 0.4715 | 0.1678 |
| | *H. panicea* | 0.2091 | 0.7105 | 0.2927 | - | 0.1638 | 0.9550 | 0.7153 | 0.7163 |
| | seawater | **0.0214** | 0.4358 | 0.2118 | 0.1733 | - | 0.1708 | 0.2178 | 0.0859 |
| | *M. lingua* | 0.2435 | 0.6882 | 0.3169 | 0.9543 | 0.1982 | - | 0.6454 | 0.7642 |
| | *M. rosacea* | 0.1011 | 0.9770 | 0.4749 | 0.6697 | 0.2506 | 0.6509 | - | 0.4156 |
| | *P. ventilabrum* | 0.3874 | 0.4844 | 0.1614 | 0.6930 | 0.1009 | 0.7526 | 0.4149 | - |
| Phylum | *A. infundibuliformis* | - | 0.1349 | **0.0470** | 0.1259 | **0.0220** | 0.1858 | 0.0539 | 0.0819 |
| | *Axinella* sp. | 0.1277 | - | 0.6663 | 0.8372 | 0.3347 | 0.8661 | 0.7233 | 0.9910 |
| | *G. barretti* | **0.0469** | 0.6546 | - | 0.1678 | 0.0559 | 0.5654 | 0.9600 | 0.4815 |
| | *H. panicea* | 0.1321 | 0.8250 | 0.1982 | - | **0.0460** | 0.9640 | 0.2757 | 0.7483 |
| | seawater | 0.0231 | 0.3585 | 0.0622 | 0.0561 | - | 0.2827 | 0.1948 | 0.1319 |
| | *M. lingua* | 0.1915 | 0.8397 | 0.5074 | 0.9617 | 0.2860 | - | 0.5734 | 0.8192 |
| | *M. rosacea* | 0.0508 | 0.6815 | 0.9579 | 0.2752 | 0.1988 | 0.5324 | - | 0.5514 |
| | *P. ventilabrum* | 0.0966 | 0.9855 | 0.4558 | 0.7135 | 0.1456 | 0.7941 | 0.5277 | - |

**Suppl. Table 5** Individual effects of depth on the prokaryotic community variance in *A. infundibuliformis* across all taxonomic ranks and OTUs (relative abundance & presence / absence). For each taxonomic rank the number of available variables and percentages of unclassified and therefore removed, reads are given next to each taxonomic rank, followed by nMDS stress values. Pairwise comparisons of group mean dispersions (permdisp: betadisper with permutest) are shown as *F* ratio (with degrees of freedom in brackets), correlation coefficient $R^2$ and *p* value. The same values are available for the permutational analysis of variance (permanova: adonis).

| | Dataset | Variables | nMDS stress | permdisp | | | permanova | | |
|---|---|---|---|---|---|---|---|---|---|
| | | | | F(1,4) | $R^2$ | *p* value | F(1,4) | $R^2$ | *p* value |
| ***A. infundibuliformis*** **shallow vs. upper twilight** | OTU relabund | 1182 | < 0.01 | 1.87 | 0.32 | 0.3 | 4.09 | 0.51 | 0.1 |
| | Species | 76 | < 0.01 | 1.35 | 0.25 | 0.4 | 10.69 | 0.73 | 0.1 |
| | Genus | 117 | < 0.01 | 0.25 | 0.06 | 0.6 | 9.04 | 0.69 | 0.1 |
| | Family | 96 | < 0.01 | 0.06 | 0.01 | 0.7 | 9.77 | 0.71 | 0.1 |
| | Order | 70 | < 0.01 | 0.15 | 0.04 | 0.8 | 8.57 | 0.68 | 0.1 |
| | Class | 45 | < 0.01 | 0.52 | 0.12 | 0.7 | 6.41 | 0.62 | 0.1 |
| | Phylum | 21 | < 0.01 | 0.46 | 0.10 | 0.7 | 5.61 | 0.58 | 0.1 |

**Suppl. Table 6** The relationships between richness & diversity and richness & evenness for sponge and seawater associated bacteria across high taxonomic ranks - from phylum to species level for each sponge taxon. *df* = degrees of freedom, $R^2$ = correlation coefficient, and *p* values for the taxon specific linear models.

| **Taxa** | **Richness** | | | **Evenness** | | |
|---|---|---|---|---|---|---|
| | ***df*** | $R^2$ | ***p* value** | ***df*** | $R^2$ | ***p* value** |
| *G. barretti* | 1,16 | 0.890 | 2.77E-09 | 1,16 | 0.394 | 0.003 |
| *M. lingua* | 1,16 | 0.850 | 3.36E-08 | 1,16 | 0.069 | 0.153 |
| *A. infundibu* | 1,34 | 0.798 | 1.40E-13 | 1,34 | 0.327 | 1.61E-04 |
| *P. ventilabru* | 1,16 | 0.943 | 1.46E-11 | 1,16 | 0.766 | 1.23E-06 |
| *Axinella* sp. | 1,16 | 0.915 | 3.56E-10 | 1,16 | 0.776 | 8.66E-07 |
| *M. rosacea* | 1,16 | 0.743 | 2.61E-06 | 1,16 | 0.317 | 0.009 |
| Seawater | 1,16 | 0.914 | 3.74E-10 | 1,16 | 0.781 | 7.04E-07 |
| *H. panicea* | 1,16 | 0.664 | 2.31E-05 | 1,16 | 0.195 | 0.038 |

**Suppl. Table 7** Individual effects of sample identity (individual sponge and seawater in triplicates) on the prokaryotic community variance across all taxonomic ranks and OTUs (relative abundance & presence / absence). For each taxonomic rank the number of available variables and percentages of unclassified and therefore removed, reads are given next to each taxonomic rank, followed by nMDS stress values. Pairwise comparisons of group mean dispersions (permdisp: betadisper with permutest) are shown as *F* ratio (with degrees of freedom in brackets), correlation coefficient $R^2$ and *p* value. The same values are available for the permutational analysis of variance (permanova: adonis).

| Datasets | Variables | Reads removed [%] | nMDS stress | permdisp | | | permanova | | |
|---|---|---|---|---|---|---|---|---|---|
| | | | | F(7,19) | $R^2$ | *p* value | F(7/19) | $R^2$ | *p* value |
| Species | 72 | 98.99 | 0.10 | 0.84[a] | 0.25 | 0.54 | 3.85[a] | 0.60 | **< 0.001** |
| Genus | 192 | 81.44 | 0.11 | 3.18 | 0.54 | **0.02** | 10.87 | 0.80 | **< 0.001** |
| Family | 163 | 51.30 | 0.11 | 1.63 | 0.38 | 0.17 | 14.47 | 0.84 | **< 0.001** |
| Order | 98 | 44.73 | 0.12 | 1.53 | 0.36 | 0.22 | 17.62 | 0.87 | **< 0.001** |
| Class | 61 | 22.80 | 0.09 | 1.82 | 0.40 | 0.13 | 20.37 | 0.88 | **< 0.001** |
| Phylum | 26 | 1.41 | 0.12 | 2.24 | 0.05 | 0.07 | 23.24 | 0.90 | **< 0.001** |

[a]F(7,18)

**Section 2: Sequence processing - quality control, filtering, and taxonomic assignments**

See Thomas *et al.*[1] for a complete description of the EMP sponge data processing: DNA was extracted from ~ 0.25 g of sponge tissue or sediment using the PowerSoil DNA Extraction kit (MoBio) according to the Earth Microbiome Project standard protocols (http://press.igsb.anl.gov/earthmicrobiome/emp-standard-protocols/dna- extraction-protocol/). Samples were extracted at laboratories at the University of Wuerzburg (Germany). Extracted DNA was shipped to the University of Colorado, Bolder, CO, USA for sequencing of the 16S rRNA gene using standard procedures of the Earth Microbiome Project (http://www.earthmicrobiome.org/emp-standard-protocols/ 16s/). Briefly, the V4 region of the 16S rRNA gene was amplified using the primer 515f–806rB and sequenced using the HiSeq2500 platform (Illumina)[2]. Raw Illumina reads were quality filtered and demultiplexed in mothur v.1.31.2 by following a modified version of the Miseq SOP[3]. Resulting sequences in fastq format were trimmed according to quality (average quality score = 30, window size = 5 bases, maximum number of homopolymers = 8, and trimmed to a minimum length of 100 base pairs). Sequences were aligned to the trimmed reference SILVA 102 database[4], which was provided by mothur. Chimeric sequences were detected using Uchime[5], and filtered out. Pairwise distances between aligned sequences were calculated (dist.seqs: cutoff = 0.05) and used for clustering. Prior to clustering, aligned sequences were phylogenetically classified based on the trimmed SILVA database. Sequences were clustered (cluster.split: splitmethod = classify, taxlevel = 4, cutoff = 0.03, hard = t, method = furthest) and converted to the shared file format (make.shared: label = 0.03). OTU representative sequences were clustered at 97% sequence similarity (get.oturep: label = 0.03) and classified based on Greengenes with a minimum cut-off of 60% identity (version gg_13_5_99 from May 2013). OTUs with single sequence across all samples (singletons) were removed. In addition, Chloroplasts sequences and OTUs with an abundance of less than 0.0001% across the dataset were removed. Also OTUs were removed from all sponge samples, if they had an abundance of > 0.01% in seawater or sediment. This last filter was applied to account for potential seawater or sediment contamination of the sponge samples.


1. Thomas, T. *et al.* Diversity, structure and convergent evolution of the global sponge microbiome. *Nat. Commun.* **7,** 11870 (2016).
2. Caporaso, J. G. *et al.* Global patterns of 16S rRNA diversity at a depth of millions of sequences per sample. *Proc. Natl. Acad. Sci.* **108,** 4516–4522 (2011).
3. Kozich, J. J., Westcott, S. L., Baxter, N. T., Highlander, S. K. & Schloss, P. D. Development of a dual-index sequencing strategy and curation pipeline for analyzing amplicon sequence data on the miseq illumina sequencing platform. *Appl. Environ. Microbiol.* **79,** 5112–5120 (2013).
4. Quast, C. *et al.* The SILVA ribosomal RNA gene database project: improved data processing and web-based tools. *Nucleic Acids Res.* **41,** D590–D596 (2013).
5. Edgar, R. C., Haas, B. J., Clemente, J. C., Quince, C. & Knight, R. UCHIME improves sensitivity and speed of chimera detection. *Bioinformatics* **27,** 2194–2200 (2011).


**Section 3: OTU table processing - from OTU to species level**

Individual amplicon abundance datasets were compiled in R by summing up the sequence reads for all available taxa and removing OTUs without approximate taxonomic classification from phylum to species level based on the available Greengenes taxonomy (see the R script below). However, the removal of unclassified sequence reads caused a considerable loss of taxonomic variables (i.e., taxa) at each rank (see Supplementary Table 7 online). For example, 98.99% of all reads at species level were removed, constituting in 72 variables for all preceding analyses at this rank. To counter this undesired loss of information unclassified reads were retained at each specific rank if the taxonomic designation of the preceding lower rank was available (see the R script below). This approach resulted in overall higher numbers of available variables at all taxonomic ranks (Table 2). For instance, at species level only 81.44% of the reads were removed, constituting in 241 taxonomic variables. Multivariate comparisons of both approaches, to ensure the viability of the dataset by retaining the sequence reads based on approximate taxonomy, showed that both types of datasets performed almost similar (Table 2 and Supplementary Table 7 online). However, especially the permanova results were overall slightly better for the datasets with an larger amount of available taxonomic variables.

The following R script comprises the above described OTU table transformations, and the basic alpha- and beta-diversity analyses. All figures and tables created for this study were derived from the output of the script below:

```
#Loading libraries
library(plyr)
library(vegan)
library(reshape)

#Loading data
# OTU abundance table with OTU taxonomy
read.csv(file="Suppl.DataEMPSwedenOTUabundancetaxonomy.csv", row.names=1, sep="\t") ->
        emp.sw # dataset: https://dx.doi.org/10.6084/m9.figshare.3470696

# Sample metadata for subsequent analyses
read.csv(file="emp.sw.habitat.csv", header=TRUE, sep="\t") -> emp.habitat # metadata:
        https://dx.doi.org/10.6084/m9.figshare.4299629.v1

# remove "unknown" OTUs
emp.sw <- droplevels(emp.sw[!grepl("unknown", emp.sw$Domain),])

##### creating taxonomic rank specific abundance tables

### phylum
emp.sw.phy <- ddply(emp.sw, .(Phylum) , numcolwise(sum)) # summarize at phylum level
emp.sw.phy <- emp.sw.phy[order(emp.sw.phy[,1]),] # sort taxa
rownames(emp.sw.phy) = emp.sw.phy[,1 ] # taxonomy as row.names
emp.sw.phy = emp.sw.phy[,-1 ] # remove first column
emp.sw.phy <- as.data.frame(t(emp.sw.phy)) # transpose dataset
emp.sw.phy$unclassified <- NULL # remove unclassified

### class
emp.sw.cla <- ddply(emp.sw, .(Class) , numcolwise(sum)) # summarize at class level
emp.sw.cla <- emp.sw.cla[order(emp.sw.cla[,1]),] # sort taxa
rownames(emp.sw.cla) = emp.sw.cla[,1 ] # taxonomy as row.names
emp.sw.cla = emp.sw.cla[,-1 ] # remove first column
emp.sw.cla <- as.data.frame(t(emp.sw.cla)) # transpose dataset
emp.sw.cla$unclassified <- NULL # remove unclassified

### order
emp.sw.ord <- ddply(emp.sw, .(Order) , numcolwise(sum)) # summarize at order level
emp.sw.ord <- emp.sw.ord[order(emp.sw.ord[,1]),] # sort taxa
rownames(emp.sw.ord) = emp.sw.ord[,1 ] # taxonomy as row.names
emp.sw.ord = emp.sw.ord[,-1 ] # remove first column
emp.sw.ord <- as.data.frame(t(emp.sw.ord)) # transpose dataset
emp.sw.ord$unclassified <- NULL # remove unclassified

#family
emp.sw.fam <- ddply(emp.sw, .(Family) , numcolwise(sum)) # summarize at family level
emp.sw.fam <- emp.sw.fam[order(emp.sw.fam[,1]),] # sort taxa
rownames(emp.sw.fam) = emp.sw.fam[,1 ] # taxonomy as row.names
emp.sw.fam = emp.sw.fam[,-1 ] # remove first column
emp.sw.fam <- as.data.frame(t(emp.sw.fam)) # transpose dataset
emp.sw.fam$unclassified <- NULL # remove unclassified

#genus
emp.sw.gen <- ddply(emp.sw, .(Genus) , numcolwise(sum)) # summarize at genus level
emp.sw.gen <- emp.sw.gen[order(emp.sw.gen[,1]),] # sort taxa
```

```
rownames(emp.sw.gen) = emp.sw.gen[,1 ] # taxonomy as row.names
emp.sw.gen = emp.sw.gen[,-1 ] # remove first column
emp.sw.gen <- as.data.frame(t(emp.sw.gen)) # transpose dataset
emp.sw.gen$unclassified <- NULL # remove unclassified

#species
emp.sw.spe <- ddply(emp.sw, .(Species) , numcolwise(sum))  # summarize at species level
emp.sw.spe <- emp.sw.spe[order(emp.sw.spe[,1]),] # sort taxa
rownames(emp.sw.spe) = emp.sw.spe[,1 ] # taxonomy as row.names
emp.sw.spe = emp.sw.spe[,-1 ] # remove first column
emp.sw.spe <- as.data.frame(t(emp.sw.spe)) # transpose dataset
emp.sw.spe$unclassified <- NULL # remove unclassified


##### creating taxonomic rank specific abundance tables with preceding taxa

emp.sw.pre <- emp.sw # copy the original OTU table

# create new taxonomy columns by combining preceding taxonomic information
emp.sw.pre <- within(emp.sw.pre, phylum <- paste(Domain, Phylum, sep='_'))
emp.sw.pre <- within(emp.sw.pre, class <- paste(Phylum, Class, sep='_'))
emp.sw.pre <- within(emp.sw.pre, order <- paste(Class, Order, sep='_'))
emp.sw.pre <- within(emp.sw.pre, family <- paste(Order, Family, sep='_'))
emp.sw.pre <- within(emp.sw.pre, genus <- paste(Family, Genus, sep='_'))
emp.sw.pre <- within(emp.sw.pre, species <- paste(Genus, Species, sep='_'))

# remove original columns
drops <- c("Domain","Phylum","Class","Order","Family","Genus","Species")
emp.sw.pre <- emp.sw.pre[ , !(names(emp.sw.pre) %in% drops)]

emp.sw.pre <- droplevels(emp.sw.pre) # drop unused levels from factors in a data frame - just in case

#phylum
ehl.pre.phy <- ddply(emp.sw.pre, "phylum", numcolwise(sum)) # summarize at phylum level
ehl.pre.phy <- ehl.pre.phy[order(ehl.pre.phy[,1]),] # sort taxa
rownames(ehl.pre.phy) = ehl.pre.phy[,1 ] # taxonomy as row.names
ehl.pre.phy = ehl.pre.phy[,-1 ] # remove first column
ehl.pre.phy <- as.data.frame(t(ehl.pre.phy)) # transpose dataset

#class
ehl.pre.cla <- ddply(emp.sw.pre, "class", numcolwise(sum)) # summarize at class level
ehl.pre.cla <- ehl.pre.cla[order(ehl.pre.cla[,1]),] # sort taxa
rownames(ehl.pre.cla) = ehl.pre.cla[,1 ] # taxonomy as row.names
ehl.pre.cla = ehl.pre.cla[,-1 ] # remove first column
ehl.pre.cla <- as.data.frame(t(ehl.pre.cla)) # transpose dataset
ehl.pre.cla$unclassified_unclassified <- NULL # remove unclassified

#order
ehl.pre.ord <- ddply(emp.sw.pre, "order", numcolwise(sum)) # summarize at order level
ehl.pre.ord <- ehl.pre.ord[order(ehl.pre.ord[,1]),] # sort taxa
rownames(ehl.pre.ord) = ehl.pre.ord[,1 ] # taxonomy as row.names
ehl.pre.ord = ehl.pre.ord[,-1 ] # remove first column
ehl.pre.ord <- as.data.frame(t(ehl.pre.ord)) # transpose dataset
ehl.pre.ord$unclassified_unclassified <- NULL # remove unclassified

#family
ehl.pre.fam <- ddply(emp.sw.pre, "family", numcolwise(sum)) # summarize at family level
ehl.pre.fam <- ehl.pre.fam[order(ehl.pre.fam[,1]),] # sort taxa
rownames(ehl.pre.fam) = ehl.pre.fam[,1 ] # taxonomy as row.names
```

```
ehl.pre.fam = ehl.pre.fam[,-1 ] # remove first column
ehl.pre.fam <- as.data.frame(t(ehl.pre.fam)) # transpose dataset
ehl.pre.fam$unclassified_unclassified <- NULL # remove unclassified

#genus
ehl.pre.gen <- ddply(emp.sw.pre, "genus", numcolwise(sum)) # summarize at genus level
ehl.pre.gen <- ehl.pre.gen[order(ehl.pre.gen[,1]),] # sort taxa
rownames(ehl.pre.gen) = ehl.pre.gen[,1 ] # taxonomy as row.names
ehl.pre.gen = ehl.pre.gen[,-1 ] # remove first column
ehl.pre.gen <- as.data.frame(t(ehl.pre.gen)) # transpose dataset
ehl.pre.gen$unclassified_unclassified <- NULL # remove unclassified

#species
ehl.pre.spe <- ddply(emp.sw.pre, "species", numcolwise(sum)) # summarize at species level
ehl.pre.spe <- ehl.pre.spe[order(ehl.pre.spe[,1]),] # sort taxa
rownames(ehl.pre.spe) = ehl.pre.spe[,1 ] # taxonomy as row.names
ehl.pre.spe = ehl.pre.spe[,-1 ] # remove first column
ehl.pre.spe <- as.data.frame(t(ehl.pre.spe)) # transpose dataset
ehl.pre.spe$unclassified_unclassified <- NULL # remove unclassified



#############
#### final dataframe preparation
#############

#OTU - rank free
df <- as.data.frame(t(emp.sw[,1:27]))

# choose one of the 12 following datasets for the analyses further below
#without preceding taxonomy
df <- emp.sw.phy
df <- emp.sw.cla
df <- emp.sw.ord
df <- emp.sw.fam
df <- emp.sw.gen
df <- emp.sw.spe
# the removal of unclassified variables at this rank caused a massive loss of data
# one complete sample is lost after applying: remove empty rows (samples) or columns (variables)
# some of the subsequent basic analyse may not work with this dataset

#with preceding taxonomy
df <- ehl.pre.phy
df <- ehl.pre.cla
df <- ehl.pre.ord
df <- ehl.pre.fam
df <- ehl.pre.gen
df <- ehl.pre.spe

# remove empty rows (samples) or columns (variables)
df <- droplevels(df[rowSums(df) != 0,])
df <- droplevels(df[,colSums(df) != 0])

#attach metadata
detach()
attach(emp.habitat)

#############
#### basic alpha and beta diversity analyses
```

```
#### relative abundance - Hellinger transformed - Bray-curtis
############

#hellinger transformation
deco.df <- decostand(df, method="hellinger")
apply(deco.df, 1, sum)

### Basic Alpha Diversity indices

emp.habitat$S <- specnumber(deco.df) # richness
emp.habitat$H <- diversity(deco.df) # Shannon
emp.habitat$J <- emp.habitat$H/log(emp.habitat$S) # Evenness

plot(emp.habitat$S ~ emp.habitat$Host_axi, las=2) # plot richness
plot(emp.habitat$H ~ emp.habitat$Host_axi, las=2) # plot Shannon
plot(emp.habitat$J ~ emp.habitat$Host_axi, las=2) # plot Evenness

### Basic Beta Diversity

# Bray-Curtis distance matrix - relative abundance
df.vegdist <- vegdist(deco.df, binary=F, method="bray")

# metaMDS
nMDS.df <- metaMDS(deco.df, distance="bray", k=2, autotransform=T, binary=F)
stressplot(nMDS.df)
nMDS.df

# empty plot
ordiplot(nMDS.df, type="n")
points(nMDS.df, dis="species", pch=19, cex=0.2, col="grey") # variables
points(nMDS.df, dis="sites", pch=19, cex=1, col="red") # samples
ordiellipse(nMDS.df, Host_axi, conf=0.85, cex=0.5, label = T, col="black") # ellipses

# multivariate betadispersion & adonis for "host" specificity
# betadisper permutest
site.bdis <- betadisper(df.vegdist, Host)
site.bdis
permu.anov <- permutest(site.bdis, pairwise=TRUE, perm=1000)
permu.anov
# permanova
adonis(df.vegdist ~ Host, perm=1000)


#############
#### indicator species analysis
#############

### Indicator species only at OTU level

library(indicspecies)

indval.g <- multipatt(deco.all, Phak, duleg = TRUE, func="IndVal.g", control = how(nperm=999))
summary(indval.g)
summary(indval.g, indvalcomp=TRUE)
summary(indval.g, alpha=0.05)
indval.g$sign
ls(indval.g)

indval.g <- multipatt(deco.all, Geod, duleg = TRUE, func="IndVal.g", control = how(nperm=999))
```

```
summary(indval.g)
summary(indval.g, indvalcomp=TRUE)
summary(indval.g, alpha=0.05)
indval.g$sign
ls(indval.g)

indval.g <- multipatt(deco.all, AxInf, duleg = TRUE, func="IndVal.g", control = how(nperm=999))
summary(indval.g)
summary(indval.g, indvalcomp=TRUE)
summary(indval.g, alpha=0.05)
indval.g$sign
ls(indval.g)

indval.g <- multipatt(deco.all, Axi, duleg = TRUE, func="IndVal.g", control = how(nperm=999))
summary(indval.g)
summary(indval.g, indvalcomp=TRUE)
summary(indval.g, alpha=0.05)
indval.g$sign
ls(indval.g)

indval.g <- multipatt(deco.all, Myca, duleg = TRUE, func="IndVal.g", control = how(nperm=999))
summary(indval.g)
summary(indval.g, indvalcomp=TRUE)
summary(indval.g, alpha=0.05)
indval.g$sign
ls(indval.g)

indval.g <- multipatt(deco.all, Myxi, duleg = TRUE, func="IndVal.g", control = how(nperm=999))
summary(indval.g)
summary(indval.g, indvalcomp=TRUE)
summary(indval.g, alpha=0.05)
indval.g$sign
ls(indval.g)

indval.g <- multipatt(deco.all, Hali, duleg = TRUE, func="IndVal.g", control = how(nperm=999))
summary(indval.g)
summary(indval.g, indvalcomp=TRUE)
summary(indval.g, alpha=0.05)
indval.g$sign
ls(indval.g)

indval.g <- multipatt(deco.all, mame, duleg = TRUE, func="IndVal.g", control = how(nperm=999))
summary(indval.g)
summary(indval.g, indvalcomp=TRUE)
summary(indval.g, alpha=0.05)
indval.g$sign
ls(indval.g)


#############
### multivariate analyses for OTU presence / absence data
#############

#Jaccard distance matrix with preceding pa decostand transformation
deco.df <- decostand(df, method="pa")
apply(deco.df, 1, sum)
df.vegdist <- vegdist(deco.df, binary=T, method="jaccard")

# beta-diversity
```

```
# metaMDS
nMDS.df <- metaMDS(deco.df, distance="jaccard", k=2, autotransform=T, binary=T)
stressplot(nMDS.df)
nMDS.df

# empty plot
ordiplot(nMDS.df, type="n")
points(nMDS.df, dis="species", pch=19, cex=0.2, col="grey") # variables
points(nMDS.df, dis="sites", pch=19, cex=1, col="red") # samples
ordiellipse(nMDS.df, Host_axi, conf=0.85, cex=0.5, label = T, col="black") # ellipses

# multivariate betadispersion & adonis for "host" specificity
# betadisper permutest
site.bdis <- betadisper(df.vegdist, Host)
site.bdis
permu.anov <- permutest(site.bdis, pairwise=TRUE, perm=1000)
permu.anov
# permanova
adonis(df.vegdist ~ Host, perm=1000)
```
